# Supplementary material for: Generic Workflow to Predict Medicine Concentrations in Human Milk Using Physiologically-Based Pharmacokinetic (PBPK) Modelling—A Contribution from the ConcePTION Project
Source: Pharmaceutics. 2023 May 11;15(5):1469. doi: 10.3390/pharmaceutics15051469 (PMC10223637; doi:10.3390/pharmaceutics15051469)
Supplement: Supplementary file 1 [file pharmaceutics-15-01469-s001.zip › File S1. Evaluation_report_amoxicillin.pdf]

# Building and evaluation of a Physiologically-Based PharmacoKinetic (PBPK) model for **amoxicillin** in adults and lactating women

*Disclaimer: The research project leading to these results was conducted as part of the ConcePTION consortium. This report only reflects the personal views of the stated authors. The results of this report are only intended for research purpose, and are not intended to be used in clinical practice.*

# Glossary

| | |
|---|---|
| AUC | Area Under the Curve |
| $C_{ave}$ | Average concentration |
| $CL_{re}$ | Reuptake clearance (i.e. from milk to blood) |
| $CL_{sec}$ | Secretion clearance (i.e. from blood to milk) |
| $C_{max}$ | Maximum (~peak) concentration |
| DID | Daily Infant Dosage (expressed for instance in mg/kg/day) |
| $f_u$ | Fraction unbound in plasma |
| GFR | Glomerular Filtration Rate |
| HBD | Hydrogen Bond Donors |
| IV | Intravenous (administration) |
| $LogD_{7.2}$ | Logarithm of the partition coefficient between an octanol phase and an aqueous (buffer) phase at pH 7.2 |
| $LogD_{7.4}$ | Logarithm of the partition coefficient between an octanol phase and an aqueous (buffer) phase at pH 7.4 |
| LogP | Logarithm of the partition coefficient between an octanol phase and (unbuffered) water as aqueous phase. This is the default parameter to express lipophilicity of a substance. |
| MD | Multiple dose |
| M/P ratio | Milk-to-Plasma ratio |
| MW | Molecular Weight (Da) |
| PBPK | Physiologically-Based PharmacoKinetic [*modeling*] |
| pKa | Logarithm of the acid dissociation constant |
| PO | Oral administration |
| PSA | Polar Surface Area |
| RID | Relative Infant Dose (%) |
| SD | Single dose |

# 1. Table of Contents

***1. Table of Contents*** ........ ***3***

***2. Introduction*** ........ ***4***

***3. Methods*** ........ ***5***

**3.1 Modelling strategy** ........ **5**

3.1.1 Reference PBPK models ........ 6

3.1.2 Lactation model ........ 6

**3.2 Data** ........ **6**

3.2.1 *In vitro* / physicochemical data ........ 6

3.2.2 Clinical data ........ 9

**3.3 Model Parameters and assumptions** ........ **12**

3.3.1 Absorption ........ 12

3.3.2 Distribution ........ 12

3.3.3 Metabolism and excretion ........ 12

3.3.4 Secretion to milk ........ 12

3.3.5 Automated parameter optimization ........ 13

**3.4 Infant dosage calculation** ........ **14**

***4. Results*** ........ ***14***

**4.1 Final input parameters** ........ **14**

**4.2 Diagnostics plots** ........ **15**

**4.3 Concentration-time profiles** ........ **17**

4.3.1 Model building ........ 18

4.3.2 Model verification ........ 23

4.3.3 Lactation PBPK model ........ 25

**4.4 Estimated infant dosage** ........ **31**

***5. Discussion*** ........ ***32***

***6. Conclusion*** ........ ***32***

***7. List of Appendix and Supplementary Materials*** ........ ***33***

***8. References*** ........ ***33***

## 2. Introduction

Amoxicillin (figure S1) is a penicillin derivative that belongs to the class of broad-spectrum antibiotics. In clinical practice, it is frequently combined with the beta-lactamase inhibitor clavulanic acid. The usual dose of amoxicillin is 250 mg to 500 mg taken 3 times a day. In combination with clavulanate the amoxicillin dose amounts to 875 mg (with 125 mg clavulanate) and is taken twice daily. The bioavailability of amoxicillin is about 88 % in humans [1]. Amoxicillin is 17 % bound to plasma proteins. Amoxicillin is predominantly (60-80 %) excreted unchanged in the urine via glomerular filtration and tubular secretion. A small fraction is metabolized to penicilloic acid.

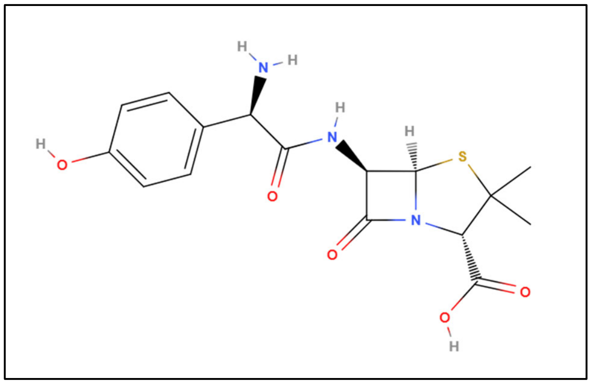


*Figure S1 Chemical Structure of amoxicillin*

The scope of this report is to:

(a) specify the details and underlying assumptions associated with the building of physiologically-based pharmacokinetic (PBPK) models for amoxicillin in adult healthy volunteers or patients, and in postpartum women during lactation.

(b) evaluate the predictive performance of these PBPK models. This is achieved by comparing model-predicted plasma or milk concentrations with corresponding clinical observations.

# 3. Methods

The software used for the development of PBPK models presented in this report is tabulated below:

| Software | Version |
|---|---|
| PK-Sim® | v9.1 |
| MoBi® | v9.1 |

## 3.1 Modelling strategy

In the present report, a reference PBPK model was first established for adults (patients as well as healthy volunteers), and subsequently verified against clinical pharmacokinetic data reported for amoxicillin in the scientific literature.

Relevant information on the anthropometry (height, weight) was gathered from the respective clinical studies, if reported. Information on physiological parameters (e.g. blood flows, organ volumes, hematocrit) in adults is available in the PK-Sim® database.

In a second step, a lactation PBPK model was developed, based on the general workflow described by Dallmann *et al. 2018* [1–3].

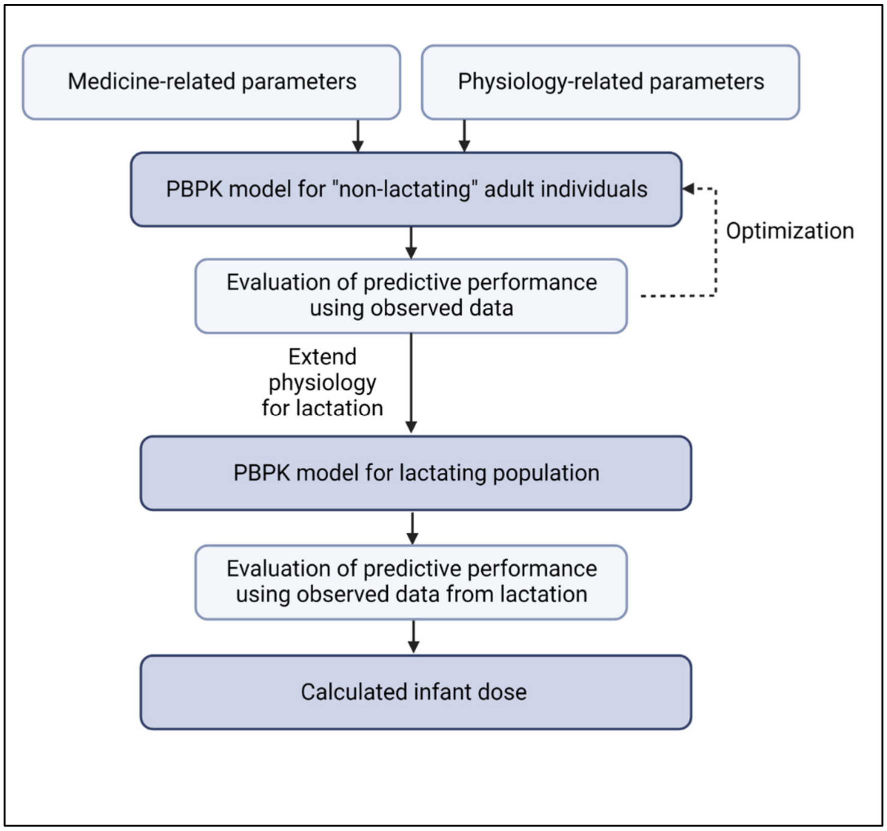


*Figure S2 General workflow that was used in the present project to develop and evaluate the lactation PBPK model*

Details about input data (physicochemical, *in vitro* and clinical data) can be found in section 3.2. Details about the structural models and their parameters can be found in section 3.3.

### 3.1.1 Reference PBPK models

The reference PBPK models were built based on studies with adult volunteers and/or adult patients, using the reported mean values for age, weight, height, and genetic background as described in each study protocol. When no information on these parameters could be found, a healthy male European individual, 30 years of age, with a body weight of 73 kg and a height of 176 cm was used.

The abundance (including population variability) of plasma proteins and enzymes/transporters that are integrated into PK-Sim are described in the publicly available 'PK-Sim Ontogeny Database Version 7.3' (PK-Sim Ontogeny Database Version 7.3).

Glomerular filtration and active first-order tubular secretion were enabled, as these represent the major mechanisms for amoxicillin renal excretion.

Structural model selection was mainly guided by biological plausibility and by visual inspection of the predicted concentration time profiles in comparison with observed data. The generally applied acceptance criterium was less than 2-fold misprediction. Uninformed parameter values (see below) were estimated using the parameter identification module of PK-Sim®.

The predictive performance of the models was evaluated by simulating:
- Single intravenous dose studies
- Single and multiple oral dose studies

For some parameters, parameter optimization was performed as described below to obtain improved concordance between predicted profiles and observed data.

### 3.1.2 Lactation model

After development of the reference model, the model was exported to MoBi® and a lactation PBPK model was constructed. To model the passage of amoxicillin into human milk, i.e. across the blood/milk biological barrier, both the secretion ($CL_{sec}$) and reuptake clearance ($CL_{re}$) values were obtained using the empirical model developed by Koshimichi *et al. 2011* [4].

## 3.2 Data

### 3.2.1 *In vitro* / physicochemical data

A literature search was performed to collect available information on physicochemical properties of amoxicillin. The obtained information from literature is summarized in Table S1. Table S2 shows the parameters that were additionally used for the lactation PBPK model.

*Table S1 Physicochemical parameters used as input for the amoxicillin PBPK models*

| **Parameter** | **Value** | **Unit** | **Description** | **Source** |
|---|---|---|---|---|
| MW | 365.40 | g/mol | Molecular weight | [1] |
| $pK_a$ | 3.23 (acid)<br>7.43 (base) | | Logarithm of the acid dissociation constant | |

| | | | | |
|---|---|---|---|---|
| Solubility (pH 7) | 3.40 | mg/mL | Aqueous solubility | [5] |
| LogP | 0.87 | - | $Log_{10}$ of the partition coefficient between octanol and water (~lipophilicity) | [1] |
| $f_u$ | 0.85 | - | Fraction unbound in human plasma | |
| **Liver plasma clearance** – "specific clearance" | 0.10 | $min^{-1}$ | Rate constant describing intrinsic hepatic plasma clearance (= $CL_{int, hep}$ normalized for liver volume) | |
| **Tubular secretion** – "TSspec" | 0.10 | $min^{-1}$ | Rate constant for the active tubular secretion process | |
| GFR fraction | 1.00 | - | Fraction of the glomerular filtration rate used for passive renal elimination | |

*Table S2 Physicochemical parameters used as input for the lactation PBPK model of amoxicillin*

| Parameter | Value | Unit | Description | Source |
|---|---|---|---|---|
| Milk logP[a] | 0.87 | - | $Log_{10}$ of the partition coefficient between octanol and water | [1] |
| | -0.13 | - | LogP of nonionic species | MarvinSketch |
| $LogD_{7.2}$ | -3.17 | | $Log_{10}$ of the partition coefficient between octanol and water at pH 7.2 | MarvinSketch |

| | | | | |
|---|---|---|---|---|
| $LogD_{7.4}$ | -3.25 | | $Log_{10}$ of the partition coefficient between octanol and water at pH 7.4 | MarvinSketch |
| HBD | 4.00 | - | Hydrogen bond donors | Pubchem |
| PSA | 158 | Å$^2$ | Polar surface area | Pubchem |

[a] Milk logP is $Log_{10}$ of the partition coefficient between octanol and water and is used as input for the calculations in the postpartum model (see equations below). In theory, this value is identical to the logP specified in Table S1. However, in some PBPK models, logP (Table S1) might be optimized using parameter identication. Therefore, it was chosen to use a separate parameter (i.e. Milk logP) to represent the logP used as input for the equations in the postpartum model.

The default equations for free fraction in human milk and logD that were implemented in the spatial structure building block that was developed for the postpartum women are described below. Alternatively, these values can be overwritten by values calculated elsewhere (e.g. MarvinSketch) or determined *in vitro.*

The free fraction in human milk was calculated with the equations proposed by Atkinson and Begg [6], as follows:

$$f_{u\_skimmed\ milk} = \frac{f_u \times 0.448}{(0.000694^{0.448} + \mathrm{f}_\mathrm{u}^{0.448})}$$

$$P_{milk} = 10^{(-0.88+1.29\times \mathrm{logD7.2})}$$

$$\text{Total free fraction in milk} = \frac{1}{(\frac{0.955}{f_{u\_skimmed\ milk}} + 0.045 \times P_{milk})}$$

Where: $f_u$ *skimmed milk:* binding to proteins in milk; $P_{milk}$: partitioning between aqueous and lipid phase of milk; Total free fraction in milk: 'total' free fraction, i.e. accounting for both protein and lipid binding processes.

LogD values taking into account up to three pka values (as provided in the compound building block), were calculated as follows:

$$LogD = \mathrm{Log}\,P + Log_{10}(logD_{factor})$$

*With Milk logP (Table S2) as input for logP*

$$\begin{aligned} LogD_{factor} = {} & \mathrm{K}_1 + (\mathrm{K}_2 + \mathrm{K}_3 + \mathrm{K}_4) \times \mathrm{base}^1 + \mathrm{K}_5 \times \mathrm{base}^{\max(\mathrm{CT}_0+\mathrm{CT}_1;-\mathrm{CT}_0-\mathrm{CT}_1)} \\ & + \mathrm{K}_6 \times \mathrm{base}^{\max(CT_0+CT_2;-CT_0-CT_2)} + \mathrm{K}_7 \times \mathrm{base}^{\max(\mathrm{CT}_2+\mathrm{CT}_1;-\mathrm{CT}_2-\mathrm{CT}_1)} \\ & + \mathrm{K}_8 \times \mathrm{base}^{\max(\mathrm{CT}_0+\mathrm{CT}_1+\mathrm{CT}_2;-\mathrm{CT}_0-\mathrm{CT}_1-\mathrm{CT}_2)} \end{aligned}$$

$K_1 = F_1 \times F_2 \times F_3$
$K_2 = (1 - F_1) \times F_2 \times F_3$
$K_3 = F_1 \times (1 - F_2) \times F_3$
$K_4 = F_1 \times F_2 \times (1 - F_3)$
$K_5 = (1 - F_1) \times (1 - F_2) \times F_3$
$K_6 = (1 - F_1) \times F_2 \times (1 - F_3)$
$K_7 = (1 - F_1) \times F_2 \times (1 - F_3)$
$K_8 = (1 - F_1) \times (1 - F_2) \times (1 - F_3)$

F1 = CT0 != CT_NEUTRAL ?  1/(1+10^(CT0*(pKa_0- pH))) : 1
F2 = CT1 != CT_NEUTRAL ?  1/(1+10^(CT1*(pKa_1- pH))) : 1
F3 = CT2 != CT_NEUTRAL ?  1/(1+10^(CT2*(pKa_2- pH))) : 1

*With CT = compound type (-1: acid; +1: base; 0: neutral), and pH = 7.2 or 7.4 respectively for* $logD_{7.2}$ *and* $logD_{7.4}$

The transports that were added in the passive transport building block for 'transfer to milk' and 'transfer from milk' are based on secretion and reuptake and clearance values, $Cl_{sec}$ and $Cl_{re}$, which were calculated according to the empirical equations proposed by Koshimichi et al. 2011 [4], as follows:

$$\text{Log}\, CL_{re} = 2.793 + 0.179 \times LogP - 0.132 \times HBD$$

$$\text{Log}\, CL_{sec} = 3.367 \times Log10(MW) - 0.164 \times (LogP - LogD) - 0.015 \times PSA - 3.912$$

### 3.2.2 Clinical data

Literature searches were performed to collect available data on amoxicillin in adults and postpartum women. The amoxicillin reference PBPK model was developed using different clinical studies with pharmacokinetic (PK) blood sampling. In the first iteration, the model framework described by Dallmann *et al.* 2020 was adopted [1]. This model was built on 6 clinical studies where amoxicillin was administered intravenously in reference individuals [7–12]. The model was extended for oral administration based on 2 clinical trials with administration of 250 mg, 2x500 mg and 1000 mg [12,13]. Finally, 3 clinical trials with a single oral administration (500 or 1000 mg), and 1 clinical trial with multiple doses were used for verification [7,11,14,15].

The evaluation of the predictive performance of the amoxicillin lactation PBPK model was performed using a study where amoxicillin was administered as a single oral dose of 2x500 mg to 6 lactating women [16]. The women were 3 days postpartum. Unfortunately, no other clinical data were available for amoxicillin during lactation.

Detailed information and data from the studies used for model building, verification, and lactation model can be found in Supplementary material 1 and 2.

#### *3.2.2.1 Model building*

The studies that were used for model building are shown in

Table S3 (**training data**).

*Table S3 Summary of studies used for PBPK model building of amoxicillin in reference populations*

| Study ID | Reference | Arm/treatment/information used for model building |
|---|---|---|
| Arancibia 1980 | [7] | 9 subjects received IV 1000 mg (single dose) |
| Arancibia 1982 | [8] | 9 subjects received IV 1000 mg (single dose) |
| Mastrandrea 1984 | [9] | 10 subjects received IV 1000 mg (single dose) |
| Tan 1981 | [10] | 6 subjects received IV 1000 mg (single dose) |
| Witkowski 1982 | [11] | 8 subjects IV 500 mg (single dose) |
| Zarowny 1974 | [12] | 8 subjects received IV 250 mg (single dose) |
| Zarowny 1974 | [12] | 8 subjects received PO 250 mg (single dose) |
| Zaid 2010 | [13] | 24 subjects received PO 1000 mg (single dose) |
| Zaid 2010 | [13] | 24 subjects received PO 2x500 mg (single dose) |

*Table S4 Demographic information*

| Study ID | Reference | Number of subjects (female ratio) | Age (year) | Weight (kg) |
|---|---|---|---|---|
| Arancibia 1980 | [7] | 9 (0.22) | 28.3 (21-45) | 66.4 (46-88) |
| Arancibia 1982 | [8] | 9 (0) | 31.2 (22-46) | 65.2 (56-74) |
| Mastrandrea 1984 | [9] | 10 (0) | - (21-36) | - |
| Tan 1981 | [10] | 6 (-) | - (19-47) | - |
| Witkowski 1982 | [11] | 10 (0.50) | 32.3 (25-44) | 67.4 (51-86) |
| Zarowny 1974 | [12] | 8 (0) | - (20-30) | 74.5 (59-91) |
| Zaid 2010 | [13] | 24 (0) | - (17-30) | - (59-85) |

### *3.2.2.2 Model verification*

The studies that were used to evaluate the predictive performance of the PBPK model are shown in

Table S5 (**verification data**).

*Table S5 Summary of studies used for model verification of amoxicillin PBPK model in reference population*

| Study ID | Reference | Arm/treatment/information used for model verification |
|---|---|---|
| Arancibia 1980 | [7] | 9 subjects received PO 500 mg (single dose) |
| Burkhardt 2002 | [14] | 12 subjects received PO 1000 mg co-amoxiclav (multiple dose) |
| Pires de Abreu 2003 | [15] | 24 subjects received PO 500 mg Std (single dose) |
| Pires de Abreu 2003 | [15] | 24 subjects received PO 500 mg Tst (single dose) |
| Witkowski 1982 | [11] | 10 subjects received PO 500 mg (single dose) |
| Witkowski 1982 | [11] | 10 subjects received PO 500 mg augmentin (single dose) |

*Table S6 Demographic information*

| Study ID | Reference | Number of subjects (female ratio) | Age (year) | Weight (kg) |
|---|---|---|---|---|
| Arancibia 1980 | [7] | 9 (0.22) | 28.3 (21-45) | 66.4 (46-88) |
| Burkhardt 2002 | [14] | 12 (0.5) | 31.75 (23-39) | 73.7 (-) |
| Pires de Abreu 2003 | [15] | 24 (0.5) | - (21-47) | - (55-59) |
| Witkowski 1982 | [11] | 10 (0.50) | 32.3 (25-44) | 67.4 (51-86) |

#### *3.2.2.3 Lactation PBPK model*

Table S7 shows the study that was used for the lactation PBPK model.

*Table S7 Summary of study used for PBPK model development of amoxicillin in lactating women*

| Study ID | Publication | Arm/treatment/information used for model building and verification |
|---|---|---|
| Kafetzis 1981 | [16] | 6 women (3 days postpartum) received PO 2x500 mg (single dose) |

## 3.3 Model Parameters and assumptions

### 3.3.1 Absorption

The release of amoxicillin from the capsule or tablet was implemented as a Weibull function. Dissolution shape and time, together with transcellular intestinal permeability were estimated during parameter optimization.

### 3.3.2 Distribution

An important parameter influencing the distribution of a compound is lipophilicity. Lipophilicity (Table S1) was optimized in the original model from Dallmann et al. 2020 [1]. The tissue partition coefficients ($K_p$) calculation was according to 'Rodgers and Rowland' and the cellular permeability calculation was 'PK-Sim Standard'.

### 3.3.3 Metabolism and excretion

The final model applies specific hepatic clearance, active tubular secretion and glomerular filtration (see Table S1). The optimized values for specific hepatic clearance and tubular secretion were 0.06 and 0.89 $min^{-1}$, respectively. The glomerular filtration was set to its default value of 1.

### 3.3.4 Secretion to milk

To model the transfer process of amoxicillin into human milk, both the secretion ($CL_{sec}$) and reuptake clearance ($CL_{re}$) were calculated using the empirical equations developed by Koshimichi *et al.* 2011 (see 3.2.1 *In vitro* / physicochemical data) [4].

First, in MoBi®, a spatial structure for the postpartum women was constructed, similar to the workflow from Dallmann *et al.* 2018 [2]. Here, breasts were added as a compartment. In addition, the human milk was connected to the plasma subcompartment of the breasts. The

human milk volume was specified as 0.5 L to represent the structure of Koshimichi *et al.* 2011, and a geometric standard deviation of 1.16 was assumed in the population. The free fraction in human milk, and logD values were implemented as the equations described previously. The transfer between plasma and milk was defined as two kinetic processes (transfer to milk and transfer from milk) under passive transports (see below). Next, the simulation was combined with the postpartum population from Job *et al.* 2021 in PK-Sim to account for the postpartum physiology [3].

<u>*Kinetics*</u>

*Transfer to milk*

$$\frac{dNmilk}{dt} = Cplasma \times f_u \times CL_{sec}$$

where Cplasma is the concentration in plasma (in breast compartment), $f_u$ is the free fraction in plasma and $CL_{sec}$ is the secretion clearance.

*Transfer from milk*

$$\frac{dNplasma}{dt} = Cmilk \times f_u \times CL_{re}$$

where Cmilk is the concentration in human milk, $f_u$ is the total free fraction in human milk (protein and lipid) and $CL_{re}$ is the reuptake clearance.

The median simulated plasma and human milk concentration-time profiles can be used to calculate the M/P ratio as follows:

$$M/P\ ratio\ = \frac{AUC_{milk}}{AUC_{plasma}}$$

### 3.3.5 Automated parameter optimization

The following table depicts the results of the final parameter optimization according to the different clinical studies.

a) Intravenous single dose administrations from Arancibia 1980, Arancibia 1982, Mastrandrea 1984, Tan 1981, Witkowski 1982 and Zarowny 1974 [7–12]:
:

| Model parameter | Optimized value | Unit |
|---|---|---|
| Lipophilicity (LogP) | -0.44 | - |
| TSspec | 0.71 | $min^{-1}$ |
| Specific clearance | 0.05 | $min^{-1}$ |

b) Zaid 2010 1000 mg PO; Zaid 2010 2x500 mg PO; Zarowny 1974 250 mg PO [12,13]

| Model parameter | Optimized value | Unit |
|---|---|---|
| Specific intestinal permeability (transcellular) | 7.08E-6 | cm/min |
| Dissolution shape | 0.75 | |
| Dissolution time (50% dissolved) | 33.82 | min |

## 3.4 Infant dosage calculation

Infant dosage via human milk was then calculated based on the predicted (average and maximal) steady-state amoxicillin concentration in human milk, as well as the daily milk intake volume. The daily infant dosage was then compared to the maternal dosage, resulting in the relative infant dose (RID).

$$Daily\ infant\ dosage = C_{average} * 150\ \frac{mL}{kg\ .day}$$

$$Daily\ infant\ dosage = C_{max} * 150\ \frac{mL}{kg\ .day}$$

$$Relative\ infant\ dose\ (RID) = \frac{Infant\ dosage}{Maternal\ dosage} * 100\ \%$$

# 4. Results

Both the reference and postpartum PBPK model of amoxicillin was developed and verified with clinical PK data.

The models were evaluated covering studies including in particular:

- Intravenous and oral administration
- Single and multiple doses
- A dose range from 250 up to 1000 mg
- Paired milk/plasma data

The model describes the elimination of amoxicillin renally via hepatic clearance, tubular secretion and glomerular filtration. Moreover, secretion and reuptake to human milk were described by $CL_{sec}$ and $CL_{re}$.

The next sections show:

- The final model parameters for the building blocks: section 4.1
- The overall predictive performance: section 4.2
- The simulated versus observed concentration-time profiles for the clinical studies used for model building and for model verification: section 4.3

## 4.1 Final input parameters

The compound values of the final postpartum PBPK model for amoxicillin are illustrated below.

**Physicochemical parameters**

| Parameter | Value | Unit | Source |
| --- | --- | --- | --- |
| MW | 365.40 | g/mol | [1] |
| pKa | 3.23 (acid)<br>7.43 (base) | - | |
| Solubility | 3.40 | mg/mL | |

| | | | |
|---|---|---|---|
| Lipophilicity | -0.44 | - | |
| $f_u$ | 0.85 | - | |
| Small molecule (Y/N) | Yes | - | - |

**Calculation methods**

| Name | Value |
|---|---|
| Tissue partition coefficients | Rodgers and Rowland |
| Cellular permeabilities | PK-Sim Standard |

**AMDE-related parameters**

| Parameter | Value | Unit | Source |
|---|---|---|---|
| Intestinal permeability | 7.08E-6 | cm/min | Parameter identification |
| Specific clearance | 0.06 | $min^{-1}$ | [1] |
| Specific tubular secretion | 0.89 | $min^{-1}$ | |
| GFR fraction | 1 | - | |

**Formulation-related parameters**

| Parameter | Value | Unit | Source |
|---|---|---|---|
| Dissolution shape | 0.75 | - | Parameter identification |
| Dissolution time | 33.82 | min | |

**Physicochemical and physiological parameters relevant to the lactation model**

| Parameter | Value | Unit | Source |
|---|---|---|---|
| Milk logP | 0.87 | - | [1] |
| HBD | 4.00 | - | Pubchem |
| PSA | 158 | $Å^2$ | Pubchem |
| $CL_{sec}$ | 7.82E-4 | L/min | Default |
| $CL_{re}$ | 4.39E-3 | L/min | Default |
| $f$u_skimmed milk[a] | 0.96 | - | Default |
| Pmilk[b] | 1.07E-5 | - | Default |
| Total free fraction in milk[c] | 1.01 | - | Default |
| $logD_{7.2}$ | -3.17 | - | MarvinSketch |
| $logD_{7.4}$ | -3.25 | - | MarvinSketch |

[a] binding to proteins in milk; [b] partitioning between aqueous and lipid phase of milk; [c] total free fraction, accounting for both protein and lipid binding

## 4.2 Diagnostics plots

The geometric mean fold errors (GMFE) on AUC and Cmax were 1.15 and 1.23 for the model building dataset, and 1.24 and 1.17 for the model verification dataset.

The following plot shows the predictive performance graph for Cmax and AUC of amoxicillin for the PBPK model performance of all data used.
Predicted over observed ratio values of all data listed in section 3.2.2 are presented below.

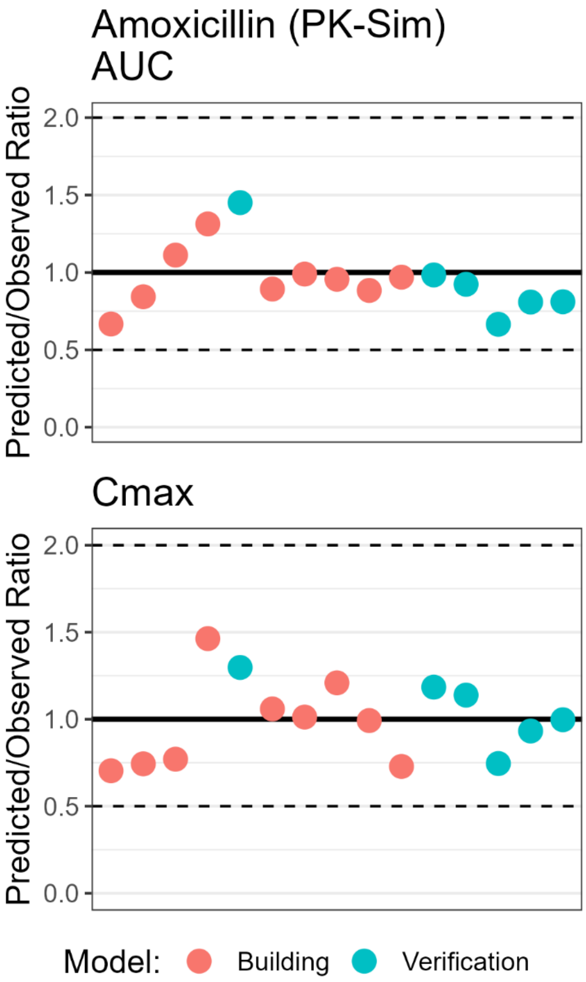


*Figure S3. Predicted over observed ratio profile*

*Table S8 Ratio between the predicted and observed pharmacokinetic parameters of amoxicillin in different dosing regimens for model building*

| Study ID/ Reference | Dose/ Route | $AUC_{obs}$ (mg*h/L) | $AUC_{pred}$ (mg*h/L) | Fold error | $C_{max\ obs}$ (mg/L) | $C_{max\ pred}$ (mg/L) | Fold error |
|---|---|---|---|---|---|---|---|
| Arancibia 1980 | 500 mg IV SD | 33.65 | 22.45 | 0.67 | 42.60 | 29.98 | 0.70 |
| Arancibia 1982 | 1000 mg IV MD | 53.63 | 45.22 | 0.84 | 79.50 | 59.11 | 0.74 |
| Mastrandrea 1984 | 1000 mg IV MD | 41.53 | 46.19 | 1.11 | 74.10 | 57.08 | 0.77 |
| Tan 1981* | 1000 mg IV MD | 13.75 | 18.06 | 1.31 | 13.16 | 19.26 | 1.46 |

| | | | | | | | |
|---|---|---|---|---|---|---|---|
| Witkowski 1982 | 500 mg IV SD | 23.49 | 22.78 | 0.97 | 40.58 | 29.57 | 0.73 |
| Zarowny 1974 | 250 mg IV SD | 11.49 | 11.37 | 0.99 | 10.48 | 10.61 | 1.01 |
| Zarowny 1s974 | 250 mg PO SD | 9.56 | 9.14 | 0.96 | 2.87 | 3.47 | 1.21 |
| Zaid 2010 | 1000 mg PO SD | 39.69 | 35.44 | 0.89 | 12.21 | 12.93 | 1.06 |
| Zaid 2010 | 2x500 mg PO SD | 39.38 | 34.81 | 0.88 | 12.89 | 12.79 | 0.99 |

*R was used to censor data before 1h (i.e. before the first observed datapoint), for calculation of the predicted AUC and $C_{max}$

*Table S9 Ratio between the predicted and observed pharmacokinetic parameters of amoxicillin in different dosing regimens used for model verification*

| Study ID/ Reference | Dose/ Route | $AUC_{obs}$ (mg*h/L) | $AUC_{pred}$ (mg*h/L) | Fold error | $C_{max\ obs}$ (mg/L) | $C_{max\ pred}$ (mg/L) | Fold error |
|---|---|---|---|---|---|---|---|
| Arancibia 1980 | 500 mg PO SD | 27.07 | 18.01 | 0.67 | 9.50 | 7.08 | 0.75 |
| Burkhardt 2002 | 1000 mg PO MD | 26.44 | 38.37 | 1.45 | 9.38 | 12.17 | 1.30 |
| Pires de Abreu 2003 | 500 mg PO Std SD | 21.31 | 17.24 | 0.81 | 7.25 | 6.76 | 0.93 |
| Pires de Abreu 2003 | 500 mg PO Tst SD | 21.26 | 17.24 | 0.81 | 6.78 | 6.76 | 1.00 |
| Witkowsk 1982 | 500 mg PO SD (1) | 18.85 | 18.55 | 0.98 | 5.97 | 7.07 | 1.18 |
| Witkowsk 1982 | 500 mg PO SD (2) | 20.08 | 18.55 | 0.92 | 6.21 | 7.07 | 1.14 |

## 4.3 Concentration-time profiles

Simulated versus observed concentration-time profiles of all data listed in section 3.2.2 are presented below. The original.pksim5 are provided in Supplemented material 3.

### 4.3.1 Model building

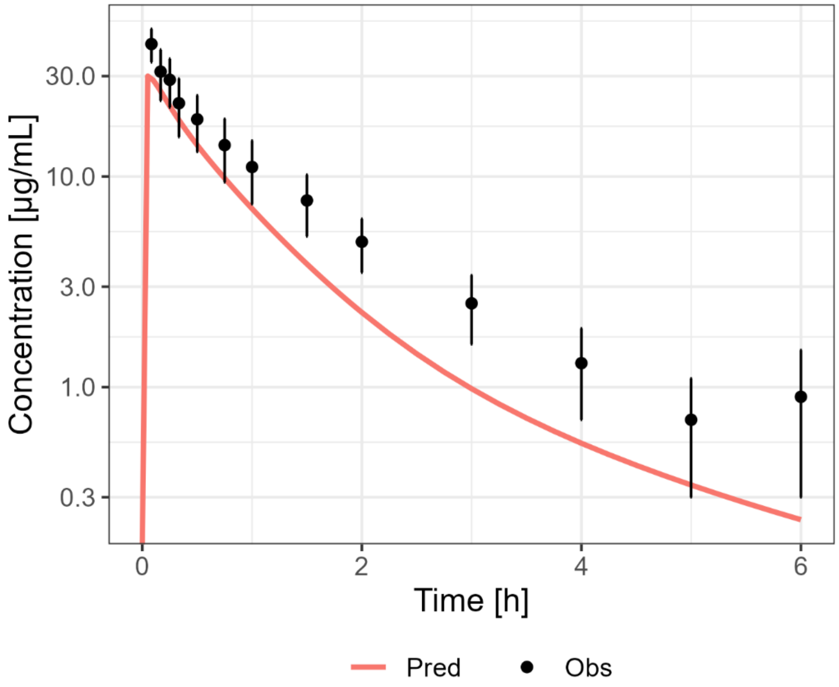


*Figure S4 Predicted (Pred) versus observed (Obs) concentration-time profile after administration of 500 mg IV SD* [7]

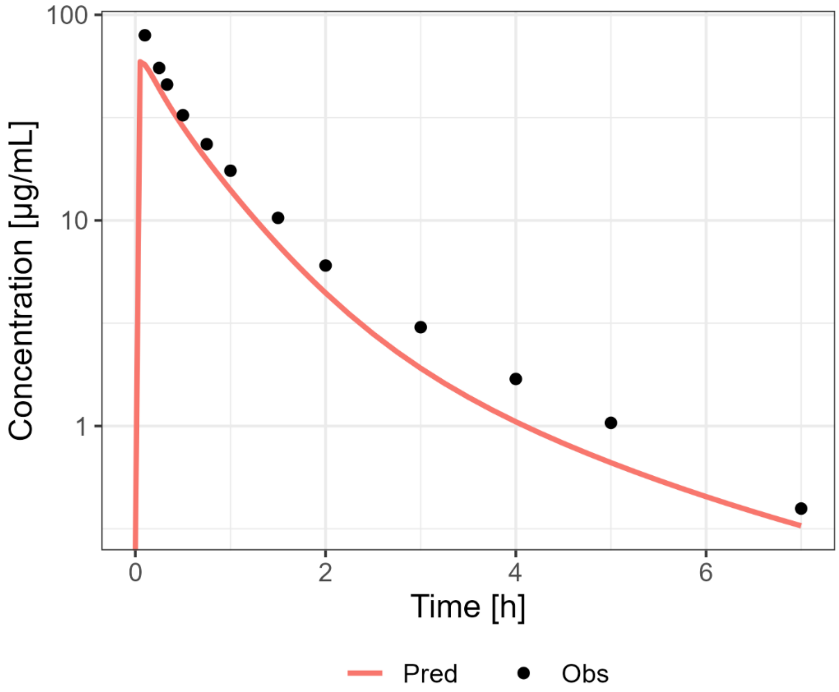


*Figure S5 Predicted (Pred) versus observed (Obs) concentration-time profile after administration of 1000 mg IV SD* [8]

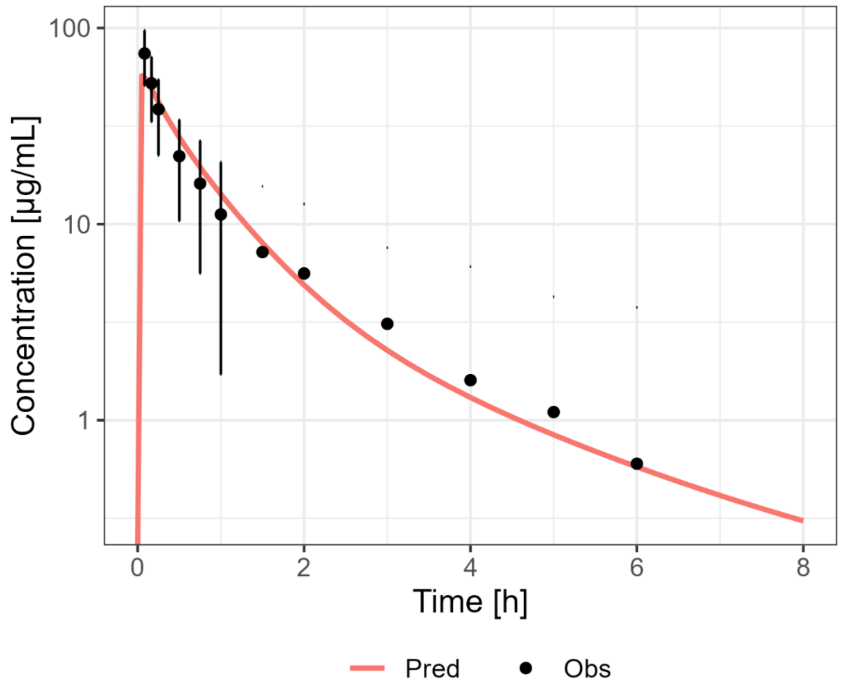


*Figure S6 Predicted (Pred) versus observed (Obs) concentration-time profile after administration of 1000 mg IV* [9]

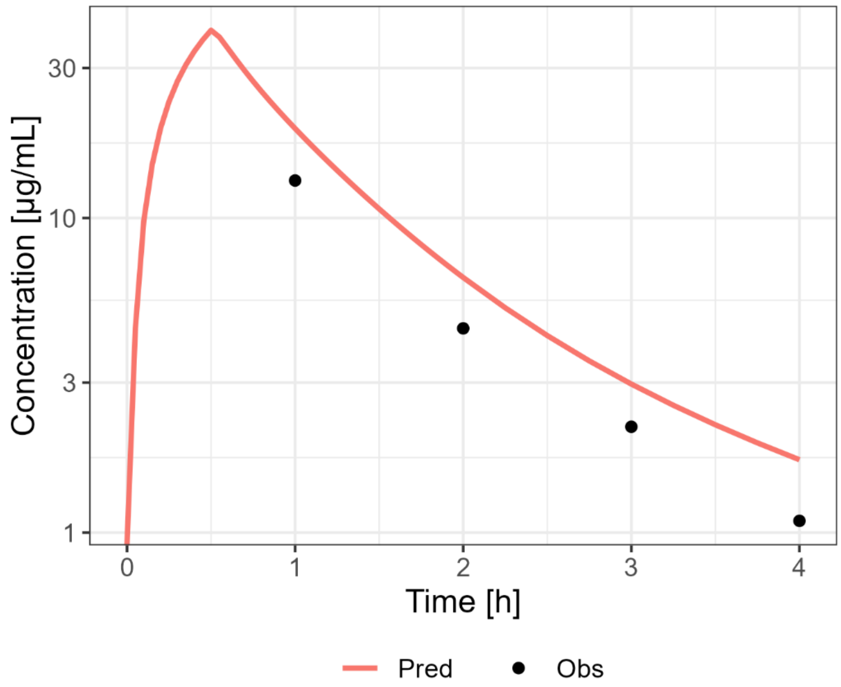


*Figure S7 Pedicted (Pred) versus observed (Obs) concentration-time profile after administration of 1000 mg IV* [10]

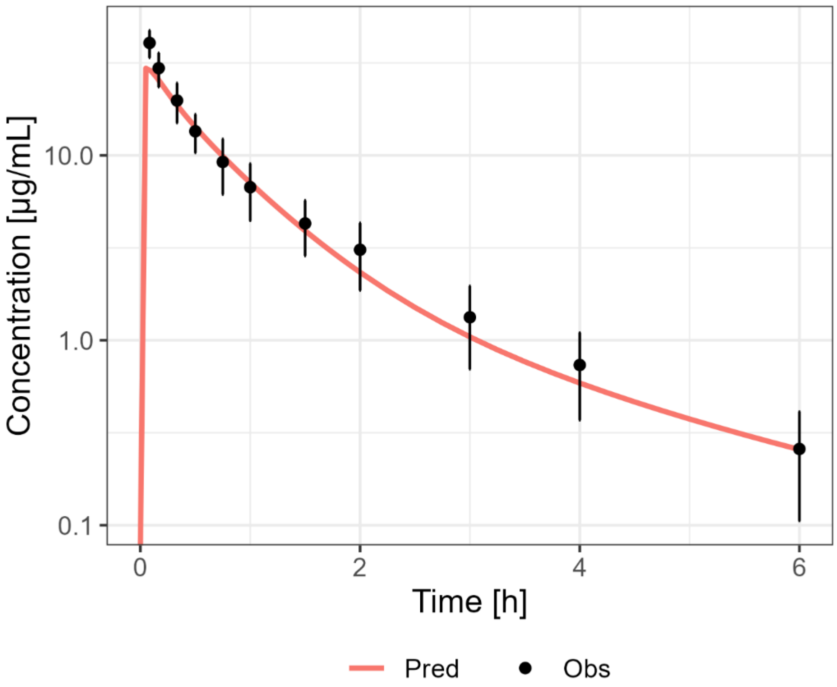


*Figure S8 Pedicted (Pred) versus observed (Obs) concentration-time profile after administration of 500 mg IV* [11]

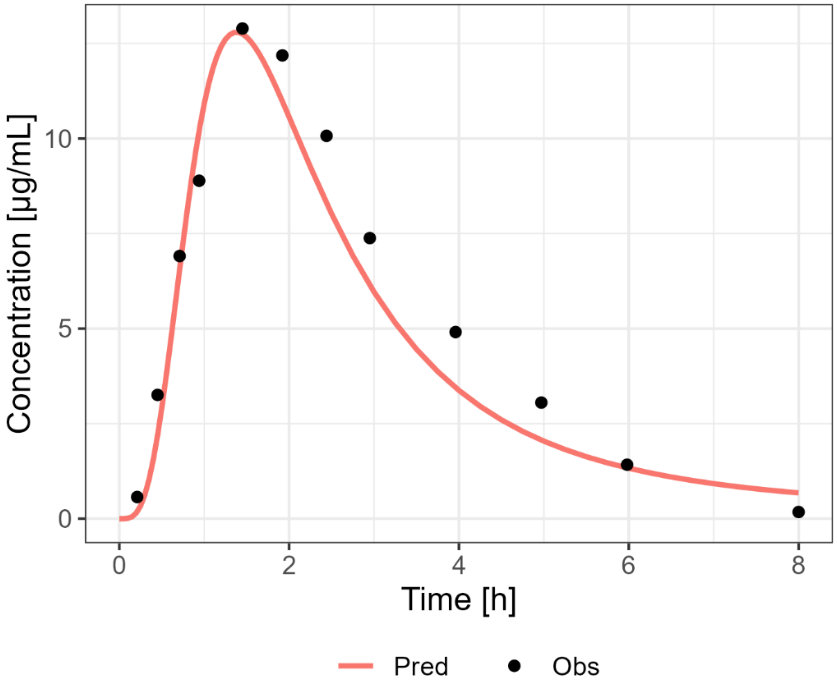


*Figure S9 Pedicted (Pred) versus observed (Obs) concentration-time profile after administration of 2x500 mg PO* [13]

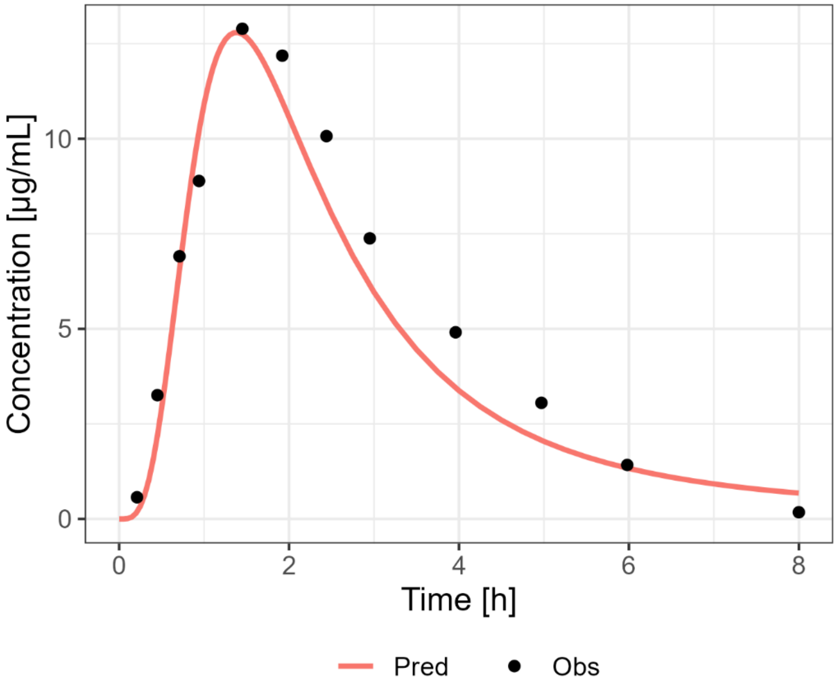


*Figure S10 Pedicted (Pred) versus observed (Obs) concentration-time profile after administration of 1000 mg PO* [13]

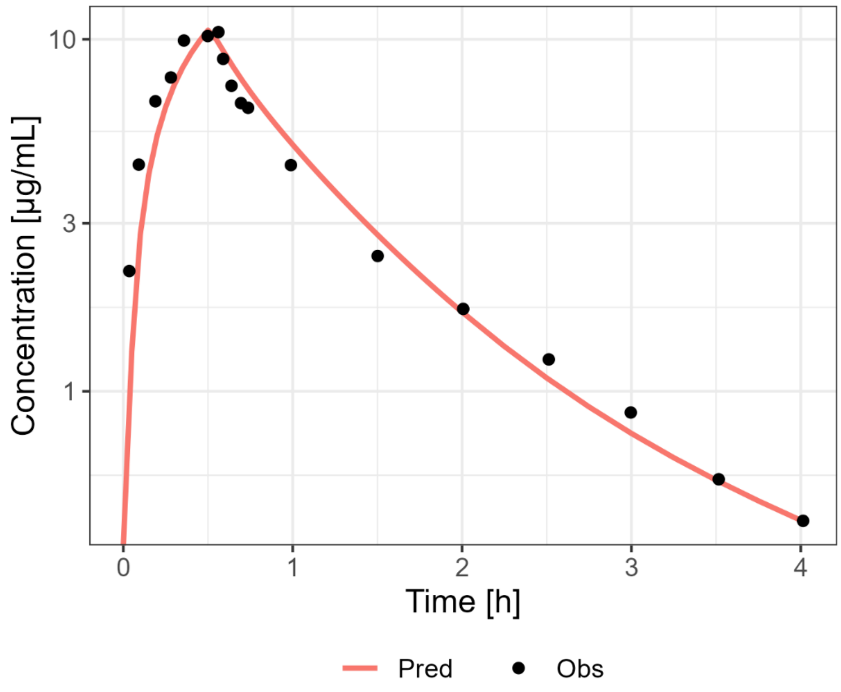


*Figure S11 Predicted (Pred) versus observed (Obs) concentration-time profile after administration of 250 mg IV* [12]

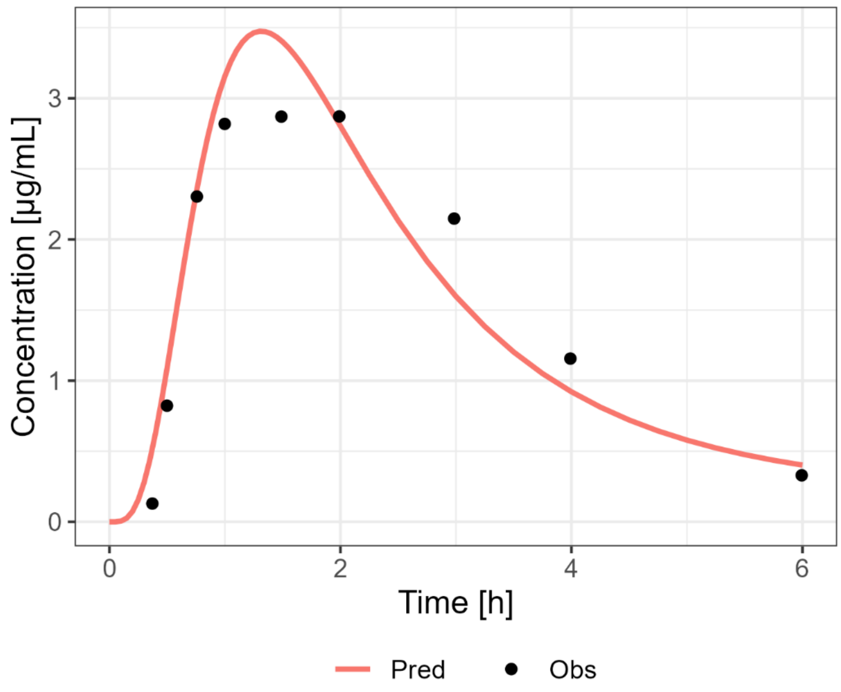


*Figure S12 Predicted (Pred) versus observed (Obs) concentration-time profile after administration of 250 mg PO* [12]

### 4.3.2 Model verification

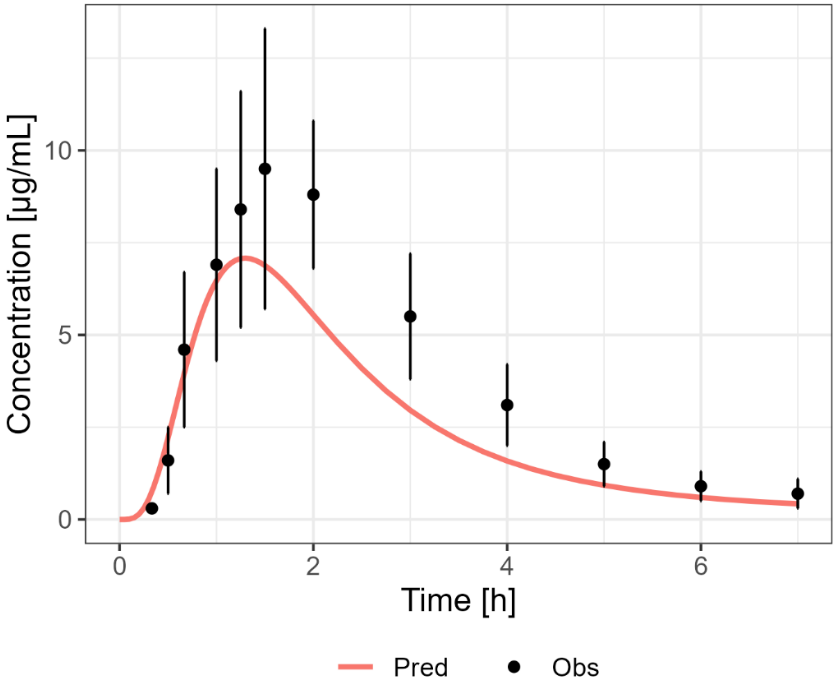


*Figure S13 Predicted (Pred) versus observed (Obs) concentration-time profile after administration of 500 mg PO* [7]

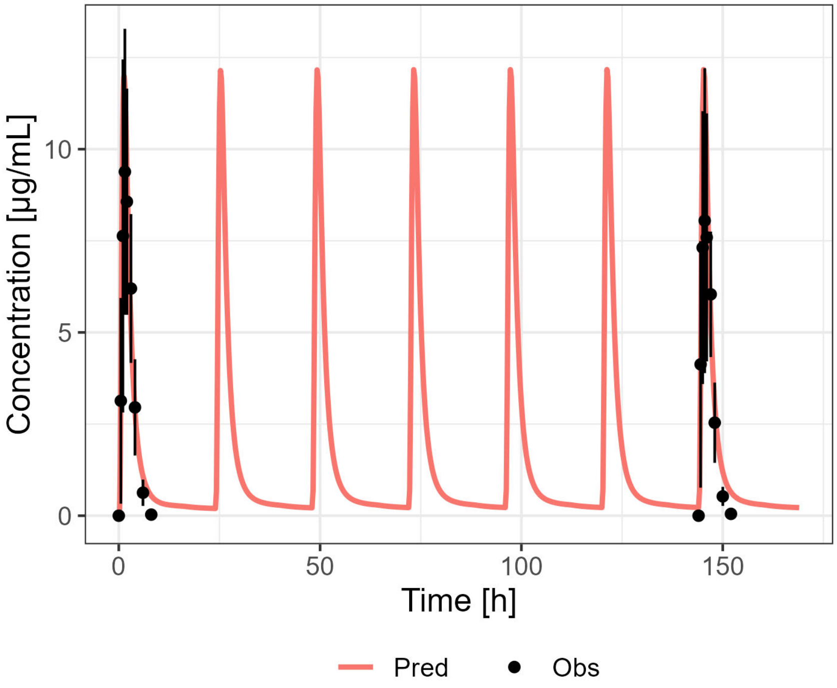


*Figure S14 Predicted (Pred) versus observed (Obs) concentration-time profile after administration of 1000 mg co-amoxiclav PO* [14]

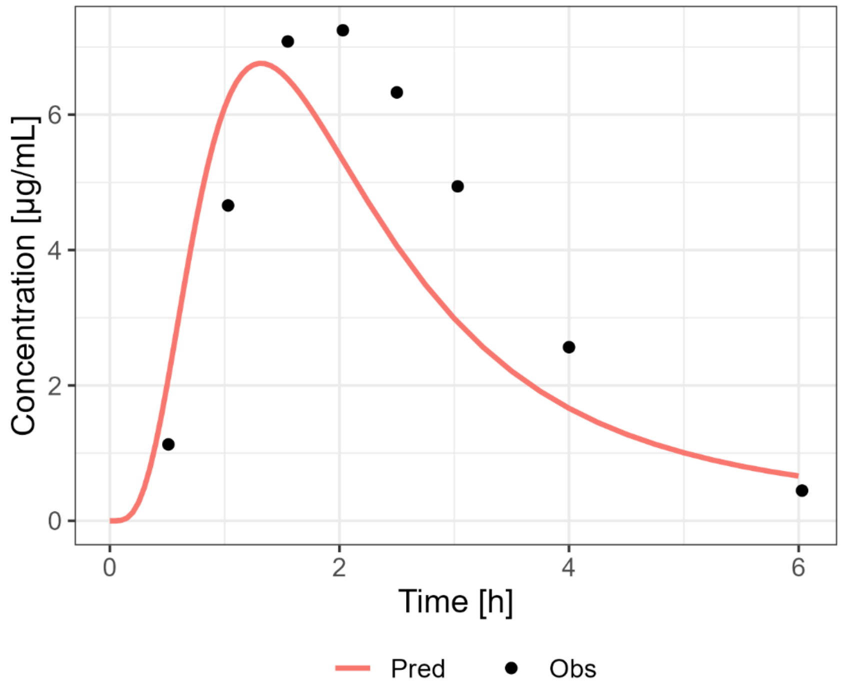


*Figure S15 Predicted (Pred) versus observed (Obs) concentration-time profile after administration of 500 mg Std PO* [15]

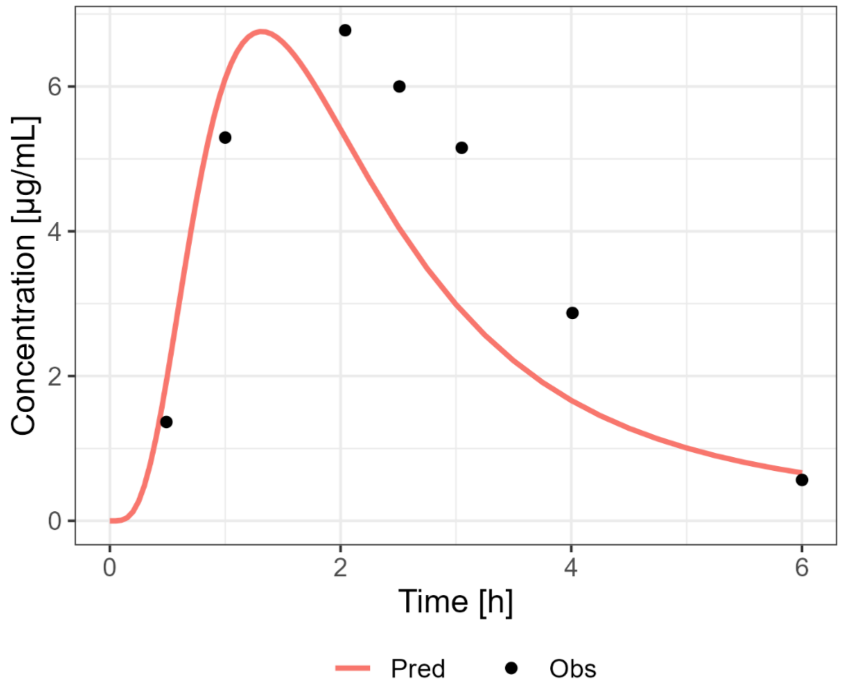


*Figure S16 Predicted (Pred) versus observed (Obs) concentration-time profile after administration of 500 mg Tst PO* [15]

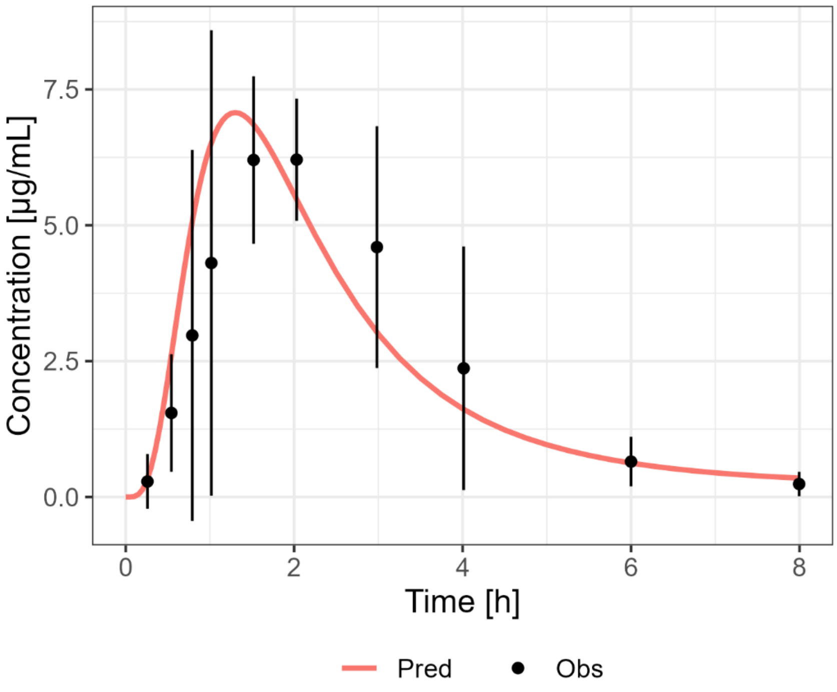


*Figure S17 Predicted (Pred) versus observed (Obs) concentration-time profile after administration of 500 mg augmentin PO* [11]

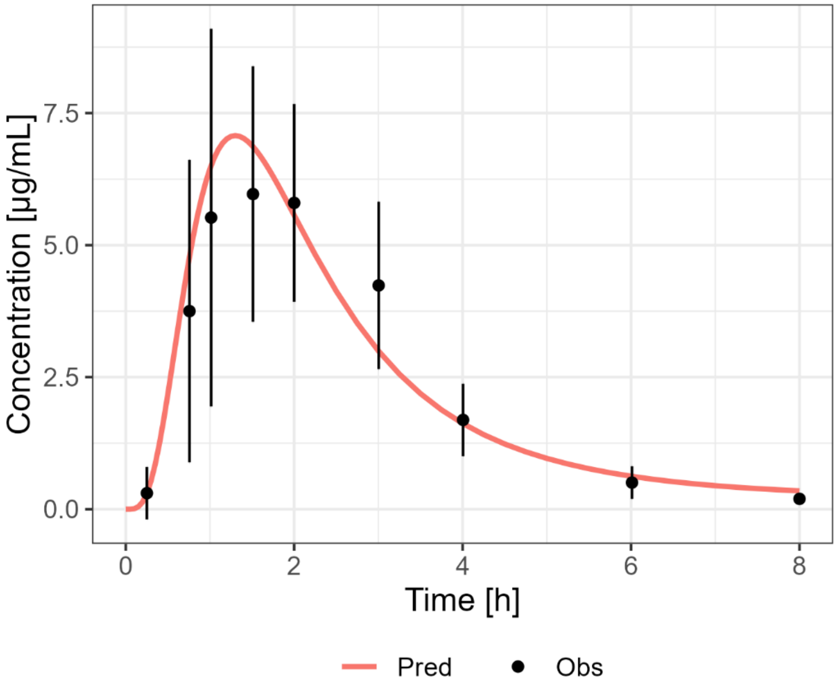


*Figure S18 Predicted (Pred) versus observed (Obs) concentration-time profile after administration of 500 mg PO* [11]

### 4.3.3 Lactation PBPK model

A sample size of 1000 individuals, three months postpartum, was used in each simulation of the virtual lactation population.

Model A: First, the transfer of amoxicillin was predicted using the LogP value of 0.87, and the default values for logD based on the equations implemented in the spatial structure building block as described above.

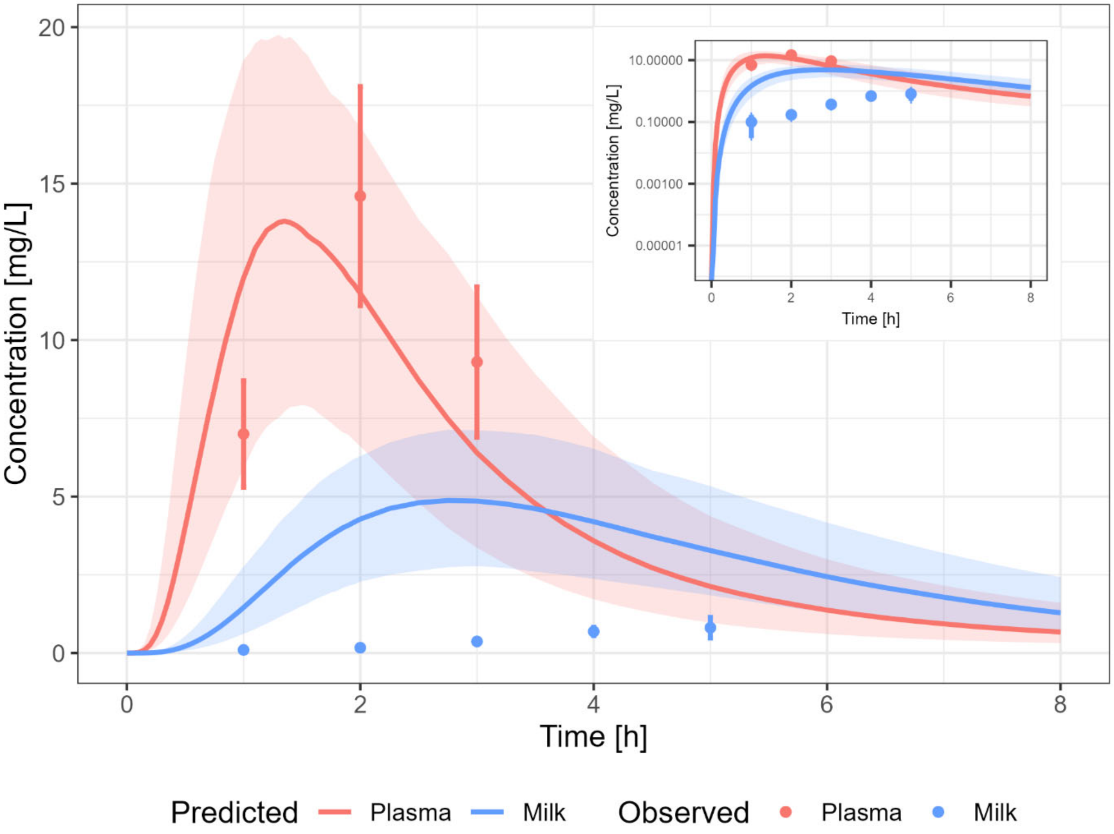


*Figure S19 Predicted (Pred) versus observed (Obs) concentration-time profile after administration of 1000 mg PO* [16]

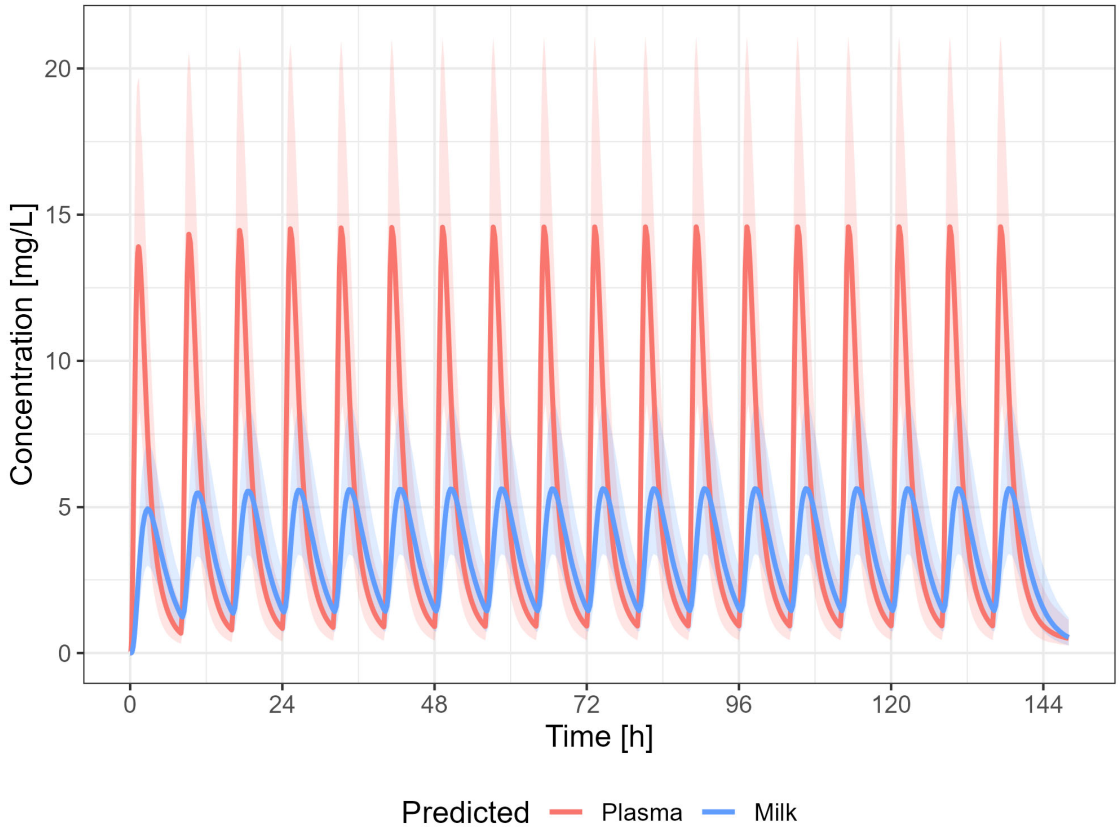


*Figure S20 Predicted (Pred) versus observed (Obs) concentration-time profile after administration of 1000 mg PO multiple dose*

A dosing regimen of PO 1000 mg three times a day was used to calculate the milk transfer of amoxicillin.

| Dosing interval: 8 h | Plasma | Milk |
|---|---|---|
| $C_{max}$ (mg/L) | 14.59 | 5.63 |
| AUC (mg*h/L) | 42.29 | 28.37 |
| Cave (mg/L) | 5.29 | 3.55 |

M/P ratio = 0.67

<u>Model B:</u> Alternatively, the logD values were overwritten with the values obtained from MarvinSketch (see section 4.2).

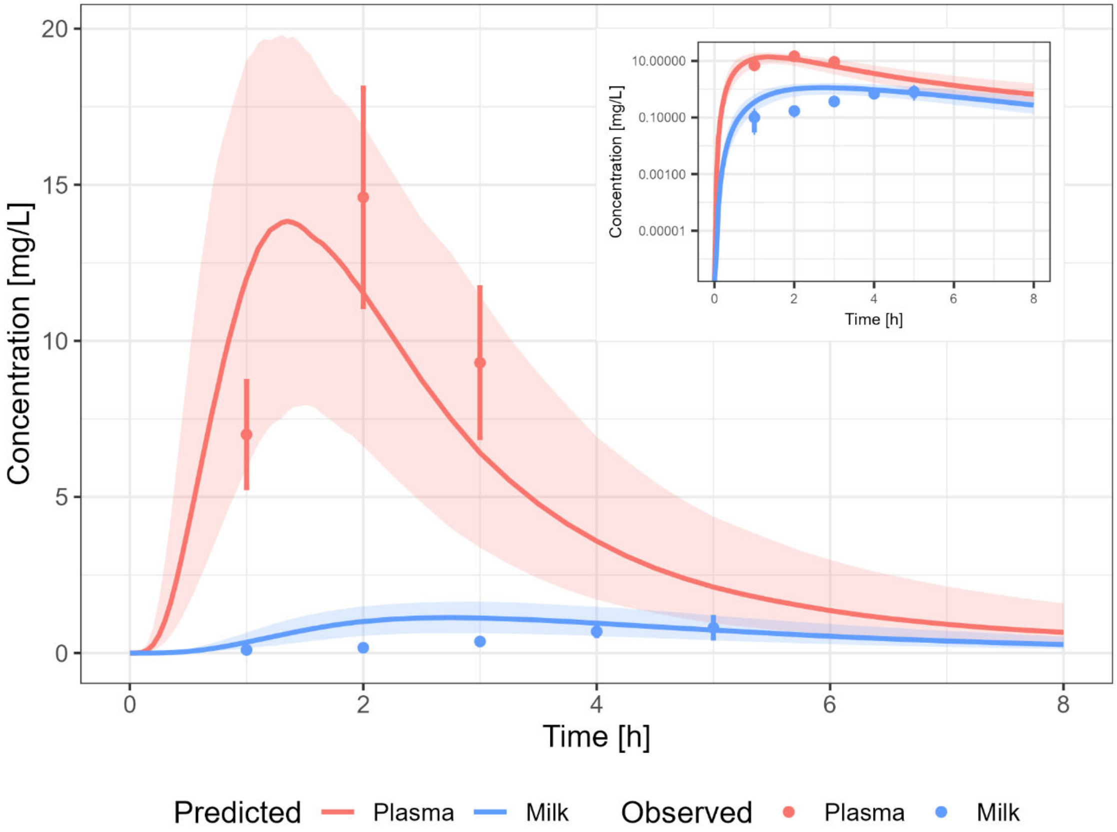


*Figure S21 Predicted (Pred) versus observed (Obs) concentration-time profile after administration of 1000 mg PO* [16]

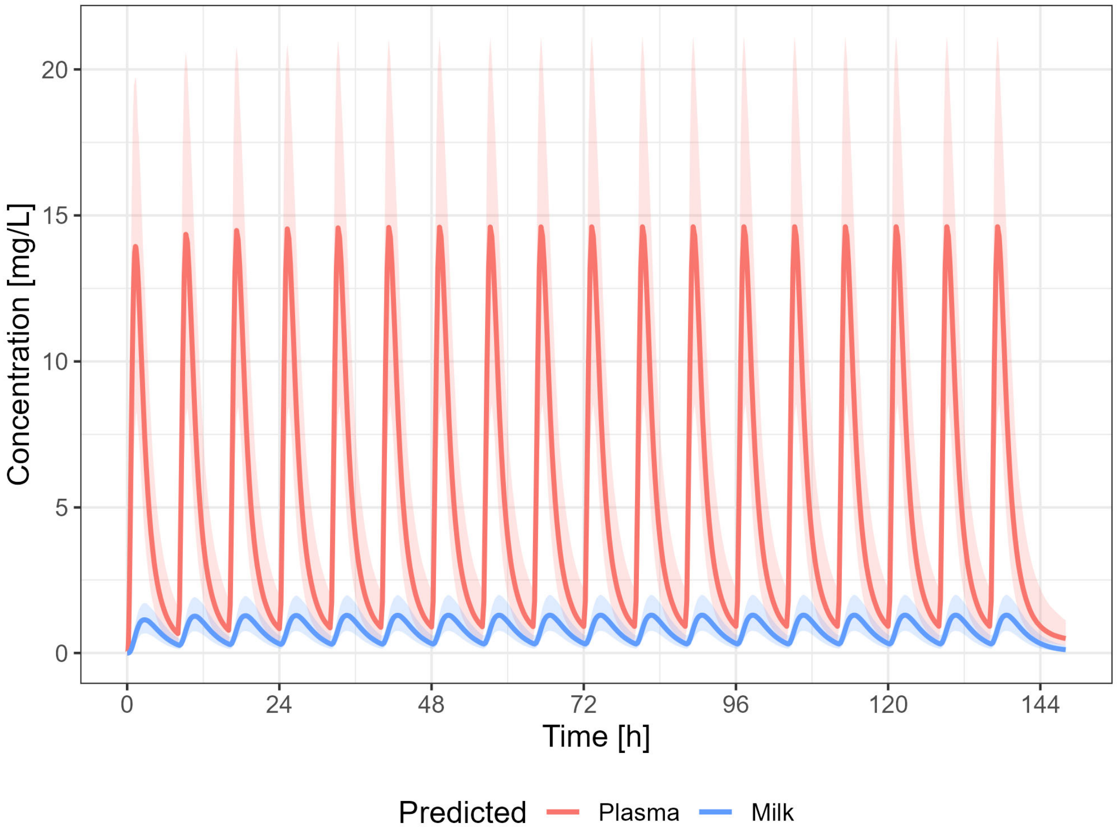


*Figure S22 Predicted (Pred) versus observed (Obs) concentration-time profile after administration of 1000 mg PO multiple dose*

A dosing regimen of PO 1000 mg three times a day was used to calculate the milk transfer of amoxicillin.

| Dosing interval: 8 h | Plasma | Milk |
|---|---|---|
| $C_{max}$ (mg/L) | 14.61 | 1.29 |
| AUC (mg*h/L) | 42.28 | 6.39 |
| Cave (mg/L) | 5.29 | 0.80 |

M/P ratio = 0.15

<u>Model C:</u> Finally, the LogP value as well as the LogD values were overwritten with the values obtained from MarvinSketch (see section 4.2).

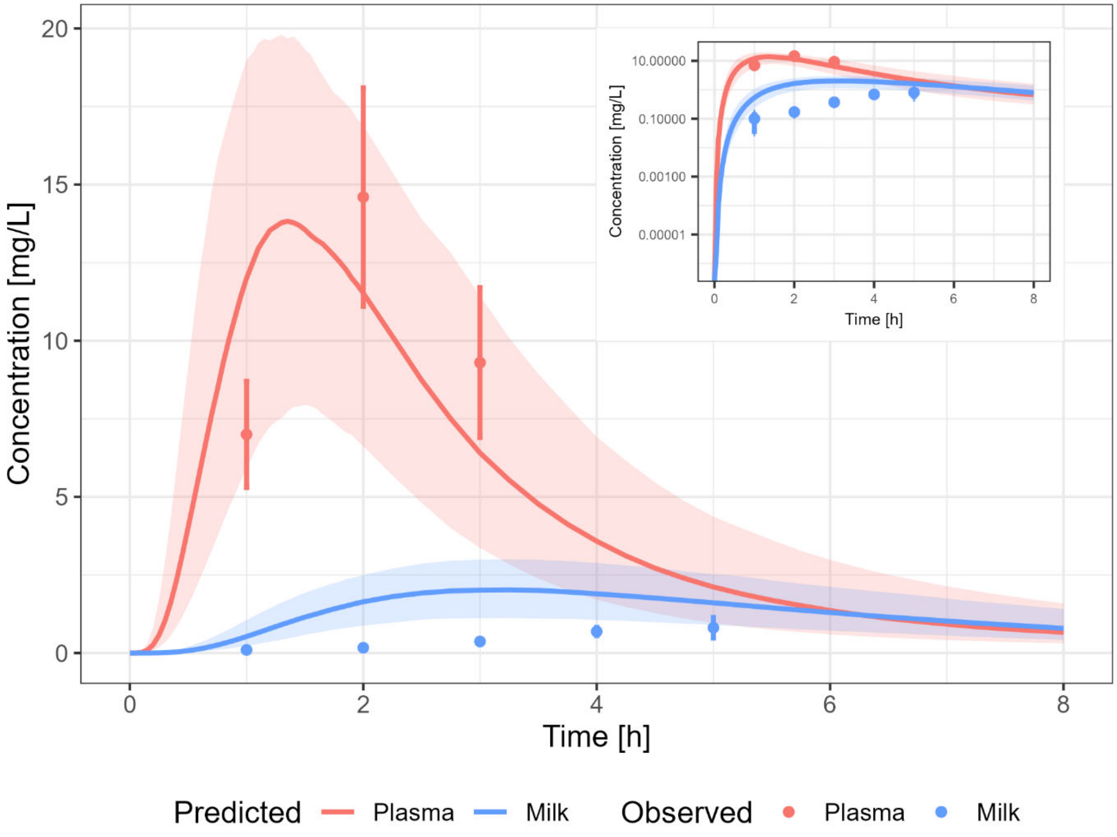


*Figure S23 Predicted (Pred) versus observed (Obs) concentration-time profile after administration of 1000 mg PO* [16]

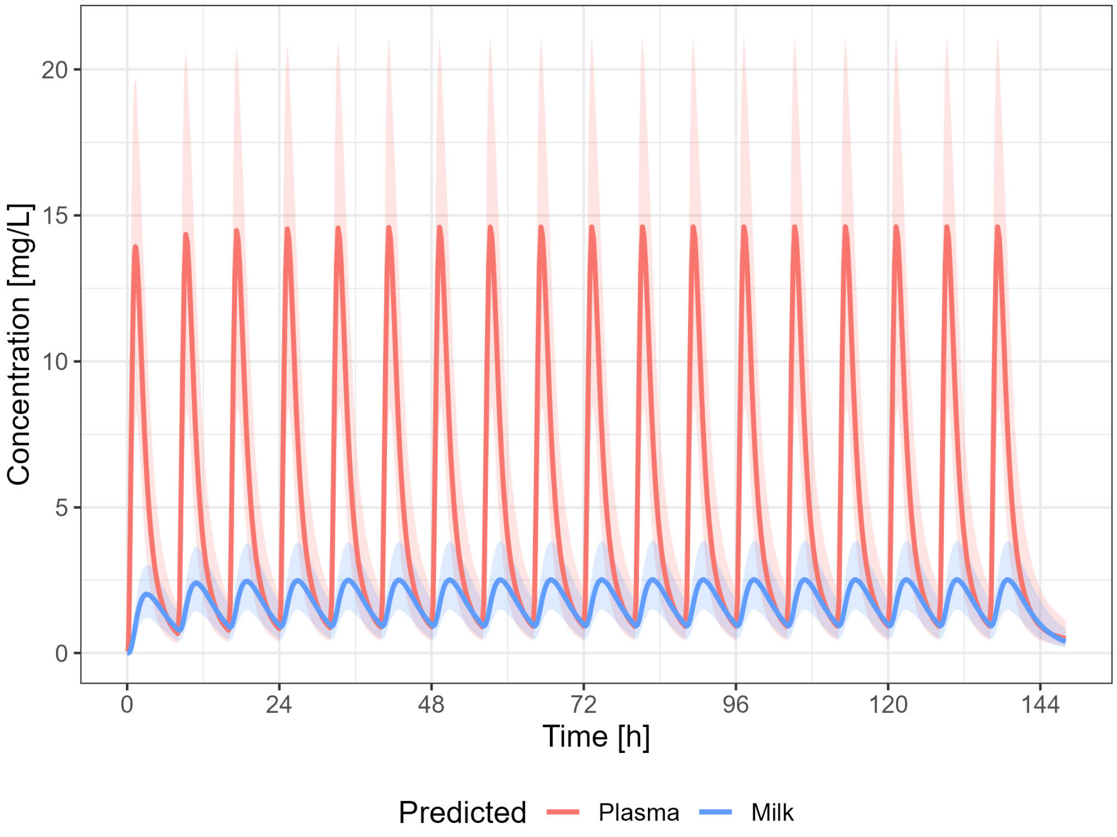


*Figure S24 Predicted (Pred) versus observed (Obs) concentration-time profile after administration of 1000 mg PO multiple dose*

A dosing regimen of PO 1000 mg three times a day was used to calculate the milk transfer of amoxicillin.

| Dosing interval: 8 h | Plasma | Milk |
|---|---|---|
| $C_{max}$ (mg/L) | 14.61 | 2.51 |
| AUC (mg*h/L) | 42.28 | 14.1 |
| Cave (mg/L) | 5.29 | 1.76 |

M/P ratio = 0.33

Model B was selected as final PBPK model for lactation for amoxicillin. The M/P ratio was 0.15.

## 4.4 Estimated infant dosage

A maternal dosing regimen of 1000 mg, every 8h was used to calculate the infant dosage. The daily infant dosage and relative infant dose (RID) for 3 months old infants were calculated using a milk intake of 150 mL/kg/day. The daily infant dosage was 0.12 mg/kg/day (RID: 0.24 %) or 0.19 mg/kg/day (RID: 0.39 %) based on the average steady-state concentration and maximum concentration in human milk, respectively.

## 5. Discussion

First, the reference PBPK model was developed and evaluated. Evaluation of the predictive performance showed that the reference PBPK model for amoxicillin was able to capture the pharmacokinetic behavior of the medicines in healthy volunteers and/or patients.

Next, the PBPK model was extended to a lactation PBPK model. The PBPK model results in an overprediction of the human milk concentrations. Importantly, the data was obtained in women at 3 days postpartum. Therefore, it cannot be excluded that the barrier at the blood-milk levels was not completely mature.

Alternatively, the LogD values used in the calculation of the milk parameters were overwritten with the LogD values obtained from MarvinSketch, which was also used to calculate LogD parameters by Koshimichi *et al. 2011.* The PBPK model results in a reasonable prediction of the human milk concentrations, with most datapoints within the 5-95$^{th}$ percentile of the population prediction.

The predicted milk-to-plasma ratio is 0.15. This is 3- to 10-fold higher than the observed M/P ratios (0.014 – 0.043). Importantly, the observed M/P ratio was taken from a single study, where three single time point M/P ratios were reported around the time of Cmax. An AUC-based M/P ratio was not available in literature. As the milk peak concentration is typically delayed with respect to the plasma peak concentration, this might explain why the predicted AUC-based M/P ratio is lower than the reported M/P ratios. Indeed, if we use the highest measured concentration in plasma (14.60 µg/mL) and human milk (0.81 µg/mL) to calculate the M/P ratio (0.06), the M/P ratio is only 2.5-fold overpredicted. Using non-compartmental analysis (NCA), and assuming that the elimination slope in human milk is identical to the slope observed in plasma, an AUC-based M/P ratio of 0.04 was calculated (4-fold prediction error).

The calculated infant dosage of amoxicillin via breastfeeding was very low, especially when compared to the maternal daily dosage.

## 6. Conclusions

The herein presented PBPK model adequately describes the PK of amoxicillin in adults including breastfeeding women. It applies elimination by hepatic clearance, glomerular filtration and tubular secretion. The PBPK model was able to reasonably predict the human milk concentrations of amoxicillin (M/P ratio: 0.15). The daily infant dosage was 0.12 mg/kg/day (RID: 0.24 %) or 0.19 mg/kg/day (RID: 0.39 %) based on the average steady-state concentration and maximum concentration in human milk, respectively.

## 7. List of Appendix and Supplementary Materials

Supplementary material 1 – ObsDataPK_OSP_reference_amoxicillin
Supplementary material 2 – ObsDataPK_OSP_lactation_amoxicillin
Supplementary material 3 – Amoxicillin.pksim5

## 8. References


1. Dallmann, A.; Himstedt, A.; Solodenko, J.; Ince, I.; Hempel, G.; Eissing, T. Integration of Physiological Changes during the Postpartum Period into a PBPK Framework and Prediction of Amoxicillin Disposition before and Shortly after Delivery. *J. Pharmacokinet. Pharmacodyn.* **2020**, *47*, 341–359, doi:10.1007/s10928-020-09706-z.
2. Dallmann, A.; Solodenko, J.; Ince, I.; Eissing, T. Applied Concepts in PBPK Modeling: How to Extend an Open Systems Pharmacology Model to the Special Population of Pregnant Women. *CPT pharmacometrics Syst. Pharmacol.* **2018**, *7*, 419–431, doi:10.1002/PSP4.12300.
3. Job, K.M.; Dallmann, A.; Parry, S.; Saade, G.; Haas, D.M.; Hughes, B.; Berens, P.; Chen, J.; Fu, C.; Humphrey, K.; et al. Development of a Generic Physiologically-Based Pharmacokinetic Model for Lactation and Prediction of Maternal and Infant Exposure to Ondansetron via Breast Milk. *Clin. Pharmacol. Ther.* **2022**, *111*, 1111–1120, doi:10.1002/cpt.2530.
4. Koshimichi, H.; Ito, K.; Hisaka, A.; Honma, M.; Suzuki, H. Analysis and Prediction of Drug Transfer into Human Milk Taking into Consideration Secretion and Reuptake Clearances across the Mammary Epithelia. *Drug Metab. Dispos.* **2011**, *39*, 2370–2380, doi:10.1124/dmd.111.040972.
5. Chemical Book.
6. Atkinson, U.C.; Begg, E.J.E.J.; Atkinson, H.C.; Begg, E.J.E.J. Prediction of Drug Distribution into Human Milk from Physicochemical Characteristics. *Clin. Pharmacokinet.* **1990**, *18*, 151–167, doi:10.2165/00003088-199018020-00005.
7. Arancibia, A.; Guttmann, J.; González, G.; González, C. Absorption and Disposition Kinetics of Amoxicillin in Normal Human Subjects. *Antimicrob. Agents Chemother.* **1980**, *17*, 199–202, doi:10.1128/AAC.17.2.199.
8. Arancibia, A.; Drouguett, M.T.; Fuentes, G.; González, G.; González, C.; Thambo, S.; Palombo, G. Pharmacokinetics of Amoxicillin in Subjects with Normal and Impaired Renal Function. *Int. J. Clin. Pharmacol. Ther. Toxicol.* **1982**, *20*, 447–453.
9. Mastrandrea, V.; Ripa, S.; La Rosa, F.; Tarsi, R. Human Intravenous and Intramuscular Pharmacokinetics of Amoxicillin. *Int. J. Clin. Pharmacol. Res.* **1984**, *4*, 209–212.
10. Tan, J.S.; Salstrom, S.J.; File, T.M. Levels of Antibiotic in Human Blood and Interstitial Fluid after Oral Administration of Bacampicillin or Phenoxymethyl Penicillin and Intravenous Administration of Amoxicillin or Ampicillin. *Rev. Infect. Dis. 3*, 121–124, doi:10.1093/clinids/3.1.121.
11. Witkowski, G.; Lode, H.; Höffken, G.; Koeppe, P. Pharmacokinetic Studies of Amoxicillin, Potassium Clavulanate and Their Combination. *Eur. J. Clin. Microbiol.* **1982**, *1*, 233–237, doi:10.1007/BF02019714.
12. Zarowny, D.; Ogilvie, R.; Tamblyn, D.; MacLeod, C.; Ruedy, J. Pharmacokinetics of Amoxicillin. *Clin. Pharmacol. Ther.* **1974**, *16*, 1045–1051, doi:10.1002/cpt19741661045.

13. Zaid, A.N.; Cortesi, R.; Kort, J.; Sweileh, W. Interchangeability of Two 500 Mg Amoxicillin Capsules with One 1000 Mg Amoxicillin Tablet after a Single Oral Administration. *Indian J. Pharm. Sci.* **2010**, *72*, 414–420, doi:10.4103/0250-474X.73904.
14. Burkhardt, O.; Borner, K.; von der Höh, N.; Köppe, P.; Pletz, M.W.; Nord, C.E.; Lode, H. Single- and Multiple-Dose Pharmacokinetics of Linezolid and Co-Amoxiclav in Healthy Human Volunteers. *J. Antimicrob. Chemother.* **2002**, *50*, 707–712, doi:10.1093/jac/dkf163.
15. Pires de Abreu, L.R.; Ortiz, R.M.; de Castro, S.C.; Pedrazzoli, J. HPLC Determination of Amoxicillin Comparative Bioavailability in Healthy Volunteers after a Single Dose Administration. *J. Pharm. Pharm. Sci. 6*, 223–230.
16. Kafetzis, A.; Siafas, C.A.; Georgakopoulos, P.A.; Papadatos, C.J. Cephalosporins Represent a Group of Antibiot- Ics of Increasing Interest . This Is Due to Their High Antibacterial Activity , Low Toxicity , and Easy Modification of the Molecule Resulting in the Production of New Compounds with Desir- Able Properties . T. *Acta Pa'diatr Scand* **1981**, *70*, 285–288.
