# Supplementary material for: Generic Workflow to Predict Medicine Concentrations in Human Milk Using Physiologically-Based Pharmacokinetic (PBPK) Modelling—A Contribution from the ConcePTION Project
Source: Pharmaceutics. 2023 May 11;15(5):1469. doi: 10.3390/pharmaceutics15051469 (PMC10223637; doi:10.3390/pharmaceutics15051469)
Supplement: Supplementary file 1 [file pharmaceutics-15-01469-s001.zip › File S10. Evaluation_report_zidovudine.pdf]

Building and evaluation of a Physiologically-Based PharmacoKinetic (PBPK) model for **zidovudine** in adults and lactating women

Disclaimer: The research project leading to these results was conducted as part of the ConcePTION consortium. This report only reflects the personal views of the stated authors. The results of this report are only intended for research purpose, and are not intended to be used in clinical practice.

Glossary

AMT	3'-amino-3'- deoxythymidine
AUC	Area Under the Curve
Bidaily	Twice a day
C_{ave}	Average concentration
CL_{re}	Reuptake clearance (i.e. from milk to blood)
CL_{sec}	Secretion clearance (i.e. from blood to milk)
C_{max}	Maximum (~peak) concentration
DID	Daily Infant Dosage (expressed for instance in mg/kg/day)
f_u	Fraction unbound in plasma
GFR	Glomerular Filtration Rate
GZDV	3′-azido-3′-deoxy-5′- O-beta-D-glucopyranuronosylthymidine
HBD	Hydrogen Bond Donors
HIV	Human immunodeficiency virus
IV	Intravenous (administration)
$LogD_{7.2}$	Logarithm of the partition coefficient between an octanol phase and an aqueous (buffer) phase at pH 7.2
$LogD_{7.4}$	Logarithm of the partition coefficient between an octanol phase and an aqueous (buffer) phase at pH 7.4
LogP	Logarithm of the partition coefficient between an octanol phase and (unbuffered) water as aqueous phase. This is the default parameter to express lipophilicity of a substance.
MD	Multiple dose
M/P ratio	Milk-to-Plasma ratio
MW	Molecular Weight (Da)
NADPH	Nicotinamide adenine dinucleotide phosphate
NRTI	Nucleoside reverse transcriptase inhibitor
PBPK	Physiologically-Based PharmacoKinetic [*modeling*]
pKa	Logarithm of the acid dissociation constant
PO	Oral administration
PSA	Polar surface area
q8hr	Every 8 hours
Q12hr	Every 12 hours
RID	Relative Infant Dose (%)
RT-PCR	Reverse transcription polymerase chain reaction
SD	Single dose
UGT	Glucuronosyl transferase

1. Table of Contents

1. Table of Contents ... 3

2. Introduction ... 4

3. Methods ... 5

3.1 Modelling strategy ... 5

3.1.1. Reference PBPK models ... 6

3.1.2. Lactation model ... 6

3.2 Data ... 6

3.2.1 *In vitro* / physicochemical data ... 6

3.2.2 Clinical data ... 10

3.3 Model Parameters and assumptions ... 12

3.3.1 Absorption ... 12

3.3.2 Distribution ... 12

3.3.3 Metabolism and excretion ... 12

3.3.4 Secretion to milk ... 12

3.3.5 Automated parameter optimization ... 13

3.4. Infant dosage calculation ... 13

4. Results ... 13

4.1 Final input parameters ... 14

4.2 Diagnostic plots ... 15

4.2 Concentration-time profiles ... 17

4.2.1 Model building ... 17

4.2.2 Model verification ... 18

4.2.3 Lactation PBPK model ... 19

4.3 Estimated infant dosage ... 20

5. Discussion ... 21

6. Conclusion ... 21

7. List of Appendix and Supplementary Materials ... 22

8. References ... 22

2. Introduction

Zidovudine (figure S1) is a nucleoside reverse transcriptase inhibitor (NRTI) with activity against human immunodeficiency virus type 1 (HIV-1) and is commonly used in combination with other anti-HIV drugs [1]. Recommended doses are 300 mg PO q12hr or 200 mg PO q8hr (600 mg/day) (Drug label Retrovir). Rapid and nearly complete absorption from the gastrointestinal tract following oral administration; however, because of first-pass metabolism, systemic bioavailability of zidovudine capsules and solution is approximately 65 % (range, 52 to 75 %) [2]. Bioavailability is increased in neonates up to 14 days of age, and it decreases in neonates over 14 days of age and children 3 months to 12 years, likely due to increased first-pass metabolism [3]. The volume of distribution of zidovudine in HIV-infected patients is 1.6 ± 0.6 L/kg (Drug label Retrovir). Zidovudine is metabolized by glucuronide conjugation to major, inactive metabolite, 3′-azido-3′-deoxy-5′- O-beta-D-glucopyranuronosylthymidine (GZDV) [2]. UGT2B7 is the primary UGT isoform that is responsible for glucuronidation [4]. The cytochrome P450 isozymes are responsible for the reduction of the azido moiety to form 3'-amino-3'- deoxythymidine (AMT) [5]. As in adult patients, the major route of elimination was by metabolism to GZDV. After intravenous (IV) dosing, about 2 9% of the dose was excreted in the urine unchanged and about 45% of the dose was excreted as GZDV. Elimination [6] half-life, in HIV-infected patients following IV administration is approximately 1.1 hours (range of 0.5 - 2.9 hours) [2].

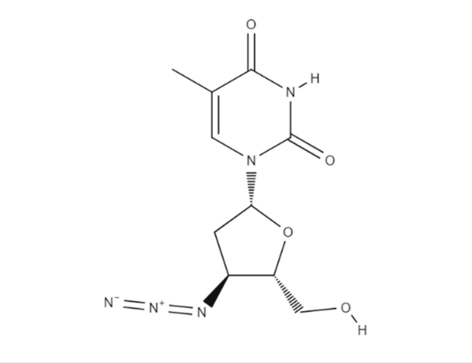

Figure S1. Chemical Structure of zidovudine

The scope of this report is to:

(a) specify the details and underlying assumptions associated with the building of physiologically-based pharmacokinetic (PBPK) models for zidovudine in adult healthy volunteers or patients, and in postpartum women during lactation.

(b) evaluate the predictive performance of these PBPK models. This is achieved by comparing model-predicted plasma or milk concentrations with corresponding clinical observations.

3. Methods

The software used for the development of PBPK models presented in this report is tabulated below:

Software	Version
PK-Sim®	v9.1
MoBi®	v9.1

3.1 Modelling strategy

In the present report, a reference PBPK model was first established for adults (patients as well as healthy volunteers), and subsequently verified against clinical pharmacokinetic data reported for zidovudine in the scientific literature.

Relevant information on the anthropometry (height, weight) was gathered from the respective clinical studies, if reported. Information on physiological parameters (e.g. blood flows, organ volumes, hematocrit) in adults is available in the PK-Sim® database.

In a second step, a lactation PBPK model was developed, based on the general workflow described by Dallmann *et al. 2018* [7–9].

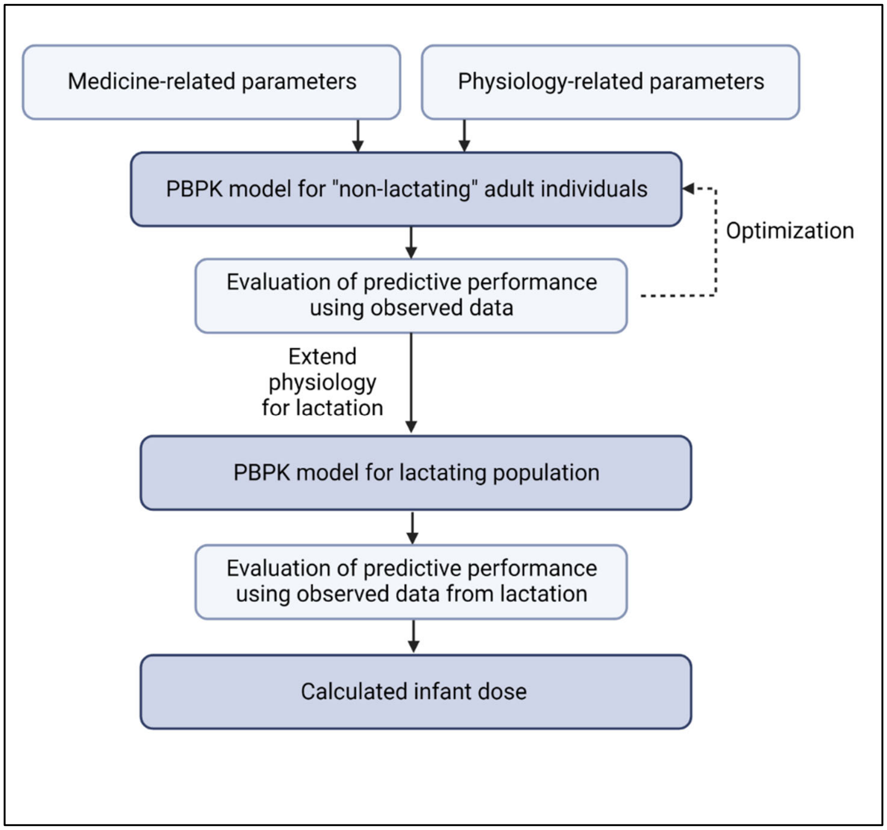

Figure S2 General workflow that was used in the present project to develop and evaluate the lactation PBPK model

Details about input data (physicochemical, *in vitro* and clinical data) can be found in section 3.2. Details about the structural models and their parameters can be found in section 3.3.

3.1.1. Reference PBPK models

The reference PBPK models were built based on studies with adult volunteers and/or adult patients, using the reported mean values for age, weight, height, and genetic background as described in each study protocol. When no information on these parameters could be found, a healthy male European individual, 30 years of age, with a body weight of 73 kg and a height of 176 cm was used.

The abundance (including population variability) of plasma proteins and enzymes/transporters that are integrated into PK-Sim are described in the publicly available 'PK-Sim Ontogeny Database Version 7.3' (PK-Sim Ontogeny Database Version 7.3).

Zidovudine is metabolized via glucuronidation (mainly UGT2B7) and NADPH dependent reduction (involvement of different cytochrome P450 enzymes and b5 reductase suggested in literature). To model the specific metabolic clearance, UGT2B7 and CYB5R4 were implemented in accordance with literature, using the PK-Sim expression database. RT-PCR profiles to define their relative expression in the different organs of the body [4,5]. Both UGT2B7 and CYB5R4 were implemented as *in vitro* metabolic rate in the presence of liver microsomes [10,11]. Glomerular filtration and active first-order tubular secretion were enabled, as they are involved in zidovudine excretion.

Structural model selection was mainly guided by biological plausibility and by visual inspection of the predicted concentration time profiles in comparison with observed data. The generally applied acceptance criterium was less than 2-fold misprediction. Uninformed parameter values (see below) were estimated using the parameter identification module of PK-Sim®.

The predictive performance of the models was evaluated by simulating:
- Single intravenous dose studies
- Single and multiple oral dose studies

For some parameters, parameter optimization was performed as described below to obtain improved concordance between predicted profiles and observed data.

3.1.2. Lactation model

After development of the reference model, the model was exported to MoBi® and a lactation PBPK model was constructed. To model the passage of zidovudine into human milk, i.e. across the blood/milk biological barrier, both the secretion (CL_{sec}) and reuptake clearance (CL_{re}) values were obtained using the empirical model developed by Koshimichi *et al. 2011* [12].

3.2 Data

3.2.1 *In vitro* / physicochemical data

A literature search was performed to collect available information on physicochemical properties of zidovudine. The obtained information from literature is summarized in Table S1.

Table S2 shows the parameters that were additionally used for the lactation PBPK model.

Table S1 Physicochemical parameters used as input for the zidovudine PBPK models

**Parameter**	**Value**	**Unit**	**Description**	**Source**
MW	267.24	g/mol	Molecular weight	Drugbank
pK_a	9.70 (acid)	-	Logarithm of the acid dissociation constant	[13]
Solubility (pH 7)	20.10	mg/mL	Aqueous solubility	Drugbank
LogP	0.05	-	Log_{10} of the partition coefficient between octanol and water (~lipophilicity)	Drugbank
f_u	0.8	-	Fraction unbound in human plasma	[13]
**AMT formation** - In vitro metabolic rate in the presence of liver microsomes – First order	1.26	µL/min/mic. protein	Metabolic enzyme activity	[10]
**GZDV formation** - In vitro metabolic rate in the presence of liver microsomes – Michaelis Menten:<br>Km<br>Vmax	 150<br>4700	 µM<br>pmol/min/mg mic. protein	Metabolic enzyme activity	[11]
**Tubular secretion** – First order – Tubular secretion	280	m1/min	Active tubular secretion	[14]
GFR fraction	1	-	Glomerular filtration	

Table S2 Physicochemical parameters used as input for the lactation PBPK model of zidovudine

Parameter	Value	Unit	Description	Source
Milk logP[a]	0.05	-	Log_{10} of the partition coefficient between octanol and water	Drugbank
HBD	2.00	-	Hydrogen bond donors	Pubchem
PSA	108.30	Å^2	Polar surface area	Pubchem

[a] Milk logP is Log_{10} of the partition coefficient between octanol and water and is used as input for the calculations in the postpartum model (see equations below). In theory, this value is identical to the logP specified in Table S1. However, in some PBPK models, logP (Table S1) might be optimized using parameter identication. Therefore, it was chosen to use a separate parameter (i.e. Milk logP) to represent the logP used as input for the equations in the postpartum model.

The default equations for free fraction in human milk and logD that were implemented in the spatial structure building block that was developed for the postpartum women are described below. Alternatively, these values can be overwritten by values calculated elsewhere (e.g. MarvinSketch) or determined *in vitro.*

The free fraction in human milk was calculated with the equations proposed by Atkinson and Begg [15], as follows:

$$f_{u\_skimmed\ milk} = \frac{f_u \times 0.448}{(0.000694^{0.448} + \mathrm{f}_{\mathrm{u}}^{0.448})}$$

$$P_{milk} = 10^{(-0.88+1.29\times \mathrm{logD7.2})}$$

$$\text{Total free fraction in milk} = \frac{1}{\left(\frac{0.955}{f_{u\_skimmed\ milk}} + 0.045 \times P_{milk}\right)}$$

Where: *f_u skimmed milk:* binding to proteins in milk; P_{milk}: partitioning between aqueous and lipid phase of milk; Total free fraction in milk: 'total' free fraction, i.e. accounting for both protein and lipid binding processes.

LogD values taking into account up to three pka values (as provided in the compound building block), were calculated as follows:

$$LogD = \mathrm{Log}\,P + Log_{10}(logD_{factor})$$

With Milk logP (Table S2) as input for logP

$$LogD_{factor} = \mathrm{K}_1 + (\mathrm{K}_2 + \mathrm{K}_3 + \mathrm{K}_4) \times \mathrm{base}^1 + \mathrm{K}_5 \times \mathrm{base}^{\max(\mathrm{CT}_0+\mathrm{CT}_1;-\mathrm{CT}_0-\mathrm{CT}_1)} + \mathrm{K}_6 \times \mathrm{base}^{\max(CT_0+CT_2;-CT_0-CT_2)} + \mathrm{K}_7 \times \mathrm{base}^{\max(\mathrm{CT}_2+\mathrm{CT}_1;-\mathrm{CT}_2-\mathrm{CT}_1)} + \mathrm{K}_8 \times \mathrm{base}^{\max(\mathrm{CT}_0+\mathrm{CT}_1+\mathrm{CT}_2;-\mathrm{CT}_0-\mathrm{CT}_1-\mathrm{CT}_2)}$$

$$K_1 = F_1 \times F_2 \times F_3$$
$$K_2 = (1 - F_1) \times F_2 \times F_3$$
$$K_3 = F_1 \times (1 - F_2) \times F_3$$
$$K_4 = F_1 \times F_2 \times (1 - F_3)$$
$$K_5 = (1 - F_1) \times (1 - F_2) \times F_3$$
$$K_6 = (1 - F_1) \times F_2 \times (1 - F_3)$$
$$K_7 = (1 - F_1) \times F_2 \times (1 - F_3)$$
$$K_8 = (1 - F_1) \times (1 - F_2) \times (1 - F_3)$$

F1 = CT0 != CT_NEUTRAL ? 1/(1+10^(CT0*(pKa_0- pH))) : 1
F2 = CT1 != CT_NEUTRAL ? 1/(1+10^(CT1*(pKa_1- pH))) : 1
F3 = CT2 != CT_NEUTRAL ? 1/(1+10^(CT2*(pKa_2- pH))) : 1

With CT = compound type (-1: acid; +1: base; 0: neutral), and pH = 7.2 or 7.4 respectively for $logD_{7.2}$ *and* $logD_{7.4}$

The transports that were added in the passive transport building block for 'transfer to milk' and 'transfer from milk' are based on secretion and reuptake and clearance values, Cl_{sec} and Cl_{re}, which were calculated according to the empirical equations proposed by Koshimichi et al. 2011 [12], as follows:

$$\mathrm{Log}\, CL_{re} = 2.793 + 0.179 \times LogP - 0.132 \times HBD$$

$$\mathrm{Log}\, CL_{sec} = 3.367 \times Log10(MW) - 0.164 \times (LogP - LogD) - 0.015 \times PSA - 3.912$$

3.2.2 Clinical data

Literature searches were performed to collect available data on zidovudine in adults and postpartum women. The zidovudine reference PBPK model was developed using a clinical studies with pharmacokinetic (PK) blood sampling after oral and intravenous administration [16]. The predictive performance of the reference PBPK model was verified using another clinical study with oral and intravenous administration, and a multiple dose study [17,18].

The evaluation of the predictive performance of the zidovudine lactation PBPK model was performed using 5 different studies were zidovudine was administered as an oral dose of 300 mg bidaily to lactating women [19–23]. The women were between 0 and 6 months postpartum. The samples were assumed to be trough samples when the exact timing was not reported in the articles.

Detailed information and data from the studies used for model building, verification, and lactation model can be found in Supplementary material 1 and 2.

3.2.2.1 Model building

The studies that were used for model building are shown in Table S3 (**training data**).

Table S3 Summary of studies used for PBPK model building of zidovudine in reference populations

Study ID	Reference	Arm/treatment/information used for model building
Moore 1995	[24]	8 patients received 120 mg IV (single dose)
Moore 1995	[24]	8 patients received 200 mg PO (single dose)

Table S4 Demographic information

Study ID	Reference	Number of subjects (female ratio)	Age (year)	Weight (kg)
Moore 1995	[24]	8 (0.13)	34.5 (-)	68.275 (-)

3.2.2.2 Model verification

The studies that were used to evaluate the predictive performance of the PBPK model are shown in Table S5 (**verification data**).

Table S5 Summary of studies used for model verification of zidovudine PBPK model in reference population

Study ID	Reference	Arm/treatment/information used for model verification
McNab 1993	[17]	16 patients received 200 mg IV (single dose)
McNab 1993	[17]	16 patients received 200 mg PO (single dose)
Laskin 1989	[18]	6 patients received 200 mg every 4 hours PO (multiple dose)

Table S6 Demographic information

Study ID	Reference	Number of subjects (female ratio)	Age (year)	Weight (kg)
McNab 1993	[17]	16 (0)	35.9 ± 6.1	67.5 ± 11.8
Laskin 1989	[18]	6 (0)	36.5 (30-54)	79.4 (73-95)

3.2.2.3 Lactation PBPK model

Table S7 shows the study that was used for the lactation PBPK model.

Table S7 Summary of study used for PBPK model development of zidovudine in lactating women

Study ID	Reference	Arm/treatment/information used for model verification
Corbett 2014	[19]	30 women (6/12/24 weeks postpartum) received PO 300 mg bidaily (multiple dose)
Giuliano 2007	[20]	80 women (0-3 days and 1 week postpartum) received PO 300 mg bidaily (multiple dose)
Mirochnick 2009	[21]	67 women (0/2/6/14/24 weeks postpartum) received PO 300 mg bidaily (multiple dose)
Palombi 2012	[22]	66 women (1/3/6 months postpartum) received PO 300 mg bidaily (multiple dose)
Shapiro 2005	[23]	20 women (2/5 months postpartum) received PO 300 mg bidaily (multiple dose)

3.3 Model Parameters and assumptions

3.3.1 Absorption

The specific intestinal permeability was based on the reported permeability coefficient [25]. Solubility was taken from drugbank. Oral administration results in rapid absorption, and was implemented as a solution.

3.3.2 Distribution

An important parameter influencing the distribution of a compound is lipophilicity. Since multiple transporters (ENT/CNT) are involved in the distribution of zidovudine, the lipophilicity was further optimized via parameter identification. The tissue partition coefficients (K_p) calculation was according to 'PK-Sim Standard' and the cellular permeability calculation was 'PK-Sim Standard'.

3.3.3 Metabolism and excretion

The final model applies metabolism via glucuronidation and reduction, and renal clearance. Glucuronidation was implemented via UGT2B7, since UGT2B7 is the main enzyme responsible for this pathway. The *in vitro* metabolic clearance in presence of liver microsomes was implemented as Michaelis Menten kinetics [11]. The content of protein in the experiment was estimated via parameter identification. Multiple enzymes are described that might play a role in the reduction. CYB5R4 was selected to represent this pathway in the PBPK model. The *in vitro* metabolic clearance in the presence of liver microsomes was implemented as first order clearance based on reported values [10]. Renal clearance was implemented as GFR and tubular secretion, based on reported values [14].

3.3.4 Secretion to milk

To model the transfer process of zidovudine into human milk, both the secretion (CL_{sec}) and reuptake clearance (CL_{re}) were calculated using the empirical equations developed by Koshimichi *et al.* 2011 (see **Error! Reference source not found.**) [4].

First, in MoBi®, a spatial structure for the postpartum women was constructed, similar to the workflow from Dallmann *et al.* 2018 [2]. Here, breasts were added as a compartment. In addition, the human milk was connected to the plasma subcompartment of the breasts. The human milk volume was specified as 0.5 L to represent the structure of Koshimichi *et al.* 2011, and a geometric standard deviation of 1.16 was assumed in the population. The free fraction in human milk, and logD values were implemented as the equations described previously. The transfer between plasma and milk was defined as two kinetic processes (transfer to milk and transfer from milk) under passive transports (see below). Next, the simulation was combined with the postpartum population from Job *et al.* 2021 in PK-Sim to account for the postpartum physiology [3].

<u>*Kinetics*</u>

Transfer to milk

$$\frac{dNmilk}{dt} = Cplasma \times f_u \times CL_{sec}$$

where Cplasma is the concentration in plasma (in breast compartment), f_u is the free fraction in plasma and CL_{sec} is the secretion clearance.

Transfer from milk

$$\frac{dNplasma}{dt} = Cmilk \times f_u \times CL_{re}$$

where Cmilk is the concentration in human milk, f_u is the total free fraction in human milk (protein and lipid) and CL_{re} is the reuptake clearance.

The median simulated plasma and human milk concentration-time profiles can be used to calculate the M/P ratio as follows:

$$M/P\ ratio\ = \frac{AUC_{milk}}{AUC_{plasma}}$$

3.3.5 Automated parameter optimization

The following table depicts the results of the final parameter optimization according to the different clinical studies.

a) Moore et al. (1995) 120 mg IV; Moore et al. (1995) 200 mg PO; Fraction excreted to urine (IV: 0.2; PO: 0.14); Fraction metabolized via glucuronidation (IV: 0.6; PO: 0.75) [24]

Model parameter	Optimized value	Unit
Lipophilicity	1.27	-
Content of proteins in liver microsomes	48.73	Pmol/mg mic. protein

3.4. Infant dosage calculation

Infant dosage via human milk was then calculated based on the predicted (average and maximal) steady-state zidovudine concentration in human milk, as well as the daily milk intake volume. The daily infant dosage was then compared to the maternal dosage, resulting in the relative infant dose (RID).

$$Daily\ infant\ dosage = C_{average} * 150\ \frac{mL}{kg\ .day}$$

$$Daily\ infant\ dosage = C_{max} * 150\ \frac{mL}{kg\ .day}$$

$$Relative\ infant\ dose\ (RID) = \frac{Infant\ dosage}{Maternal\ dosage} * 100\ \%$$

4. Results

Both the reference and postpartum PBPK model of zidovudine was developed and verified with clinical PK data.

The models were evaluated covering studies including in particular:

- Intravenous and oral administration
- Single and multiple doses

The model describes metabolism via glucuronidation (UGT2B7) and reduction (CYB5R4) as well as renal clearance via GFR and active tubular secretion. Moreover, secretion and reuptake to human milk were described by CL_{sec} and CL_{re}.

The next sections show:
- The final model parameters for the building blocks: section 4.1
- The overall predictive performance: section 4.2
- The simulated versus observed concentration-time profiles for the clinical studies used for model building and for model verification: section 4.3

4.1 Final input parameters

The compound values of the final postpartum PBPK model for zidovudine are illustrated below.

Physicochemical parameters

Parameter	Value	Unit	Source
MW	267.24	g/mol	Drugbank
pKa	9.70 (acid)	-	[13]
Solubility	20.10	mg/mL	Drugbank
Lipophilicity	1.27	Log units	Parameter identification
f_u	0.80	-	[13]
Small molecule (Y/N)	Yes	-	-
Plasma protein binding partner	Albumin	-	-

Calculation methods

Name	Value
Tissue partition coefficients	PK-Sim Standard
Cellular permeabilities	PK-Sim Standard

AMDE-related parameters

Parameter	Value	Unit	Source
Intestinal permeability	5.34E-4	cm/s	[25]
**AMT formation** - In vitro metabolic rate in the presence of liver microsomes – First order	1.26	µL/min/mic. protein	[10]
**GZDV formation** - In vitro metabolic rate in the presence of liver microsomes – Michaelis Menten: Km	150	µM	[11]

Vmax	4700	pmol/min/mg mic. protein	
GFR fraction	1.00		
**Tubular secretion** – first order	280	mL/min	[14]

Physicochemical and physiological parameters relevant to the lactation model

**Parameter**	**Value**	**Unit**	**Source**
Milk logP	0.05	-	Pubchem
HBD	2.00	-	Pubchem
PSA	108.30	Å^2	Pubchem
CL_{sec}	7.18E-3	L/min	Default
CL_{re}	5.75E-3	L/min	Default
*f*u_skimmed milk[a]	0.96	-	Default
Pmilk[b]	0.15	-	Default
Total free fraction in milk[c]	1.00	-	Default
$logD_{7.2}$	0.05	-	Default
$logD_{7.4}$	0.05	-	Default

[a] binding to proteins in milk; [b] partitioning between aqueous and lipid phase of milk; [c] total free fraction, accounting for both protein and lipid binding

4.2 Diagnostic plots

The geometric mean fold errors (GMFE) on AUC and Cmax were 1.28 and 1.25 for the model building dataset, and 1.04 and 1.39 for the model verification dataset.

The following plot shows the predictive performance graph for Cmax and AUC of zidovudine for the PBPK model performance of all data used.

Predicted over observed ratio values of all data listed in section 3.2.2 are presented below.

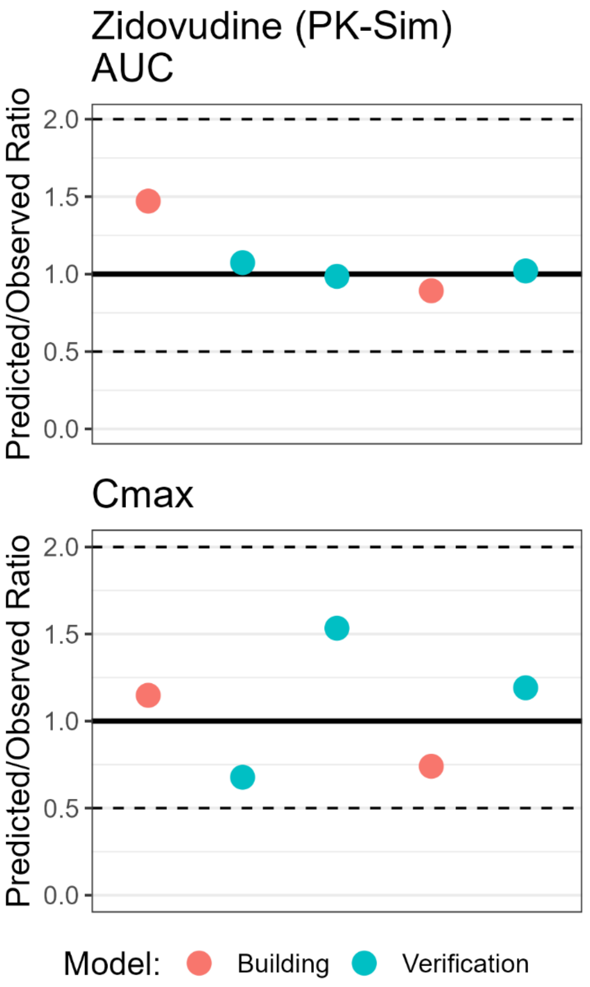

Figure S3. Predicted over observed ratio profile

Table S8 Ratio between the predicted and observed pharmacokinetic parameters of zidovudine in different dosing regimens for model building

Study ID/ Reference	Dose/ Route	AUC_{obs} (mg*h/L)	AUC_{pred} (mg*h/L)	Fold error	$Cmax_{obs}$ (mg/L)	$Cmax_{pred}$ (mg/L)	Fold error
Moore 1995 [24]	120 mg IV SD	1.04	1.53	1.47	1.42	1.63	1.15
Moore 1995 [24]	200 mg PO SD	1.58	1.41	0.89	1.85	1.37	0.74

Table S9 Ratio between the predicted and observed pharmacokinetic parameters of zidovudine in different dosing regimens used for model verification

Study ID/ Reference	Dose/ Route	AUC_{obs} (mg*h/L)	AUC_{pred} (mg*h/L)	Fold error	$Cmax_{obs}$ (mg/L)	$Cmax_{pred}$ (mg/L)	Fold error
McNab 1993 [17]	200 mg IV SD	2.30	2.47	1.07	3.07	2.08	0.68
McNab 1993 [17]	200 mg PO SD	1.39	1.37	0.99	0.90	1.38	1.53
Laskin 1989 [18]	200 mg/4h PO MD	1.48	1.51	1.02	1.15	1.37	1.19

4.2 Concentration-time profiles

Simulated versus observed concentration-time profiles of all data listed in section 3.2.2 are presented below. The original.pksim5 are provided in Supplemented material 3.

4.2.1 Model building

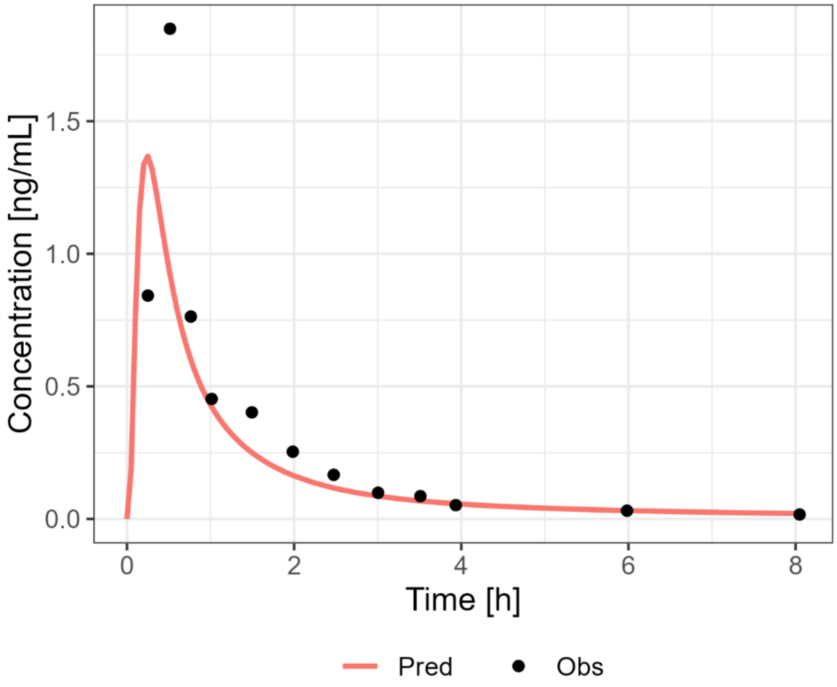

Figure S4 Predicted (Pred) versus observed (Obs) concentration-time profile after administration of 200 mg PO [24]

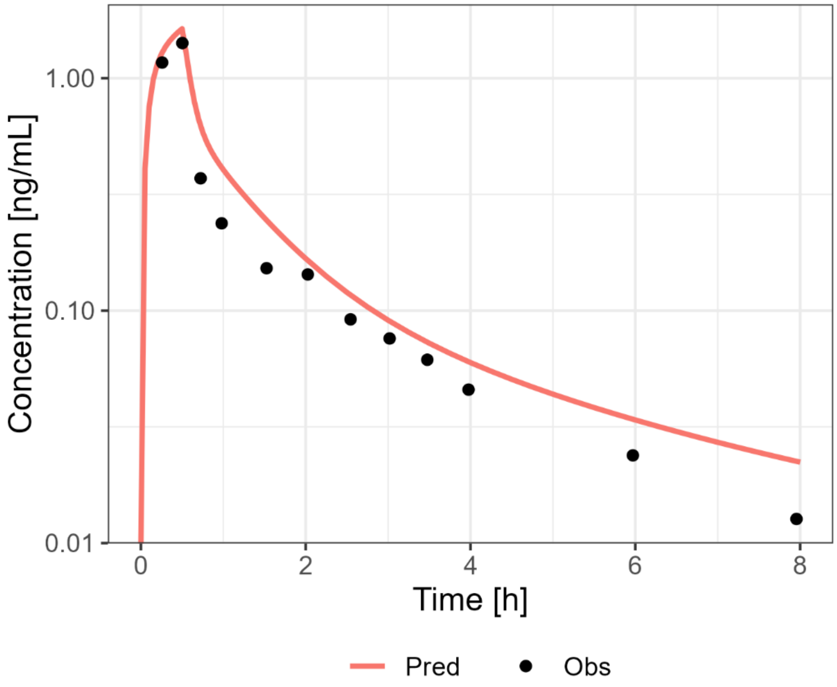

Figure S5 Predicted (Pred) versus observed (Obs) concentration-time profile after administration of 120 mg IV [24]

4.2.2 Model verification

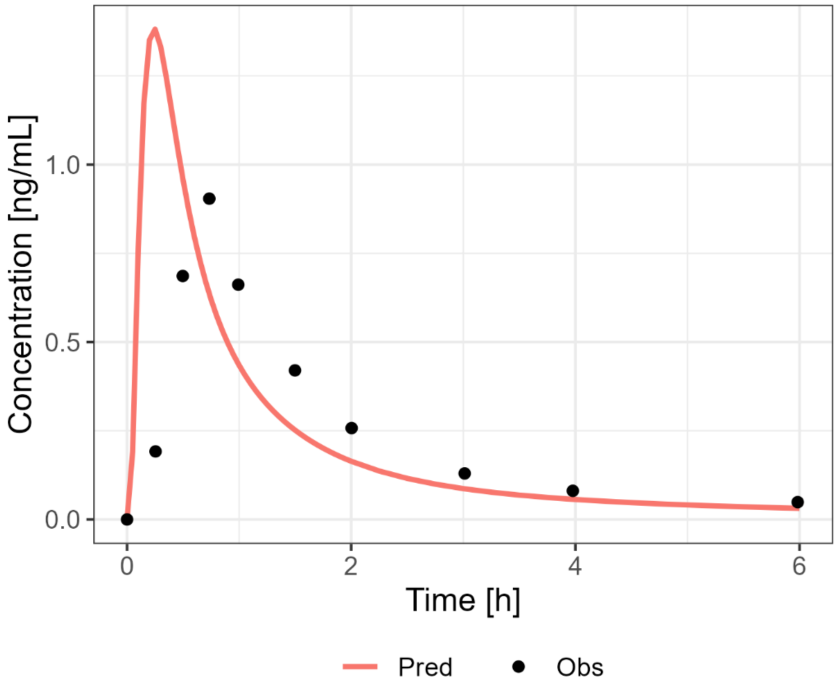

Figure S6 Predicted (Pred) versus observed (Obs) concentration-time profile after administration of 200 mg PO [17]

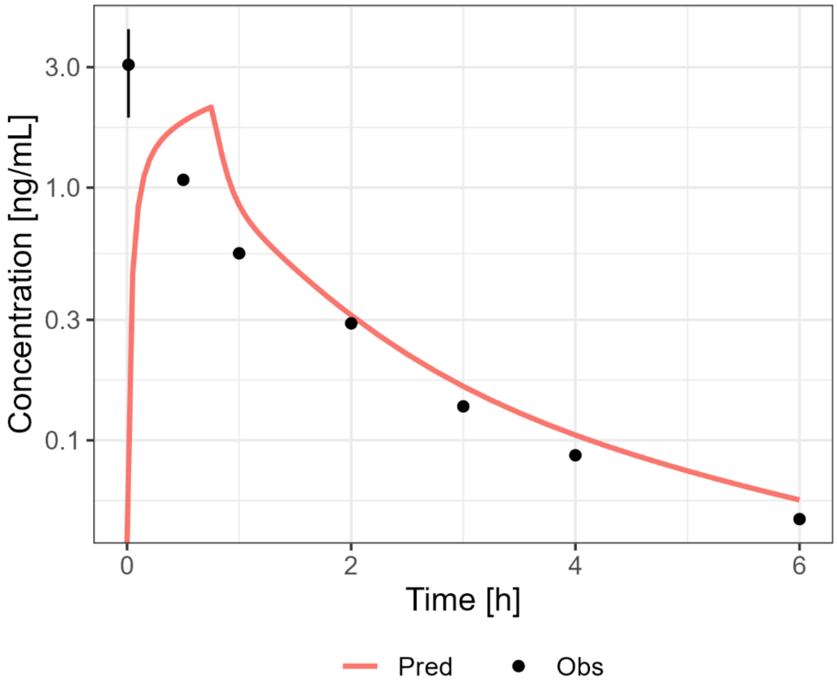

Figure S7 Predicted (Pred) versus observed (Obs) concentration-time profile after administration of 200 mg IV [17]

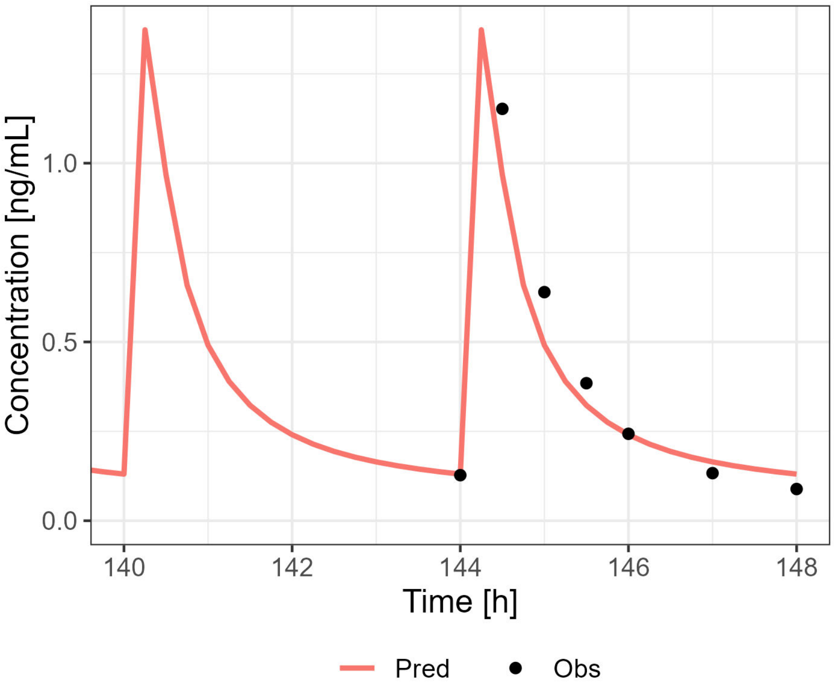

Figure S8 Predicted (Pred) versus observed (Obs) concentration-time profile after administration of 200 mg PO MD [18]

4.2.3 Lactation PBPK model

A sample size of 1000 individuals, three months postpartum, was used in each simulation of the virtual lactation population.

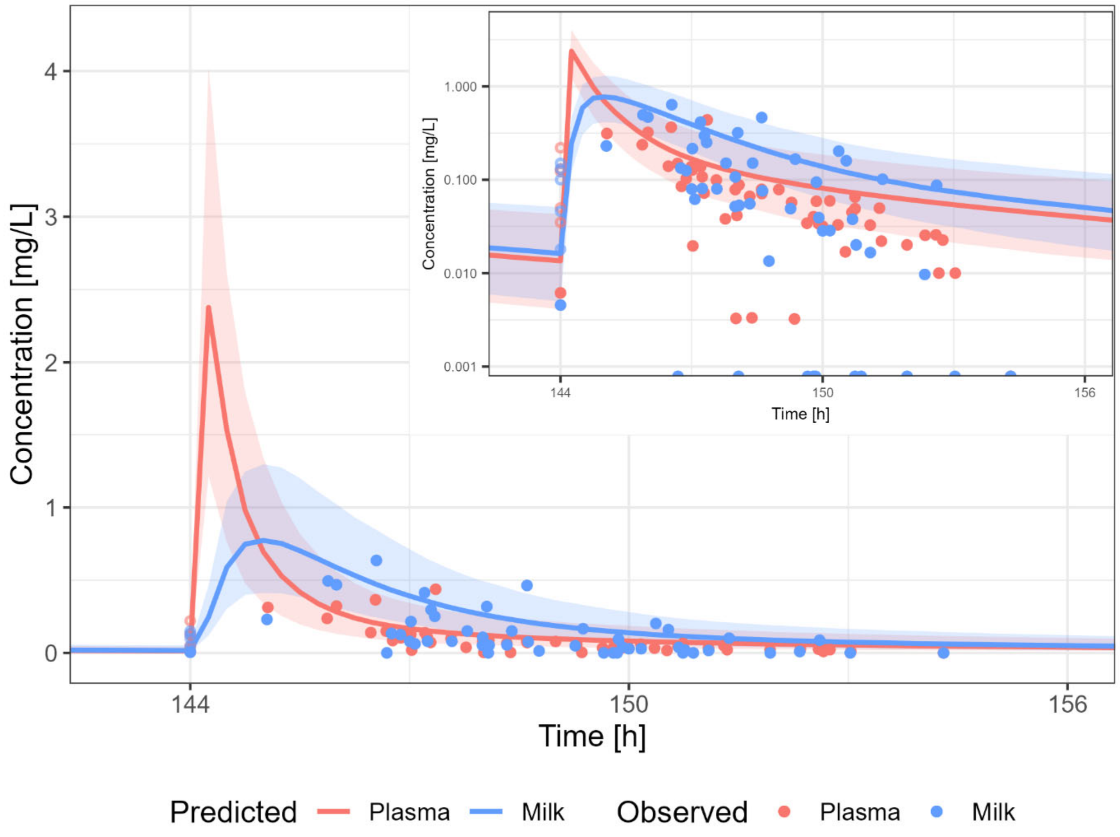

Figure S9 Predicted (Pred) versus observed (Obs) concentration-time profile after administration of 300 mg bid PO [20–22,26,27]

A dosing regimen of PO 300 mg bidaily was assumed to calculate the milk transfer of zidovudine.

Dosing interval: 12 h	Plasma	Milk
Cmax (mg/L)	2.4	0.77
AUC (mg*h/L)	2.93	3.21
Cave (mg/L)	0.24	0.27

M/P ratio = 1.10

4.3 Estimated infant dosage

A maternal dosing regimen of PO 300 mg bidaily was used to calculate the infant dosage. The daily infant dosage and relative infant dose (RID) for 3 months old infant were calculated using a milk intake of 150 mL/kg/day. The daily infant dosage was 0.04 mg/kg/day (RID: 0.40 %) or 0.12 mg/kg/day (RID: 1.16 %) based on the average steady-state concentration and maximum concentration in human milk, respectively.

5. Discussion

First, the reference PBPK model was developed and evaluated. Evaluation of the predictive performance showed that the reference PBPK model for zidovudine was able to capture the pharmacokinetic behavior of the medicines in healthy volunteers and/or patients.

Next, the PBPK model was extended to a lactation PBPK model. The PBPK model results in an acceptable prediction of the human milk concentrations, with most datapoints within the 5-95th percentile of the population prediction.

The predicted M/P ratio was within the observed range of M/P ratios (0.3-3.21).

The calculated infant dosage of zidovudine via breastfeeding was low, especially when compared to the maternal daily dosage.

6. Conclusions

The herein presented PBPK model adequately describes the PK of zidovudine in adults including breastfeeding women. In particular, it applies quantitative metabolism by glucuronidation via UGT2B7 and reduction via CYB5R4, and renal excretion via GFR and tubular secretion. The PBPK model was able to predict the human milk concentrations of zidovudine (M/P ratio: 1.10). The daily infant dosage was 0.04 mg/kg/day (RID: 0.40 %) or 0.12 mg/kg/day (RID: 1.16 %) based on the average steady-state concentration and maximum concentration in human milk, respectively.

7. List of Appendix and Supplementary Materials

Supplementary material 1 – ObsDataPK_OSP_reference_zidovudine
Supplementary material 2 – ObsDataPK_OSP_lactation_zidovudine
Supplementary material 3 – Zidovudine.pksim5

8. References

1. Fischl, M.A.; Richman, D.D.; Grieco, M.H.; Gottlieb, M.S.; Volberding, P.A.; Laskin, O.L.; Leedom, J.M.; Groopman, J.E.; Mildvan, D.; Schooley, R.T.; et al. The Efficacy of Azidothymidine (AZT) in the Treatment of Patients with AIDS and AIDS-Related Complex. *N. Engl. J. Med.* **1987**, *317*, 185–191, doi:10.1056/NEJM198707233170401.
2. Blum, M.R.; Chittick, G.E.; Begley, J.A.; Zong, J. Steady-State Pharmacokinetics of Emtricitabine and Tenofovir Disoproxil Fumarate Administered Alone and in Combination in Healthy Volunteers. *J. Clin. Pharmacol.* **2007**, *47*, 751–759, doi:10.1177/0091270007300951.
3. MIROCHNICK, M.; CAPPARELLI, E.; CONNOR, J. Pharmacokinetics of Zidovudine in Infants: A Population Analysis across Studies. *Clin. Pharmacol. Ther.* **1999**, *66*, 16–24, doi:10.1016/S0009-9236(99)70049-4.
4. Court, M.H.; Krishnaswamy, S.; Hao, Q.; Duan, S.X.; Patten, C.J.; von Moltke, L.L.; Greenblatt, D.J. EVALUATION OF 3′-AZIDO-3′-DEOXYTHYMIDINE, MORPHINE, AND CODEINE AS PROBE SUBSTRATES FOR UDP-GLUCURONOSYLTRANSFERASE 2B7 (UGT2B7) IN HUMAN LIVER MICROSOMES: SPECIFICITY AND INFLUENCE OF THE UGT2B7 * 2 POLYMORPHISM. *Drug Metab. Dispos.* **2003**, *31*, 1125–1133, doi:10.1124/dmd.31.9.1125.
5. Pan-Zhou, X.R.; Cretton-Scott, E.; Zhou, X.J.; Yang, M.X.; Lasker, J.M.; Sommadossi, J.P. Role of Human Liver P450s and Cytochrome B5 in the Reductive Metabolism of 3'-Azido-3'-Deoxythymidine (AZT) to 3'-Amino-3'-Deoxythymidine. *Biochem. Pharmacol.* **1998**, *55*, 757–766, doi:10.1016/S0006-2952(97)00538-8.
6. TGF Alpha - Wikipedia Available online: https://en.wikipedia.org/wiki/TGF_alpha (accessed on 3 January 2020).
7. Dallmann, A.; Solodenko, J.; Ince, I.; Eissing, T. Applied Concepts in PBPK Modeling: How to Extend an Open Systems Pharmacology Model to the Special Population of Pregnant Women. *CPT pharmacometrics Syst. Pharmacol.* **2018**, *7*, 419–431, doi:10.1002/PSP4.12300.
8. Dallmann, A.; Himstedt, A.; Solodenko, J.; Ince, I.; Hempel, G.; Eissing, T. Integration of Physiological Changes during the Postpartum Period into a PBPK Framework and Prediction of Amoxicillin Disposition before and Shortly after Delivery. *J. Pharmacokinet. Pharmacodyn.* **2020**, *47*, 341–359, doi:10.1007/s10928-020-09706-z.
9. Job, K.M.; Dallmann, A.; Parry, S.; Saade, G.; Haas, D.M.; Hughes, B.; Berens, P.; Chen, J.; Fu, C.; Humphrey, K.; et al. Development of a Generic Physiologically-Based Pharmacokinetic Model for Lactation and Prediction of Maternal and Infant Exposure to Ondansetron via Breast Milk. *Clin. Pharmacol. Ther.* **2022**, *111*, 1111–1120, doi:10.1002/cpt.2530.
10. Salem, F.; Small, B.G.; Johnson, T.N. Development and Application of a Pediatric Mechanistic Kidney Model. *CPT Pharmacometrics Syst. Pharmacol.* **2022**, *11*, 854–866, doi:10.1002/psp4.12798.
11. Walsky, R.L.; Bauman, J.N.; Bourcier, K.; Giddens, G.; Lapham, K.; Negahban, A.;

Ryder, T.F.; Obach, R.S.; Hyland, R.; Goosen, T.C. Optimized Assays for Human UDP-Glucuronosyltransferase (UGT) Activities: Altered Alamethicin Concentration and Utility to Screen for UGT Inhibitors. *Drug Metab. Dispos.* **2012**, *40*, 1051–1065, doi:10.1124/dmd.111.043117.
12. Koshimichi, H.; Ito, K.; Hisaka, A.; Honma, M.; Suzuki, H. Analysis and Prediction of Drug Transfer into Human Milk Taking into Consideration Secretion and Reuptake Clearances across the Mammary Epithelia. *Drug Metab. Dispos.* **2011**, *39*, 2370–2380, doi:10.1124/dmd.111.040972.
13. Zhang, Z.; Unadkat, J.D. Development of a Novel Maternal-Fetal Physiologically Based Pharmacokinetic Model II: Verification of the Model for Passive Placental Permeability Drugs. *Drug Metab. Dispos.* **2017**, *45*, 939–946, doi:10.1124/dmd.116.073957.
14. Beach, J.W. Chemotherapeutic Agents for Human Immunodeficiency Virus Infection: Mechanism of Action, Pharmacokinetics, Metabolism, and Adverse Reactions. *Clin. Ther.* **1998**, *20*, 2–25, doi:10.1016/S0149-2918(98)80031-3.
15. Atkinson, U.C.; Begg, E.J.E.J.; Atkinson, H.C.; Begg, E.J.E.J. Prediction of Drug Distribution into Human Milk from Physicochemical Characteristics. *Clin. Pharmacokinet.* **1990**, *18*, 151–167, doi:10.2165/00003088-199018020-00005.
16. Moore, K.H.; Raasch, R.H.; Brouwer, K.L.; Opheim, K.; Cheeseman, S.H.; Eyster, E.; Lemon, S.M.; van der Horst, C.M. Pharmacokinetics and Bioavailability of Zidovudine and Its Glucuronidated Metabolite in Patients with Human Immunodeficiency Virus Infection and Hepatic Disease (AIDS Clinical Trials Group Protocol 062). *Antimicrob. Agents Chemother.* **1995**, *39*, 2732–2737, doi:10.1128/aac.39.12.2732.
17. Macnab, K.A.; Gill, M.J.; Sutherland, L.R.; De Boer Visser, N.; Church, D. Erratic Zidovudine Bioavailability in HIV Seropositive Patients. *J. Antimicrob. Chemother.* **1993**, *31*, 421–428, doi:10.1093/jac/31.3.421.
18. Laskin, O.L.; De Miranda, P.; Blum, M.R. Azidothymidine Steady-State Pharmacokinetics in Patients with AIDS and AIDS-Related Complex. *J. Infect. Dis.* **1989**, *159*, 745–747, doi:10.1093/infdis/159.4.745.
19. Corbett, A.H.; Kayira, D.; White, N.R.; Davis, N.L.; Kourtis, A.P.; Chasela, C.; Martinson, F.; Phiri, G.; Musisi, B.; Kamwendo, D.; et al. Antiretroviral Pharmacokinetics in Mothers and Breastfeeding Infants from 6 to 24 Weeks Postpartum: Results of the Ban Study. *Antivir. Ther.* **2014**, *19*, 587–595, doi:10.3851/IMP2739 LK - http://limo.libis.be/resolver?&sid=EMBASE&issn=20402058&id=doi:10.3851%2FIMP2739&atitle=Antiretroviral+pharmacokinetics+in+mothers+and+breastfeeding+infants+from+6+to+24+weeks+postpartum%3A+Results+of+the+ban+study&stitle=Antiviral+Ther.&title=Antiviral+Therapy&volume=19&issue=6&spage=587&epage=595&aulast=Corbett&aufirst=Amanda+H.&auinit=A.H.&aufull=Corbett+A.H.&coden=ANTHF&isbn=&pages=587-595&date=2014&auinit1=A&auinitm=H.
20. Giuliano, M.; Guidotti, G.; Andreotti, M.; Pirillo, M.F.; Villani, P.; Liotta, G.; Marazzi, M.C.; Mancini, M.G.; Cusato, M.; Germano, P.; et al. Triple Antiretroviral Prophylaxis Administered during Pregnancy and after Delivery Significantly Reduces Breast Milk Viral Load: A Study within the Drug Resource Enhancement against AIDS and Malnutrition Program. *J. Acquir. Immune Defic. Syndr.* **2007**, *44*, 286–291, doi:10.1097/QAI.0b013e31802c5441.
21. Mirochnick, M.; Thomas, T.; Capparelli, E.; Zeh, C.; Holland, D.; Masaba, R.; Odhiambo, P.; Fowler, M.G.; Weidle, P.J.; Thigpen, M.C. Antiretroviral Concentrations in Breast-Feeding Infants of Mothers Receiving Highly Active

Antiretroviral Therapy. *Antimicrob. Agents Chemother.* **2009**, *53*, 1170–1176, doi:10.1128/AAC.01117-08.

22. Palombi, L.; Pirillo, M.F.; Andreotti, M.; Liotta, G.; Erba, F.; Sagno, J.B.; Maulidi, M.; Ceffa, S.; Jere, H.; Marchei, E.; et al. Antiretroviral Prophylaxis for Breastfeeding Transmission in Malawi: Drug Concentrations, Virological Efficacy and Safety. *Antivir. Ther.* **2012**, *17*, 1511–1519, doi:10.3851/IMP2315.
23. R.L., S.; D.T., H.; E., C.; S., L.; I., T.; C., W.; L., S.; T., P.; M., E.; J.D., C.; et al. Antiretroviral Concentrations in Breast-Feeding Infants of Women in Botswana Receiving Antiretroviral Treatment. *J. Infect. Dis.* **2005**, *192*, 720–727, doi:10.1086/432483 LK - http://limo.libis.be/resolver?&sid=EMBASE&issn=00221899&id=doi:10.1086%2F432483&atitle=Antiretroviral+concentrations+in+breast-feeding+infants+of+women+in+Botswana+receiving+antiretroviral+treatment&stitle=J.+Infect.+Dis.&title=Journal+of+Infectious+Diseases&volume=192&issue=5&spage=720&epage=727&aulast=Shapiro&aufirst=Roger+L.&auinit=R.L.&aufull=Shapiro+R.L.&coden=JIDIA&isbn=&pages=720-727&date=2005&auinit1=R&auinitm=L.
24. Moore, K.H.; Raasch, R.H.; Brouwer, K.L.; Opheim, K.; Cheeseman, S.H.; Eyster, E.; Lemon, S.M.; van der Horst, C.M. Pharmacokinetics and Bioavailability of Zidovudine and Its Glucuronidated Metabolite in Patients with Human Immunodeficiency Virus Infection and Hepatic Disease (AIDS Clinical Trials Group Protocol 062). *Antimicrob. Agents Chemother.* **1995**, *39*, 2732–2737, doi:10.1128/AAC.39.12.2732.
25. Dezani, A.B.; Pereira, T.M.; Caffaro, A.M.; Reis, J.M.; Serra, C.H. dos R. Determination of Lamivudine and Zidovudine Permeability Using a Different Ex Vivo Method in Franz Cells. *J. Pharmacol. Toxicol. Methods* **2013**, *67*, 194–202, doi:10.1016/j.vascn.2013.01.005.
26. Corbett, A.H.; Kayira, D.; White, N.R.; Davis, N.L.; Kourtis, A.P.; Chasela, C.; Martinson, F.; Phiri, G.; Musisi, B.; Kamwendo, D.; et al. Antiretroviral Pharmacokinetics in Mothers and Breastfeeding Infants from 6 to 24 Weeks Post-Partum: Results of the Ban Study. *Antivir. Ther.* **2014**, *19*, 587–595, doi:10.3851/IMP2739.
27. Shapiro, R.L.; Holland, D.T.; Capparelli, E.; Lockman, S.; Thior, I.; Wester, C.; Stevens, L.; Peter, T.; Essex, M.; Connor, J.D.; et al. Antiretroviral Concentrations in Breast-Feeding Infants of Women in Botswana Receiving Antiretroviral Treatment. *J. Infect. Dis.* **2005**, *192*, 720–727, doi:10.1086/432483.
