# Supplementary material for: Generic Workflow to Predict Medicine Concentrations in Human Milk Using Physiologically-Based Pharmacokinetic (PBPK) Modelling—A Contribution from the ConcePTION Project
Source: Pharmaceutics. 2023 May 11;15(5):1469. doi: 10.3390/pharmaceutics15051469 (PMC10223637; doi:10.3390/pharmaceutics15051469)
Supplement: Supplementary file 1 [file pharmaceutics-15-01469-s001.zip › File S2. Evaluation_report_caffeine.pdf]

Building and evaluation of a Physiologically-Based PharmacoKinetic (PBPK) model for caffeine in adults and lactating women

Disclaimer: The research project leading to these results was conducted as part of the ConcePTION consortium. This report only reflects the personal views of the stated authors. The results of this report are only intended for research purpose, and are not intended to be used in clinical practice.

Glossary

AUC	Area Under the Curve
Cave	Average concentration
CL_{re}	Reuptake clearance (i.e. from milk to blood)
CL_{sec}	Secretion clearance (i.e. from blood to milk)
Cmax	Maximum (~peak) concentration
DID	Daily Infant Dosage (expressed for instance in mg/kg/day)
f_u	Fraction unbound in plasma
GFR	Glomerular Filtration Rate
HBD	Hydrogen Bond Donors
IV	Intravenous administration
$LogD_{7.2}$	Logarithm of the partition coefficient between an octanol phase and an aqueous (buffer) phase at pH 7.2
$LogD_{7.4}$	Logarithm of the partition coefficient between an octanol phase and an aqueous (buffer) phase at pH 7.4
LogP	Logarithm of the partition coefficient between an octanol phase and (unbuffered) water as aqueous phase. This is the default parameter to express lipophilicity of a substance.
MD	Multiple dose
M/P ratio	Milk-to-Plasma ratio
MW	Molecular Weight (Da)
PBPK	Physiologically Based PharmacoKinetic [modeling]
pKa	Logarithm of the acid dissociation constant
PO	Oral administration
PSA	Polar Surface Area
RID	Relative Infant Dose (%)
RT-PCR	Reverse transcription polymerase chain reaction
SD	Single dose

1. Table of Contents

1. Table of Contents ***3***

2. Introduction ***4***

3. Methods ***5***

3.1 Modelling strategy **5**

3.1.1 Reference PBPK models 6

3.1.2 Lactation model 6

3.2 Data **6**

3.2.1 *In vitro* / physicochemical data 6

3.2.2 Clinical data 9

3.3 Model Parameters and assumptions **10**

3.3.1 Absorption 10

3.3.2 Distribution 10

3.3.3 Metabolism and excretion 10

3.3.4 Secretion to milk 10

3.4. Infant dosage calculation **11**

4. Results ***12***

4.1 Final input parameters **12**

4.2 Diagnostic plots **13**

4.3 Concentration-time profiles **15**

4.3.1 Model building and verification 15

4.3.2 Lactation PBPK model 19

4.4 Estimated infant dosage **39**

5. Discussion ***39***

6. Conclusion ***40***

7. List of Appendix and Supplementary Materials ***41***

8. References ***41***

2. Introduction

Caffeine is a methylxanthine and stimulant of the central nervous system [1]. The absorption is rapidly, reaching a peak concentration after 0.5-2 hours. The bioavailability in adults is 100 %. The volume of distribution is 0.6 L/kg in adults. Protein binding is 30 %. Metabolism of caffeine is mainly hepatic, via cytochrome P450 1A2. Only 2 % of the parent is found in urine, as caffeine is absorbed in the renal tubule. The half-life of caffeine is 5 hours in adults.

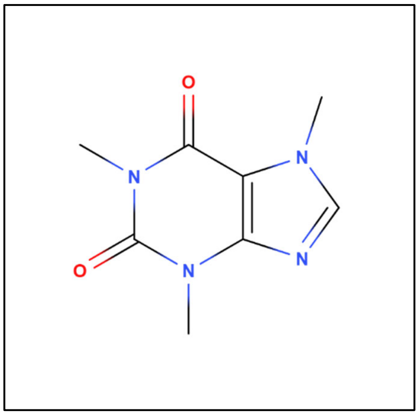

Figure S1. Chemical structure of caffeine

The scope of this report is to:

(a) specify the details and underlying assumptions associated with the building of physiologically-based pharmacokinetic (PBPK) models for caffeine in adult healthy volunteers or patients, and in postpartum women during lactation.

(b) evaluate the predictive performance of these PBPK models. This is achieved by comparing model-predicted plasma or milk concentrations with corresponding clinical observations.

3. Methods

The software used for the development of PBPK models presented in this report is tabulated below:

Software	Version
PK-Sim®	v9.1
MoBi®	v9.1

3.1 Modelling strategy

In the present report, a reference PBPK model was first established for adults (patients as well as healthy volunteers), and subsequently verified against clinical pharmacokinetic data reported for caffeine in the scientific literature.

Relevant information on the anthropometry (height, weight) was gathered from the respective clinical studies, if reported. Information on physiological parameters (e.g. blood flows, organ volumes, hematocrit) in adults is available in the PK-Sim® database.

In a second step, a lactation PBPK model was developed, based on the general workflow described by Dallmann *et al. 2018* [2–4].

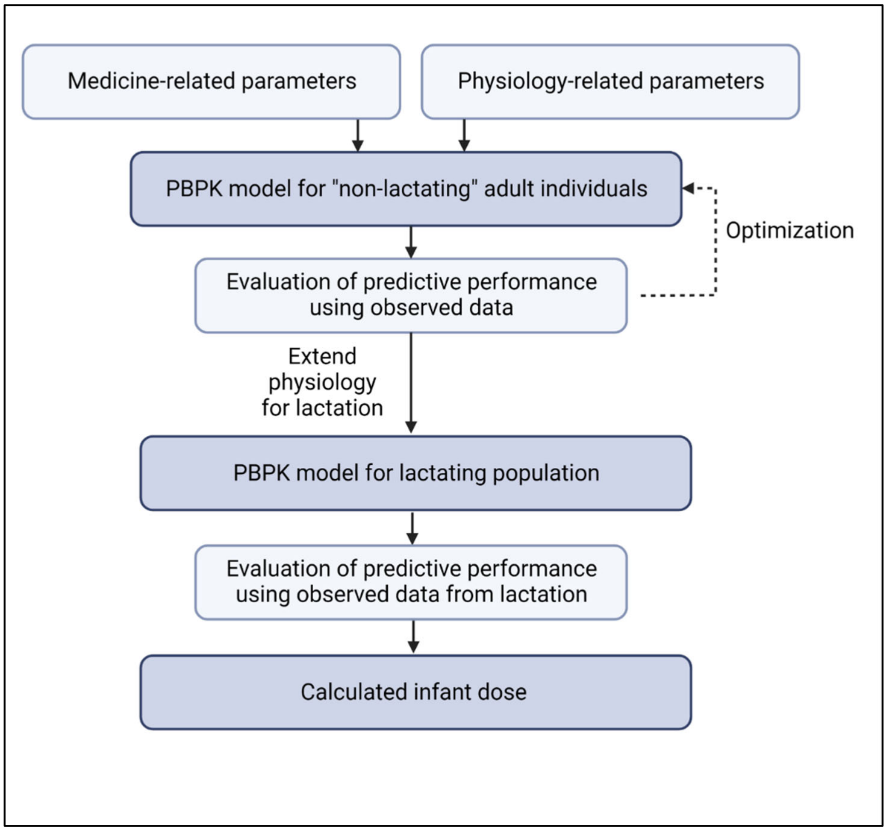

Figure S2. General workflow that was used in the present project to develop and evaluate the lactation PBPK model.

Details about input data (physicochemical, *in vitro* and clinical data) can be found in section 3.2. Details about the structural models and their parameters can be found in section 3.3.

3.1.1 Reference PBPK models

The reference PBPK models for caffeine were taken from the template database in PK-Sim® (https://github.com/Open-Systems-Pharmacology/Example_Caffeine).

The abundance (including population variability) of plasma proteins and enzymes/transporters that are integrated into PK-Sim® are described in the publicly available 'PK-Sim® Ontogeny Database Version 7.3' (PK-Sim Ontogeny Database Version 7.3).

The specific metabolic clearance of caffeine was assumed to be via CYP1A2. The CYP enzymes were implemented in accordance with literature, using the PK-Sim expression database RT-PCR profiles to define their relative expression in the different organs of the body. Renal excretion was implemented as kidney plasma clearance.

Structural model selection was mainly guided by biological plausibility and by visual inspection of the predicted concentration time profiles in comparison with observed data. The generally applied acceptance criterium was less than 2-fold misprediction.

The predictive performance of the models was evaluated by simulating:

- Intravenous and oral administration of 3-5 mg/kg
- Oral administration of 250-500 mg

3.1.2 Lactation model

After development of the reference model, the model was exported to MoBi® and a lactation PBPK model was constructed. To model the passage of caffeine into human milk, i.e. across the blood/milk biological barrier, both the secretion (CL_{sec}) and reuptake clearance (CL_{re}) values were obtained using the empirical model developed by Koshimichi *et al. 2011* [5].

3.2 Data

3.2.1 *In vitro* / physicochemical data

A literature search was performed to collect available information on physicochemical properties of caffeine. The obtained information from literature is summarized in **Error! Reference source not found.**. **Error! Reference source not found.** shows the parameters that were additionally used for the lactation PBPK model.

Table S1. Physicochemical parameters used as input for the caffeine PBPK models

**Parameter**	**Value**	**Unit**	**Description**	**Source**
MW	194.20	g/mol	Molecular weight	Drugbank
pK_a	0.80 (base)	-	Logarithm of the acid dissociation constant	

Solubility (pH 7)	21.60	mg/mL	Aqueous solubility	
Log P	-0.07	-	Log_{10} of the partition coefficient between octanol and water (~lipophilicity)	Drugbank
f_u	0.7	-	Fraction unbound in human plasma	[6]
CYP1A2:<br>- Km<br>- kcat	 14.70<br>1.01	 µmol/L<br>min^{-1}	Michaelis-Menten constant & catalytic rate constant	Parameter identification
Renal clearance – specific clearance	2.46E-03	min^{-1}	Rate constant for the renal plasma clearance (first order) process	[7]

Table S2. Physicochemical parameters used as input for the lactation PBPK model of caffeine

Parameter	Value	Unit	Description	Source
Milk logP[a]	-0.07	-	Log10 of the partition coefficient between octanol and water	Drugbank
HBD	0	-	Hydrogen bond donors	Pubchem
PSA	58.44	$Å^2$	Polar surface area	Pubchem

[a] Milk logP is Log_{10} of the partition coefficient between octanol and water and is used as input for the calculations in the postpartum model (see equations below). In theory, this value is identical to the logP specified in Table S1. However, in some PBPK models, logP (Table S1) might be optimized using parameter identication. Therefore, it was chosen to use a separate parameter (i.e. Milk logP) to represent the logP used as input for the equations in the postpartum model.

The default equations for free fraction in human milk and logD that were implemented in the spatial structure building block that was developed for the postpartum women are described below. Alternatively, these values can be overwritten by values calculated elsewhere (e.g. MarvinSketch) or determined *in vitro*.

The free fraction in human milk was calculated with the equations proposed by Atkinson and Begg [8], as follows:

$$f_{u\_skimmed\ milk} = \frac{f_u \times 0.448}{(0.000694^{0.448} + f_u^{0.448})}$$

$$P_{milk} = 10^{(-0.88+1.29\times \log D7.2)}$$

$$\text{Total free fraction in milk} = \frac{1}{(\frac{0.955}{f_{u\_skimmed\ milk}} + 0.045 \times P_{milk})}$$

Where: f_u *skimmed milk:* binding to proteins in milk; P_{milk}: partitioning between aqueous and lipid phase of milk; Total free fraction in milk: 'total' free fraction, i.e. accounting for both protein and lipid binding processes.

LogD values taking into account up to three pka values (as provided in the compound building block), were calculated as follows:

$$LogD = \text{Log}\,P + Log_{10}(logD_{factor})$$

With Milk logP (Table S2) as input for logP

$$LogD_{factor} = K_1 + (K_2 + K_3 + K_4) \times \text{base}^1 + K_5 \times \text{base}^{\max(CT_0+CT_1;-CT_0-CT_1)} + K_6 \times \text{base}^{\max(CT_0+CT_2;-CT_0-CT_2)} + K_7 \times \text{base}^{\max(CT_2+CT_1;-CT_2-CT_1)} + K_8 \times \text{base}^{\max(CT_0+CT_1+CT_2;-CT_0-CT_1-CT_2)}$$

$$K_1 = F_1 \times F_2 \times F_3$$
$$K_2 = (1 - F_1) \times F_2 \times F_3$$
$$K_3 = F_1 \times (1 - F_2) \times F_3$$
$$K_4 = F_1 \times F_2 \times (1 - F_3)$$
$$K_5 = (1 - F_1) \times (1 - F_2) \times F_3$$
$$K_6 = (1 - F_1) \times F_2 \times (1 - F_3)$$
$$K_7 = (1 - F_1) \times F_2 \times (1 - F_3)$$
$$K_8 = (1 - F_1) \times (1 - F_2) \times (1 - F_3)$$

F1 = CT0 != CT_NEUTRAL ? 1/(1+10^(CT0*(pKa_0- pH))) : 1
F2 = CT1 != CT_NEUTRAL ? 1/(1+10^(CT1*(pKa_1- pH))) : 1
F3 = CT2 != CT_NEUTRAL ? 1/(1+10^(CT2*(pKa_2- pH))) : 1

With CT = compound type (-1: acid; +1: base; 0: neutral), and pH = 7.2 or 7.4 respectively for $logD_{7.2}$ *and* $logD_{7.4}$

The transports that were added in the passive transport building block for 'transfer to milk' and 'transfer from milk' are based on secretion and reuptake and clearance values, Cl_{sec} and Cl_{re}, which were calculated according to the empirical equations proposed by Koshimichi et al. 2011 [5], as follows:

$$\text{Log}\,CL_{re} = 2.793 + 0.179 \times LogP - 0.132 \times HBD$$

$$\text{Log}\,CL_{sec} = 3.367 \times Log10(MW) - 0.164 \times (LogP - LogD) - 0.015 \times PSA - 3.912$$

3.2.2 Clinical data

Literature searches were performed to collect available data on caffeine in adults and postpartum women. The caffeine PBPK model was taken from the PK-Sim template database. They performed simulations for 5 clinical trials with intravenous and oral administration [9–13].

The evaluation of the predictive performance of the caffeine lactation PBPK model was performed using 7 different studies were caffeine was administered as single or multiple doses to lactating women [14–20]. The women were between 11 days and 1 year postpartum.

Detailed information and data from the studies used for model building, verification, and lactation model can be found in Supplementary material 1 and 2.

3.2.2.1 Model building and verification

The studies that were used for model building and evaluation of the predictive performance in the template are shown in **Error! Reference source not found.**.

Table S3. Summary of studies used for PBPK model building and verification of caffeine in reference populations.

Study ID	Reference	Arm/treatment/information used for model building and verification
Wahllander 1989	[13]	8 subjects received IV 3 mg/kg (single dose)
Blanchard 1983	[9]	8 subjects received IV 5 mg/kg (single dose)
Blanchard 1983	[9]	8 subjects received PO 5 mg/kg
Cysneiros 2007	[10]	12 subjects received PO 250 mg
Cysneiros 2007	[10]	12 subjects received PO 500 mg
Kaplan 1997	[11]	12 subjects received PO 250 mg
Maish 1996	[12]	10 subjects received PO 3 mg/kg
Maish 1996	[12]	6 subjects received PO 3 mg/kg
Maish 1996	[12]	10 subjects received PO 3 mg/kg

Table S4 Demographic information

Study ID	Reference	Number of subjects (female ratio)	Age (year)	Weight (kg)
Wahllander 1989	[13]	8 (0)	**-**	**-**
Blanchard 1983	[9]	8 (0)	20.45 (18.8-24)	74.36 ± 5.81
Cysneiros 2007	[10]	12 (-)	- (18-35)	-
Kaplan 1997	[11]	12 (0.58)	28.83 (20-46)	67.06 (-)
Maish 1996	[12]	10 (-)	28 ± 4.4	77.7 ±7.8

3.2.2.2 Lactation PBPK model

Error! Reference source not found. shows the study that was used for the lactation PBPK model.

Table S5. Summary of study used for PBPK model development of caffeine in lactating women

Study ID	Publication	Arm/treatment/information used for model building and verification
Bailey 1982	[14]	1 (smoking) woman received PO different doses (multiple dose)
Calvaresi 2016	[15]	1 woman (3 months postpartum) received PO 80 mg (single dose)
Findlay 1981	[16]	1 woman (7 weeks postpartum) received PO 64 mg (single dose)
Ryu 1985a	[17]	11 women (13 weeks postpartum) received PO 100 mg (multiple dose)
Ryu 1985b	[18]	9 women (11 - 127 days postpartum) received PO 150 mg (multiple dose)
Stavchansky 1988	[19]	6 women (3.5 – 17 weeks postpartum) received 100 mg PO (single dose)
Tyrala 1978	[20]	5 women (4 months – 1 year postpartum) received 150 mg PO (single dose)
Tyrala 1978	[20]	1 woman (4 months – 1 year postpartum) received PO 300 mg (single dose)

3.3 Model Parameters and assumptions

3.3.1 Absorption

Caffeine is rapidly absorbed. Solution was selected as formulation for the oral administration. Intestinal permeability was optimized via parameter identification. Solubility was taken from literature.

3.3.2 Distribution

An important parameter influencing the distribution of a compound is lipophilicity. Lipophilicity (Table S1) was taken from Drugbank. The tissue partition coefficients (Kp) calculation was according to 'PK-Sim Standard' and the cellular permeability calculation was 'PK-Sim Standard'.

3.3.3 Metabolism and excretion

The final model applies metabolism by CYP1A2 and renal excretion (Table S1). CYP1A2 was implemented as *in vitro* metabolic rate in the presence of liver microsomes – Michaelis-Menten. The values were determined via parameter identification. The renal clearance was implemented as kidney plasma clearance, and taken from literature [7].

3.3.4 Secretion to milk

To model the transfer process of caffeine into human milk, both the secretion (CL_{sec}) and reuptake clearance (CL_{re}) were calculated using the empirical equations developed by Koshimichi *et al.* 2011 (see **Error! Reference source not found.**) [4].

First, in MoBi®, a spatial structure for the postpartum women was constructed, similar to the workflow from Dallmann *et al.* 2018 [2]. Here, breasts were added as a compartment. In

addition, the human milk was connected to the plasma subcompartment of the breasts. The human milk volume was specified as 0.5 L to represent the structure of Koshimichi *et al.* 2011, and a geometric standard deviation of 1.16 was assumed in the population. The free fraction in human milk, and logD values were implemented as the equations described previously. The transfer between plasma and milk was defined as two kinetic processes (transfer to milk and transfer from milk) under passive transports (see below). Next, the simulation was combined with the postpartum population from Job *et al.* 2021 in PK-Sim to account for the postpartum physiology [3].

<u>*Kinetics*</u>

Transfer to milk

$$\frac{dNmilk}{dt} = Cplasma \times f_u \times CL_{sec}$$

where Cplasma is the concentration in plasma (in breast compartment), f_u is the free fraction in plasma and CL_{sec} is the secretion clearance.

Transfer from milk

$$\frac{dNplasma}{dt} = Cmilk \times f_u \times CL_{re}$$

where Cmilk is the concentration in human milk, f_u is the total free fraction in human milk (protein and lipid) and CL_{re} is the reuptake clearance.

The median median simulated plasma and human milk concentration-time profiles can be used to calculate the M/P ratio as follows:

$$M/P\ ratio\ = \frac{AUC_{milk}}{AUC_{plasma}}$$

3.4. Infant dosage calculation

Infant dosage via human milk was then calculated based on the predicted (average and maximal) steady-state caffeine concentration in human milk, as well as the daily milk intake volume. The daily infant dosage was then compared to the maternal dosage, resulting in the relative infant dose (RID).

$$Daily\ infant\ dosage = C_{average} * 150\ \frac{mL}{kg\ .day}$$

$$Daily\ infant\ dosage = C_{max} * 150\ \frac{mL}{kg\ .day}$$

$$Relative\ infant\ dose\ (RID) = \frac{Infant\ dosage}{Maternal\ dosage} * 100\ \%$$

4. Results

Both the reference and postpartum PBPK model of caffeine was developed and verified with clinical PK data.

The models were evaluated covering studies including in particular:

- Intravenous and oral administration of 3-5 mg/kg
- Oral administration of 250-500 mg

The model describes the metabolism via CYP1A2 and renal excretion for caffeine. Moreover, secretion and reuptake to human milk were described by CL_{sec} and CL_{re}.

The next sections show:

- The final model parameters for the building blocks: section 4.1
- The overall predictive performance: section 4.2
- The simulated versus observed concentration-time profiles for the clinical studies used for model building and for model verification: section 4.3

4.1 Final input parameters

The compound values of the final postpartum PBPK model for caffeine are illustrated below.

Physicochemical parameters

Parameter	Value	Unit	Source
MW	194.20	g/mol	Drugbank
pKa	0.80 (base)	-	
Solubility	21.60	mg/mL	
Lipophilicity	-0.07	-	Drugbank
f_u	0.7	-	[6]
Small molecule (Y/N)	Yes	-	-
Plasma protein binding partner	Albumin	-	-

Calculation methods

Name	Value
Tissue partition coefficients	PK-Sim standard
Cellular permeabilities	PK-Sim standard

AMDE-related parameters

Parameter	Value	Unit	Source
Intestinal permeability	6.85E-06	dm/min	Parameter identification
CYP1A2:<br>- Km<br>- kcat	 14.70<br>1.01	 µmol/L<br>min-1	Parameter identification

Renal clearance – specific clearance	2.46E-03	min-1	[7]

Formulation-related parameters
Type: Solution

Physicochemical and physiological parameters relevant to the lactation model

**Parameter**	**Value**	**Unit**	**Source**
Milk log P	-0.07	-	Pubchem
HBD	0	-	Pubchem
PSA	58.44	$Å^2$	Pubchem
CL_{sec}	0.01	L/min	Default
CL_{re}	0.01	L/min	Default
*f*u_skimmed milk[a]	0.96	-	Default
Pmilk[b]	0.11	-	Default
Total free fraction in milk[c]	1.00	-	Default
$logD_{7.2}$	-0.07	-	Default
$logD_{7.4}$	-0.07	-	Default

[a] binding to proteins in milk; [b] partitioning between aqueous and lipid phase of milk; [c] total free fraction, accounting for both protein and lipid binding

4.2 Diagnostic plots

The geometric mean fold errors (GMFE) on AUC and Cmax were 1.25 and 1.14.
The following shows the predictive performance graph for Cmax and AUC of caffeine for the PBPK model performance of all data used.
Predicted over observed ratio values of all data listed in section 3.2.2 are presented below.

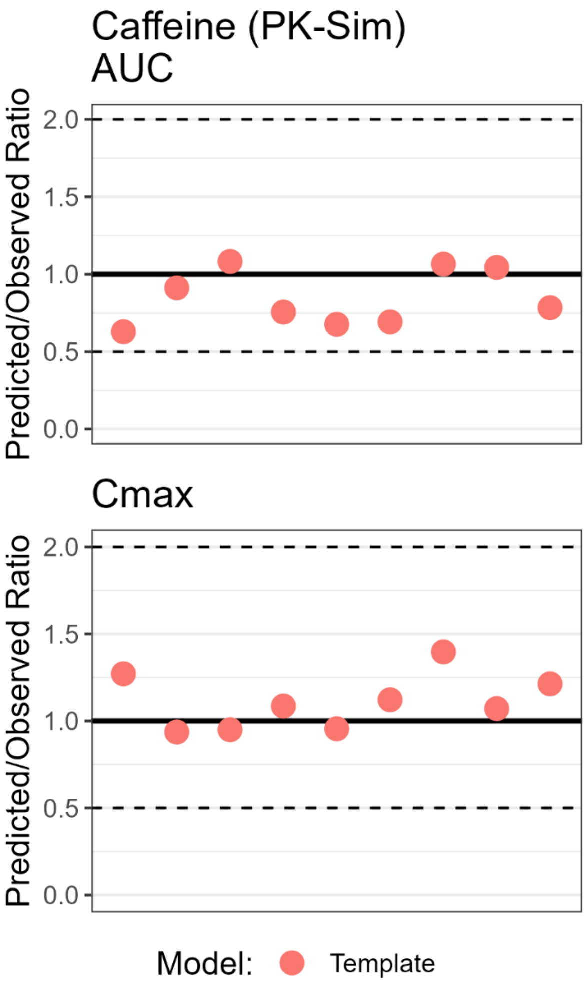

Figure S3. Predicted over observed ratio profile

Table S6. Ratio between the predicted and observed pharmacokinetic parameters of caffeine in different dosing regimens for model building.

Study ID/ Reference	Dose/ Route	AUC_{obs} (mg*h/L)	AUC_{pred} (mg*h/L)	Fold error	$Cmax_{obs}$ (mg/L)	$Cmax_{pred}$ (mg/L)	Fold error
Blanchard 1983 [9]	5 mg/kg IV SD	54.33	57.87	1.07	8.82	12.32	1.40
Wahllander 1989 [13]	3 mg/kg IV SD	24.17	26.16	1.08	4.37	4.15	0.95
Blanchard 1983 [9]	5 mg/kg PO SD	52.62	54.93	1.04	8.63	9.24	1.07
Cysneiros 2007 [10]	250 mg PO SD	55.15	34.67	0.63	4.87	6.19	1.27
Cysneiros 2007 [10]	500 mg PO SD	120.62	94.58	0.78	10.60	12.86	1.21
Kaplan 1997 [11]	250 mg PO SD	28.26	25.77	0.91	6.61	6.19	0.94

Maish 1996 [12]	3 mg/kg PO SD (1)	46.97	32.51	0.69	4.94	5.54	1.12
Maish 1996 [12]	3 mg/kg PO SD (2)	48.72	32.95	0.68	6.23	5.95	0.96
Maish 1996 [12]	3 mg/kg PO SD (3)	43.60	32.95	0.76	5.48	5.95	1.09

4.3 Concentration-time profiles

Simulated versus observed concentration-time profiles of all data listed in section 3.2.2 are presented below. The original.pksim5 are provided in Supplemented material 3.

4.3.1 Model building and verification

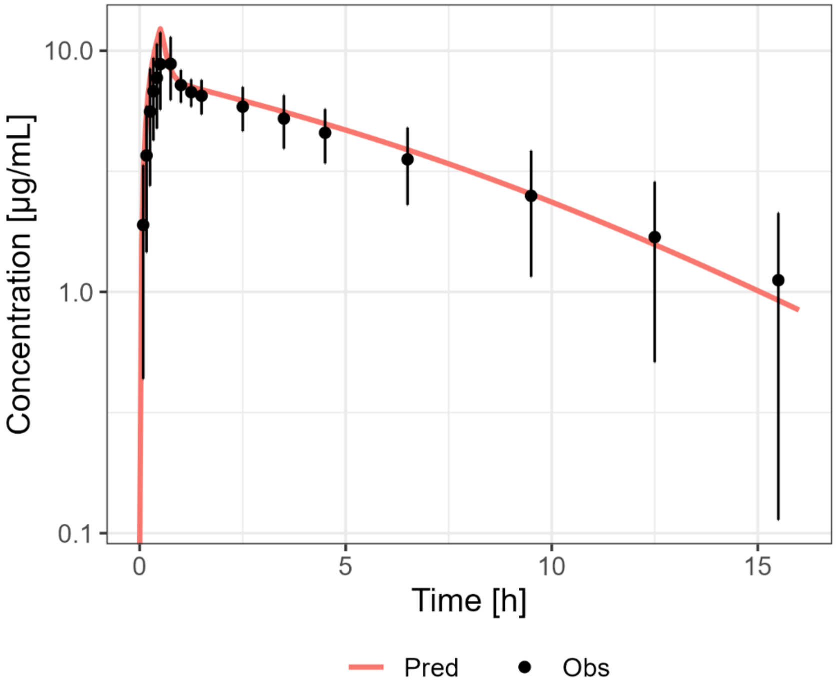

Figure S4 Predicted (Pred) versus observed (Obs) concentration-time profile after administration of 5 mg/kg IV SD [9]

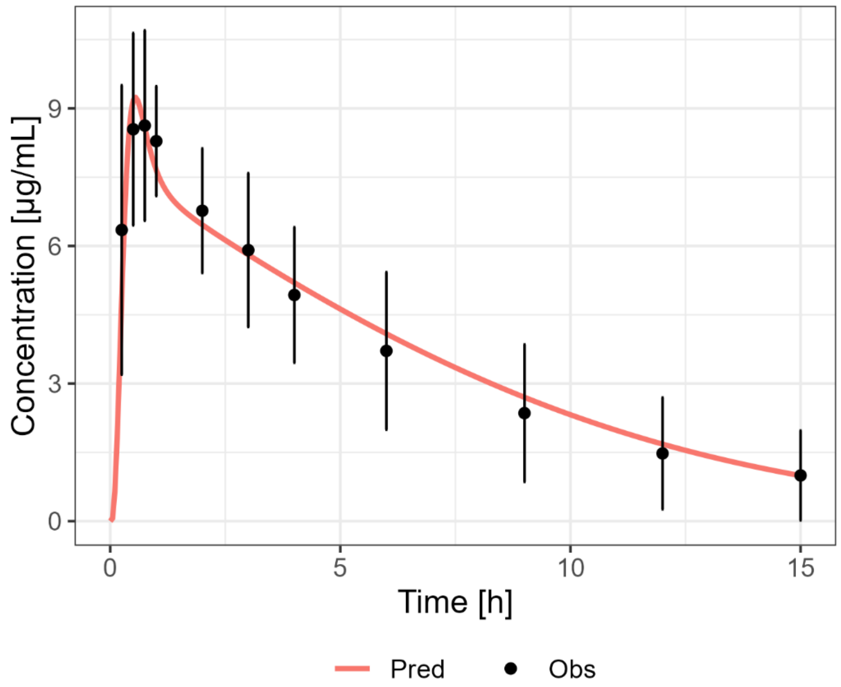

Figure S5. Predicted (Pred) versus observed (Obs) concentration-time profile after administration of 5 mg/kg PO SD [9]

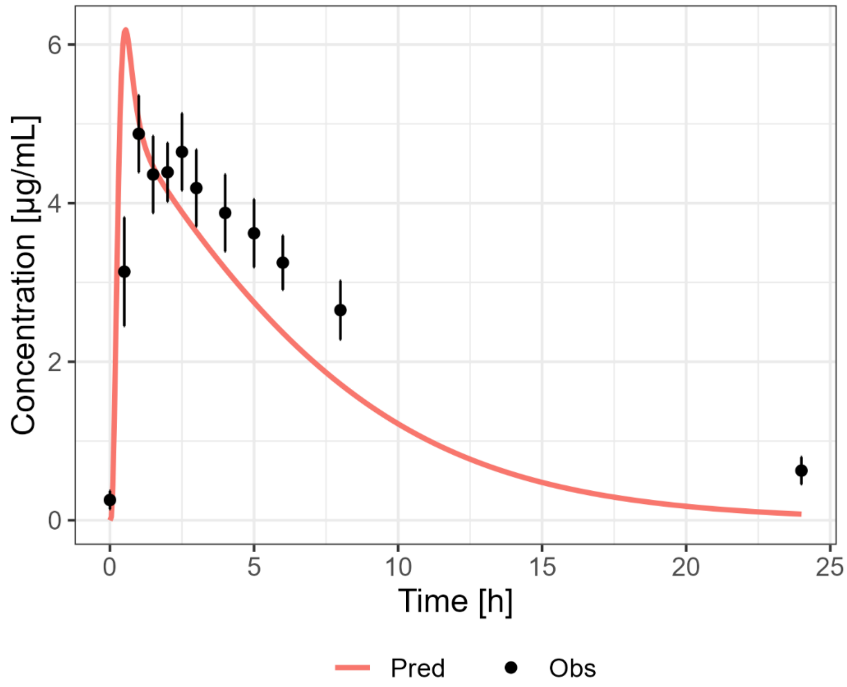

Figure S6 Predicted (Pred) versus observed (Obs) concentration-time profile after administration of 250 mg PO [10]

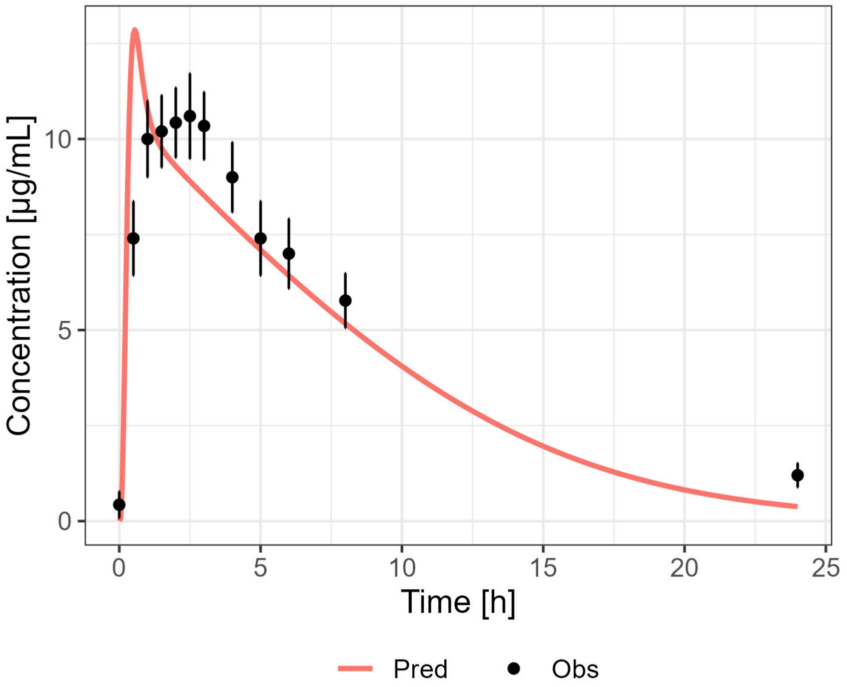

Figure S7 Predicted (Pred) versus observed (Obs) concentration-time profile after administration of 500 mg PO [10]

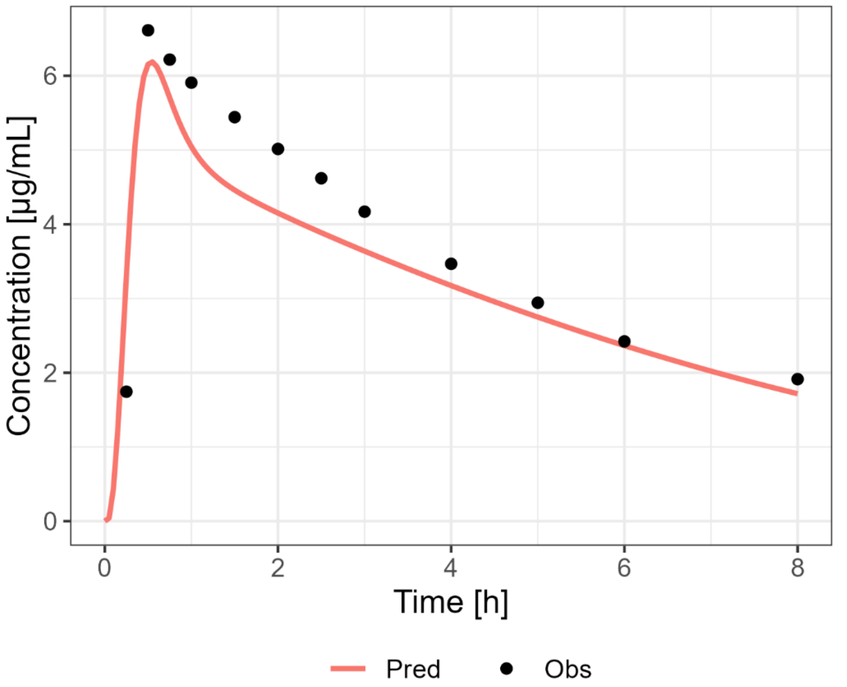

Figure S8 Predicted (Pred) versus observed (Obs) concentration-time profile after administration of 250 mg PO [11]

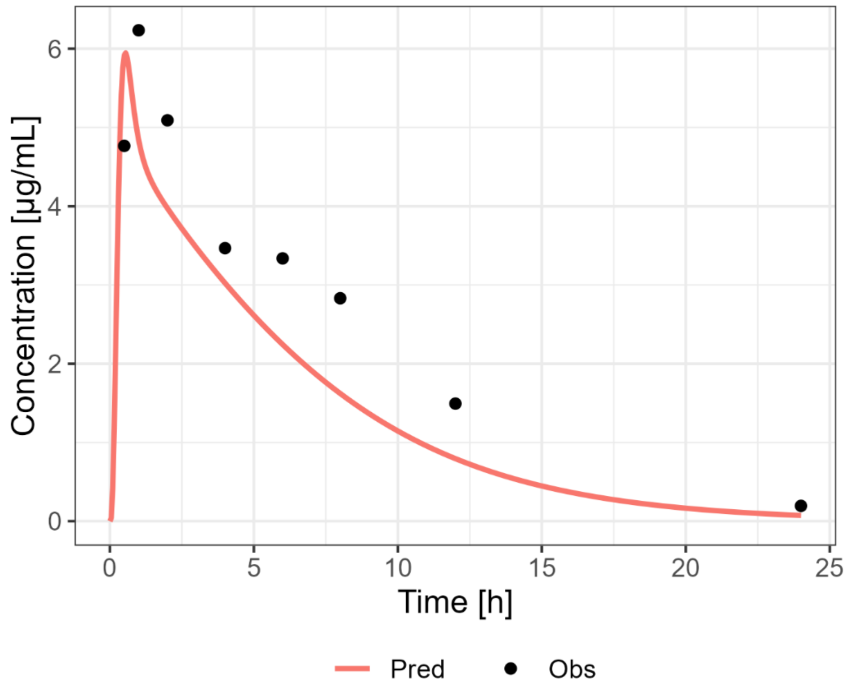

Figure S9 Predicted (Pred) versus observed (Obs) concentration-time profile after administration of 3 mg/kg PO grapefruit multiple exposure [12]

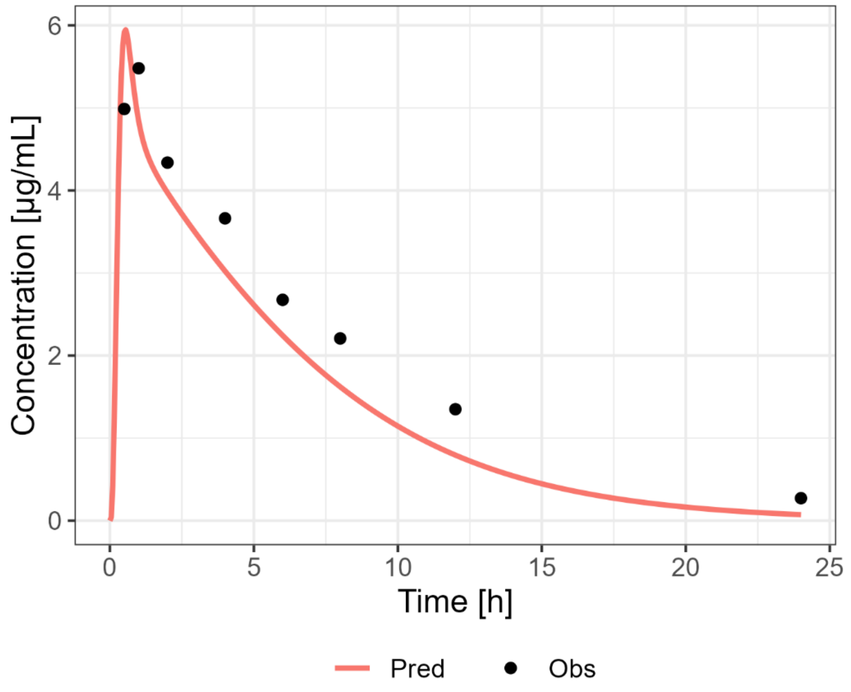

Figure S10 Predicted (Pred) versus observed (Obs) concentration-time profile after administration of 3 mg/kg PO grapefruit [12]

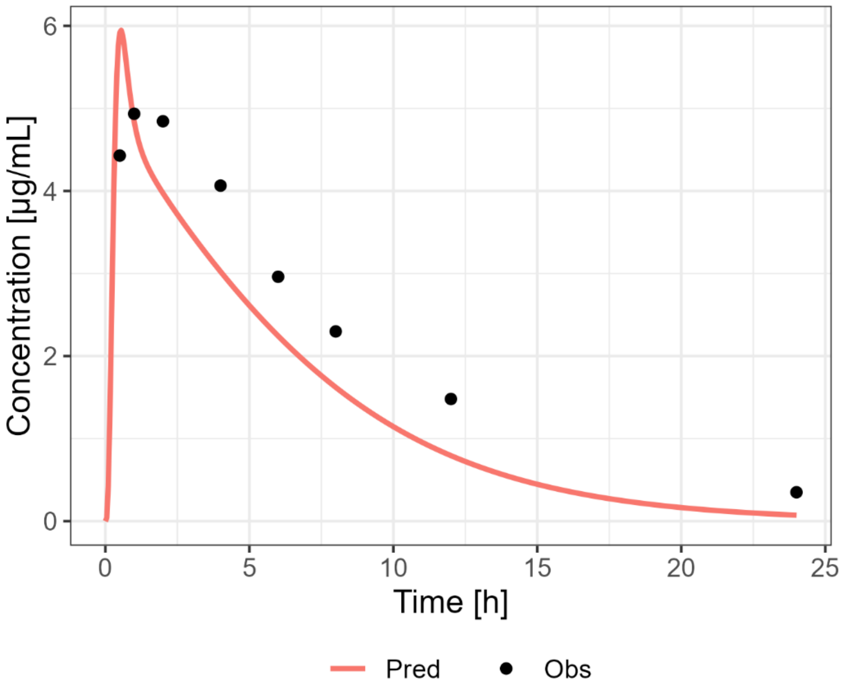

Figure S11 Predicted (Pred) versus observed (Obs) concentration-time profile after administration of 3 mg/kg PO water [12]

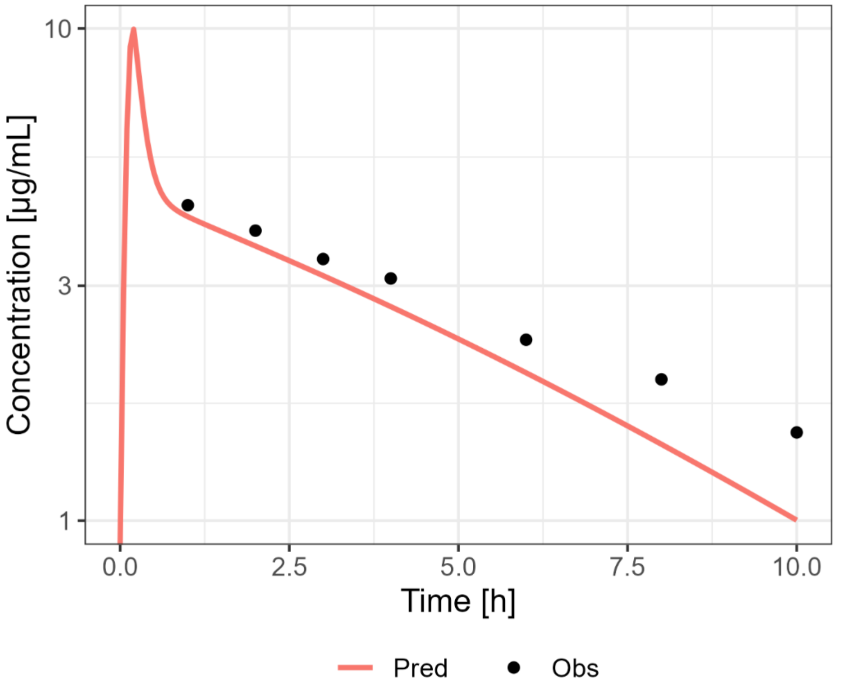

Figure S12 Predicted (Pred) versus observed (Obs) concentration-time profile after administration of 3 mg/kg IV [13]

4.3.2 Lactation PBPK model

A sample size of 1000 individuals, three months postpartum, was used in each simulation of the virtual lactation population.

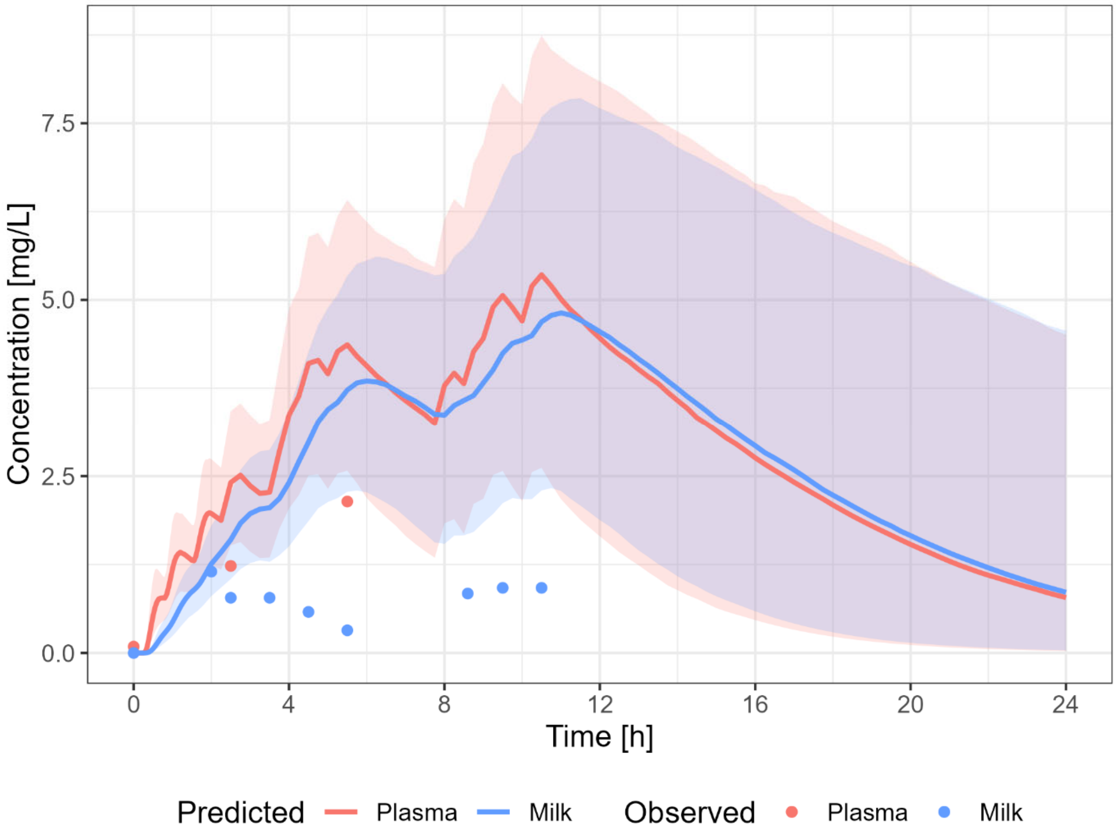

Figure S13 Pedicted (Pred) versus observed (Obs) concentration-time profile after administration of 33-35 mg PO [14]

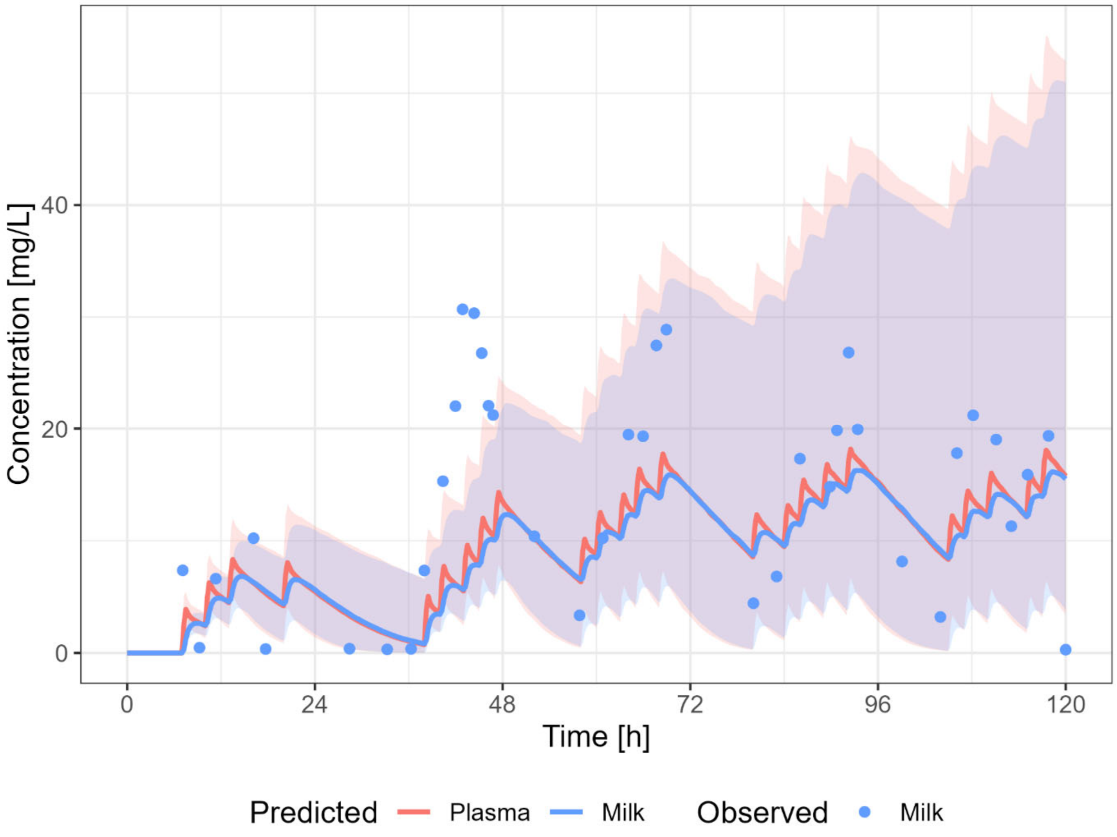

Figure S14 Pedicted (Pred) versus observed (Obs) concentration-time profile after administration of 150 mg PO [18]

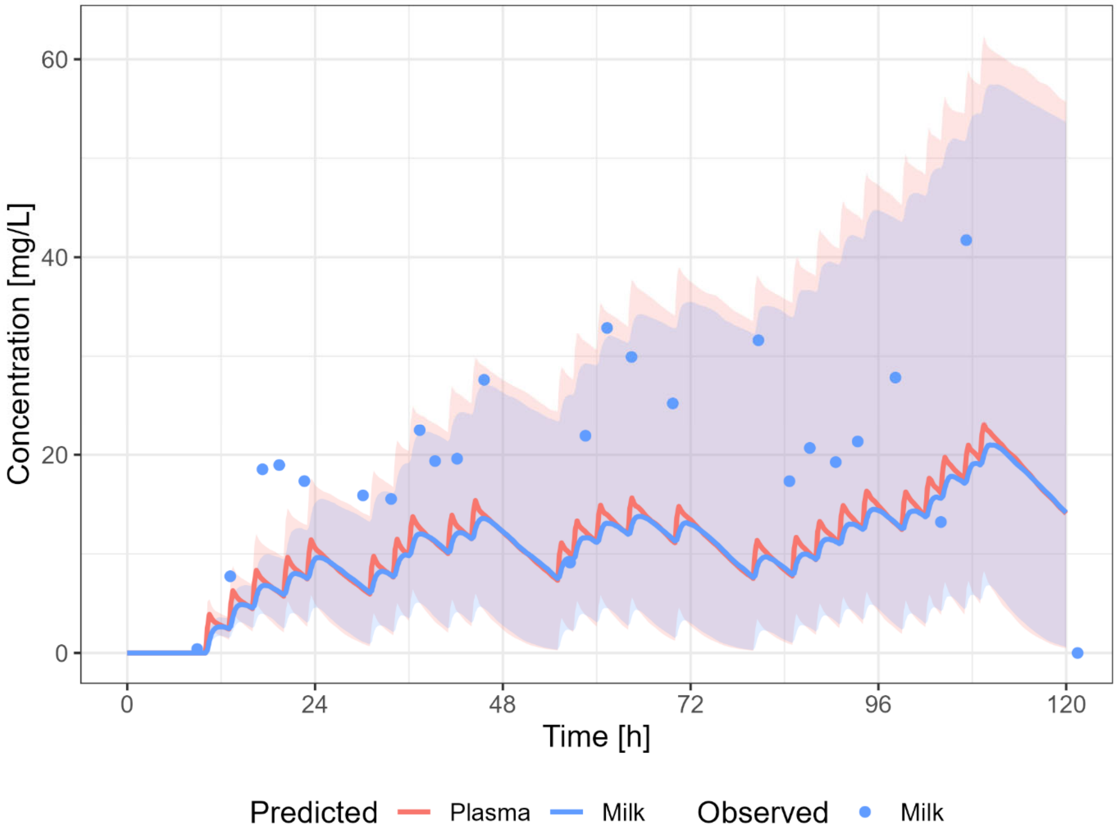

Figure S15 Pedicted (Pred) versus observed (Obs) concentration-time profile after administration of 150 mg PO [18]

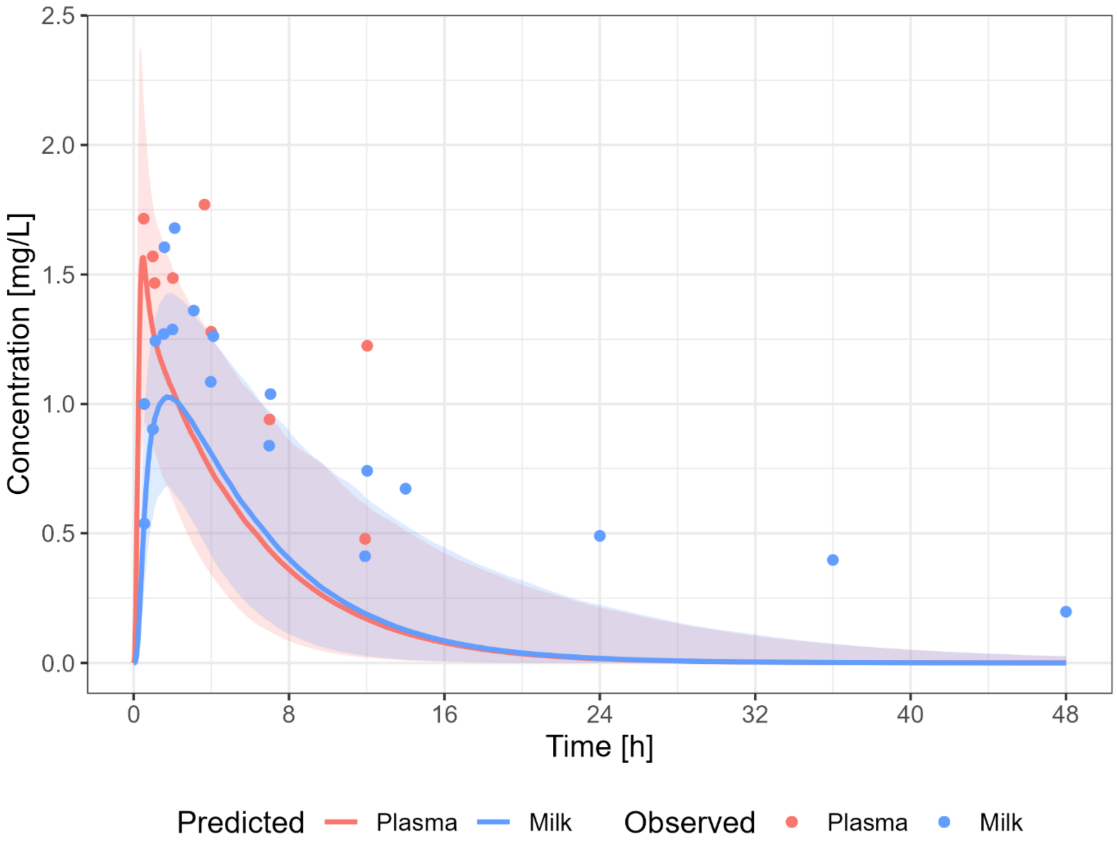

Figure S16 Pedicted (Pred) versus observed (Obs) concentration-time profile after administration of 64 mg PO SD [16]

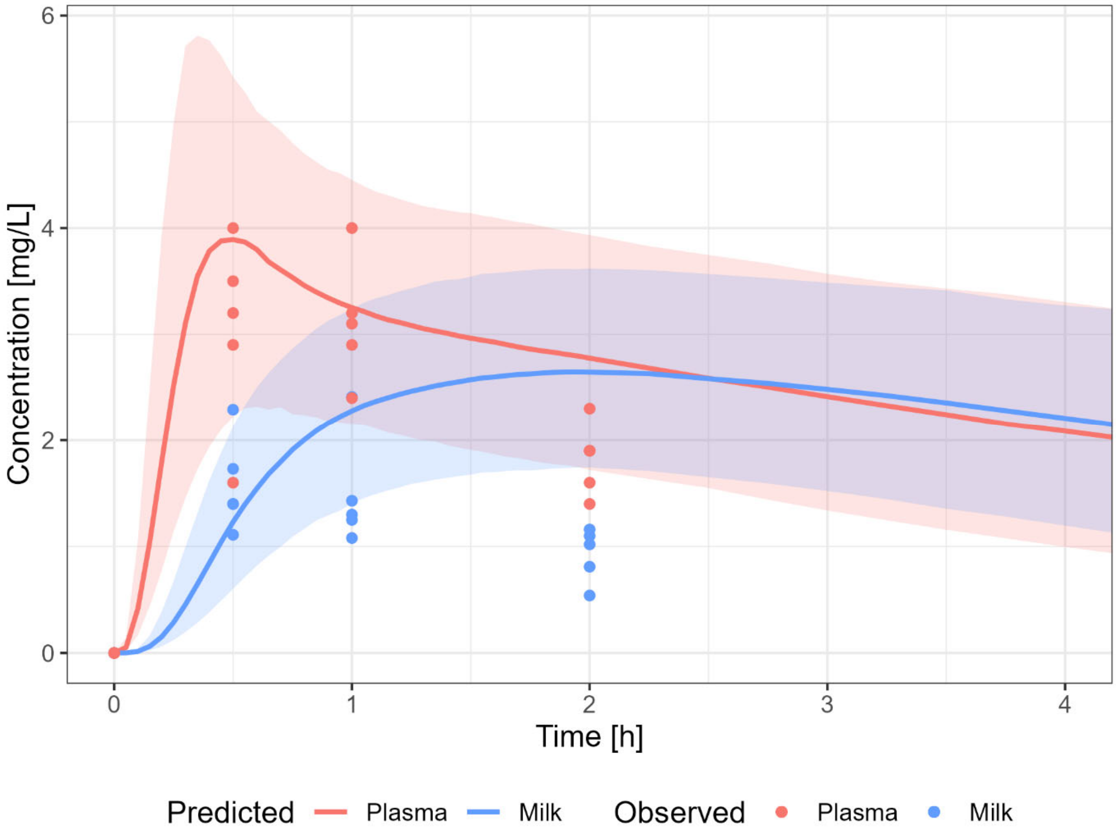

Figure S17 Pedicted (Pred) versus observed (Obs) concentration-time profile after administration of 150 mg PO SD [20]

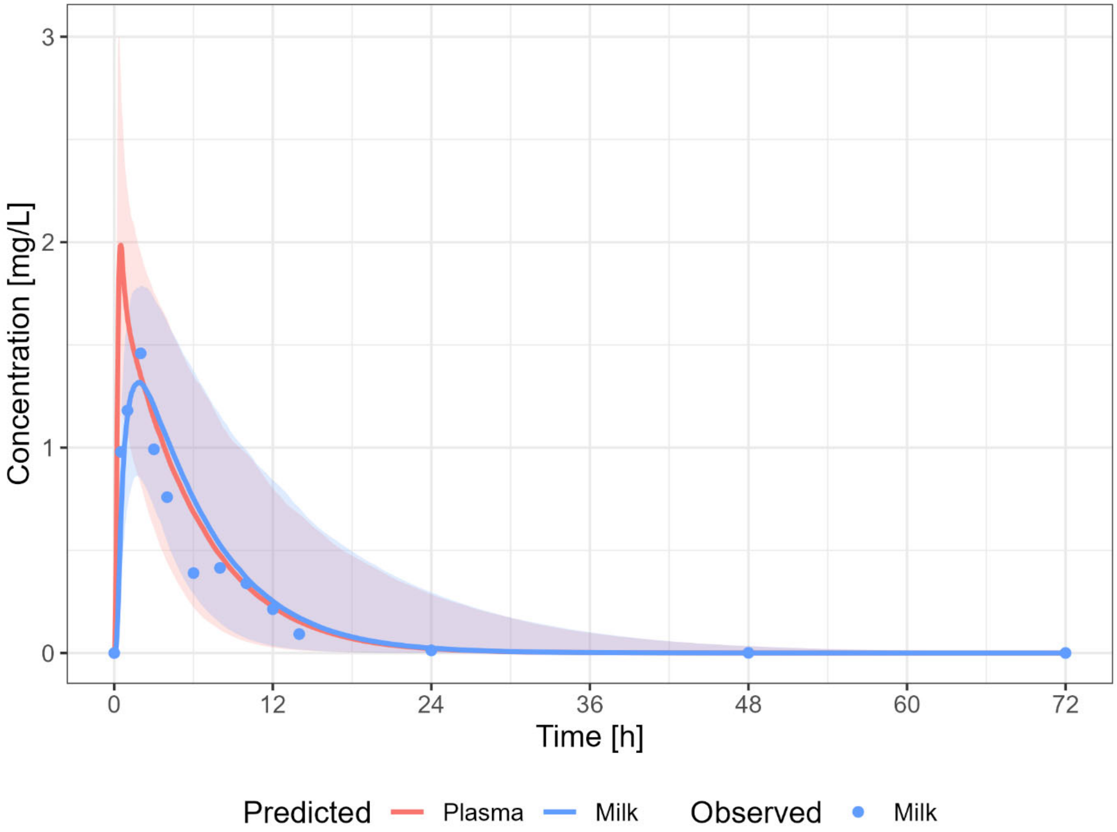

Figure S18 Pedicted (Pred) versus observed (Obs) concentration-time profile after administration of 80 mg PO SD [15]

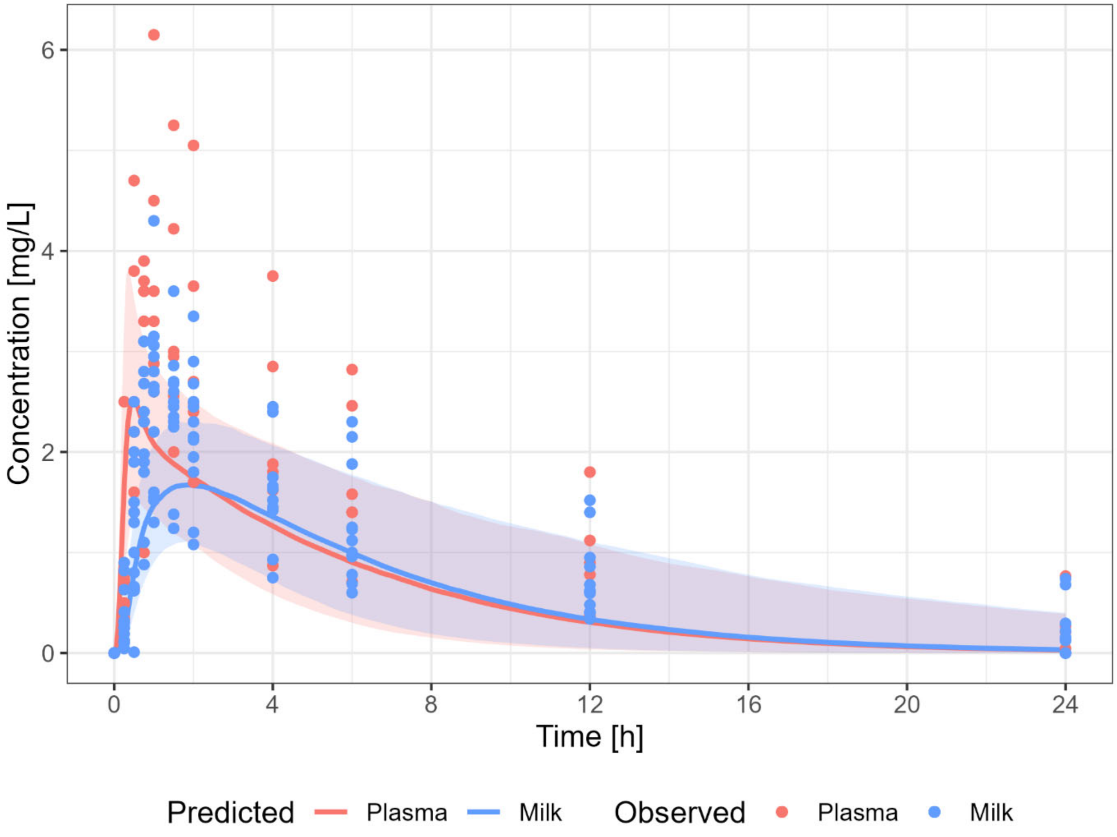

Figure S19 Pedicted (Pred) versus observed (Obs) concentration-time profile after administration of 100 mg PO SD [19]

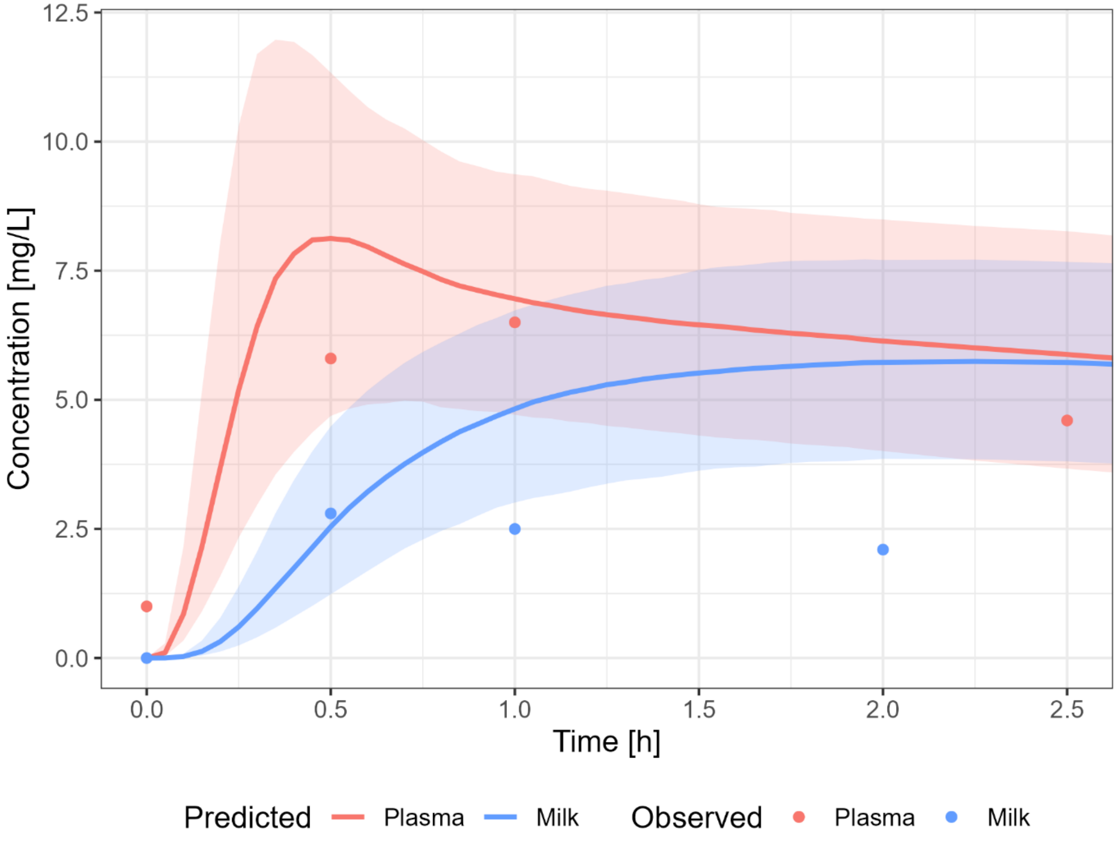

Figure S20 Pedicted (Pred) versus observed (Obs) concentration-time profile after administration of 300 mg PO SD [20]

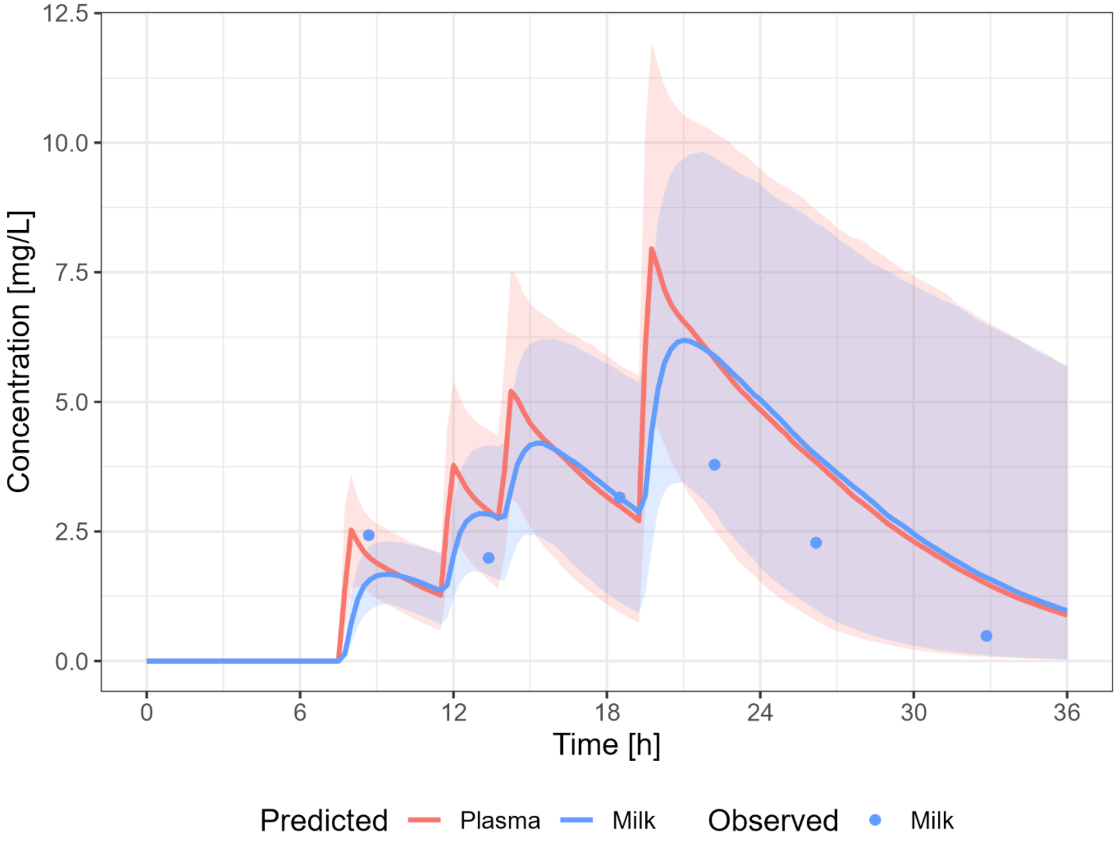

Figure S21 Pedicted (Pred) versus observed (Obs) concentration-time profile after administration of 100 mg PO MD subject 14 [17]

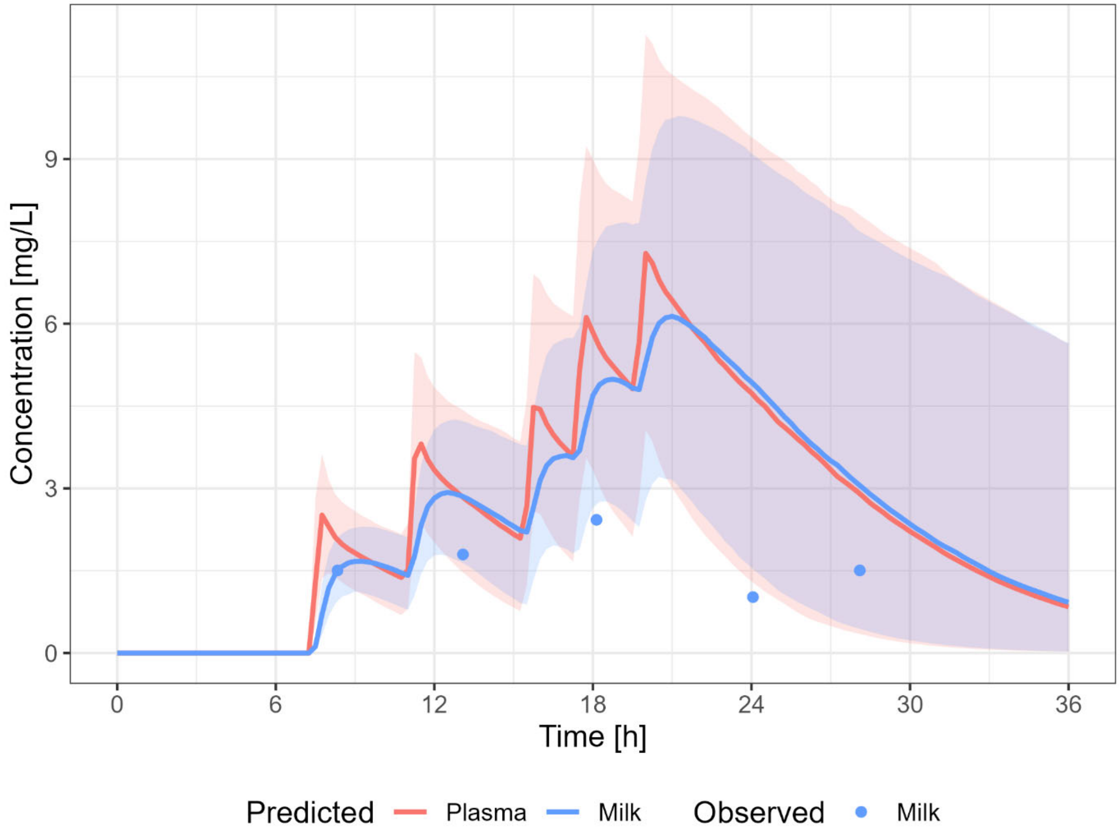

Figure S22 Pedicted (Pred) versus observed (Obs) concentration-time profile after administration of 100 mg PO MD subject 12 [17]

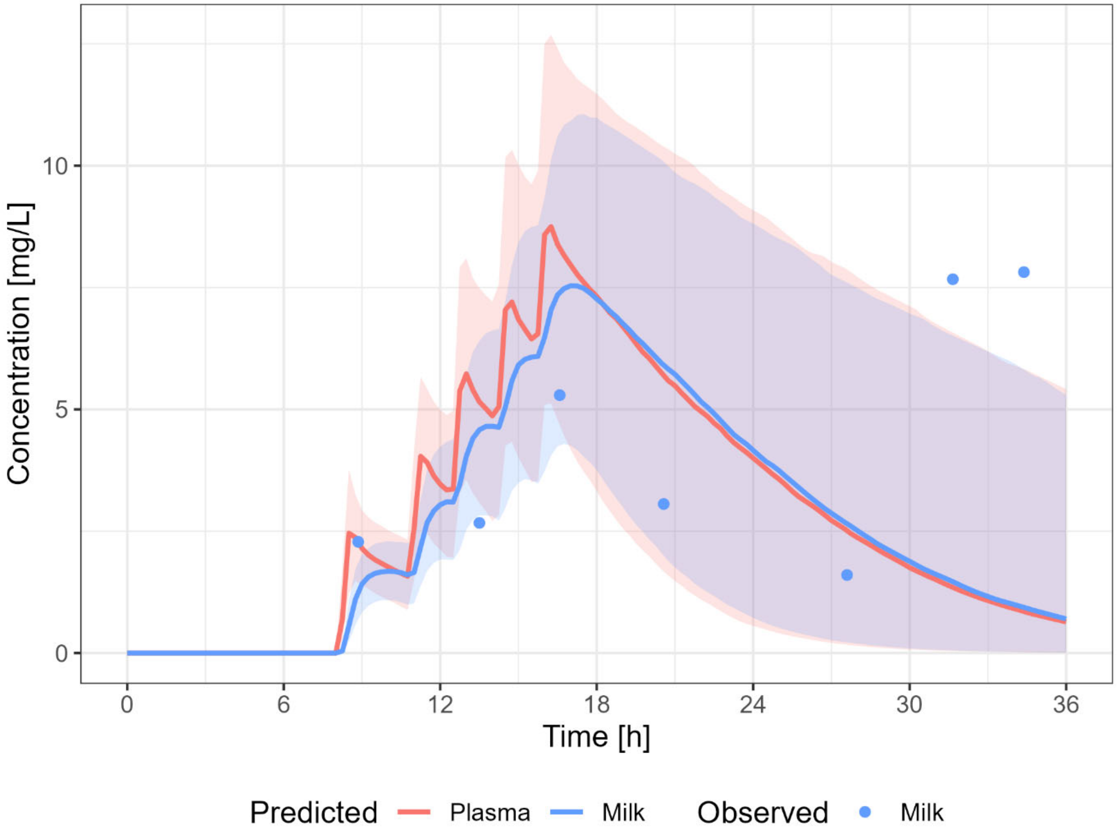

Figure S23 Pedicted (Pred) versus observed (Obs) concentration-time profile after administration of 100 mg PO MD subject 13 [17]

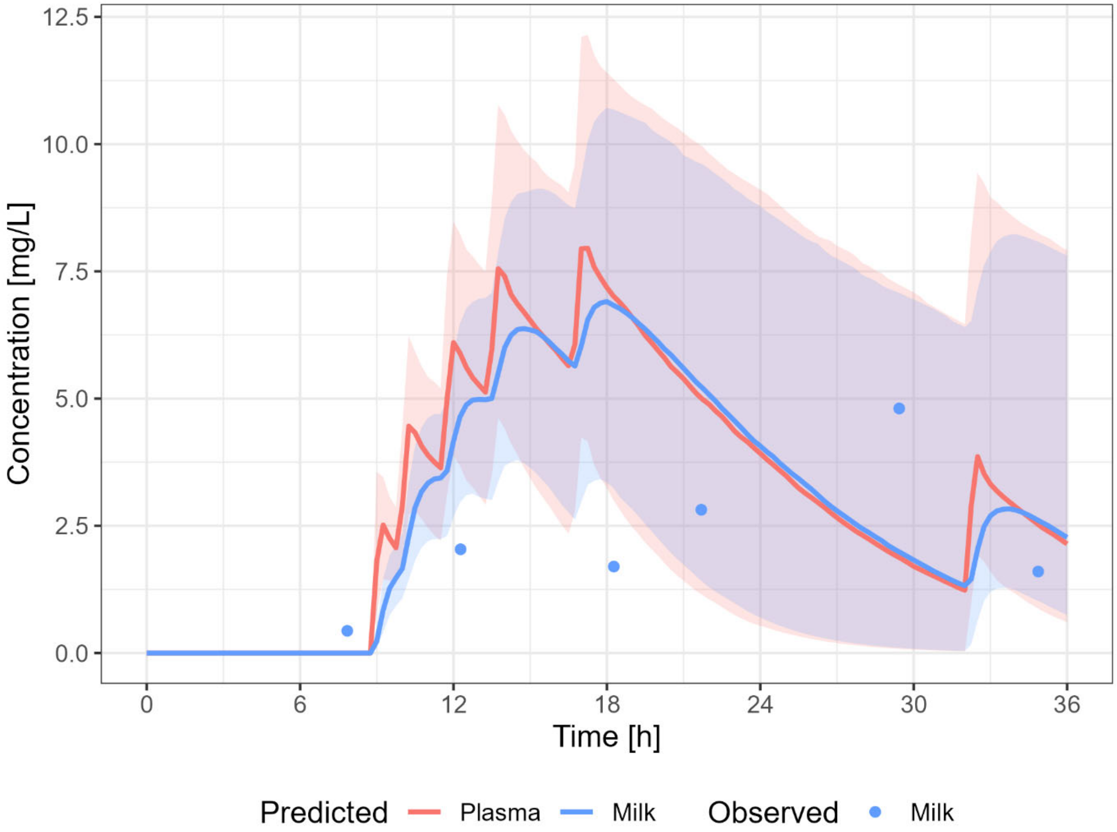

Figure S24 Pedicted (Pred) versus observed (Obs) concentration-time profile after administration of 100 mg PO MD subject 15 [17]

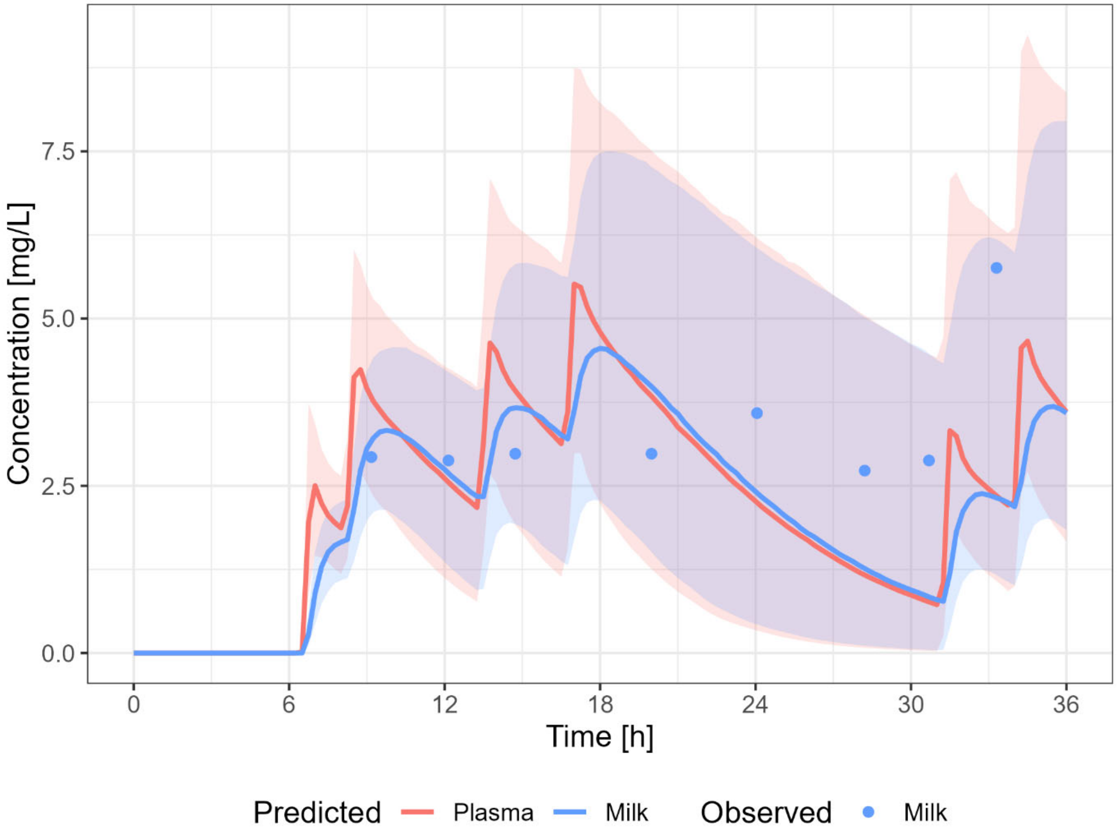

Figure S25 Pedicted (Pred) versus observed (Obs) concentration-time profile after administration of 100 mg PO MD subject 16 [17]

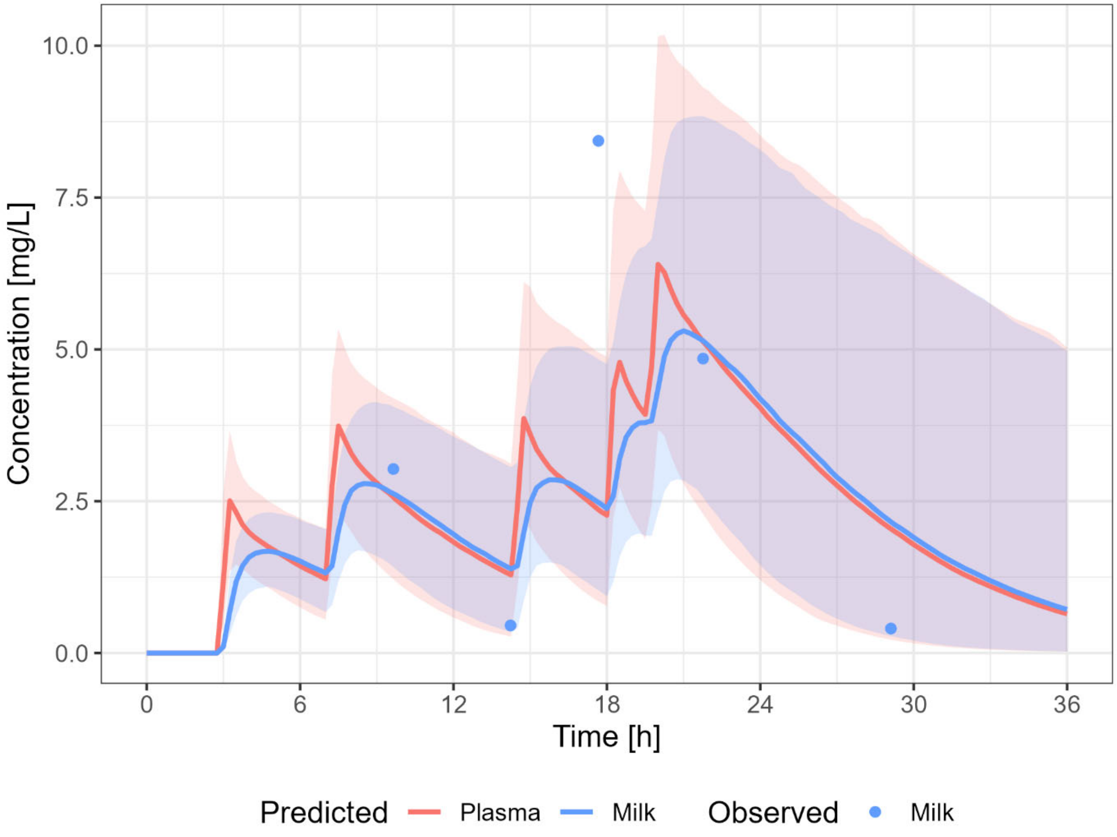

Figure S26 Pedicted (Pred) versus observed (Obs) concentration-time profile after administration of 100 mg PO MD subject 17 [17]

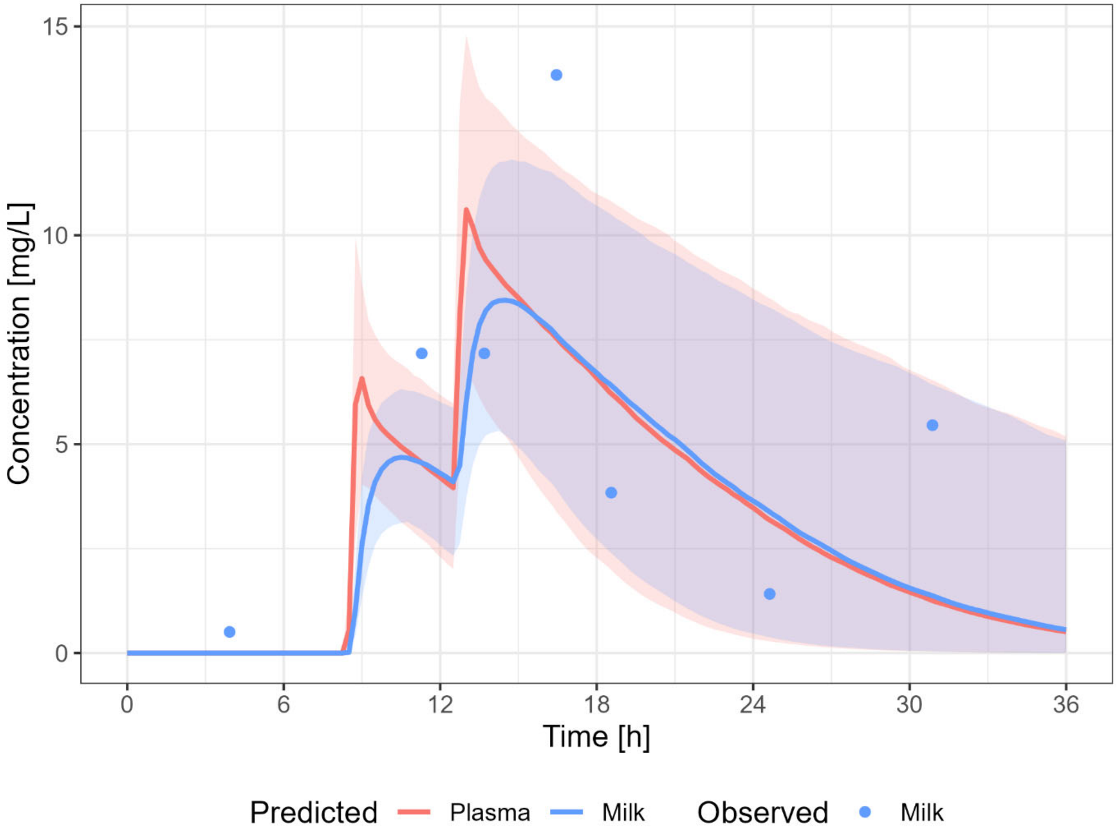

Figure S27 Pedicted (Pred) versus observed (Obs) concentration-time profile after administration of 100 mg PO MD subject 18 [17]

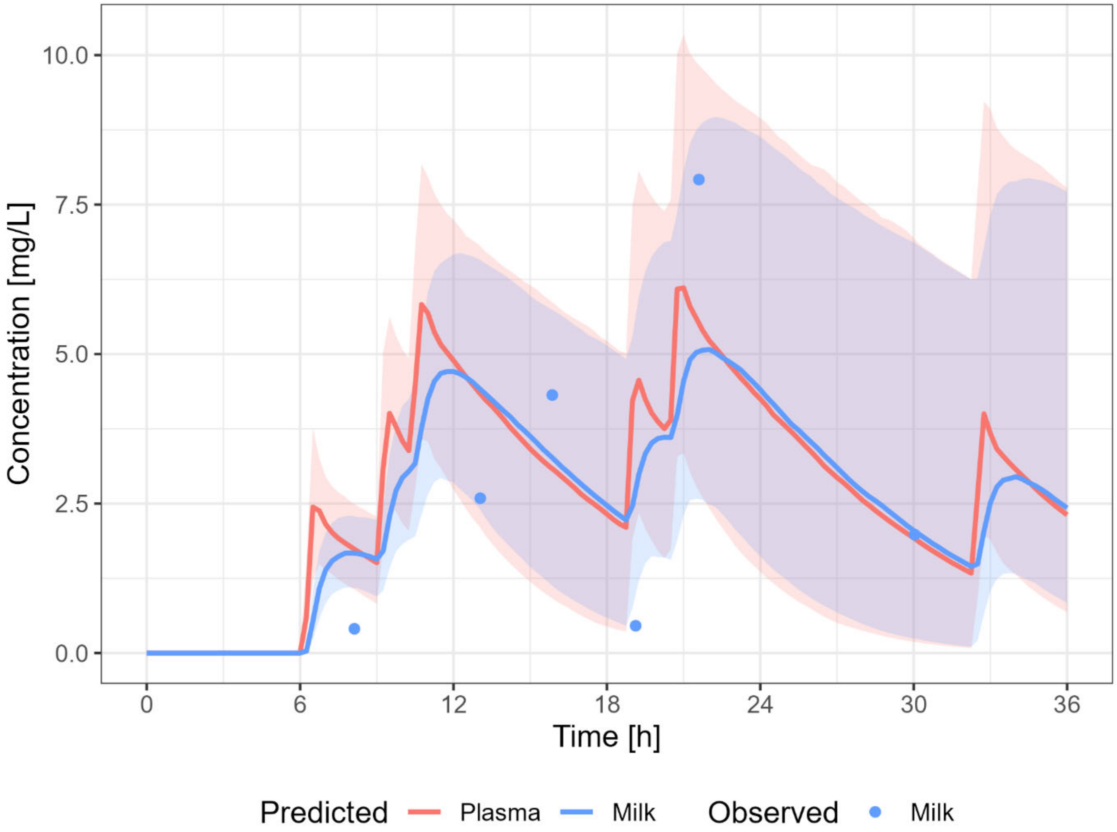

Figure S28 Pedicted (Pred) versus observed (Obs) concentration-time profile after administration of 100 mg PO MD subject 19 [17]

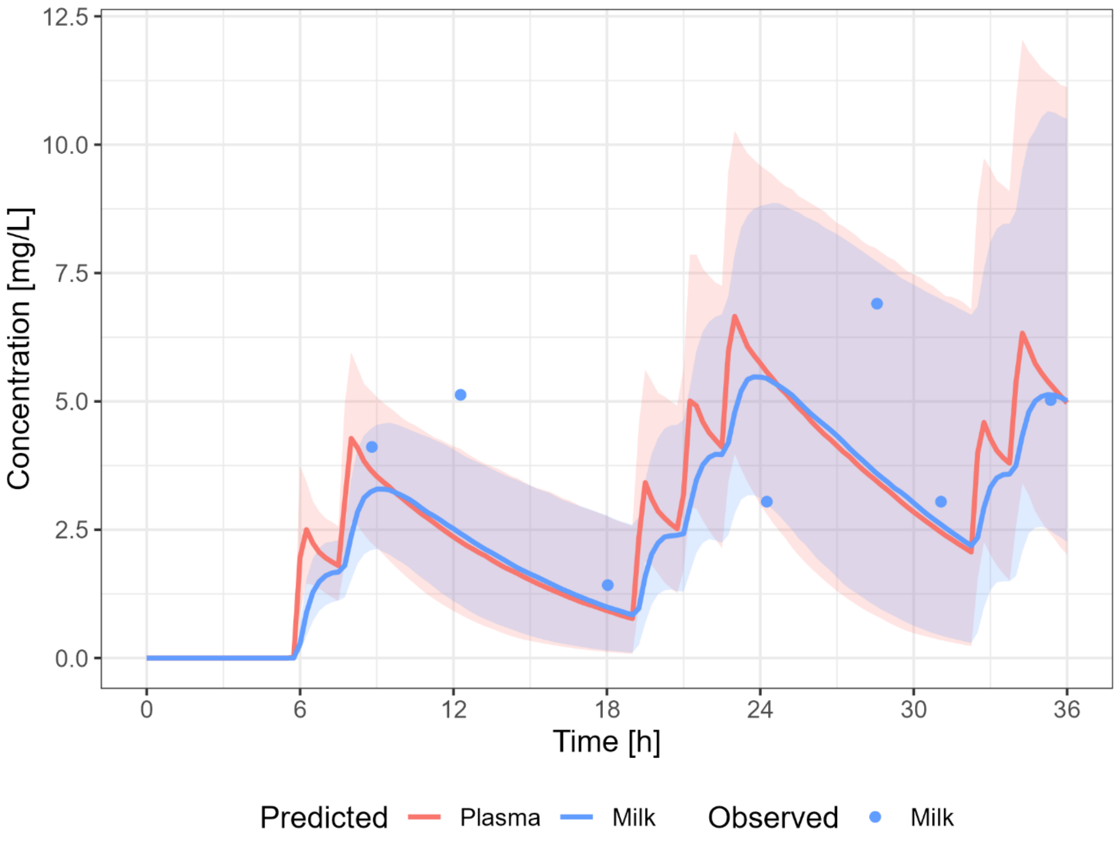

Figure S29 Pedicted (Pred) versus observed (Obs) concentration-time profile after administration of 100 mg PO MD subject 20 [17]

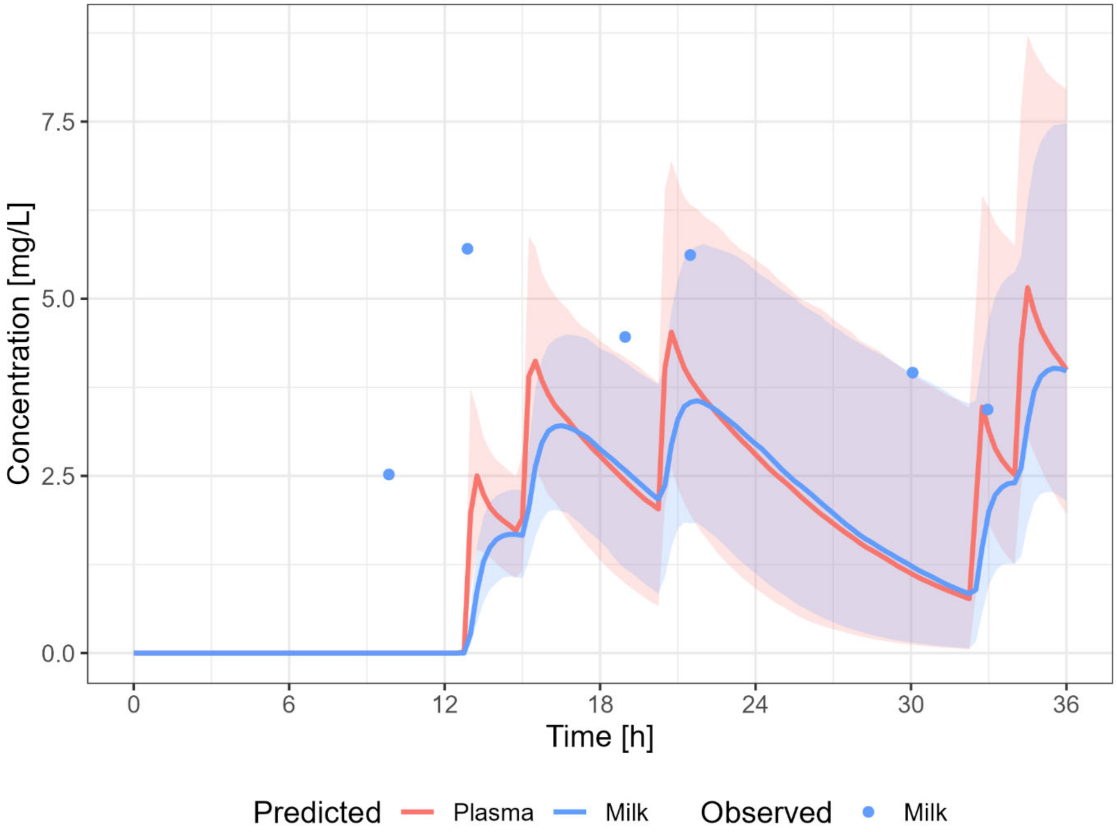

Figure S30 Pedicted (Pred) versus observed (Obs) concentration-time profile after administration of 100 mg PO MD subject 21 [17]

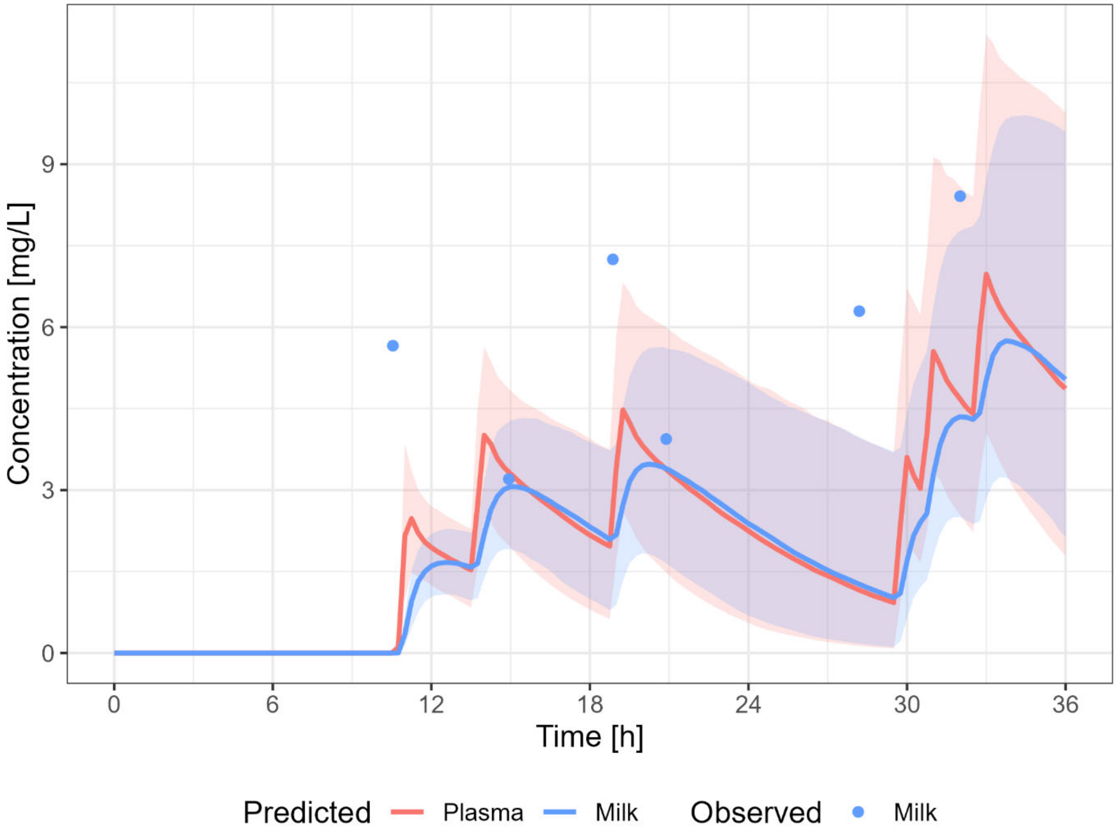

Figure S31 Pedicted (Pred) versus observed (Obs) concentration-time profile after administration of 100 mg PO MD subject 22 [17]

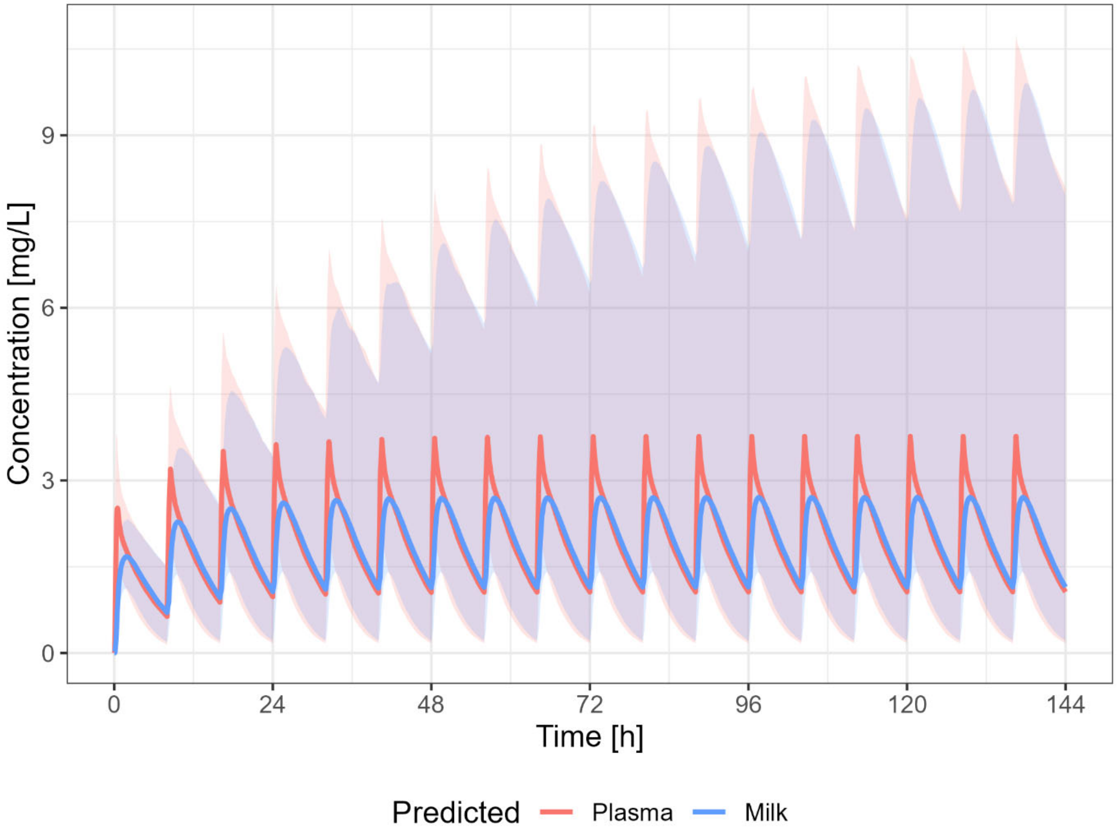

Figure S32 Pedicted (Pred) versus observed (Obs) concentration-time profile after administration of 100 mg PO MD

A dosing regimen of PO 100 mg, three times a day was used to calculate the milk transfer of caffeine.

Dosing interval: 8 h	Plasma	Milk
Cmax (mg/L)	3.77	2.71
AUC (mg*h/L)	16.73	15.88
Cave (mg/L)	2.09	1.99

M/P ratio = 0.95

4.4 Estimated infant dosage

A dosing regimen of 100 mg, every 8 h was assumed to calculate the infant dosage. The daily infant dosage and relative infant dose (RID) for 3 months old infants were calculated using a milk intake of 150 mL/kg/day. The daily infant dosage was 0.30 mg/kg/day (RID: 5.98 %) or 0.41 mg/kg/day (RID: 8.17 %) based on the average steady-state concentration and maximum concentration in human milk, respectively.

5. Discussion

First, the reference PBPK model was developed and evaluated. Evaluation of the predictive performance showed that the reference PBPK model for caffeine was able to capture the pharmacokinetic behavior of the medicines in healthy volunteers and/or patients.
Next, the PBPK model was extended to a lactation PBPK model. The PBPK model results in an acceptable prediction of the human milk concentrations, with most datapoints within the

5-95th percentile of the population prediction. Importantly, some of the studies were performed at the home of the participants, and relied on the subjects to reports the time and amount of each dose. In some participants, there is already caffeine measured in human milk before the first dose was reported. Therefore, it cannot be excluded that the participants did not report the time of each dose reliably and/or that they received additional caffeine via their diet.

The predicted M/P ratio (0.95) was within the observed range of M/P ratios (0.52 - 1.16).

The calculated infant dosage of caffeine via breastfeeding was low (less than 10 % of the maternal daily dosage).

6. Conclusions

The herein presented PBPK model adequately describes the PK of caffeine in adults including breastfeeding women. In particular, it applies quantitative metabolism by cytochrome P450 1A2 and renal clearance. The PBPK model was able to predict the human milk concentrations of caffeine (M/P ratio: 0.95). The daily infant dosage was 0.30 mg/kg/day (RID: 5.98 %) or 0.41 mg/kg/day (RID: 8.17 %) based on the average steady-state concentration and maximum concentration in human milk, respectively.

7. List of Appendix and Supplementary Materials

Supplementary material 1 – ObsDataPK_OSP_reference_caffeine
Supplementary material 2 – ObsDataPK_OSP_lactation_caffeine
Supplementary material 3 – Caffeine.pksim5

8. References

1. Drugbank - Caffeine Available online: https://go.drugbank.com/drugs/DB00201.
2. Dallmann, A.; Himstedt, A.; Solodenko, J.; Ince, I.; Hempel, G.; Eissing, T. Integration of Physiological Changes during the Postpartum Period into a PBPK Framework and Prediction of Amoxicillin Disposition before and Shortly after Delivery. *J. Pharmacokinet. Pharmacodyn.* **2020**, *47*, 341–359, doi:10.1007/s10928-020-09706-z.
3. Dallmann, A.; Solodenko, J.; Ince, I.; Eissing, T. Applied Concepts in PBPK Modeling: How to Extend an Open Systems Pharmacology Model to the Special Population of Pregnant Women. *CPT pharmacometrics Syst. Pharmacol.* **2018**, *7*, 419–431, doi:10.1002/PSP4.12300.
4. Job, K.M.; Dallmann, A.; Parry, S.; Saade, G.; Haas, D.M.; Hughes, B.; Berens, P.; Chen, J.; Fu, C.; Humphrey, K.; et al. Development of a Generic Physiologically-Based Pharmacokinetic Model for Lactation and Prediction of Maternal and Infant Exposure to Ondansetron via Breast Milk. *Clin. Pharmacol. Ther.* **2022**, *111*, 1111–1120, doi:10.1002/cpt.2530.
5. Koshimichi, H.; Ito, K.; Hisaka, A.; Honma, M.; Suzuki, H. Analysis and Prediction of Drug Transfer into Human Milk Taking into Consideration Secretion and Reuptake Clearances across the Mammary Epithelia. *Drug Metab. Dispos.* **2011**, *39*, 2370–2380, doi:10.1124/dmd.111.040972.
6. Lelo, A.; Birkett, D.; Robson, R.; Miners, J. Comparative Pharmacokinetics of Caffeine and Its Primary Demethylated Metabolites Paraxanthine, Theobromine and Theophylline in Man. *Br. J. Clin. Pharmacol.* **1986**, *22*, 177–182, doi:10.1111/j.1365-2125.1986.tb05246.x.
7. Birkett, D.J.; Miners, J. 0 Caffeine Renal Clearance and Urine Caffeine Concentrations during Steady State Dosing. Implications for Monitoring Caffeine Intake during Sports Events. *J. clin. Pharmac* **1991**, *31*, 405–408, doi:10.1111/j.1365-2125.1991.tb05553.x.
8. Atkinson, U.C.; Begg, E.J.E.J.; Atkinson, H.C.; Begg, E.J.E.J. Prediction of Drug Distribution into Human Milk from Physicochemical Characteristics. *Clin. Pharmacokinet.* **1990**, *18*, 151–167, doi:10.2165/00003088-199018020-00005.
9. Blanchard, J.; Sawers, S.J.A. Comparative Pharmacokinetics of Caffeine in Young and Elderly Men. *J. Pharmacokinet. Biopharm.* **1983**, *11*, 109–126, doi:10.1007/BF01061844.
10. Cysneiros, R.M.; Farkas, D.; Harmatz, J.S.; Von Moltke, L.L.; Greenblatt, D.J. Pharmacokinetic and Pharmacodynamic Interactions between Zolpidem and Caffeine. *Clin. Pharmacol. Ther.* **2007**, *82*, 54–62, doi:10.1038/sj.clpt.6100211.
11. Kaplan, G.B.; Greenblatt, D.J.; Ehrenberg, B.L.; Goddard, J.E.; Cotreau, M.M.; Harmatz, J.S.; Shader, R.I. Dose-Dependent Pharmacokinetics and Psychomotor Effects of Caffeine in Humans. *J. Clin. Pharmacol.* **1997**, *37*, 693–703, doi:10.1002/j.1552-4604.1997.tb04356.x.
12. Maish, W.A.; Hampton, E.M.; Whitsett, T.L.; Shepard, J.D.; Lovallo, W.R. Influence of Grapefruit Juice on Caffeine Pharmacokinetics and Pharmacodynamics.

Pharmacotherapy 16, 1046–1052, doi:https://doi.org/10.1002/j.1875-9114.1996.tb03031.x.

13. Wahlländer, A.; Paumgartner, G. Effect of Ketoconazole and Terbinafine on the Pharmacokinetics of Caffeine in Healthy Volunteers. *Eur. J. Clin. Pharmacol.* **1989**, *37*, 279–283, doi:10.1007/BF00679784.
14. Bailey, D.N.; Weibert, R.T.; Naylor, A.J. A Study of Salicylate and Caffeine Excretion in the Breast Milk of Two Nursing Mothers. *J. Anal. Toxicol.* **1982**, *6*, 64–68, doi:10.1093/jat/6.2.64.
15. Calvaresi, V.; Escuder, D.; Minutillo, A.; Bastons-Compta, A.; García-Algar, O.; Alonso, C.R.P.; Pacifici, R.; Pichini, S. Transfer of Nicotine, Cotinine and Caffeine into Breast Milk in a Smoker Mother Consuming Caffeinated Drinks. *J. Anal. Toxicol.* **2016**, *40*, 473–477, doi:10.1093/jat/bkw034.
16. Findlay, J.W.A.; DeAngelis, R.L.; Kearney, M.F.; Welch, R.M.; Findlay, J.M. Analgesie Drugs in Breast Milk and Plasma*. *Clin. Pharmacol. Ther.* **1981**, *29*, 625–633, doi:10.1038/clpt.1981.87.
17. Ryu, J.E. Effect of Maternal Caffeine Consumption on Heart Rate and Sleep Time of Breast-Fed Infants. *Dev. Pharmacol. Ther.* **1985**, *8*, 355–363, doi:10.1159/000457060.
18. Ryu, J.E. Caffeine in Human Milk and in Serum of Breast-Fed Infants. *Dev. Pharmacol. Ther.* **1985**, *8*, 329–337, doi:10.1159/000457057.
19. Stavchansky, S.; Delgado, M.; Joshi, A.; Combs, A.; Sagraves, R. Pharmacokinetics of Caffeine in Breast Milk and Plasma after Single Oral Administration of Caffeine to Lactating Mothers. *Biopharm. Drug Dispos.* **1988**, *9*, 285–299, doi:10.1002/bod.2510090307.
20. Tyrala, E.E.; Dodson, W.E. Caffeine Secretion into Breast Milk. *Arch. Dis. Child.* **1979**, *54*, 787–800, doi:10.1136/adc.54.10.787.
