# Supplementary material for: Generic Workflow to Predict Medicine Concentrations in Human Milk Using Physiologically-Based Pharmacokinetic (PBPK) Modelling—A Contribution from the ConcePTION Project
Source: Pharmaceutics. 2023 May 11;15(5):1469. doi: 10.3390/pharmaceutics15051469 (PMC10223637; doi:10.3390/pharmaceutics15051469)
Supplement: Supplementary file 1 [file pharmaceutics-15-01469-s001.zip › File S3. Evaluation_report_cetirizine.pdf]

Building and evaluation of a Physiologically-Based PharmacoKinetic (PBPK) model for **cetirizine** in adults and lactating women

Disclaimer: The research project leading to these results was conducted as part of the ConcePTION consortium. This report only reflects the personal views of the stated authors. The results of this report are only intended for research purpose, and are not intended to be used in clinical practice.

Glossary

AUC	Area Under the Curve
C_{ave}	Average concentration
CL_{re}	Reuptake clearance (i.e. from milk to blood)
CL_{sec}	Secretion clearance (i.e. from blood to milk)
C_{max}	Maximum (~peak) concentration
DID	Daily Infant Dosage (expressed for instance in mg/kg/day)
f_u	Fraction unbound in plasma
GFR	Glomerular Filtration Rate
HBD	Hydrogen Bond Donors
IV	Intravenous administration
$LogD_{7.2}$	Logarithm of the partition coefficient between an octanol phase and an aqueous (buffer) phase at pH 7.2
$LogD_{7.4}$	Logarithm of the partition coefficient between an octanol phase and an aqueous (buffer) phase at pH 7.4
LogP	Logarithm of the partition coefficient between an octanol phase and (unbuffered) water as aqueous phase. This is the default parameter to express lipophilicity of a substance.
MD	Multiple doses
M/P ratio	Milk-to-Plasma ratio
MW	Molecular Weight (Da)
PBPK	Physiologically-Based PharmacoKinetic [*modeling*]
pKa	Logarithm of the acid dissociation constant
PO	Oral administration
PSA	Polar Surface Area
RID	Relative Infant Dose (%)
SD	Single dose

1. Table of Contents

1. Table of Contents 3

2. Introduction 4

3. Methods 5

3.1 Modelling strategy 5

3.1.1. Reference PBPK models 6

3.1.2. Lactation model 6

3.2 Data 6

3.2.1 *In vitro* / physicochemical data 6

3.2.2 Clinical data 9

3.3 Model Parameters and assumptions 10

3.3.1 Absorption 10

3.3.2 Distribution 10

3.3.3 Metabolism and excretion 11

3.3.4 Secretion to milk 11

3.3.5 Automated parameter optimization 11

3.4. Infant dosage calculation 12

4. Results 12

4.1 Final input parameters 12

4.2 Diagnostic plots 14

4.3 Concentration-time profiles 16

4.3.1 Model building 16

4.3.2 Model verification 17

4.3.3 Lactation PBPK model 21

4.4 Estimated Pediatric exposure 24

5. Discussion 25

6. Conclusion 25

7. List of Appendix and Supplementary Materials 26

8. References 26

2. Introduction

Cetirizine (Figure S1) is a second generation H1-antihistaminicum. It is a racemic mixture of levo- and dextrocetirizine. The recommended dose is 10 mg/day of cetirizine hydrochloride [1]. Absorption of cetirizine is rapid and estimated to be more than 70 %. Peak concentrations around 341 ng/mL are achieved within 1 h [1]. The apparent volume of distribution is 0.44 L/kg [drugbank]. Ceitirizine is highly bound to plasma proteins (93 %). Cetirizine is mainly eliminated unchanged into urine. The half-life is around 5h.

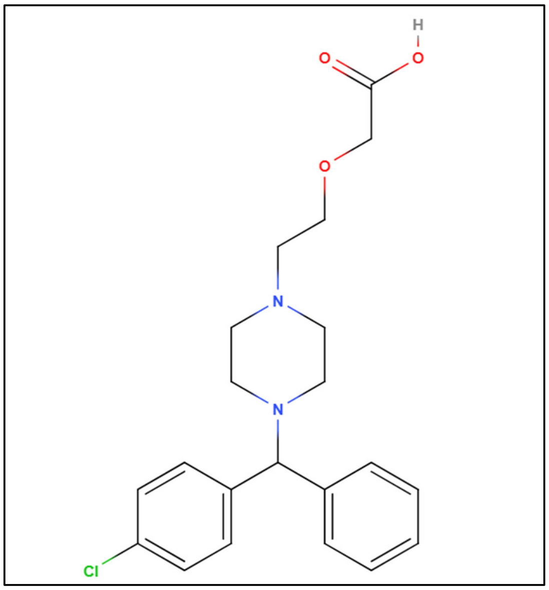

Figure S1 Chemical Structure of cetirizine

The scope of this report is to:

(a) specify the details and underlying assumptions associated with the building of physiologically-based pharmacokinetic (PBPK) models for cetirizine in adult healthy volunteers or patients, and in postpartum women during lactation.

(b) evaluate the predictive performance of these PBPK models. This is achieved by comparing model-predicted plasma or milk concentrations with corresponding clinical observations.

3. Methods

The software used for the development of PBPK models presented in this report is tabulated below:

Software	Version
PK-Sim®	v9.1
MoBi®	v9.1

3.1 Modelling strategy

In the present report, a reference PBPK model was first established for adults (patients as well as healthy volunteers), and subsequently verified against clinical pharmacokinetic data reported for cetirizine in the scientific literature.

Relevant information on the anthropometry (height, weight) was gathered from the respective clinical studies, if reported. Information on physiological parameters (e.g. blood flows, organ volumes, hematocrit) in adults is available in the PK-Sim® database.

In a second step, a lactation PBPK model was developed, based on the general workflow described by Dallmann *et al. 2018* [2–4].

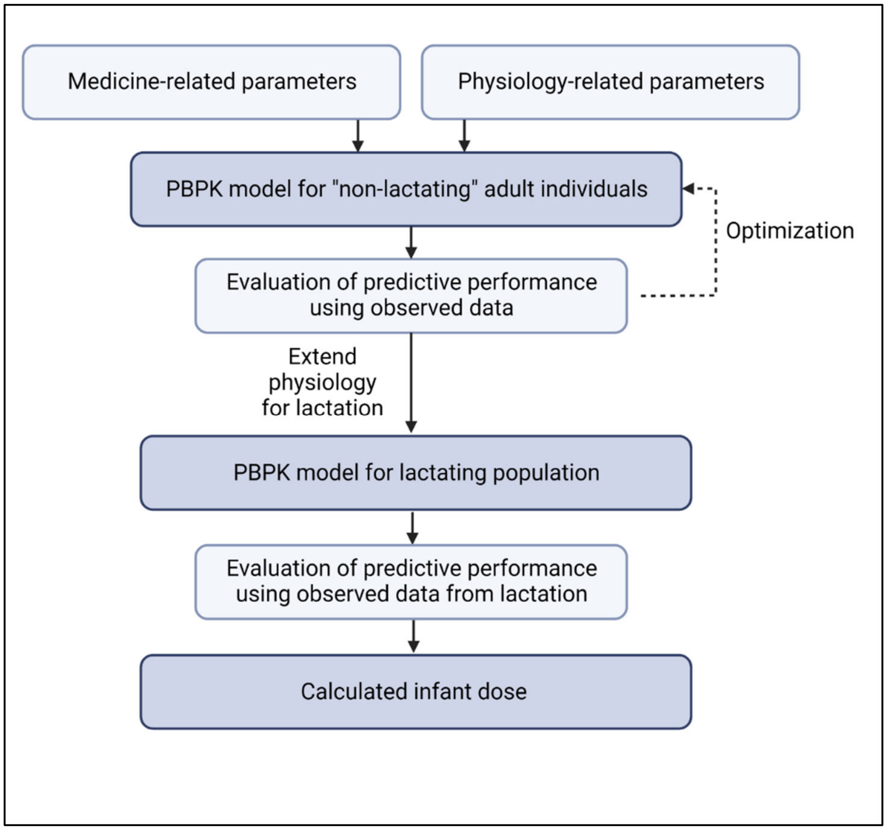

Figure S2 General workflow that was used in the present project to develop and evaluate the lactation PBPK model

Details about input data (physicochemical, *in vitro* and clinical data) can be found in section 3.2. Details about the structural models and their parameters can be found in section 3.3.

3.1.1. Reference PBPK models

The reference PBPK models were built based on studies with adult volunteers and/or adult patients, using the reported mean values for age, weight, height, and genetic background as described in each study protocol. When no information on these parameters could be found, a healthy male European individual, 30 years of age, with a body weight of 73 kg and a height of 176 cm was used.

The abundance (including population variability) of plasma proteins and enzymes/transporters that are integrated into PK-Sim are described in the publicly available 'PK-Sim Ontogeny Database Version 7.3' (PK-Sim Ontogeny Database Version 7.3).

Structural model selection was mainly guided by biological plausibility and by visual inspection of the predicted concentration time profiles in comparison with observed data. The generally applied acceptance criterium was less than 2-fold misprediction. Uninformed parameter values (see below) were estimated using the parameter identification module of PK-Sim®.

The predictive performance of the models was evaluated by simulating:

- Oral administration of 10 and 20 mg
- Single and multiple oral dose studies
- Fed and fasted state

For some parameters, parameter optimization was performed as described below to obtain improved concordance between predicted profiles and observed data.

3.1.2. Lactation model

After development of the reference model, the model was exported to MoBi® and a lactation PBPK model was constructed. To model the passage of cetirizine into human milk, i.e. across the blood/milk biological barrier, both the secretion (CL_{sec}) and reuptake clearance (CL_{re}) values were obtained using the empirical model developed by Koshimichi *et al. 2011* [5].

3.2 Data

3.2.1 *In vitro* / physicochemical data

A literature search was performed to collect available information on physicochemical properties of cetirizine. The obtained information from literature is summarized in **Error! Reference source not found.**. **Error! Reference source not found.** shows the parameters that were additionally used for the lactation PBPK model.

Table S1 Physicochemical parameters used as input for the cetirizine PBPK models

Parameter	Value	Unit	Description	Source
MW	388.90	g/mol	Molecular weight	Drugbank
pK_a	2.9 (acid)<br>8.0 (base)<br>2.2 (base)		Logarithm of the acid dissociation constant	[1]

Solubility (pH 7)	101	mg/mL	Aqueous solubility	Drugbank
Log P	1.50	-	Log_{10} of the partition coefficient between octanol and water (~lipophilicity)	[6]
f_u	0.07		Fraction unbound in human plasma	Drugbank
**Kidney plasma clearance –** "plasma clearance"	0.49	mL/min/kg	Calculated based on apparent clearance (34.3 mL/min) and assumed bioavailability of 0.7	[1]

Table S2 Physicochemical parameters used as input for the lactation PBPK model of cetirizine

Parameter	Value	Unit	Description	Source
Milk logP[a]	1.50	-	Log10 of the partition coefficient between octanol and water	[6]
	3.48	-	LogP of nonionic species	MarvinSketch
$LogD_{7.2}$	0.26		Log_{10} of the partition coefficient between octanol and water at pH 7.2	MarvinSketch
$LogD_{7.4}$	0.24		Log_{10} of the partition coefficient between octanol and water at pH 7.4	MarvinSketch
HBD	1.00		Hydrogen bond donors	Pubchem
PSA	53.00	$Å^2$	Polar surface area	Pubchem

[a] Milk logP is Log_{10} of the partition coefficient between octanol and water and is used as input for the calculations in the postpartum model (see equations below). In theory, this value is identical to the logP specified in Table S1. However, in some PBPK models, logP (Table S1) might be optimized using parameter identication. Therefore, it was chosen to use a separate parameter (i.e. Milk logP) to represent the logP used as input for the equations in the postpartum model.

The default equations for free fraction in human milk and logD that were implemented in the spatial structure building block that was developed for the postpartum women are described below. Alternatively, these values can be overwritten by values calculated elsewhere (e.g. MarvinSketch) or determined *in vitro.*

The free fraction in human milk was calculated with the equations proposed by Atkinson and Begg [7], as follows:

$$f_{u\_skimmed\ milk} = \frac{f_u \times 0.448}{(0.000694^{0.448} + f_u^{0.448})}$$

$$P_{milk} = 10^{(-0.88+1.29\times \mathrm{logD7.2})}$$

$$\text{Total free fraction in milk} = \frac{1}{(\frac{0.955}{f_{u\_skimmed\ milk}} + 0.045 \times P_{milk})}$$

Where: f_u *skimmed milk:* binding to proteins in milk; P_{milk}: partitioning between aqueous and lipid phase of milk; Total free fraction in milk: 'total' free fraction, i.e. accounting for both protein and lipid binding processes.

LogD values taking into account up to three pka values (as provided in the compound building block), were calculated as follows:

$$LogD = \mathrm{Log}\,P + Log_{10}(logD_{factor})$$

With Milk logP (Table S2) as input for logP

$$LogD_{factor} = \mathrm{K}_1 + (\mathrm{K}_2 + \mathrm{K}_3 + \mathrm{K}_4) \times \mathrm{base}^1 + \mathrm{K}_5 \times \mathrm{base}^{\max(\mathrm{CT}_0+\mathrm{CT}_1;-\mathrm{CT}_0-\mathrm{CT}_1)} + \mathrm{K}_6 \times \mathrm{base}^{\max(CT_0+CT_2;-CT_0-CT_2)} + \mathrm{K}_7 \times \mathrm{base}^{\max(\mathrm{CT}_2+\mathrm{CT}_1;-\mathrm{CT}_2-\mathrm{CT}_1)} + \mathrm{K}_8 \times \mathrm{base}^{\max(\mathrm{CT}_0+\mathrm{CT}_1+\mathrm{CT}_2;-\mathrm{CT}_0-\mathrm{CT}_1-\mathrm{CT}_2)}$$

$$K_1 = F_1 \times F_2 \times F_3$$
$$K_2 = (1 - F_1) \times F_2 \times F_3$$
$$K_3 = F_1 \times (1 - F_2) \times F_3$$
$$K_4 = F_1 \times F_2 \times (1 - F_3)$$
$$K_5 = (1 - F_1) \times (1 - F_2) \times F_3$$
$$K_6 = (1 - F_1) \times F_2 \times (1 - F_3)$$
$$K_7 = (1 - F_1) \times F_2 \times (1 - F_3)$$
$$K_8 = (1 - F_1) \times (1 - F_2) \times (1 - F_3)$$

F1 = CT0 != CT_NEUTRAL ? 1/(1+10^(CT0*(pKa_0- pH))) : 1
F2 = CT1 != CT_NEUTRAL ? 1/(1+10^(CT1*(pKa_1- pH))) : 1
F3 = CT2 != CT_NEUTRAL ? 1/(1+10^(CT2*(pKa_2- pH))) : 1

With CT = compound type (-1: acid; +1: base; 0: neutral), and pH = 7.2 or 7.4 respectively for $logD_{7.2}$ *and* $logD_{7.4}$

The transports that were added in the passive transport building block for 'transfer to milk' and 'transfer from milk' are based on secretion and reuptake and clearance values, Cl_{sec} and Cl_{re}, which were calculated according to the empirical equations proposed by Koshimichi et al. 2011 [5], as follows:

$$\text{Log } CL_{re} = 2.793 + 0.179 \times LogP - 0.132 \times HBD$$

$$\text{Log } CL_{sec} = 3.367 \times Log10(MW) - 0.164 \times (LogP - LogD) - 0.015 \times PSA - 3.912$$

3.2.2 Clinical data

Literature searches were performed to collect available data on cetirizine in adults and postpartum women. The cetirizine reference PBPK model was developed using a clinical study with pharmacokinetic (PK) blood sampling after oral administration of 20 mg cetirizine [8]. Additionally, 4 clinical trials with an oral administration (10 mg), one of them being with multiple dose administration, were used for verification [9–11] [12].

The evaluation of the predictive performance of the cetirizine lactation PBPK model was performed using a study where cetirizine was administered as an oral dose of 10 mg per day to 3 lactating women [13]. The women were 5 to 6 months postpartum.

Detailed information and data from the studies used for model building, verification, and lactation model can be found in Supplementary material 1 and 2.

3.2.2.1 Model building

The studies that were used for model building are shown in Table S3 (training data).

Table S3 Summary of studies used for PBPK model building of cetirizine in reference populations

Study ID	Reference	Arm/treatment/information used for model building
Xu 2011	[8]	18 subjects received 20 mg PO tablet (single dose)
Xu 2011	[8]	18 subjects received 20 mg PO tablet (single dose)

Table S4 Demographic information

Study ID	Reference	Number of subjects (female ratio)	Age (year)	Weight (kg)
Xu 2011	[8]	18 (0)	32.28 ± 4.73	66.33 ± 5.78

3.2.2.2 Model verification

The studies that were used to evaluate the predictive performance of the PBPK model are shown in Table S5 (**verification data**).

Table S5 Summary of studies used for model verification of cetirizine PBPK model in reference population

Study ID	Reference	Arm/treatment/information used for model verification
El-Say 2016	[9]	6 subjects received 10 mg PO tablet (single dose)
Korsgren 2007	[10]	8 subjects received 10 mg PO tablet (multiple dose)

Pharmacology 2009	[12]	28 subjects received 10 mg PO tablet (single dose)
Pharmacology 2009	[12]	28 subjects received 10 mg PO tablet (single dose)
Pharmacology 2009	[12]	28 subjects received 10 mg PO tablet (single dose)
Pharmacology 2009	[12]	28 subjects received 10 mg PO tablet (single dose) in fed condition
Derakhshandeh 2009	[11]	12 subjects received 10 mg PO tablet (single dose)
Derakhshandeh 2009	[11]	12 subjects received 10 mg PO tablet (single dose)

Table S6 Demographic information

**Study ID**	**Reference**	**Number of subjects (female ratio)**	**Age (year)**	**Weight (kg)**
El-Say 2016	[9]	8 (0)	27 ± 1.9	69.5 ± 5.9
Korsgren 2007	[10]	8 (-)	-	-
Pharmacology 2009	[12]	28 (-)	-	-
Derakhshandeh 2009	[11]	12 (-)	29 ± 2.3	75.3 ± 9.5

3.2.2.3 Lactation PBPK model

Table S7 shows the study that was used for the lactation PBPK model.

Table S7 Summary of study used for PBPK model development of cetirizine in lactating women

**Study ID**	**Publication**	**Arm/treatment/information used for model building and verification**
Wilkerson 2021	[13]	3 women (5/6 months postpartum) received PO 10 mg (multiple dose)

3.3 Model Parameters and assumptions

3.3.1 Absorption

Cetirizine is rapidly absorbed, and administered as cetirizine hydrochloride. Therefore, we calculated the corresponding dose of cetirizine as input for the PBPK model based on the molecular weight. The *in vitro* dissolution profile was used for the formulation [14]. The intestinal permeability was based on the reported caco-2 permeability [15].

3.3.2 Distribution

An important parameter influencing the distribution of a compound is lipophilicity. The logPz was used as input for lipophilicity [6]. The tissue partition coefficients (Kp) calculation was according to 'Rodgers and Rowland' and the cellular permeability calculation was 'PK-Sim Standard'.

3.3.3 Metabolism and excretion

The final model applies renal elimination as kidney plasma clearance. The implemented plasma clearance was based on the reported total clearance, and the assumed bioavailability (F = 0.7) [1]. The clearance was further optimized via parameter identification based on observed data [8].

3.3.4 Secretion to milk

To model the transfer process of amoxicillin into human milk, both the secretion (CL_{sec}) and reuptake clearance (CL_{re}) were calculated using the empirical equations developed by Koshimichi *et al.* 2011 (see **Error! Reference source not found.**) [4].

First, in MoBi®, a spatial structure for the postpartum women was constructed, similar to the workflow from Dallmann *et al.* 2018 [2]. Here, breasts were added as a compartment. In addition, the human milk was connected to the plasma subcompartment of the breasts. The human milk volume was specified as 0.5 L to represent the structure of Koshimichi *et al.* 2011, and a geometric standard deviation of 1.16 was assumed in the population. The free fraction in human milk, and logD values were implemented as the equations described previously. The transfer between plasma and milk was defined as two kinetic processes (transfer to milk and transfer from milk) under passive transports (see below). Next, the simulation was combined with the postpartum population from Job *et al.* 2021 in PK-Sim to account for the postpartum physiology [3].

<u>*Kinetics*</u>

Transfer to milk

$$\frac{dNmilk}{dt} = Cplasma \times f_u \times CL_{sec}$$

where Cplasma is the concentration in plasma (in breast compartment), f_u is the free fraction in plasma and CL_{sec} is the secretion clearance.

Transfer from milk

$$\frac{dNplasma}{dt} = Cmilk \times f_u \times CL_{re}$$

where Cmilk is the concentration in human milk, f_u is the total free fraction in human milk (protein and lipid) and CL_{re} is the reuptake clearance.

The median simulated plasma and human milk concentration-time profiles can be used to calculate the M/P ratio as follows:

$$M/P\ ratio\ = \frac{AUC_{milk}}{AUC_{plasma}}$$

3.3.5 Automated parameter optimization

The following table depicts the results of the final parameter optimization according to the different clinical studies.

a) Xu et al. (2011) 20 mg PO [8]

**Model parameter**	**Optimized value**	**Unit**
Kidney - Plasma clearance	0.33	mL/min/kg

3.4. Infant dosage calculation

Infant dosage via human milk was then calculated based on the predicted (average and maximal) steady-state cetirizine concentration in human milk, as well as the daily milk intake volume. The daily infant dosage was then compared to the maternal dosage, resulting in the relative infant dose (RID).

$$Daily\ infant\ dosage = C_{average} * 150\ \frac{mL}{kg\ .day}$$

$$Daily\ infant\ dosage = C_{max} * 150\ \frac{mL}{kg\ .day}$$

$$Relative\ infant\ dose\ (RID) = \frac{Infant\ dosage}{Maternal\ dosage} * 100\ \%$$

4. Results

Both the reference and postpartum PBPK model of cetirizine was developed and verified with clinical PK data.

The models were evaluated covering studies including in particular:

- Single and multiple doses
- Oral dose range from 10 up to 20 mg
- Fed and fasted state
- Paired milk/plasma data

The model describes the elimination of cetirizine renally via kidney plasma clearance. Moreover, secretion and reuptake to human milk were described by CL_{sec} and CL_{re}.

The next sections show:

- The final model parameters for the building blocks: section 4.1
- The overall predictive performance: section 4.2
- The simulated versus observed concentration-time profiles for the clinical studies used for model building and for model verification: section 4.3

4.1 Final input parameters

The compound values of the final postpartum PBPK model for cetirizine are illustrated below.

Physicochemical parameters

Parameter	Value	Unit	Source
MW	388.90	g/mol	Drugbank
pKa	2.90 (acid)<br>8.00 (base)<br>2.2 (base)	-	[1]
Solubility	101.00	mg/mL	Drugbank
Lipophilicity	1.50	-	[6]
f_u	0.07	-	Drugbank
Small molecule (Y/N)	Yes	-	
Plasma protein binding partner	Albumin		

Calculation methods

Name	Value
Tissue partition coefficients	Rodgers and Rowland
Cellular permeabilities	PK-Sim Standard

AMDE-related parameters

Parameter	Value	Unit	Source
Intestinal permeability	1.14E-6	cm/min	[15]
Kidney plasma clearance	0.33	mL/min/kg	Parameter identification

Formulation-related parameters

Type: Table

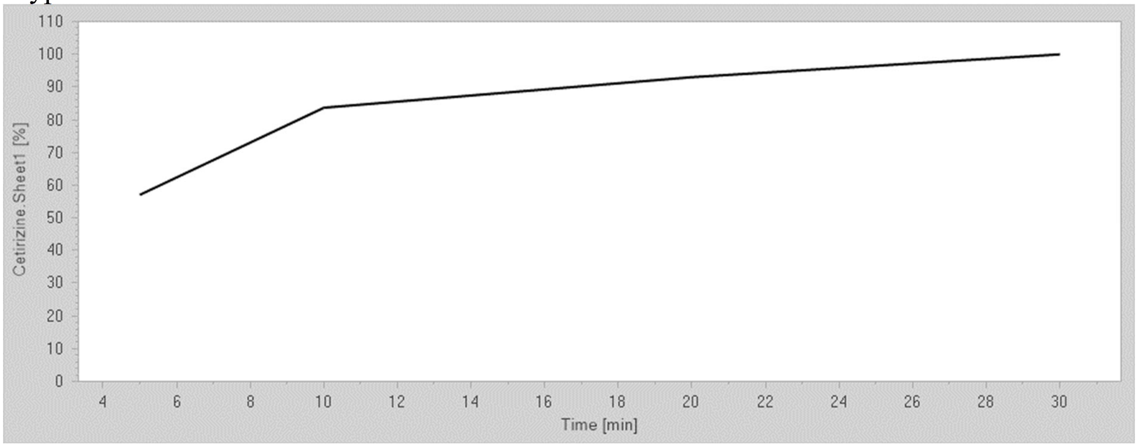

In vitro dissolution profile of cetirizine

Physicochemical and physiological parameters relevant to the lactation model

Parameter	Value	Unit	Source
Milk log P	3.48	Log units	MarvinSketch
HBD	1.00	-	Pubchem
PSA	53.00	$Å^2$	Pubchem
CL_{sec}	0.05	L/min	Default
CL_{re}	0.03	L/min	Default
*f*u_skimmed milk[*a*]	0.89	-	Default
Pmilk[b]	0.29	-	Default
Total free fraction in milk[c]	0.92	-	Default
$logD_{7.2}$	0.26	Log units	MarvinSketch
$logD_{7.4}$	0.24	Log units	MarvinSketch

[a] binding to proteins in milk; [b] partitioning between aqueous and lipid phase of milk; [c] total free fraction, accounting for both protein and lipid binding

4.2 Diagnostic plots

The geometric mean fold errors (GMFE) on AUC and Cmax were 1.06 and 1.10 for the model building dataset, and 1.08 and 1.07 for the model verification dataset.
The following shows the predictive performance graph for Cmax and AUC of cetirizine for the PBPK model performance of all data used.
Predicted over observed ratio values of all data listed in section 3.2.2 are presented below.

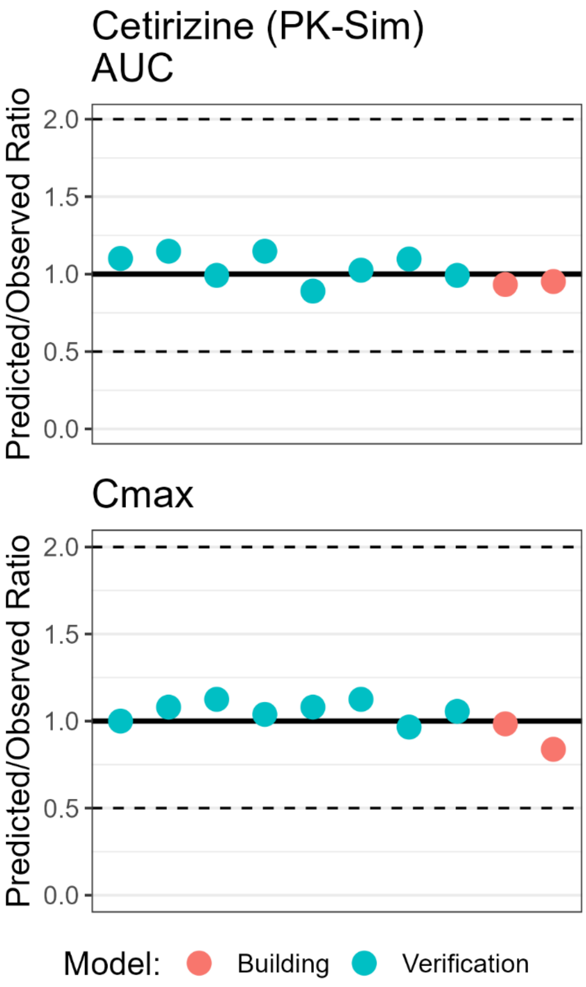

Figure S3 Predicted over observed ratio profile

Table S8 Ratio between the predicted and observed pharmacokinetic parameters of cetirizine in different dosing regimens for model building

Study ID/ Reference	Dose/ Route	AUC_{obs} (mg*h/L)	AUC_{pred} (mg*h/L)	Fold error	$C_{max\ obs}$ (mg/L)	$C_{max\ pred}$ (mg/L)	Fold error
Xu 2011	20 mg PO SD (1)	6.39	5.96	0.93	0.63	0.62	0.98
Xu 2011	20 mg PO SD (2)	6.26	5.96	0.95	0.74	0.62	0.84

Table S9 Ratio between the predicted and observed pharmacokinetic parameters of cetirizine in different dosing regimens used for model verification

Study ID/ Reference	Dose/ Route	AUC_{obs} (mg*h/L)	AUC_{pred} (mg*h/L)	Fold error	$C_{max\ obs}$ (mg/L)	$C_{max\ pred}$ (mg/L)	Fold error
Pharmacology 2009	10 mg PO SD (1)	2.45	2.43	0.99	0.24	0.27	1.13
Pharmacology 2009	10 mg PO SD (2)	2.73	2.43	0.89	0.25	0.27	1.08
Pharmacology 2009	10 mg PO SD fed	2.41	2.39	0.99	0.18	0.19	1.06
Pharmacology 2009	10 mg PO SD (3)	2.37	2.43	1.03	0.24	0.27	1.13
Derakhshandeh 2009	10 mg PO SD (1)	2.03	2.33	1.15	0.25	0.27	1.08
Derakhshandeh 2009	10 mg PO SD (2)	2.03	2.33	1.15	0.26	0.27	1.04
El-Say 2016	10 mg PO SD	2.36	2.59	1.10	0.29	0.28	0.97
Korsgren 2007	10 mg PO MD	2.27	2.50	1.10	0.29	0.29	1.00

4.3 Concentration-time profiles

Simulated versus observed concentration-time profiles of all data listed in section 3.2.2 are presented below. The original.pksim5 are provided in Supplemented material 3.

4.3.1 Model building

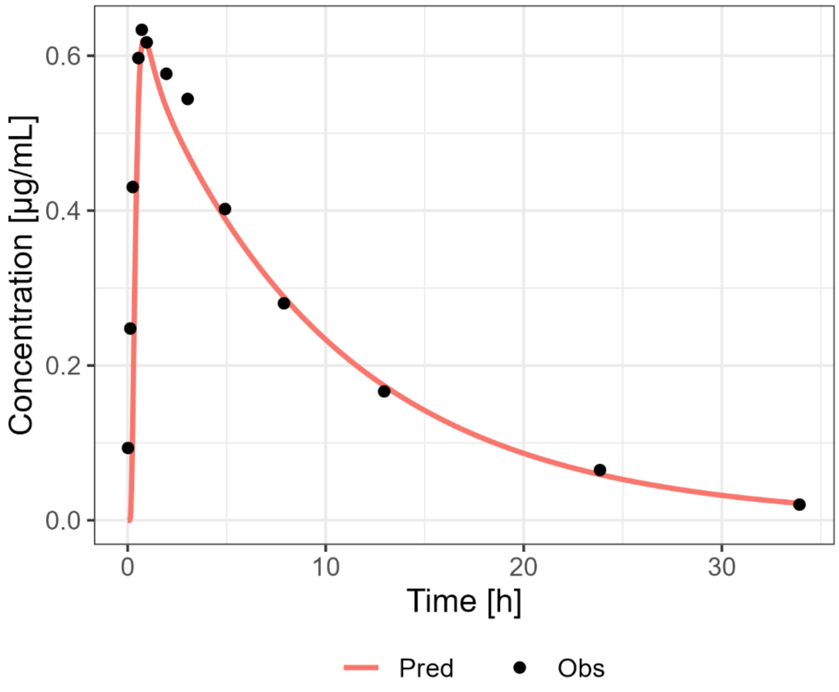

Figure S4 Predicted (Pred) versus observed (Obs) concentration-time profile after administration of 20 mg PO SD reference [8]

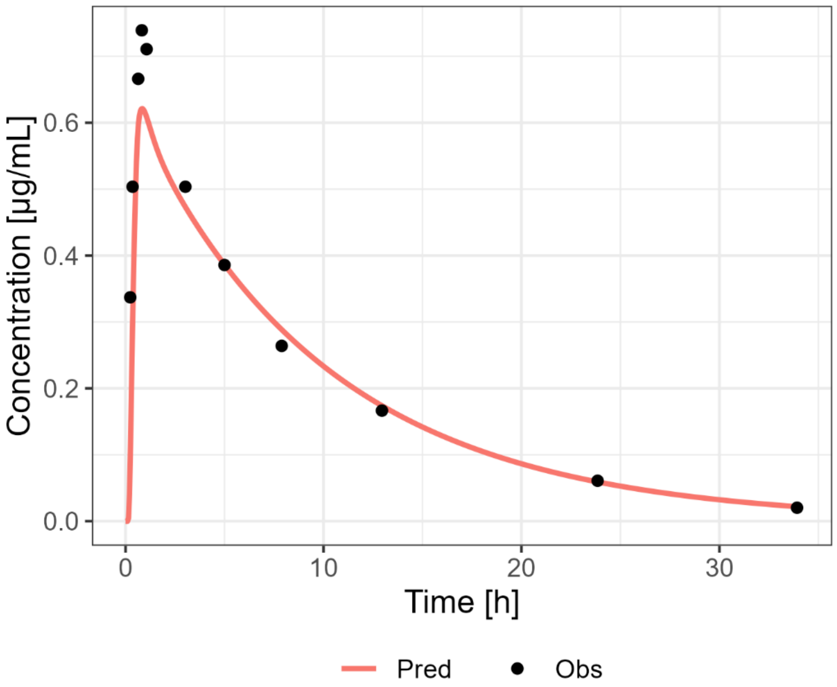

Figure S5 Predicted (Pred) versus observed (Obs) concentration-time profile after administration of 20 mg PO SD test [8]

4.3.2 Model verification

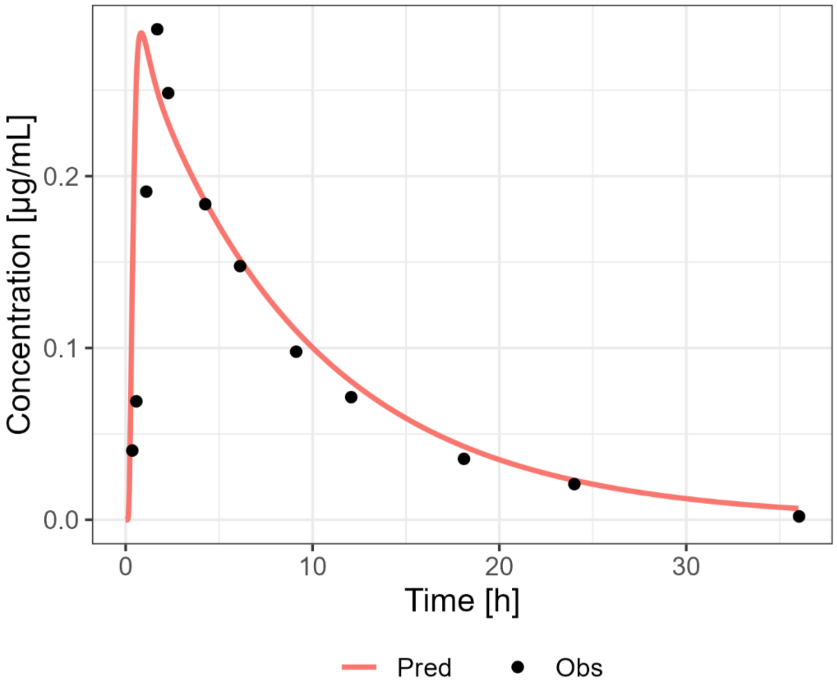

Figure S6 Pedicted (Pred) versus observed (Obs) concentration-time profile after administration of 10 mg PO SD [9]

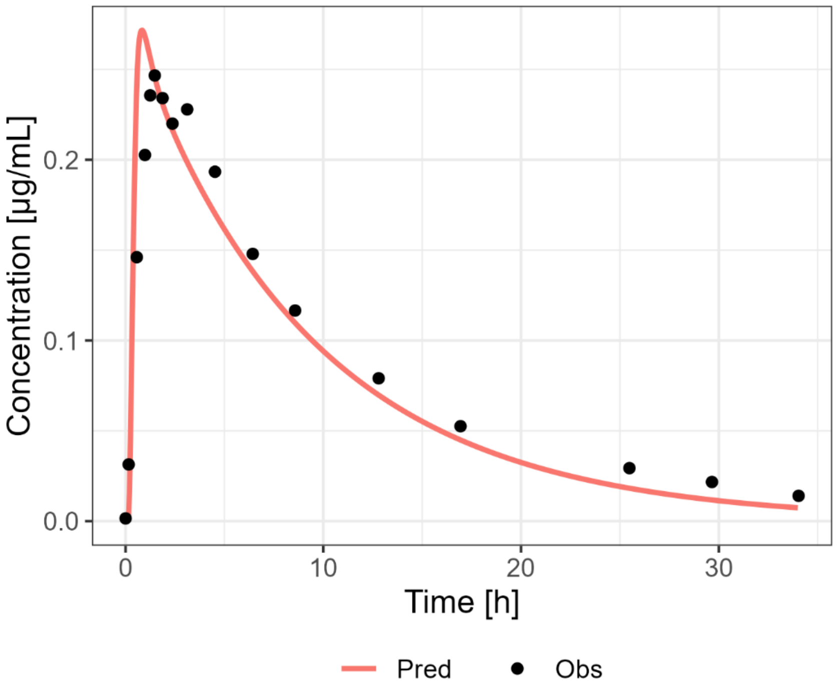

Figure S7 Pedicted (Pred) versus observed (Obs) concentration-time profile after administration of 10 mg PO SD [12]

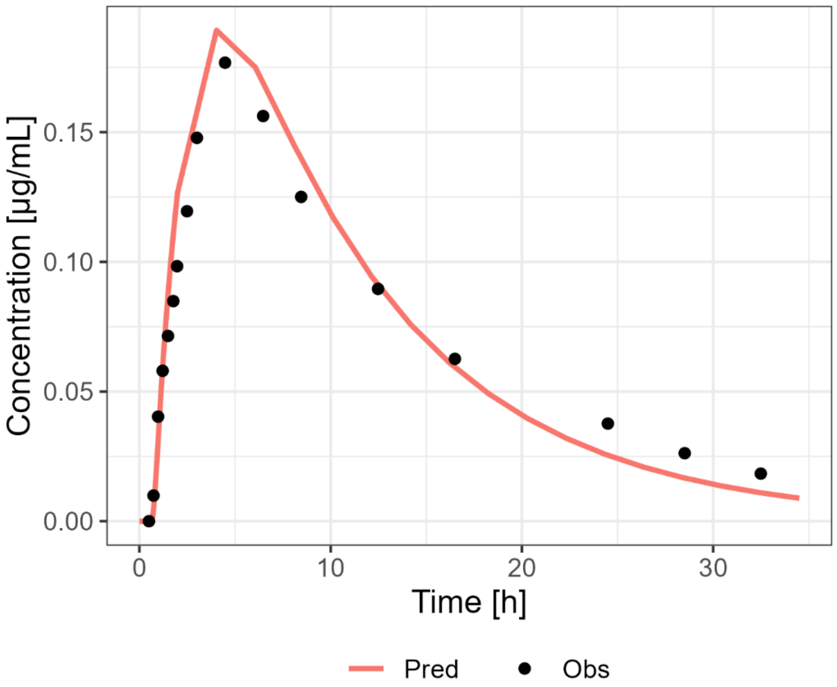

Figure S8 Pedicted (Pred) versus observed (Obs) concentration-time profile after administration of 10 mg PO SD fed [12]

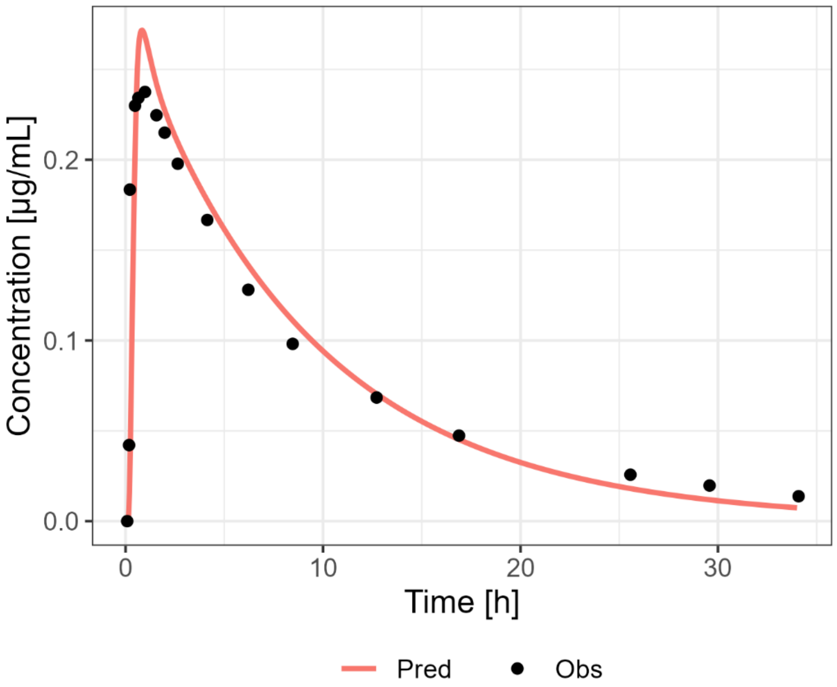

Figure S9 Pedicted (Pred) versus observed (Obs) concentration-time profile after administration of 10 mg PO SD ODT [12]

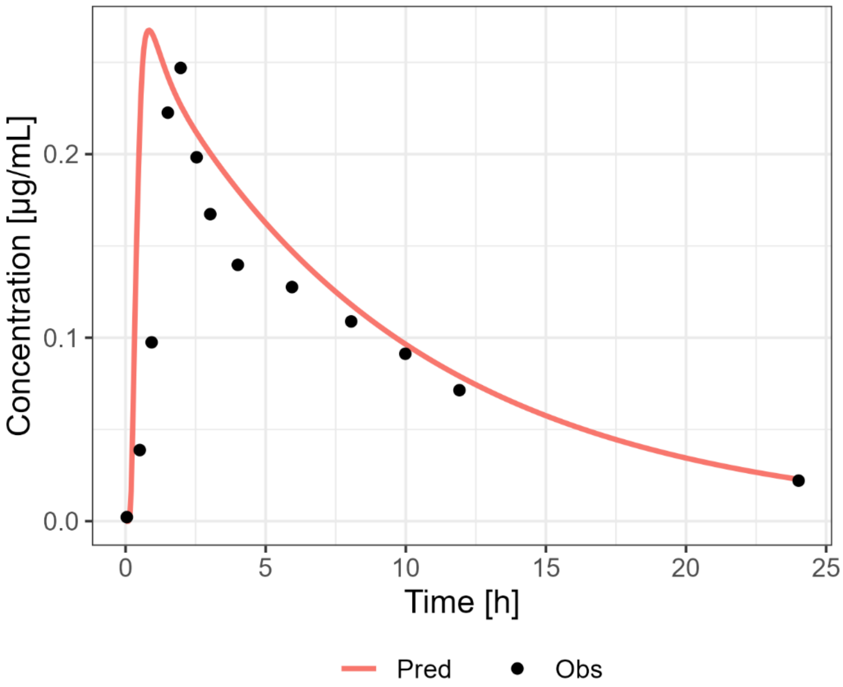

Figure S10 Pedicted (Pred) versus observed (Obs) concentration-time profile after administration of 10 mg PO SD Zyrtecset [11]

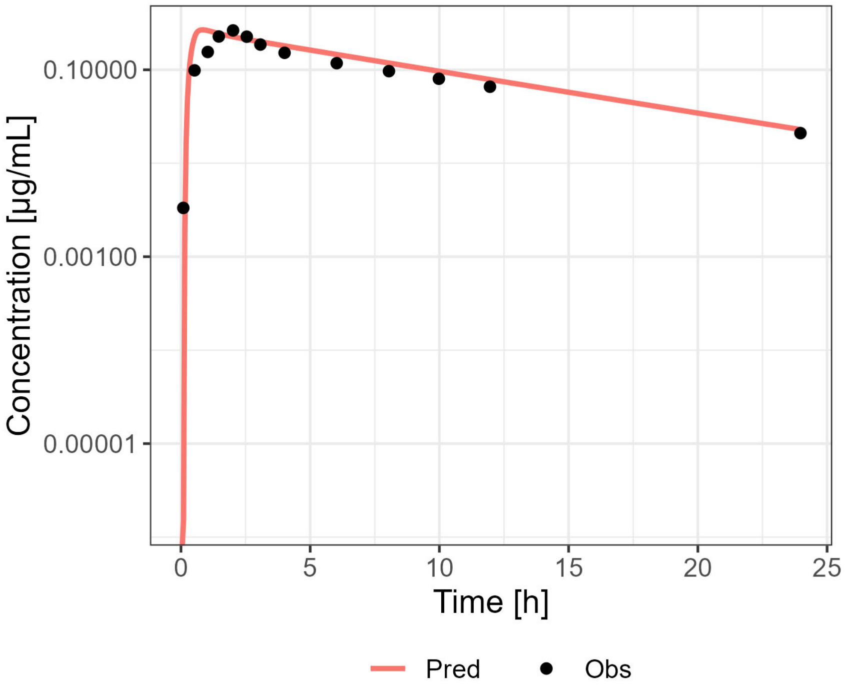

Figure S11 Pedicted (Pred) versus observed (Obs) concentration-time profile after administration of 10 mg PO SD test [11]

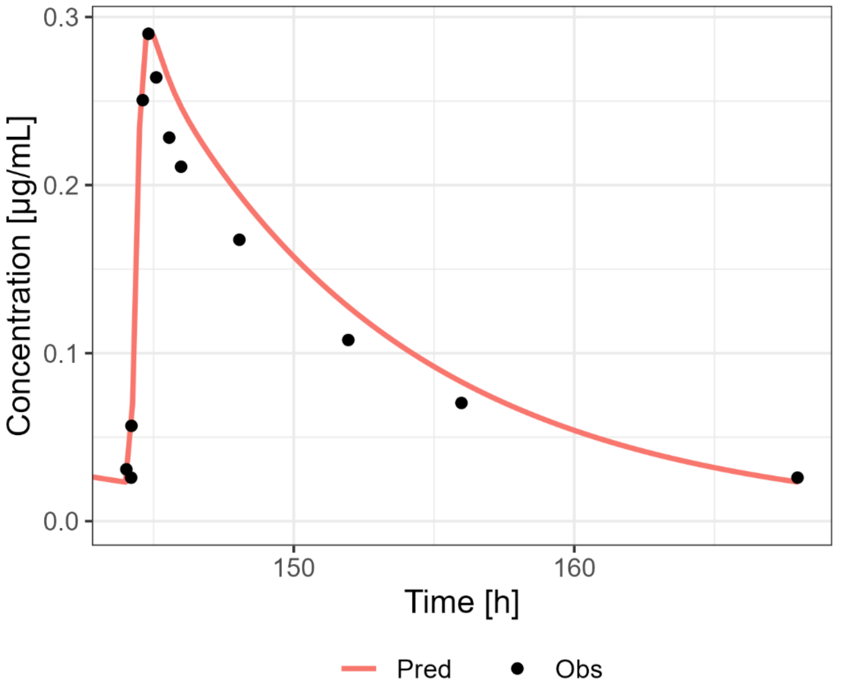

Figure S12 Pedicted (Pred) versus observed (Obs) concentration-time profile after administration of 10 mg/day PO SD [10]

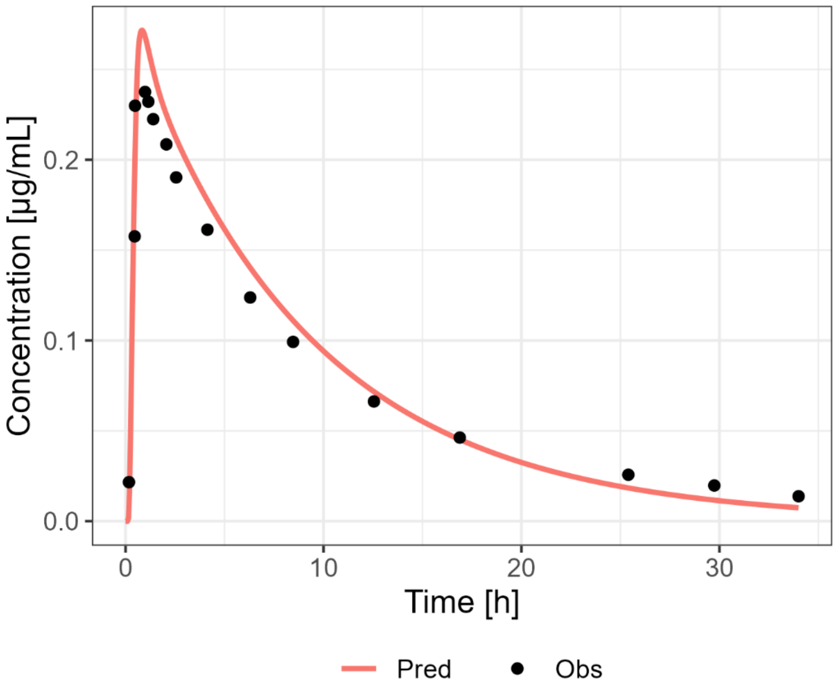

Figure S13 Pedicted (Pred) versus observed (Obs) concentration-time profile after administration of 10 mg PO SD Zyrtex [12]

4.3.3 Lactation PBPK model

A sample size of 1000 individuals, three months postpartum, was used in each simulation of the virtual lactation population.

<u>Model A</u>: First, the transfer of cetirizine was predicted using the LogP value of 1.50, and the default values for logD based on the equations implemented in the spatial structure building block as described above.

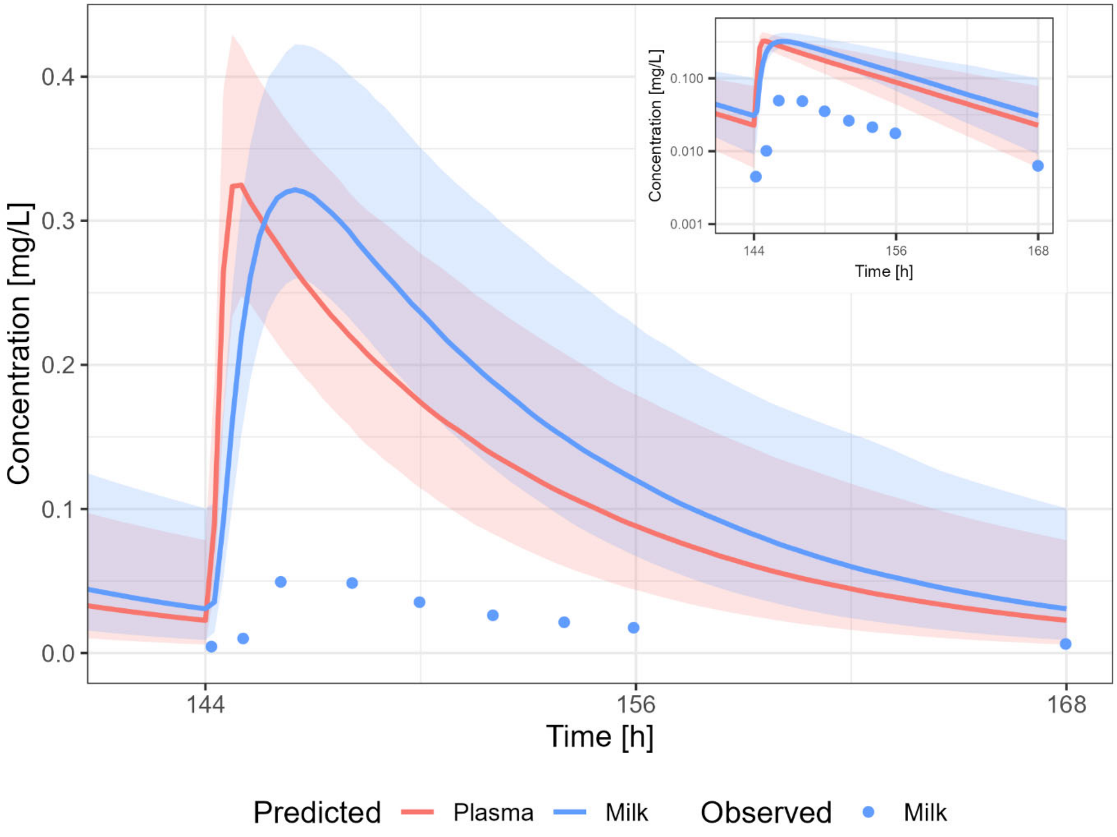

Figure S14 Pedicted (Pred) versus observed (Obs) concentration-time profile after administration of 10 mg MD [13]

A dosing regimen of PO 10 daily was assumed to calculate the milk transfer of cetirizine.

Dosing interval: 24 h	Plasma	Milk
C_{max} (mg/L)	0.32	0.32
AUC (mg*h/L)	2.74	3.35
Cave (mg/L)	0.11	0.14

M/P ratio = 1.22

Model B: Alternatively, the logD values were overwritten with the values obtained from MarvinSketch (see section 4.2).

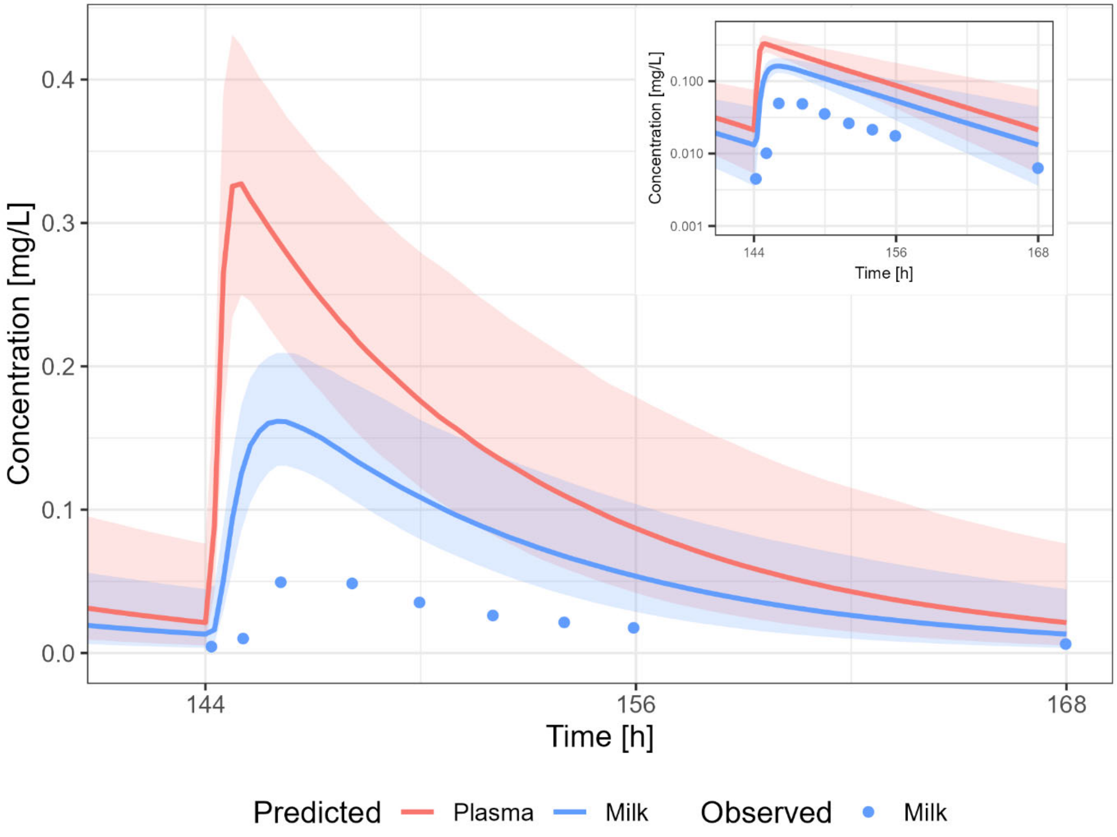

Figure S15 Pedicted (Pred) versus observed (Obs) concentration-time profile after administration of 10 mg MD [13]

A dosing regimen of PO 10 daily was assumed to calculate the milk transfer of cetirizine.

Dosing interval: 24 h	Plasma	Milk
C_{max} (mg/L)	0.33	0.16
AUC (mg*h/L)	2.74	1.57
Cave (mg/L)	0.11	0.07

M/P ratio = 0.57

Model C: Finally, the LogP value as well as the LogD values were overwritten with the values obtained from MarvinSketch (see section 4.2).

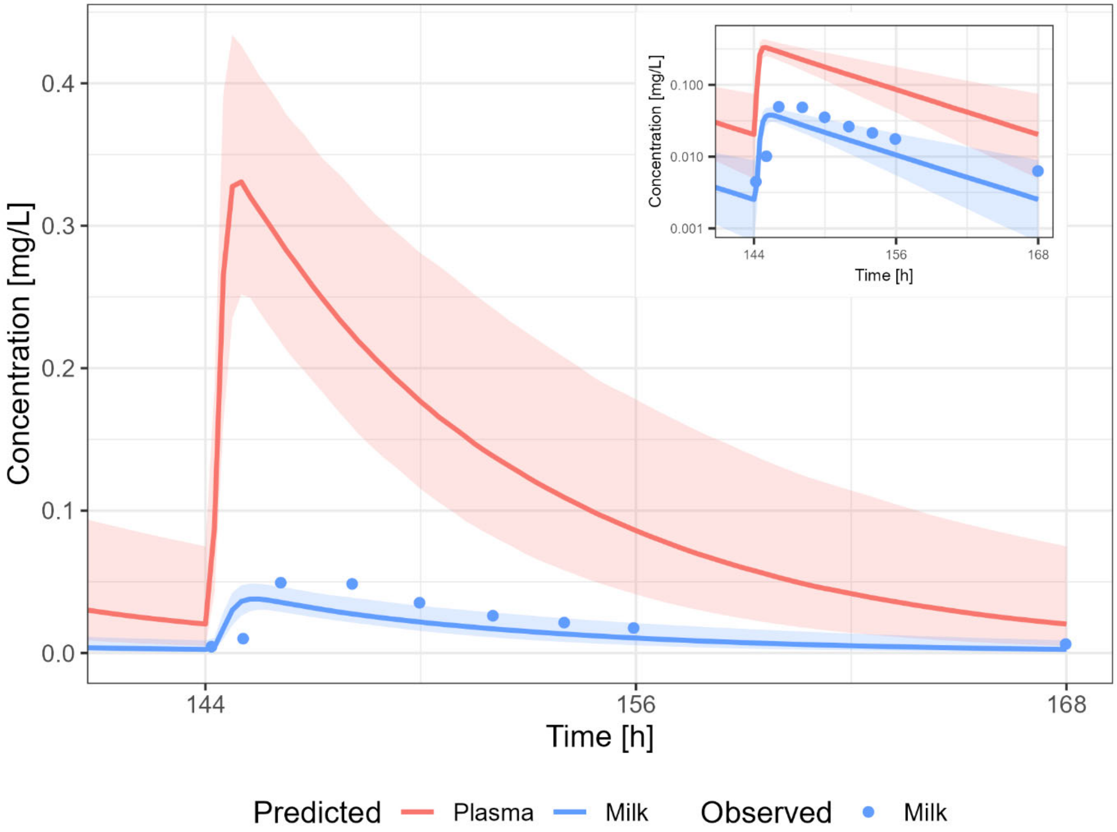

Figure S16 Pedicted (Pred) versus observed (Obs) concentration-time profile after administration of 10 mg MD [13]

A dosing regimen of PO 10 daily was assumed to calculate the milk transfer of cetirizine.

Dosing interval: 24 h	Plasma	Milk
C_{max} (mg/L)	0.33	0.04
AUC (mg*h/L)	2.74	0.33
Cave (mg/L)	0.11	0.01

M/P ratio = 0.12

Option C was selected as final PBPK model for lactation for amoxicillin. The M/P ratio was 0.12.

4.4 Estimated Pediatric exposure

A maternal dosing regimen of 10 mg cetirizine hydrochloride (= 8.42 mg cetirizine), every 24 h was used to calculate the infant exposure. The daily infant dosage and relative infant dose (RID) for 3 months old infants were calculated using a milk intake of 150 mL/kg/day. The daily infant dosage was 0.002 mg/kg/day (RID: 1.24 %) or 0.01 mg/kg/day (RID: 3.62 %) based on the average steady-state concentration and maximum concentration in human milk, respectively.

5. Discussion

First, the reference PBPK model was developed and evaluated. Evaluation of the predictive performance showed that the reference PBPK model for cetirizine was able to capture the pharmacokinetic behavior of the medicines in healthy volunteers and/or patients.

Next, the PBPK model was extended to a lactation PBPK model. The PBPK model results in an overprediction of the human milk concentrations.

Alternatively, the LogP and LogD values used in the calculation of the milk parameters were overwritten with the LogP and LogD values obtained from MarvinSketch, which was also used to calculate LogD parameters by Koshimichi *et al. 2011.* The PBPK model results in a reasonable prediction of the human milk concentrations, with most datapoints within the 5-95th percentile of the population prediction.

An observed M/P ratio could not be found in literature. Therefore, the M/P ratio was calculated using the observed steady-state AUC in human milk (0.50 mg*h/L), and the observed plasma concentration in non-lactating patients receiving the same dosing regimen (2.50 mg*h/L). The resulting M/P ratio was 0.2, which is similar to the predicted M/P ratio (0.12).

The calculated infant dosage of cetirizine via breastfeeding was very low, especially when compared to the maternal daily dosage.

6. Conclusions

The herein presented PBPK model adequately describes the PK of cetirizine in adults, including breastfeeding women. In particular, it applies renal clearance. The PBPK model was able to reasonably predict the human milk concentrations of cetirizine (M/P ratio: 0.12). The daily infant dosage was 0.002 mg/kg/day (RID: 1.24 %) or 0.01 mg/kg/day (RID: 3.62 %) based on the average steady-state concentration and maximum concentration in human milk, respectively.

7. List of Appendix and Supplementary Materials

Supplementary material 1 – ObsDataPK_OSP_reference_Cetirizine
Supplementary material 2 – ObsDataPK_OSP_lactation_Cetirizine
Supplementary material 3 – Cetirizine.pksim5

8. References

1. Chen, C. Physicochemical, Pharmacological and Pharmacokinetic Properties of the Zwitterionic Antihistamines Cetirizine and Levocetirizine. *Curr. Med. Chem.* **2008**, *15*, 2173–2191, doi:10.2174/092986708785747625.
2. Dallmann, A.; Himstedt, A.; Solodenko, J.; Ince, I.; Hempel, G.; Eissing, T. Integration of Physiological Changes during the Postpartum Period into a PBPK Framework and Prediction of Amoxicillin Disposition before and Shortly after Delivery. *J. Pharmacokinet. Pharmacodyn.* **2020**, *47*, 341–359, doi:10.1007/s10928-020-09706-z.
3. Dallmann, A.; Solodenko, J.; Ince, I.; Eissing, T. Applied Concepts in PBPK Modeling: How to Extend an Open Systems Pharmacology Model to the Special Population of Pregnant Women. *CPT pharmacometrics Syst. Pharmacol.* **2018**, *7*, 419–431, doi:10.1002/PSP4.12300.
4. Job, K.M.; Dallmann, A.; Parry, S.; Saade, G.; Haas, D.M.; Hughes, B.; Berens, P.; Chen, J.; Fu, C.; Humphrey, K.; et al. Development of a Generic Physiologically-Based Pharmacokinetic Model for Lactation and Prediction of Maternal and Infant Exposure to Ondansetron via Breast Milk. *Clin. Pharmacol. Ther.* **2022**, *111*, 1111–1120, doi:10.1002/cpt.2530.
5. Koshimichi, H.; Ito, K.; Hisaka, A.; Honma, M.; Suzuki, H. Analysis and Prediction of Drug Transfer into Human Milk Taking into Consideration Secretion and Reuptake Clearances across the Mammary Epithelia. *Drug Metab. Dispos.* **2011**, *39*, 2370–2380, doi:10.1124/dmd.111.040972.
6. Pagliara, A.; Testa, B.; Carrupt, P.A.; Jolliet, P.; Morin, C.; Morin, D.; Urien, S.; Tillement, J.P.; Rihoux, J.P. Molecular Properties and Pharmacokinetic Behavior of Cetirizine, a Zwitterionic H1-Receptor Antagonist. *J. Med. Chem.* **1998**, *41*, 853–863, doi:10.1021/jm9704311.
7. Atkinson, U.C.; Begg, E.J.E.J.; Atkinson, H.C.; Begg, E.J.E.J. Prediction of Drug Distribution into Human Milk from Physicochemical Characteristics. *Clin. Pharmacokinet.* **1990**, *18*, 151–167, doi:10.2165/00003088-199018020-00005.
8. Xu; Feng-Guo P h a r m Ac Okine t i c s a Nd B Ioeq u i v a Len c e S Tu d y of T w o C e t i r i z Ine H y d r o c Hlo r Ide F o r m u l a t Ion s in H e a l t h y C Hine s e Ma Le V Ol u n t Ee Rs. **2011**, *59*, 0–4.
9. El-Say 2016.Pdf.
10. Korsgren, M.; Andersson, M.; Borgå, O.; Larsson, L.; Aldén-Raboisson, M.; Malmqvist, U.; Greiff, L. Clinical Efficacy and Pharmacokinetic Profiles of Intranasal and Oral Cetirizine in a Repeated Allergen Challenge Model of Allergic Rhinitis. *Ann. Allergy, Asthma Immunol.* **2007**, *98*, 316–321, doi:10.1016/S1081-1206(10)60876-9.
11. Derakhshandeh, K.; Mohebbi, M. Oral Bioavailability and Pharmacokinetic Study of Cetrizine HCl in Iranian Healthy Volunteers. *Res. Pharm. Sci.* **2009**, *4*, 113–121.
12. Pharmacology, C. Center for Drug Evaluation and Clinical Pharmacology and Biopharmaceutics Review (S). *Fda* **2009**, 1–5.
13. Wilkerson, H.; Datta, P.; Rewers-Felkins, K.; Baker, T.; Hale, T.W. Maternal Transfer

of Cetirizine Into Human Milk. *J. Hum. Lact.* **2021**, *37*, 135–138, doi:10.1177/0890334420949847.
14. Xhafaj, D.; Malaj, L.; Mileti, M. A Comparative Quality Control Study of Cetirizine Hydrochloride 10mg Tablets on the Albanian Pharmaceutical Market. *Int. J. Pharm. Pharm. Sci.* **2015**, *7*, 504–507.
15. Lee, J.B.; Zgair, A.; Dhiaa, A.T.; Zang, S.; Kagan, L.; Kim, T.H.; Kim, M.G.; Yun, H.; Fischer, P.M.; Gershkovich, P. View Metadata, Citation and Similar Papers at Core.Ac.Uk. *PENGARUH Pengguna. PASTA LABU KUNING (Cucurbita Moschata) UNTUK SUBSTITUSI TEPUNG TERIGU DENGAN PENAMBAHAN TEPUNG ANGKAK DALAM PEMBUATAN MIE KERING* **2020**, *1*, 274–282.
