# Supplementary material for: Generic Workflow to Predict Medicine Concentrations in Human Milk Using Physiologically-Based Pharmacokinetic (PBPK) Modelling—A Contribution from the ConcePTION Project
Source: Pharmaceutics. 2023 May 11;15(5):1469. doi: 10.3390/pharmaceutics15051469 (PMC10223637; doi:10.3390/pharmaceutics15051469)
Supplement: Supplementary file 1 [file pharmaceutics-15-01469-s001.zip › File S4. Evaluation_report_levetiracetam.pdf]

Building and evaluation of a Physiologically-Based PharmacoKinetic (PBPK) model for **levetiracetam** in adults and lactating women

Disclaimer: The research project leading to these results was conducted as part of the ConcePTION consortium. This report only reflects the personal views of the stated authors. The results of this report are only intended for research purpose, and are not intended to be used in clinical practice.

Glossary

AUC	Area Under the Curve
Cave	Average concentration
CES1	Carboxylesterase 1
CL_{re}	Reuptake clearance (i.e. from milk to blood)
CL_{sec}	Secretion clearance (i.e. from blood to milk)
Cmax	Maximum (~peak) concentration
DID	Daily Infant Dosage (expressed for instance in mg/kg/day)
f_u	Fraction unbound in plasma
GFR	Glomerular Filtration Rate
HBD	Hydrogen bond acceptor
IV	Intravenous administration
$LogD_{7.2}$	Logarithm of the partition coefficient between an octanol phase and an aqueous (buffer) phase at pH 7.2
$LogD_{7.4}$	Logarithm of the partition coefficient between an octanol phase and an aqueous (buffer) phase at pH 7.4
LogP	Logarithm of the partition coefficient between an octanol phase and (unbuffered) water as aqueous phase. This is the default parameter to express lipophilicity of a substance.
MD	Multiple doses
M/P ratio	Milk-to-Plasma ratio
MW	Molecular Weight (Da)
PAMPA	Parallel artificial membrane permeability assay
PBPK	Physiologically-Based PharmacoKinetic [*modeling*]
pKa	Logarithm of the acid dissociation constant
PO	Oral administration
PSA	Polar Surface Area
RID	Relative Infant Dose (%)
SD	Single dose

1. Table of Contents

1. Table of Contents 3

2. Introduction 4

3. Methods 5

3.1 Modelling strategy 5

3.1.1. Reference PBPK models 6

3.1.2. Lactation model 6

3.2 Data 6

3.2.1 *In vitro* / physicochemical data 6

3.2.2 Clinical data 9

3.3 Model Parameters and assumptions 11

3.3.1 Absorption 11

2.3.2 Distribution 11

3.3.3 Metabolism and excretion 12

3.3.4 Secretion to milk 12

3.3.5 Automated parameter optimization 13

3.4. Infant dosage calculation 13

4. Results 13

4.1 Final input parameters 13

4.2 Diagnostic plots 15

4.3 Concentration-time profiles 17

4.3.1 Model building 18

4.3.2 Model verification 19

4.3.3 Lactation PBPK model 26

4.4 Estimated Infant exposure 33

5. Discussion 33

6. Conclusion 34

7. List of Appendix and Supplementary Materials 35

8. References 35

2. Introduction

Levetiracetam Figure S1 is an (S)-enantiomer of the ethyl analog of piracetam. The medicine is approved for partial seizures. Levetiracetam is given orally or intravenously at a minimum of 500 mg twice daily with gradual upward titration to a maximum of 3 g/day. Rapid and nearly complete absorption from the gastrointestinal tract following oral administration. The plasma protein binding is less than 10 %. The volume of distribution ranges from 0.5 to 0.9 L/kg in adults and from 0.6 to 0.9 L/kg in children [1]. Levetiracetam is submitted to a partial metabolism, mainly by serine-type esterases present in red cells and liver. The apparent total body clearance amounts to 4.4 L/h. The half-life varies between 7.4 h and 7.9 h [2].

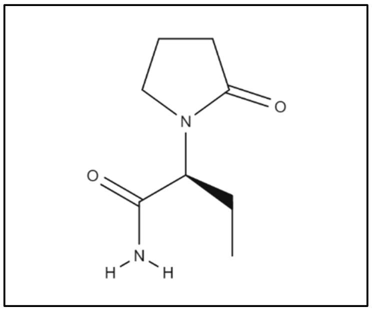

Figure S1 Chemical Structure of levetiracetam

The scope of this report is to:

(a) specify the details and underlying assumptions associated with the building of physiologically-based pharmacokinetic (PBPK) models for levetiracetam in adult healthy volunteers or patients, and in postpartum women during lactation.

(b) evaluate the predictive performance of these PBPK models. This is achieved by comparing model-predicted plasma or milk concentrations with corresponding clinical observations.

3. Methods

The software used for the development of PBPK models presented in this report is tabulated below:

Software	Version
PK-Sim®	v9.1
MoBi®	v9.1

3.1 Modelling strategy

In the present report, a reference PBPK model was first established for adults (patients as well as healthy volunteers), and subsequently verified against clinical pharmacokinetic data reported for levetiracetam in the scientific literature.

Relevant information on the anthropometry (height, weight) was gathered from the respective clinical studies, if reported. Information on physiological parameters (e.g. blood flows, organ volumes, hematocrit) in adults is available in the PK-Sim® database.

In a second step, a lactation PBPK model was developed, based on the general workflow described by Dallmann *et al. 2018* [3–5].

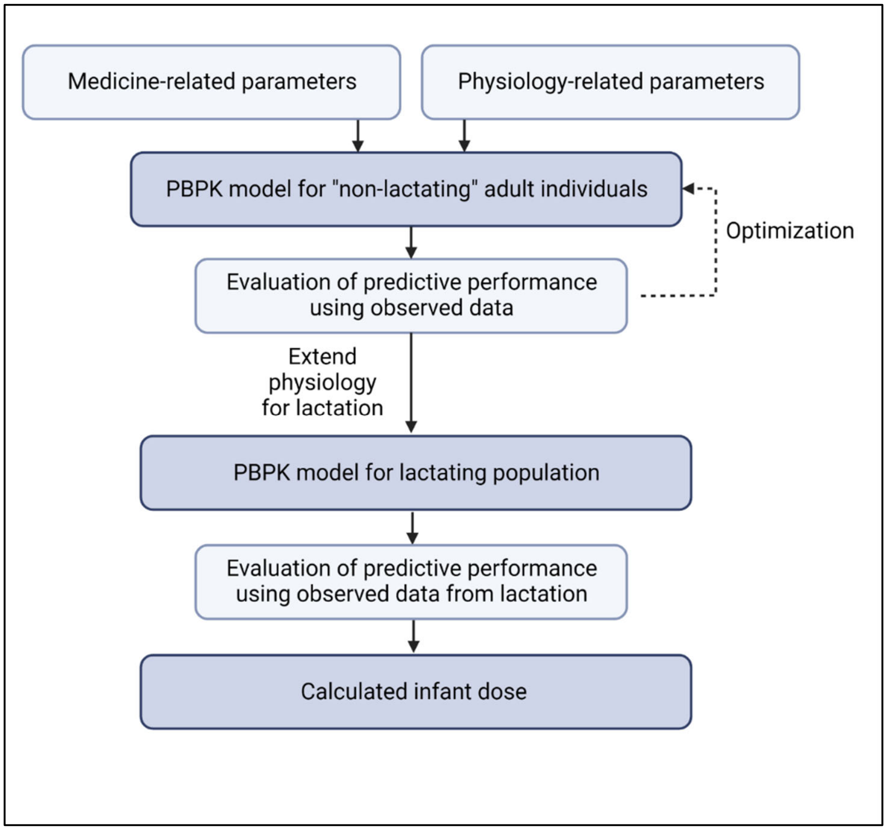

Figure S2 General workflow that was used in the present project to develop and evaluate the lactation PBPK model

Details about input data (physicochemical, *in vitro* and clinical data) can be found in section 3.2. Details about the structural models and their parameters can be found in section 3.3.

3.1.1. Reference PBPK models

The reference PBPK models were built based on studies with adult volunteers and/or adult patients, using the reported mean values for age, weight, height, and genetic background as described in each study protocol. When no information on these parameters could be found, a healthy male European individual, 30 years of age, with a body weight of 73 kg and a height of 176 cm was used.

The abundance (including population variability) of plasma proteins and enzymes/transporters that are integrated into PK-Sim are described in the publicly available 'PK-Sim Ontogeny Database Version 7.3' (PK-Sim Ontogeny Database Version 7.3).

It was assumed that carboxylesterase 1 (CES1) was responsible for the metabolism of levetiracetam. CES1 was implemented in accordance with literature [6]. The expression profile was manually set to have 100% expression in blood cells and liver [2]. Renal clearance was implemented as kidney plasma clearance, in accordance with literature [7].

Structural model selection was mainly guided by biological plausibility and by visual inspection of the predicted concentration time profiles in comparison with observed data. The generally applied acceptance criterium was less than 2-fold misprediction. Uninformed parameter values (see below) were estimated using the parameter identification module of PK-Sim®.

The predictive performance of the models was evaluated by simulating:

- Single intravenous dose studies
- Single and multiple oral dose studies

For some parameters, parameter optimization was performed as described below to obtain improved concordance between predicted profiles and observed data.

3.1.2. Lactation model

After development of the reference model, the model was exported to MoBi® and a lactation PBPK model was constructed. To model the passage of levetiracetam into human milk, i.e. across the blood/milk biological barrier, both the secretion (CL_{sec}) and reuptake clearance (CL_{re}) values were obtained using the empirical model developed by Koshimichi *et al. 2011* [8].

3.2 Data

3.2.1 *In vitro* / physicochemical data

A literature search was performed to collect available information on physicochemical properties of levetiracetam. The obtained information from literature is summarized in **Error! Reference source not found.**. **Error! Reference source not found.** shows the parameters that were additionally used for the lactation PBPK model.

Table S1 Physicochemical parameters used as input for the levetiracetam PBPK models

Parameter	Value	Unit	Description	Source
MW	170.21	g/mol	Molecular weight	Drugbank [9]
pK_a	- (neutral)	-	Acid dissociation constant	-
Solubility (pH 7)	1040.00	mg/mL	Aqueous solubility	Drugbank [9]
Log P	-0.60	-	Log_{10} of the partition coefficient between octanol and water (~lipophilicity)	Drugbank [9]
f_u	0.90	-	Fraction unbound in human plasma	Drugbank [9]
**Kidney plasma clearance** – plasma clearance	0.60	mL/min/kg	Renal clearance	[7]
**In vitro clearance** – Michaelis-Menten – CES1<br>- Km<br>- Vmax	 <br><br><br>- 435<br>- 129	 <br><br><br>- µmol/L<br>- pmol/ml/min	Metabolism	[6]

Table S2 Physicochemical parameters used as input for the lactation PBPK model of levetiracetam

Parameter	Value	Unit	Description	Source
Milk logP[a]	-0.60	-	Log_{10} of the partition coefficient between octanol and water	Drugbank [9]
HBD	1.00	-	Hydrogen bond donors	Pubchem
PSA	63.40	Å^2	Polar surface area	Pubchem

[a] Milk logP is Log_{10} of the partition coefficient between octanol and water and is used as input for the calculations in the postpartum model (see equations below). In theory, this value is identical to the logP specified in Table S1. However, in some PBPK models, logP (Table S1) might be optimized using parameter identication. Therefore, it was chosen to use a separate

parameter (i.e. Milk logP) to represent the logP used as input for the equations in the postpartum model.

The default equations for free fraction in human milk and logD that were implemented in the spatial structure building block that was developed for the postpartum women are described below. Alternatively, these values can be overwritten by values calculated elsewhere (e.g. MarvinSketch) or determined *in vitro.*

The free fraction in human milk was calculated with the equations proposed by Atkinson and Begg [10], as follows:

$$f_{u\_skimmed\ milk} = \frac{f_u \times 0.448}{(0.000694^{0.448} + \mathrm{f}_{\mathrm{u}}^{0.448})}$$

$$P_{milk} = 10^{(-0.88+1.29\times \mathrm{logD7.2})}$$

$$\text{Total free fraction in milk} = \frac{1}{(\frac{0.955}{f_{u\_skimmed\ milk}} + 0.045 \times P_{milk})}$$

Where: f_u *skimmed milk:* binding to proteins in milk; P_{milk}: partitioning between aqueous and lipid phase of milk; Total free fraction in milk: 'total' free fraction, i.e. accounting for both protein and lipid binding processes.

LogD values taking into account up to three pka values (as provided in the compound building block), were calculated as follows:

$$LogD = \mathrm{Log}\,P + Log_{10}(logD_{factor})$$

With Milk logP (Table S2) as input for logP

$$LogD_{factor} = \mathrm{K_1} + (\mathrm{K_2} + \mathrm{K_3} + \mathrm{K_4}) \times \mathrm{base}^1 + \mathrm{K_5} \times \mathrm{base}^{\max(\mathrm{CT_0}+\mathrm{CT_1};-\mathrm{CT_0}-\mathrm{CT_1})} + \mathrm{K_6} \times \mathrm{base}^{\max(CT_0+CT_2;-CT_0-CT_2)} + \mathrm{K_7} \times \mathrm{base}^{\max(\mathrm{CT_2}+\mathrm{CT_1};-\mathrm{CT_2}-\mathrm{CT_1})} + \mathrm{K_8} \times \mathrm{base}^{\max(\mathrm{CT_0}+\mathrm{CT_1}+\mathrm{CT_2};-\mathrm{CT_0}-\mathrm{CT_1}-\mathrm{CT_2})}$$

$$K_1 = F_1 \times F_2 \times F_3$$
$$K_2 = (1 - F_1) \times F_2 \times F_3$$
$$K_3 = F_1 \times (1 - F_2) \times F_3$$
$$K_4 = F_1 \times F_2 \times (1 - F_3)$$
$$K_5 = (1 - F_1) \times (1 - F_2) \times F_3$$
$$K_6 = (1 - F_1) \times F_2 \times (1 - F_3)$$
$$K_7 = (1 - F_1) \times F_2 \times (1 - F_3)$$
$$K_8 = (1 - F_1) \times (1 - F_2) \times (1 - F_3)$$

```
F1 = CT0 != CT_NEUTRAL ?  1/(1+10^(CT0*(pKa_0- pH))) : 1
F2 = CT1 != CT_NEUTRAL ?  1/(1+10^(CT1*(pKa_1- pH))) : 1
F3 = CT2 != CT_NEUTRAL ?  1/(1+10^(CT2*(pKa_2- pH))) : 1
```

With CT = compound type (-1: acid; +1: base; 0: neutral), and pH = 7.2 or 7.4 respectively for $logD_{7.2}$ *and* $logD_{7.4}$

The transports that were added in the passive transport building block for 'transfer to milk' and 'transfer from milk' are based on secretion and reuptake and clearance values, Cl_{sec} and Cl_{re}, which were calculated according to the empirical equations proposed by Koshimich et al. 2011 [8], as follows:

$$\text{Log}\, CL_{re} = 2.793 + 0.179 \times LogP - 0.132 \times HBD$$

$$\text{Log}\, CL_{sec} = 3.367 \times Log10(MW) - 0.164 \times (LogP - LogD) - 0.015 \times PSA - 3.912$$

3.2.2 Clinical data

Literature searches were performed to collect available data on levetiracetam in adults and postpartum women. The levetiracetam reference PBPK model was developed using different clinical studies with pharmacokinetic (PK) blood sampling after single and multiple dose intravenous infusion of levetiracetam [11]. Seven studies with intravenous (IV) and oral (PO) administration of levetiracetam, in a range of 500 – 5000 mg were used for evaluation of the predictive performance of the PBPK model for levetiracetam [6,11–16].

The evaluation of the predictive performance of the levetiracetam lactation PBPK model was performed using 5 different studies where levetiracetam was administered in doses ranging from 1000 to 3500 mg/day to lactating women [17–21]. The women were between 3 days and 22 weeks postpartum. The samples were assumed to be trough samples if the exact timing was not reported in the articles.

Detailed information and data from the studies used for model building, verification, and lactation model can be found in Supplementary material 1 and 2.

3.2.2.1 Model building

The studies that were used for model building are shown in **Error! Reference source not found.** (**training data**).

Table S3 Summary of studies used for PBPK model building of levetiracetam in reference populations

**Study ID**	**Reference**	**Arm/treatment/information used for model building**
Ramael 2006	[11]	18 subjects received 1500 mg IV (single dose)
Ramael 2006	[11]	18 subjects received 1500 mg IV (multiple dose)

Table S4 Demographic information

**Study ID**	**Reference**	**Number of subjects (female ratio)**	**Age (year)**	**Weight (kg)**
Ramael 2006	[11]	18 (0.50)	35 (19-52)	73.3 (50-94)

3.2.2.2 Model verification

The studies that were used to evaluate the predictive performance of the PBPK model are shown in Table S5 (**verification data**).

Table S5 Summary of studies used for model verification of levetiracetam PBPK model in reference population

Study ID	Reference	Arm/treatment/information used for model verification
Benedetti 2003	[2]	4 subjects received 500 mg PO (single dose)
Coupez 2003	[12]	16 subjects received 1500 mg PO (single dose)
Patsalos 2000	[13]	8 subjects received 1000 mg PO (single dose)
Patsalos 2000	[13]	8 subjects received 2000 mg PO (single dose)
Patsalos 2000	[13]	8 subjects received 3500 mg PO (single dose)
Patsalos 2000	[13]	8 subjects received 500 mg PO (single dose)
Patsalos 2000	[13]	8 subjects received 5000 mg PO (single dose)
Ramael 2006	[11]	18 subjects received 1500 mg PO (single dose)
Spencer 2011	[14]	12 subjects received 500 mg IV (multiple dose)
Toublanc 2014	[15]	25 subjects received 1500 mg IV (single dose)
Toublanc 2014	[15]	16 Japanese subjects received 1500 mg IV (multiple dose)
Toublanc 2014	[15]	16 Caucasian subjects received 1500 mg IV (multiple dose)
Toublanc 2014	[15]	25 subjects received 1500 mg PO (single dose)
Zhao 2006	[16]	26 subjects received 500 mg PO (single dose)
Zhao 2006	[16]	26 subjects received 1500 mg PO (single dose)

Table S6 Demographic information

Study ID	Reference	Number of subjects (female ratio)	Age (year)	Weight (kg)
Benedetti 2003	[2]	4 (0)	35.75 (32-51)	80.45 (69.3-88.3)
Coupez 2003	[12]	16 (0.38)	- (22-52)	-
Patsalos 2000	[13]	8 (-)	-	-
Ramael 2006	[11]	18 (5)	35 (19-52)	73.3 (50-94)
Spencer 2011	[14]	12 (0.58)	54 ± 14	89 ± 19
Toublanc 2014	[15]	25 (-)<br>16 (-)<br>16 (-)	29.7 (20-50)<br>29.9 (22-40)<br>27.1 (20-39)	50.27 (45.9-79.7)<br>66.19 (61.2-75.3)<br>72.12 (66.3-80)
Zhao 2006	[16]	26 (0)	24 ± 4.3	64.3 ± 6.8

3.2.2.3 Lactation PBPK model

Table S7 shows the study that was used for the lactation PBPK model.

Table S7 Summary of study used for PBPK model development of levetiracetam in lactating women

Study ID	Publication	Arm/treatment/information used for model building and verification
Dinavitser 2022	[17]	20 women (3-22 weeks postpartum) received PO 2525 (1500-3750) mg/day (multiple dose)
Johannessen 2005	[18]	1 woman (3-5 days postpartum) received PO 1500 mg/day (multiple dose)

Johannessen 2005	[18]	1 woman (3-5 days postpartum) received PO 3500 mg/day (multiple dose)
Johannessen 2005	[18]	1 woman (3-5 days postpartum) received PO 2000 mg/day (multiple dose)
Johannessen 2005	[18]	1 woman (3-5 days postpartum) received PO 2500 mg/day (multiple dose)
Johannessen 2005	[18]	1 woman (3-5 days postpartum) received PO 2000 mg/day (multiple dose)
Johannessen 2005	[18]	1 woman (3-5 days postpartum) received PO 3000 mg/day (multiple dose)
Johannessen 2005	[18]	1 woman (3-5 days postpartum) received PO 2500 mg/day (multiple dose)
Kacirova 2021	[19]	8 woman (7-13 days postpartum) received PO 2000 mg/day (multiple dose)
Tomson 2007	[20]	1 women (23 days postpartum) received PO 3000 mg/day (multiple dose)
Tomson 2007	[20]	1 women (12 days postpartum) received PO 1000 mg/day (multiple dose)
Tomson 2007	[20]	1 women (15 days postpartum) received PO 1000 mg/day (multiple dose)
Tomson 2007	[20]	1 women (12 days postpartum) received PO 2500 mg/day (multiple dose)
Tomson 2007	[20]	1 women (14 days postpartum) received PO 1000 mg/day (multiple dose)
Tomson 2007	[20]	1 women (13 days postpartum) received PO 2500 mg/day (multiple dose)
Tomson 2007	[20]	1 women (21 days postpartum) received PO 2500 mg/day (multiple dose)
Tomson 2007	[20]	1 women (21 days postpartum) received PO 2000 mg/day (multiple dose)
Tomson 2007	[20]	1 women (4 days postpartum) received PO 2000 mg/day (multiple dose)
Tomson 2007	[20]	1 women (10 days postpartum) received PO 2500 mg/day (multiple dose)
Tomson 2007	[20]	1 women (10 days postpartum) received PO 3000 mg/day (multiple dose)
Ylikoyila 2015	[21]	1 women received PO 2000 mg/day (multiple dose)

3.3 Model Parameters and assumptions

3.3.1 Absorption

The release of levetiracetam from the tablet was implemented according to the dissolution profile [22]. A permeability values determined via PAMPA from literature was used for intestinal permeability [23].

2.3.2 Distribution

An important parameter influencing the distribution of a compound is lipophilicity. Lipophilicity was taken from Drugbank. The tissue partition coefficients (Kp) calculation was according to ‘PK-Sim standard’ and the cellular permeability calculation was ‘PK-Sim standard’.

3.3.3 Metabolism and excretion

The final model applies metabolism and kidney plasma clearance. The kidney plasma clearance was found in literature [7]. The exact enzymes involved in the metabolism of levetiracetam are not known, but it is often assumed that esterases in blood and liver are involved. In the PBPK model, we assumed that CES1 is responsible for the metabolism. The relative expression was manually put to 1 in liver and blood cells. Michaelis-Menten kinetics for metabolism of levetiracetam were found in literature [6]. The Vmax was further optimized in parameter identification by fitting to observed data and known fraction metabolized (34%) and fraction excreted in urine (66%) [11].

3.3.4 Secretion to milk

To model the transfer process of levetiracetam into human milk, both the secretion (CL_{sec}) and reuptake clearance (CL_{re}) were calculated using the empirical equations developed by Koshimichi *et al.* 2011 (see **Error! Reference source not found.**) [4].

First, in MoBi®, a spatial structure for the postpartum women was constructed, similar to the workflow from Dallmann *et al.* 2018 [2]. Here, breasts were added as a compartment. In addition, the human milk was connected to the plasma subcompartment of the breasts. The human milk volume was specified as 0.5 L to represent the structure of Koshimichi *et al.* 2011, and a geometric standard deviation of 1.16 was assumed in the population. The free fraction in human milk, and logD values were implemented as the equations described previously. The transfer between plasma and milk was defined as two kinetic processes (transfer to milk and transfer from milk) under passive transports (see below). Next, the simulation was combined with the postpartum population from Job *et al.* 2021 in PK-Sim to account for the postpartum physiology [3].

<u>*Kinetics*</u>

Transfer to milk

$$\frac{dNmilk}{dt} = Cplasma \times f_u \times CL_{sec}$$

where Cplasma is the concentration in plasma (in breast compartment), f_u is the free fraction in plasma and CL_{sec} is the secretion clearance.

Transfer from milk

$$\frac{dNplasma}{dt} = Cmilk \times f_u \times CL_{re}$$

where Cmilk is the concentration in human milk, f_u is the total free fraction in human milk (protein and lipid) and CL_{re} is the reuptake clearance.

The simulated plasma and human milk concentration-time profiles can be used to calculate the M/P ratio as follows:

$$M/P\ ratio\ = \frac{AUC_{milk}}{AUC_{plasma}}$$

3.3.5 Automated parameter optimization

The following table depicts the results of the final parameter optimization according to the different clinical studies.

a) Ramael (2006) 1500 mg IV (single and multiple doses) [11]; fraction excreted to urine (66%); and fraction metabolized (34 %) [7]:

Model parameter	Optimized value	Unit
**In vitro clearance** – Michaelis-Menten – CES1 -Vmax	3751.41	pmol/mL/min

3.4. Infant dosage calculation

Infant dosage via human milk was then calculated based on the predicted (average and maximal) steady-state levetiracetam concentration in human milk, as well as the daily milk intake volume. The daily infant dosage was then compared to the maternal dosage, resulting in the relative infant dose (RID).

$$Daily\ infant\ dosage = C_{average} * 150\ \frac{mL}{kg\ .day}$$

$$Daily\ infant\ dosage = C_{max} * 150\ \frac{mL}{kg\ .day}$$

$$Relative\ infant\ dose\ (RID) = \frac{Infant\ dosage}{Maternal\ dosage} * 100\ \%$$

4. Results

Both the reference and postpartum PBPK model of levetiracetam was developed and verified with clinical PK data.

The models were evaluated covering studies including in particular:

- Intravenous and oral administration
- Single and multiple doses
- A dose range from 500 up to 5000 mg
- Different ethnicities

The model describes the elimination of levetiracetam via CES1 and kidney plasma clearance. Moreover, secretion and reuptake to human milk were described by CL_{sec} and CL_{re}.

The next sections show:

- The final model parameters for the building blocks: section 4.1
- The overall predictive performance: section 4.2
- The simulated versus observed concentration-time profiles for the clinical studies used for model building and for model verification: section 4.3

4.1 Final input parameters

The compound values of the final postpartum PBPK model for levetiracetam are illustrated below.

Physicochemical parameters

Parameter	Value	Unit	Source
MW	170.21	g/mol	Drugbank
pKa	- (neutral)	-	-
Solubility	1040.00	mg/mL	Drugbank
Lipophilicity	-0.60	-	Drugbank
f_u	0.90	-	Drugbank
Small molecule (Y/N)	Yes	-	-
Plasma protein binding partner	Albumin	-	-

Calculation methods

Name	Value
Tissue partition coefficients	PK-Sim standard
Cellular permeabilities	PK-Sim standard

AMDE-related parameters

Parameter	Value	Unit	Source
**In vitro clearance** – Michaelis-Menten – CES1:<br>- Km<br>- Vmax	 <br><br>- 435<br>- 3751.41	 <br><br>- µmol/L<br>- pmol/mL/min	 <br><br>[6]<br>Parameter identification
**Relative expression** CES1:<br>- Blood cells<br>- Liver	 <br>- 1.00<br>- 1.00	-	Assumption
Specific intestinal permeability	2.47E-5	cm/s	[23]

Formulation-related parameters

Type: Solution

Type: tablet formulation

Time (min)	Levetiracetam (%)
0	0
5	35
10	63
15	87
20	93
30	100
40	100

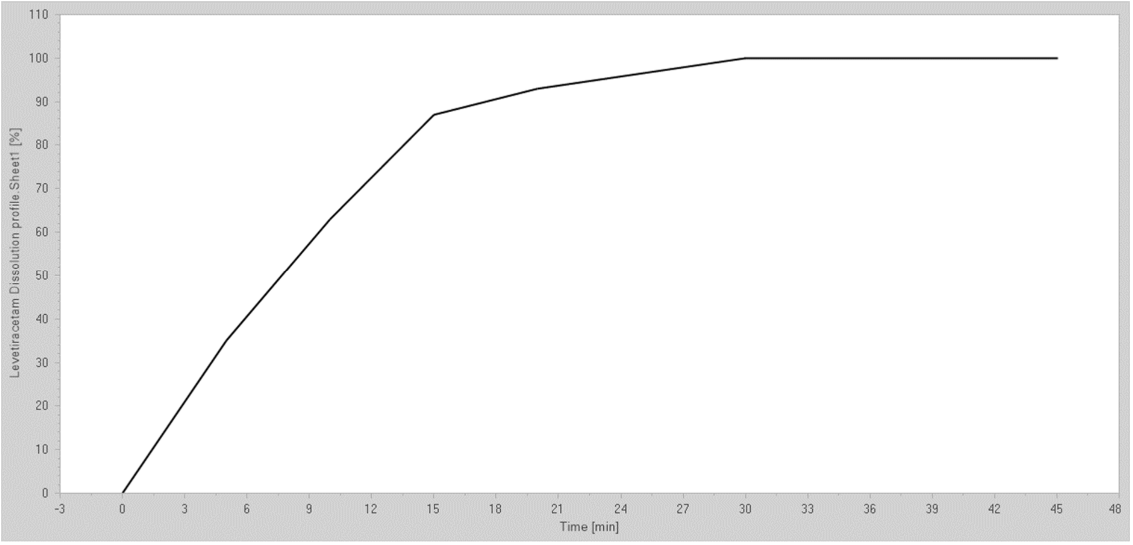

Figure S3 In vitro dissolution profile of levetiracetam for the tablet formulation

Physicochemical and physiological parameters relevant to the lactation model

Parameter	Value	Unit	Source
Milk log P	-0.60	-	Drugbank
HBD	1.00	-	Pubchem
PSA	63.40	Å^2	Pubchem
CL_{sec}	7.42E-3	L/min	Default
CL_{re}	5.96E-3	L/min	Default
*fu*_skimmed milk[a]	0.96	-	Default
Pmilk[b]	0.02	-	Default
Total free fraction in milk[c]	1.01	-	Default
$logD_{7.2}$	-0.60	-	Default
$logD_{7.4}$	-0.60	-	Default

[a] binding to proteins in milk; [b] partitioning between aqueous and lipid phase of milk; [c] total free fraction, accounting for both protein and lipid binding

4.2 Diagnostic plots

The geometric mean fold errors (GMFE) on AUC and Cmax were 1.15 and 1.23 for the model building dataset, and 1.13 and 1.14 for the model verification dataset.
The following shows the predictive performance graph for Cmax and AUC of levetiracetam for the PBPK model performance of all data used.
Predicted over observed ratio values of all data listed in section 3.2.2 are presented below.

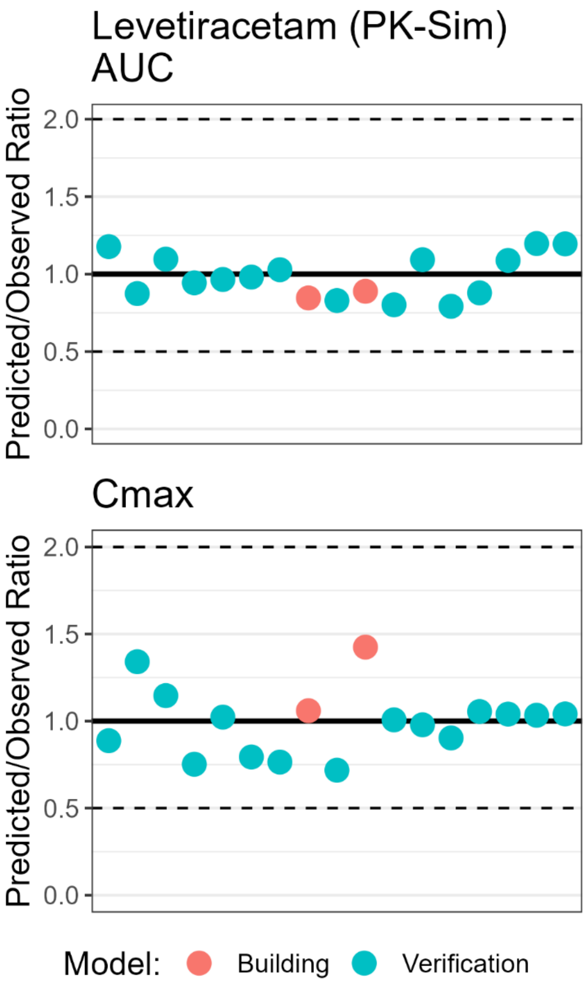

Figure S4. Predicted over observed ratio profile

Table S8 Ratio between the predicted and observed pharmacokinetic parameters of levetiracetam in different dosing regimens for model building

Study ID/ Reference	Dose/ Route	AUC_{obs} (mg*h/L)	AUC_{pred} (mg*h/L)	Fold error	$Cmax_{obs}$ (mg/L)	$Cmax_{pred}$ (mg/L)	Fold error
Ramael 2006 [11]	1500 mg IV SD	384.36	341.70	0.89	40.73	58.03	1.42
Ramael 2006 [11]	1500 mg IV MD	442.56	374.39	0.85	71.63	75.89	1.06

Table S9 Ratio between the predicted and observed pharmacokinetic parameters of levetiracetam in different dosing regimens used for model verification

Study ID/ Reference	Dose/ Route	AUC_{obs} (mg*h/L)	AUC_{pred} (mg*h/L)	Fold error	$Cmax_{obs}$ (mg/L)	$Cmax_{pred}$ (mg/L)	Fold error
Benedetti 2003 [2]	500 mg PO SD	112.60	106.28	0.94	14.52	10.92	0.75
Coupez 2003 [12]	1500 mg PO SD	392.19	344.67	0.88	33.63	35.47	1.05
Patsalos 2000 [13]	1000 mg PO SD	177.75	171.58	0.97	23.24	23.77	1.02

Patsalos 2000 [13]	2000 mg PO SD	388.86	423.19	1.09	45.97	47.81	1.04
Patsalos 2000 [13]	3500 mg PO SD	729.55	873.47	1.20	81.34	84.06	1.03
Patsalos 2000 [13]	500 mg PO SD	97.17	84.96	0.87	8.83	11.84	1.34
Patsalos 2000 [13]	5000 mg PO SD	1073.93	1283.47	1.20	115.70	120.44	1.04
Ramael 2006 [11]	1500 mg PO SD	389.15	308.28	0.79	39.64	35.80	0.90
Spencer 2011 [14]	500 mg IV MD	114.21	134.49	1.18	28.03	24.87	0.89
Toublanc 2014 [15]	1500 mg IV SD	462.72	383.67	0.83	96.69	69.40	0.72
Toublanc 2014 [15]	1500 mg PO SD	473.67	379.76	0.80	41.73	42.02	1.01
Toublanc 2014 [15]	1500 mg IV MD (1)	401.35	393.69	0.98	98.99	78.57	0.79
Toublanc 2014 [15]	1500 mg IV MD (2)	389.97	401.00	1.03	109.07	83.38	0.76
Zhao 2006 [16]	1500 mg PO SD	337.45	368.80	1.09	40.05	39.19	0.98
Zhao 2006 [16]	500 mg PO SD	108.37	118.84	1.10	11.29	12.94	1.15

4.3 Concentration-time profiles

Simulated versus observed concentration-time profiles of all data listed in section 3.2.2 are presented below. The original.pksim5 are provided in Supplemented material 3.

4.3.1 Model building

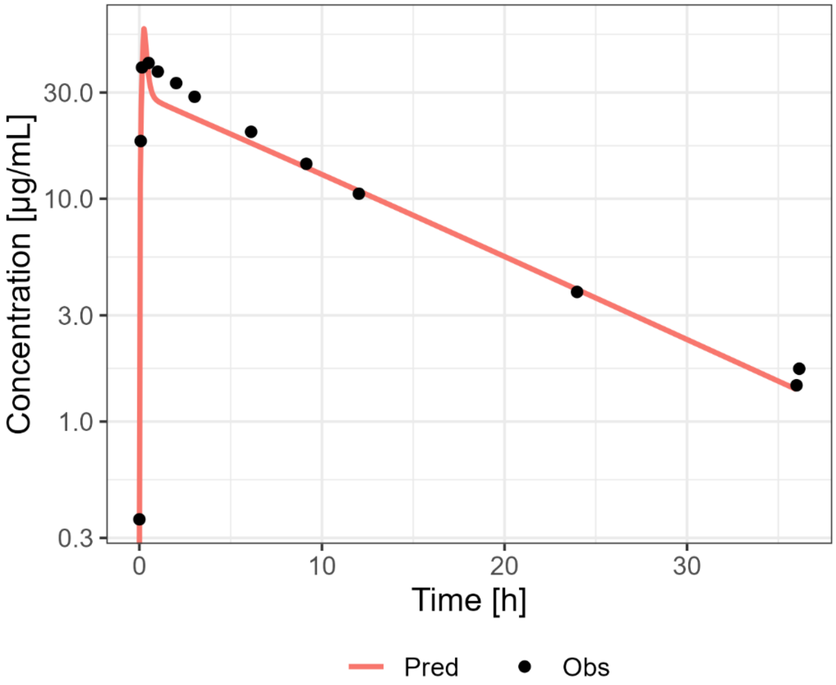

Figure S5 Predicted (Pred) versus observed (Obs) concentration-time profile after administration of 1500 mg IV MD [11]

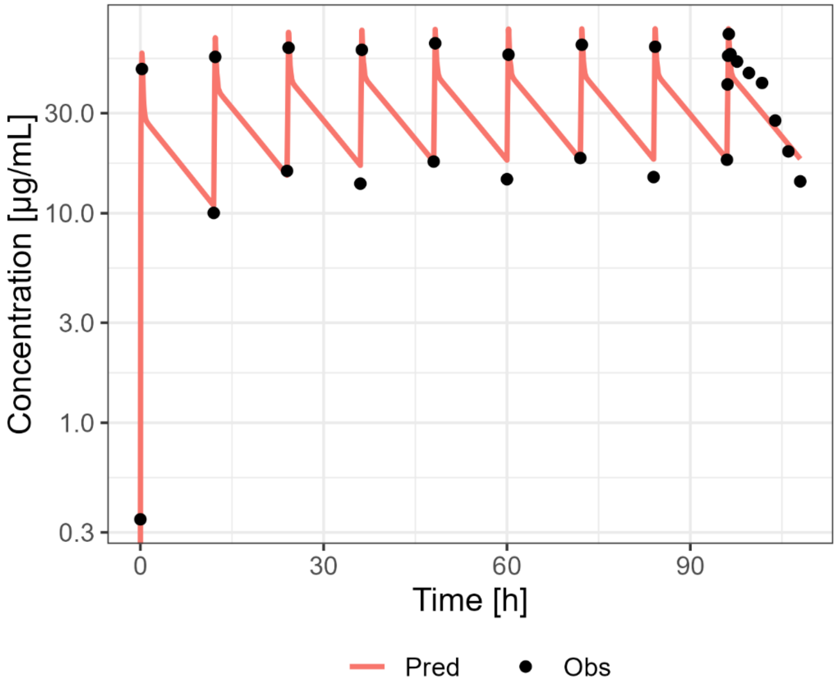

Figure S6 Predicted (Pred) versus observed (Obs) concentration-time profile after administration of 1500 mg IV MD [11]

4.3.2 Model verification

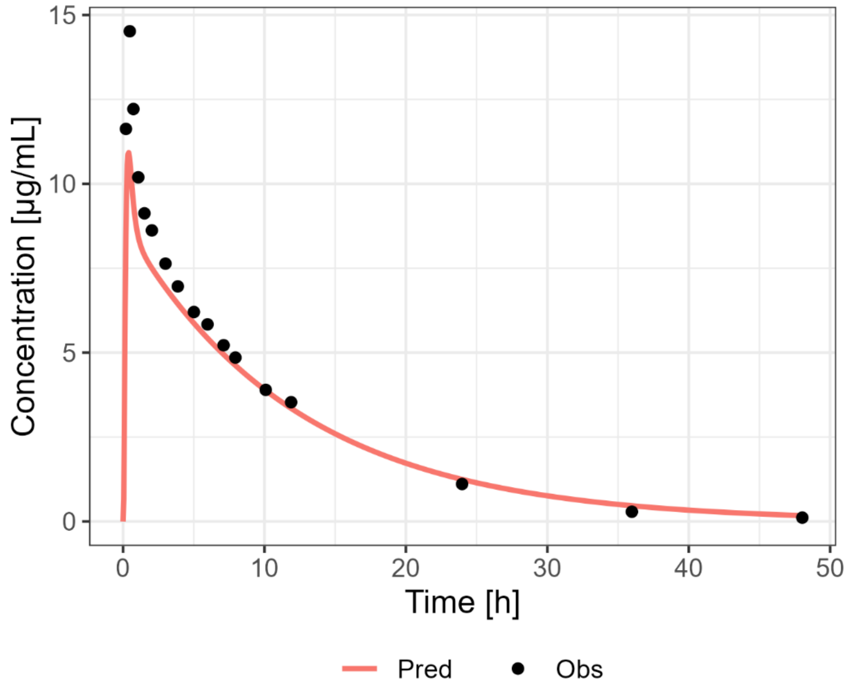

Figure S7 Predicted (Pred) versus observed (Obs) concentration-time profile after administration of 500 mg PO [6]

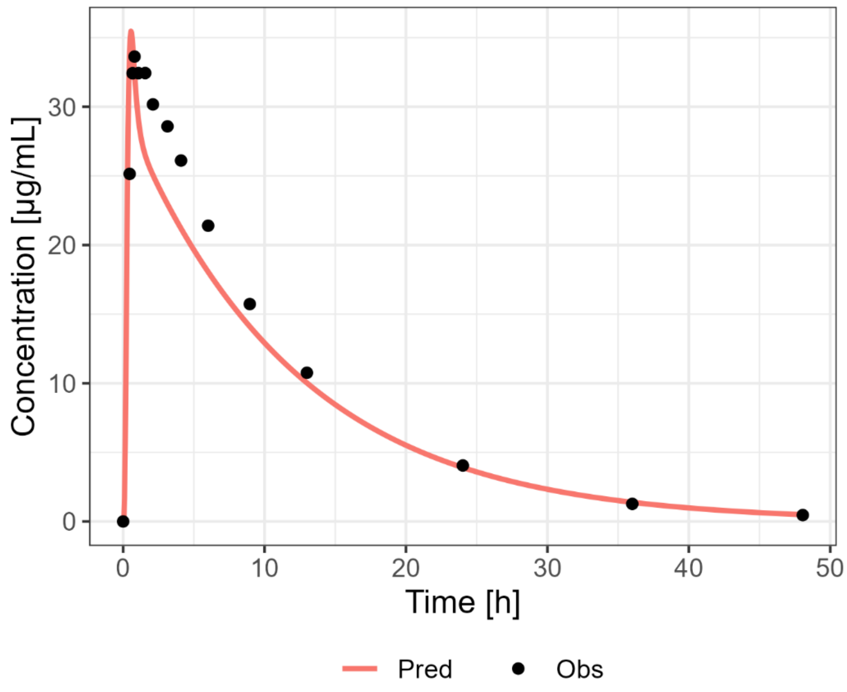

Figure S8 Predicted (Pred) versus observed (Obs) concentration-time profile after administration of 1500 mg PO [12]

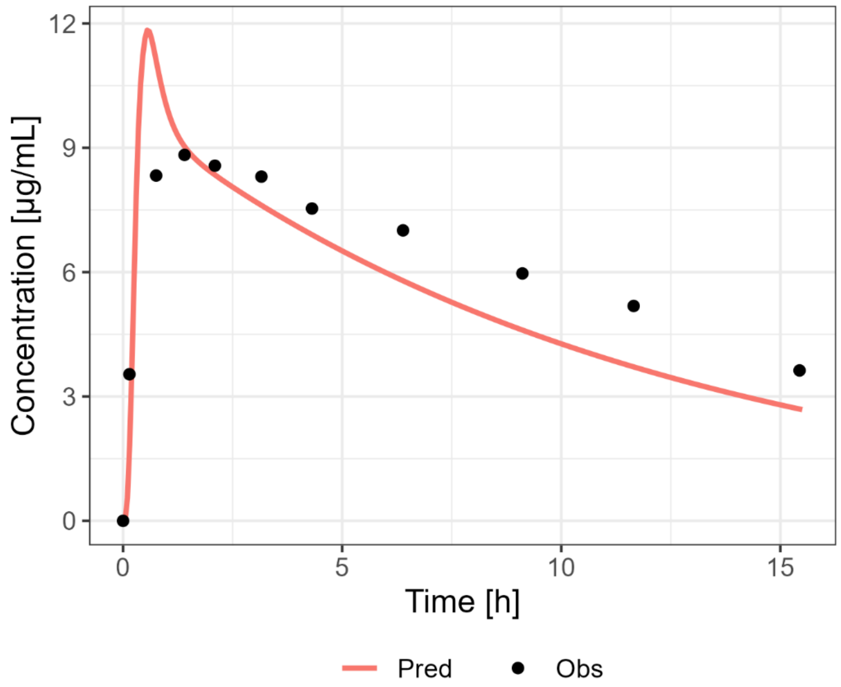

Figure S9 Predicted (Pred) versus observed (Obs) concentration-time profile after administration of 500 mg PO [13]

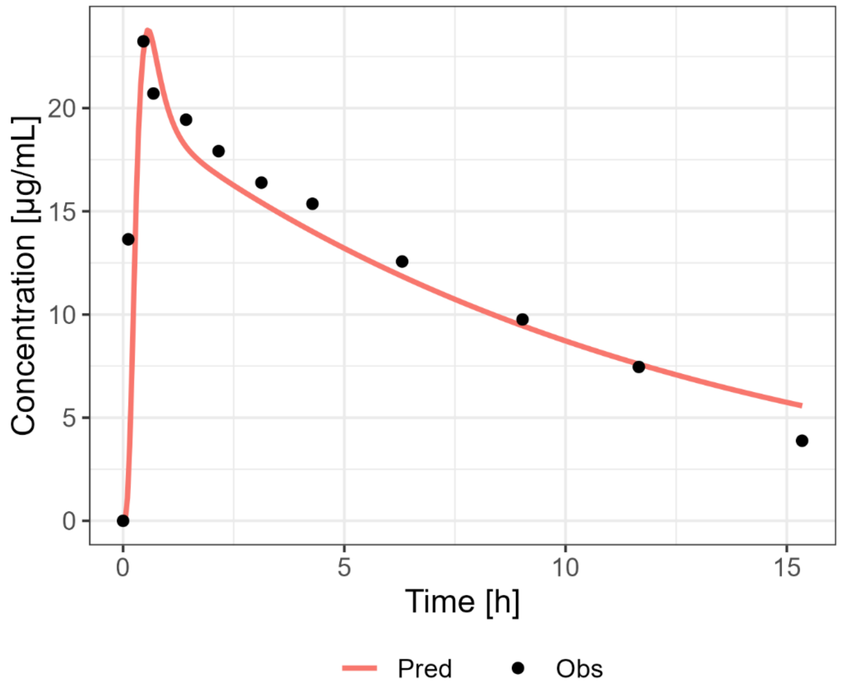

Figure S10 Predicted (Pred) versus observed (Obs) concentration-time profile after administration of 1000 mg PO [13]

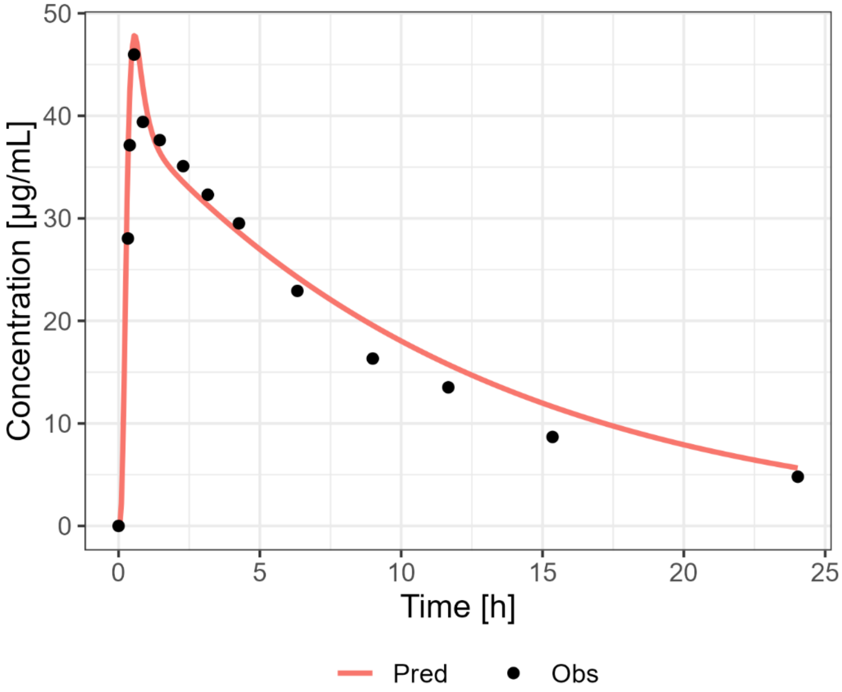

Figure S11 Predicted (Pred) versus observed (Obs) concentration-time profile after administration of 2000 mg PO [13]

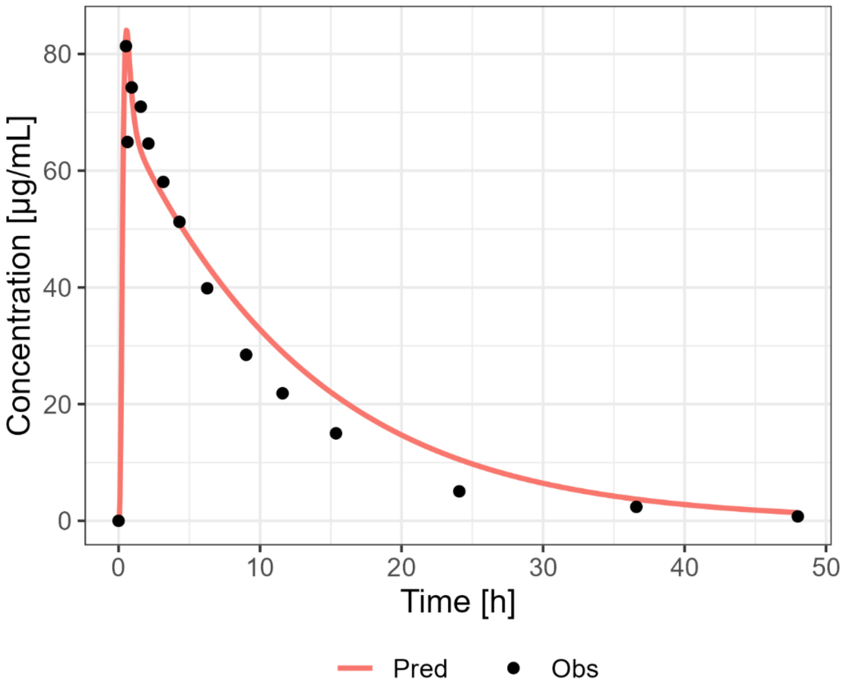

Figure S12 Predicted (Pred) versus observed (Obs) concentration-time profile after administration of 3500 mg PO [13]

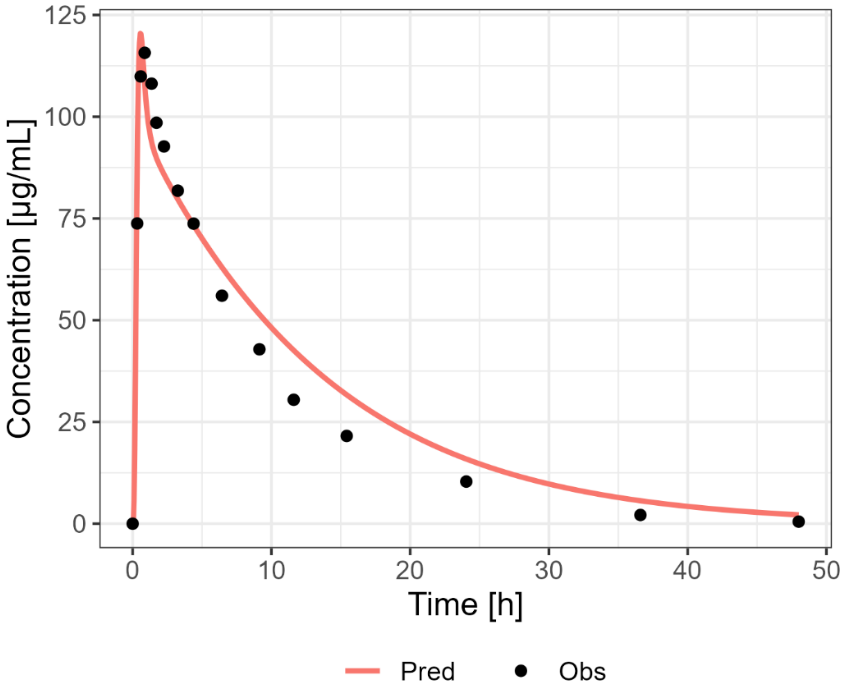

Figure S13 Predicted (Pred) versus observed (Obs) concentration-time profile after administration of 5000 mg PO [13]

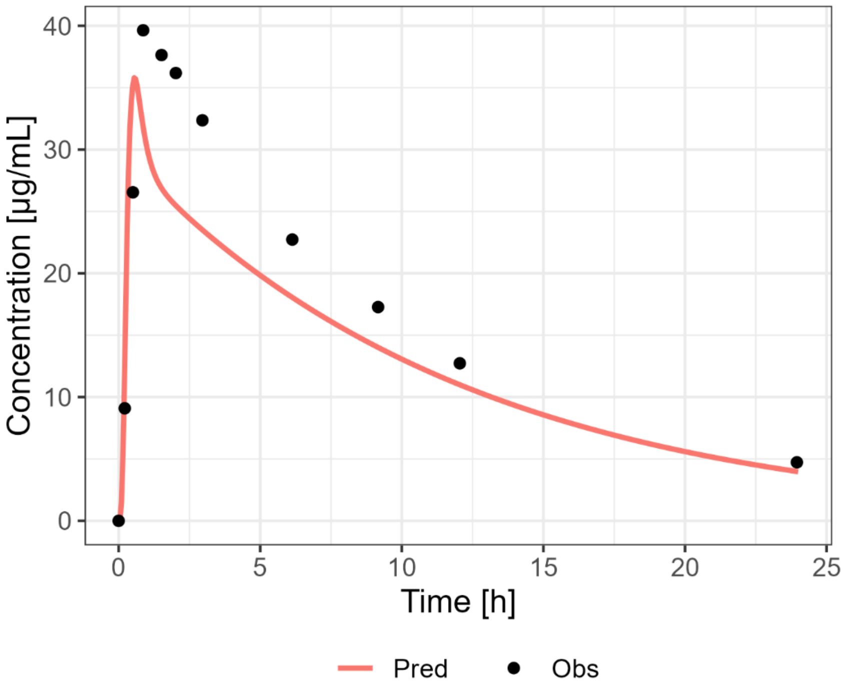

Figure S14 Predicted (Pred) versus observed (Obs) concentration-time profile after administration of 1500 mg PO [11]

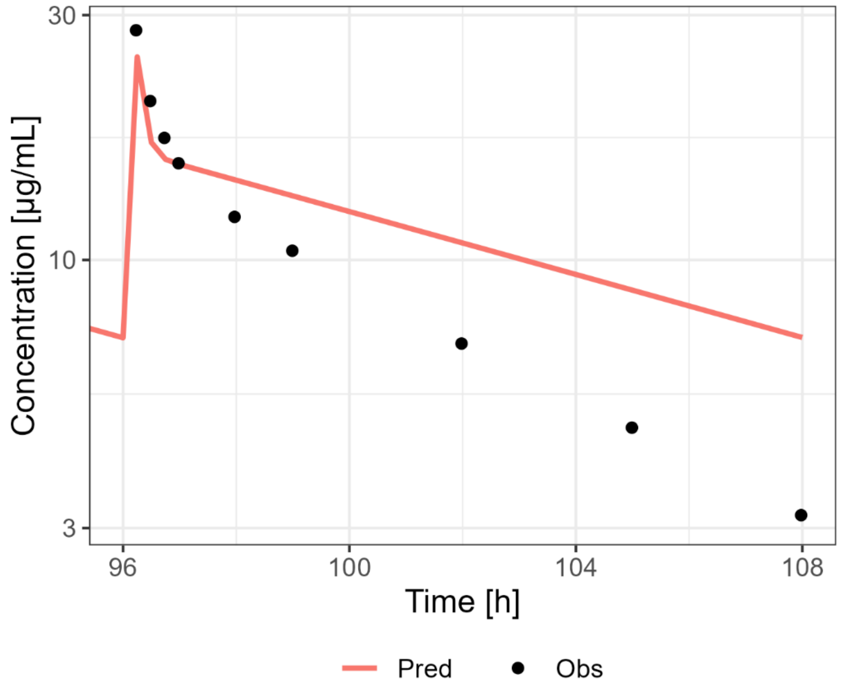

Figure S15 Predicted (Pred) versus observed (Obs) concentration-time profile after administration of 500 mg IV MD [14]

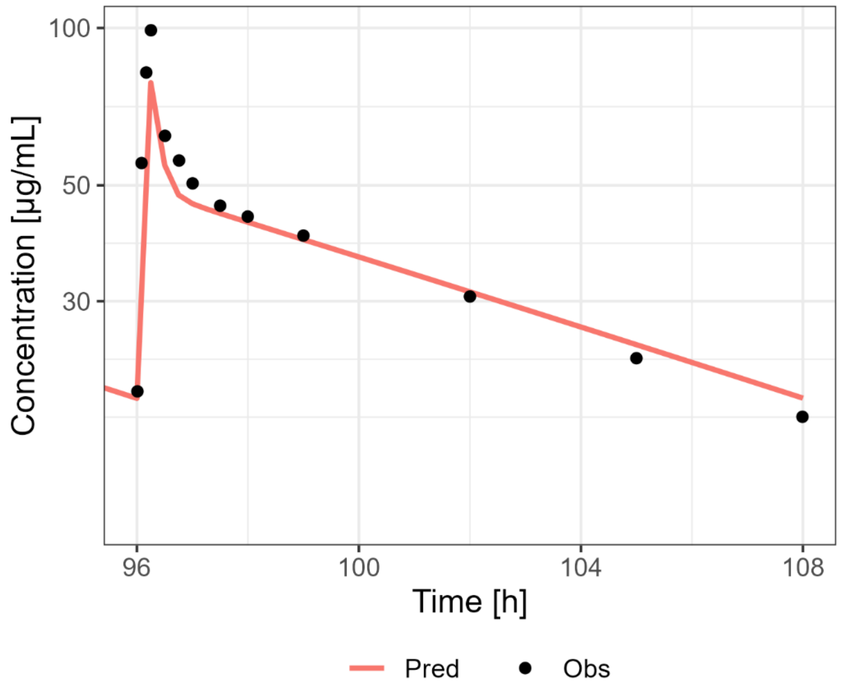

Figure S16 Predicted (Pred) versus observed (Obs) concentration-time profile after administration of 1500 mg IV MD Caucasian [15]

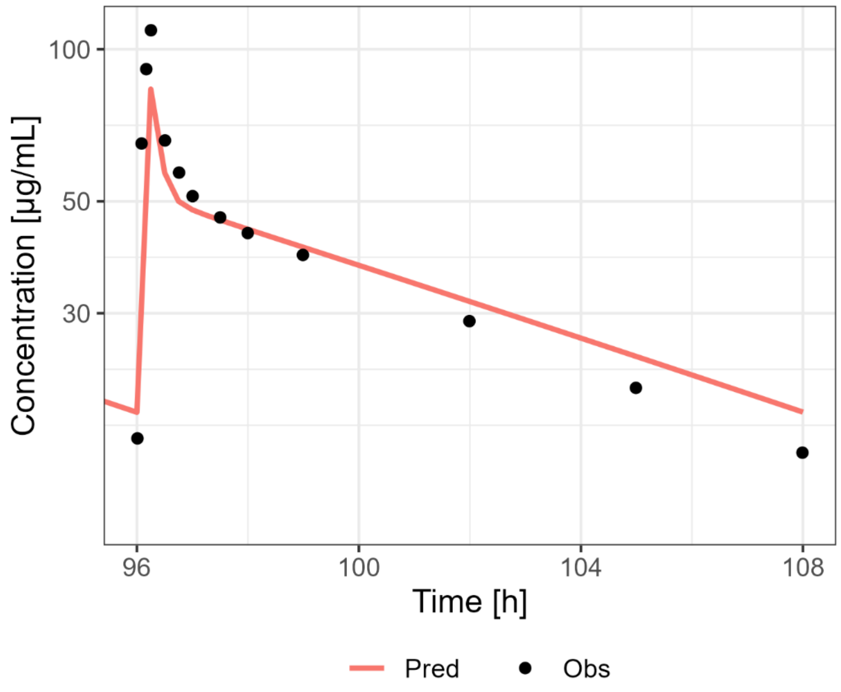

Figure S17 Predicted (Pred) versus observed (Obs) concentration-time profile after administration of 1500 mg IV MD Japanese [15]

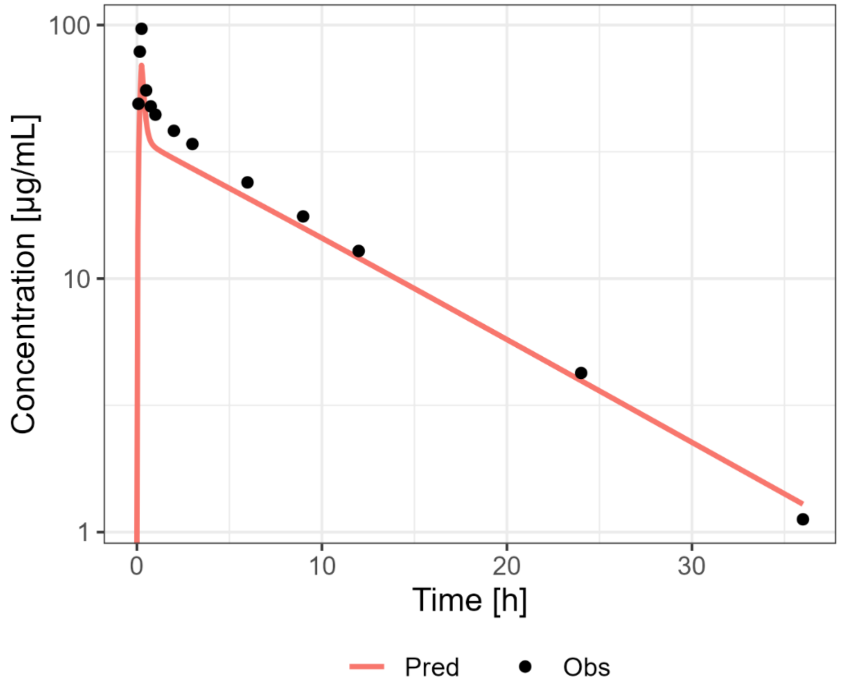

Figure S18 Predicted (Pred) versus observed (Obs) concentration-time profile after administration of 1500 mg IV [15]

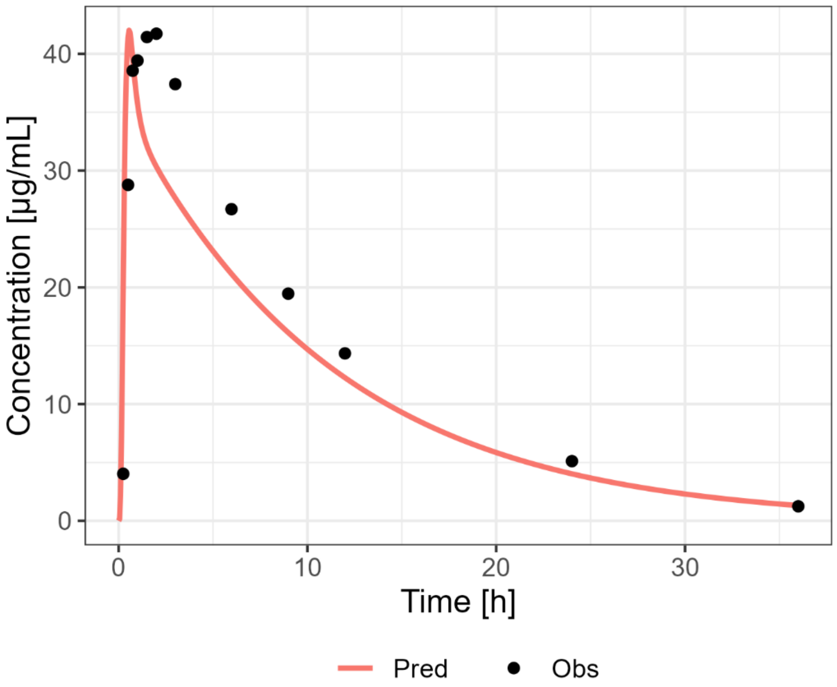

Figure S19 Predicted (Pred) versus observed (Obs) concentration-time profile after administration of 1500 mg PO [15]

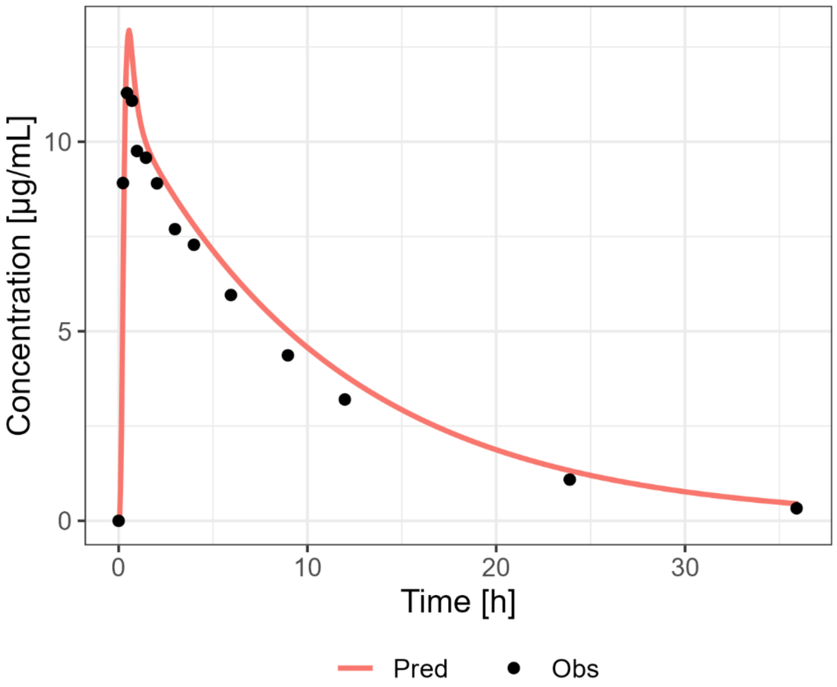

Figure S20 Predicted (Pred) versus observed (Obs) concentration-time profile after administration of 500 mg PO [16]

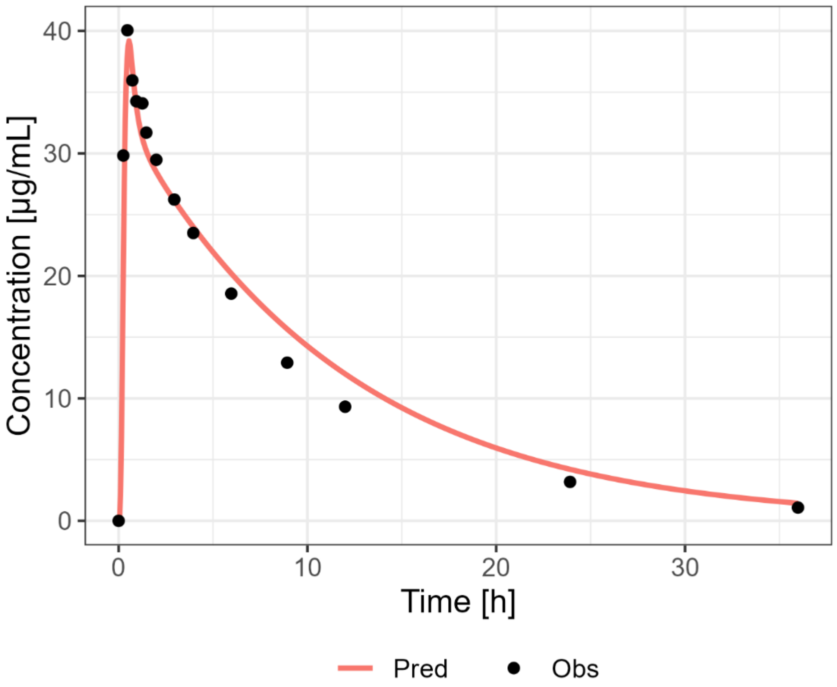

Figure S21 Predicted (Pred) versus observed (Obs) concentration-time profile after administration of 1500 mg PO [16]

4.3.3 Lactation PBPK model

A sample size of 1000 individuals, three months postpartum, was used in each simulation of the virtual lactation population.

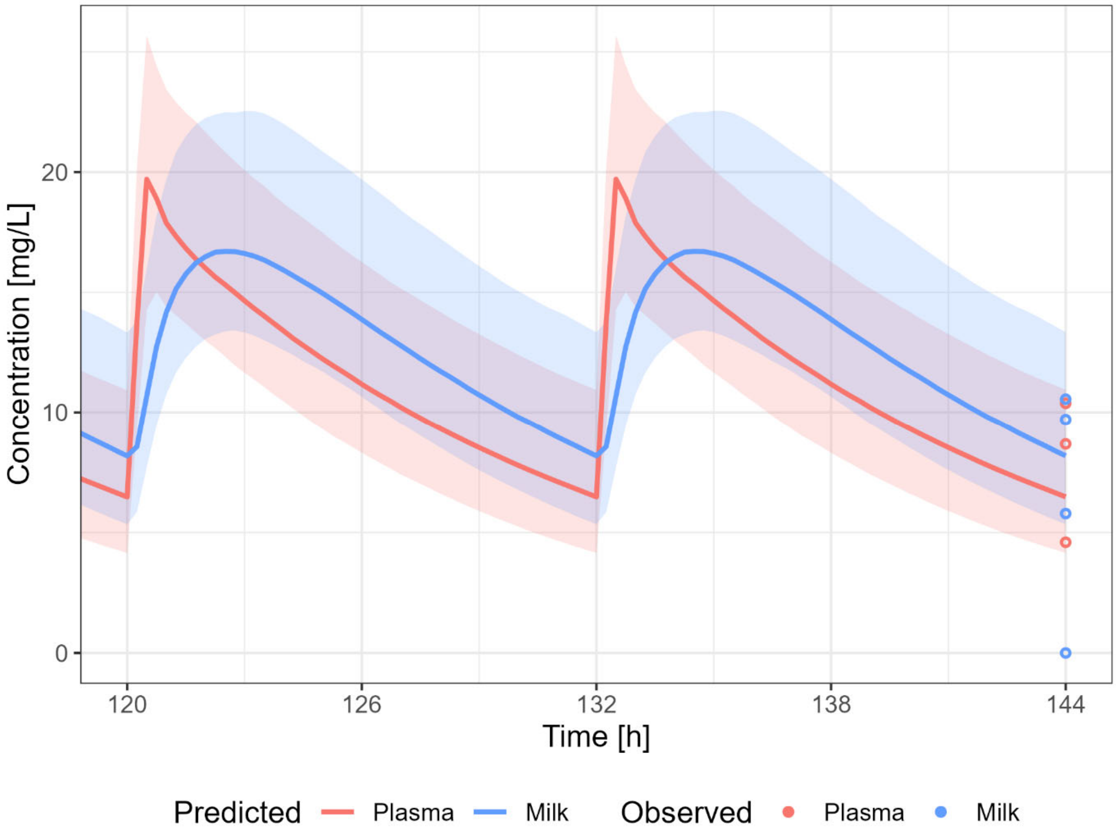

Figure S22 Pedicted (Pred) versus observed (Obs) concentration-time profile after administration of 1000 mg PO MD [20,21]

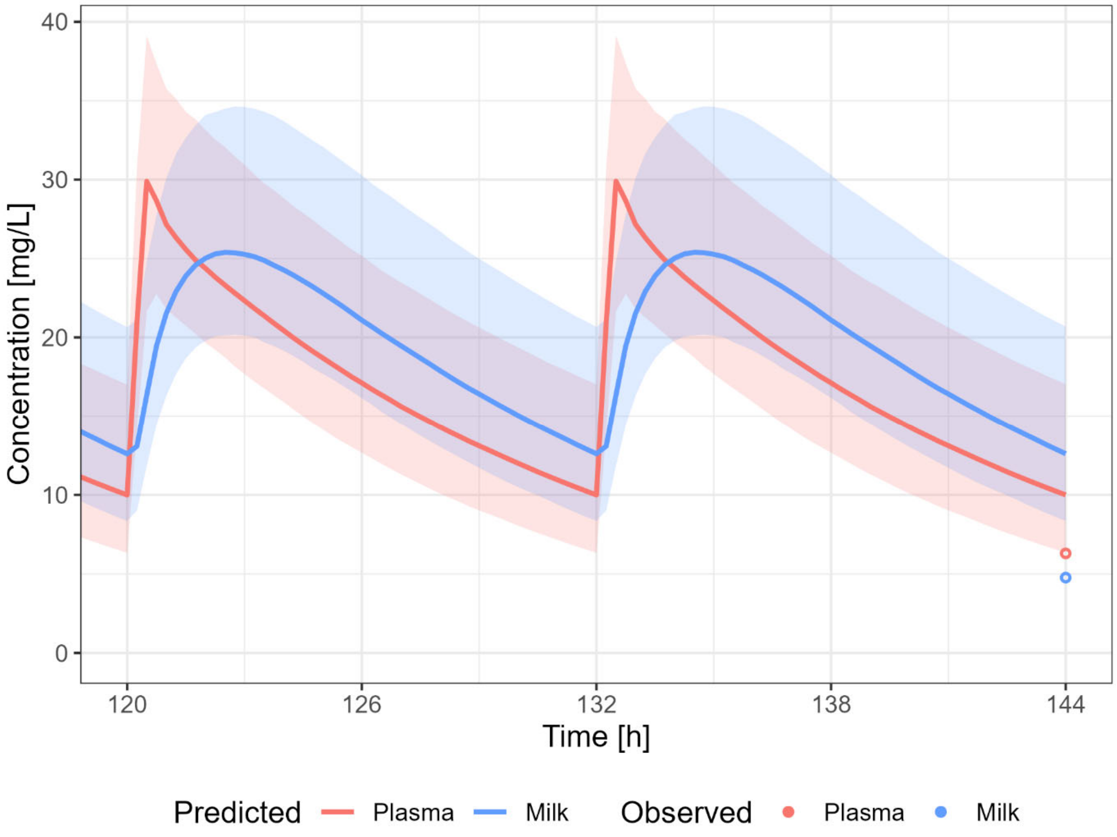

Figure S23 Pedicted (Pred) versus observed (Obs) concentration-time profile after administration of 1500 mg PO MD [18]

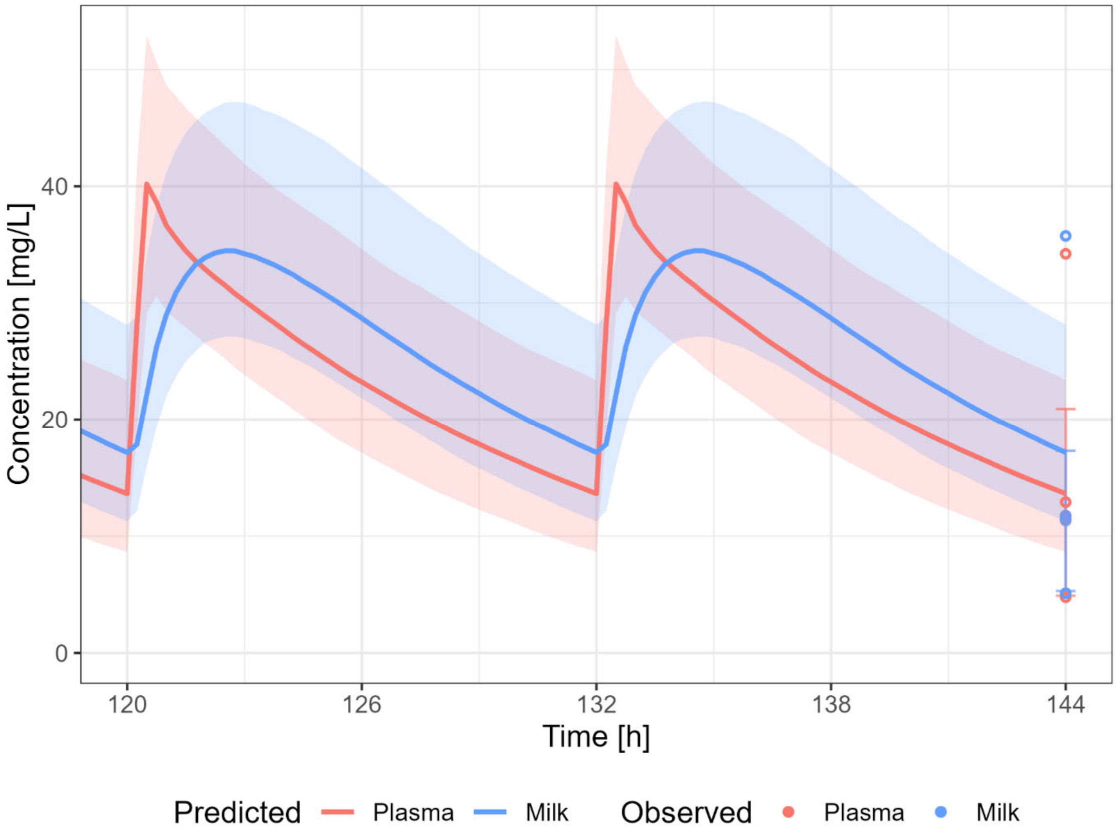

Figure S24 Pedicted (Pred) versus observed (Obs) concentration-time profile after administration of 2000 mg PO MD [18–20]

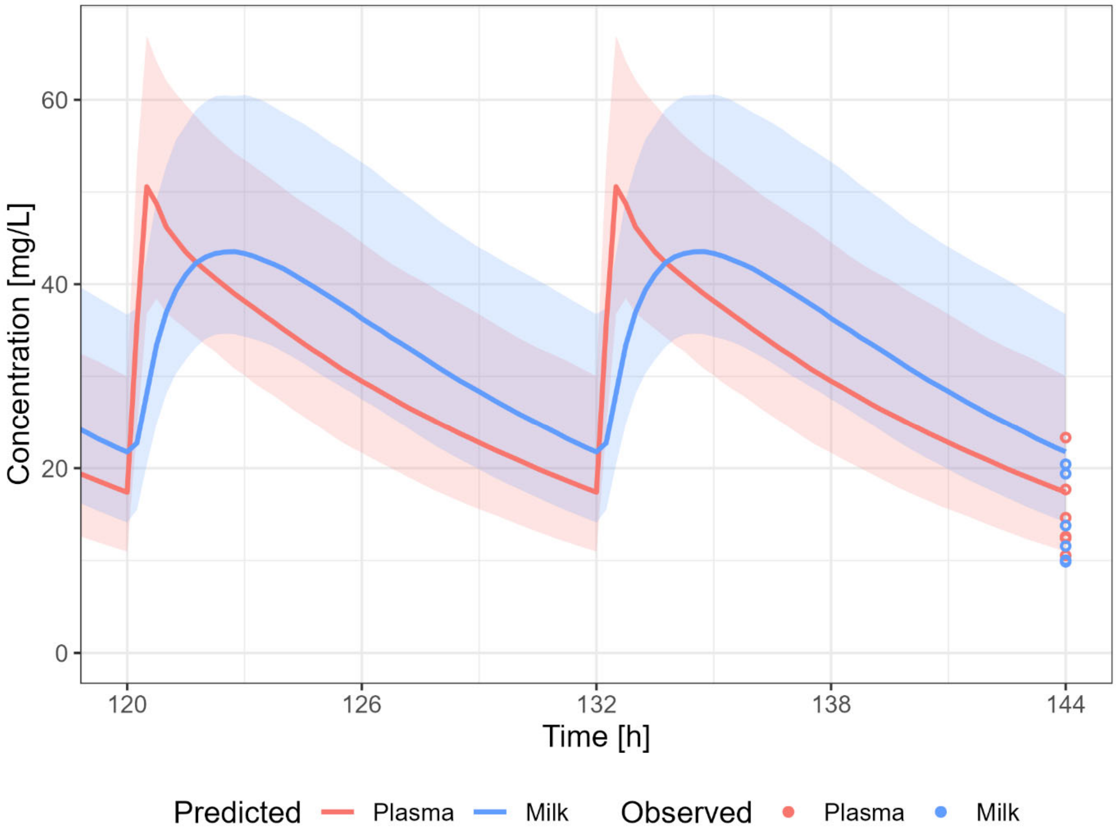

Figure S25 Pedicted (Pred) versus observed (Obs) concentration-time profile after administration of 2500 mg PO MD [18,20]

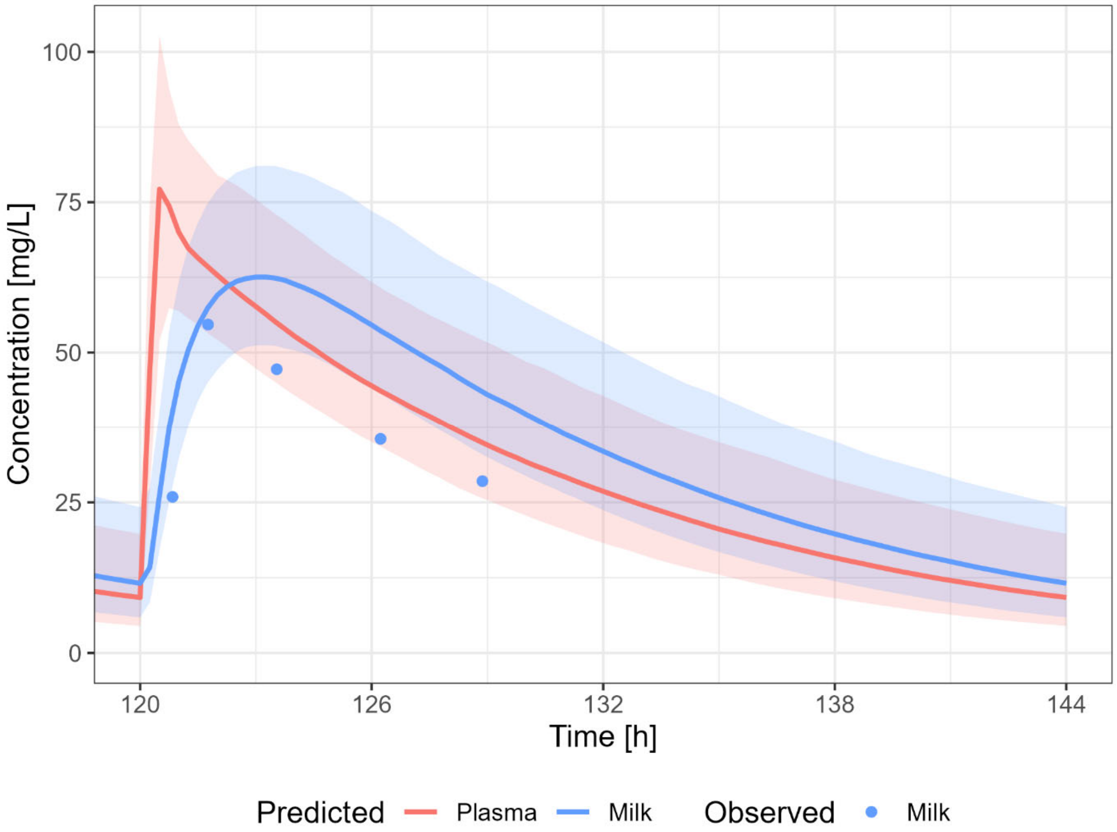

Figure S26 Pedicted (Pred) versus observed (Obs) concentration-time profile after administration of 2525 mg PO MD [17]

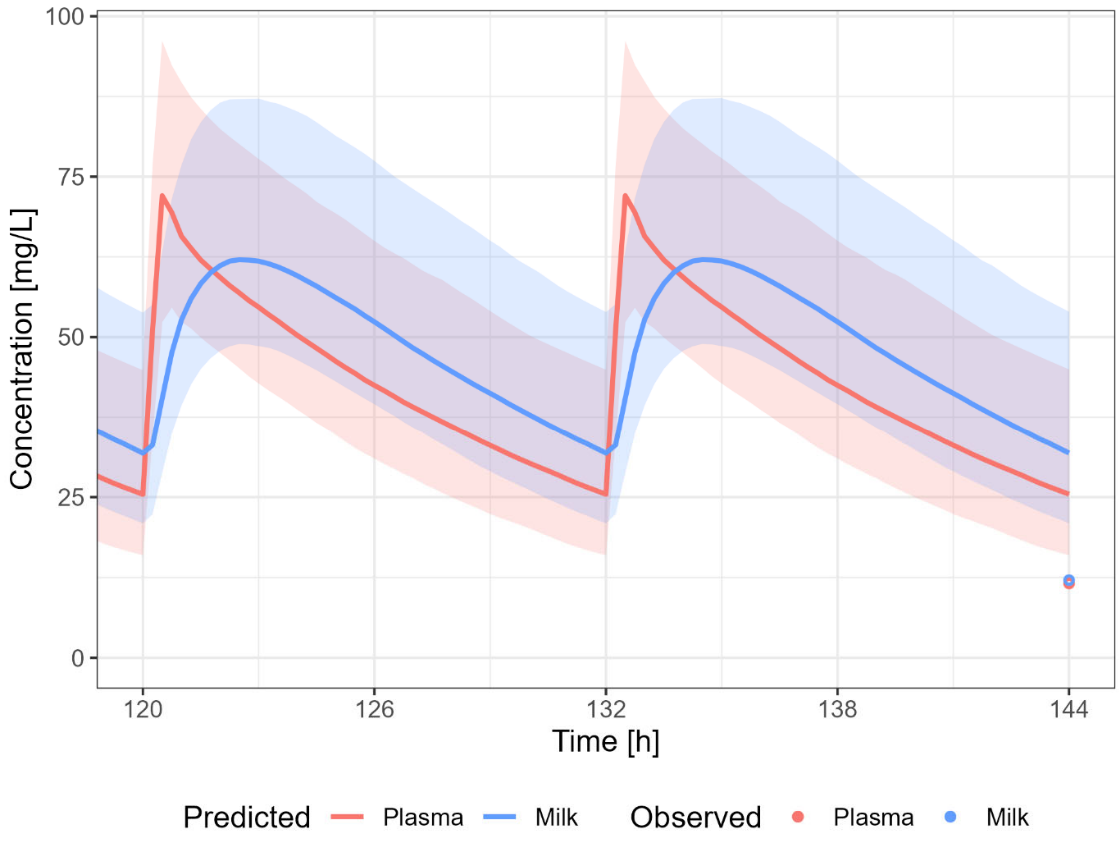

Figure S27 Pedicted (Pred) versus observed (Obs) concentration-time profile after administration of 3500 mg PO MD [18]

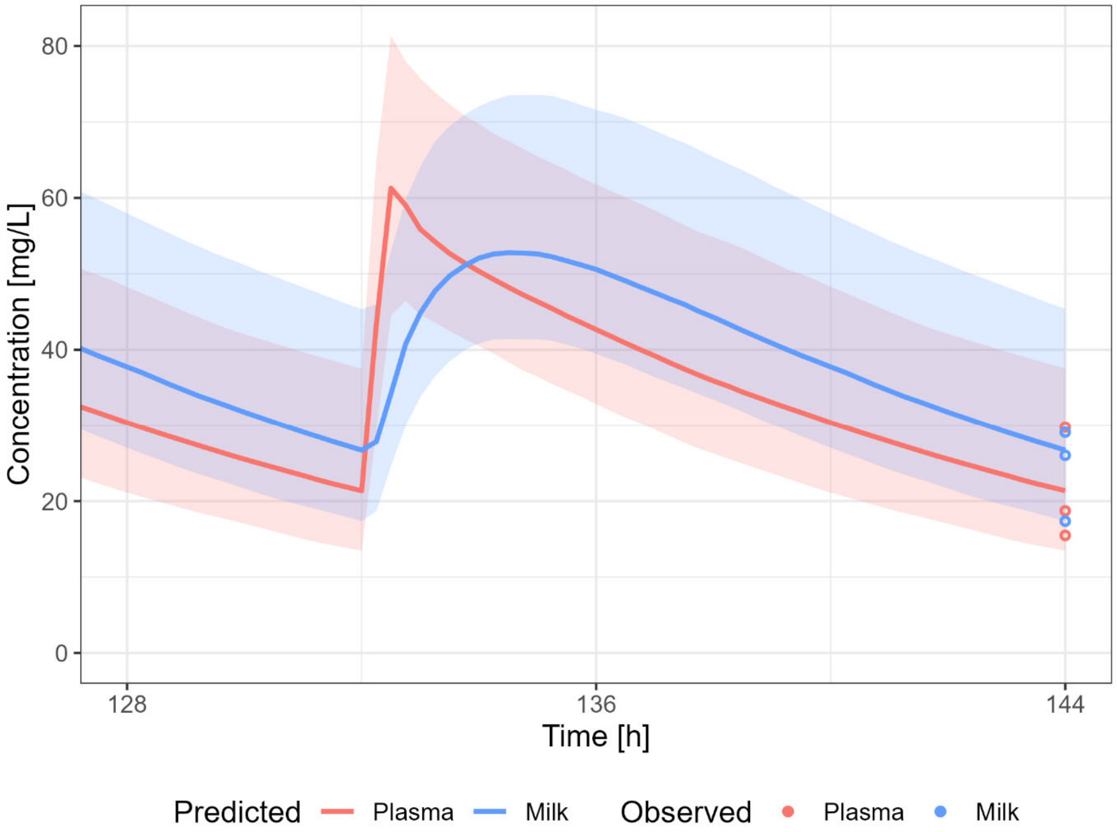

Figure S28 Pedicted (Pred) versus observed (Obs) concentration-time profile after administration of 3000 mg PO MD [18,20]

A dosing regimen of PO 1500 mg bidaily (3000 mg/day) was used to calculate the milk transfer of levetiracetam.

Dosing interval: 12 h	Plasma	Milk
Cmax (mg/L)	61.28	52.78
AUC (mg*h/L)	443.33	492.64
Cave (mg/L)	36.94	41.05

M/P ratio = 1.11

4.4 Estimated Infant exposure

A maternal dosing regimen of 1500 mg, every 12h was assumed to calculate the pediatric exposure. The daily infant dosage and relative infant dose (RID) for 3 months old infants were calculated using a milk intake of 150 mL/kg/day. The daily infant dosage was 6.16 mg/kg/day (RID: 12 %) or 7.92 mg/kg/day (RID: 16 %) based on the average steady-state concentration and maximum concentration in human milk, respectively.

5. Discussion

First, the reference PBPK model was developed and evaluated. Evaluation of the predictive performance showed that the reference PBPK model for levetiracetam was able to capture the pharmacokinetic behavior of the medicines in healthy volunteers and/or patients.

Next, the PBPK model was extended to a lactation PBPK model. The PBPK model results in a reasonable prediction of the human milk concentrations, with most datapoints within the 5-95th percentile of the population prediction.

The predicted M/P ratio (1.11) was within the observed range of M/P ratios.

The calculated infant dosage of levetiracetam via breastfeeding was 12 or 16 % of the maternal daily dosage.

6. Conclusions

The herein presented PBPK model adequately describes the PK of levetiracetam in adults including breastfeeding women. It applies excretion by kidney plasma clearance and CES1 metabolism. The PBPK model was able to predict the human milk concentrations of levetiracetam (M/P ratio: 1.11). The daily infant dosage was 6.16 mg/kg/day (RID: 12 %) or 7.92 mg/kg/day (RID: 16 %) based on the average steady-state concentration and maximum concentration in human milk, respectively.

7. List of Appendix and Supplementary Materials

Supplementary material 1 – ObsDataPK_OSP_reference_levetiracetam
Supplementary material 2 – ObsDataPK_OSP_lactation_levetiracetam
Supplementary material 3 – Levetiracetam.pksim5

8. References

1. Wright, C.; Downing, J.; Mungall, D.; Khan, O.; Williams, A.; Fonkem, E.; Garrett, D.; Aceves, J.; Kirmani, B. Clinical Pharmacology and Pharmacokinetics of Levetiracetam. *Front. Neurol.* **2013**, *4 DEC*, 192, doi:10.3389/fneur.2013.00192 LK - http://limo.libis.be/resolver?&sid=EMBASE&issn=16642295&id=doi:10.3389%2Ffneur.2013.00192&atitle=Clinical+pharmacology+and+pharmacokinetics+of+levetiracetam&stitle=Front.+Neurol.&title=Frontiers+in+Neurology&volume=4+DEC&issue=&spage=&epage=&aulast=Wright&aufirst=Chanin&auinit=C.&aufull=Wright+C.&coden=&isbn=&pages=-&date=2013&auinit1=C&auinitm=.
2. Strolin Benedetti, M.; Whomsley, R.; Nicolas, J.-M.; Young, C.; Baltes, E. Pharmacokinetics and Metabolism of 14C-Levetiracetam, a New Antiepileptic Agent, in Healthy Volunteers. *Eur. J. Clin. Pharmacol.* **2003**, *59*, 621–630, doi:10.1007/s00228-003-0655-6.
3. Dallmann, A.; Solodenko, J.; Ince, I.; Eissing, T. Applied Concepts in PBPK Modeling: How to Extend an Open Systems Pharmacology Model to the Special Population of Pregnant Women. *CPT pharmacometrics Syst. Pharmacol.* **2018**, *7*, 419–431, doi:10.1002/PSP4.12300.
4. Dallmann, A.; Himstedt, A.; Solodenko, J.; Ince, I.; Hempel, G.; Eissing, T. Integration of Physiological Changes during the Postpartum Period into a PBPK Framework and Prediction of Amoxicillin Disposition before and Shortly after Delivery. *J. Pharmacokinet. Pharmacodyn.* **2020**, *47*, 341–359, doi:10.1007/s10928-020-09706-z.
5. Job, K.M.; Dallmann, A.; Parry, S.; Saade, G.; Haas, D.M.; Hughes, B.; Berens, P.; Chen, J.; Fu, C.; Humphrey, K.; et al. Development of a Generic Physiologically-Based Pharmacokinetic Model for Lactation and Prediction of Maternal and Infant Exposure to Ondansetron via Breast Milk. *Clin. Pharmacol. Ther.* **2022**, *111*, 1111–1120, doi:10.1002/cpt.2530.
6. Strolin, M.; Rhys, B.Æ. Pharmacokinetics and Metabolism of 14 C-Levetiracetam , a New Antiepileptic Agent , in Healthy Volunteers. **2003**, 621–630, doi:10.1007/s00228-003-0655-6.
7. Patsalos, P.N. Of Levetiracetam. **2004**, *43*, 707–724.
8. Koshimichi, H.; Ito, K.; Hisaka, A.; Honma, M.; Suzuki, H. Analysis and Prediction of Drug Transfer into Human Milk Taking into Consideration Secretion and Reuptake Clearances across the Mammary Epithelia. *Drug Metab. Dispos.* **2011**, *39*, 2370–2380, doi:10.1124/dmd.111.040972.
9. Wishart, D.S.; Feunang, Y.D.; Guo, A.C.; Lo, E.J.; Marcu, A.; Grant, J.R.; Sajed, T.; Johnson, D.; Li, C.; Sayeeda, Z.; et al. DrugBank 5.0: A Major Update to the DrugBank Database for 2018. *Nucleic Acids Res.* **2017**, *46*, 1074–1082, doi:10.1093/nar/gkx1037.
10. Atkinson, U.C.; Begg, E.J.E.J.; Atkinson, H.C.; Begg, E.J.E.J. Prediction of Drug Distribution into Human Milk from Physicochemical Characteristics. *Clin. Pharmacokinet.* **1990**, *18*, 151–167, doi:10.2165/00003088-199018020-00005.
11. Ramael, S.; De Smedt, F.; Toublanc, N.; Otoul, C.; Boulanger, P.; Riethuisen, J.-M.;

Stockis, A. Single-Dose Bioavailability of Levetiracetam Intravenous Infusion Relative to Oral Tablets and Multiple-Dose Pharmacokinetics and Tolerability of Levetiracetam Intravenous Infusion Compared with Placebo in Healthy Subjects. *Clin. Ther.* **2006**, *28*, 734–744, doi:10.1016/j.clinthera.2006.05.004.

12. Coupez, R.; Nicolas, J.; Browne, T.R. Levetiracetam , a New Antiepileptic Agent : Lack of In Vitro and In Vivo Pharmacokinetic Interaction with Valproic Acid.
13. Patsalos, P.N. Pharmacokinetic Profile of Levetiracetam : Toward Ideal Characteristics. **2000**, *85*, 77–85.
14. Spencer, D.D.; Pharm, D.; Jacobi, J.; Pharm, D.; Fleck, J.D.; Kays, M.B.; Pharm, D.; Pharmacokinetics, L.; Neurocritical, I.N.; Patients, C. Steady-State Pharmacokinetics of Intravenous Levetiracetam in Neurocritical Care Patients. **2011**, *31*, 934–941.
15. Toublanc, N.; Okagaki, T.; Boyce, M.; Chan, R.; Mugitani, A.; Watanabe, S.; Yamamoto, K.; Yoshida, K.; Andreas, J.-O. Pharmacokinetics of the Antiepileptic Drug Levetiracetam in Healthy Japanese and Caucasian Volunteers Following Intravenous Administration. *Eur. J. Drug Metab. Pharmacokinet.* **2015**, *40*, 461–469, doi:10.1007/s13318-014-0227-4.
16. Zhao, Q.; Jiang, J.; Li, X.; Lu, Z.; Hu, P. Single-Dose Pharmacokinetics of Levetiracetam in Healthy Chinese Male Subjects. *Br. J. Clin. Pharmacol.* **2007**, *63*, 614–617, doi:10.1111/j.1365-2125.2006.02782.x LK - http://limo.libis.be/resolver?&sid=EMBASE&issn=03065251&id=doi:10.1111%2Fj.1365-2125.2006.02782.x&atitle=Single-dose+pharmacokinetics+of+levetiracetam+in+healthy+Chinese+male+subjects&stitle=Br.+J.+Clin.+Pharmacol.&title=British+Journal+of+Clinical+Pharmacology&volume=63&issue=5&spage=614&epage=617&aulast=Hu&aufirst=Pei&auinit=P.&aufull=Hu+P.&coden=BCPHB&isbn=&pages=614-617&date=2007&auinit1=P&auinitm=.
17. Dinavitser, N.; Kohn, E.; Berlin, M.; Brandriss, N.; Bar-Chaim, A.; Keidar, R.; Livne, A.; Stepensky, D.; Berkovitch, M.; Sheinberg, R. Levetiracetam in Lactation: How Much Is Excreted into Human Breast Milk? *Br. J. Clin. Pharmacol.* **2022**, *88*, 199–205, doi:10.1111/bcp.14940.
18. Johannessen, S.I.; Helde, G.; Brodtkorb, E. Levetiracetam Concentrations in Serum and in Breast Milk at Birth and during Lactation. *Epilepsia* **2005**, *46*, 775–777, doi:10.1111/j.1528-1167.2005.54804.x.
19. Kacirova, I.; Grundmann, M.; Brozmanova, H. Umbilical Cord, Maternal Milk and Breastfed Infant Levetiracetam Concentrations Monitoring at Delivery and during Early Postpartum Period. *Pharmaceutics* **2021**, *13*, doi:10.3390/pharmaceutics13030398.
20. Tomson, T.; Palm, R.; Källén, K.; Ben-Menachem, E.; Söderfeldt, B.; Danielsson, B.; Johansson, R.; Luef, G.; Öhman, I. Pharmacokinetics of Levetiracetam during Pregnancy, Delivery, in the Neonatal Period, and Lactation. *Epilepsia* **2007**, *48*, 1111–1116, doi:10.1111/j.1528-1167.2007.01032.x.
21. Ylikotila, P.; Ketola, R.A.; Timonen, S.; Malm, H.; Ruuskanen, J.O. Early Pregnancy Cerebral Venous Thrombosis and Status Epilepticus Treated with Levetiracetam and Lacosamide throughout Pregnancy. *Reprod. Toxicol.* **2015**, *57*, 204–206, doi:10.1016/j.reprotox.2015.07.068.
22. Application, E.P. European Patent Application (51). **2012**, *1*, 1–11.
23. Petruševska, M.; Berglez, S.; Krisch, I.; Legen, I.; Megušar, K.; Peternel, L.; Abrahamsson, B.; Cristofoletti, R.; Groot, D.W.; Kopp, S.; et al. Biowaiver Monographs for Immediate Release Solid Oral Dosage Forms: Levetiracetam. *J. Pharm. Sci.* **2015**, *104*, 2676–2687, doi:10.1002/jps.24350.
