# Supplementary material for: Generic Workflow to Predict Medicine Concentrations in Human Milk Using Physiologically-Based Pharmacokinetic (PBPK) Modelling—A Contribution from the ConcePTION Project
Source: Pharmaceutics. 2023 May 11;15(5):1469. doi: 10.3390/pharmaceutics15051469 (PMC10223637; doi:10.3390/pharmaceutics15051469)
Supplement: Supplementary file 1 [file pharmaceutics-15-01469-s001.zip › File S5. Evaluation_report_metformin.pdf]

Building and evaluation of a Physiologically-Based PharmacoKinetic (PBPK) model for **metformin** in adults and lactating women

Disclaimer: The research project leading to these results was conducted as part of the ConcePTION consortium. This report only reflects the personal views of the stated authors. The results of this report are only intended for research purpose, and are not intended to be used in clinical practice.

Glossary

AUC	Area Under the Curve
C_{ave}	Average concentration
CL_{re}	Reuptake clearance (i.e. from milk to blood)
CL_{sec}	Secretion clearance (i.e. from blood to milk)
C_{max}	Maximum (~peak) concentration
DID	Daily Infant Dosage (expressed for instance in mg/kg/day)
f_u	Fraction unbound in plasma
GFR	Glomerular Filtration Rate
HBD	Hydrogen Bond Donors
IV	Intravenous administration
$LogD_{7.2}$	Logarithm of the partition coefficient between an octanol phase and an aqueous (buffer) phase at pH 7.2
$LogD_{7.4}$	Logarithm of the partition coefficient between an octanol phase and an aqueous (buffer) phase at pH 7.4
LogP	Logarithm of the partition coefficient between an octanol phase and (unbuffered) water as aqueous phase. This is the default parameter to express lipophilicity of a substance.
MD	Multiple doses
M/P ratio	Milk-to-Plasma ratio
MW	Molecular Weight (Da)
PBPK	Physiologically-Based PharmacoKinetic [*modeling*]
pKa	Logarithm of the acid dissociation constant
PO	Oral administration
PSA	Polar Surface Area
RID	Relative Infant Dose
SD	Single dose

1. Table of Contents

1. Table of Contents ***3***

2. Introduction ***4***

3. Methods ***5***

3.1 Modelling strategy **5**
3.1.1. Reference PBPK models 6
3.1.2. Lactation model 6

3.2 Data **6**
3.2.1 *In vitro* / physicochemical data 6
3.2.2 Clinical data 9

3.3 Model Parameters and assumptions **9**
3.3.1 Absorption 9
3.3.2 Distribution 9
3.3.3 Metabolism and excretion 9
3.3.4 Secretion to milk 10

3.4. Infant dosage calculation **10**

4. Results ***11***

4.1 Final input parameters **11**

4.2 Diagnostic plots **13**

4.3 Concentration-time profiles **16**
4.3.1 Model building 16
4.3.2 Model verification 22
4.3.3 Lactation PBPK model 35

4.4 Estimated Pediatric exposure **39**

5. Discussion ***39***

6. Conclusion ***40***

7. List of Appendix and Supplementary Materials ***41***

8. References ***41***

2. Introduction

Metformin (Figure S1) is an oral antidiabetic that is used in first-line therapy for the treatment of type 2 diabetes mellitus [1]. The starting doses are 500 mg twice a day or 850 mg once a day. The dose can be increased to a maximum of 2550 mg a day based on glycemic control and tolerability (Drug label Glucophage). Metformin absorption occurs at the upper intestine, the bioavailability is 50-60 %. The average volume of distribution after 0.25 g metformin HCl IV administration in four healthy male subjects was 276 L [2]. Metformin is not eliminated by metabolism, but is excreted unchanged in urine and feces. It is a substrate of OCT1&2 (Organic Cation Transporter), MATE1 (Multidrug and Toxin Extrusion Protein) and PMAT (Plasma Membrane Monoamine Transporter) [1].

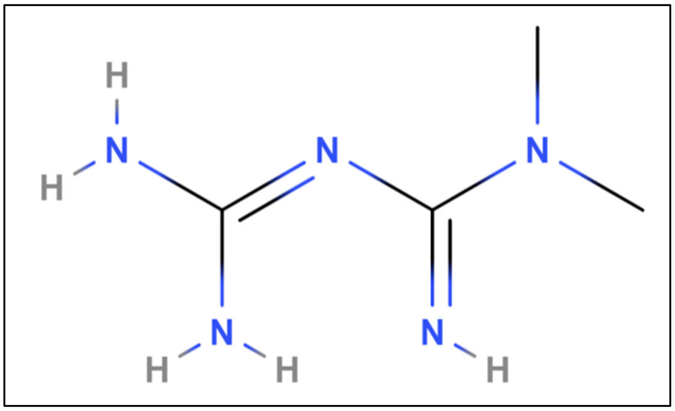

Figure S1 Chemical Structure of metformin

The scope of this report is to:

(a) specify the details and underlying assumptions associated with the building of physiologically-based pharmacokinetic (PBPK) models for metformin in adult healthy volunteers or patients, and in postpartum women during lactation.

(b) evaluate the predictive performance of these PBPK models. This is achieved by comparing model-predicted plasma or milk concentrations with corresponding clinical observations.

3. Methods

The software used for the development of PBPK models presented in this report is tabulated below:

**Software**	**Version**
PK-Sim®	v9.1
MoBi®	v9.1

3.1 Modelling strategy

In the present report, a reference PBPK model was first established for adults (patients as well as healthy volunteers), and subsequently verified against clinical pharmacokinetic data reported for metformin in the scientific literature.

Relevant information on the anthropometry (height, weight) was gathered from the respective clinical studies, if reported. Information on physiological parameters (e.g. blood flows, organ volumes, hematocrit) in adults is available in the PK-Sim® database.

In a second step, a lactation PBPK model was developed, based on the general workflow described by Dallmann *et al. 2018* [3–5].

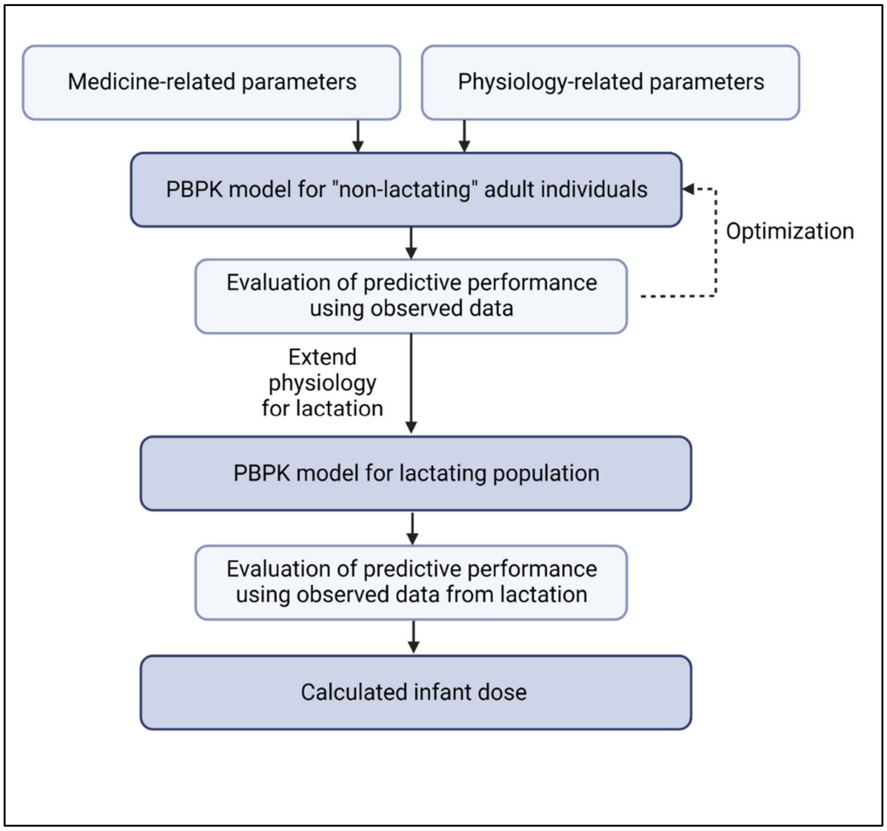

Figure S2 General workflow that was used in the present project to develop and evaluate the lactation PBPK model

Details about input data (physicochemical, *in vitro* and clinical data) can be found in section 3.2. Details about the structural models and their parameters can be found in section 3.3.

3.1.1. Reference PBPK models

The reference PBPK model was taken from literature [1]. Model building and evaluation were based on 39 studies, and are described in the paper and supplementary material. Glomerular filtration and active transport were enabled, as they are involved in metformin circulation. Active transport was implemented via OCT1, OCT2, MATE1 and PMAT. The abundance (including population variability) of plasma proteins and enzymes/transporters that are integrated into PK-Sim are described in the publicly available 'PK-Sim Ontogeny Database Version 7.3' (PK-Sim Ontogeny Database Version 7.3). In addition, the P (intracellular -> interstitial) was adapted in the simulations to account for oral absorption at the upper intestine.

3.1.2. Lactation model

After development of the reference model, the model was exported to MoBi® and a lactation PBPK model was constructed. To model the passage of metformin into human milk, i.e. across the blood/milk biological barrier, both the secretion (CL_{sec}) and reuptake clearance (CL_{re}) values were obtained using the empirical model developed by Koshimichi *et al. 2011* [6].

3.2 Data

3.2.1 *In vitro* / physicochemical data

A literature search was performed to collect available information on physicochemical properties of metformin. The obtained information from literature is summarized in **Error! Reference source not found.**. **Error! Reference source not found.** shows the parameters that were additionally used for the lactation PBPK model.

Table S1 Physicochemical parameters used as input for the metformin PBPK models

Parameter	Value	Unit	Description	Source
MW	129.16	g/mol	Molecular weight	[1]
pK_a	2.80 (base)<br>11.50 (acid)		Logarithm of the acid dissociation constant	
Solubility (pH 7)	350.90	mg/mL	Aqueous solubility	
Log P	-1.43	-	Log10 of the partition coefficient between octanol and water (~lipophilicity)	

f_u	1.00		Fraction unbound in human plasma	
OCT1 Vmax	641.19	µmol/L/min	Specific active transport	
OCT1 Km	1180.00	µM		
OCT2 Vmax	51723.79	µmol/L/min		
OCT2 Km	810.00	µM		
MATE1 Vmax	165.69	µmol/L/min		
MATE1 Km	283.00	µM		
PMAT Vmax	76.47	µmol/L/min		
PMAT Km	367.57	µM		
PMAT Hill coefficient	3.00			
GFR fraction	1.00	-	Fraction of the glomerular filtration rate used for passive renal elimination	

Table S2 Physicochemical parameters used as input for the lactation PBPK model of metformin

Parameter	Value	Unit	Description	Source
Milk logP[a]	-1.43	-	Log10 of the partition coefficient between octanol and water	[1]
HBD	3.00		Hydrogen bond donors	Pubchem
PSA	91.50	$Å^2$	Polar surface area	Pubchem

[a] Milk logP is Log_{10} of the partition coefficient between octanol and water and is used as input for the calculations in the postpartum model (see equations below). In theory, this value is identical to the logP specified in Table S1. However, in some PBPK models, logP (Table S1) might be optimized using parameter identication. Therefore, it was chosen to use a separate parameter (i.e. Milk logP) to represent the logP used as input for the equations in the postpartum model.

The default equations for free fraction in human milk and logD that were implemented in the spatial structure building block that was developed for the postpartum women are described below. Alternatively, these values can be overwritten by values calculated elsewhere (e.g. MarvinSketch) or determined *in vitro.*

The free fraction in human milk was calculated with the equations proposed by Atkinson and Begg [7], as follows:

$$f_{u\_skimmed\ milk} = \frac{f_u \times 0.448}{(0.000694^{0.448} + \mathrm{f}_{\mathrm{u}}^{0.448})}$$

$$P_{milk} = 10^{(-0.88+1.29\times \log D7.2)}$$

$$\text{Total free fraction in milk} = \frac{1}{(\frac{0.955}{f_{u\_skimmed\ milk}} + 0.045 \times P_{milk})}$$

Where: f_u *skimmed milk:* binding to proteins in milk; P_{milk}: partitioning between aqueous and lipid phase of milk; Total free fraction in milk: 'total' free fraction, i.e. accounting for both protein and lipid binding processes.

LogD values taking into account up to three pka values (as provided in the compound building block), were calculated as follows:

$$LogD = \text{Log}\,P + Log_{10}(logD_{factor})$$

With Milk logP (Table S2) as input for logP

$$LogD_{factor} = \mathrm{K}_1 + (\mathrm{K}_2 + \mathrm{K}_3 + \mathrm{K}_4) \times \mathrm{base}^1 + \mathrm{K}_5 \times \mathrm{base}^{\max(\mathrm{CT}_0+\mathrm{CT}_1;-\mathrm{CT}_0-\mathrm{CT}_1)} + \mathrm{K}_6 \times \mathrm{base}^{\max(CT_0+CT_2;-CT_0-CT_2)} + \mathrm{K}_7 \times \mathrm{base}^{\max(\mathrm{CT}_2+\mathrm{CT}_1;-\mathrm{CT}_2-\mathrm{CT}_1)} + \mathrm{K}_8 \times \mathrm{base}^{\max(\mathrm{CT}_0+\mathrm{CT}_1+\mathrm{CT}_2;-\mathrm{CT}_0-\mathrm{CT}_1-\mathrm{CT}_2)}$$

$K_1 = F_1 \times F_2 \times F_3$
$K_2 = (1 - F_1) \times F_2 \times F_3$
$K_3 = F_1 \times (1 - F_2) \times F_3$
$K_4 = F_1 \times F_2 \times (1 - F_3)$
$K_5 = (1 - F_1) \times (1 - F_2) \times F_3$
$K_6 = (1 - F_1) \times F_2 \times (1 - F_3)$
$K_7 = (1 - F_1) \times F_2 \times (1 - F_3)$
$K_8 = (1 - F_1) \times (1 - F_2) \times (1 - F_3)$

F1 = CT0 != CT_NEUTRAL ? 1/(1+10^(CT0*(pKa_0- pH))) : 1
F2 = CT1 != CT_NEUTRAL ? 1/(1+10^(CT1*(pKa_1- pH))) : 1
F3 = CT2 != CT_NEUTRAL ? 1/(1+10^(CT2*(pKa_2- pH))) : 1

With CT = compound type (-1: acid; +1: base; 0: neutral), and pH = 7.2 or 7.4 respectively for $logD_{7.2}$ *and* $logD_{7.4}$

The transports that were added in the passive transport building block for 'transfer to milk' and 'transfer from milk' are based on secretion and reuptake and clearance values, Cl_{sec} and Cl_{re}, which were calculated according to the empirical equations proposed by Koshimich et al. 2011 [6], as follows:

$$\text{Log}\,CL_{re} = 2.793 + 0.179 \times LogP - 0.132 \times HBD$$

$$\text{Log}\,CL_{sec} = 3.367 \times Log10(MW) - 0.164 \times (LogP - LogD) - 0.015 \times PSA - 3.912$$

3.2.2 Clinical data

The reference PBPK model was taken from literature [1]. The model was developed and verified using 39 clinical trials with intravenous and oral administration. Information about the simulated trials and demographic information is described in the electronic supplementary material [1].

The evaluation of the predictive performance of the metformin lactation PBPK model was performed using 5 different studies were metformin was administered as an oral dose of 500 mg bidaily to lactating women [8–12]. The women time postpartum was either early postpartum, not later than 3 months postpartum, or not mentioned in the studies. The samples were assumed to be through samples if the exact timing was not reported in the articles.

Detailed information and data from the studies used for model building, verification, and lactation model can be found in Supplementary material 1 and 2.

3.2.2.1 Lactation PBPK model

Table S3 shows the study that was used for the lactation PBPK model.

Table S3 Summary of study used for model development of metformin PBPK model in lactating women

Study ID	Publication	Arm/treatment/information used for model building and verification
Briggs 2005	[8]	5 women (early postpartum) received PO 500 mg bidaily (multiple dose)
Eyal 2010	[9]	1 woman (> 3 months postpartum) received PO 1500 mg/day (multiple dose)
Gardiner 2003	[10]	5 women received PO 500 mg (single dose)
Gardiner 2003	[10]	3 women received PO 500 mg bidaily (multiple dose)
Hale 2002	[11]	5 women received PO 500 mg thrice daily (multiple dose)
Zhang 2002	[12]	1 woman received PO 500 mg bidaily (multiple dose)

3.3 Model Parameters and assumptions

3.3.1 Absorption

The release of metformin from the tablet was implemented as a Weibull function. Specific intestinal permeability was identified during parameter identification.

3.3.2 Distribution

The tissue partition coefficients (K_p) calculation was according to 'PK-Sim Standard' and the cellular permeability calculation was 'Charge dependent Schmitt normalized to PK-Sim'.

3.3.3 Metabolism and excretion

The final model applies transportation and glomerular filtration. The transportation kinetics were identified during parameter identification and the glomerular filtration was set to its default value of 1.

3.3.4 Secretion to milk

To model the transfer process of metformin into human milk, both the secretion (CL_{sec}) and reuptake clearance (CL_{re}) were calculated using the empirical equations developed by Koshimichi *et al.* 2011 (see **Error! Reference source not found.**) [6].

First, in MoBi®, a spatial structure for the postpartum women was constructed, similar to the workflow from Dallmann *et al.* 2018 [3]. Here, breasts were added as a compartment. In addition, the human milk was connected to the plasma subcompartment of the breasts. The human milk volume was specified as 0.5 L to represent the structure of Koshimichi *et al.* 2011, and a geometric standard deviation of 1.16 was assumed in the population. The free fraction in human milk, and logD values were implemented as the equations described previously. The transfer between plasma and milk was defined as two kinetic processes (transfer to milk and transfer from milk) under passive transports (see below). Next, the simulation was combined with the postpartum population from Job *et al.* 2021 in PK-Sim to account for the postpartum physiology [5].

<u>*Kinetics*</u>

Transfer to milk

$$\frac{dNmilk}{dt} = Cplasma \times f_u \times CL_{sec}$$

where Cplasma is the concentration in plasma (in breast compartment), f_u is the free fraction in plasma and CL_{sec} is the secretion clearance.

Transfer from milk

$$\frac{dNplasma}{dt} = Cmilk \times f_u \times CL_{re}$$

where Cmilk is the concentration in human milk, f_u is the total free fraction in human milk (protein and lipid) and CL_{re} is the reuptake clearance.

The median simulated plasma and human milk concentration-time profiles can be used to calculate the M/P ratio as follows:

$$M/P\ ratio\ = \frac{AUC_{milk}}{AUC_{plasma}}$$

3.4. Infant dosage calculation

Infant dosage via human milk was then calculated based on the predicted (average and maximal) steady-state metformin concentration in human milk, as well as the daily milk intake volume. The daily infant dosage was then compared to the maternal dosage, resulting in the relative infant dose (RID).

$$Daily\ infant\ dosage = C_{average} * 150\ \frac{mL}{kg\ .day}$$

$$Daily\ infant\ dosage = C_{max} * 150\ \frac{mL}{kg\ .day}$$

$$Relative\ infant\ dose\ (RID) = \frac{Infant\ dosage}{Maternal\ dosage} * 100\ \%$$

4. Results

The reference PBPK model of metformin was taken from literature [1]. The postpartum PBPK model of metformin was developed and verified with clinical PK data.

The model was evaluated covering studies including in particular:

- Intravenous and oral administration
- Single and multiple doses
- Paired milk/plasma data

The model quantifies the transport via OCT1&2, MATE1 and PMAT; and glomerular filtration for metformin. Moreover, secretion and reuptake to human milk were described by CL_{sec} and CL_{re}.

The next sections show:

- The final model parameters for the building blocks: section 4.1
- The overall predictive performance: section 4.2
- The simulated versus observed concentration-time profiles for the clinical studies used for model building and for model verification: section 4.3

4.1 Final input parameters

The compound values of the final postpartum PBPK model for metformin are illustrated below.

Physicochemical parameters

<table>
<tr><th>Parameter</th><th>Value</th><th>Unit</th><th>Source</th></tr>
<tr><td>MW</td><td>129.16</td><td>g/mol</td><td rowspan="5">[1]</td></tr>
<tr><td>pKa</td><td>2.80 (base)
11.50 (acid)</td><td>-</td></tr>
<tr><td>Solubility</td><td>350.90</td><td>mg/mL</td></tr>
<tr><td>Lipophilicity</td><td>-1.43</td><td>-</td></tr>
<tr><td>f_u</td><td>1.00</td><td>-</td></tr>
<tr><td>Small molecule (Y/N)</td><td>Yes</td><td>-</td><td>-</td></tr>
</table>

Calculation methods

Name	Value
Tissue partition coefficients	PK-Sim Standard
Cellular permeabilities	Charge dependent Schimtt normalized to PK-Sim

ADME-related parameters

Parameter	Value	Unit	Source
Intestinal permeability	8.49E-7	cm/min	[1]
OCT1 Vmax	641.19	µmol/L/min	
OCT1 Km	1180.00	µM	
OCT2 Vmax	51723.79	µmol/L/min	
OCT2 Km	810	µM	
MATE1 Vmax	165.69	µmol/L/min	
MATE1 Km	283.00	µM	
PMAT Vmax	76.47	µmol/L/min	
PMAT Km	367.57	µM	
PMAT Hill coefficient	3.00		
GFR fraction	1	-	

Simulation parameters

Parameter	Value	Unit	Source
P (intracellular -> interstitial) Duodenum	1.65E-5	cm/min	[13]
P (intracellular -> interstitial) Upper Jejunum	1.65E-5	cm/min	
P (intracellular -> interstitial) Lower Jejunum	1.65E-5	cm/min	
P (intracellular -> interstitial) Upper Ileum	1.65E-5	cm/min	
P (intracellular -> interstitial) Lower Ileum	1.65E-5	cm/min	
P (intracellular -> interstitial) Cecum	0	cm/min	
P (intracellular -> interstitial) Colon Ascendens	0	cm/min	
P (intracellular -> interstitial) Colon Transversum	0	cm/min	
P (intracellular -> interstitial) Colon Descendens / Distal Colon 1	0	cm/min	
P (intracellular -> interstitial) Colon Sigmoid / Distal Colon 2	0	cm/min	
P (intracellular -> interstitial) Rectum	0	cm/min	

Formulation-related parameters
Type: Solution

Type: Weibull

Parameter	Value	Unit	Source
Dissolution time	7.90	Min	[1]
Lag time	0	Min	
Dissolution shape	1.36		
Use as suspension	Yes		

Type: Weibull with food

Parameter	Value	Unit	Source
Dissolution time	7.90	Min	[1]
Lag time	0	Min	
Dissolution shape	0.11		
Use as suspension	Yes		

Physicochemical and physiological parameters relevant to the lactation model

Parameter	Value	Unit	Source
Milk log P	-1.43	Log units	[1]
HBD	3.00	**-**	Pubchem
PSA	91.50	$Å^2$	Pubchem
CL_{sec}	3.62E-4	L/min	Default
CL_{re}	2.31E-3	L/min	Default
*fu*_skimmed milk[a]	0.96	-	Default
Pmilk[b]	2.71E-7	-	Default
Total free fraction in milk[c]	1.01	-	Default
$logD_{7.2}$	-4.41	Log units	Default
$logD_{7.4}$	-4.40	Log units	Default

[a] binding to proteins in milk; [b] partitioning between aqueous and lipid phase of milk; [c] total free fraction, accounting for both protein and lipid binding

4.2 Diagnostic plots

The geometric mean fold errors (GMFE) on AUC and Cmax were 1.12 and 1.13 for the model building dataset, and 1.22 and 1.27 for the model verification dataset.
The diagnostic plots for the reference PBPK model are described in the electronic supplementary material published by Hanke *et al.* (2020) [1]. The following shows the predictive performance graph for Cmax and AUC of metformin for the PBPK model performance of all data used.

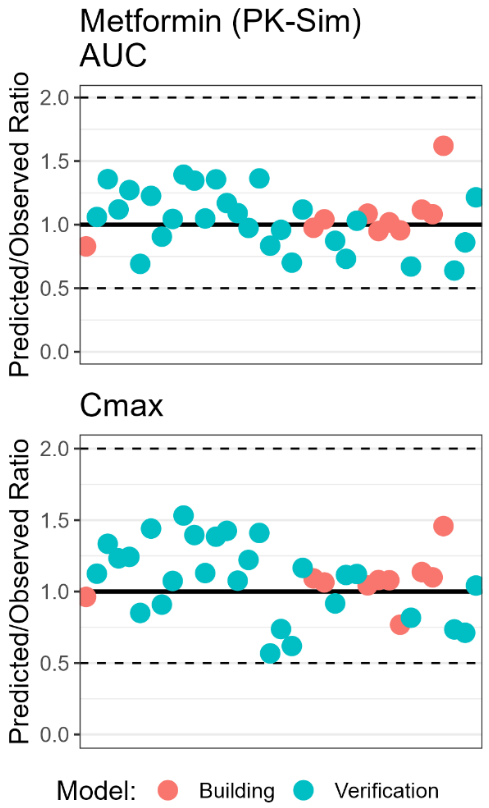

Figure S3 Predicted over observed ratio profile

Table S4 Ratio between the predicted and observed pharmacokinetic parameters of metformin in different dosing regimens for model building

Study ID/ Reference	Dose/ Route	AUC_{obs} (mg*h/L)	AUC_{pred} (mg*h/L)	Fold error	$Cmax_{obs}$ (mg/L)	$Cmax_{pred}$ (mg/L)	Fold error
Boehringer 2018 [14]	500 mg PO SD	6.97	5.78	0.83	1.01	0.97	0.96
Sambol 1996 [15]	1700 mg PO SD	17.39	16.97	0.98	2.41	2.63	1.09
Sambol 1996 [15]	2550 mg PO SD	22.63	23.59	1.04	3.40	3.62	1.06
Sambol 1996 [16][17]	850 mg PO SD	8.84	9.61	1.09	1.49	1.55	1.04
Sambol 1996 [17]	850 mg PO SD	7.06	6.71	0.95	0.92	0.99	1.08
Sambol 1996 [17]	850 mg PO SD	9.06	9.23	1.02	1.39	1.50	1.08
Sirtori 1978 [18]	1000 mg IV SD	23.77	22.72	0.96	32.27	24.78	0.77
Stopfer 2016 [19]	PO 500 mg SD	5.58	6.24	1.12	0.89	1.01	1.14
Stopfer 2018 [20]	PO 500 mg SD	5.58	6.04	1.08	0.89	0.98	1.10
Tucker 1981 [21]	250 mg IV SD	4.44	7.19	1.62	7.53	10.98	1.46

Table S5 Ratio between the predicted and observed pharmacokinetic parameters of metformin in different dosing regimens used for model verification

Study ID/ Reference	Dose/ Route	AUC_{obs} (mg*h/L)	AUC_{pred} (mg*h/L)	Fold error	$Cmax_{obs}$ (mg/L)	$Cmax_{pred}$ (mg/L)	Fold error
Caillé 1993 [22]	500 mg PO SD	4.20	4.45	1.06	0.63	0.71	1.13
Chen 2009 [23]	850 mg PO SD	7.73	10.49	1.36	1.36	1.82	1.33
Cho 2011 [24]	750 mg PO MD	9.03	10.11	1.12	1.46	1.80	1.23
Cho 2014 [25]	750 mg PO MD	7.75	9.86	1.27	1.42	1.77	1.24
DiCicco 2014 [26]	500 mg PO SD	6.59	4.56	0.69	0.87	0.74	0.85
Ding 2014 [27]	750 mg PO MD	7.79	9.55	1.23	1.16	1.68	1.44
Gan 2016 [28]	500 mg PO bid	15.03	13.62	0.91	2.34	2.13	0.91
Gusler 2001 [29]	500 mg PO SD	4.94	5.16	1.05	0.71	0.76	1.07
Hibma 2016 [30]	850 mg PO MD	7.08	9.86	1.39	1.15	1.76	1.53
Jang 2016 [31]	500 mg PO MD	5.98	8.06	1.35	1.08	1.50	1.40
Johansson 2014 [32]	1000 mg PO SD	10.12	10.62	1.05	1.47	1.67	1.13
Kim 2014 [33]	500 mg PO MD	5.89	8.00	1.36	1.04	1.44	1.38
Manitpisitkul 2014 [34]	500 mg PO MD	5.76	6.74	1.17	0.83	1.19	1.42
Morissey 2016 [35]	850 mg PO SD	10.40	11.35	1.09	1.69	1.82	1.08
Najib 2002 [36]	500 mg PO SD	6.60	6.44	0.97	0.93	1.14	1.22
Oh 2015 [37]	500 mg PO MD	6.00	8.18	1.36	1.08	1.52	1.41
Pentikainen 1978 [38]	500 mg IV SD	17.16	14.33	0.83	33.61	19.07	0.57
Pentikainen 1978 [38]	500 mg PO SD	7.98	7.66	0.96	1.63	1.20	0.74
Robert 2003 [39]	850 mg PO SD	7.98	5.60	0.70	1.57	0.97	0.62
Sambol 1995 [40]	850 mg PO SD	8.74	9.79	1.12	1.33	1.55	1.17
Sambol 1996 [17]	850 mg PO SD	11.21	9.79	0.87	1.67	1.53	0.92
Sambol 1996 [17]	850 mg PO MD	14.77	10.80	0.73	1.81	2.02	1.12

Sambol 1996 [16]	500 mg PO SD	6.42	6.62	1.03	0.96	1.08	1.12
Somogyi 1987 [41]	PO 250 mg/day	4.24	2.85	0.67	0.57	0.47	0.82
Tucker 1981 [21]	1500 mg PO SD	18.79	12.01	0.64	2.33	1.71	0.74
Tucker 1981 [21]	500 mg PO SD	5.30	4.56	0.86	0.98	0.70	0.71
Wang 2008 [42]	500 mg PO MD	5.49	6.67	1.22	1.08	1.13	1.04

4.3 Concentration-time profiles

Simulated versus observed concentration-time profiles of all data listed in section 3.2.2 are presented below. The original.pksim5 are provided in Supplemented material 3.

4.3.1 Model building

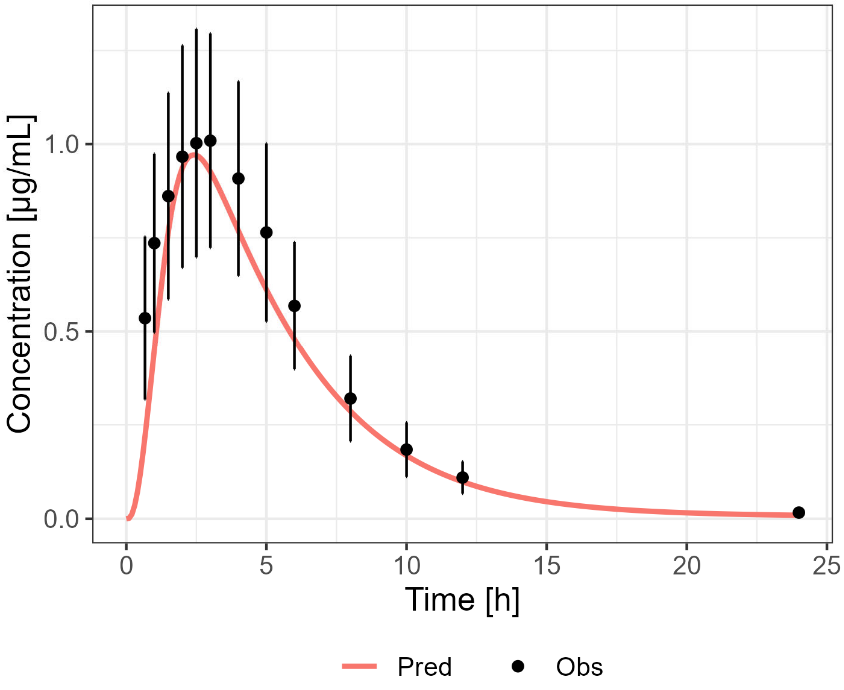

Figure S4 Predicted (Pred) versus observed (Obs) concentration-time profile after administration of 500 mg PO SD [14]

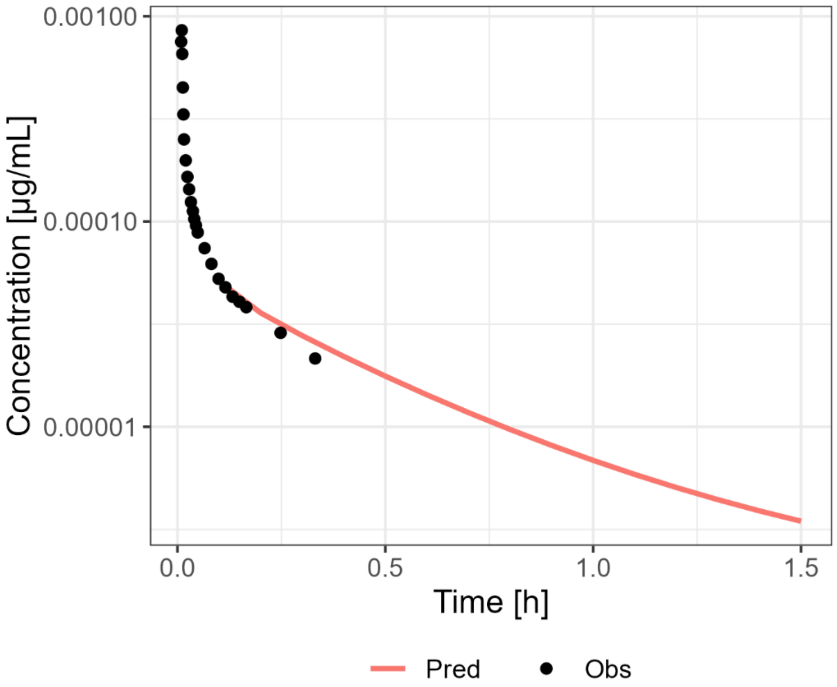

Figure S5 Predicted (Pred) versus observed (Obs) concentration-time profile after administration of 1.445 µg IV [40]

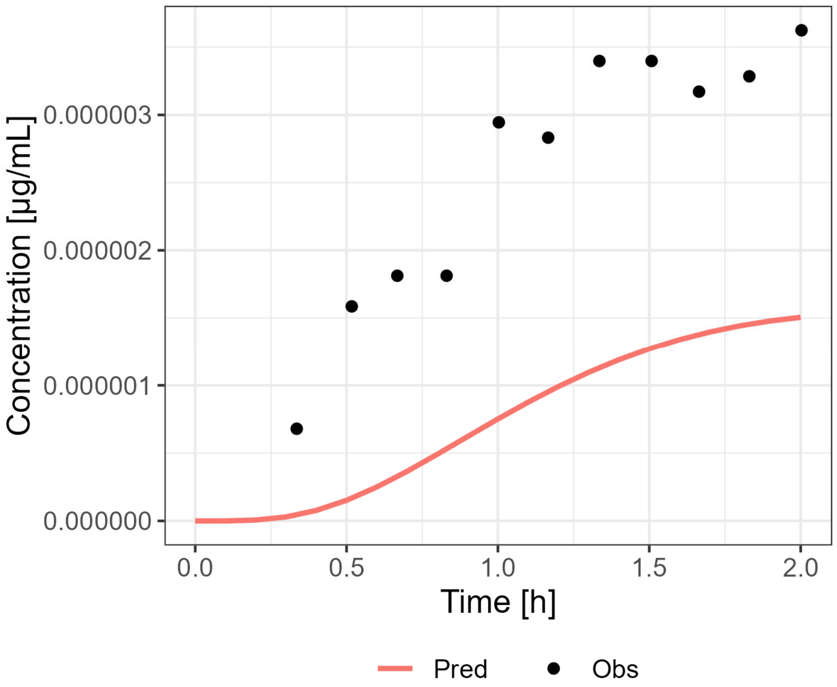

Figure S6 Predicted (Pred) versus observed (Obs) concentration-time profile after administration of 0.8556 µg PO [40]

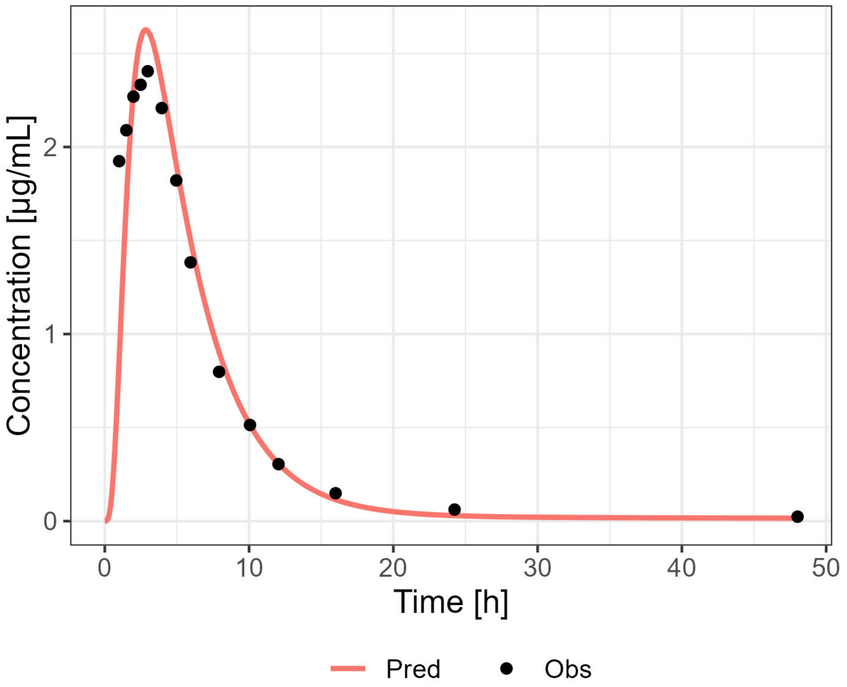

Figure S7 Predicted (Pred) versus observed (Obs) concentration-time profile after administration of 1700 mg PO [17]

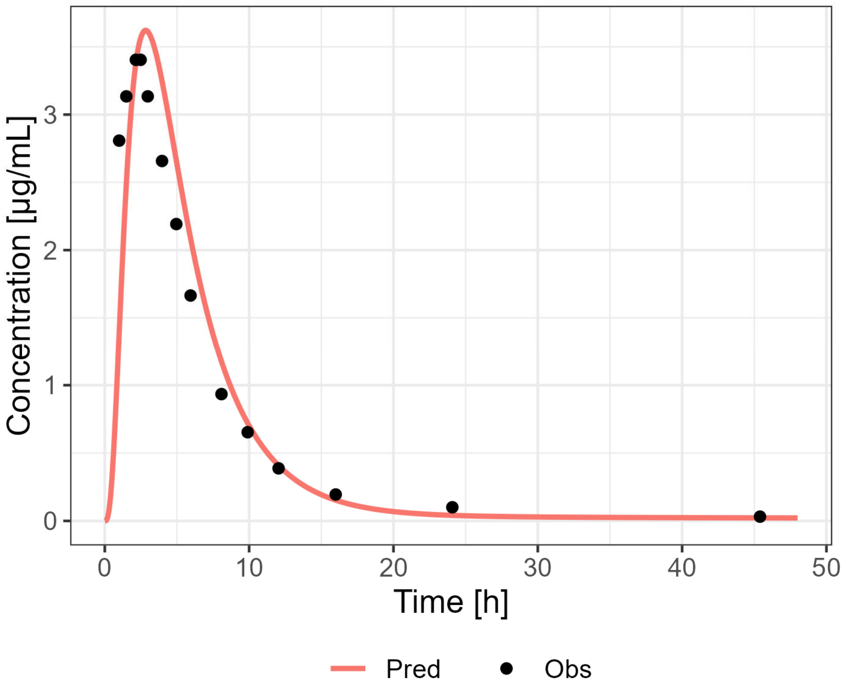

Figure S8 Predicted (Pred) versus observed (Obs) concentration-time profile after administration of 2550 mg PO [17]

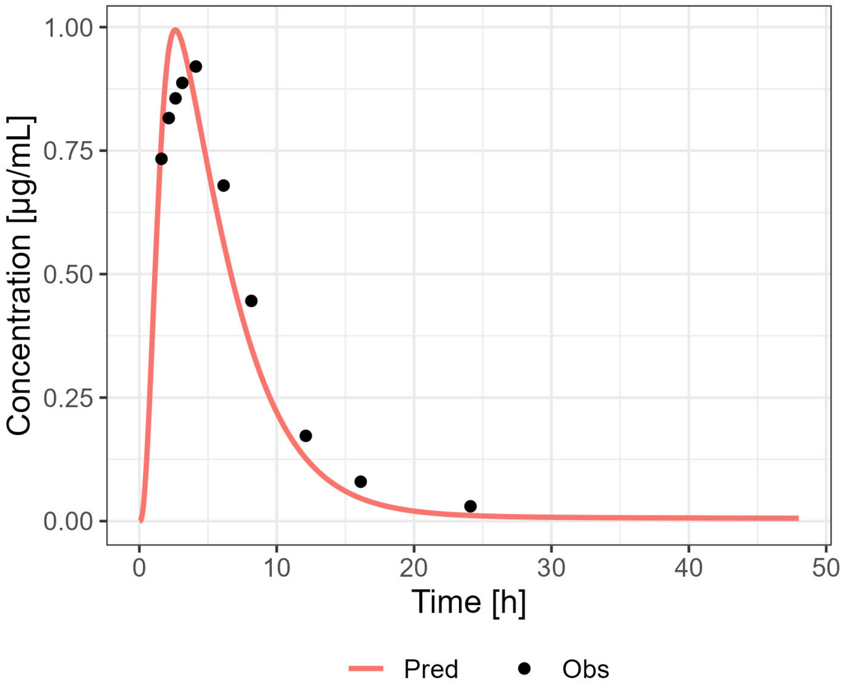

Figure S9 Predicted (Pred) versus observed (Obs) concentration-time profile after administration of 850 mg PO fed [15]

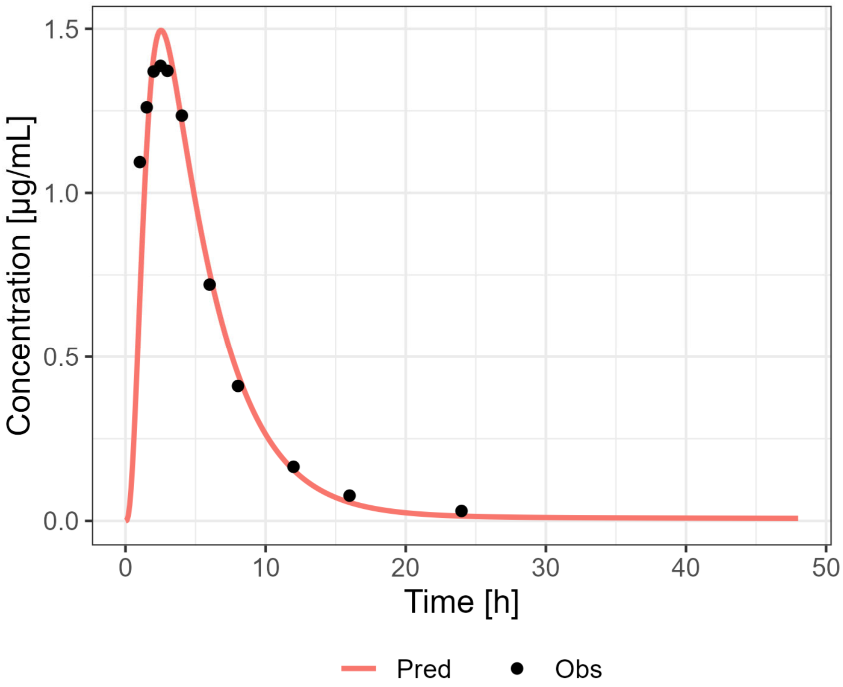

Figure S10 Predicted (Pred) versus observed (Obs) concentration-time profile after administration of 850 mg solution PO fed [15]

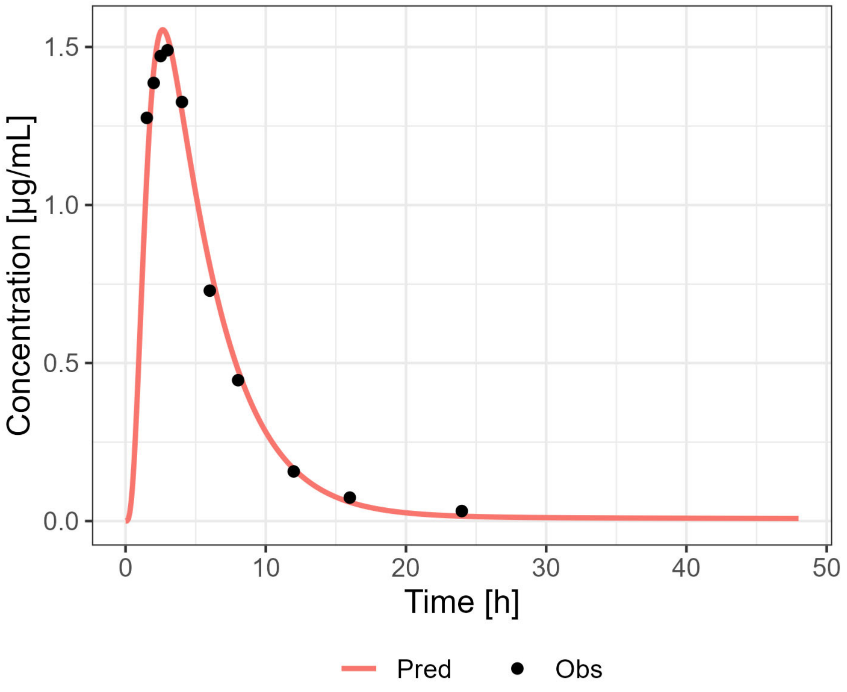

Figure S11 Predicted (Pred) versus observed (Obs) concentration-time profile after administration of 850 mg PO [15]

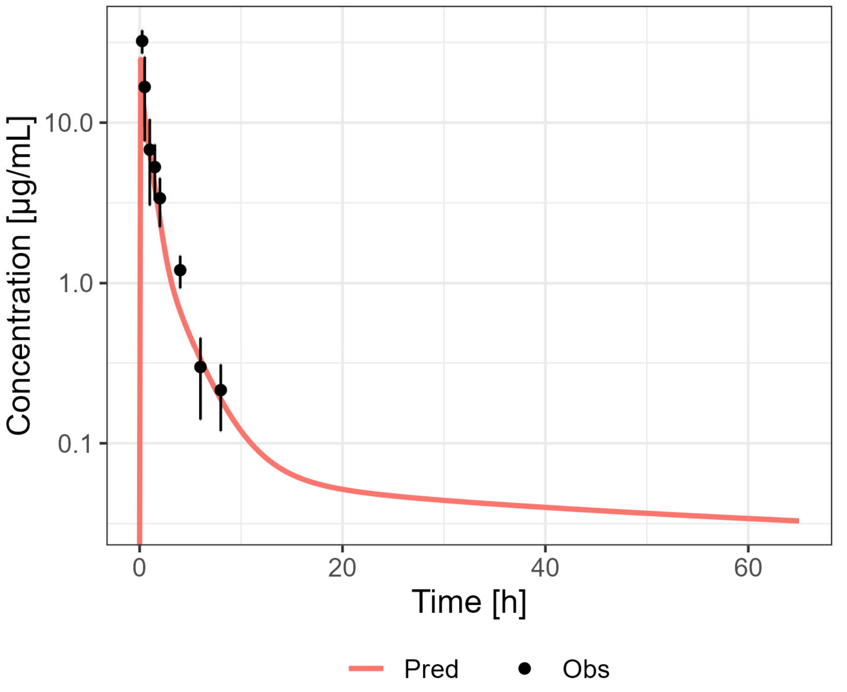

Figure S12 Predicted (Pred) versus observed (Obs) concentration-time profile after administration of 1000 mg IV [18]

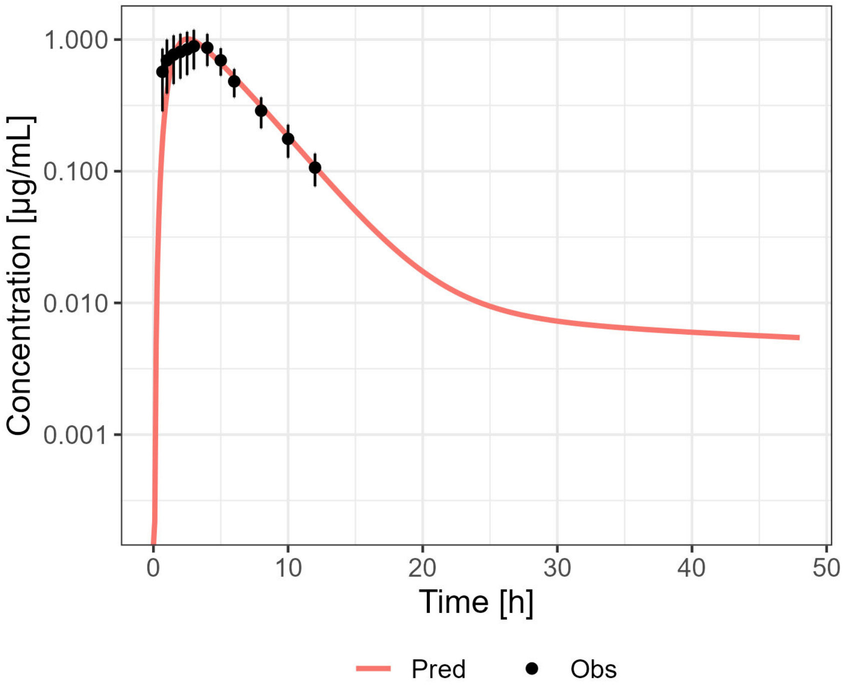

Figure S13 Predicted (Pred) versus observed (Obs) concentration-time profile after administration of metformin [19]

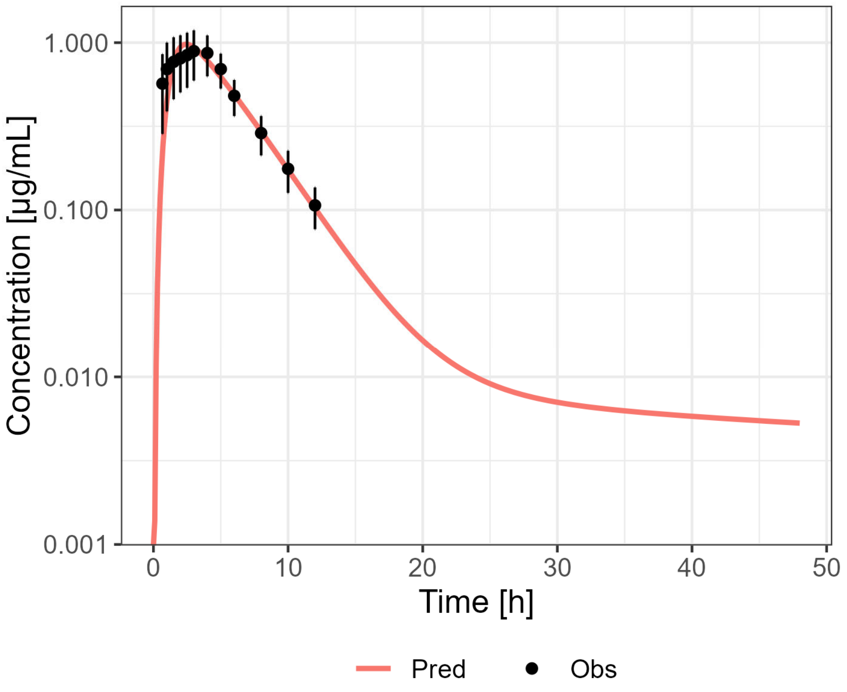

Figure S14 Predicted (Pred) versus observed (Obs) concentration-time profile after administration of metformin [20]

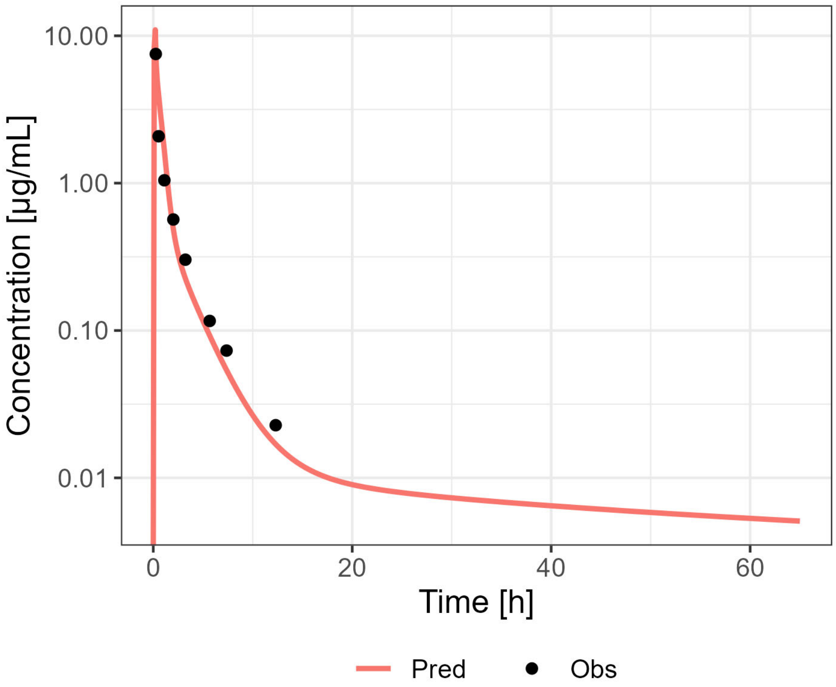

Figure S15 Predicted (Pred) versus observed (Obs) concentration-time profile after administration of 250 mg IV SD [21]

4.3.2 Model verification

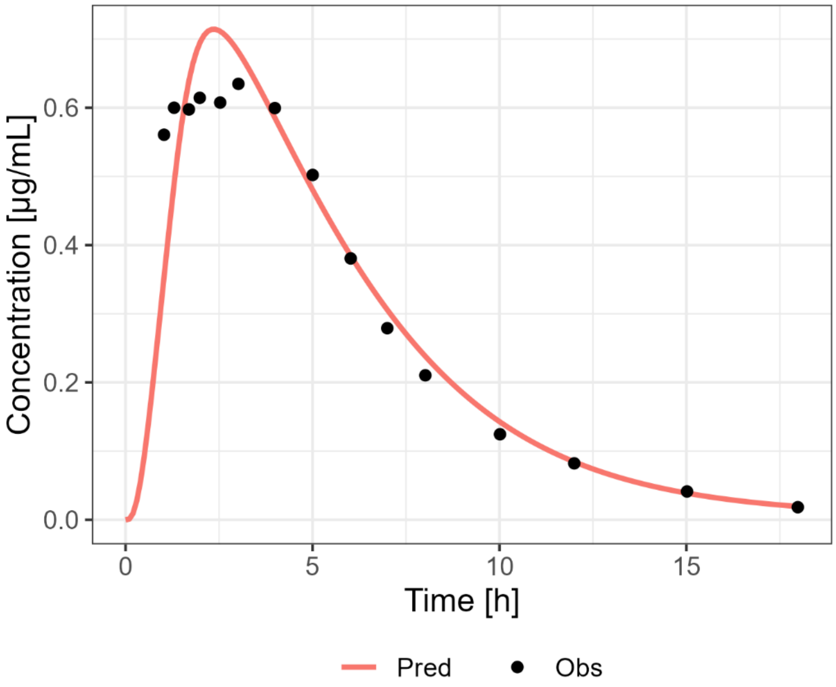

Figure S16 Predicted (Pred) versus observed (Obs) concentration-time profile after administration of 500 mg PO [22]

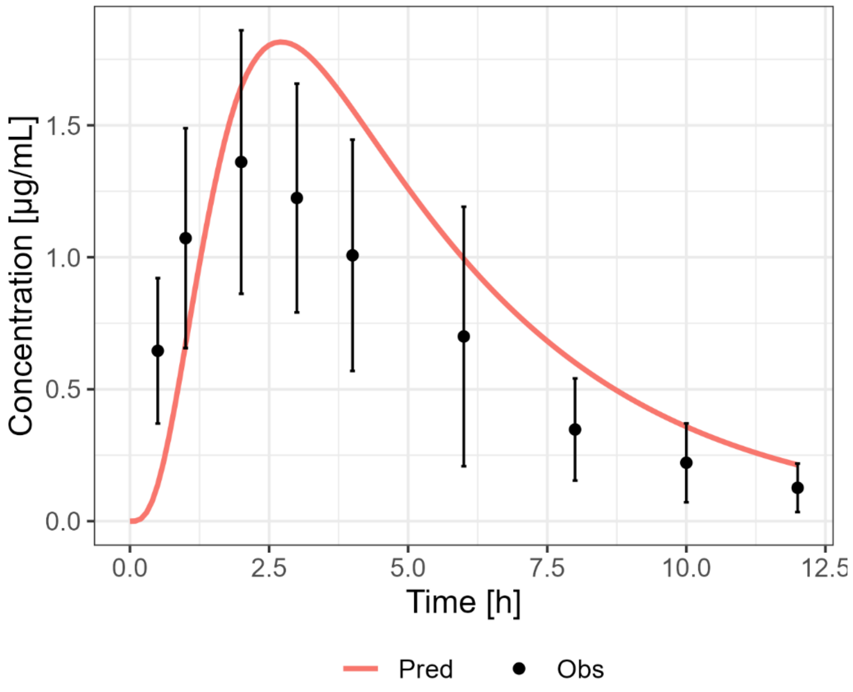

Figure S17 Predicted (Pred) versus observed (Obs) concentration-time profile after administration of 850 mg PO [23]

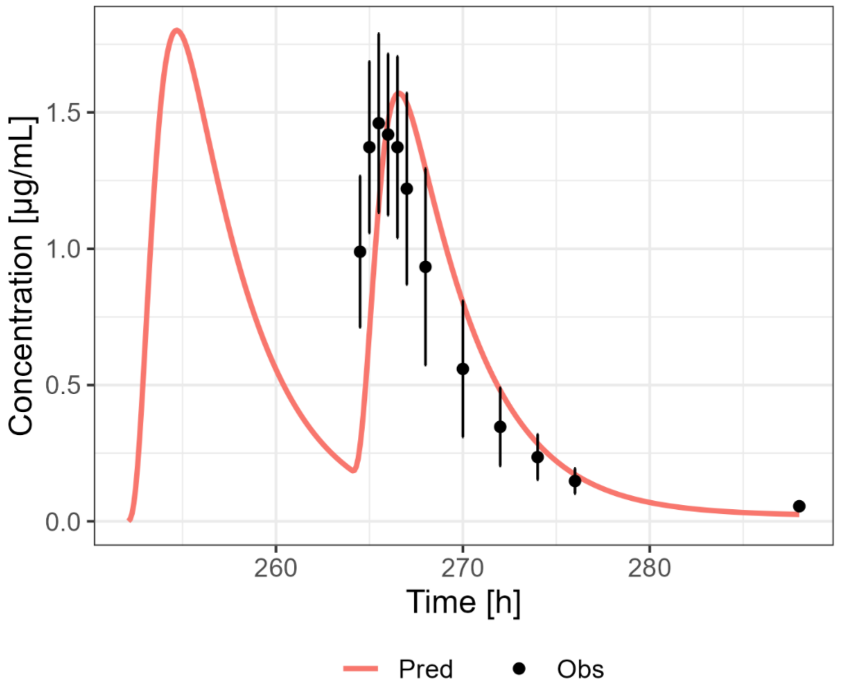

Figure S18 Predicted (Pred) versus observed (Obs) concentration-time profile after administration of 750 mg bid PO [24]

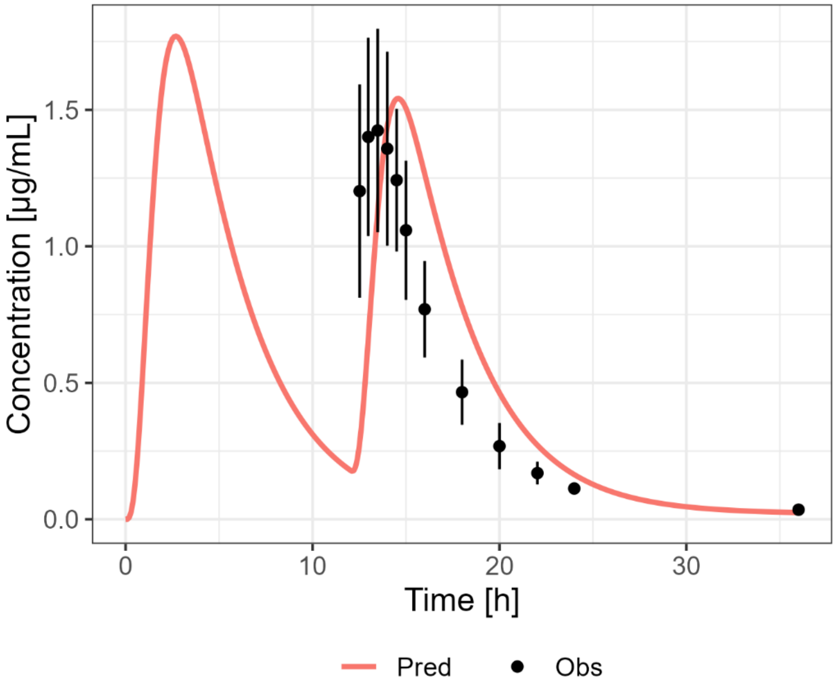

Figure S19 Predicted (Pred) versus observed (Obs) concentration-time profile after administration of 750 mg bid PO [25]

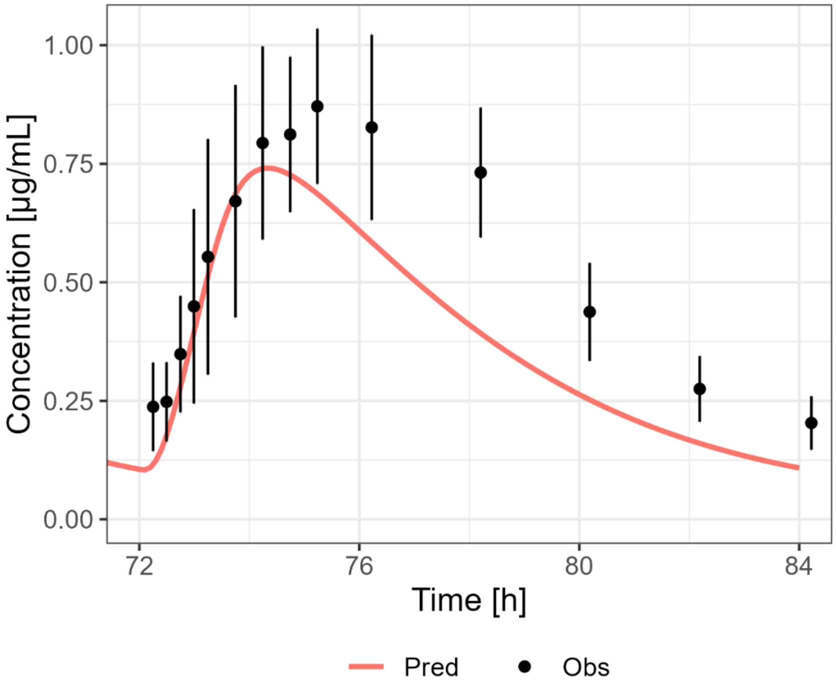

Figure S20 Predicted (Pred) versus observed (Obs) concentration-time profile after administration of 500 mg PO [26]

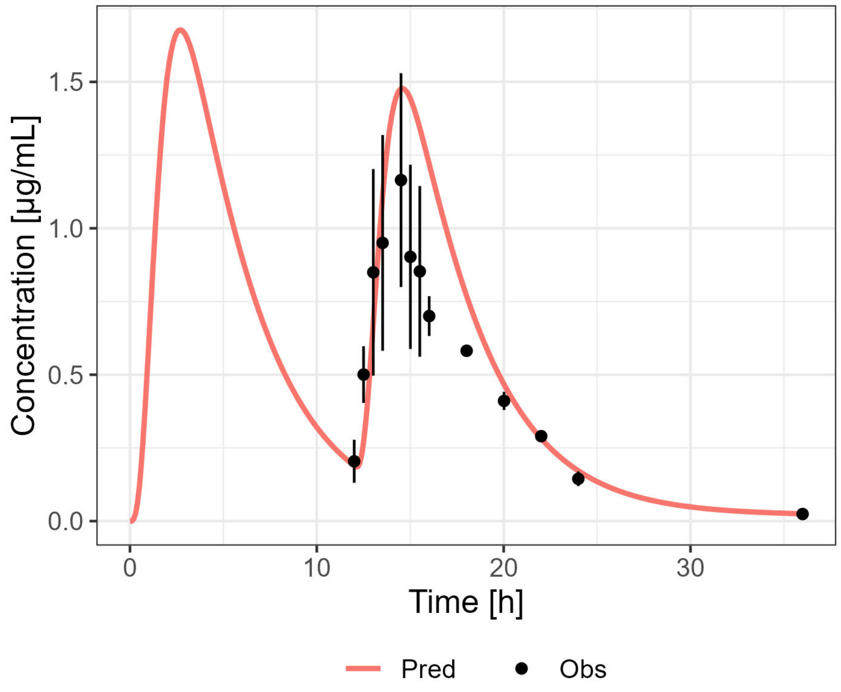

Figure S21 Predicted (Pred) versus observed (Obs) concentration-time profile after administration of 750 mg bid PO [27]

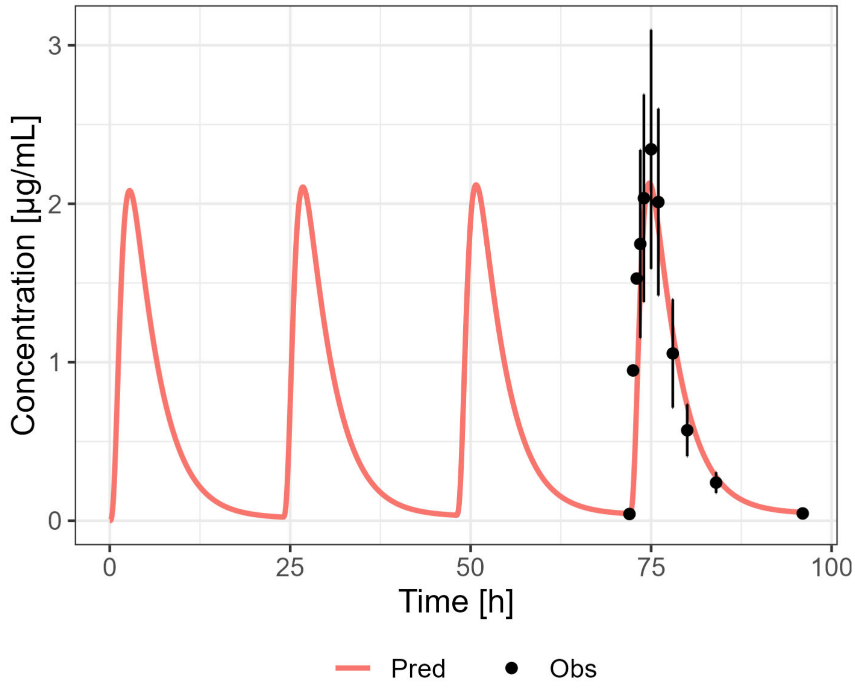

Figure S22 Predicted (Pred) versus observed (Obs) concentration-time profile after administration of 1000 mg PO daily [28]

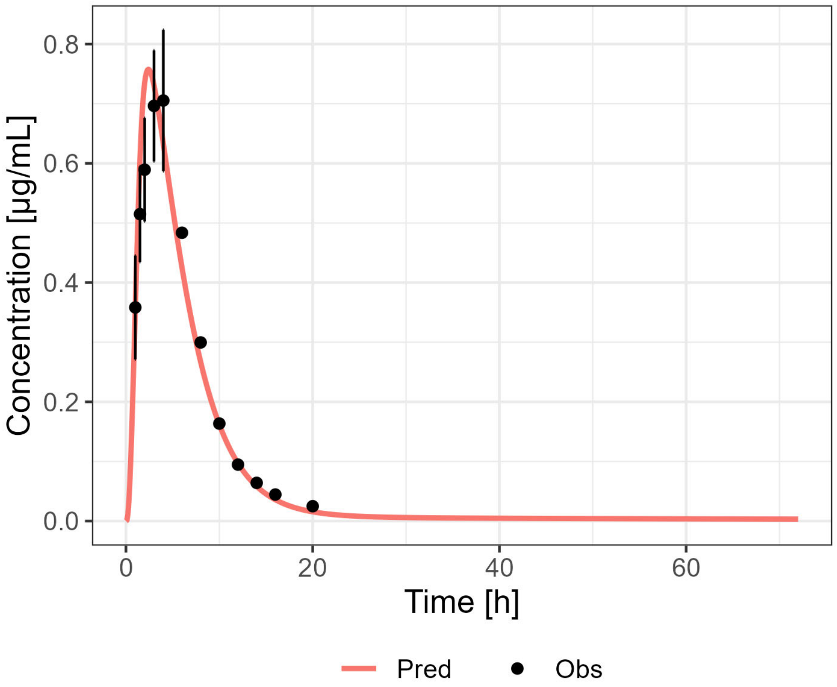

Figure S23 Predicted (Pred) versus observed (Obs) concentration-time profile after administration of 500 mg PO [29]

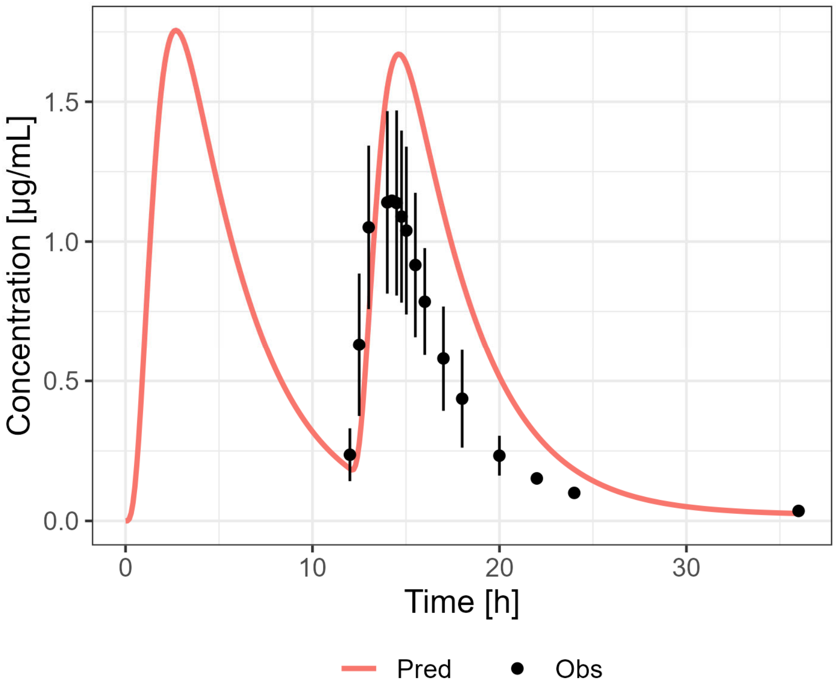

Figure S24 Predicted (Pred) versus observed (Obs) concentration-time profile after administration of 850 mg bid PO [30]

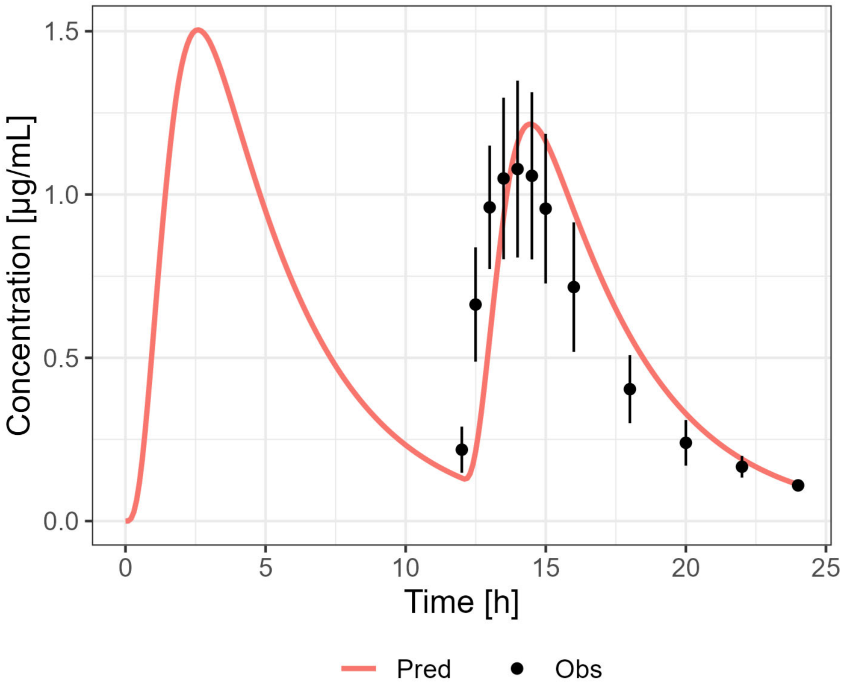

Figure S25 Predicted (Pred) versus observed (Obs) concentration-time profile after administration of 500 mg bid PO [31]

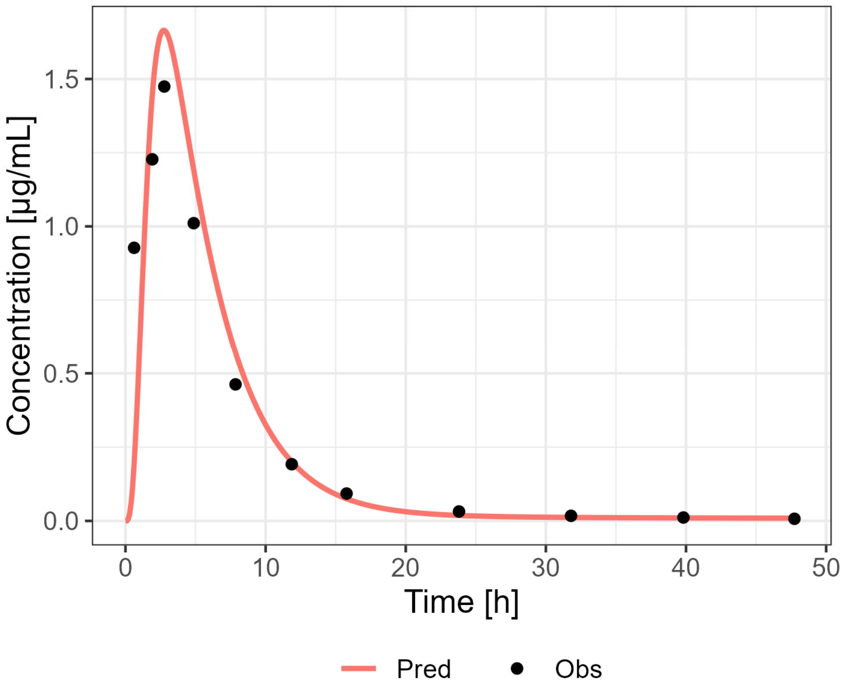

Figure S26 Predicted (Pred) versus observed (Obs) concentration-time profile after administration of 1000 mg PO [32]

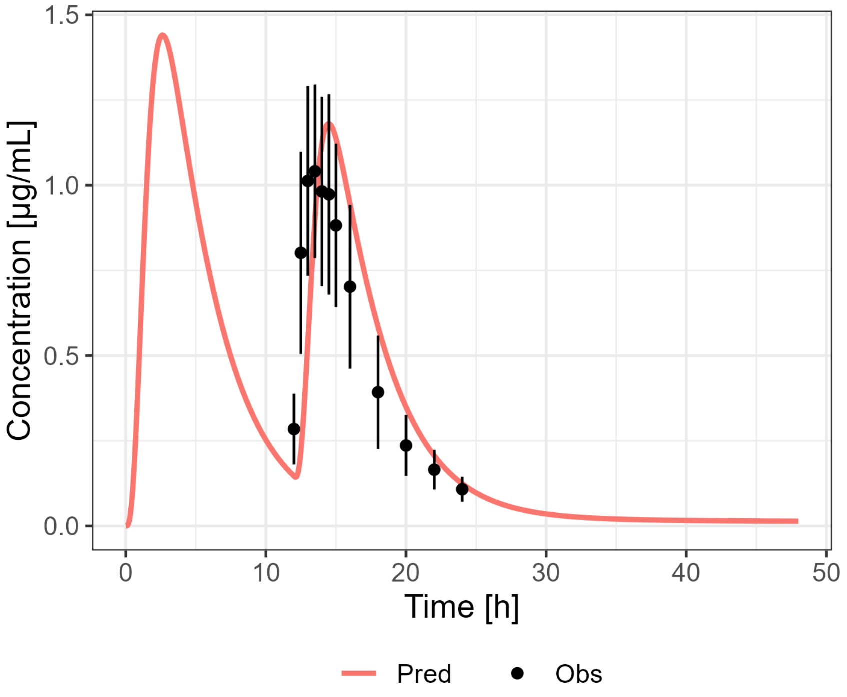

Figure S27 Predicted (Pred) versus observed (Obs) concentration-time profile after administration of 500 mg bid PO [33]

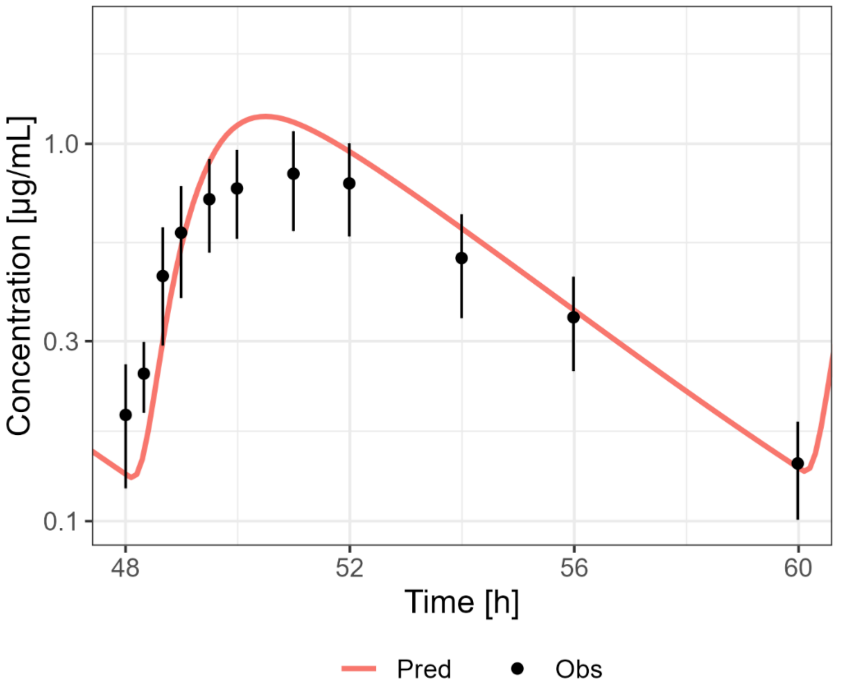

Figure S28 Predicted (Pred) versus observed (Obs) concentration-time profile after administration of 500 mg bid PO [34]

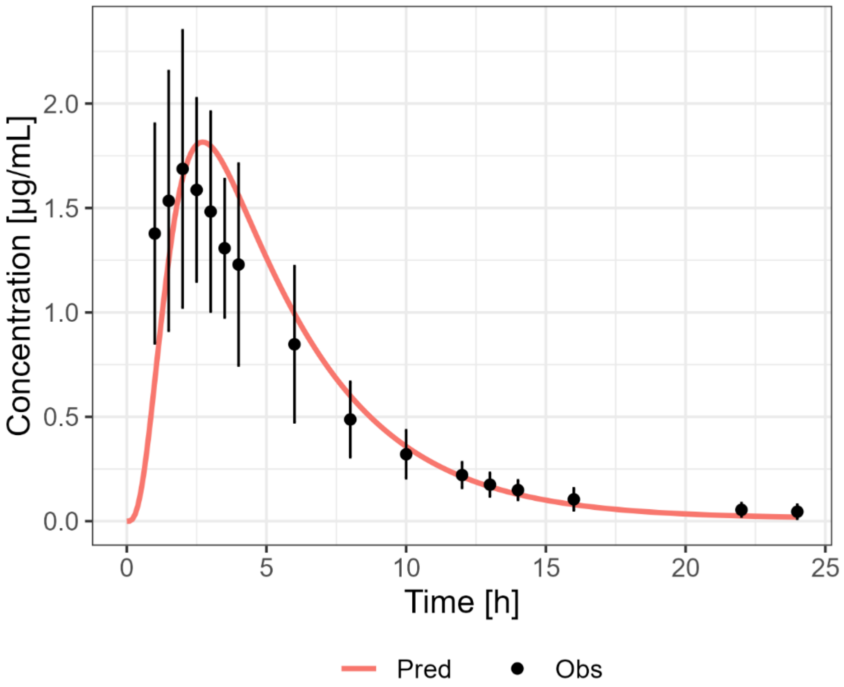

Figure S29 Predicted (Pred) versus observed (Obs) concentration-time profile after administration of 850 mg PO [35]

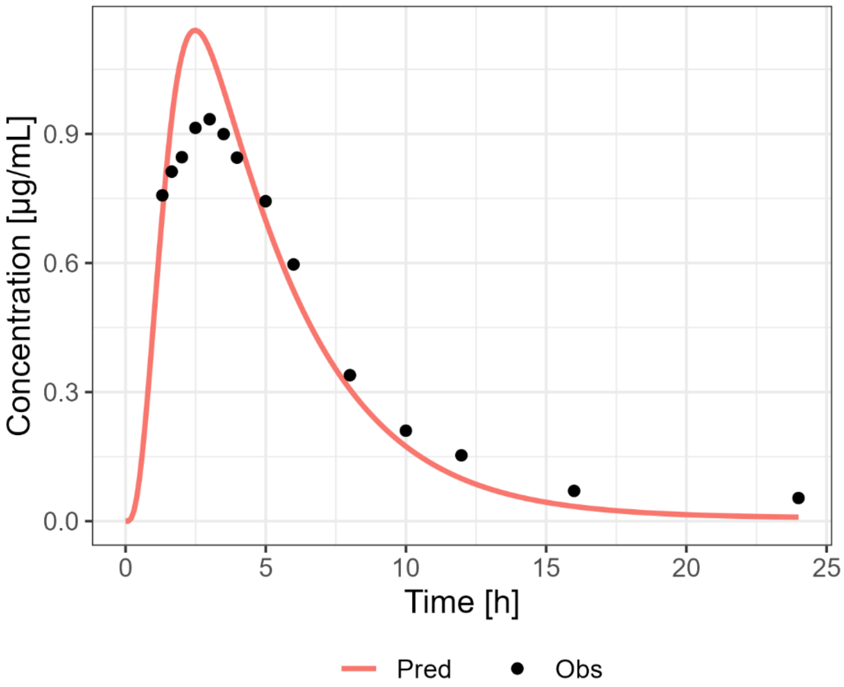

Figure S30 Predicted (Pred) versus observed (Obs) concentration-time profile after administration of 500 mg PO [36]

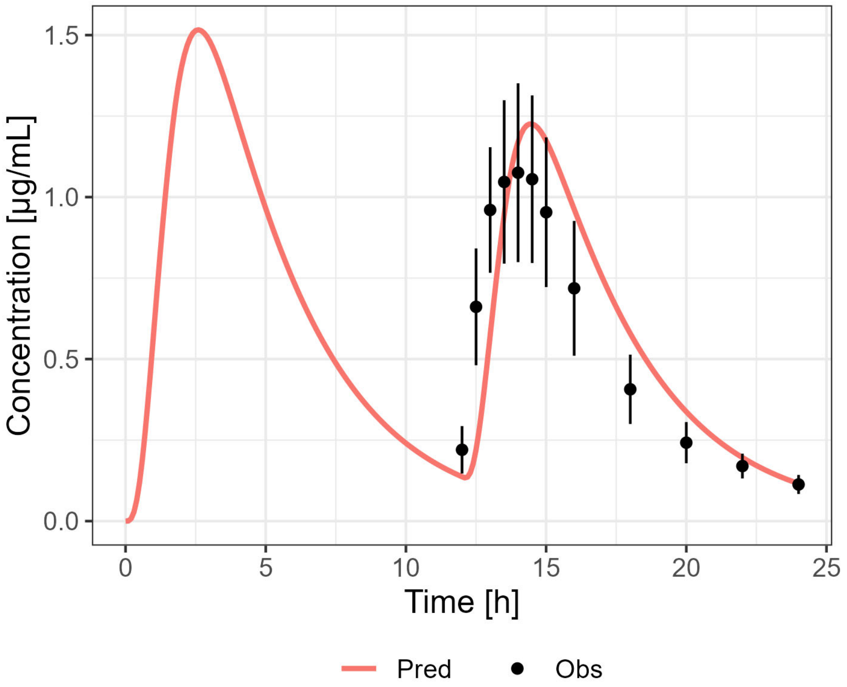

Figure S31 Predicted (Pred) versus observed (Obs) concentration-time profile after administration of 500 mg bid PO [37]

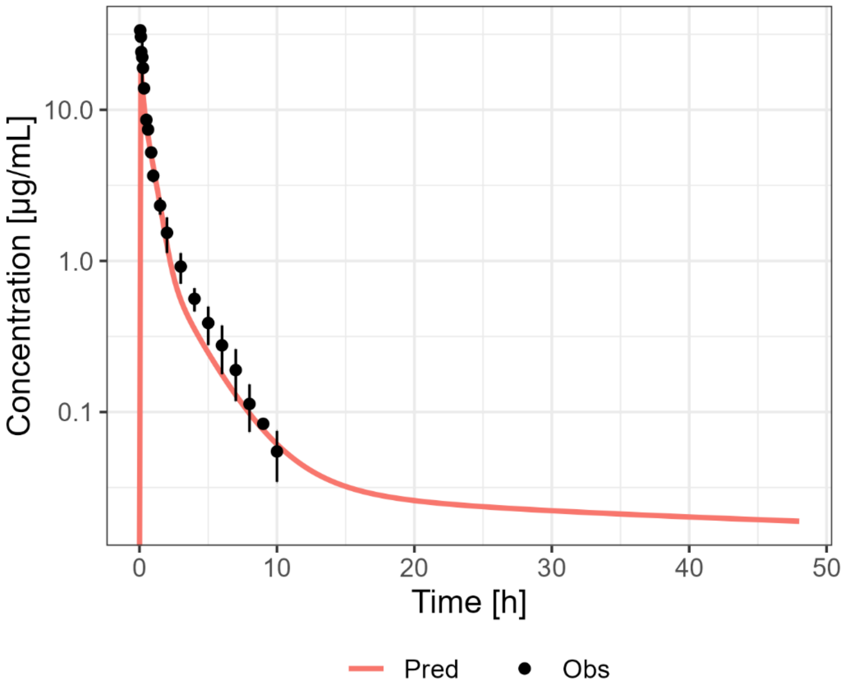

Figure S32 Predicted (Pred) versus observed (Obs) concentration-time profile after administration of 500 mg IV [38]

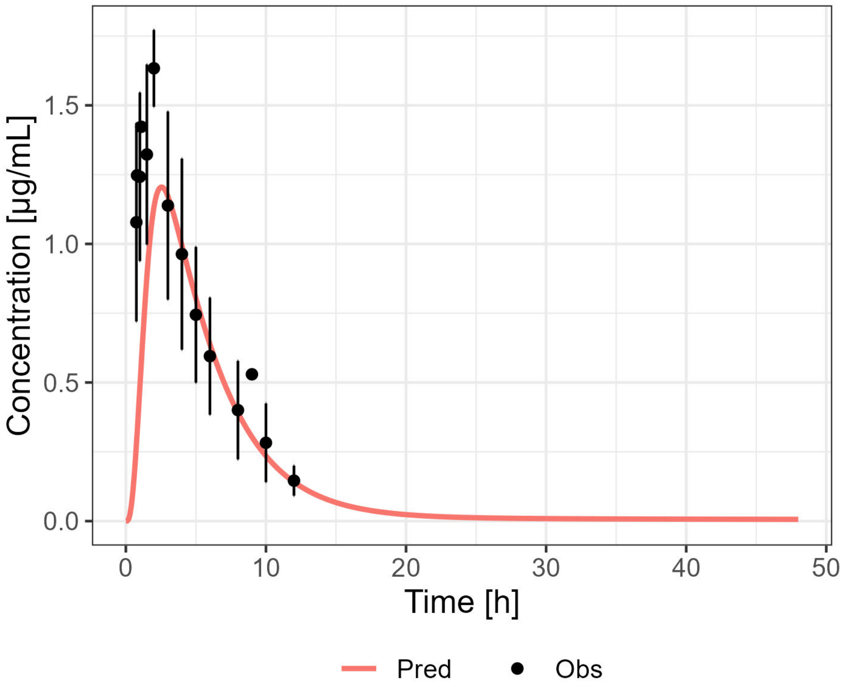

Figure S33 Predicted (Pred) versus observed (Obs) concentration-time profile after administration of 500 mg PO [38]

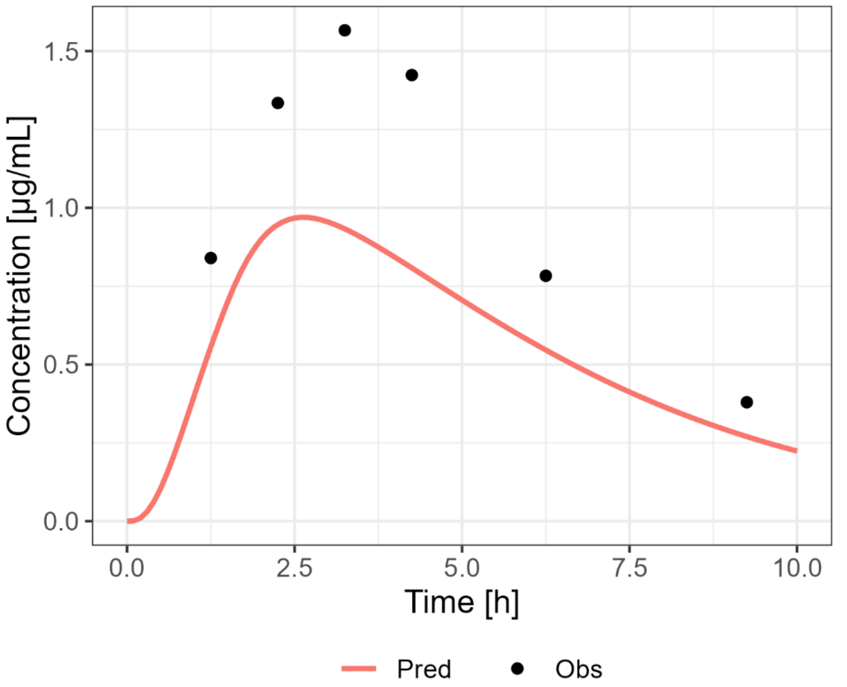

Figure S34 Predicted (Pred) versus observed (Obs) concentration-time profile after administration of 850 mg PO [39]

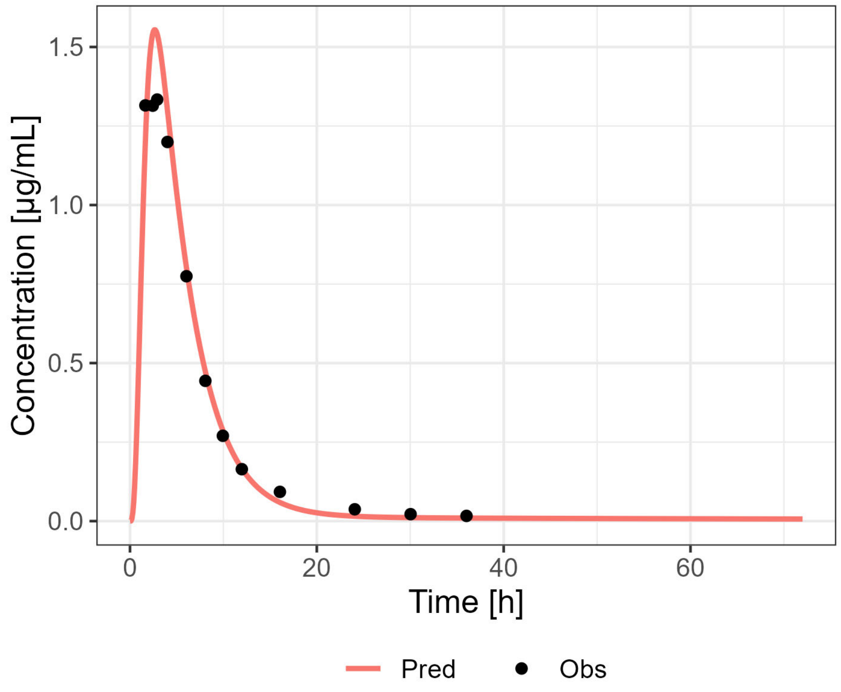

Figure S35 Predicted (Pred) versus observed (Obs) concentration-time profile after administration of 850 mg PO [16]

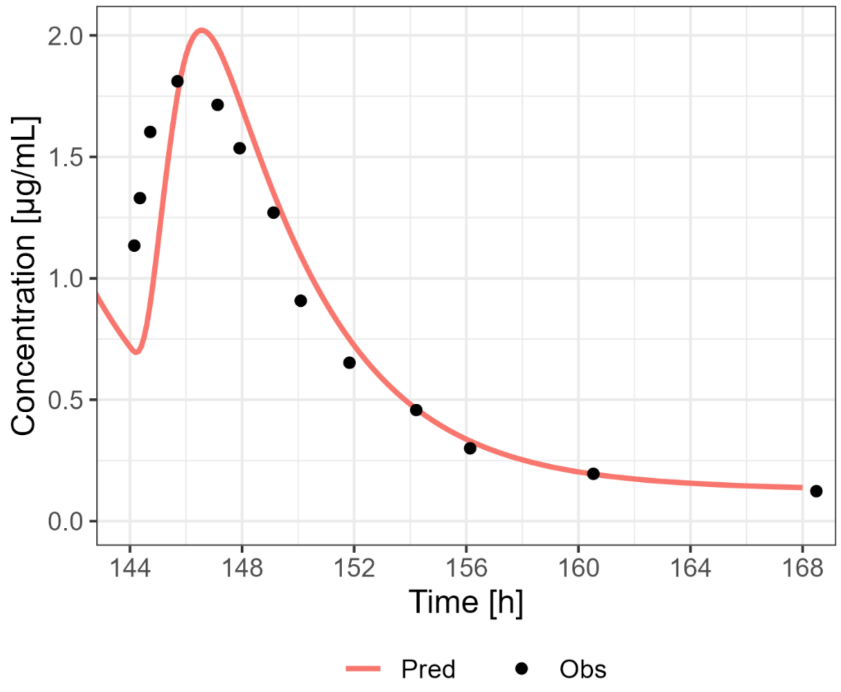

Figure S36 Predicted (Pred) versus observed (Obs) concentration-time profile after administration of 850 mg tid PO [15]

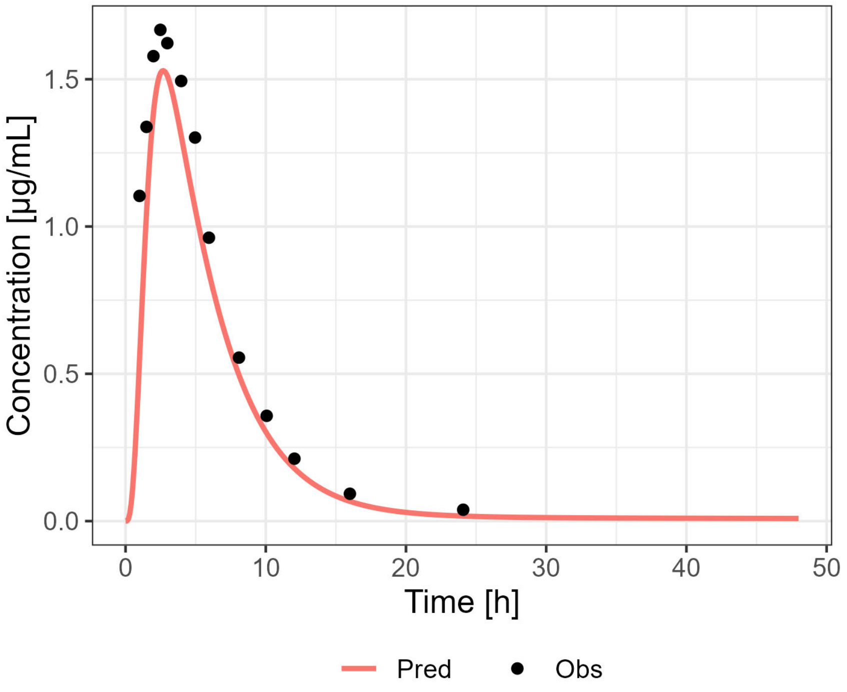

Figure S37 Predicted (Pred) versus observed (Obs) concentration-time profile after administration of 850 mg PO [15]

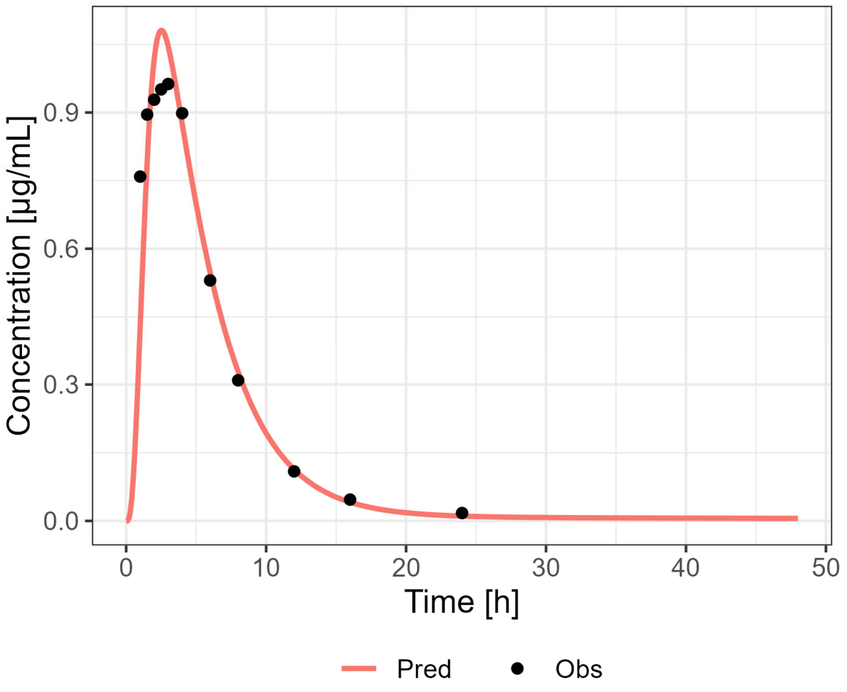

Figure S38 Predicted (Pred) versus observed (Obs) concentration-time profile after administration of 500 mg PO [17]

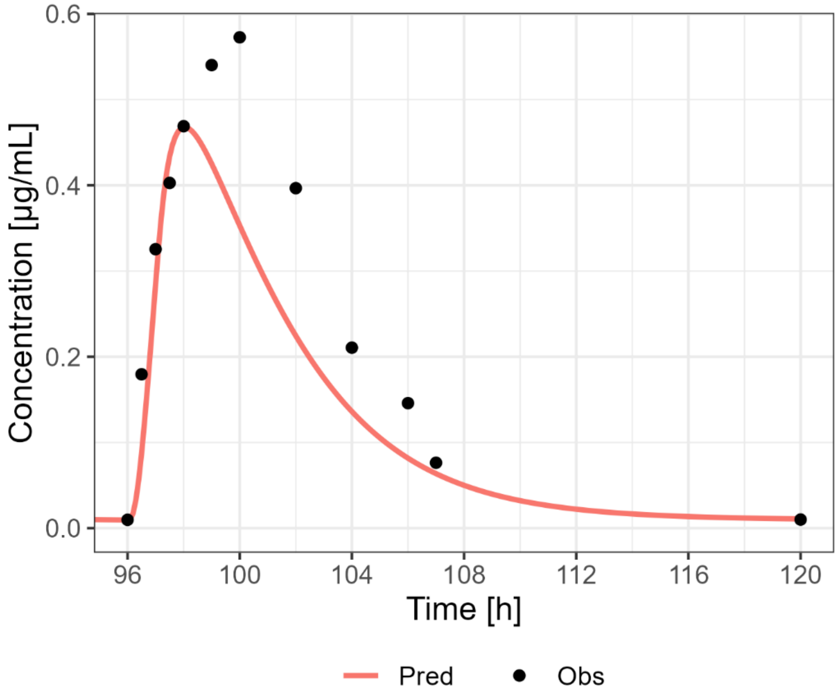

Figure S39 Predicted (Pred) versus observed (Obs) concentration-time profile after administration of 250 mg/day PO [41]

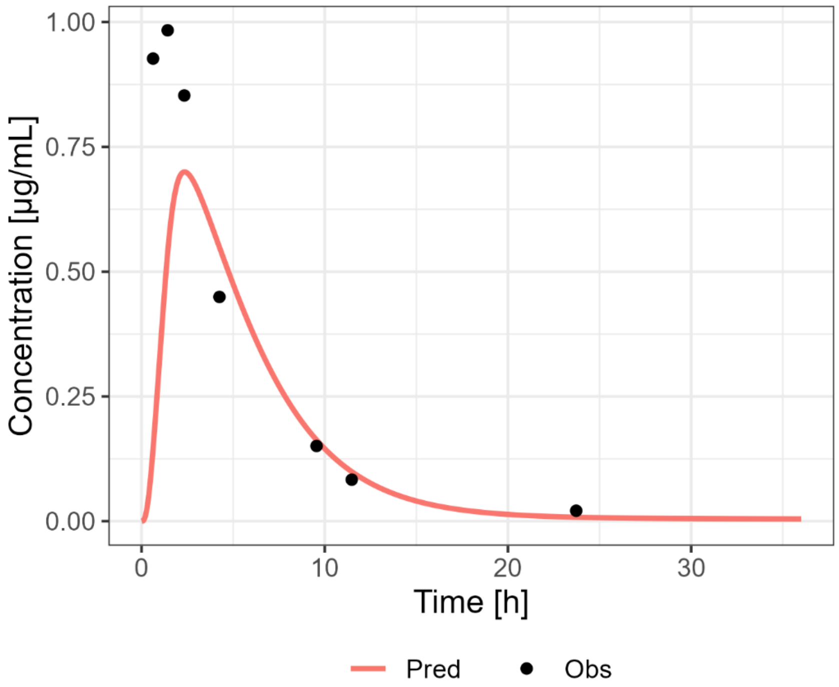

Figure S40 Predicted (Pred) versus observed (Obs) concentration-time profile after administration of 500 mg fed PO [21]

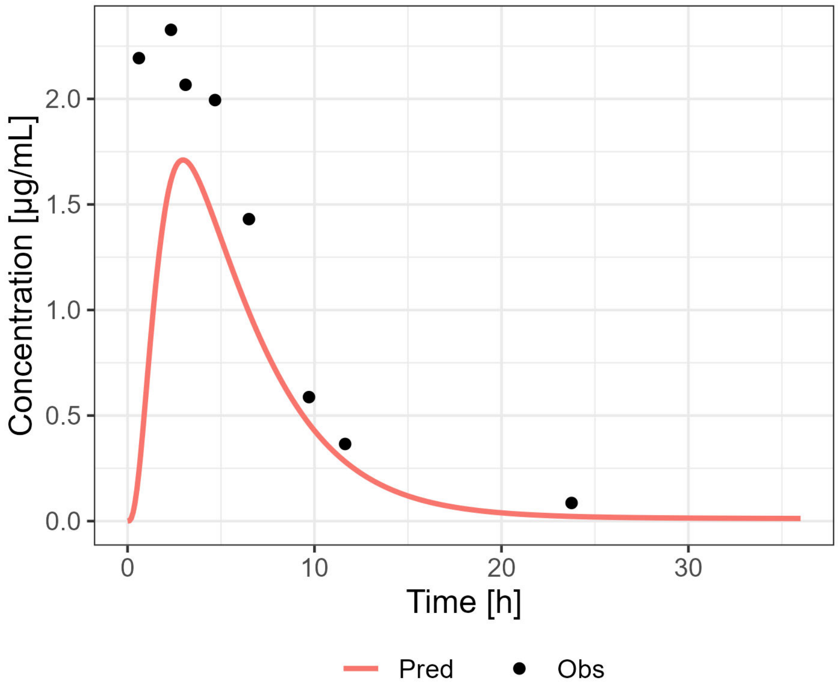

Figure S41 Predicted (Pred) versus observed (Obs) concentration-time profile after administration of 1500 mg fed PO [21]

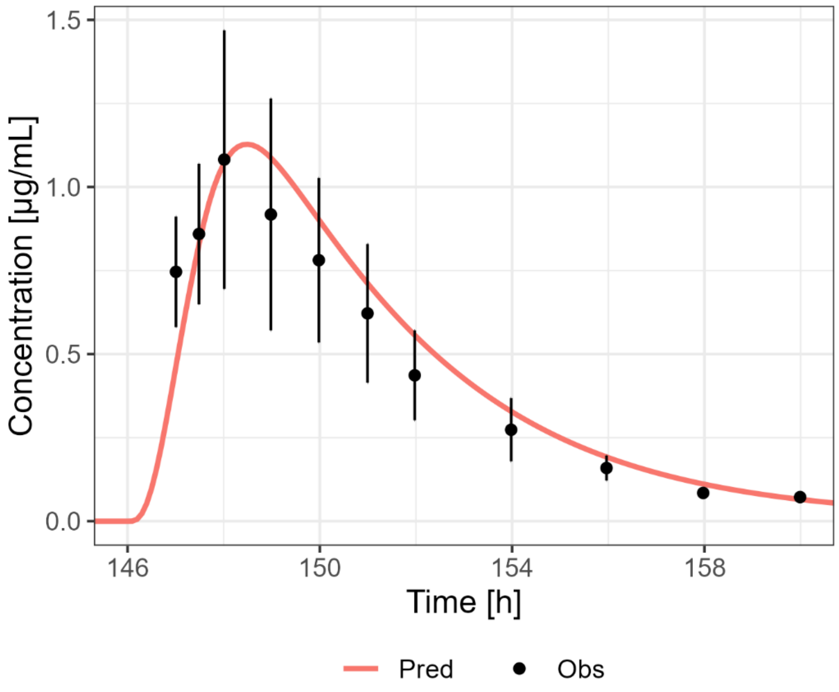

Figure S42 Predicted (Pred) versus observed (Obs) concentration-time profile after administration of 500 mg PO [42]

4.3.3 Lactation PBPK model

A sample size of 1000 individuals, three months postpartum, was used in each simulation of the virtual lactation population.

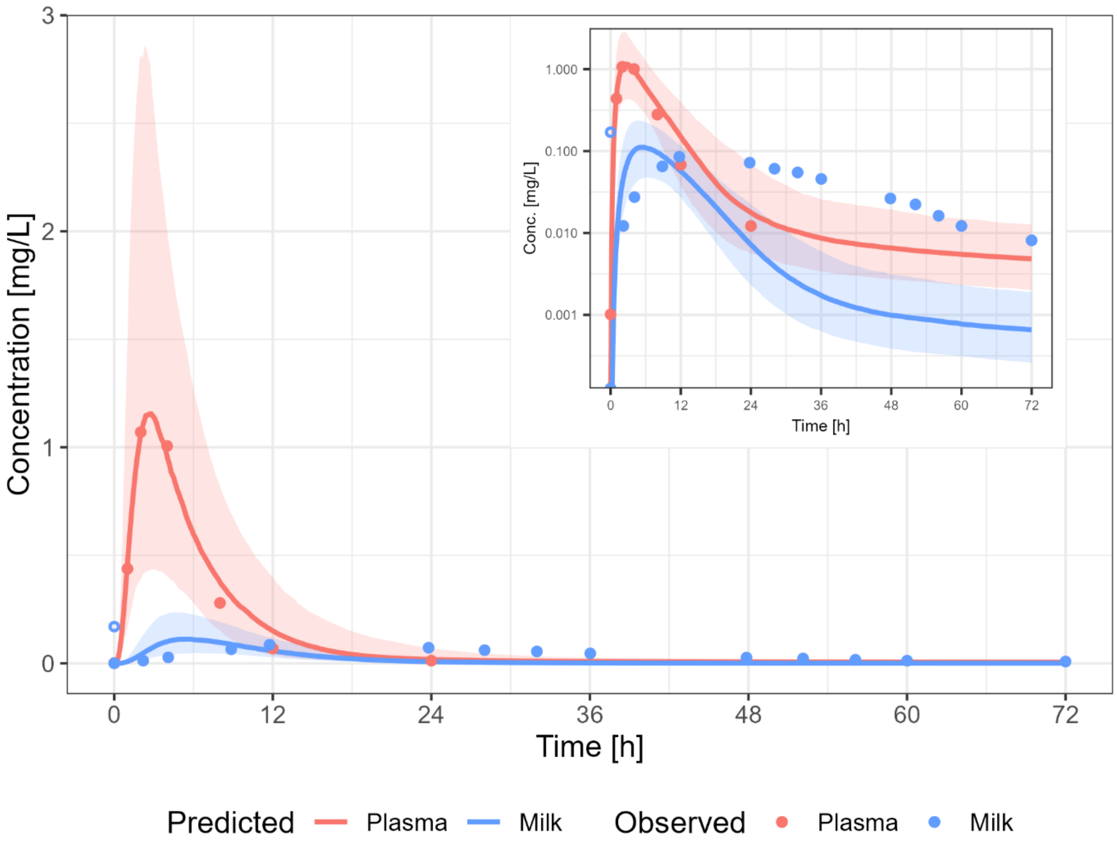

Figure S43 Predicted (Pred) versus observed (Obs) concentration-time profile after administration of 500 mg PO SD [10]

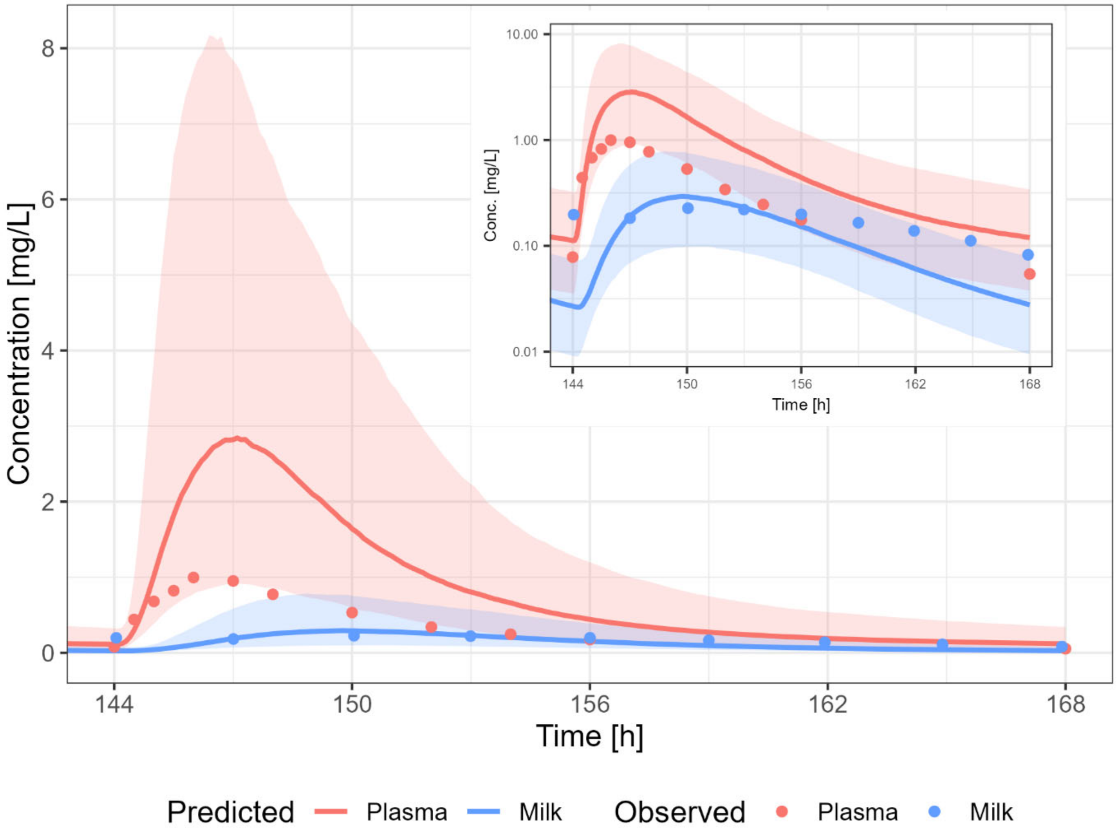

Figure S44 Predicted (Pred) versus observed (Obs) concentration-time profile after administration of 1500 mg/day PO [9]

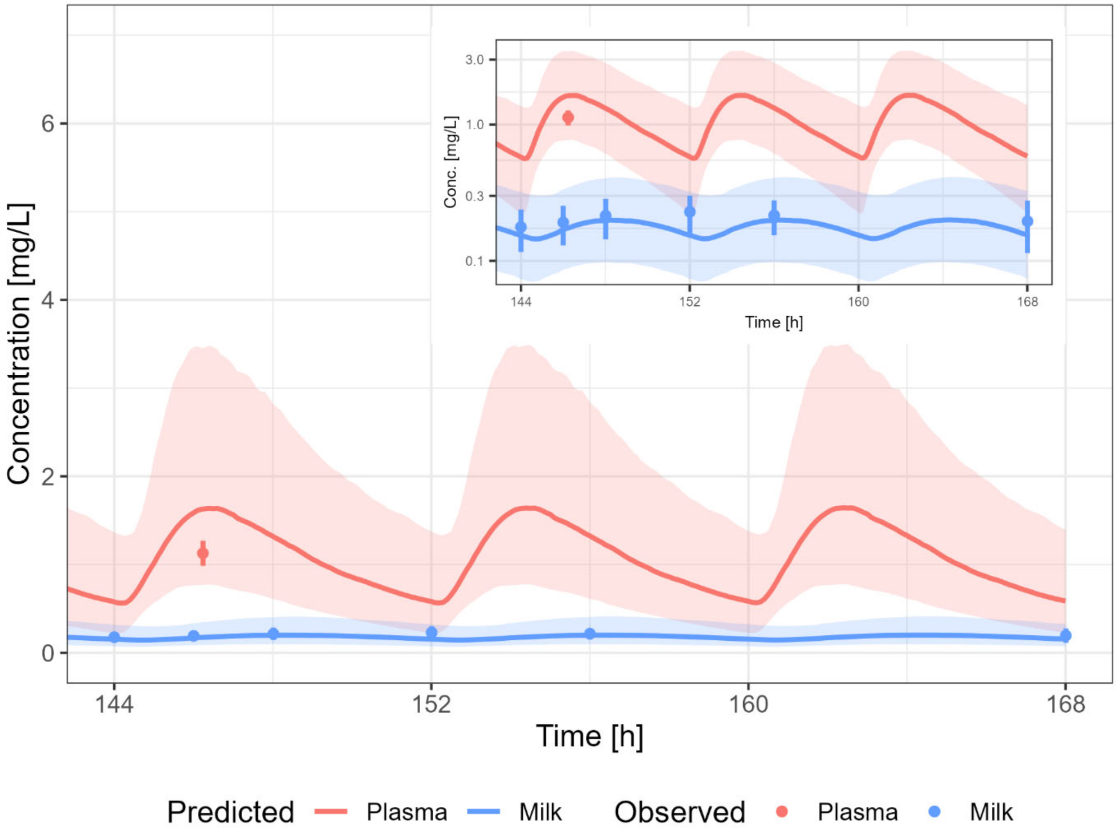

Figure S45 Predicted (Pred) versus observed (Obs) concentration-time profile after administration of 500 mg tid PO [11]

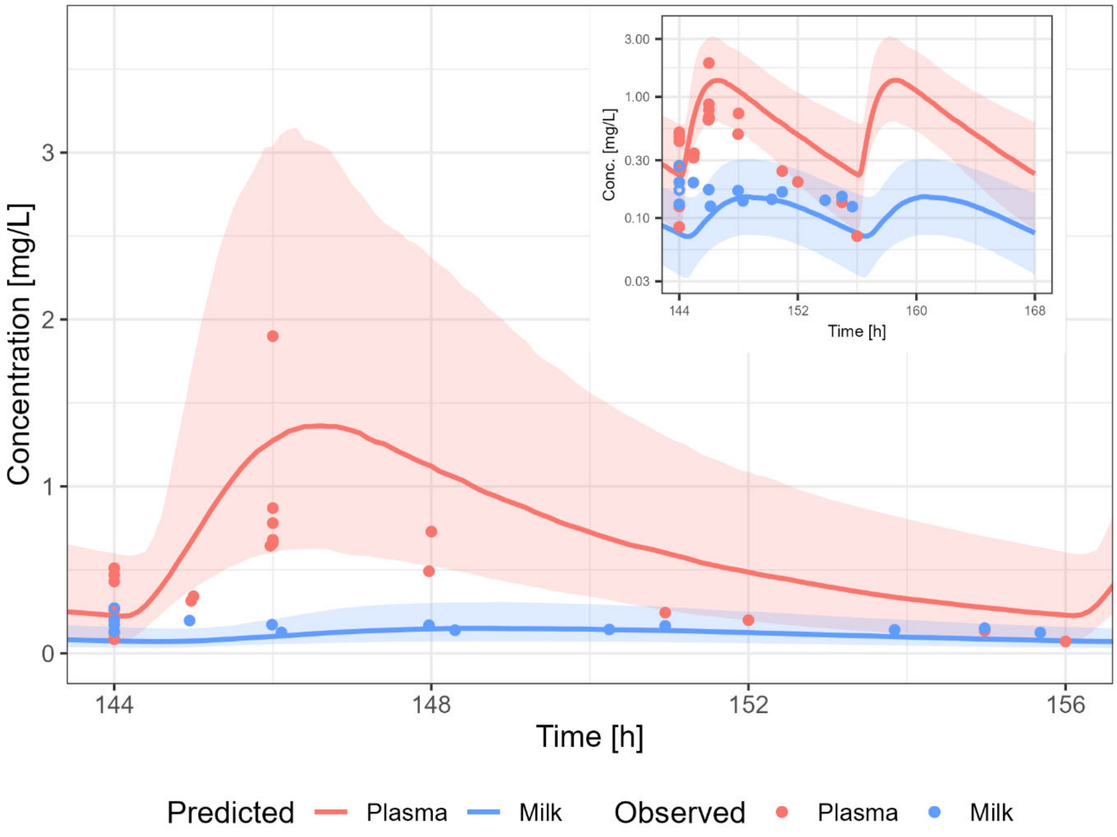

Figure S46 Predicted (Pred) versus observed (Obs) concentration-time profile after administration of 500 mg bid PO [8,10,12]

A dosing regimen of PO 500 mg bidaily was assumed to calculate the milk transfer of metformin.

Dosing interval: 12 h	Plasma	Milk
C_{max} (mg/L)	1.37	0.15
AUC (mg*h/L)	8.34	1.37
Cave (mg/L)	0.70	0.11

M/P ratio = 0.16

4.4 Estimated Pediatric exposure

A maternal dosing regimen of 500 mg bidaily was used to calculate the infant exposure. The daily infant dosage and relative infant dose (RID) for 3 months old infants were calculated using a milk intake of 150 mL/kg/day. The daily infant dosage was 0.02 mg/kg/day (RID: 0.10 %) or 0.02 mg/kg/day (RID: 0.14 %) based on the average steady-state concentration and maximum concentration in human milk, respectively.

5. Discussion

The reference PBPK model was taken from literature.

Next, the PBPK model was extended to a lactation PBPK model. The single dose simulations results in an overpredicted of the elimination phase from human milk. This might be due to transporter involvement. Potentially, organic anion transporter (OCT) could be involved in the secretion of metformin into human milk. Another possible explanation is that the complex RBC partitioning is not fully captured in the PBPK model. Importantly, this effect is less pronounced after multiple dose administration. In clinical practice, metformin is administered bidaily or thrice daily. At these administration regimens, most of the observed datapoints are within the 9-95th percentile of the population prediction. Overall, the PBPK model results in a reasonable prediction of the human milk concentrations.

The predicted M/P ratio is within the observed range, be it at the lower end of the range.

The calculated infant dosage of metformin via breastfeeding was very low, especially when compared to the maternal daily dosage.

6. Conclusions

The herein presented PBPK model adequately describes the PK of metformin in adults including breastfeeding women. In particular, it applies transport OCT1&2, MATE1 and PMAT; and glomerular filtration. The PBPK model was able to predict the human milk concentrations of metformin (M/P ratio: 0.16). The daily infant dosage was 0.02 mg/kg/day (RID: 0.10 %) or 0.02 mg/kg/day (RID: 0.14 %) based on the average steady-state concentration and maximum concentration in human milk, respectively.

7. List of Appendix and Supplementary Materials

Supplementary material 1 – ObsDataPK_OSP_reference_Metformin
Supplementary material 2 – ObsDataPK_OSP_lactation_Metformin
Supplementary material 3 – Metformin.pksim5

8. References

1. Hanke, N.; Türk, D.; Selzer, D.; Ishiguro, N.; Ebner, T.; Wiebe, S.; Müller, F.; Stopfer, P.; Nock, V.; Lehr, T. A Comprehensive Whole-Body Physiologically Based Pharmacokinetic Drug–Drug–Gene Interaction Model of Metformin and Cimetidine in Healthy Adults and Renally Impaired Individuals. *Clin. Pharmacokinet.* **2020**, *59*, 1419–1431, doi:10.1007/s40262-020-00896-w.
2. Tucker, G.; Casey, C.; Phillips, P.; Connor, H.; Ward, J.; Woods, H. Metformin Kinetics in Healthy Subjects and in Patients with Diabetes Mellitus. *Br. J. Clin. Pharmacol.* **1981**, *12*, 235–246, doi:10.1111/j.1365-2125.1981.tb01206.x.
3. Dallmann, A.; Solodenko, J.; Ince, I.; Eissing, T. Applied Concepts in PBPK Modeling: How to Extend an Open Systems Pharmacology Model to the Special Population of Pregnant Women. *CPT pharmacometrics Syst. Pharmacol.* **2018**, *7*, 419–431, doi:10.1002/PSP4.12300.
4. Dallmann, A.; Himstedt, A.; Solodenko, J.; Ince, I.; Hempel, G.; Eissing, T. Integration of Physiological Changes during the Postpartum Period into a PBPK Framework and Prediction of Amoxicillin Disposition before and Shortly after Delivery. *J. Pharmacokinet. Pharmacodyn.* **2020**, *47*, 341–359, doi:10.1007/s10928-020-09706-z.
5. Job, K.M.; Dallmann, A.; Parry, S.; Saade, G.; Haas, D.M.; Hughes, B.; Berens, P.; Chen, J.; Fu, C.; Humphrey, K.; et al. Development of a Generic Physiologically-Based Pharmacokinetic Model for Lactation and Prediction of Maternal and Infant Exposure to Ondansetron via Breast Milk. *Clin. Pharmacol. Ther.* **2022**, *111*, 1111–1120, doi:10.1002/cpt.2530.
6. Koshimichi, H.; Ito, K.; Hisaka, A.; Honma, M.; Suzuki, H. Analysis and Prediction of Drug Transfer into Human Milk Taking into Consideration Secretion and Reuptake Clearances across the Mammary Epithelia. *Drug Metab. Dispos.* **2011**, *39*, 2370–2380, doi:10.1124/dmd.111.040972.
7. Atkinson, U.C.; Begg, E.J.E.J.; Atkinson, H.C.; Begg, E.J.E.J. Prediction of Drug Distribution into Human Milk from Physicochemical Characteristics. *Clin. Pharmacokinet.* **1990**, *18*, 151–167, doi:10.2165/00003088-199018020-00005.
8. Briggs, G.G.; Ambrose, P.J.; Nageotte, M.P.; Padilla, G.; Wan, S.; G.G., B.; P.J., A.; M.P., N.; G., P.; S., W. Excretion of Metformin into Breast Milk and the Effect on Nursing Infants. *Obstet. Gynecol.* **2005**, *105*, 1437–1441, doi:10.1097/01.AOG.0000163249.65810.5b LK - http://limo.libis.be/resolver?&sid=EMBASE&issn=00297844&id=doi:10.1097%2F01.AOG.0000163249.65810.5b&atitle=Excretion+of+metformin+into+breast+milk+and+the+effect+on+nursing+infants&stitle=Obstet.+Gynecol.&title=Obstetrics+and+Gynecology&volume=105&issue=6&spage=1437&epage=1441&aulast=Briggs&aufirst=Gerald+G.&auinit=G.G.&aufull=Briggs+G.G.&coden=OBGNA&isbn=&pages=1437-1441&date=2005&auinit1=G&auinitm=G.
9. Eyal, S.; Easterling, T.R.; Carr, D.; Umans, J.G.; Miodovnik, M.; Hankins, G.D.V.; Clark, S.M.; Risler, L.; Wang, J.; Kelly, E.J.; et al. Pharmacokinetics of Metformin

during Pregnancy. *Drug Metab. Dispos.* **2010**, *38*, 833–840, doi:10.1124/dmd.109.031245.
10. Gardiner, S.J.; Kirkpatrick, C.M.J.; Begg, E.J.; Zhang, M.; Moore, M.P.; Saville, D.J. Transfer of Metformin into Human Milk. *Clin. Pharmacol. Ther.* **2003**, *73*, 71–77, doi:10.1067/mcp.2003.9.
11. Hale, T.W.; Kristensen, J.H.; Hackett, L.P.; Kohan, R.; Ilett, K.F. Transfer of Metformin into Human Milk. *Diabetologia* **2002**, *45*, 1509–1514, doi:10.1007/s00125-002-0939-x.
12. Zhang, M.; Moore, G.A.; Lever, M.; Gardiner, S.J.; Kirkpatrick, C.M.J.; Begg, E.J. Rapid and Simple High-Performance Liquid Chromatographic Assay for the Determination of Metformin in Human Plasma and Breast Milk. *J. Chromatogr. B, Anal. Technol. Biomed. life Sci.* **2002**, *766*, 175–179, doi:10.1016/s0378-4347(01)00430-3.
13. Hanke, N.; Türk, D.; Selzer, D.; Ishiguro, N.; Ebner, T.; Wiebe, S.; Müller, F.; Stopfer, P.; Nock, V.; Lehr, T. A Comprehensive Whole-Body Physiologically Based Pharmacokinetic Drug–Drug–Gene Interaction Model of Metformin and Cimetidine in Healthy Adults and Renally Impaired Individuals. *Clin. Pharmacokinet.* **2020**, *59*, 1419–1431, doi:10.1007/s40262-020-00896-w.
14. Boehringer Ingelheim Pharma GmbH & Co KG The Effect of Potent Inhibitors of Drug Transporters (Verapamil, Rifampin, Cimetidine, Probenecid) on Pharmacokinetics of a Transporter Probe Drug Cocktail Consisting of Digoxin, Furosemide, Metformin and Rosuvastatin. 2018, BI Trial No. 0352-2100. EudraCT 2017-001549-29.
15. SAMBOL, N.C.; BROOKES, L.G.; CHIANG, J.; GOODMAN, A.M.; LIN, E.T.; LIU, C.Y.; BENET, L.Z. Food Intake and Dosage Level, but Not Tablet vs Solution Dosage Form, Affect the Absorption of Metformin HC1 in Man. *Br. J. Clin. Pharmacol.* **1996**, *42*, 510–512, doi:10.1111/j.1365-2125.1996.tb00017.x.
16. Sambol, N.C.; Chiang, J.; Lin, E.T.; Goodman, A.M.; Liu, C.Y.; Benet, L.Z.; Cogan, M.G. Kidney Function and Age Are Both Predictors of Pharmacokinetics of Metformin. *J. Clin. Pharmacol.* **1995**, *35*, 1094–1102, doi:10.1002/j.1552-4604.1995.tb04033.x.
17. Sambol, N.C.; Chiang, J.; O’Conner, M.; Liu, C.Y.; Lin, E.T.; Goodman, A.M.; Benet, L.Z.; Karam, J.H. Pharmacokinetics and Pharmacodynamics of Metformin in Healthy Subjects and Patients with Noninsulin-Dependent Diabetes Mellitus. *J. Clin. Pharmacol.* **1996**, *36*, 1012–1021, doi:10.1177/009127009603601105.
18. Sirtori, C.R.; Franceschini, G.; Galli-Kienle, M.; Cighetti, G.; Galli, G.; Bondioli, A.; Conti, F. Disposition of Metformin (N,N-Dimethylbiguanide) in Man. *Clin. Pharmacol. Ther.* **1978**, *24*, 683–693, doi:10.1002/cpt1978246683.
19. Stopfer, P.; Giessmann, T.; Hohl, K.; Sharma, A.; Ishiguro, N.; Taub, M.; Zimdahl-Gelling, H.; Gansser, D.; Wein, M.; Ebner, T.; et al. Pharmacokinetic Evaluation of a Drug Transporter Cocktail Consisting of Digoxin, Furosemide, Metformin, and Rosuvastatin. *Clin. Pharmacol. Ther.* **2016**, *100*, 259–267, doi:10.1002/cpt.406.
20. Stopfer, P.; Giessmann, T.; Hohl, K.; Sharma, A.; Ishiguro, N.; Taub, M.E.; Jungnik, A.; Gansser, D.; Ebner, T.; Müller, F. Effects of Metformin and Furosemide on Rosuvastatin Pharmacokinetics in Healthy Volunteers: Implications for Their Use as Probe Drugs in a Transporter Cocktail. *Eur. J. Drug Metab. Pharmacokinet.* **2018**, *43*, 69–80, doi:10.1007/s13318-017-0427-9.
21. Tucker, G.; Casey, C.; Phillips, P.; Connor, H.; Ward, J.; Woods, H. Metformin Kinetics in Healthy Subjects and in Patients with Diabetes Mellitus. *Br. J. Clin. Pharmacol.* **1981**, *12*, 235–246, doi:10.1111/j.1365-2125.1981.tb01206.x.

22. Caillé, G.; Lacasse, Y.; Raymond, M.; Landriault, H.; Perrotta, M.; Picirilli, G.; Thiffault, J.; Spénard, J. Bioavailability of Metformin in Tablet Form Using a New High Pressure Liquid Chromatography Assay Method. *Biopharm. Drug Dispos.* **1993**, *14*, 257–263, doi:10.1002/bdd.2510140308.
23. Chen, Y.; Li, S.; Brown, C.; Cheatham, S.; Castro, R.A.; Leabman, M.K.; Urban, T.J.; Chen, L.; Yee, S.W.; Choi, J.H.; et al. Effect of Genetic Variation in the Organic Cation Transporter 2 on the Renal Elimination of Metformin. *Pharmacogenet. Genomics* **2009**, *19*, 497–504, doi:10.1097/FPC.0b013e32832cc7e9.
24. Cho, S.K.; Yoon, J.S.; Lee, M.G.; Lee, D.H.; Lim, L.A.; Park, K.; Park, M.S.; Chung, J.-Y. Rifampin Enhances the Glucose-Lowering Effect of Metformin and Increases OCT1 MRNA Levels in Healthy Participants. *Clin. Pharmacol. Ther.* **2011**, *89*, 416–421, doi:10.1038/clpt.2010.266.
25. Cho, S.K.; Kim, C.O.; Park, E.S.; Chung, J.-Y. Verapamil Decreases the Glucose-Lowering Effect of Metformin in Healthy Volunteers. *Br. J. Clin. Pharmacol.* **2014**, *78*, 1426–1432, doi:10.1111/bcp.12476.
26. Di Cicco, R.A.; Allen, A.; Carr, A.; Fowles, S.; Jorkasky, D.K.; Freed, M.I. Rosiglitazone Does Not Alter the Pharmacokinetics of Metformin. *J. Clin. Pharmacol.* **2000**, *40*, 1280–1285.
27. Ding, Y.; Jia, Y.; Song, Y.; Lu, C.; Li, Y.; Chen, M.; Wang, M.; Wen, A. The Effect of Lansoprazole, an OCT Inhibitor, on Metformin Pharmacokinetics in Healthy Subjects. *Eur. J. Clin. Pharmacol.* **2014**, *70*, 141–146, doi:10.1007/s00228-013-1604-7.
28. Gan, L.; Jiang, X.; Mendonza, A.; Swan, T.; Reynolds, C.; Nguyen, J.; Pal, P.; Neelakantham, S.; Dahlke, M.; Langenickel, T.; et al. Pharmacokinetic Drug-Drug Interaction Assessment of LCZ696 (an Angiotensin Receptor Neprilysin Inhibitor) with Omeprazole, Metformin or Levonorgestrel-Ethinyl Estradiol in Healthy Subjects. *Clin. Pharmacol. Drug Dev.* **2016**, *5*, 27–39, doi:10.1002/cpdd.181.
29. Gusler, G.; Gorsline, J.; Levy, G.; Zhang, S.Z.; Weston, I.E.; Naret, D.; Berner, B. Pharmacokinetics of Metformin Gastric-Retentive Tablets in Healthy Volunteers. *J. Clin. Pharmacol.* **2001**, *41*, 655–661, doi:10.1177/00912700122010546.
30. Hibma, J.E.; Zur, A.A.; Castro, R.A.; Wittwer, M.B.; Keizer, R.J.; Yee, S.W.; Goswami, S.; Stocker, S.L.; Zhang, X.; Huang, Y.; et al. The Effect of Famotidine, a MATE1-Selective Inhibitor, on the Pharmacokinetics and Pharmacodynamics of Metformin. *Clin. Pharmacokinet.* **2016**, *55*, 711–721, doi:10.1007/s40262-015-0346-3.
31. Jang, K.; Chung, H.; Yoon, J.; Moon, S.-J.; Yoon, S.H.; Yu, K.-S.; Kim, K.; Chung, J.-Y. Pharmacokinetics, Safety, and Tolerability of Metformin in Healthy Elderly Subjects. *J. Clin. Pharmacol.* **2016**, *56*, 1104–1110, doi:10.1002/jcph.699.
32. Johansson, S.; Read, J.; Oliver, S.; Steinberg, M.; Li, Y.; Lisbon, E.; Mathews, D.; Leese, P.T.; Martin, P. Pharmacokinetic Evaluations of the Co-Administrations of Vandetanib and Metformin, Digoxin, Midazolam, Omeprazole or Ranitidine. *Clin. Pharmacokinet.* **2014**, *53*, 837–847, doi:10.1007/s40262-014-0161-2.
33. Kim, A.; Chung, I.; Yoon, S.H.; Yu, K.-S.; Lim, K.S.; Cho, J.-Y.; Lee, H.; Jang, I.-J.; Chung, J.Y. Effects of Proton Pump Inhibitors on Metformin Pharmacokinetics and Pharmacodynamics. *Drug Metab. Dispos.* **2014**, *42*, 1174–1179, doi:10.1124/dmd.113.055616.
34. Manitpisitkul, P.; Curtin, C.R.; Shalayda, K.; Wang, S.-S.; Ford, L.; Heald, D. Pharmacokinetic Interactions between Topiramate and Pioglitazone and Metformin. *Epilepsy Res.* **2014**, *108*, 1519–1532, doi:10.1016/j.eplepsyres.2014.08.013.
35. Morrissey, K.M.; Stocker, S.L.; Chen, E.C.; Castro, R.A.; Brett, C.M.; Giacomini, K.M. The Effect of Nizatidine, a MATE2K Selective Inhibitor, on the Pharmacokinetics and Pharmacodynamics of Metformin in Healthy Volunteers. *Clin.*

Pharmacokinet. **2016**, *55*, 495–506, doi:10.1007/s40262-015-0332-9.
36. Najib, N.; Idkaidek, N.; Beshtawi, M.; Bader, M.; Admour, I.; Alam, S.M.; Zaman, Q.; Dham, R. Bioequivalence Evaluation of Two Brands of Metformin 500 Mg Tablets (Dialon ® & Glucophage ®) - in Healthy Human Volunteers. *Biopharm. Drug Dispos.* **2002**, *23*, 301–306, doi:10.1002/bdd.326.
37. Oh, J.; Chung, H.; Park, S.-I.; Yi, S.J.; Jang, K.; Kim, A.H.; Yoon, J.; Cho, J.-Y.; Yoon, S.H.; Jang, I.-J.; et al. Inhibition of the Multidrug and Toxin Extrusion (MATE) Transporter by Pyrimethamine Increases the Plasma Concentration of Metformin but Does Not Increase Antihyperglycaemic Activity in Humans. *Diabetes, Obes. Metab.* **2016**, *18*, 104–108, doi:10.1111/dom.12577.
38. Pentik�inen, P.J.; Neuvonen, P.J.; Penttil�, A. Pharmacokinetics of Metformin after Intravenous and Oral Administration to Man. *Eur. J. Clin. Pharmacol.* **1979**, *16*, 195–202, doi:10.1007/BF00562061.
39. Robert, F.; Fendri, S.; Hary, L.; Lacroix, C.; Andréjak, M.; Lalau, J. Kinetics of Plasma and Erythrocyte Metformin after Acute Administration in Healthy Subjects. *Diabetes Metab.* **2003**, *29*, 279–283, doi:10.1016/S1262-3636(07)70037-X.
40. Gormsen, L.C.; Sundelin, E.I.; Jensen, J.B.; Vendelbo, M.H.; Jakobsen, S.; Munk, O.L.; Hougaard Christensen, M.M.; Brøsen, K.; Frøkiær, J.; Jessen, N. In Vivo Imaging of Human 11 C-Metformin in Peripheral Organs: Dosimetry, Biodistribution, and Kinetic Analyses. *J. Nucl. Med.* **2016**, *57*, 1920–1926, doi:10.2967/jnumed.116.177774.
41. Somogyi, A.; Stockley, C.; Keal, J.; Rolan, P.; Bochner, F. Reduction of Metformin Renal Tubular Secretion by Cimetidine in Man. *Br. J. Clin. Pharmacol.* **1987**, *23*, 545–551, doi:10.1111/j.1365-2125.1987.tb03090.x.
42. Wang, Z.-J.; Yin, O.Q.P.; Tomlinson, B.; Chow, M.S.S. OCT2 Polymorphisms and In-Vivo Renal Functional Consequence: Studies with Metformin and Cimetidine. *Pharmacogenet. Genomics* **2008**, *18*, 637–645, doi:10.1097/FPC.0b013e328302cd41.
