# Supplementary material for: Generic Workflow to Predict Medicine Concentrations in Human Milk Using Physiologically-Based Pharmacokinetic (PBPK) Modelling—A Contribution from the ConcePTION Project
Source: Pharmaceutics. 2023 May 11;15(5):1469. doi: 10.3390/pharmaceutics15051469 (PMC10223637; doi:10.3390/pharmaceutics15051469)
Supplement: Supplementary file 1 [file pharmaceutics-15-01469-s001.zip › File S6. Evaluation_report_nevirapine.pdf]

Building and evaluation of a Physiologically-Based PharmacoKinetic (PBPK) model for **nevirapine** in adults and lactating women

Disclaimer: The research project leading to these results was conducted as part of the ConcePTION consortium. This report only reflects the personal views of the stated authors. The results of this report are only intended for research purpose, and are not intended to be used in clinical practice.

Glossary

AUC	Area Under the Curve
C_{ave}	Average concentration
CL_{re}	Reuptake clearance (i.e. from milk to blood)
CL_{sec}	Secretion clearance (i.e. from blood to milk)
C_{max}	Maximum (~peak) concentration
DID	Daily Infant Dosage (expressed for instance in mg/kg/day)
f_u	Fraction unbound in plasma
GFR	Glomerular Filtration Rate
HBD	Hydrogen Bond Donors
IV	Intravenous (administration)
$LogD_{7.2}$	Logarithm of the partition coefficient between an octanol phase and an aqueous (buffer) phase at pH 7.2
$LogD_{7.4}$	Logarithm of the partition coefficient between an octanol phase and an aqueous (buffer) phase at pH 7.4
LogP	Logarithm of the partition coefficient between an octanol phase and (unbuffered) water as aqueous phase. This is the default parameter to express lipophilicity of a substance.
MD	Multiple dose
M/P ratio	Milk-to-Plasma ratio
MW	Molecular Weight (Da)
PBPK	Physiologically-Based PharmacoKinetic [*modeling*]
pKa	Logarithm of the acid dissociation constant
PO	Oral administration
PSA	Polar Surface Area
RID	Relative Infant Dose (%)

1. Table of Contents

1. Table of Contents ***3***

2. Introduction ***4***

3. Methods ***5***

3.1 Modelling strategy **5**

3.1.1. Reference PBPK models 6
3.1.2. Lactation model 6

3.2 Data **6**

3.2.1 *In vitro* / physicochemical data 6
3.2.2 Clinical data 9

3.3 Model Parameters and assumptions **12**

3.3.1 Absorption 12
3.3.2 Distribution 12
3.3.3 Metabolism and excretion 12
3.3.4 Secretion to milk 12
3.3.5 Automated parameter optimization 13

3.4. Infant dosage calculation **13**

4. Results ***13***

4.1 Final input parameters **14**

4.2 Diagnostic plots **15**

4.3 Concentration-time profiles **17**

4.3.1 Model building 18
4.3.2 Model verification 20
4.3.3 Lactation PBPK model 25

4.4 Estimated Pediatric exposure **26**

5. Discussion ***27***

6. Conclusion ***27***

7. List of Appendix and Supplementary Materials ***28***

8. References ***28***

2. Introduction

Nevirapine (Figure S1) is a non-nucleoside reverse transcriptase inhibitor (NNRTI). Nevirapine is used in combination with nucleoside analogues for the treatment of HIV-1 infection and AIDS [1]. The recommended dose of nevirapine is 200 mg/day for 14 days, followed by 200 mg twice-daily, in combination with other retroviral agents. Absorption of nevirapine is more than 90 %, and peak concentrations around 2 μg/mL are reached within 4 h [1]. The apparent volume of distribution in healthy volunteers after IV administration is 1.21 L/kg [1]. Nevirapine is for 60 % bound to plasma proteins [1]. Metabolism of nevirapine is mainly hepatic, via cytochrome P450 enzymes to different hydroxylated metabolites [1]. CYP3A4 is the main enzyme involved, and there is a minor role for CYP2B6 and CYP2D6 pathways [2]. In addition, nevirapine is also an inducer for CYP3A4, 2D6 and 2B6 [2]. Less than 5 % is excreted unchanged in the urine. Nevirapine has a long half-life (45 h) [1].

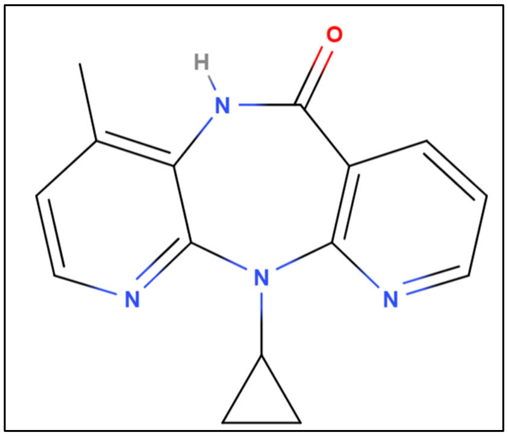

Figure S1 Chemical Structure of nevirapine

The scope of this report is to:

(a) specify the details and underlying assumptions associated with the building of physiologically-based pharmacokinetic (PBPK) models for nevirapine in adult healthy volunteers or patients, and in postpartum women during lactation.

(b) evaluate the predictive performance of these PBPK models. This is achieved by comparing model-predicted plasma or milk concentrations with corresponding clinical observations.

3. Methods

The software used for the development of PBPK models presented in this report is tabulated below:

Software	Version
PK-Sim®	v9.1
MoBi®	v9.1

3.1 Modelling strategy

In the present report, a reference PBPK model was first established for adults (patients as well as healthy volunteers), and subsequently verified against clinical pharmacokinetic data reported for nevirapine in the scientific literature.

Relevant information on the anthropometry (height, weight) was gathered from the respective clinical studies, if reported. Information on physiological parameters (e.g. blood flows, organ volumes, hematocrit) in adults is available in the PK-Sim® database.

In a second step, a lactation PBPK model was developed, based on the general workflow described by Dallmann *et al. 2018* [3–5].

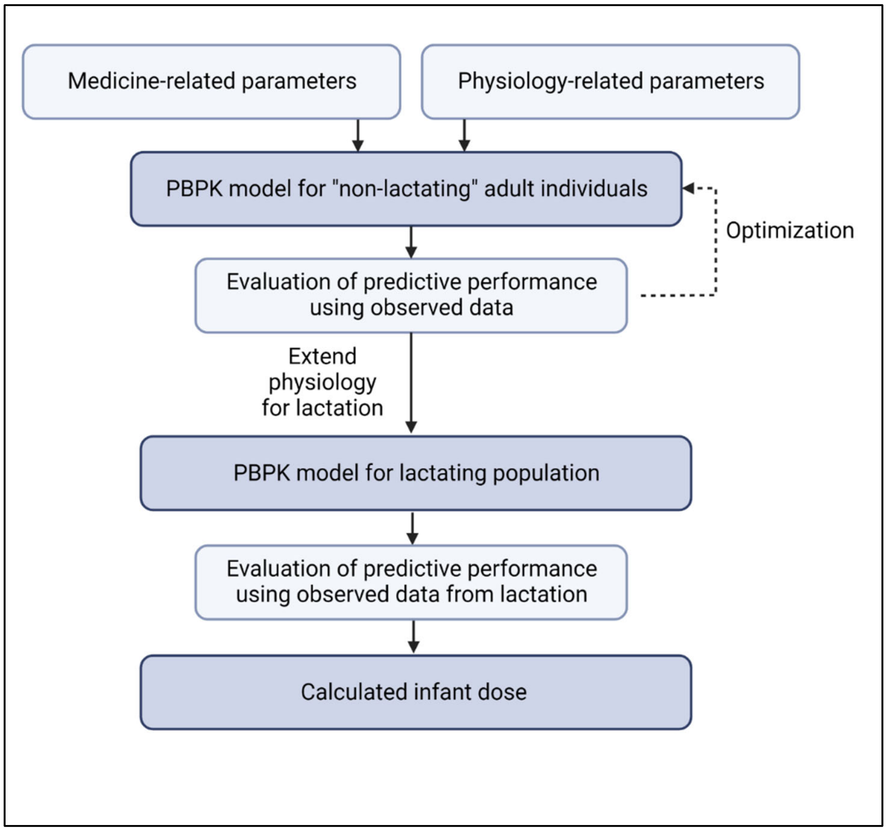

Figure S2 General workflow that was used in the present project to develop and evaluate the lactation PBPK model

Details about input data (physicochemical, *in vitro* and clinical data) can be found in section 3.2. Details about the structural models and their parameters can be found in section 3.3.

3.1.1. Reference PBPK models

The reference PBPK models were built based on studies with adult volunteers and/or adult patients, using the reported mean values for age, weight, height, and genetic background as described in each study protocol. When no information on these parameters could be found, a healthy male European individual, 30 years of age, with a body weight of 73 kg and a height of 176 cm was used.

The abundance (including population variability) of plasma proteins and enzymes/transporters that are integrated into PK-Sim are described in the publicly available 'PK-Sim Ontogeny Database Version 7.3' (PK-Sim Ontogeny Database Version 7.3).

The specific metabolic clearance of nevirapine was assumed to be via CYP3A4, CYP2D6, CYP2B6 and CYP2C9 [2]. The CYP enzymes were implemented in accordance with literature, using the PK-Sim expression database RT-PCR profiles to define their relative expression in the different organs of the body. Intrinsic clearance values were further optimized as a factor. Auto-induction of CYP3A4, CYP2D6 and CYP2B6 was included for multiple dose studies. Renal excretion was implemented as kidney plasma clearance.

Structural model selection was mainly guided by biological plausibility and by visual inspection of the predicted concentration time profiles in comparison with observed data. The generally applied acceptance criterium was less than 2-fold misprediction. Uninformed parameter values (see below) were estimated using the parameter identification module of PK-Sim®.

The predictive performance of the models was evaluated by simulating:

- Single intravenous dose studies
- Single and multiple oral dose studies
- Fed and fasted state
- Males and female subjects

For some parameters, parameter optimization was performed as described below to obtain improved concordance between predicted profiles and observed data.

3.1.2. Lactation model

After development of the reference model, the model was exported to MoBi® and a lactation PBPK model was constructed. To model the passage of nevirapine into human milk, i.e. across the blood/milk biological barrier, both the secretion (CL_{sec}) and reuptake clearance (CL_{re}) values were obtained using the empirical model developed by Koshimichi *et al. 2011* [6].

3.2 Data

3.2.1 *In vitro* / physicochemical data

A literature search was performed to collect available information on physicochemical properties of nevirapine. The obtained information from literature is summarized in **Error! Reference source not found.**. **Error! Reference source not found.** shows the parameters that were additionally used for the lactation PBPK model.

Table S1 Physicochemical parameters used as input for the nevirapine PBPK models

**Parameter**	**Value**	**Unit**	**Description**	**Source**
MW	266.2979	g/mol	Molecular weight	Drugbank [1]
pK_a	2.8 (base)		Logarithm of the acid dissociation constant	[2]
Solubility (pH 7)	100	mg/mL	Aqueous solubility	Pubchem [1]
LogP	1.93	-	Log_{10} of the partition coefficient between octanol and water (~lipophilicity)	[2]
f_u	0.4		Fraction unbound in human plasma	
Renal clearance – plasma clearance	0.001	L/h/kg		
Hepatic clearance:<br>- 3A4<br>- 2B6<br>- 2D6<br>- Other (2C9)	 - 1.74<br>- 0.55<br>- 0.33<br>- 0.55	L/h	Intrinsic clearance – first order	
Induction:<br>EC50<br>Emax:<br>- 3A4<br>- 2B6<br>- 2D6	 1.00<br><br>1.49<br>4.00<br>1.54	µmol/L	Auto-induction	

Table S2 Physicochemical parameters used as input for the lactation PBPK model of nevirapine

Parameter	Value	Unit	Description	Source
Milk logP[a]	1.93	-	Log_{10} of the partition coefficient between octanol and water	[2]
HBD	1.00		Hydrogen bond donors	Pubchem
PSA	58.10	$Å^2$	Polar surface area	Pubchem

[a] Milk logP is Log_{10} of the partition coefficient between octanol and water and is used as input for the calculations in the postpartum model (see equations below). In theory, this value is identical to the logP specified in Table S1. However, in some PBPK models, logP (Table S1) might be optimized using parameter identication. Therefore, it was chosen to use a separate parameter (i.e. Milk logP) to represent the logP used as input for the equations in the postpartum model.

The default equations for free fraction in human milk and logD that were implemented in the spatial structure building block that was developed for the postpartum women are described below. Alternatively, these values can be overwritten by values calculated elsewhere (e.g. MarvinSketch) or determined *in vitro.*

The free fraction in human milk was calculated with the equations proposed by Atkinson and Begg [7], as follows:

$$f_{u\_skimmed\ milk} = \frac{f_u \times 0.448}{(0.000694^{0.448} + f_u^{0.448})}$$

$$P_{milk} = 10^{(-0.88+1.29 \times \mathrm{logD7.2})}$$

$$\text{Total free fraction in milk} = \frac{1}{\left(\frac{0.955}{f_{u\_skimmed\ milk}} + 0.045 \times P_{milk}\right)}$$

Where: *f_u skimmed milk:* binding to proteins in milk; P_{milk}: partitioning between aqueous and lipid phase of milk; Total free fraction in milk: 'total' free fraction, i.e. accounting for both protein and lipid binding processes.

LogD values taking into account up to three pka values (as provided in the compound building block), were calculated as follows:

$$LogD = \mathrm{Log}\,P + Log_{10}(logD_{factor})$$

With Milk logP (Table S2) as input for logP

$$LogD_{factor} = \mathrm{K}_1 + (\mathrm{K}_2 + \mathrm{K}_3 + \mathrm{K}_4) \times \mathrm{base}^1 + \mathrm{K}_5 \times \mathrm{base}^{\max(\mathrm{CT}_0+\mathrm{CT}_1;-\mathrm{CT}_0-\mathrm{CT}_1)} + \mathrm{K}_6 \times \mathrm{base}^{\max(CT_0+CT_2;-CT_0-CT_2)} + \mathrm{K}_7 \times \mathrm{base}^{\max(\mathrm{CT}_2+\mathrm{CT}_1;-\mathrm{CT}_2-\mathrm{CT}_1)} + \mathrm{K}_8 \times \mathrm{base}^{\max(\mathrm{CT}_0+\mathrm{CT}_1+\mathrm{CT}_2;-\mathrm{CT}_0-\mathrm{CT}_1-\mathrm{CT}_2)}$$

$K_1 = F_1 \times F_2 \times F_3$
$K_2 = (1 - F_1) \times F_2 \times F_3$
$K_3 = F_1 \times (1 - F_2) \times F_3$
$K_4 = F_1 \times F_2 \times (1 - F_3)$
$K_5 = (1 - F_1) \times (1 - F_2) \times F_3$
$K_6 = (1 - F_1) \times F_2 \times (1 - F_3)$
$K_7 = (1 - F_1) \times F_2 \times (1 - F_3)$
$K_8 = (1 - F_1) \times (1 - F_2) \times (1 - F_3)$

F1 = CT0 != CT_NEUTRAL ? 1/(1+10^(CT0*(pKa_0- pH))) : 1
F2 = CT1 != CT_NEUTRAL ? 1/(1+10^(CT1*(pKa_1- pH))) : 1
F3 = CT2 != CT_NEUTRAL ? 1/(1+10^(CT2*(pKa_2- pH))) : 1

With CT = compound type (-1: acid; +1: base; 0: neutral), and pH = 7.2 or 7.4 respectively for $logD_{7.2}$ *and* $logD_{7.4}$

The transports that were added in the passive transport building block for 'transfer to milk' and 'transfer from milk' are based on secretion and reuptake and clearance values, Cl_{sec} and Cl_{re}, which were calculated according to the empirical equations proposed by Koshimichi et al. 2011 [6], as follows:

$$\mathrm{Log}\, CL_{re} = 2.793 + 0.179 \times LogP - 0.132 \times HBD$$

$$\mathrm{Log}\, CL_{sec} = 3.367 \times Log10(MW) - 0.164 \times (LogP - LogD) - 0.015 \times PSA - 3.912$$

3.2.2 Clinical data

Literature searches were performed to collect available data on nevirapine in adults and breastfeeding women. The nevirapine PBPK model was developed using different clinical studies with pharmacokinetic (PK) blood sampling. First, a clinical trial with single intravenous and oral solution administration of two doses was taken into account to estimate solubility and clearance parameters [8]. Subsequently, a multiple dose study with a high oral dose (400 mg/day) was used to further optimize solubility [9]. The other arms of both studies were used for evaluation of the predictive performance. In addition, 5 other studies with single and multiple oral administrations in males and females were used for model verification [10–14].

The evaluation of the predictive performance of the nevirapine lactation PBPK model was performed using 8 different studies were nevirapine was administered as an oral dose of 200 mg bidaily to lactating women [15–22]. The women were between 7 days and 6 months postpartum. The samples were assumed to be through samples if the exact timing was not reported in the articles.

Detailed information and data from the studies used for model building, verification, and lactation model can be found in Supplementary material 1 and 2.

3.2.2.1 Model building

The studies that were used for model building are shown in Table S3 (**training data**).

Table S3 Summary of studies used for PBPK model building of nevirapine in reference populations

Study ID	Reference	Arm/treatment/information used for model building
Lamson 1999	[8]	12 subjects received 15 mg IV (single dose)
Lamson 1999	[8]	12 subjects received 50 mg PO solution (single dose)
Lamson 1999	[8]	24 subjects received 200 mg PO solution (single dose)
Kappelhoff 2015	[9]	205 subjects received 400 mg/day PO (multiple dose)

Table S4 Demographic information

Study ID	Reference	Number of subjects (female ratio)	Age (year)	Weight (kg)
Lamson 1999	[8]	12 (0)<br>24 (0)	29.8 (20-49)<br>26.9 (18-43)	80.6 (67.7-96.8)<br>78.5 (58.8-95.5)
Kappelhoff 2015	[9]	205 (0.38)	35 (-)	-

3.2.2.2 Model verification

The studies that were used to evaluate the predictive performance of the PBPK model are shown in

Table S5 (**verification data**).

Table S5 Summary of studies used for model verification of nevirapine PBPK model in reference population

Study ID	Reference	Arm/treatment/information used for model verification
Lamson 1999	[8]	12 subjects received 50 mg PO tablet (single dose)
Lamson 1999	[8]	24 subjects received 200 mg PO tablet (single dose)
Kappelhoff 2015	[9]	373 subjects received 200 mg twice-daily PO (multiple dose)
Fan-Havard 2013	[10]	10 subjects received 200 mg PO (single dose)
Fan-Havard 2013	[10]	10 subjects received 200 mg twice-daily PO (multiple dose)
Marier 2007	[11]	64 subjects received 200 mg PO (single dose)

Ribera 2001	[12]	5 subjects received 200 mg twice-daily PO (multiple dose)
Riska 1999	[13]	8 subjects received 50 mg PO at steady-state (multiple dose)
Von Hentig 2006	[14]	14 males received 200 mg twice-daily PO (multiple dose)
Von Hentig 2006	[14]	13 females received 200 mg twice-daily PO (multiple dose)

Table S6 Demographic information

**Study ID**	**Reference**	**Number of subjects (female ratio)**	**Age (year)**	**Weight (kg)**
Lamson 1999	[8]	12 (0)<br>24 (0)	29.8 (20-49)<br>26.9 (18-43)	80.6 (67.7-96.8)<br>78.5 (58.8-95.5)
Kappelhoff 2015	[9]	373 (0.39)	36 (-)	-
Fan-Havard 2013	[10]	10 (0.80)<br>10 (0.50)	25 (21-43)<br>32 (28-44)	68 (59-100)<br>52 (42-66)
Marier 2007	[11]	64 (0)	36 (21-52)	73.8 (60.6-89.9)
Ribera 2001	[12]	5 (0)	-	-
Riska 1999	[13]	11 (-)	- (20-34)	- (62.6-84.4)
Von Hentig 2006	[14]	14 (0)<br>13 (1)	33.47 (27-58)	58.8 (44-84)

3.2.2.3 Lactation PBPK model

Table S7 shows the study that was used for the lactation PBPK model.

Table S7 Summary of study used for PBPK model development of nevirapine in lactating women

**Study ID**	**Publication**	**Arm/treatment/information used for model building and verification**
Bennetto-Hood 2007	[15]	1 woman (8 weeks postpartum) received PO 200 mg bidaily (multiple dose)
Giuliano 2007	[16]	40 women (7 days postpartum) received PO 200 mg bidaily (multiple dose)
Mirochnick 2009	[17]	Women (2/6/14/24 weeks postpartum) received PO 200 mg bidaily (multiple dose)
Olagunju 2015	[18]	5 women (15-183 days postpartum) received PO 200 mg bidaily (multiple dose)
Olagunju 2016	[19]	28 women (1.4-73.9 weeks postpartum) received PO 200 mg bidaily (multiple dose)
Palombi 2012	[20]	66 women (1/3/6 month postpartum) received PO 200 mg bidaily (multiple dose)
Shapiro 2005	[21]	20 women (2/5 months postpartum) received PO 200 mg bidaily (multiple dose)
Shapiro 2013	[22]	15 women (30 days postpartum) received PO 200 mg bidaily (multiple dose)

3.3 Model Parameters and assumptions

3.3.1 Absorption

There is no clear difference in absorption between an oral solution and a tablet [8]. Therefore, solution was used as formulation in all the studies. Intestinal permeability was calculated from lipophilicity according to PK-Sim standard methods. Solubility of nevirapine is pH dependent, and literature values were highly variable. Therefore, solubility was estimated during parameter optimization [8,9].

3.3.2 Distribution

An important parameter influencing the distribution of a compound is lipophilicity. Lipophilicity was taken from literature [2]. The tissue partition coefficients (Kp) calculation was according to 'Rodgers and Rowland' and the cellular permeability calculation was 'PK-Sim Standard'.

3.3.3 Metabolism and excretion

The final model applies metabolism by CYP enzymes (CYP3A4, CYP2B6, CYP2D6, and CYP2C9) and renal excretion. For CYP enzymes, the first order intrinsic clearance values were calculated via the well-stirred model based on literature [2], and further optimized as factor via parameter identification based on observed clinical data [8]. Renal elimination was implemented as kidney plasma clearance, based on the value reported in literature [2].

3.3.4 Secretion to milk

To model the transfer process of nevirapine into human milk, both the secretion (CL_{sec}) and reuptake clearance (CL_{re}) were calculated using the empirical equations developed by Koshimichi *et al.* 2011 (see **Error! Reference source not found.**) [4].

First, in MoBi®, a spatial structure for the postpartum women was constructed, similar to the workflow from Dallmann *et al.* 2018 [2]. Here, breasts were added as a compartment. In addition, the human milk was connected to the plasma subcompartment of the breasts. The human milk volume was specified as 0.5 L to represent the structure of Koshimichi *et al.* 2011, and a geometric standard deviation of 1.16 was assumed in the population. The free fraction in human milk, and logD values were implemented as the equations described previously. The transfer between plasma and milk was defined as two kinetic processes (transfer to milk and transfer from milk) under passive transports (see below). Next, the simulation was combined with the postpartum population from Job *et al.* 2021 in PK-Sim to account for the postpartum physiology [3].

<u>*Kinetics*</u>

Transfer to milk

$$\frac{dNmilk}{dt} = Cplasma \times f_u \times CL_{sec}$$

where Cplasma is the concentration in plasma (in breast compartment), f_u is the free fraction in plasma and CL_{sec} is the secretion clearance.

Transfer from milk

$$\frac{dNplasma}{dt} = Cmilk \times f_u \times CL_{re}$$

where Cmilk is the concentration in human milk, f_u is the total free fraction in human milk (protein and lipid) and CL_{re} is the reuptake clearance.

The median simulated plasma and human milk concentration-time profiles can be used to calculate the M/P ratio as follows:

$$M/P\ ratio\ = \frac{AUC_{milk}}{AUC_{plasma}}$$

3.3.5 Automated parameter optimization

The following table depicts the results of the final parameter identification according to the different clinical studies.

a) Lamson et al. (1999) 15 mg IV (single dose); Lamson et al. (1999) 50 mg PO solution (single dose); Lamson et al. (1999) 200 mg PO solution (single dose) [8]

Model parameter	Optimized value	Unit
Solubility at reference pH	264.58	mg/L
Intrinsic clearance factor	0.22	

b) Kappelhoff et al. 2015 400 mg/day PO (multiple dose)

Model parameter	Optimized value	Unit
Solubility at reference pH	797.00	mg/L

3.4. Infant dosage calculation

Infant dosage via human milk was then calculated based on the predicted (average and maximal) steady-state nevirapine concentration in human milk, as well as the daily milk intake volume. The daily infant dosage was then compared to the maternal dosage, resulting in the relative infant dose (RID).

$$Daily\ infant\ dosage = C_{average} * 150\ \frac{mL}{kg\ .day}$$

$$Daily\ infant\ dosage = C_{max} * 150\ \frac{mL}{kg\ .day}$$

$$Relative\ infant\ dose\ (RID) = \frac{Infant\ dosage}{Maternal\ dosage} * 100\ \%$$

4. Results

Both the reference and postpartum PBPK model of nevirapine were developed and verified with clinical PK data.

The model was evaluated covering studies including in particular:

- Intravenous and oral administration
- Single and multiple doses
- A dose range from 15 up to 400 mg
- Males and females subjects

The model describes the metabolism of nevirapine via Cytochrome P450 enzymes and renal excretion. Moreover, secretion and reuptake to human milk were described by CL_{sec} and CL_{re}.

The next sections show:

- The final model parameters for the building blocks: section 4.1
- The overall predictive performance: section 4.2
- The simulated versus observed concentration-time profiles for the clinical studies used for model building and for model verification: section 4.3

4.1 Final input parameters

The compound values of the final postpartum PBPK model for nevirapine are illustrated below.

Physicochemical parameters

Parameter	Value	Unit	Source
MW	266.2979	g/mol	Drugbank
pKa	2.80	-	Drugbank
Solubility	797.00	mg/mL	Parameter identification
Lipophilicity	1.93	-	[2]
f_u	0.40	-	Drugbank
Small molecule (Y/N)	Y	-	
Plasma protein binding partner	Albumin		

Calculation methods

Name	Value
Tissue partition coefficients	Rodgers and Rowland
Cellular permeabilities	PK-Sim Standard

AMDE-related parameters

Parameter	Value	Unit	Source
Intestinal permeability	1.66E-5	cm/min	Default value
Intrinsic clearance – first order (CYP3A4)	0.38	L/h	Parameter identification
Intrinsic clearance – first order (CYP2B6)	0.12	L/h	Parameter identification
Intrinsic clearance – first order (CYP2D6)	0.07	L/h	Parameter identification
Intrinsic clearance – first order (CYP2C9)	0.12	L/h	Parameter identification

Kidney plasma clearance	1.00E-3	L/h/kg	[2]
Auto-induction (CYP3A4):<br>- EC50<br>- Emax	- 1.00<br>- 1.49	µmol/L	[2]
Auto-induction (CYP2B6):<br>- EC50<br>- Emax	- 1.00<br>- 4.00	µmol/L	[2]
Auto-induction (CYP2D6):<br>- EC50<br>- Emax	- 1.00<br>- 1.54	µmol/L	[2]

Formulation-related parameters
Type: Solution

Physicochemical and physiological parameters relevant to the lactation model

Parameter	Value	Unit	Source
Milk logP	1.93	Log units	[2]
HBD	1.00	-	Pubchem
PSA	58.10	Å^2	Pubchem
CL_{sec}	0.04	L/min	Default
CL_{re}	0.02	L/min	Default
*fu*_skimmed milk[a]	0.95	-	Default
Pmilk[b]	40.71	-	Default
Total free fraction in milk[c]	0.35	-	Default
$logD_{7.2}$	1.93	Log units	Default
$logD_{7.4}$	1.93	Log units	Default

[a] binding to proteins in milk; [b] partitioning between aqueous and lipid phase of milk; [c] total free fraction, accounting for both protein and lipid binding

4.2 Diagnostic plots

The geometric mean fold errors (GMFE) on AUC and Cmax were 1.07 and 1.20 for the model building dataset, and 1.13 and 1.34 for the model verification dataset.
The following plot shows the predictive performance graph for Cmax and AUC of nevirapine for the PBPK model performance of all data used.
Predicted over observed ratio values of all data listed in section 3.2.2 are presented below.

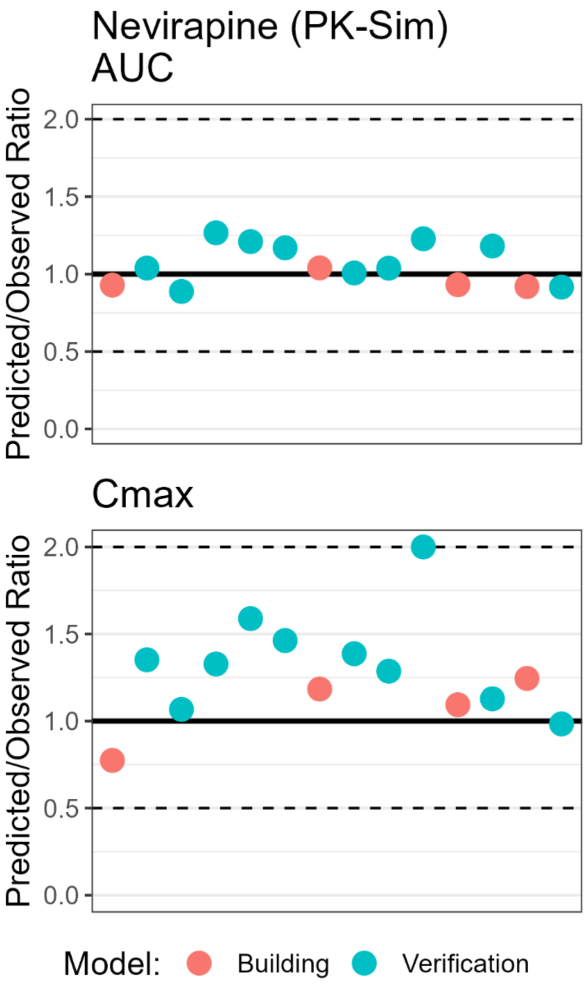

Figure S3 Predicted over observed ratio profile

Table S8 Ratio between the predicted and observed pharmacokinetic parameters of nevirapine in different dosing regimens for model building

Study ID/ Reference	Dose/ Route	AUC_{obs} (mg*h/L)	AUC_{pred} (mg*h/L)	Fold error	$C_{max\ obs}$ (mg/L)	$C_{max\ pred}$ (mg/L)	Fold error
Lamson 1999	15 mg IV SD	7.88	7.32	0.93	0.31	0.24	0.77
Lamson 1999	50 mg PO SD (1)	31.59	29.05	0.92	0.49	0.61	1.24
Lamson 1999	200 mg PO SD (1)	117.21	121.93	1.04	2.12	2.51	1.18
Kappelhoff 2015	400 mg PO MD	125.29	116.70	0.93	7.91	8.65	1.09

Table S9 Ratio between the predicted and observed pharmacokinetic parameters of nevirapine in different dosing regimens used for model verification

Study ID/ Reference	Dose/ Route	AUC_{obs} (mg*h/L)	AUC_{pred} (mg*h/L)	Fold error	$C_{max\ obs}$ (mg/L)	$C_{max\ pred}$ (mg/L)	Fold error
Lamson 1999	50 mg PO SD (2)	31.71	29.05	0.92	0.62	0.61	0.98
Lamson 1999	200 mg PO SD (2)	121.03	121.93	1.01	1.81	2.51	1.39
Kappelhoff 2015	200 mg PO MD	65.535	58.20	0.89	6.56	7.00	1.07
Fan-Havard 2013	200 mg SD	125.42	154.01	1.23	1.43	2.86	2.00
Fan-Havard 2013	200 mg MD[23]	72.32	75.14	1.04	6.99	9.45	1.35
Marier 2007	200 mg PO SD	85.76	89.06	1.04	2.06	2.65	1.29
Ribera 2001	200 mg PO MD	45.73	57.92	1.27	5.28	7.01	1.33
Riska 1999	50 mg PO MD	115.77	136.76	1.18	4.14	4.67	1.13
Von Hentig 2006	200 mg PO MD female	56.60	68.48	1.21	5.42	8.61	1.59
Von Hentig 2006	200 mg PO MD male	50.21	58.73	1.17	4.82	7.05	1.46

4.3 Concentration-time profiles

Simulated versus observed concentration-time profiles of all data listed in section 3.2.2 are presented below. The original.pksim5 are provided in Supplemented material 3.

4.3.1 Model building

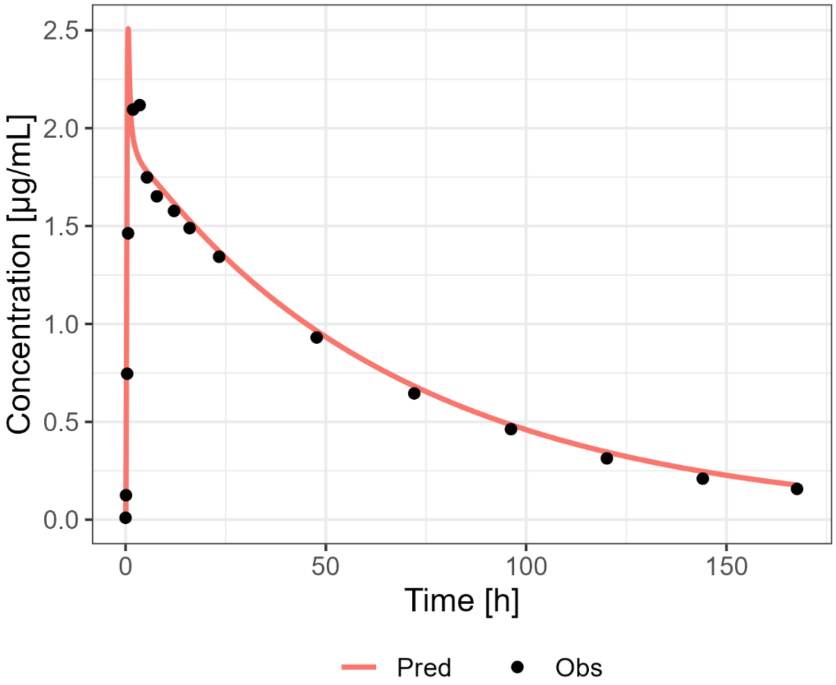

Figure S4 Predicted (Pred) versus observed (Obs) concentration-time profile after administration of 200 mg PO SD [8]

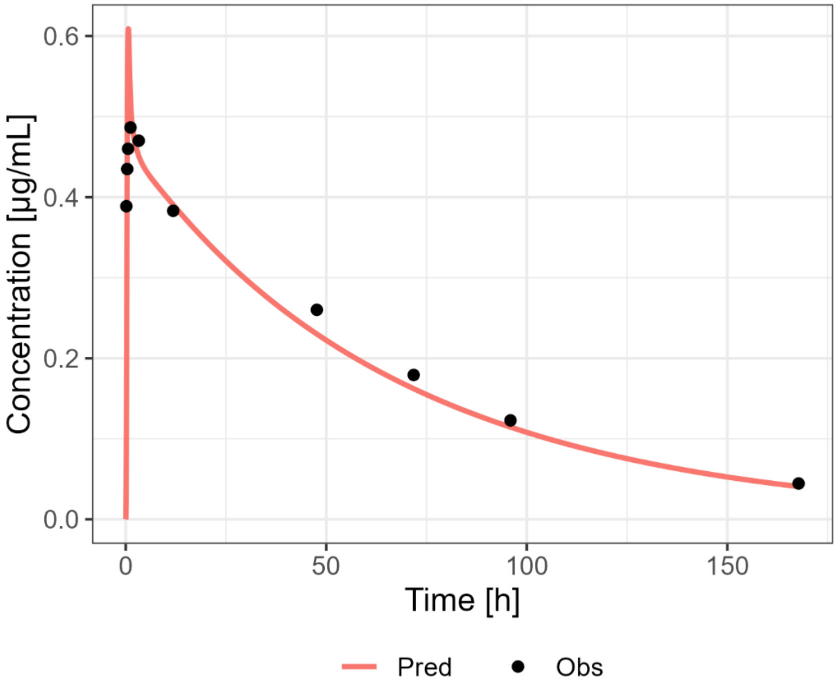

Figure S5 Predicted (Pred) versus observed (Obs) concentration-time profile after administration of 50 mg PO [8]

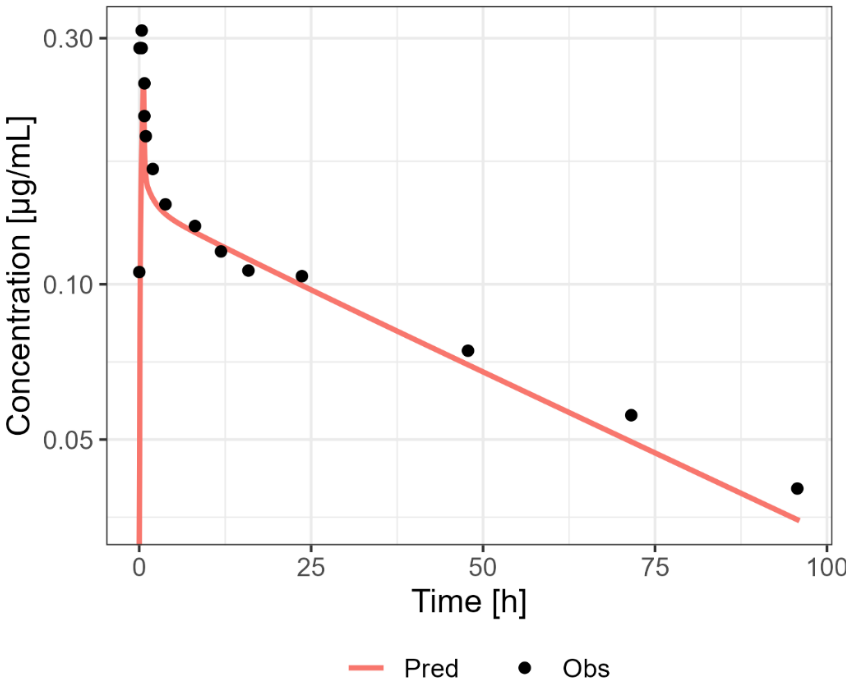

Figure S6 Predicted (Pred) versus observed (Obs) concentration-time profile after administration of 15 mg IV SD [8]

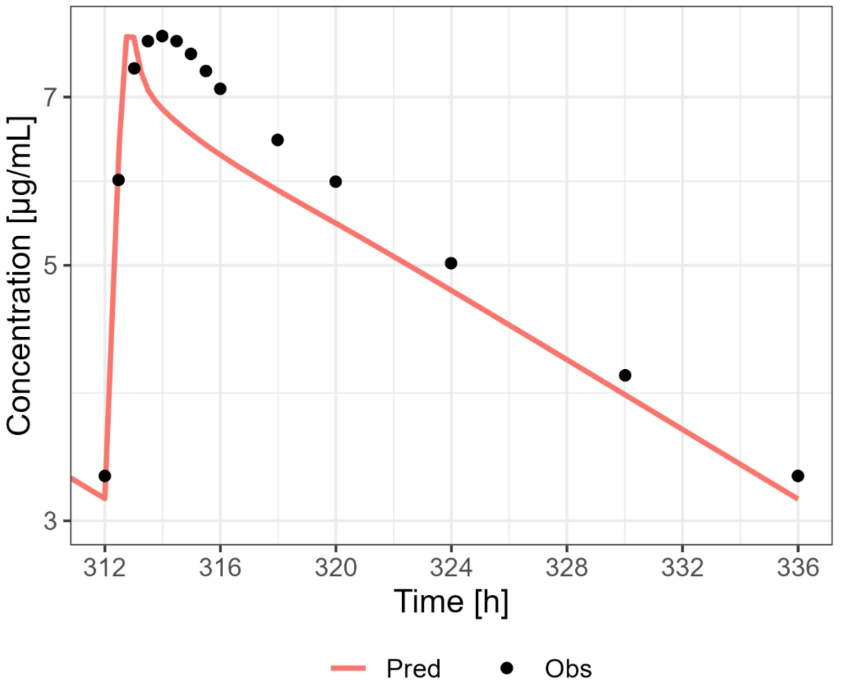

Figure S7 Predicted (Pred) versus observed (Obs) concentration-time profile after administration of 400 mg daily MD [9]

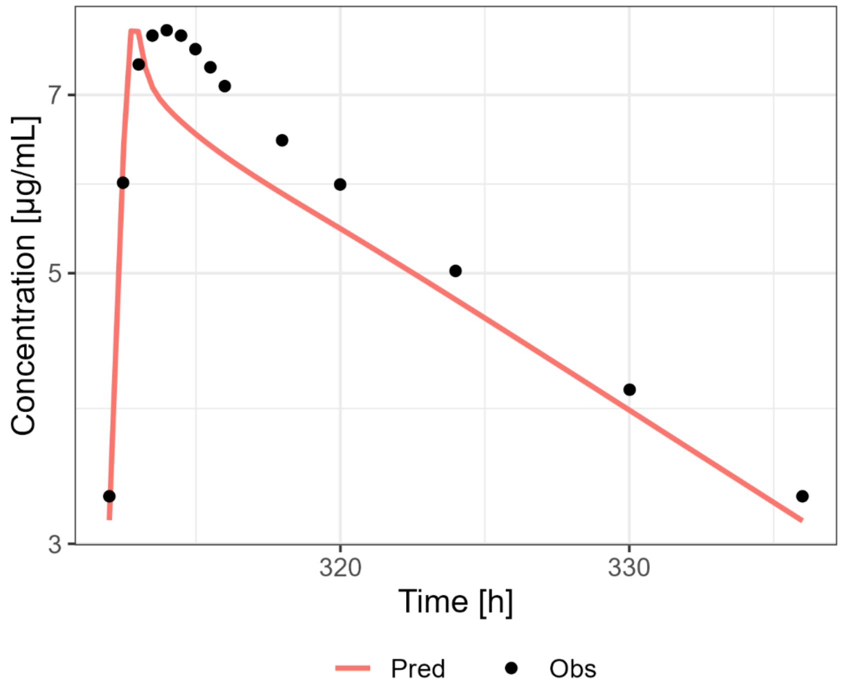

Figure S8 Predicted (Pred) versus observed (Obs) concentration-time profile after administration of 400 mg daily MD [9]

4.3.2 Model verification

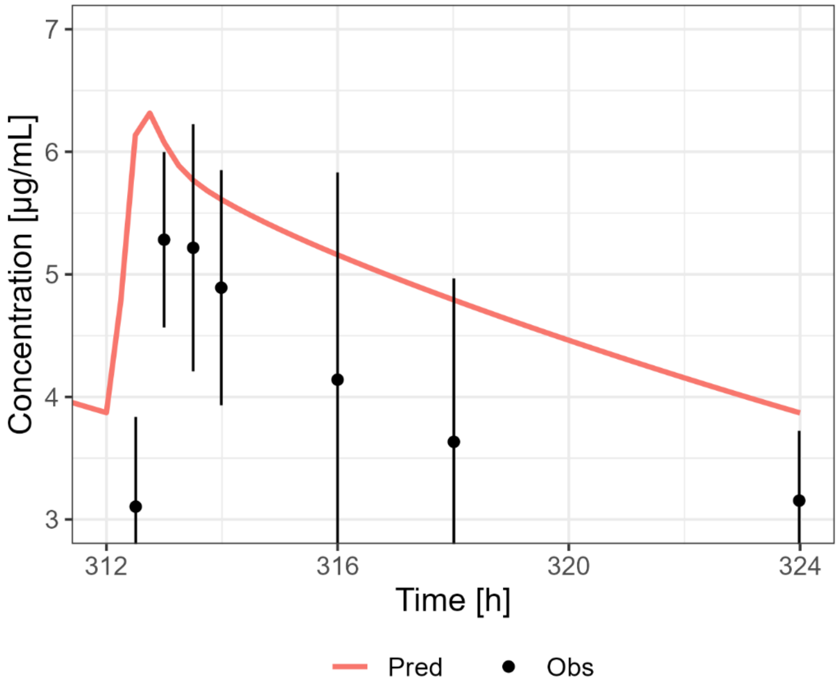

Figure S9 Predicted (Pred) versus observed (Obs) concentration-time profile after administration of 200 mg bid PO MD [12]

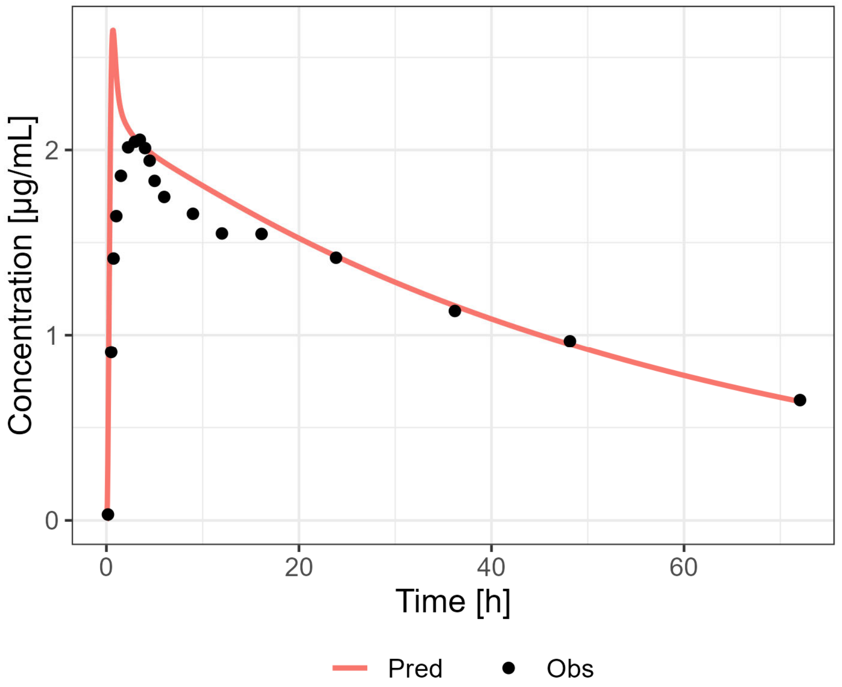

Figure S10 Predicted (Pred) versus observed (Obs) concentration-time profile after administration of 200 mg PO SD [11]

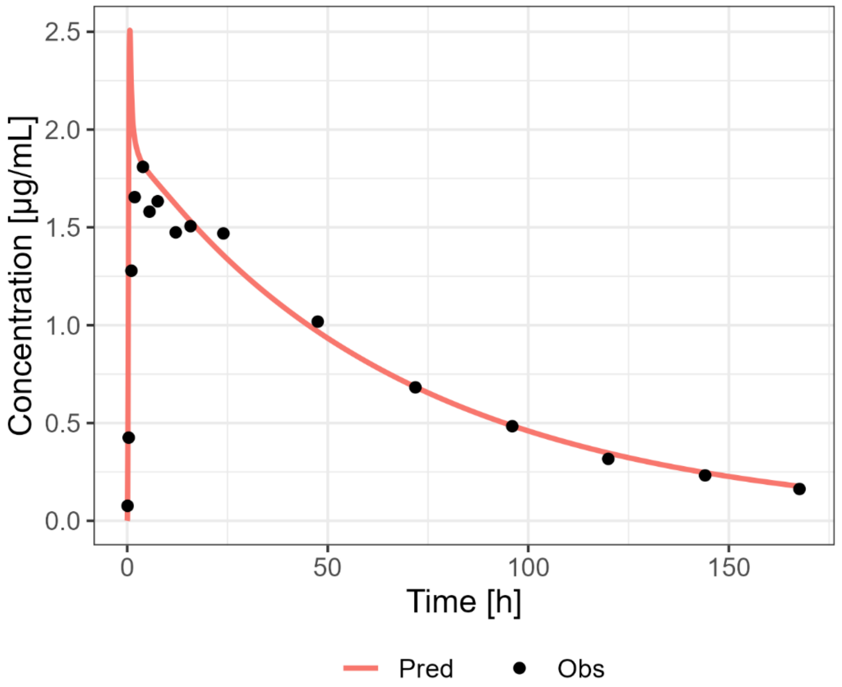

Figure S11 Predicted (Pred) versus observed (Obs) concentration-time profile after administration of 200 mg PO SD [8]

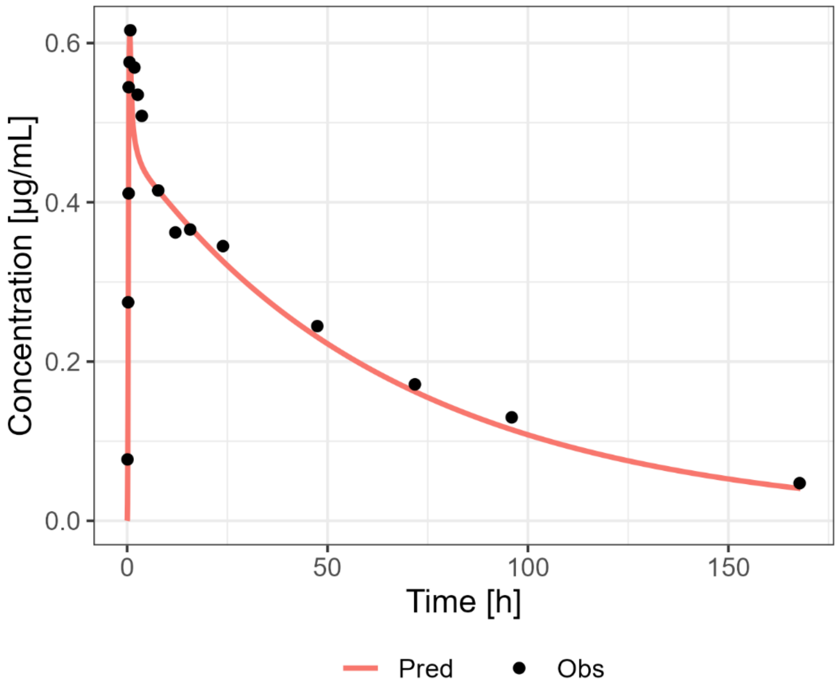

Figure S12 Predicted (Pred) versus observed (Obs) concentration-time profile after administration of 50 mg PO SD [8]

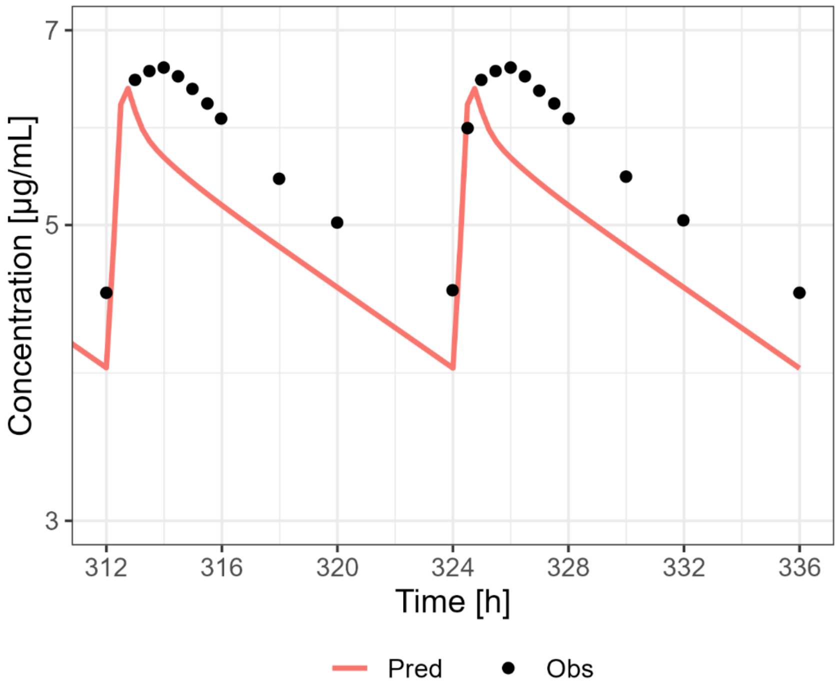

Figure S13 Predicted (Pred) versus observed (Obs) concentration-time profile after administration of 200 mg bid MD [9]

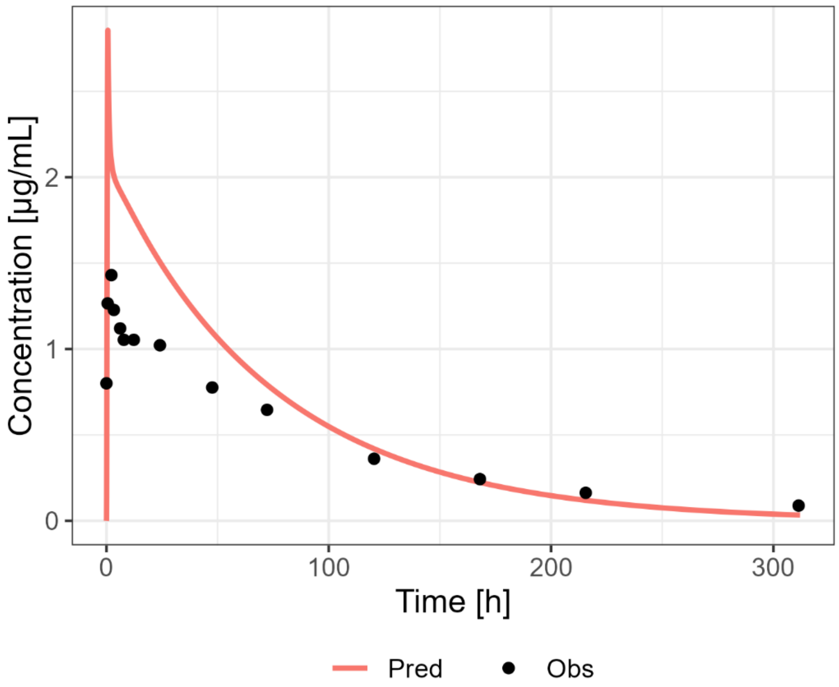

Figure S14 Predicted (Pred) versus observed (Obs) concentration-time profile after administration of 200 mg PO SD [10]

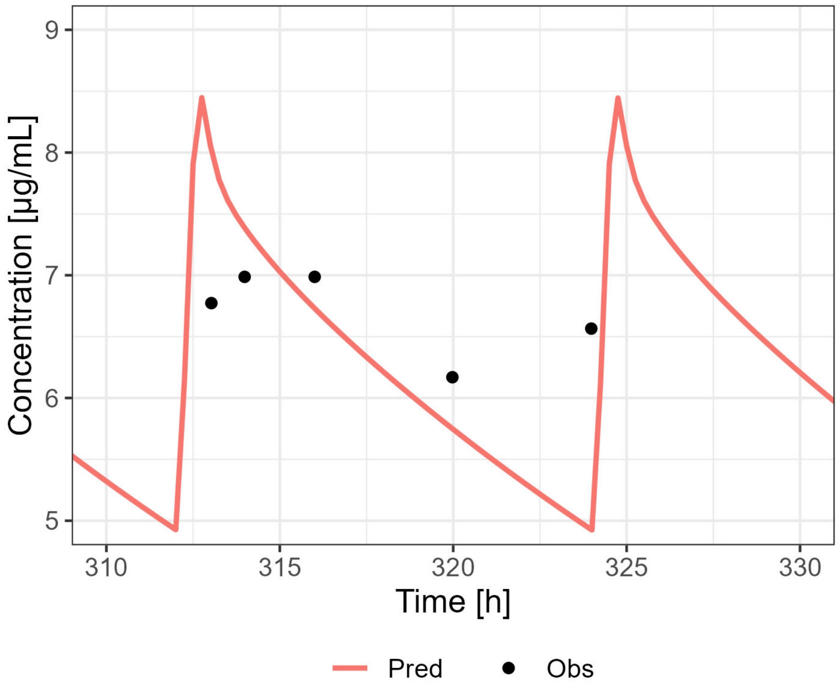

Figure S15 Predicted (Pred) versus observed (Obs) concentration-time profile after administration of 200 mg bid PO MD [10]

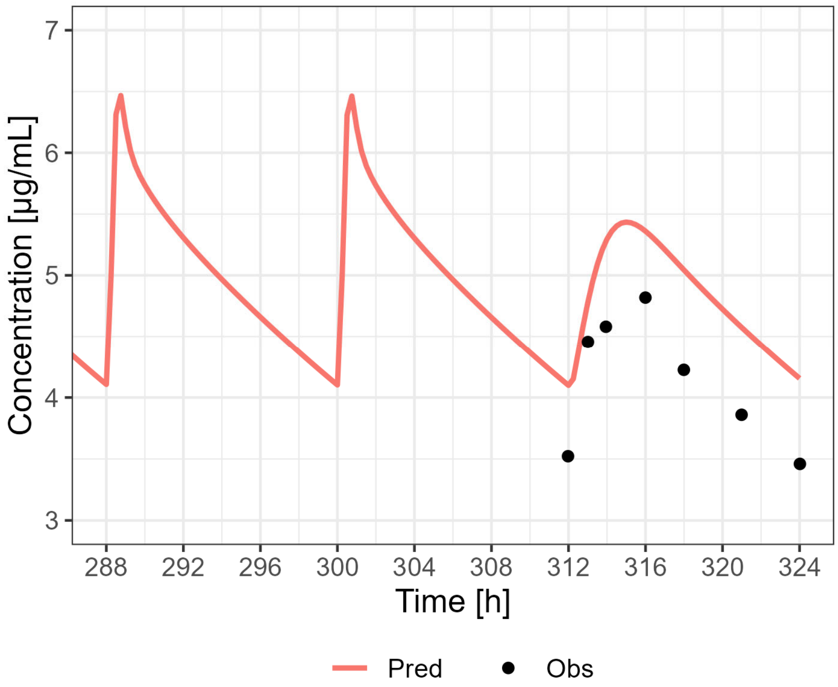

Figure S16 Predicted (Pred) versus observed (Obs) concentration-time profile after administration of 200 mg bid male MD [14]

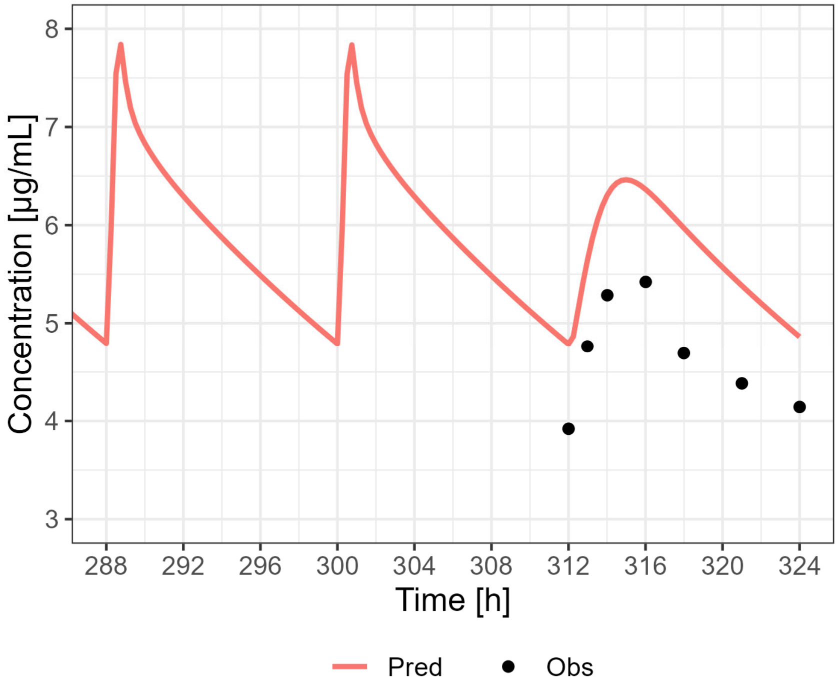

Figure S17 Predicted (Pred) versus observed (Obs) concentration-time profile after administration of 200 mg bid female MD [14]

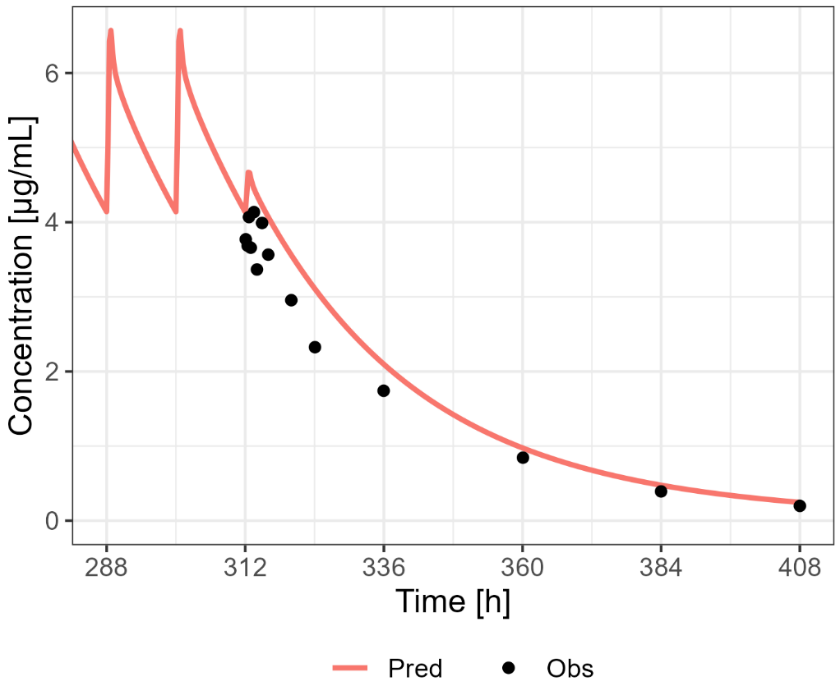

Figure S18 Predicted (Pred) versus observed (Obs) concentration-time profile after administration of 50 mg at steady-state PO [13]

4.3.3 Lactation PBPK model

A sample size of 1000 individuals, three months postpartum, was used in each simulation of the virtual lactation population.

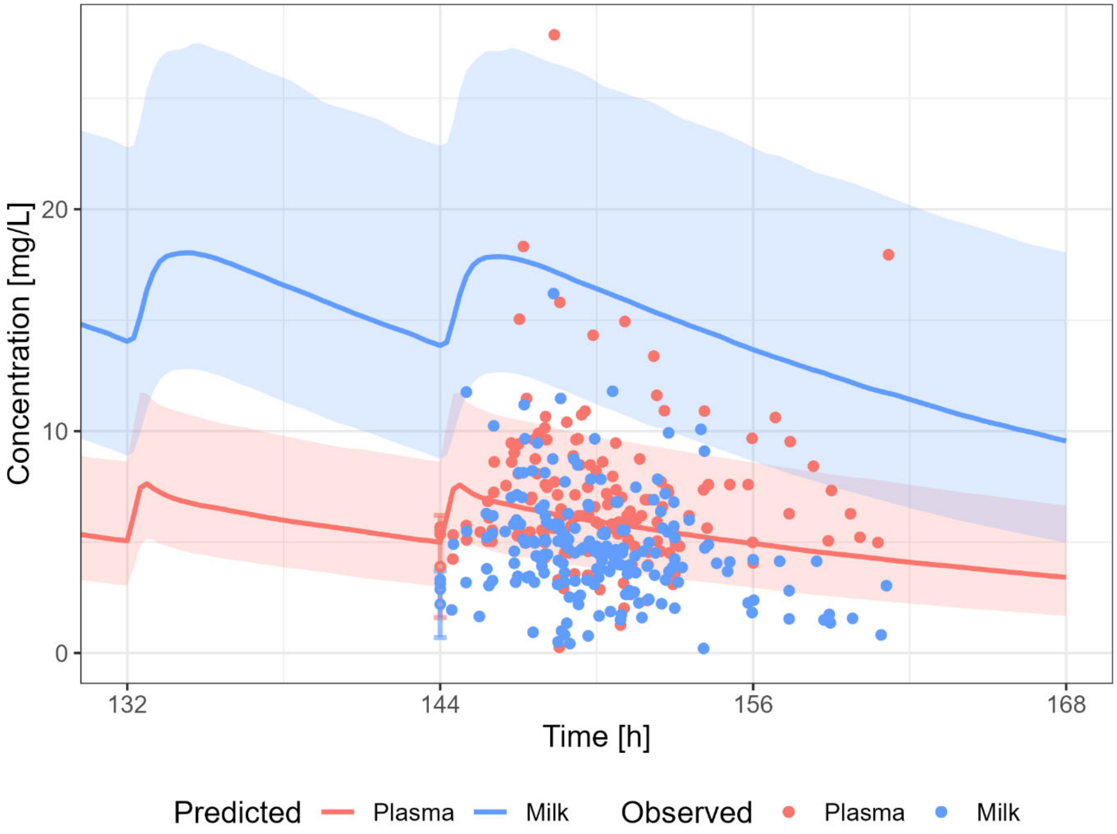

Figure S19 Predicted (Pred) versus observed (Obs) concentration-time profile after administration of 200 mg bid PO MD [15–22]

A dosing regimen of PO 200 mg bidaily was assumed to calculate the milk transfer of nevirapine.

Dosing interval: 12 h	Plasma	Milk
C_{max} (mg/L)	7.79	18.53
AUC (mg*h/L)	72.53	194.17
Cave (mg/L)	6.04	16.18

M/P ratio = 2.68

The PBPK model results in an overprediction of the human milk concentrations.

4.4 Estimated Pediatric exposure

A maternal dosing regimen of 200 mg bidaily was assumed to calculate the infant dosage. The daily infant dosage and relative infant dose (RID) for 3 months old infants were calculated using a milk intake of 150 mL/kg/day. The daily infant dosage was 2.43 mg/kg/day (RID: 37 %) or 2.78 mg/kg/day (RID: 42 %) based on the average steady-state concentration and maximum concentration in human milk, respectively.

5. Discussion

First, the reference PBPK model was developed and evaluated. Evaluation of the predictive performance showed that the reference PBPK model for nevirapine was able to capture the pharmacokinetic behavior of the medicines in healthy volunteers and/or patients.

Next, the PBPK model was extended to a lactation PBPK model. The PBPK model results in an overprediction of the human milk concentrations.

The predicted M/P ratio is 2- to 13-fold higher than the observed range of M/P ratios (0.12 – 5.2).

The calculated infant dosage should be interpreted with caution, since the predicted human milk concentration was too high compared to the observed data.

6. Conclusions

The herein presented PBPK model adequately describes the PK of nevirapine in adults, including breastfeeding women. In particular, it applies quantitative metabolism by cytochrome P450 (CYP3A4, CYP2B6, CYP2D6 and CYP2C9) and renal clearance. The PBPK model for lactation results in an overprediction of the milk concentration (M/P ratio: 2.68). The daily infant dosage was 2.43 mg/kg/day (RID: 37 %) or 2.78 mg/kg/day (RID: 42 %) based on the average steady-state concentration and maximum concentration in human milk, respectively.

7. List of Appendix and Supplementary Materials

Supplementary material 1 – ObsDataPK_OSP_reference_Nevirapine
Supplementary material 2 – ObsDataPK_OSP_lactation_Nevirapine
Supplementary material 3 – Nevirapine.pksim5

8. References

1. Nevirapine: Uses, Interactions, Mechanism of Action | DrugBank Online Available online: https://go.drugbank.com/drugs/DB00238 (accessed on 19 July 2022).
2. De Sousa Mendes, M.; Lui, G.; Zheng, Y.; Pressiat, C.; Hirt, D.; Valade, E.; Bouazza, N.; Foissac, F.; Blanche, S.; Treluyer, J.-M.; et al. A Physiologically-Based Pharmacokinetic Model to Predict Human Fetal Exposure for a Drug Metabolized by Several CYP450 Pathways. *Clin. Pharmacokinet.* **2017**, *56*, 537–550, doi:10.1007/s40262-016-0457-5.
3. Dallmann, A.; Solodenko, J.; Ince, I.; Eissing, T. Applied Concepts in PBPK Modeling: How to Extend an Open Systems Pharmacology Model to the Special Population of Pregnant Women. *CPT pharmacometrics Syst. Pharmacol.* **2018**, *7*, 419–431, doi:10.1002/PSP4.12300.
4. Dallmann, A.; Himstedt, A.; Solodenko, J.; Ince, I.; Hempel, G.; Eissing, T. Integration of Physiological Changes during the Postpartum Period into a PBPK Framework and Prediction of Amoxicillin Disposition before and Shortly after Delivery. *J. Pharmacokinet. Pharmacodyn.* **2020**, *47*, 341–359, doi:10.1007/s10928-020-09706-z.
5. Job, K.M.; Dallmann, A.; Parry, S.; Saade, G.; Haas, D.M.; Hughes, B.; Berens, P.; Chen, J.; Fu, C.; Humphrey, K.; et al. Development of a Generic Physiologically-Based Pharmacokinetic Model for Lactation and Prediction of Maternal and Infant Exposure to Ondansetron via Breast Milk. *Clin. Pharmacol. Ther.* **2022**, *111*, 1111–1120, doi:10.1002/cpt.2530.
6. Koshimichi, H.; Ito, K.; Hisaka, A.; Honma, M.; Suzuki, H. Analysis and Prediction of Drug Transfer into Human Milk Taking into Consideration Secretion and Reuptake Clearances across the Mammary Epithelia. *Drug Metab. Dispos.* **2011**, *39*, 2370–2380, doi:10.1124/dmd.111.040972.
7. Atkinson, U.C.; Begg, E.J.E.J.; Atkinson, H.C.; Begg, E.J.E.J. Prediction of Drug Distribution into Human Milk from Physicochemical Characteristics. *Clin. Pharmacokinet.* **1990**, *18*, 151–167, doi:10.2165/00003088-199018020-00005.
8. Lamson, M.J.; Sabo, J.P.; MacGregor, T.R.; Pav, J.W.; Rowland, L.; Hawi, A.; Cappola, M.; Robinson, P. Single Dose Pharmacokinetics and Bioavailability of Nevirapine in Healthy Volunteers. *Biopharm. Drug Dispos.* **1999**, *20*, 285–291, doi:10.1002/(sici)1099-081x(199909)20:6<285::aid-bdd187>3.0.co;2-v.
9. Kappelhoff, B.S.; Huitema, A.D.R.; van Leth, F.; Robinson, P.A.; MacGregor, T.R.; Lange, J.M.A.; Beijnen, J.H.; 2NN Study Group Pharmacokinetics of Nevirapine: Once-Daily versus Twice-Daily Dosing in the 2NN Study. *HIV Clin. Trials 6*, 254–261, doi:10.1310/B5VU-FU5F-QNWC-UDCK.
10. Fan-Havard, P.; Liu, Z.; Chou, M.; Ling, Y.; Barrail-Tran, A.; Haas, D.W.; Taburet, A.-M.M. Pharmacokinetics of Phase I Nevirapine Metabolites Following a Single Dose and at Steady State. *Antimicrob. Agents Chemother.* **2013**, *57*, 2154–2160, doi:10.1128/AAC.02294-12.
11. Marier, J.F.; Dimarco, M.; Guilbaud, R.; Dodard, C.; Morelli, G.; Tippabhotla, S.K.;

Singla, A.K.; Thudi, N.R.; Monif, T. Pharmacokinetics of Lamivudine, Zidovudine, and Nevirapine Administered as a Fixed-Dose Combination Formulation versus Coadministration of the Individual Products. *J. Clin. Pharmacol.* **2007**, *47*, 1381–1389, doi:10.1177/0091270007307572.

12. Ribera, E.; Pou, L.; Lopez, R.M.; Crespo, M.; Falco, V.; Ocaña, I.; Ruiz, I.; Pahissa, A. Pharmacokinetic Interaction between Nevirapine and Rifampicin in HIV-Infected Patients with Tuberculosis. *J. Acquir. Immune Defic. Syndr.* **2001**, *28*, 450–453, doi:10.1097/00042560-200112150-00007.
13. Riska, P.; Lamson, M.; MacGregor, T.; Sabo, J.; Hattox, S.; Pav, J.; Keirns, J. Disposition and Biotransformation of the Antiretroviral Drug Nevirapine in Humans. *Drug Metab. Dispos.* **1999**, *27*, 895–901.
14. von Hentig, N.; Carlebach, A.; Gute, P.; Knecht, G.; Klauke, S.; Rohrbacher, M.; Stocker, H.; Kurowski, M.; Harder, S.; Staszewski, S.; et al. A Comparison of the Steady-State Pharmacokinetics of Nevirapine in Men, Nonpregnant Women and Women in Late Pregnancy. *Br. J. Clin. Pharmacol.* **2006**, *62*, 552–559, doi:10.1111/j.1365-2125.2006.02664.x.
15. Bennetto-Hood, C.; Aldrovandi, G.M.; King, J.R.; Woodman, K.; Ashouri, N.; Acosta, E.P. Persistence of Nevirapine in Breast Milk after Discontinuation of Treatment. *Clin. Infect. Dis. an Off. Publ. Infect. Dis. Soc. Am.* **2007**, *45*, 391–394, doi:10.1086/519427.
16. Giuliano, M.; Guidotti, G.; Andreotti, M.; Pirillo, M.F.; Villani, P.; Liotta, G.; Marazzi, M.C.; Mancini, M.G.; Cusato, M.; Germano, P.; et al. Triple Antiretroviral Prophylaxis Administered during Pregnancy and after Delivery Significantly Reduces Breast Milk Viral Load: A Study within the Drug Resource Enhancement against AIDS and Malnutrition Program. *J. Acquir. Immune Defic. Syndr.* **2007**, *44*, 286–291, doi:10.1097/QAI.0b013e31802c5441.
17. Mirochnick, M.; Thomas, T.; Capparelli, E.; Zeh, C.; Holland, D.; Masaba, R.; Odhiambo, P.; Fowler, M.G.; Weidle, P.J.; Thigpen, M.C. Antiretroviral Concentrations in Breast-Feeding Infants of Mothers Receiving Highly Active Antiretroviral Therapy. *Antimicrob. Agents Chemother.* **2009**, *53*, 1170–1176, doi:10.1128/AAC.01117-08.
18. Olagunju, A.; Amara, A.; Waitt, C.; Else, L.; Penchala, S.D.; Bolaji, O.; Soyinka, J.; Siccardi, M.; Back, D.; Owen, A.; et al. Validation and Clinical Application of a Method to Quantify Nevirapine in Dried Blood Spots and Dried Breast-Milk Spots. *J. Antimicrob. Chemother.* **2015**, *70*, 2816–2822, doi:10.1093/jac/dkv174.
19. Olagunju, A.; Khoo, S.; Owen, A. Pharmacogenetics of Nevirapine Excretion into Breast Milk and Infants' Exposure through Breast Milk versus Postexposure Prophylaxis. *Pharmacogenomics* **2016**, *17*, 891–906, doi:10.2217/pgs-2015-0016.
20. Palombi, L.; Pirillo, M.F.; Andreotti, M.; Liotta, G.; Erba, F.; Sagno, J.B.; Maulidi, M.; Ceffa, S.; Jere, H.; Marchei, E.; et al. Antiretroviral Prophylaxis for Breastfeeding Transmission in Malawi: Drug Concentrations, Virological Efficacy and Safety. *Antivir. Ther.* **2012**, *17*, 1511–1519, doi:10.3851/IMP2315.
21. Shapiro, R.L.; Holland, D.T.; Capparelli, E.; Lockman, S.; Thior, I.; Wester, C.; Stevens, L.; Peter, T.; Essex, M.; Connor, J.D.; et al. Antiretroviral Concentrations in Breast-Feeding Infants of Women in Botswana Receiving Antiretroviral Treatment. *J. Infect. Dis.* **2005**, *192*, 720–727, doi:10.1086/432483.
22. Shapiro, R.L.; Rossi, S.; Ogwu, A.; Moss, M.; Leidner, J.; Moffat, C.; Lockman, S.; Moyo, S.; Makhema, J.; Essex, M.; et al. Therapeutic Levels of Lopinavir in Late Pregnancy and Abacavir Passage into Breast Milk in the Mma Bana Study, Botswana. *Antivir. Ther.* **2013**, *18*, 1–6, doi:10.3851/IMP2474.

23. Fan-Havard, P.; Liu, Z.; Chou, M.; Ling, Y.; Barrail-Tran, A.; Haas, D.W.; Taburet, A.-M.A.-M. Pharmacokinetics of Phase i Nevirapine Metabolites Following a Single Dose and at Steady State. *Antimicrob. Agents Chemother.* **2013**, *57*, 2154–2160, doi:10.1128/AAC.02294-12 LK - http://limo.libis.be/resolver?&sid=EMBASE&issn=00664804&id=doi:10.1128%2FAAC.02294-12&atitle=Pharmacokinetics+of+phase+i+nevirapine+metabolites+following+a+single+dose+and+at+steady+state&stitle=Antimicrob.+Agents+Chemother.&title=Antimicrobial+Agents+and+Chemotherapy&volume=57&issue=5&spage=2154&epage=2160&aulast=Fan-Havard&aufirst=Patty&auinit=P.&aufull=Fan-Havard+P.&coden=AMACC&isbn=&pages=2154-2160&date=2013&auinit1=P&auinitm=.
