# Supplementary material for: Generic Workflow to Predict Medicine Concentrations in Human Milk Using Physiologically-Based Pharmacokinetic (PBPK) Modelling—A Contribution from the ConcePTION Project
Source: Pharmaceutics. 2023 May 11;15(5):1469. doi: 10.3390/pharmaceutics15051469 (PMC10223637; doi:10.3390/pharmaceutics15051469)
Supplement: Supplementary file 1 [file pharmaceutics-15-01469-s001.zip › File S7. Evaluation_report_sertraline.pdf]

Building and evaluation of a Physiologically-Based PharmacoKinetic (PBPK) model for **sertraline** in adults and lactating women

Disclaimer: The research project leading to these results was conducted as part of the ConcePTION consortium. This report only reflects the personal views of the stated authors. The results of this report are only intended for research purpose, and are not intended to be used in clinical practice.

Glossary

AUC	Area Under the Curve
B/P ratio	Blood-to-Plasma ratio
C_{ave}	Average concentration
CL_{re}	Reuptake clearance (i.e. from milk to blood)
CL_{sec}	Secretion clearance (i.e. from blood to milk)
C_{max}	Maximum (~peak) concentration
DID	Daily Infant Dosage (expressed for instance in mg/kg/day)
DMS	N-desmethyl sertraline
f_u	Fraction unbound in plasma
GFR	Glomerular Filtration Rate
HBD	Hydrogen Bond Donors
IV	Intravenous (administration)
$LogD_{7.2}$	Logarithm of the partition coefficient between an octanol phase and an aqueous (buffer) phase at pH 7.2
$LogD_{7.4}$	Logarithm of the partition coefficient between an octanol phase and an aqueous (buffer) phase at pH 7.4
LogP	Logarithm of the partition coefficient between an octanol phase and (unbuffered) water as aqueous phase. This is the default parameter to express lipophilicity of a substance.
MD	Multiple dose
M/P ratio	Milk-to-Plasma ratio
MW	Molecular Weight (Da)
PBPK	Physiologically-Based PharmacoKinetic [*modeling*]
pKa	Logarithm of the acid dissociation constant
PO	Oral administration
PSA	Polar Surface Area
RID	Relative Infant Dose (%)
RT-PCR	Reverse transcription polymerase chain reaction
SD	Single dose

1. Table of Contents

1. Table of Contents 3

2. Introduction 4

3. Methods 5

3.1 Modelling strategy 5

3.1.1. Reference PBPK models 6

3.1.2. Lactation model 6

3.2 Data 6

3.2.1 *In vitro* / physicochemical data 6

3.2.2 Clinical data 10

3.3 Model Parameters and assumptions 12

3.3.1 Absorption 12

3.3.2 Distribution 12

3.3.3 Metabolism and excretion 13

3.3.4 Secretion to milk 13

3.3.5 Automated parameter optimization 14

3.4. Infant dosage calculation 14

4. Results 14

4.1 Final input parameters 15

4.2 Diagnostic plots 17

4.3 Concentration-time profiles 19

4.3.1 Model building 20

4.3.2 Model verification 22

4.3.3 Lactation PBPK model 33

4.4 Estimated Infant dosage 51

5. Discussion 52

6. Conclusion 52

7. List of Appendix and Supplementary Materials 53

8. References 53

2. Introduction

Sertraline (figure S1) is a selective serotonin reuptake inhibitor (SSRI) and is used to treat major depressive disorder, panic disorder, obsessive-compulsive disorder and post-traumatic stress disorder [1]. Recommended doses range from 25 mg up to 200 mg a day (Drug label Zoloft). Sertraline is slowly absorbed from the gastrointestinal tract following oral administration reaching maximum plasma concentrations at 4-8 hours after oral administration; bioavailability of sertraline is estimated to be 44 %. The volume of distribution of sertraline is 20 L/kg [2]. It is reported that sertraline is metabolized to N-desmethyl sertraline (DMS) by CYP2D6, CYP2C9, CYP2B6, CYP2C19, CYP3A4 and CYP2E1 [3]. DMS appears to have 5-10 % of the potency of sertraline on serotonin uptake inhibition [4]. Sertraline is metabolized to sertraline carbamic acid, N-hydroxy-sertraline and the deamidated ketone of sertraline. These metabolites are conjugated with glucuronic acid and undergo biliary and urinary excretion. Sertraline undergoes extensive first-pass oxidation to form DMS. The elimination half-life ranges from 22 to 36 hours [1].

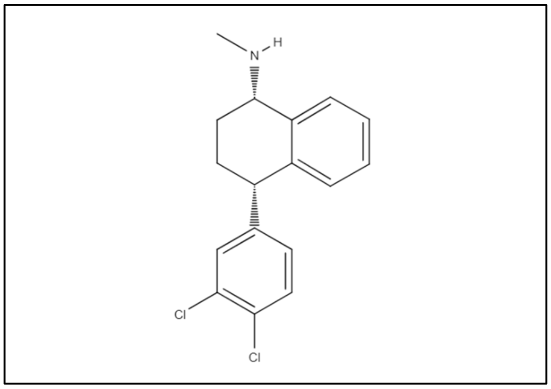

Figure S1 Chemical Structure of sertraline

The scope of this report is to:

(a) specify the details and underlying assumptions associated with the building of physiologically-based pharmacokinetic (PBPK) models for sertraline in adult healthy volunteers or patients, and in postpartum women during lactation.

(b) evaluate the predictive performance of these PBPK models. This is achieved by comparing model-predicted plasma or milk concentrations with corresponding clinical observations.

3. Methods

The software used for the development of PBPK models presented in this report is tabulated below:

Software	Version
PK-Sim®	v9.1
MoBi®	v9.1

3.1 Modelling strategy

In the present report, a reference PBPK model was first established for adults (patients as well as healthy volunteers), and subsequently verified against clinical pharmacokinetic data reported for sertraline in the scientific literature.

Relevant information on the anthropometry (height, weight) was gathered from the respective clinical studies, if reported. Information on physiological parameters (e.g. blood flows, organ volumes, hematocrit) in adults is available in the PK-Sim® database.

In a second step, a lactation PBPK model was developed, based on the general workflow described by Dallmann *et al. 2018* [5–7].

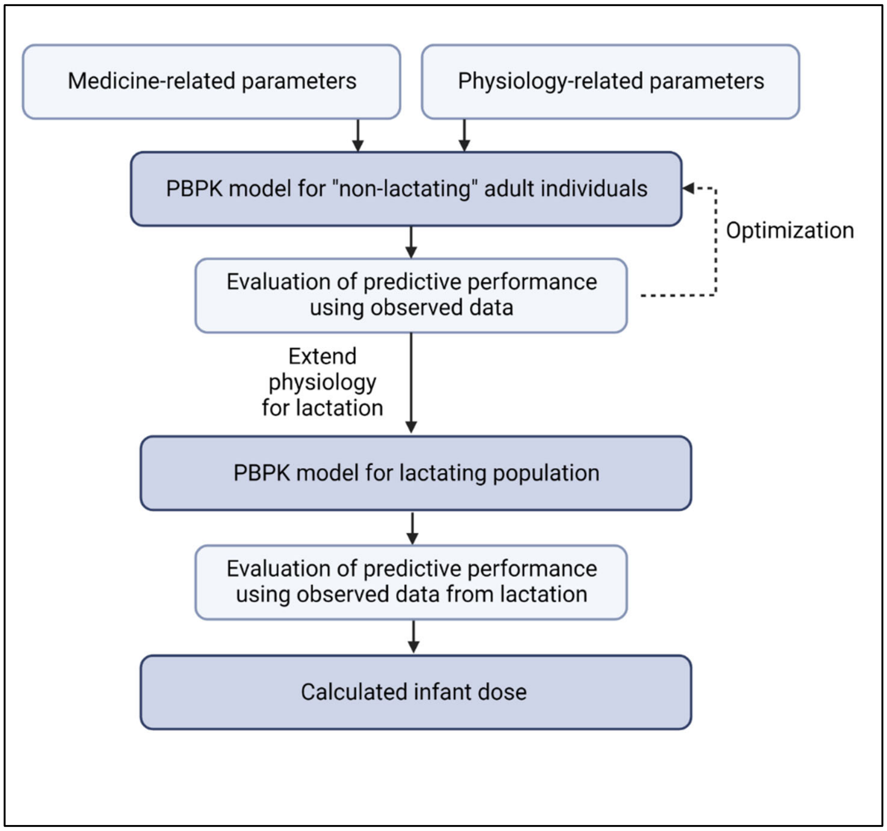

Figure S2 General workflow that was used in the present project to develop and evaluate the lactation PBPK model

Details about input data (physicochemical, *in vitro* and clinical data) can be found in section 3.2. Details about the structural models and their parameters can be found in section 3.3.

3.1.1. Reference PBPK models

The reference PBPK model was built based on studies with adult volunteers and/or adult patients, using the reported mean values for age, weight, height, and genetic background as described in each study protocol. When no information on these parameters could be found, a healthy male European individual, 30 years of age, with a body weight of 73 kg and a height of 176 cm was used.

The abundance (including population variability) of plasma proteins and enzymes/transporters that are integrated into PK-Sim are described in the publicly available 'PK-Sim Ontogeny Database Version 7.3' (PK-Sim Ontogeny Database Version 7.3).

To model the specific metabolic clearance of sertraline; CYP2D6, CYP2C9, CYP2B6, CYP2C19, CYP3A4 and CYP2E1 were implemented in accordance with literature, using the PK-Sim expression database RT-PCR profiles to define their relative expression in the different organs of the body. Those enzymes were implemented using *in vitro* metabolic rate in the presence of liver microsomes. Glomerular filtration was enabled as it is involved in the excretion.

Structural model selection was mainly guided by biological plausibility and by visual inspection of the predicted concentration time profiles in comparison with observed data. The generally applied acceptance criterium was less than 2-fold misprediction. Uninformed parameter values (see below) were estimated using the parameter identification module of PK-Sim®.

The predictive performance of the models was evaluated by simulating:
- Single intravenous dose studies
- Single and multiple oral dose studies

For some parameters, parameter optimization was performed as described below to obtain improved concordance between predicted profiles and observed data.

3.1.2. Lactation model

After development of the reference model, the model was exported to MoBi® and a lactation PBPK model was constructed. To model the passage of sertraline into human milk, i.e. across the blood/milk biological barrier, both the secretion (CL_{sec}) and reuptake clearance (CL_{re}) values were obtained using the empirical model developed by Koshimichi *et al. 2011* [8].

3.2 Data

3.2.1 *In vitro* / physicochemical data

A literature search was performed to collect available information on physicochemical properties of sertraline. The obtained information from literature is summarized in Table S1 and Table S2 show the parameters that were additionally used for the lactation PBPK models.

Sertraline

Table S1 Physicochemical parameters used as input for the sertraline PBPK models

Parameter	Value	Unit	Description	Source
MW<br>Has halogens	306.00<br>Yes, 2 Cl-	g/mol	Molecular weight (two halogen atoms)	[9]
pK_a	9.43 (base)	-	Logarithm of the acid dissociation constant	[9]
Solubility (pH 5.3)	3.80	mg/mL	Solubility	Drugbank
LogP	5.5	-	Log_{10} of the partition coefficient between octanol and water (~lipophilicity)	[9]
f_u	0.023	-	Fraction unbound in human plasma	[9]
CYP2B6 - In vitro CL for liver microsomes	2.7	µL/min/mg mic. Protein	Metabolic enzyme activity	[3]
CYP2B6 - Content of CYP proteins in liver microsomes	41	Pmol/mg mic. protein		
CYP2C19 - In vitro CL for liver microsomes	2.4	µL/min/mg mic. Protein		
CYP2C19 - Content of CYP proteins in liver microsomes	19	Pmol/mg mic. protein		
CYP2C9 - In vitro CL for liver microsomes	5.5	µL/min/mg mic. Protein		
CYP2C9 - Content of CYP proteins in liver microsomes	96	Pmol/mg mic. protein		
CYP2D6 - In vitro CL for liver microsomes	6.8	µL/min/mg mic. Protein		
CYP2D6 - Content of CYP proteins in liver microsomes	22	Pmol/mg mic. protein		

CYP3A4 - In vitro CL for liver microsomes	1.8	µL/min/mg mic. Protein		
CYP3A4 - Content of CYP proteins in liver microsomes	142	Pmol/mg mic. protein		
CYP2E1 - In vitro CL for liver microsomes	9.5	µL/min/mg mic. Protein		
CYP2E1 - Content of CYP proteins in liver microsomes	49	Pmol/mg mic. protein		
B/P concentration ratio	2.925	-	-	[10]
GFR fraction	1.00	-	Fraction of the glomerular filtration rate used for passive renal elimination	
Intestinal permeability	5.70E-6	Cm/s	Intestinal permeability	[11]

Table S2 Physicochemical parameters used as input for the lactation PBPK model of sertraline

**Parameter**	**Value**	**Unit**	**Description**	**Source**
Milk logP[a]	5.50	-	Log_{10} of the partition coefficient between octanol and water	[9]
	5.00	-		MarvinSketch
HBD	1.00	-	Hydrogen bond donors	Pubchem
PSA	12.03	Å^2	Polar surface area	Pubchem

[a] Milk logP is Log_{10} of the partition coefficient between octanol and water and is used as input for the calculations in the postpartum model (see equations below). In theory, this value is identical to the logP specified in Table S1. However, in some PBPK models, logP (Table S1) might be optimized using parameter identication. Therefore, it was chosen to use a separate parameter (i.e. Milk logP) to represent the logP used as input for the equations in the postpartum model.

The default equations for free fraction in human milk and logD that were implemented in the spatial structure building block that was developed for the postpartum women are described below. Alternatively, these values can be overwritten by values calculated elsewhere (e.g. MarvinSketch) or determined *in vitro.*

The free fraction in human milk was calculated with the equations proposed by Atkinson and Begg [12], as follows:

$$f_{u\_skimmed\ milk} = \frac{f_u \times 0.448}{(0.000694^{0.448} + f_u^{0.448})}$$

$$P_{milk} = 10^{(-0.88+1.29\times \text{logD7.2})}$$

$$\text{Total free fraction in milk} = \frac{1}{(\frac{0.955}{f_{u\_skimmed\ milk}} + 0.045 \times P_{milk})}$$

Where: *f_u skimmed milk:* binding to proteins in milk; P_{milk}: partitioning between aqueous and lipid phase of milk; Total free fraction in milk: 'total' free fraction, i.e. accounting for both protein and lipid binding processes.

LogD values taking into account up to three pka values (as provided in the compound building block), were calculated as follows:

$$LogD = \text{Log}\,P + Log_{10}(logD_{factor})$$

With Milk logP (Table S2) as input for logP

$$LogD_{factor} = K_1 + (K_2 + K_3 + K_4) \times \text{base}^1 + K_5 \times \text{base}^{\max(CT_0+CT_1;-CT_0-CT_1)} + K_6 \times \text{base}^{\max(CT_0+CT_2;-CT_0-CT_2)} + K_7 \times \text{base}^{\max(CT_2+CT_1;-CT_2-CT_1)} + K_8 \times \text{base}^{\max(CT_0+CT_1+CT_2;-CT_0-CT_1-CT_2)}$$

$K_1 = F_1 \times F_2 \times F_3$
$K_2 = (1 - F_1) \times F_2 \times F_3$
$K_3 = F_1 \times (1 - F_2) \times F_3$
$K_4 = F_1 \times F_2 \times (1 - F_3)$
$K_5 = (1 - F_1) \times (1 - F_2) \times F_3$
$K_6 = (1 - F_1) \times F_2 \times (1 - F_3)$
$K_7 = (1 - F_1) \times F_2 \times (1 - F_3)$
$K_8 = (1 - F_1) \times (1 - F_2) \times (1 - F_3)$

F1 = CT0 != CT_NEUTRAL ? 1/(1+10^(CT0*(pKa_0- pH))) : 1
F2 = CT1 != CT_NEUTRAL ? 1/(1+10^(CT1*(pKa_1- pH))) : 1
F3 = CT2 != CT_NEUTRAL ? 1/(1+10^(CT2*(pKa_2- pH))) : 1

With CT = compound type (-1: acid; +1: base; 0: neutral), and pH = 7.2 or 7.4 respectively for $logD_{7.2}$ and $logD_{7.4}$

The transports that were added in the passive transport building block for 'transfer to milk' and 'transfer from milk' are based on secretion and reuptake and clearance values, Cl_{sec} and Cl_{re}, which were calculated according to the empirical equations proposed by Koshimich et al. 2011 [8], as follows:

$$\text{Log}\,CL_{re} = 2.793 + 0.179 \times LogP - 0.132 \times HBD$$

$$\text{Log}\,CL_{sec} = 3.367 \times Log10(MW) - 0.164 \times (LogP - LogD) - 0.015 \times PSA - 3.912$$

3.2.2 Clinical data

Literature searches were performed to collect available data on sertraline in adults and postpartum women. The sertraline reference PBPK model was developed using 4 different clinical studies with pharmacokinetic (PK) blood sampling. In the first iteration, the clinical study was used where 12 subjects received 100 mg sertraline IV [13]. Subsequently, three clinical trials with oral administration in a range of 100 up to 400 mg were taken into account [14–16]. Next, 9 clinical trials with oral administrations were used for evaluation of the prediction performance [14,17–24].

The evaluation of the predictive performance of the sertraline lactation PBPK model was performed using 11 different studies were sertraline was administered with doses ranging from 25-200 mg/day to lactating women [25–35]. The women were between 1 day and 14.2 months postpartum. The samples were assumed to be trough samples when the exact timing was not reported in the articles.

Detailed information and data from the studies used for model building, verification, and lactation model can be found in Supplementary material 1 and 2.

3.2.2.1 Model building

The studies that were used for model building are shown in Table S3 (**training data**).

Table S3 Summary of studies used for PBPK model building of sertraline in reference populations

**Study ID**	**Reference**	**Arm/treatment/information used for model building**
Sutton 2009	[13]	12 subjects received IV 100 mg
Saletu 1986	[16]	10 subjects received PO 100 mg
Saletu 1986	[16]	10 subjects received PO 200 mg
Saletu 1986	[16]	10 subjects received PO 400 mg
Warrington 1991	[14]	24 subjects received PO 50 mg
Warrington 1991	[14]	24 subjects received PO 100 mg
Warrington 1991	[14]	24 subjects received PO 200 mg
Yue 2016	[15]	20 subjects received PO 100 mg

Table S4 Demographic information

**Study ID**	**Reference**	**Number of subjects (female ratio)**	**Age (year)**	**Weight (kg)**
Sutton 2009	[13]	12 (-)	-	-
Saletu 1986	[16]	10 (0.50)	26 (19-31)	62 (50-80)
Warrington 1991	[14]	24 (0)	- (18-45)	-
Yue 2016	[15]	20 (0)	-	-

3.2.2.2 Model verification

The studies that were used to evaluate the predictive performance of the PBPK model are shown in Table S5 (**verification data**).

Table S5 Summary of studies used for model verification of sertraline PBPK model in reference population

Study ID	Reference	Arm/treatment/information used for model verification
Allard 2013	[17]	27 females received PO 50 mg/day
Kang 2011	[18]	24 subjects received PO 50 mg (reference)
Kang 2011	[18]	24 subjects received PO 50 mg (test)
Koytchev 2004	[19]	24 subjects received PO 50 mg (reference)
Koytchev 2004	[19]	24 subjects received PO 50 mg (test)
Nagy 2020	[20]	19 subjects received PO 50 mg
Nagy 2020	[20]	19 subjects received PO 100 mg/day
Ronfled 1997b	[21]	14 females received PO 200 mg/day
Ronfled 1997b	[21]	12 males received PO 200 mg/day
Ronfeld 1997a	[22]	31 subjects received PO 100 mg (morning)
Ronfeld 1997a	[22]	31 subjects received PO 100 mg (evening)
Ronfeld 1997a	[22]	32 subjects received PO 100 mg (fasted)
Ronfeld 1997a	[22]	32 subjects received PO 100 mg (fed)
Wang 2001	[23]	6 PM and 6 EM received PO 100 mg
Warrington 1991	[14]	Females received 200 mg/day
Warrington 1991	[14]	Males received 200 mg/day
Zhang 2011	[24]	18 subjects received PO 50 mg (reference)
Zhang 2011	[24]	18 subjects received PO 50 mg (test)

Table S6 Demographic information

Study ID	Reference	Number of subjects (female ratio)	Age (year)	Weight (kg)
Allard 2013	[17]	27 (1)	30.1 (20-44)	
Kang 2011	[18]	24 (0)	23.5 ± 1.74	64.04 ± 7.1
Koytchev 2004	[19]	24 (0)	26.9 (18-43)	71.3 (60-93)
Nagy 2020	[20]	19 (0.16)	31.3 (20-45)	76.6 (57-89.9)
Ronfled 1997b	[21]	12 (0)<br>14 (1)	30.6 (21-43)<br>34.4 (20-45)	72.5 (64.6-83.2)<br>62.1 (52.3-79.6)
Ronfeld 1997a	[22]	31 (-)<br>32 (-)	26.9 (18-44)<br>26.6 (18-43)	78.5 (61.4-90.9)<br>75.1 (61.4-89.1)
Wang 2001	[23]	6 (0)	20 (19-22)	65 (54-80)
Warrington 1991	[14]	24 (0)	- (18-45)	-
Zhang 2011	[24]	18 (0)	-	-

3.2.2.3 Lactation PBPK model

Table S7 shows the study that was used for the lactation PBPK model.

Table S7 Summary of study used for PBPK model development of sertraline in lactating women

Study ID	Publication	Arm/treatment/information used for model building and verification
Dodd 2000	[25]	Sertraline was measured in women receiving PO 50-150 mg/day (multiple dose)
Kristensen 1998	[26]	Sertraline and N-desmethylsertraline were measured in women (1.8-14.2 months postpartum) receiving PO 50 mg/day (multiple dose)
Müller 2013	[28]	Sertraline and N-desmethylsertraline were measured in women (< 9 days postpartum) receiving PO 150 mg/day (multiple dose)
Oyesten Berle 2003	[30]	Sertraline was measured in women (5-34 weeks postpartum) receiving PO 64 mg/day (multiple dose)
Oberlander 2005	[29]	Sertraline was measured in women (2 months postpartum) receiving PO 87.5 mg/day (multiple dose)
Pogliani 2019	[31]	Sertraline was measured in women (3-7 days postpartum) receiving PO 25-75 mg/day (multiple dose)
Rodrigues Salazar 2016	[32]	Sertraline was measured in women receiving PO 50 mg/day (multiple dose)
Schoretsanitis 2018	[33]	Sertraline was measured in women (1-14 days postpartum) receiving PO 50 mg/day (multiple dose)
Weisskopf 2017	[34]	Sertraline was measured in women (4 weeks postpartum) receiving PO 50 mg/day (multiple dose)
Weissman 2004	[27]	Sertraline and N-desmethylsertraline were measured in women (4.0-19.6 weeks postpartum) receiving PO 50-100 mg/day (multiple dose)
Wisner 1998	[35]	Sertraline and N-desmethylsertraline were measured in women (<22 weeks postpartum) receiving PO 50-200 mg/day (multiple dose)

3.3 Model Parameters and assumptions

3.3.1 Absorption

The release of sertraline from the tablet was implemented using the observed dissolution profile [18]. The intestinal permeability of sertraline was estimated using parameter optimization.

3.3.2 Distribution

An important parameter influencing the distribution of a compound is lipophilicity. The lipophilicity of sertraline and DMS were taken from literature [9]. The tissue partition coefficients (Kp) calculation was according to 'Rodgers and Rowland' and the cellular permeability calculation was 'PK-Sim Standard' for both sertraline. As indicated by Alhadab and Brundage (2020) [10], the B/P ratio of sertraline has a major influence on the shape of the

concentration-time profile. To this end, their reported B/P ratio (2.925) was put in the simulation parameters.

3.3.3 Metabolism and excretion

The final model applies metabolism by CYP2D6, CYP2C9, CYP2B6, CYP2C19, CYP3A4 , CYP23E1 and glomerular filtration for sertraline. The in vitro clearance values were taken from literature, and were manually optimized as a factor [36]. Renal clearance of sertraline was implemented as glomerular filtration, with the fraction set to its default value of 1.

3.3.4 Secretion to milk

To model the transfer process of sertraline into human milk, both the secretion (CL_{sec}) and reuptake clearance (CL_{re}) were calculated using the empirical equations developed by Koshimichi *et al.* 2011 (see 3.2.1 *In vitro* / physicochemical data) [8].

First, in MoBi®, a spatial structure for the postpartum women was constructed, similar to the workflow from Dallmann *et al.* 2018 [5]. Here, breasts were added as a compartment. In addition, the human milk was connected to the plasma subcompartment of the breasts. The human milk volume was specified as 0.5 L to represent the structure of Koshimichi *et al.* 2011, and a geometric standard deviation of 1.16 was assumed in the population. The free fraction in human milk, and logD values were implemented as the equations described previously. The transfer between plasma and milk was defined as two kinetic processes (transfer to milk and transfer from milk) under passive transports (see below). Next, the simulation was combined with the postpartum population from Job *et al.* 2021 in PK-Sim to account for the postpartum physiology [7].

<u>*Kinetics*</u>

Transfer to milk

$$\frac{dNmilk}{dt} = Cplasma \times f_u \times CL_{sec}$$

where Cplasma is the concentration in plasma (in breast compartment), f_u is the free fraction in plasma and CL_{sec} is the secretion clearance.

Transfer from milk

$$\frac{dNplasma}{dt} = Cmilk \times f_u \times CL_{re}$$

where Cmilk is the concentration in human milk, f_u is the total free fraction in human milk (protein and lipid) and CL_{re} is the reuptake clearance.

The median simulated plasma and human milk concentration-time profiles can be used to calculate the M/P ratio as follows:

$$M/P\ ratio\ = \frac{AUC_{milk}}{AUC_{plasma}}$$

3.3.5 Automated parameter optimization

The following table depicts the results of the final parameter optimization according to the different clinical studies.

a) Sertraline:
   Sutton 2009 IV 100 mg [13]

Model parameter	Optimized value	Unit
In vitro clearance for liver microsomes as factor	16	-

b) Nagy 2020 PO 50 mg; Nagy 2020 PO 100 mg (multiple dose); Yue 2016 PO 100 mg [15,20]:

Model parameter	Optimized value	Unit
Specific intestinal permeability (transcellular)	5.36E-7	cm/s

3.4. Infant dosage calculation

Infant dosage via human milk was then calculated based on the predicted (average and maximal) steady-state sertraline concentration in human milk, as well as the daily milk intake volume. The daily infant dosage was then compared to the maternal dosage, resulting in the relative infant dose (RID).

$$Daily\ infant\ dosage = C_{average} * 150\ \frac{mL}{kg\ .day}$$

$$Daily\ infant\ dosage = C_{max} * 150\ \frac{mL}{kg\ .day}$$

$$Relative\ infant\ dose\ (RID) = \frac{Infant\ dosage}{Maternal\ dosage} * 100\ \%$$

4. Results

Both the reference and postpartum PBPK model of sertraline was developed and verified with clinical PK data.

The models were evaluated covering studies including in particular:

- Intravenous and oral administration
- Single and multiple doses
- A dose range from 50 to 400 mg

The model describes metabolism via CYP2D6, CYP2C9, CYP2B6, CYP2C19, CYP3A4, CYP2E1 and glomerular filtration. Moreover, secretion and reuptake to human milk were described by CL_{sec} and CL_{re}.

The next sections show:

- The final model parameters for the building blocks: section 4.1
- The overall predictive performance: section 4.2
- The simulated versus observed concentration-time profiles for the clinical studies used for model building and for model verification: section 4.3

4.1 Final input parameters

The compound values of the final postpartum PBPK model for sertraline are illustrated below.

Physicochemical parameters

**Parameter**	**Value**	**Unit**	**Source**
MW	306.00<br>Yes, 2 Cl-	g/mol	[9]
pKa	9.43 (base)	-	[9]
Solubility	3.80	mg/mL	Drugbank
Lipophilicity	5.5	-	[9]
f_u	0.023	-	[9]
Small molecule (Y/N)	Yes	-	-
Plasma protein binding partner	Albumin	-	-
B/P concentration ratio	2.925	-	Parameter identification

Calculation methods

**Name**	**Value**
Tissue partition coefficients	Rodgers and Rowland
Cellular permeabilities	PK-Sim Standard

AMDE-related parameters

**Parameter**	**Value**	**Unit**	**Source**
CYP2B6 - In vitro CL for liver microsomes	43.20	µL/min/mg mic. Protein	Manual fit
CYP2B6 - Content of CYP proteins in liver microsomes	41	Pmol/mg mic. protein	
CYP2C19 - In vitro CL for liver microsomes	38.40	µL/min/mg mic. Protein	
CYP2C19 - Content of CYP proteins in liver microsomes	19	Pmol/mg mic. protein	
CYP2C9 - In vitro CL for liver microsomes	88.00	µL/min/mg mic. Protein	
CYP2C9 - Content of CYP proteins in liver microsomes	96	Pmol/mg mic. protein	

CYP2D6 - In vitro CL for liver microsomes	108.80	µL/min/mg mic. Protein	
CYP2D6 - Content of CYP proteins in liver microsomes	22	Pmol/mg mic. protein	
CYP3A4 - In vitro CL for liver microsomes	28.80	µL/min/mg mic. Protein	
CYP3A4 - Content of CYP proteins in liver microsomes	142	Pmol/mg mic. protein	
CYP2E1 - In vitro CL for liver microsomes	152.00	µL/min/mg mic. Protein	
CYP2E1 - Content of CYP proteins in liver microsomes	49	Pmol/mg mic. protein	
Specific intestinal permeability (transcellular)	5.36E-7	cm/s	Parameter identification
GFR	1	-	-

Formulation-related parameters
Type: solution

Type: Tablet formulation – reference (Zoloft)

Parameter	Value	Unit	Source
Use as suspension (Y/N)	Yes	-	

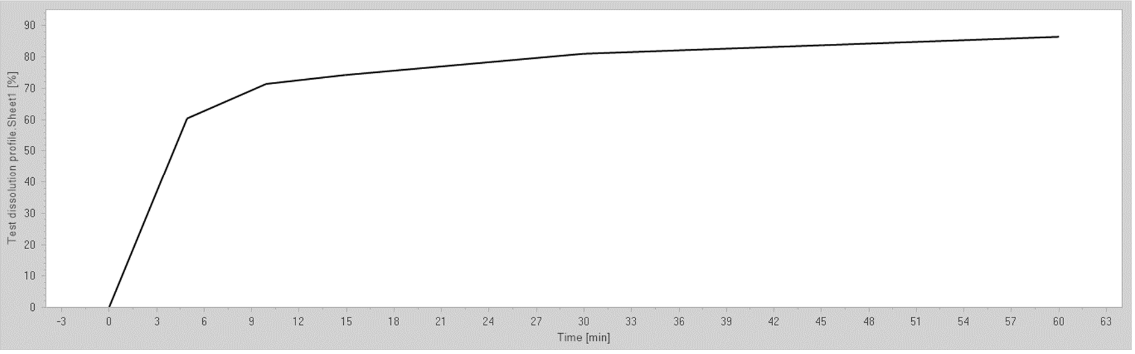

Figure S3 In vitro dissolution profile of sertraline for the tablet formulation (reference) [18]

Physicochemical and physiological parameters relevant to the lactation model

Parameter	Value	Unit	Source
Milk logP	5.00	-	MarvinSketch
HBD	1.00	-	MarvinSketch

PSA	12.03	Å^2	MarvinSketch
CL_{sec}	0.15	L/min	Default
CL_{re}	0.06	L/min	Default
*fu*_skimmed milk[a]	0.83	-	Default
Pmilk[b]	599.63	-	Default
Total free fraction in milk[c]	0.04	-	Default
$logD_{7.2}$	2.84	-	Default
$logD_{7.4}$	3.01	-	Default

[a] binding to proteins in milk; [b] partitioning between aqueous and lipid phase of milk; [c] total free fraction, accounting for both protein and lipid binding

4.2 Diagnostic plots

The geometric mean fold errors (GMFE) on AUC and Cmax were 1.32 and 1.17 for the model building dataset, and 1.51 and 1.33 for the model verification dataset.
The following plot shows the predictive performance graph for Cmax and AUC of sertraline for the PBPK model performance of all data used.
Predicted over observed ratio values of all data listed in section 3.2.2 are presented below.

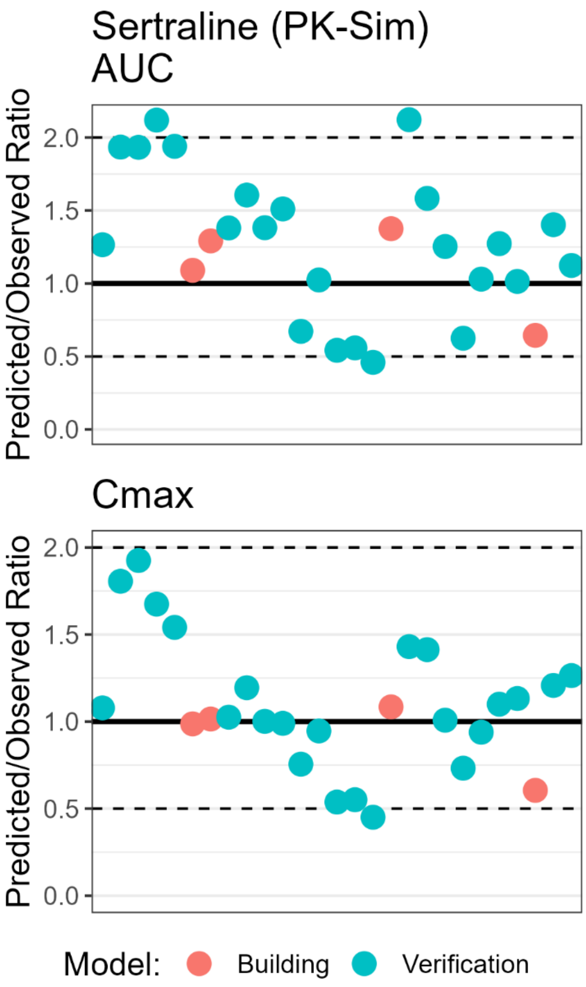

Figure S4. Predicted over observed ratio profile for sertraline

Table S8 Ratio between the predicted and observed pharmacokinetic parameters of sertraline in different dosing regimens for model building

Study ID/ Reference	Dose/ Route	AUC_{obs} (µg*h/L)	AUC_{pred} (µg*h/L)	Fold error	$Cmax_{obs}$ (µg/L)	$Cmax_{pred}$ (µg/L)	Fold error
Nagy 2020 [20]	100 mg PO MD	2062.01	2248.17	1.09	52.59	51.90	0.99
Nagy 2020 [20]	50 mg PO SD	171.92	222.10	1.29	11.58	11.75	1.01
Sutton 2009 [8]	100 mg IV SD	1443.88	1985.83	1.38	52.07	56.46	1.08
Yue 2016 [15]	100 mg PO SD	1538.56	992.14	0.64	40.12	24.27	0.60

Table S9 Ratio between the predicted and observed pharmacokinetic parameters of sertraline in different dosing regimens used for model verification

Study ID/ Reference	Dose/ Route	AUC_{obs} (µg*h/L)	AUC_{pred} (µg*h/L)	Fold error	$Cmax_{obs}$ (µg/L)	$Cmax_{pred}$ (µg/L)	Fold error
Allard 2013 [17]	50 mg PO MD	486.66	615.87	1.27	28.15	30.36	1.08
Kang 2011 [18]	50 mg PO reference	257.53	498.11	1.93	7.42	13.40	1.81
Kang 2011 [18]	50 mg PO test	257.76	498.11	1.93	6.96	13.40	1.93
Koytchev 2004 [19]	50 mg PO reference capsule	218.43	462.87	2.12	7.50	12.56	1.67
Koytchev 2004 [19]	50 mg PO test capsule	259.90	504.12	1.94	8.15	12.56	1.54
Ronfeld 1997b [21]	200 mg MD female	7831.83	5265.78	0.67	159.74	120.65	0.76
Ronfeld 1997b [21]	200 mg MD male	4138.4	4238.15	1.02	111.11	105.15	0.95
Ronfeld 1997a [22]	100 mg PO SD (1)	651.70	899.71	1.38	22.97	23.55	1.03
Ronfeld 1997a [22]	100 mg PO SD (2)	569.18	913.90	1.61	20.3	24.25	1.19
Ronfeld 1997a [22]	100 mg PO SD (3)	661.22	913.90	1.38	24.22	24.25	1.00
Ronfeld 1997a [22]	100 mg PO SD (4)	595.51	899.71	1.51	23.79	23.55	0.99

Saletu 1986 [16]	100 mg PO SD	305.18	165.53	0.54	52.19	28.06	0.54
Saletu 1986 [16]	200 mg PO SD	592.08	330.86	0.56	101.78	56.09	0.55
Saletu 1986 [16]	400 mg PO SD	1439.16	661.97	0.46	249.03	112.22	0.45
Wang 2001 [23]	100 mg PO SD EM	633.55	1344.22	2.12	21.88	31.30	1.43
Wang 2001 [23]	100 mg PO SD PM	920.94	1457.75	1.58	22.15	31.30	1.41
Warrington 1991 [14]	100 mg PO SD	791.61	992.14	1.25	24.08	24.27	1.01
Warrington 1991 [14]	200 mg PO SD	1380.00	1756.94	1.27	44.16	48.57	1.10
Warrington 1991 [14]	50 mg PO SD	391.35	396.94	1.01	10.95	12.40	1.13
Warrington 1991 [14]	200 mg MD female	8389.16	5244.98	0.63	163.73	119.89	0.73
Warrington 1991 [14]	200 mg MD male	4215.62	4341.88	1.03	110.57	103.81	0.94
Zhang 2011 [24]	50 mg PO reference	461.13	518.08	1.12	13.10	16.56	1.26
Zhang 2011 [24]	50 mg PO test	458.23	642.82	1.40	13.34	16.11	1.21

4.3 Concentration-time profiles

Simulated versus observed concentration-time profiles of all data listed in section 3.2.2 are presented below. The original.pksim5 are provided in Supplemented material 3.

4.3.1 Model building

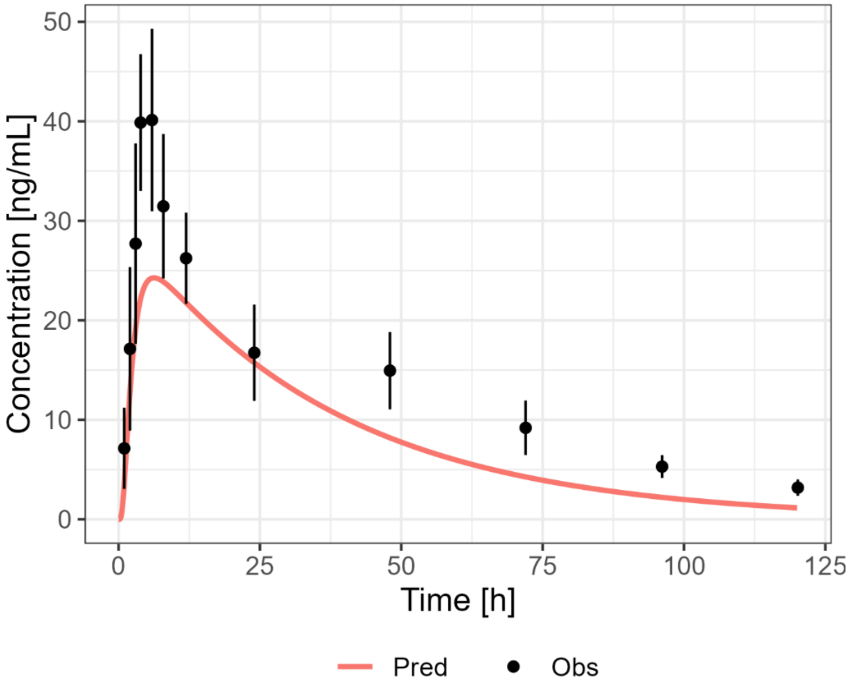

Figure S5 Predicted (Pred) versus observed (Obs) concentration-time profile after administration of 100 mg PO [15]

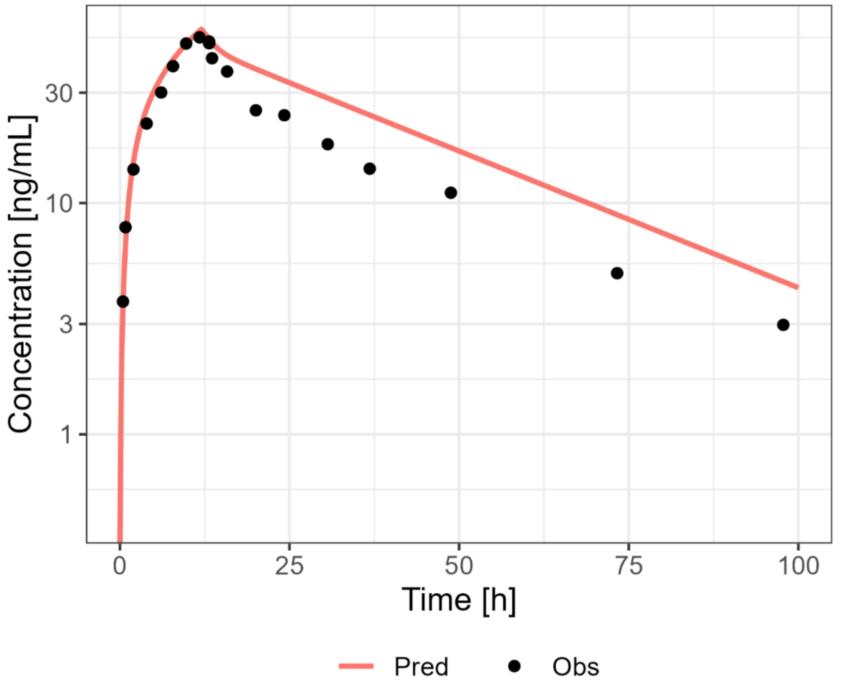

Figure S6 Predicted (Pred) versus observed (Obs) concentration-time profile after administration of 100 mg IV [13]

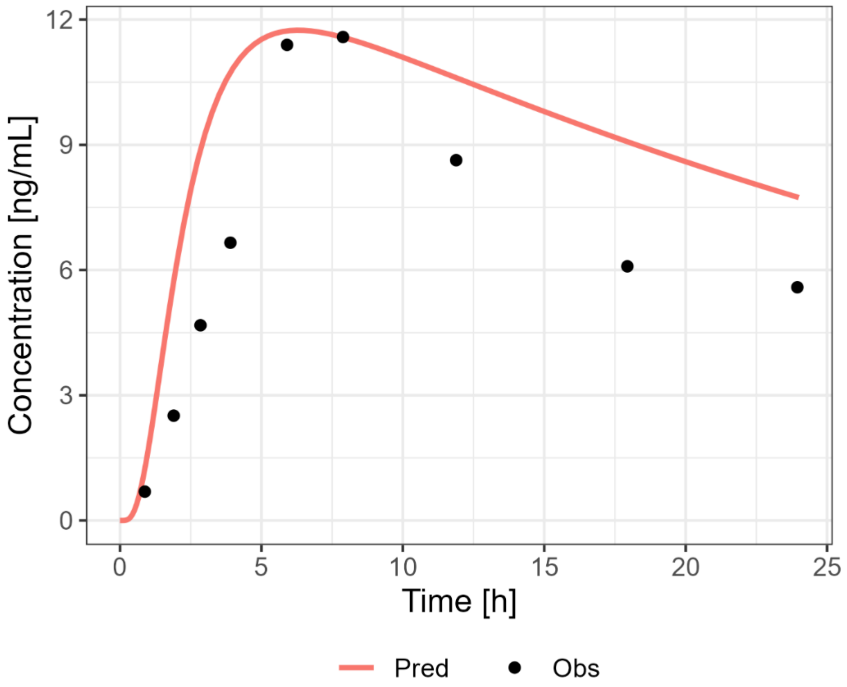

Figure S7 Predicted (Pred) versus observed (Obs) concentration-time profile after administration of 50 mg PO [20]

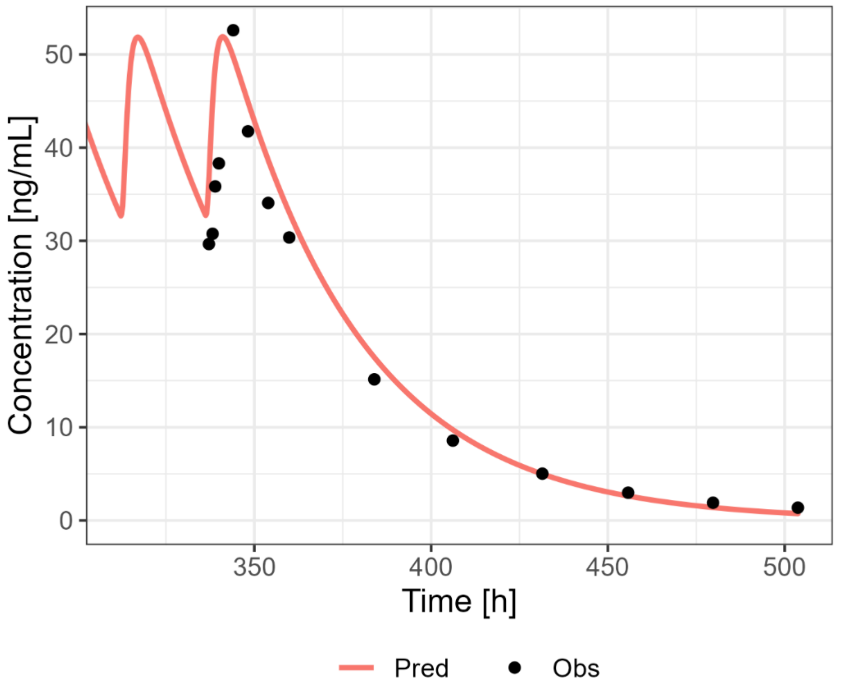

Figure S8 Predicted (Pred) versus observed (Obs) concentration-time profile after administration of 100 mg PO [20]

4.3.2 Model verification

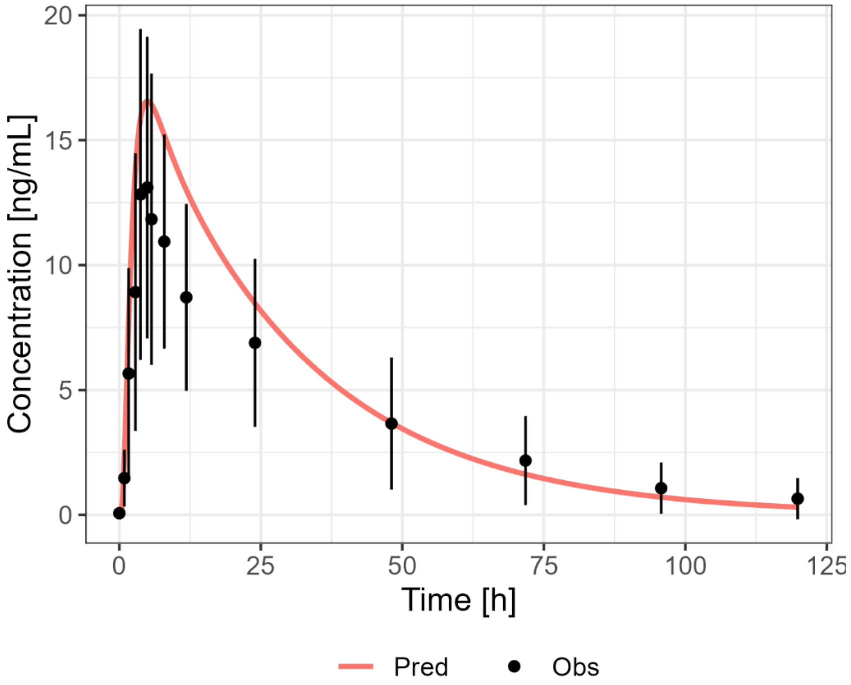

Figure S9 Predicted (Pred) versus observed (Obs) concentration-time profile after administration of 50 mg PO reference [24]

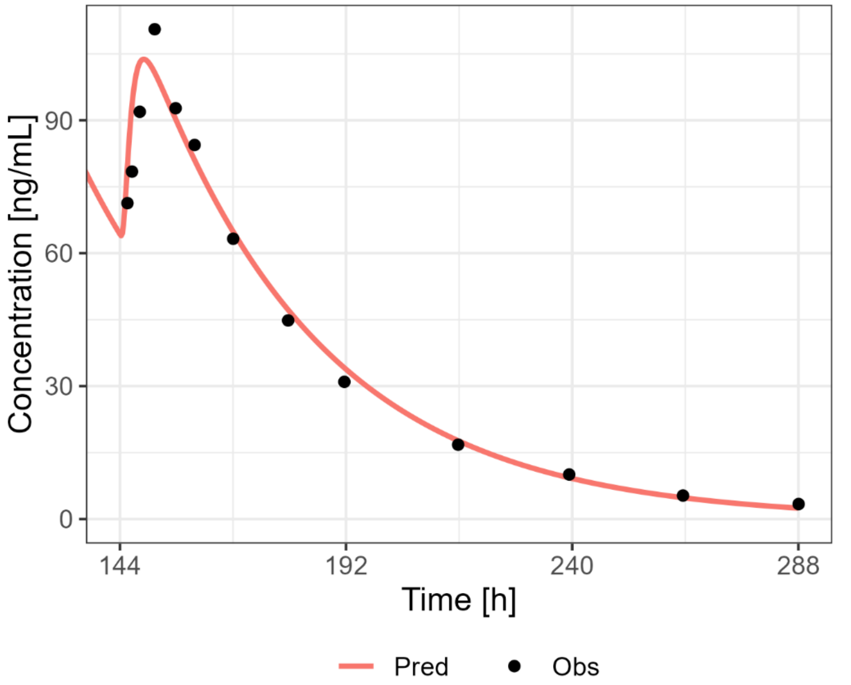

Figure S10 Predicted (Pred) versus observed (Obs) concentration-time profile after administration of 200 mg/day male PO [14]

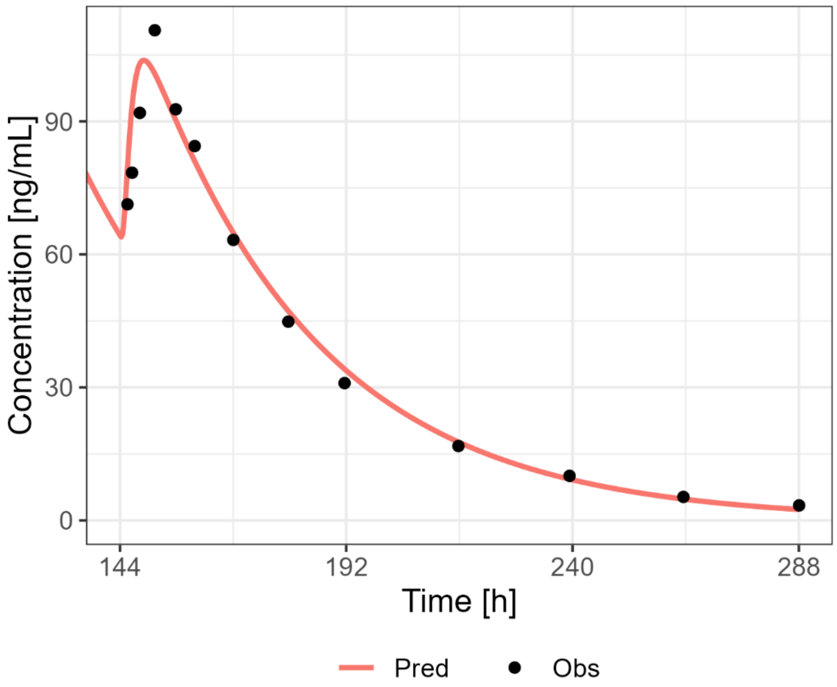

Figure S11 Predicted (Pred) versus observed (Obs) concentration-time profile after administration of 200 mg/day female PO [14]

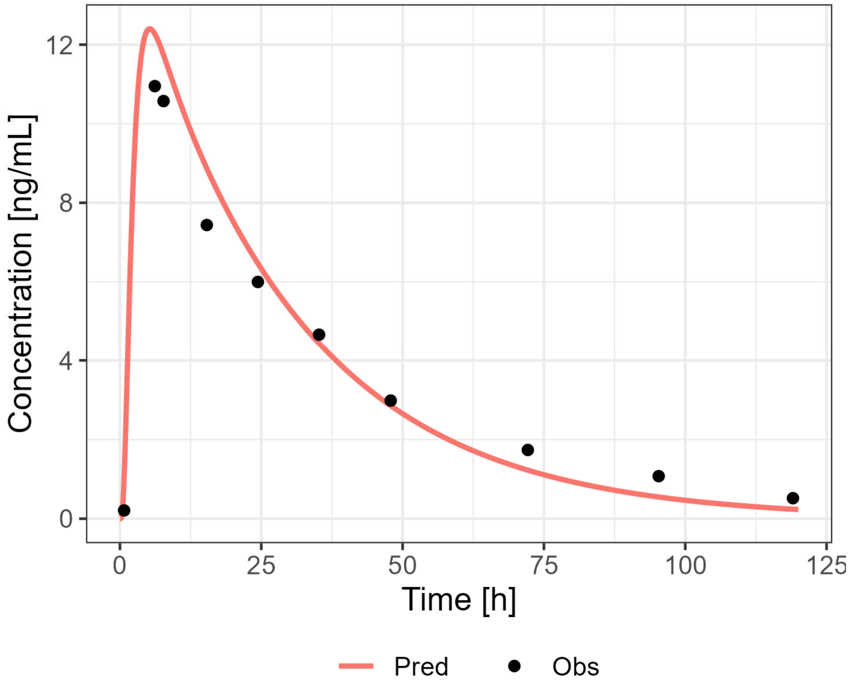

Figure S12 Predicted (Pred) versus observed (Obs) concentration-time profile after administration of 50 mg PO [14]

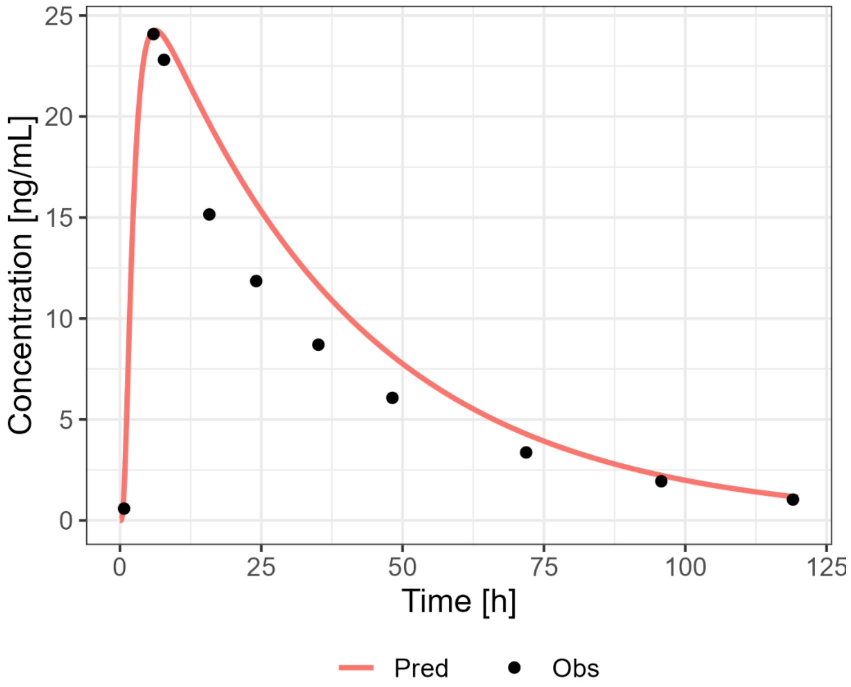

Figure S13 Predicted (Pred) versus observed (Obs) concentration-time profile after administration of 100 mg PO [14]

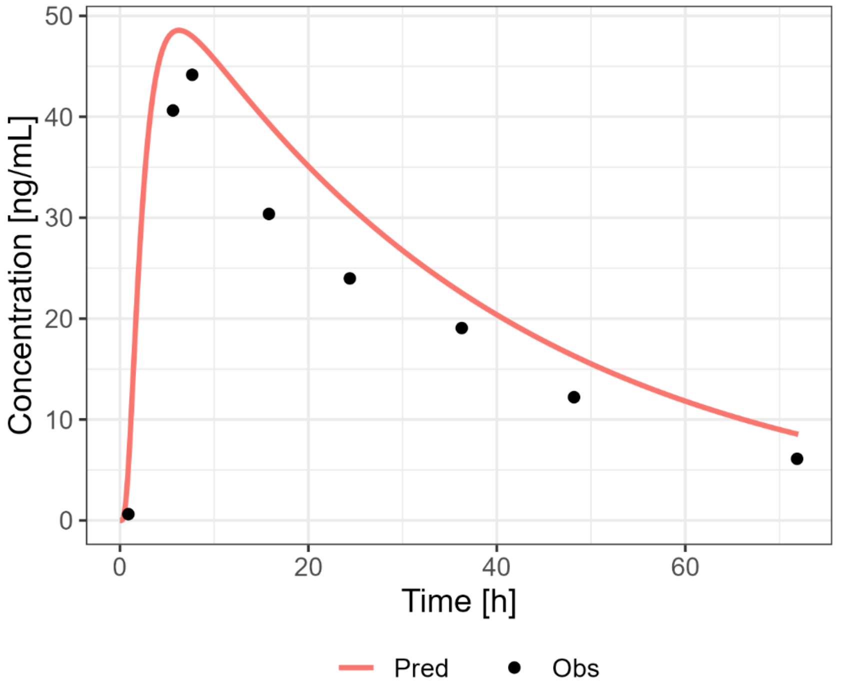

Figure S14 Predicted (Pred) versus observed (Obs) concentration-time profile after administration of 200 mg PO [14]

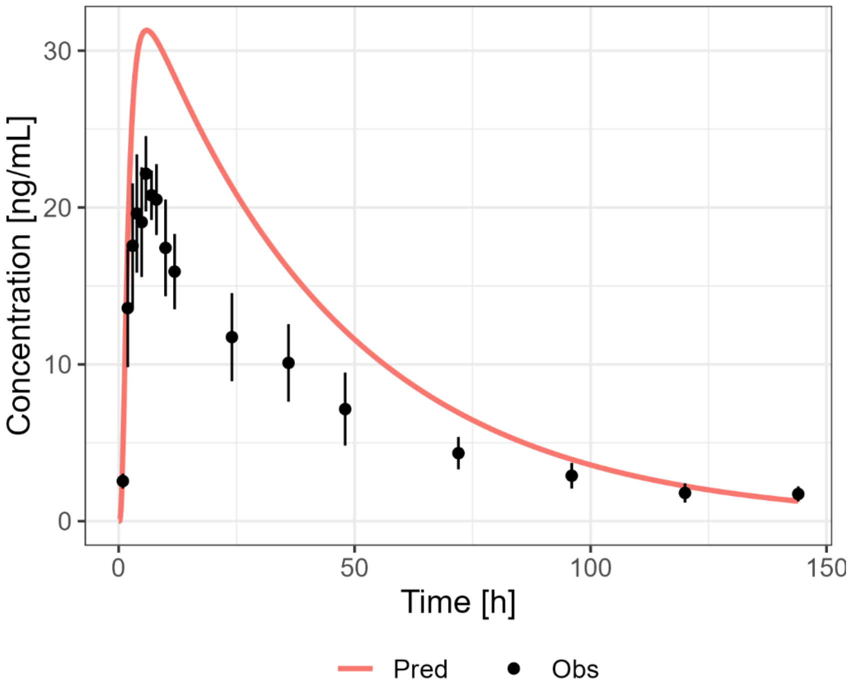

Figure S15 Predicted (Pred) versus observed (Obs) concentration-time profile after administration of 100 mg PO PM [23]

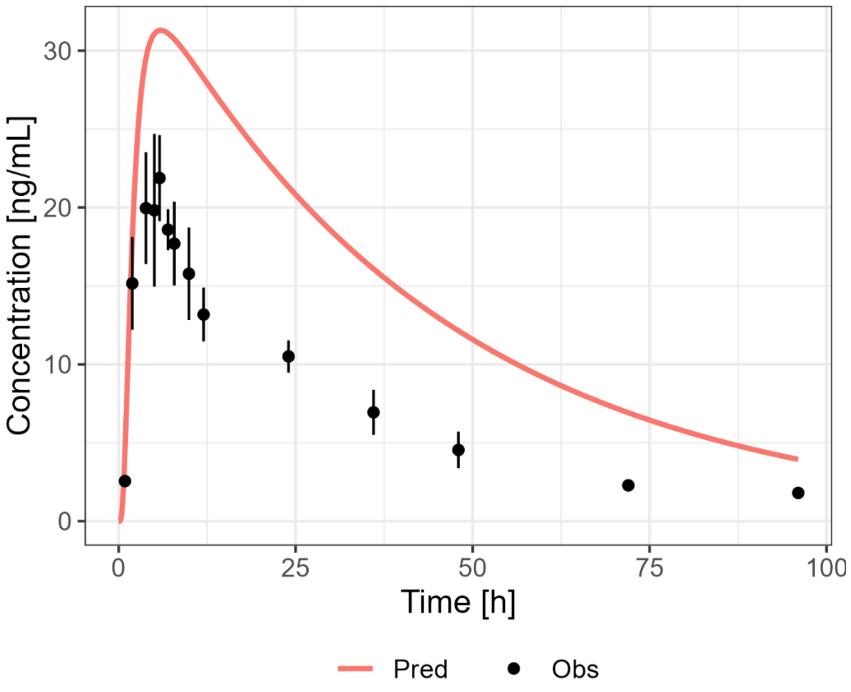

Figure S16 Predicted (Pred) versus observed (Obs) concentration-time profile after administration of 100 mg PO EM [23]

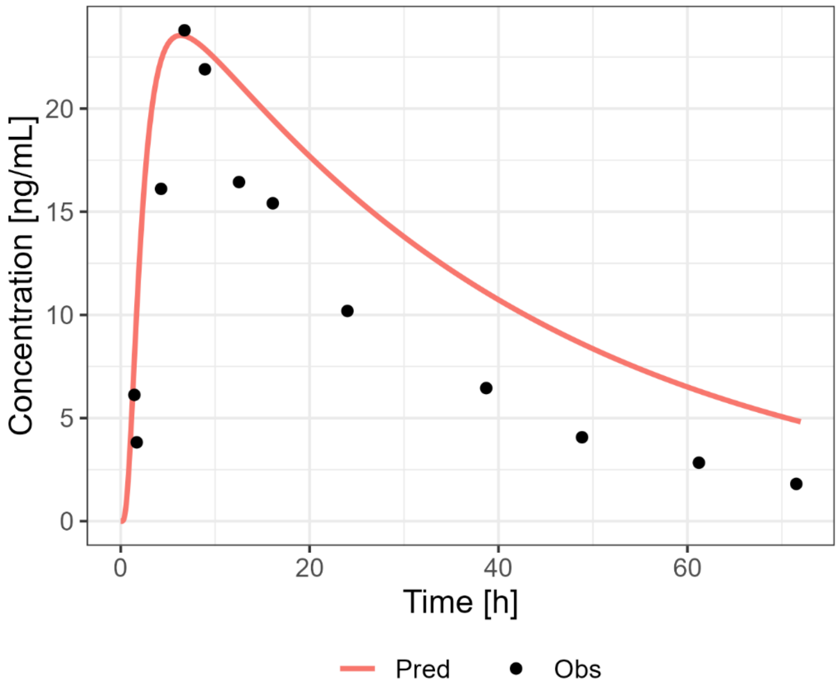

Figure S17 Predicted (Pred) versus observed (Obs) concentration-time profile after administration of 100 mg morning PO [21]

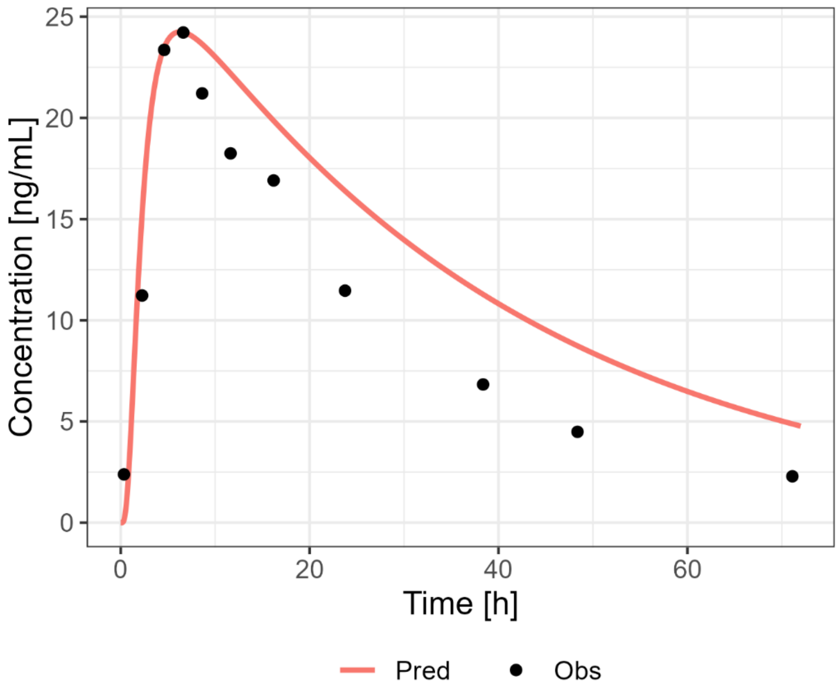

Figure S18 Predicted (Pred) versus observed (Obs) concentration-time profile after administration of 100 mg PO fed [21]

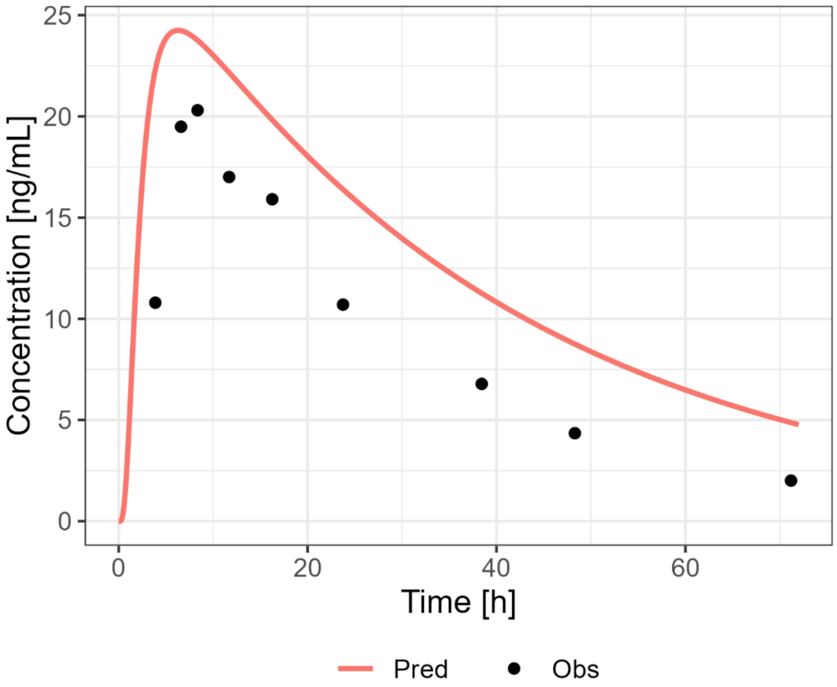

Figure S19 Predicted (Pred) versus observed (Obs) concentration-time profile after administration of 100 mg PO fasted [21]

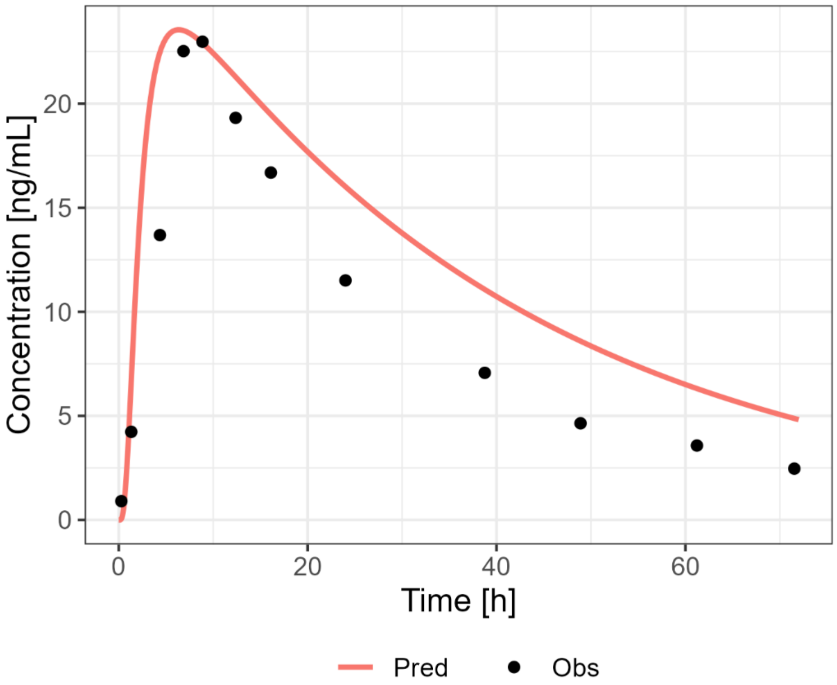

Figure S20 Predicted (Pred) versus observed (Obs) concentration-time profile after administration of 100 mg PO evening [21]

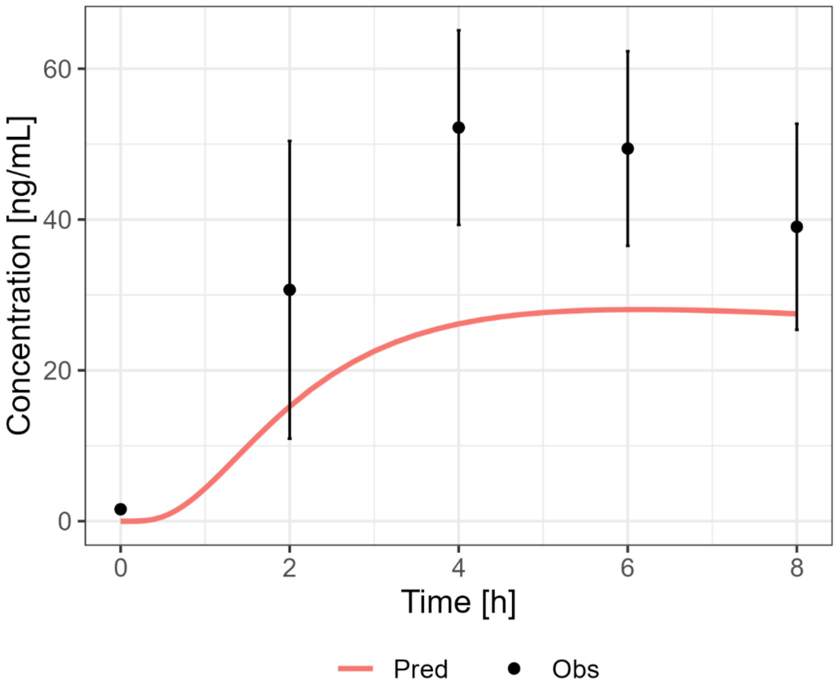

Figure S21 Predicted (Pred) versus observed (Obs) concentration-time profile after administration of 100 mg PO [16]

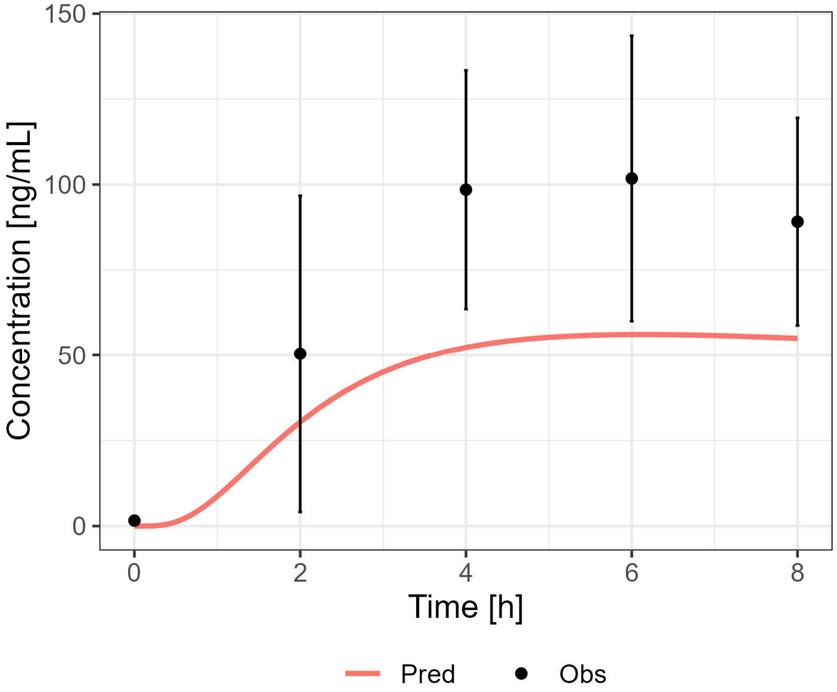

Figure S22 Predicted (Pred) versus observed (Obs) concentration-time profile after administration of 200 mg PO [16]

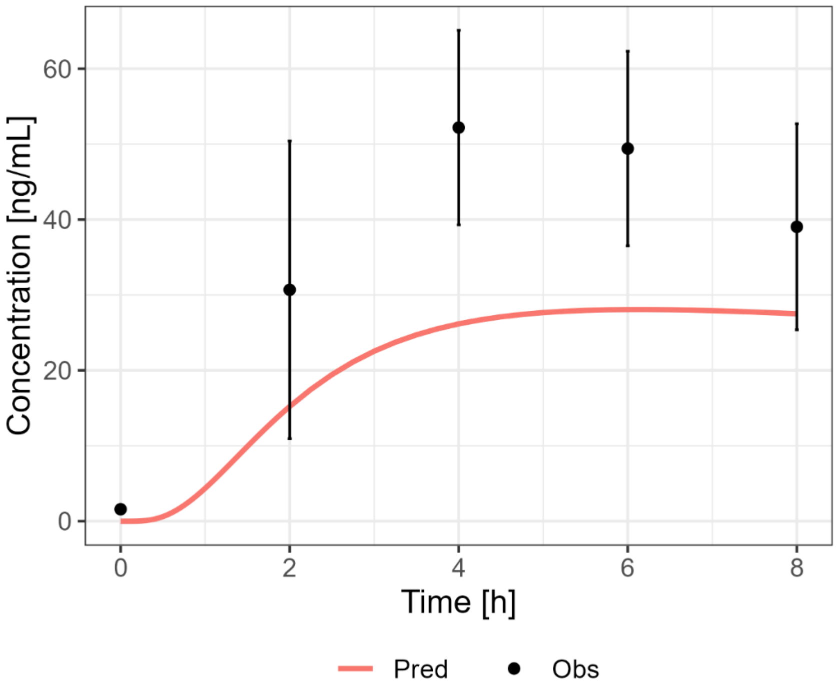

Figure S23 Predicted (Pred) versus observed (Obs) concentration-time profile after administration of 400 mg PO [16]

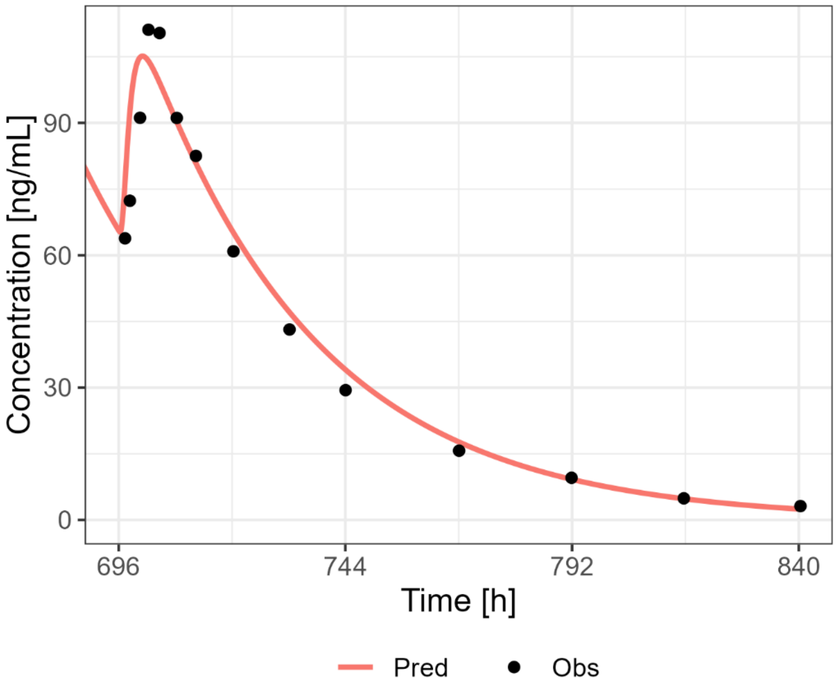

Figure S24 Predicted (Pred) versus observed (Obs) concentration-time profile after administration of 200 mg/day male PO [22]

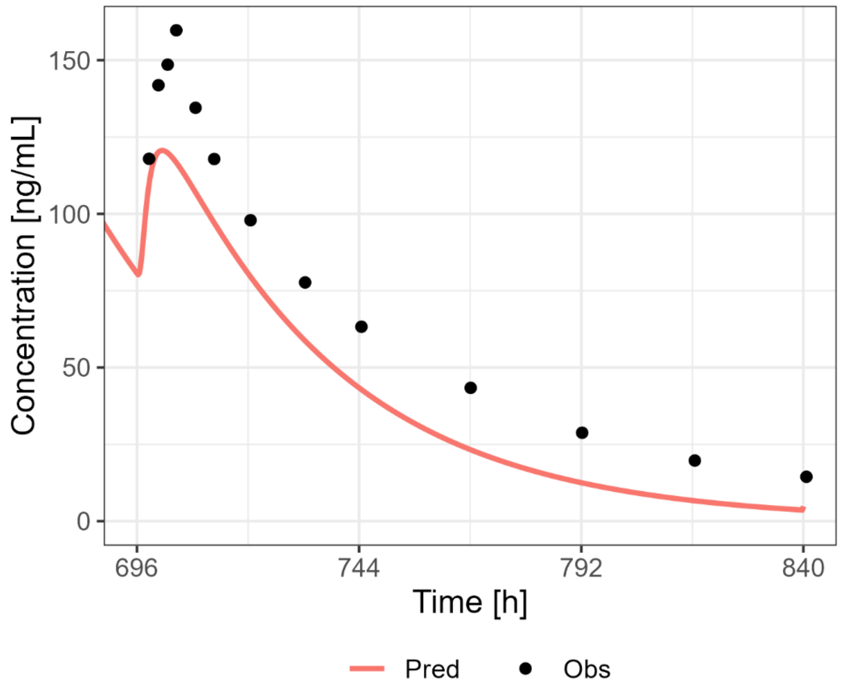

Figure S25 Predicted (Pred) versus observed (Obs) concentration-time profile after administration of 200 mg/day female PO [22]

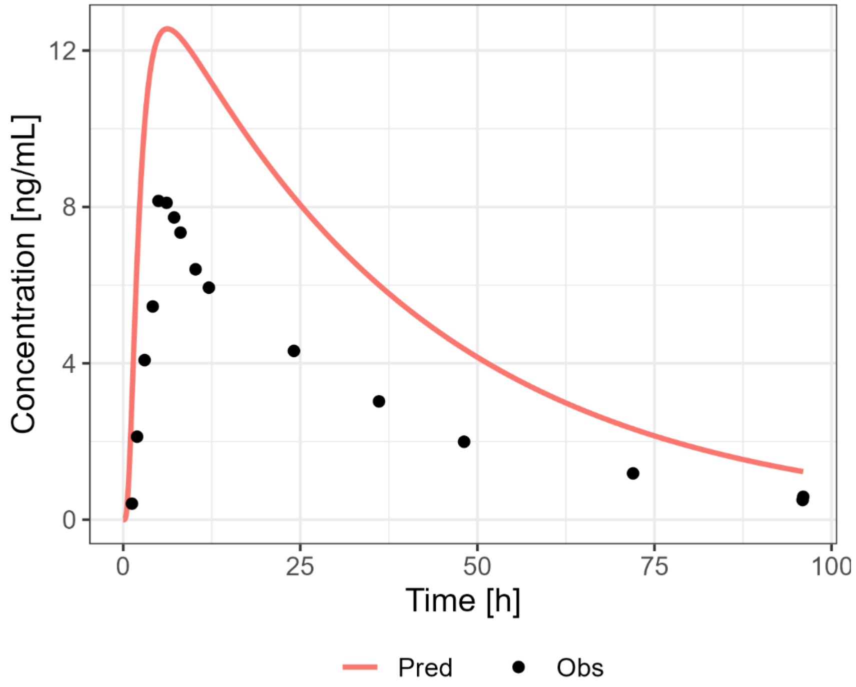

Figure S26 Predicted (Pred) versus observed (Obs) concentration-time profile after administration of 50 mg PO test capsule [19]

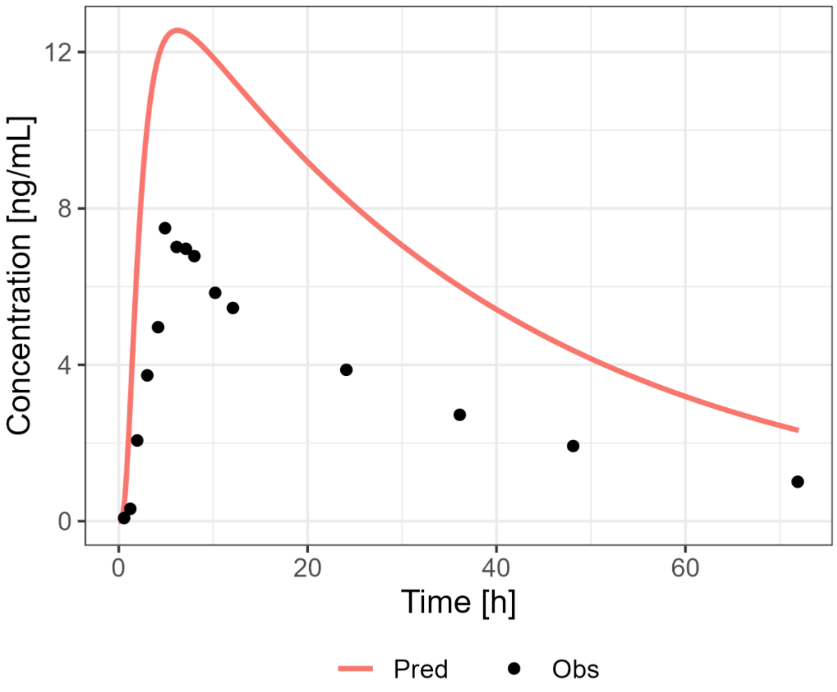

Figure S27 Predicted (Pred) versus observed (Obs) concentration-time profile after administration of 50 mg PO reference capsule [19]

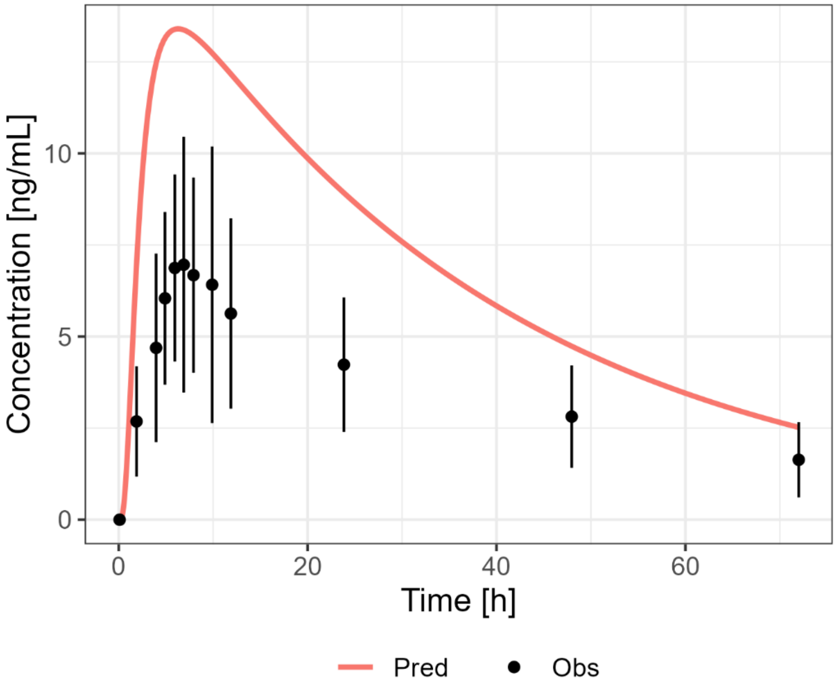

Figure S28 Predicted (Pred) versus observed (Obs) concentration-time profile after administration of 50 mg test PO [18]

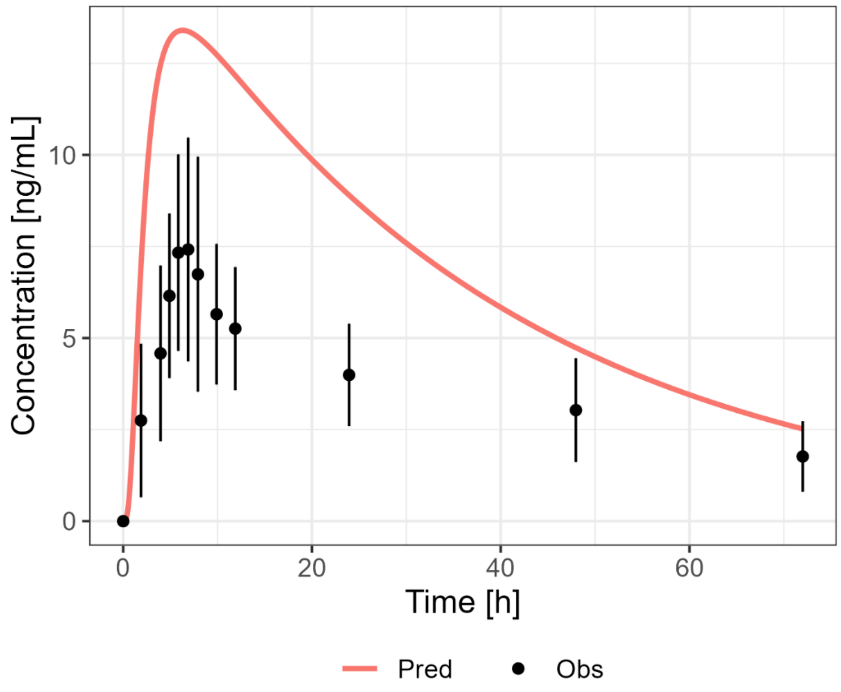

Figure S29 Predicted (Pred) versus observed (Obs) concentration-time profile after administration of 50 mg reference PO [18]

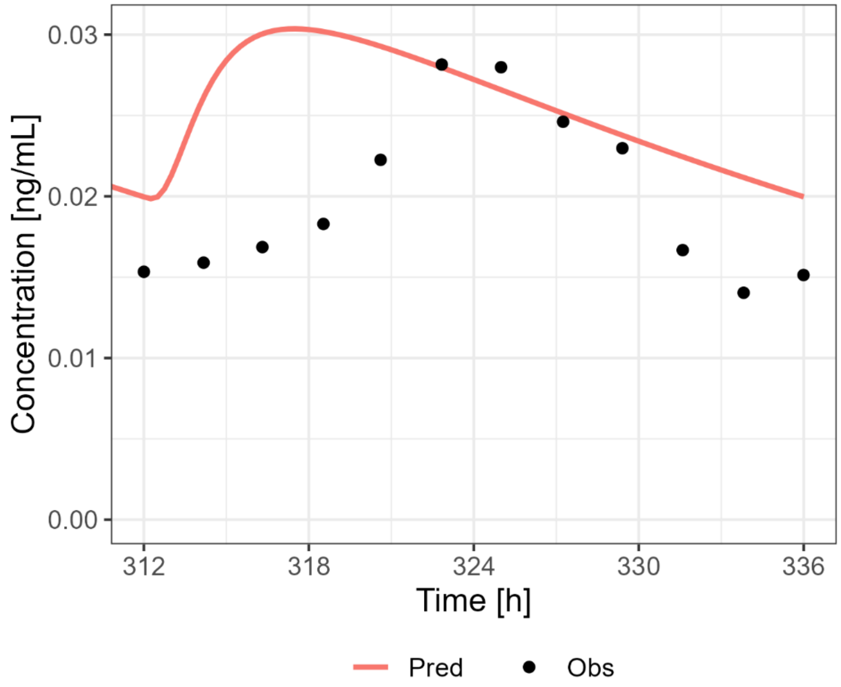

Figure S30 Predicted (Pred) versus observed (Obs) concentration-time profile after administration of 50 mg/day PO [17]

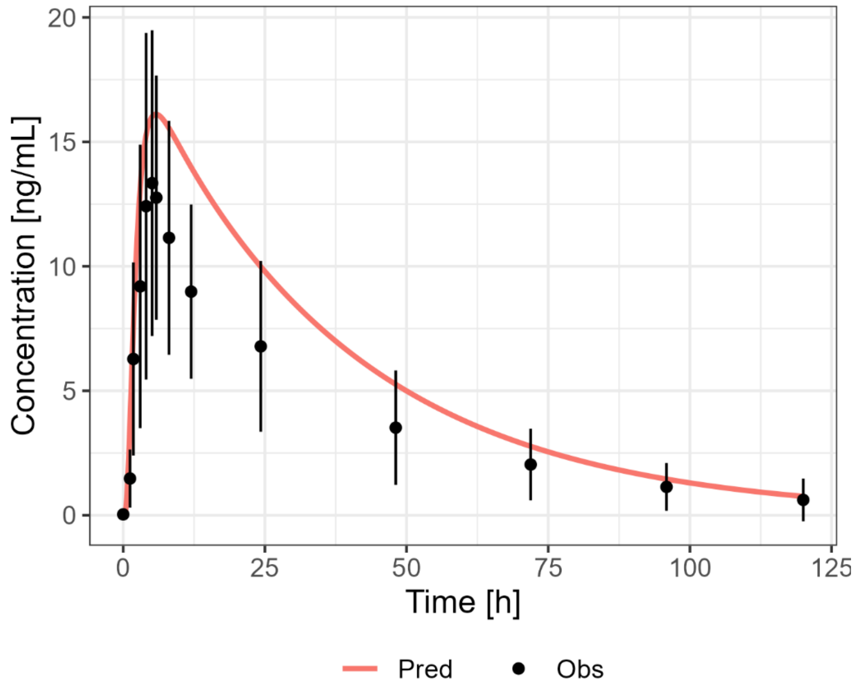

Figure S31 Predicted (Pred) versus observed (Obs) concentration-time profile after administration of 50 mg PO test [24]

4.3.3 Lactation PBPK model

A sample size of 1000 individuals, three months postpartum, was used in each simulation of the virtual lactation population.

<u>Model A</u>: First, the transfer of sertraline was predicted using the LogP value of 0.5.50, and the default values for logD based on the equations implemented in the spatial structure building block as described above.

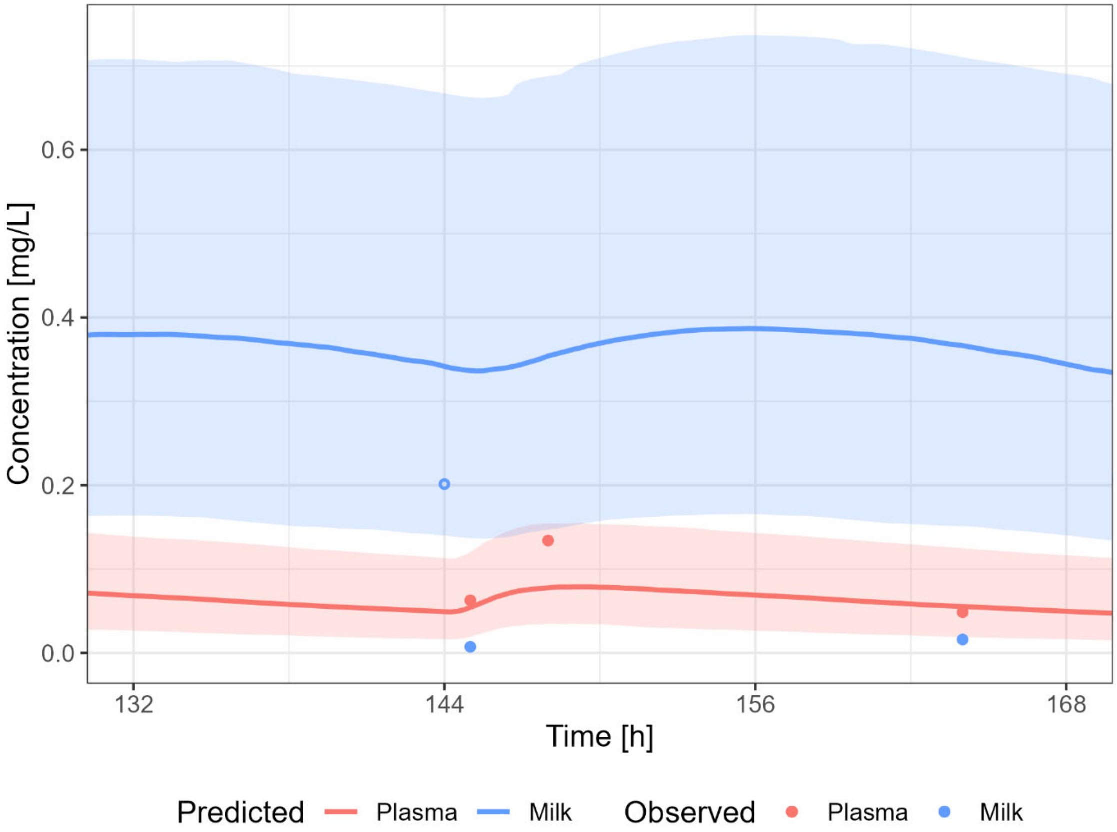

Figure S32 Predicted (Pred) versus observed (Obs) concentration-time profile after administration of 150 mg/day [25,28,35]

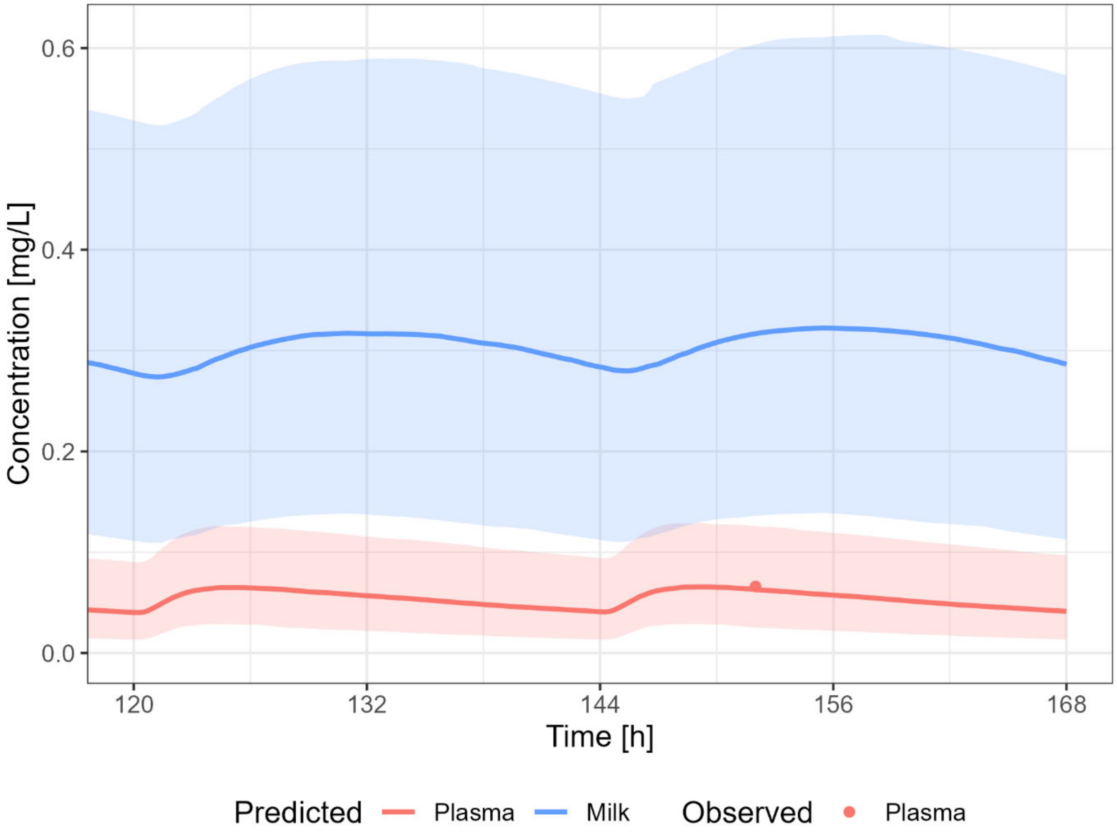

Figure S33 Predicted (Pred) versus observed (Obs) concentration-time profile after administration of 125 mg/day [35]

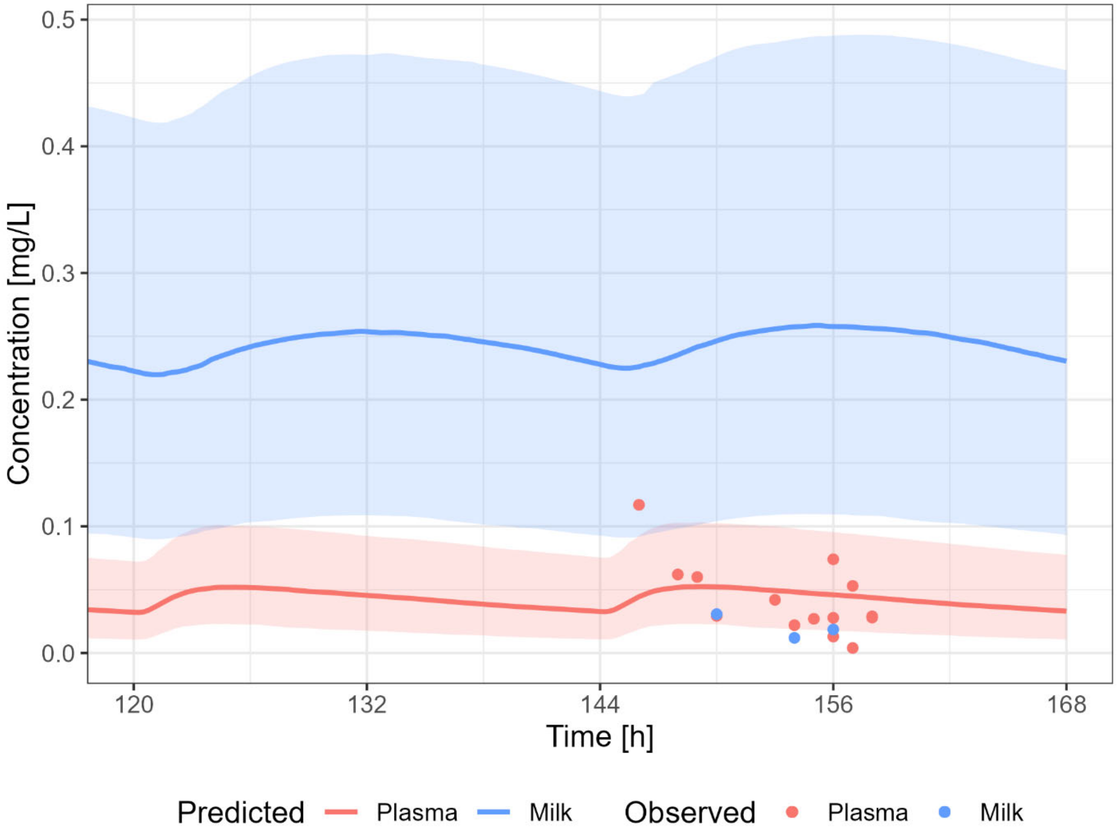

Figure S34 Predicted (Pred) versus observed (Obs) concentration-time profile after administration of 100 mg/day [25,27,35]

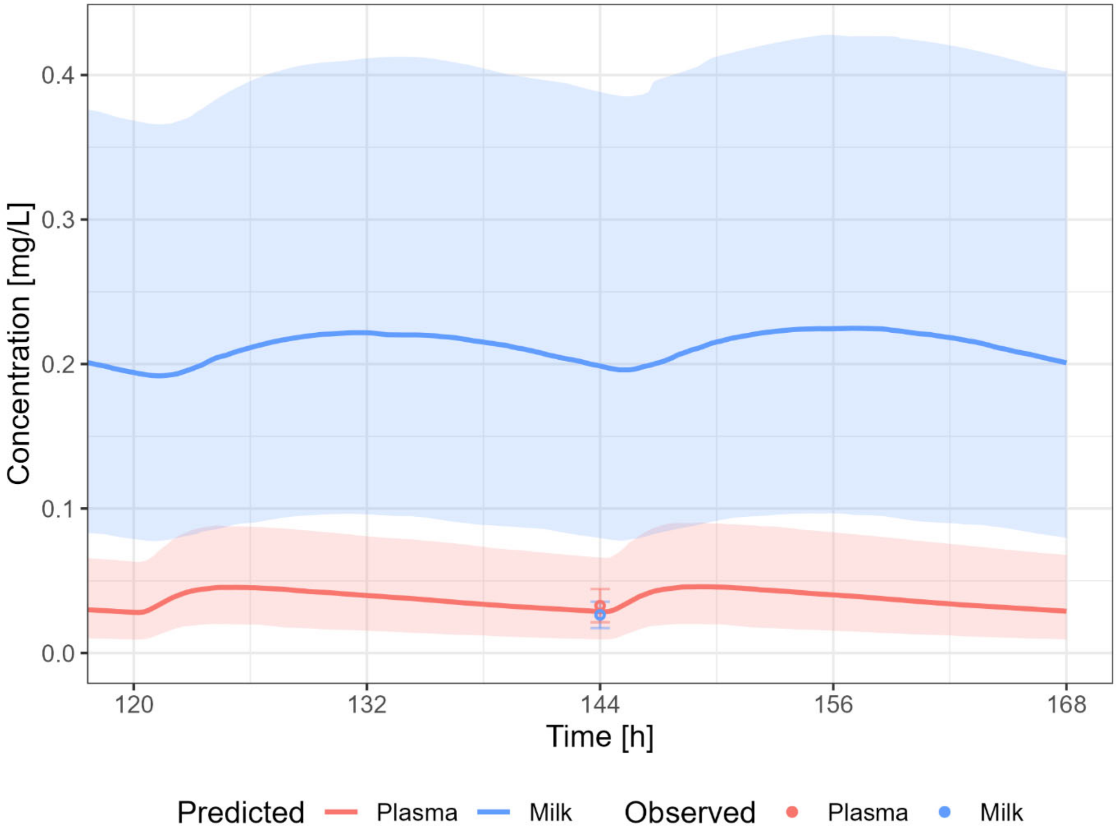

Figure S35 Predicted (Pred) versus observed (Obs) concentration-time profile after administration of 87.5 mg/day [29]

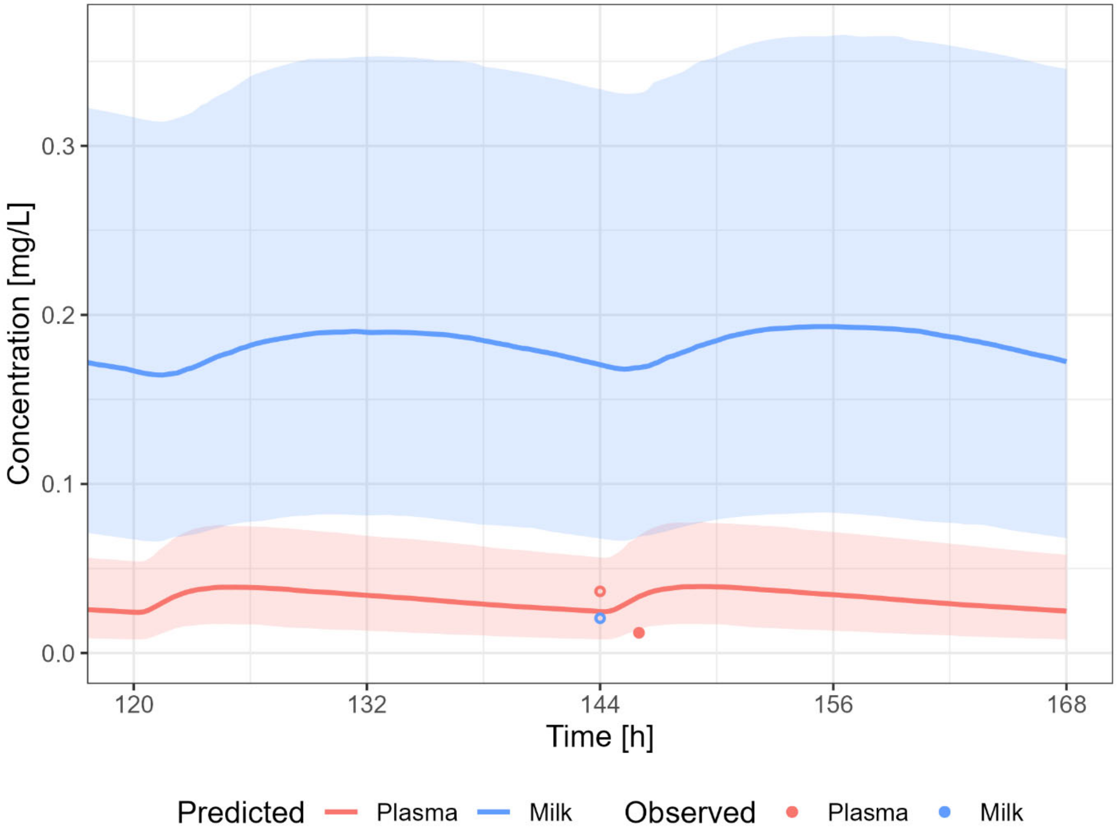

Figure S36 Predicted (Pred) versus observed (Obs) concentration-time profile after administration of 75 mg/day [31,35]

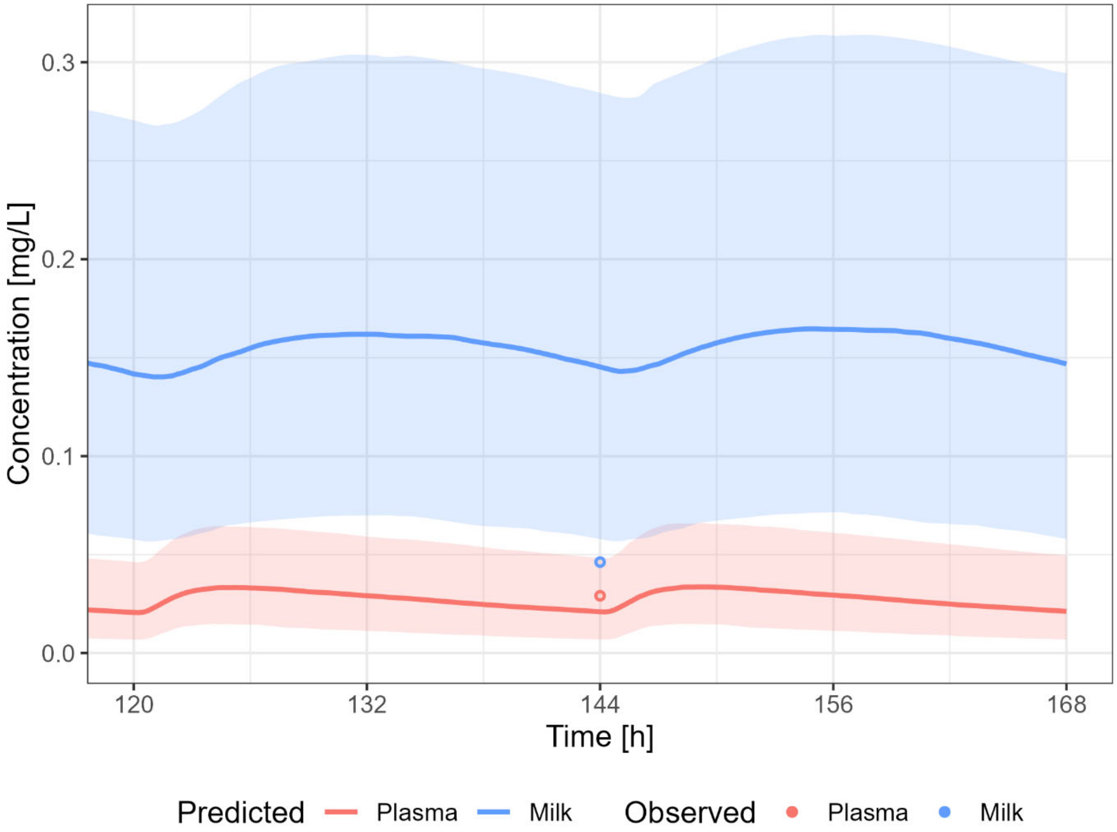

Figure S37 Predicted (Pred) versus observed (Obs) concentration-time profile after administration of 64 mg/day [30]

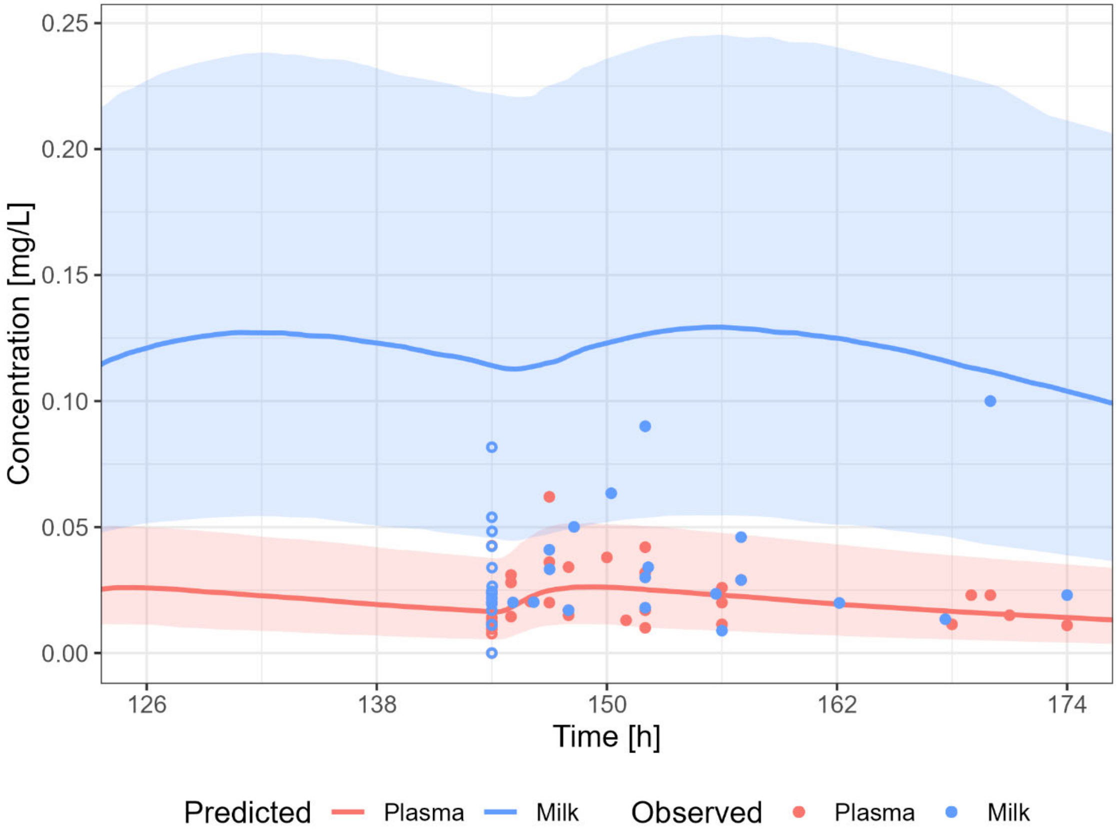

Figure S38 Predicted (Pred) versus observed (Obs) concentration-time profile after administration of 50 mg/day [25–27,31–35]

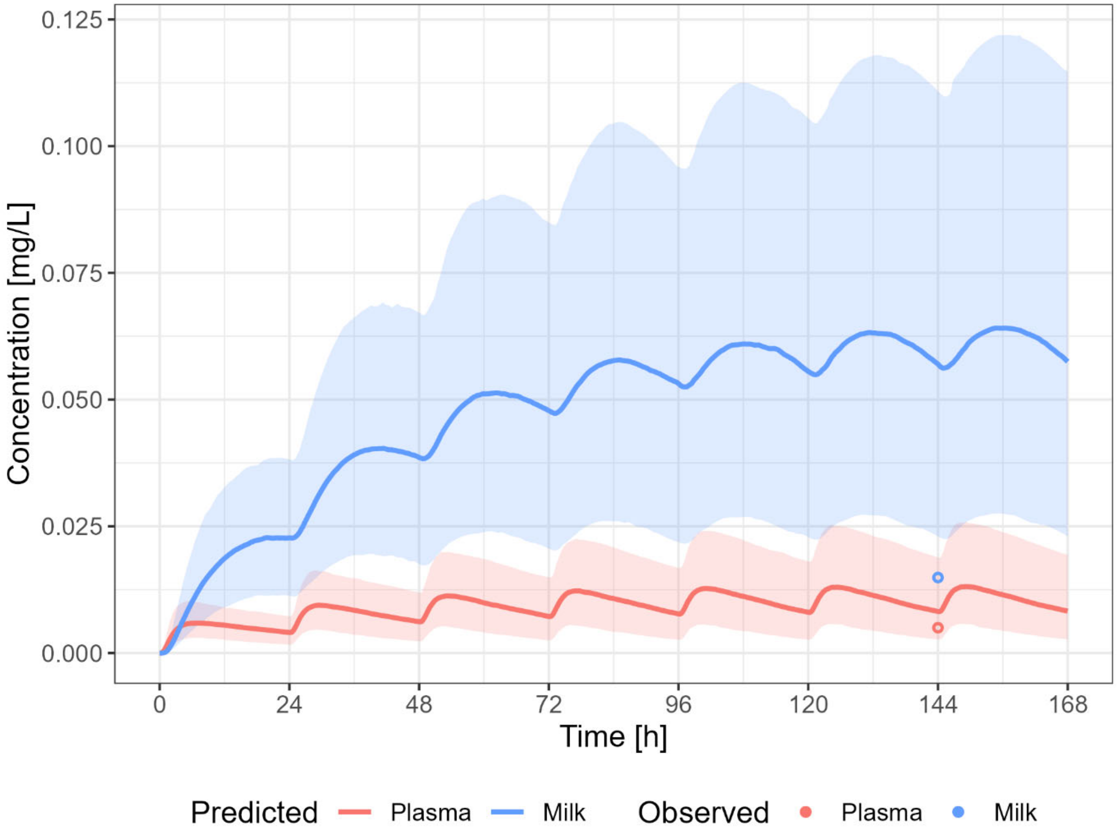

Figure S39 Predicted (Pred) versus observed (Obs) concentration-time profile after administration of 25 mg/day [31]

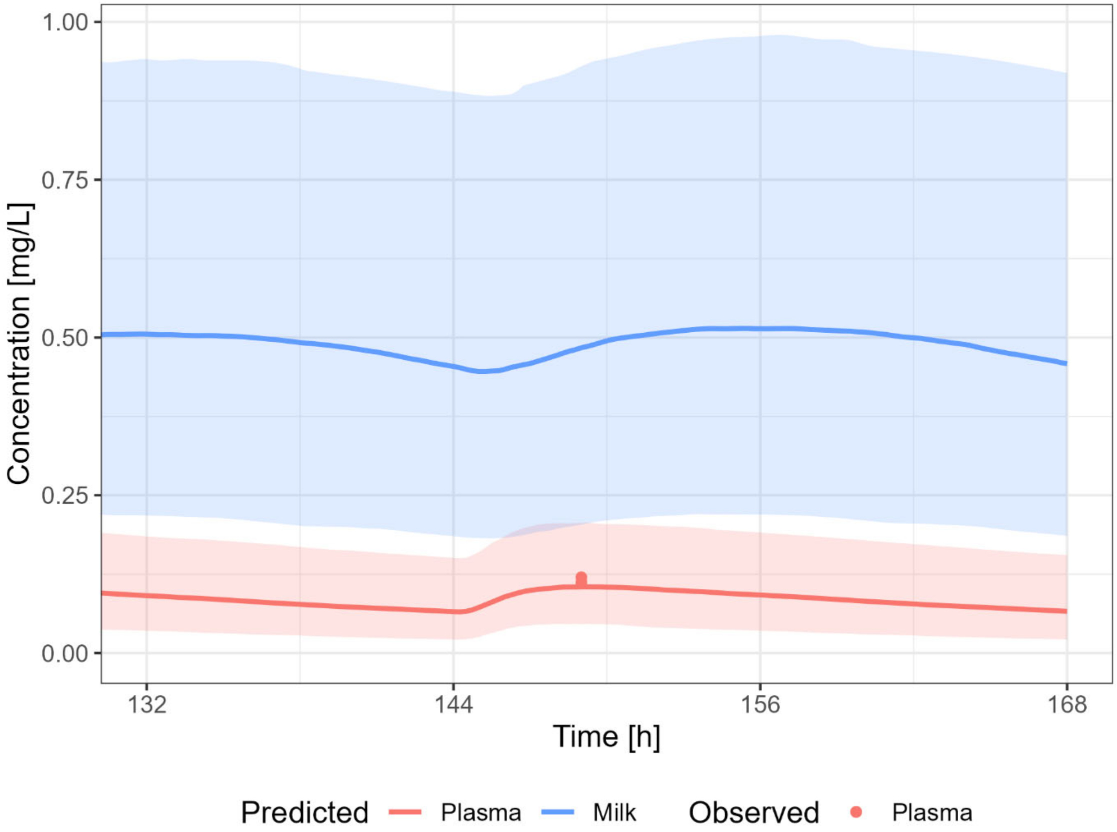

Figure S40 Predicted (Pred) versus observed (Obs) concentration-time profile after administration of 200 mg/day [35]

A dosing regimen of PO 50 mg daily was used to calculate the milk transfer of sertraline.

Dosing interval: 24 h	Plasma	Milk
C_{max} (mg/L)	0.03	0.13
AUC (mg*h/L)	0.52	2.90
Cave (mg/L)	0.02	0.12

M/P ratio, sertraline = 5.58

<u>Model B:</u> Alternatively, the LogP value as well was overwritten with the value obtained from MarvinSketch (see section 4.2).

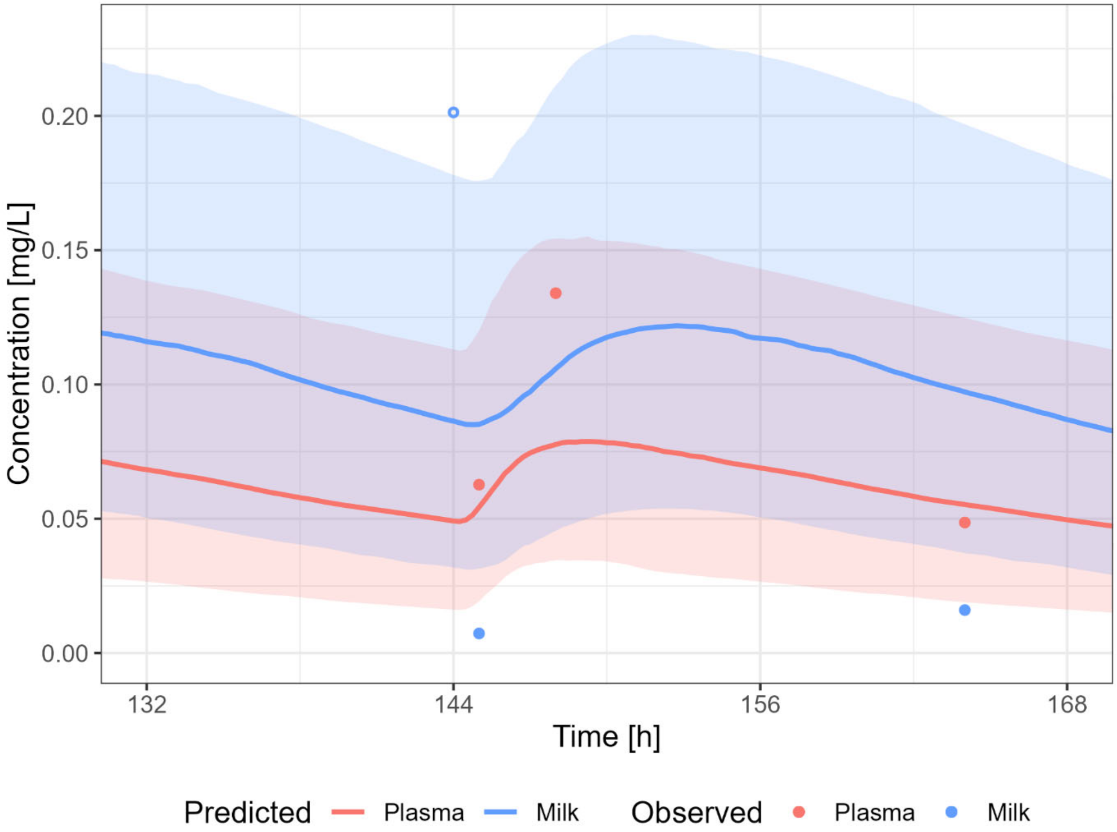

Figure S41 Predicted (Pred) versus observed (Obs) concentration-time profile after administration of 150 mg/day [25,28,35]

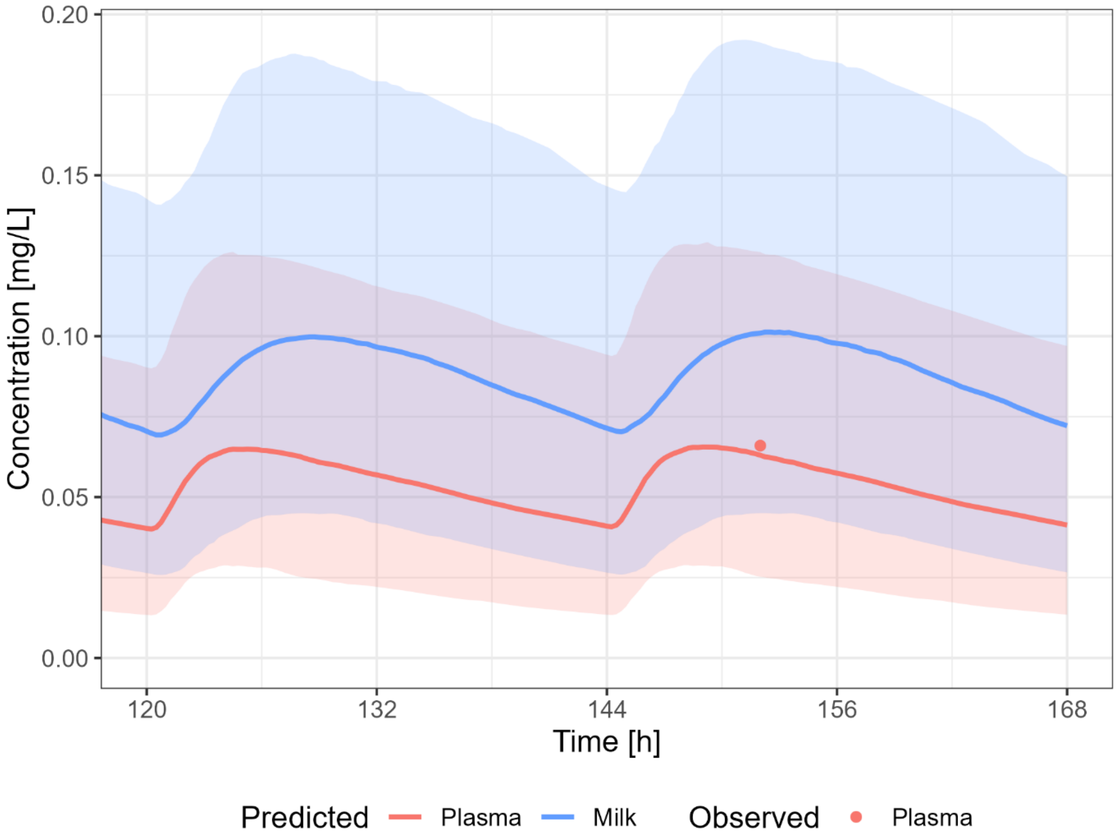

Figure S42 Predicted (Pred) versus observed (Obs) concentration-time profile after administration of 125 mg/day [35]

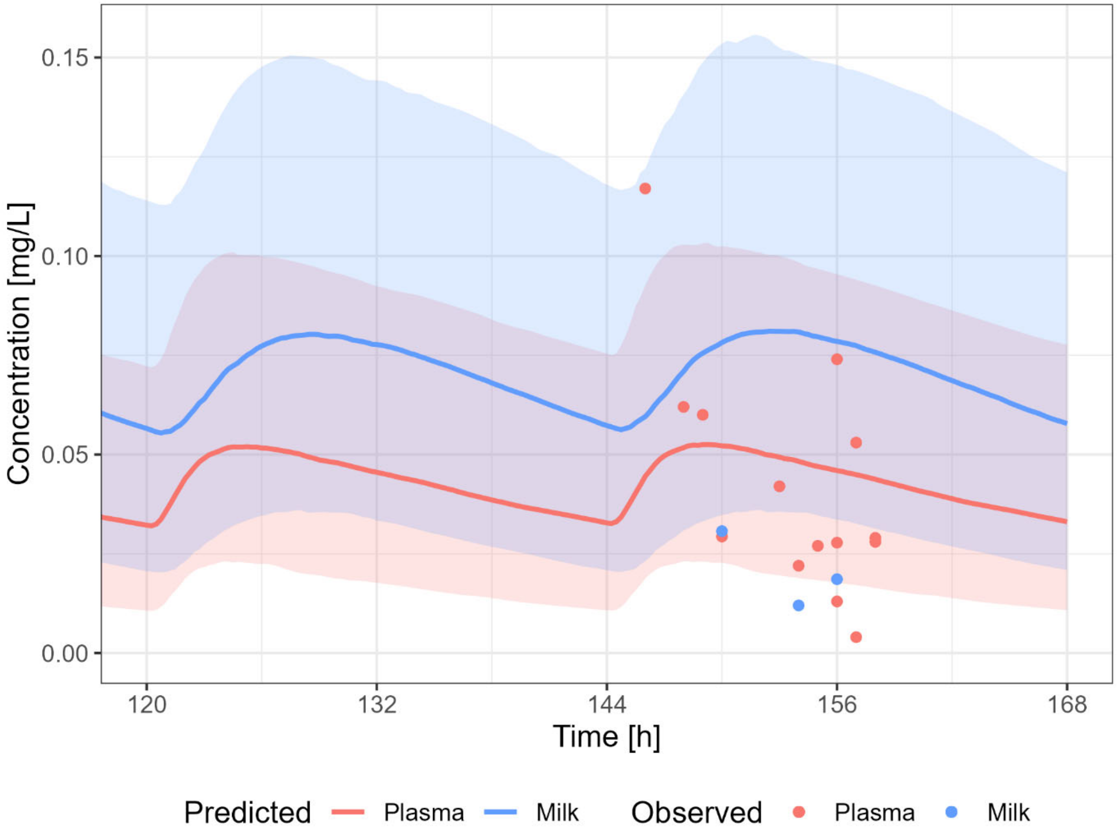

Figure S43 Predicted (Pred) versus observed (Obs) concentration-time profile after administration of 100 mg/day [25,27,35]

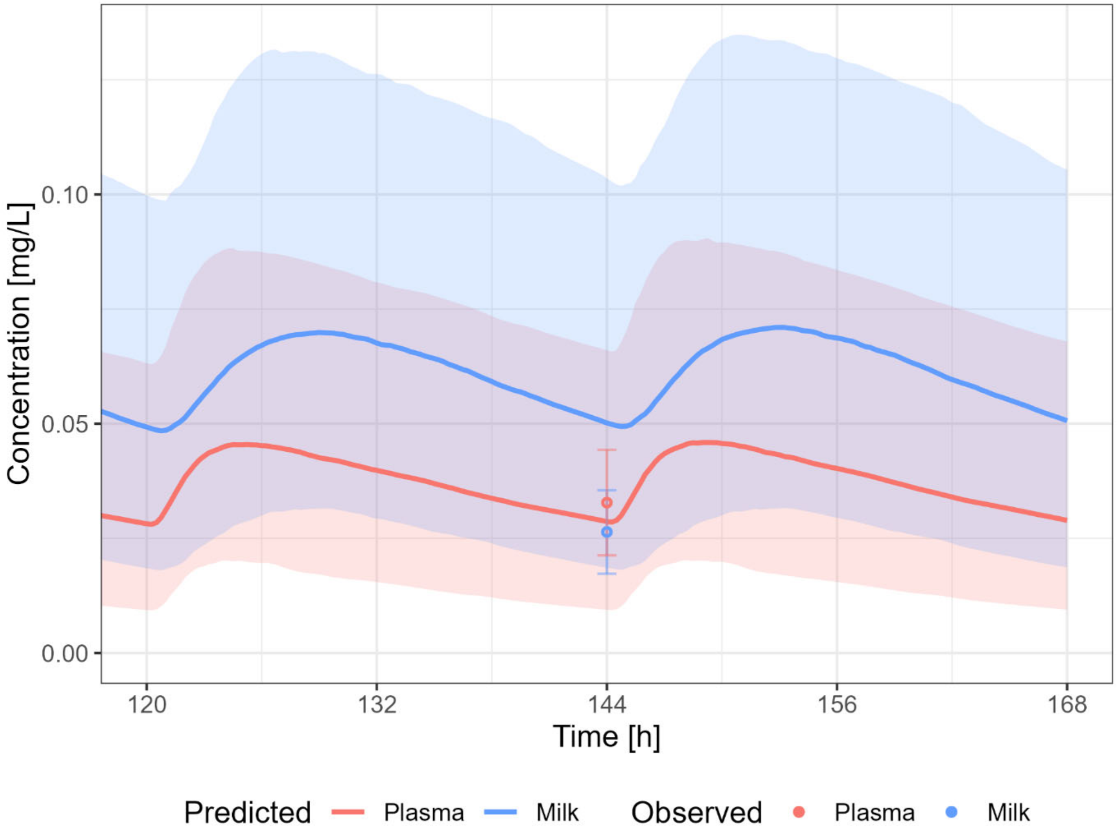

Figure S44 Predicted (Pred) versus observed (Obs) concentration-time profile after administration of 87.5 mg/day [29]

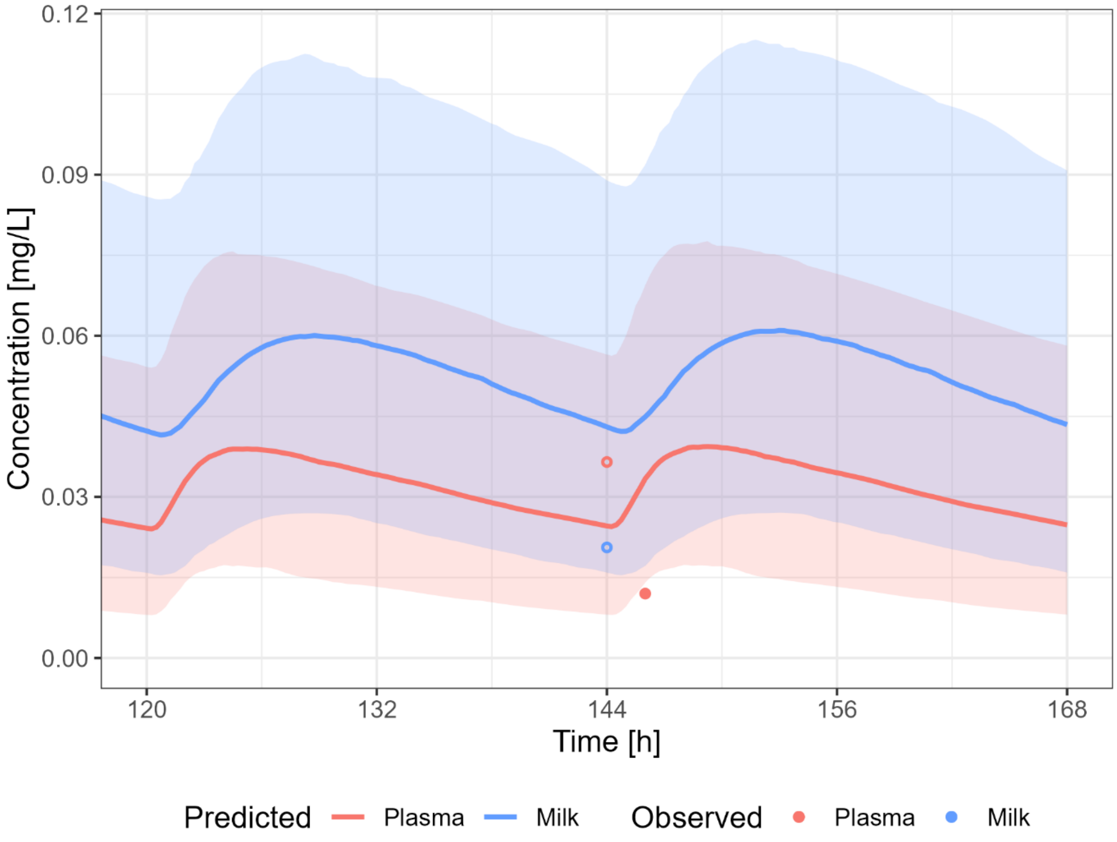

Figure S45 Predicted (Pred) versus observed (Obs) concentration-time profile after administration of 75 mg/day [31,35]

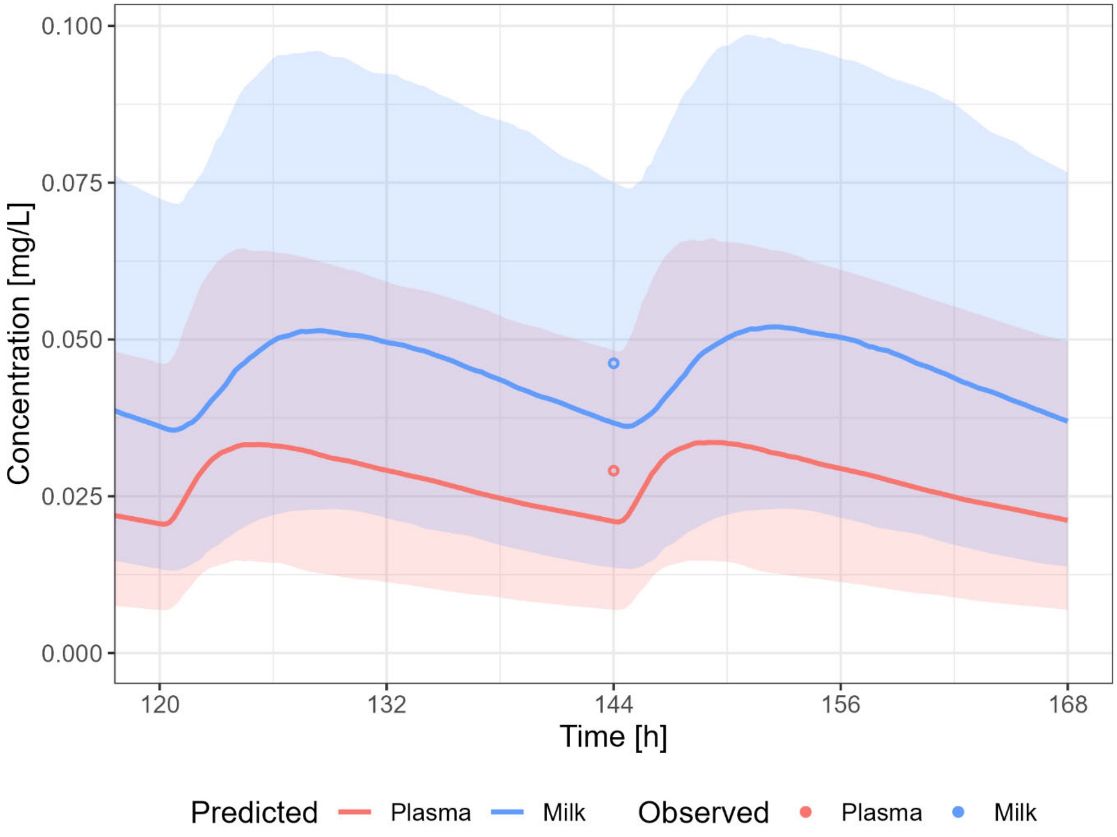

Figure S46 Predicted (Pred) versus observed (Obs) concentration-time profile after administration of 64 mg/day [30]

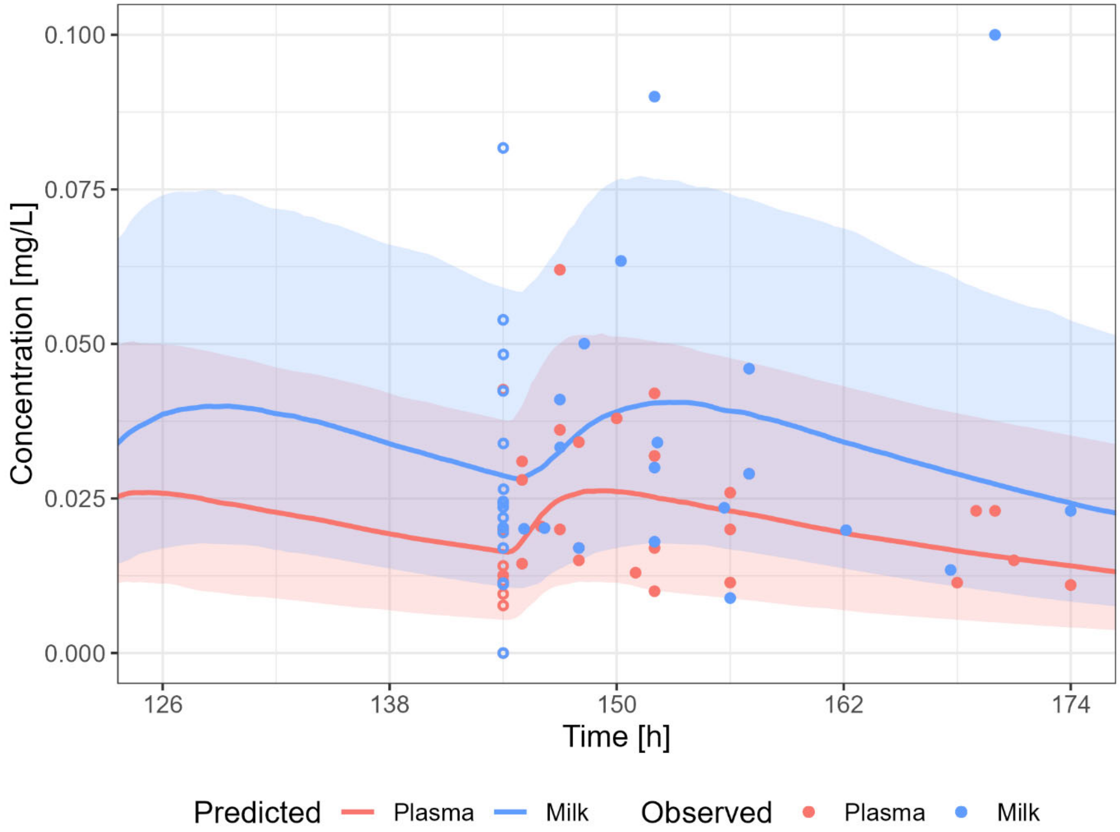

Figure S47 Predicted (Pred) versus observed (Obs) concentration-time profile after administration of 50 mg/day [25–27,31–35]

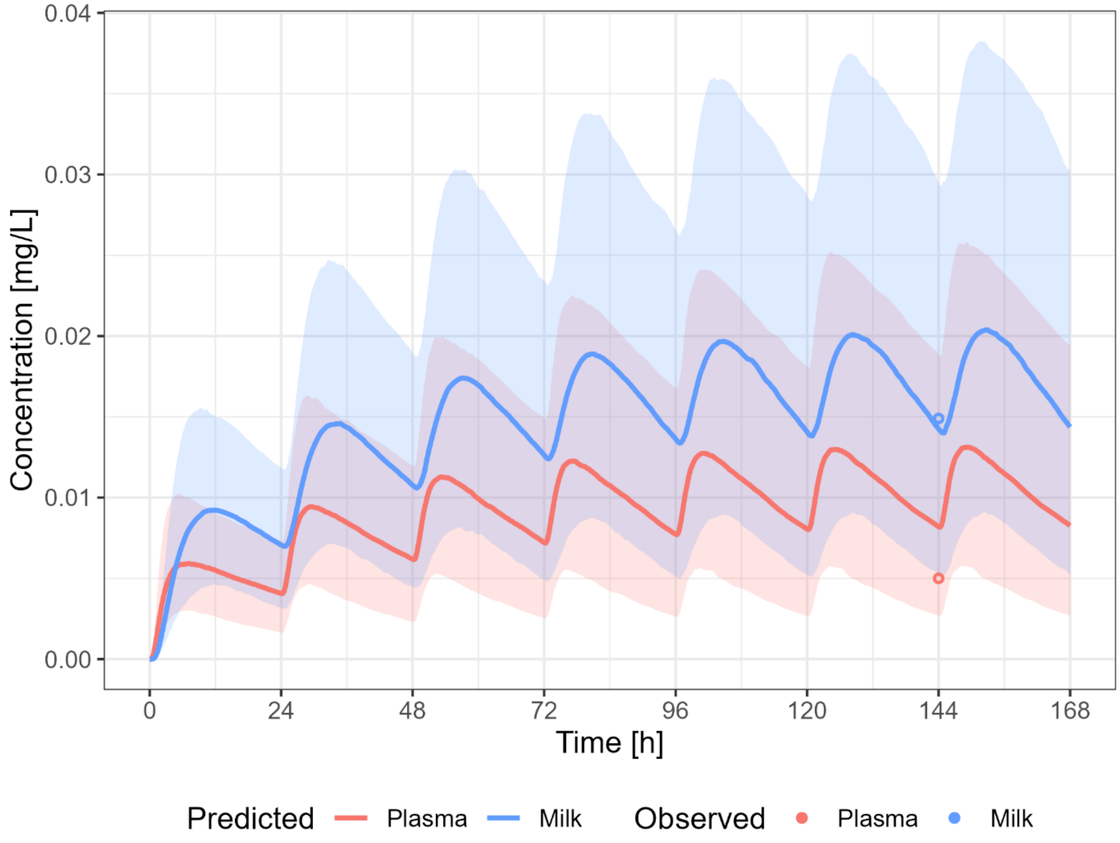

Figure S48 Predicted (Pred) versus observed (Obs) concentration-time profile after administration of 25 mg/day [31]

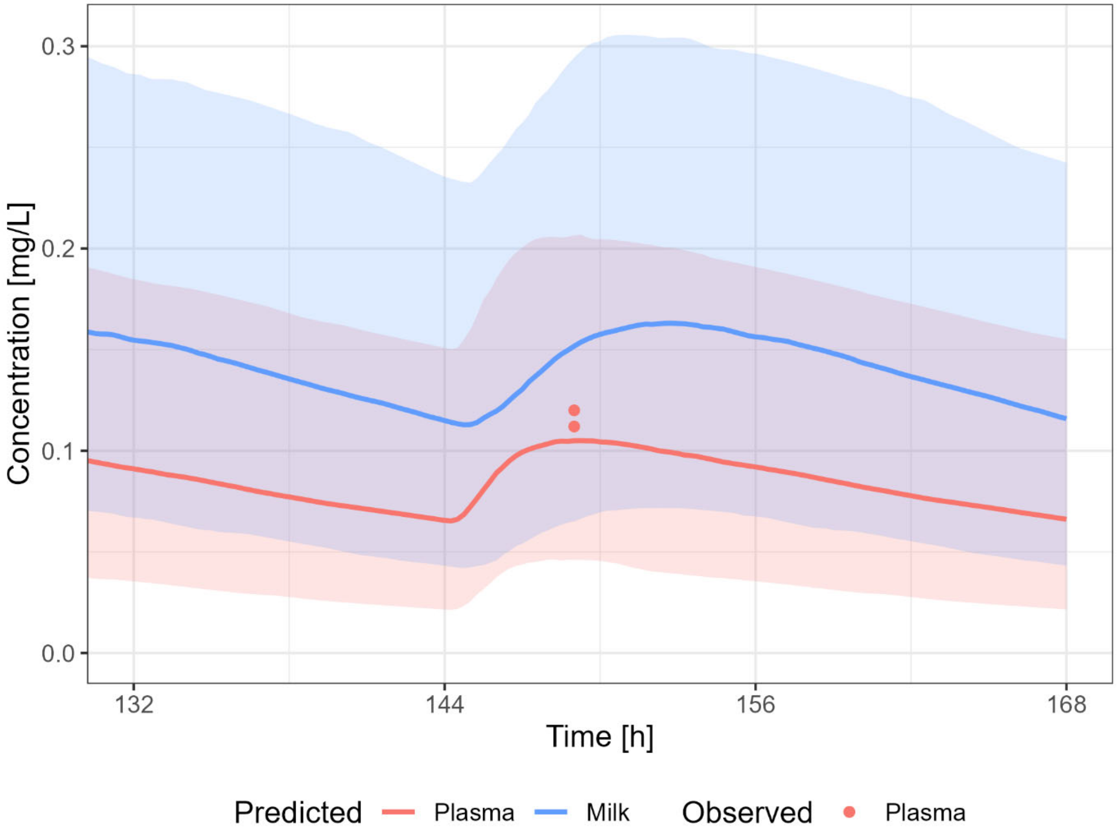

Figure S49 Predicted (Pred) versus observed (Obs) concentration-time profile after administration of 200 mg/day [35]

A dosing regimen of PO 50 mg daily was used to calculate the milk transfer of sertraline.

Dosing interval: 24 h	Plasma	Milk
C_{max} (mg/L)	0.03	0.04
AUC (mg*h/L)	0.52	0.84
Cave (mg/L)	0.02	0.04

M/P ratio, sertraline = 1.62

Model B was selected as final PBPK model for lactation for sertraline. The M/P ratio was 1.62.

4.4 Estimated Infant dosage

A maternal dosing regimen of 50 mg daily was assumed to calculate the infant dosage. The daily infant dosage and relative infant dose (RID) for 3 months old infants for sertraline were calculated using a milk intake of 150 mL/kg/day. The daily infant dosage was 0.005 mg/kg/day (RID: 0.63 %) or 0.01 mg/kg/day (RID: 0.72 %) based on the average steady-state concentration and maximum concentration in human milk, respectively.

5. Discussion

First, the reference PBPK model was developed and evaluated. Evaluation of the predictive performance showed that the reference PBPK model for sertraline was, taken the high variability in the observed data into account, able to capture the pharmacokinetic behavior of the medicines in healthy volunteers and/or patients. The PBPK model was able to adequately predict the AUC and Cmax for 89 % of the simulations for sertraline. Further mechanistic insight (e.g. involvement of different genotypes for CYP enzymes) in the source of the variability could furhter improve the reference PBPK models.

Next, the PBPK model was extended to a lactation PBPK model. The PBPK model results in an acceptable prediction of the human milk concentrations of sertraline, with most datapoints within the 5-95th percentile of the population prediction. It must be noted that there is a huge variability in observed data between different subjects and/or datasets.

The predicted M/P ratio is within the observed range, but it needs to be mentioned that the observed range is very broad (0.12 – 5.2).

The calculated infant dosage of sertraline via breastfeeding was very low, especially when compared to the maternal daily dosage.

6. Conclusions

The herein presented PBPK model adequately describes the PK of sertraline in adults, including breastfeeding women. In particular, it applies quantitative metabolism by CYP2D6, CYP2C9, CYP2B6, CYP2C19, CYP3A4, CYP2E1 and glomerular. The PBPK model was able to predict the human milk concentrations of sertraline (M/P ratio: 1.62). The daily infant dosage was 0.005 mg/kg/day (RID: 0.63 %) or 0.01 mg/kg/day (RID: 0.72 %) based on the average steady-state concentration and maximum concentration in human milk, respectively..

7. List of Appendix and Supplementary Materials

Supplementary material 1 – ObsDataPK_OSP_reference_sertraline
Supplementary material 2 – ObsDataPK_OSP_lactation_sertraline
Supplementary material 3 – Sertraline.pksim5

8. References

1. DeVane, C.L.; Liston, H.L.; Markowitz, J.S. Clinical Pharmacokinetics of Sertraline. *Clin. Pharmacokinet.* **2002**, *41*, 1247–1266, doi:10.2165/00003088-200241150-00002.
2. Kristensen, J.H.; Ilett, K.F.; Dusci, L.J.; Hackett, L.P.; Yapp, P.; Wojnar-Horton, R.E.; Roberts, M.J.; Paech, M. Distribution and Excretion of Sertraline and N-Desmethylsertraline in Human Milk. *Br. J. Clin. Pharmacol.* **1998**, *45*, 453–457, doi:10.1046/j.1365-2125.1998.00705.x.
3. Kobayashi, K.; Ishizuka, T.; Shimada, N.; Yoshimura, Y.; Kamijima, K.; Chiba, K. Sertraline N-Demethylation Is Catalyzed by Multiple Isoforms of Human Cytochrome P-450 in Vitro. *Drug Metab. Dispos.* **1999**, *27*, 763–766.
4. Owens, M.J.; Morgan, W.N.; Plott, S.J.; Nemeroff, C.B. Neurotransmitter Receptor and Transporter Binding Profile of Antidepressants and Their Metabolites. *J. Pharmacol. Exp. Ther.* **1997**, *283*, 1305–1322.
5. Dallmann, A.; Solodenko, J.; Ince, I.; Eissing, T. Applied Concepts in PBPK Modeling: How to Extend an Open Systems Pharmacology Model to the Special Population of Pregnant Women. *CPT pharmacometrics Syst. Pharmacol.* **2018**, *7*, 419–431, doi:10.1002/PSP4.12300.
6. Dallmann, A.; Himstedt, A.; Solodenko, J.; Ince, I.; Hempel, G.; Eissing, T. Integration of Physiological Changes during the Postpartum Period into a PBPK Framework and Prediction of Amoxicillin Disposition before and Shortly after Delivery. *J. Pharmacokinet. Pharmacodyn.* **2020**, *47*, 341–359, doi:10.1007/s10928-020-09706-z.
7. Job, K.M.; Dallmann, A.; Parry, S.; Saade, G.; Haas, D.M.; Hughes, B.; Berens, P.; Chen, J.; Fu, C.; Humphrey, K.; et al. Development of a Generic Physiologically-Based Pharmacokinetic Model for Lactation and Prediction of Maternal and Infant Exposure to Ondansetron via Breast Milk. *Clin. Pharmacol. Ther.* **2022**, *111*, 1111–1120, doi:10.1002/cpt.2530.
8. Koshimichi, H.; Ito, K.; Hisaka, A.; Honma, M.; Suzuki, H. Analysis and Prediction of Drug Transfer into Human Milk Taking into Consideration Secretion and Reuptake Clearances across the Mammary Epithelia. *Drug Metab. Dispos.* **2011**, *39*, 2370–2380, doi:10.1124/dmd.111.040972.
9. Templeton, I.E.; Chen, Y.; Mao, J.; Lin, J.; Yu, H.; Peters, S.; Shebley, M.; Varma, M. V. Quantitative Prediction of Drug-Drug Interactions Involving Inhibitory Metabolites in Drug Development: How Can Physiologically Based Pharmacokinetic Modeling Help? *CPT Pharmacometrics Syst. Pharmacol.* **2016**, *5*, 505–515, doi:10.1002/psp4.12110.
10. Alhadab, A.A.; Brundage, R.C. Physiologically-Based Pharmacokinetic Model of Sertraline in Human to Predict Clinical Relevance of Concentrations at Target Tissues. *Clin. Pharmacol. Ther.* **2020**, *108*, 136–144, doi:10.1002/cpt.1824.
11. Templeton, I.E.; Chen, Y.; Mao, J.; Lin, J.; Yu, H.; Peters, S.; Shebley, M.; Varma, M. V Quantitative Prediction of Drug-Drug Interactions Involving Inhibitory Metabolites in Drug Development: How Can Physiologically Based Pharmacokinetic Modeling

Help? *CPT pharmacometrics Syst. Pharmacol.* **2016**, *5*, 505–515, doi:10.1002/psp4.12110.
12. Atkinson, U.C.; Begg, E.J.E.J.; Atkinson, H.C.; Begg, E.J.E.J. Prediction of Drug Distribution into Human Milk from Physicochemical Characteristics. *Clin. Pharmacokinet.* **1990**, *18*, 151–167, doi:10.2165/00003088-199018020-00005.
13. Sutton, S.C. Role of Physiological Intestinal Water in Oral Absorption. *AAPS J.* **2009**, *11*, 277–285, doi:10.1208/s12248-009-9087-2.
14. WARRINGTON, S.J. Clinical Implications of the Pharmacology of Sertraline. *Int. Clin. Psychopharmacol.* **1991**, *6*, 11–22, doi:10.1097/00004850-199112002-00004.
15. Yue, X.H.; Wang, Z.; Tian, D.D.; Zhang, J.W.; Zhu, K.; Ye, Q. Determination of Sertraline in Human Plasma by UPLC-MS/MS and Its Application to a Pharmacokinetic Study. *J. Chromatogr. Sci.* **2016**, *54*, 195–199, doi:10.1093/chromsci/bmv128.
16. Saletu, B.; Grünberger, J.; Linzmayer, L. On Central Effects of Serotonin Re-Uptake Inhibitors: Quantitative EEG and Psychometric Studies with Sertraline and Zimelidine. *J. Neural Transm.* **1986**, *67*, 241–266, doi:10.1007/BF01243351.
17. Allard, S.; Sainati, S.M.; Roth-Schechter, B.F. Coadministration of Short-Term Zolpidem with Sertraline in Healthy Women. *J. Clin. Pharmacol.* **1999**, *39*, 184–191.
18. Kang, H.-A.; Cho, H.-Y.; Lee, Y.-B. Bioequivalence of Traline Tablet to Zoloft ® Tablet (Sertraline HCI 50 Mg) . *J. Pharm. Investig.* **2011**, *41*, 317–322, doi:10.4333/kps.2011.41.5.317.
19. Koytchev, R.; Ozalp, Y.; Erenmemisoglu, A.; Van Der Meer, M.J.; Alpan, R.S. Serotonin Reuptake Inhibitors: Bioequivalence of Sertraline Capsules. *Arzneimittel-Forschung/Drug Res.* **2004**, *54*, 629–633, doi:10.1055/s-0031-1297061.
20. Nagy, C.F.; Kumar, D.; Perdomo, C.A.; Wason, S.; Cullen, E.I.; Pratt, R.D. Concurrent Administration of Donepezil HCl and Sertraline HCl in Healthy Volunteers: Assessment of Pharmacokinetic Changes and Safety Following Single and Multiple Oral Doses. *Br. J. Clin. Pharmacol.* **2004**, *58 Suppl 1*, 25–33, doi:10.1111/j.1365-2125.2004.01801.x.
21. Ronfeld, R.A.; Tremaine, L.M.; Wilner, K.D. Pharmacokinetics of Sertraline and Its N-Demethyl Metabolite in Elderly and Young Male and Female Volunteers. *Clin. Pharmacokinet.* **1997**, *32*, 22–30.
22. Ronfeld, R.A.; Wilner, K.D.; Baris, B.A. Sertraline. Chronopharmacokinetics and the Effect of Coadministration with Food. *Clin. Pharmacokinet.* **1997**, *32*, 50–55.
23. Wang, J.H.; Liu, Z.Q.; Wang, W.; Chen, X.P.; Shu, Y.; He, N.; Zhou, H.H. Pharmacokinetics of Sertraline in Relation to Genetic Polymorphism of CYP2C19. *Clin. Pharmacol. Ther.* **2001**, *70*, 42–47, doi:10.1067/mcp.2001.116513.
24. Zhang, M.; Gao, F.; Cui, X.; Zhang, Y.; Sun, Y.; Gu, J. Development and Validation of an Improved Method for the Quantitation of Sertraline in Human Plasma Using LC-MS-MS and Its Application to Bioequivalence Studies. *J. Chromatogr. Sci.* **2011**, *49*, 89–93, doi:10.1093/chrsci/49.2.89.
25. Dodd, S.; Stocky, A.; Buist, A.; Burrows, G.D.; Maguire, K.; Norman, T.R. Sertraline in Paired Blood Plasma and Breast-Milk Samples from Nursing Mothers. *Hum. Psychopharmacol.* **2000**, *15*, 161–264, doi:10.1002/1099-1077(200006)15:4<161::aid-hup167>3.0.co;2-2.
26. Kristensen; Ilett; Dusci; Hackett; Yapp; Wojnar-Horton; Roberts; Paech Distribution and Excretion of Sertraline and N-Desmethylsertraline in Human Milk. *Br. J. Clin. Pharmacol.* **1998**, *45*, 453–457, doi:10.1046/j.1365-2125.1998.00705.x.
27. Weissman, A.M.; Levy, B.T.; Hartz, A.J.; Bentler, S.; Donohue, M.; Ellingrod, V.L.; Wisner, K.L. Pooled Analysis of Antidepressant Levels in Lactating Mothers, Breast

Milk, and Nursing Infants. *Am. J. Psychiatry* **2004**, *161*, 1066–1078, doi:10.1176/appi.ajp.161.6.1066.
28. Müller, M.J.; Preuß, C.; Paul, T.; Streit, F.; Brandhorst, G.; Seeliger, S. Serotonergic Overstimulation in a Preterm Infant after Sertraline Intake via Breastmilk. *Breastfeed. Med.* **2013**, *8*, 327–329, doi:10.1089/bfm.2012.0084.
29. Oberlander, T.F.; Grunau, R.E.; Fitzgerald, C.; Papsdorf, M.; Rurak, D.; Riggs, W.; T.F., O.; R.E., G.; C., F.; M., P.; et al. Pain Reactivity in 2-Month-Old Infants after Prenatal and Postnatal Selective Serotonin Reuptake Inhibitor Medication Exposure. *Pediatrics* **2005**, *115*, 411–425, doi:10.1542/peds.2004-0420 LK - http://limo.libis.be/resolver?&sid=EMBASE&issn=00314005&id=doi:10.1542%2Fpeds.2004-0420&atitle=Pain+reactivity+in+2-month-old+infants+after+prenatal+and+postnatal+selective+serotonin+reuptake+inhibitor+medication+exposure&stitle=Pediatrics&title=Pediatrics&volume=115&issue=2&spage=411&epage=425&aulast=Oberlander&aufirst=Tim+F.&auinit=T.F.&aufull=Oberlander+T.F.&coden=PEDIA&isbn=&pages=411-425&date=2005&auinit1=T&auinitm=F.
30. Berle, J.Ø.; Steen, V.M.; Aamo, T.O.; Breilid, H.; Zahlsen, K.; Spigset, O.; J.O., B.; V.M., S.; T.O., A.; H., B.; et al. Breastfeeding during Maternal Antidepressant Treatment with Serotonin Reuptake Inhibitors: Infant Exposure, Clinical Symptoms, and Cytochrome P450 Genotypes. *J. Clin. Psychiatry* **2004**, *65*, 1228–1234, doi:10.4088/jcp.v65n0911.
31. Pogliani, L.; Baldelli, S.; Cattaneo, D.; Pileri, P.; Clementi, E.; Cetin, I.; Zuccotti, G. Selective Serotonin Reuptake Inhibitors' Passage into Human Milk of Lactating Women. *J. Matern. Neonatal Med.* **2019**, *32*, 3020–3025, doi:10.1080/14767058.2018.1455180.
32. Salazar, F.R.; D'Avila, F.B.; de Oliveira, M.H.; Ferreira, P.L.; Bergold, A.M. Development and Validation of a Bioanalytical Method for Five Antidepressants in Human Milk by LC–MS. *J. Pharm. Biomed. Anal.* **2016**, *129*, 502–508, doi:10.1016/j.jpba.2016.07.047.
33. Schoretsanitis, G.; Augustin, M.; Saßmannshausen, H.; Franz, C.; Gründer, G.; Paulzen, M. Antidepressants in Breast Milk; Comparative Analysis of Excretion Ratios. *Arch. Womens. Ment. Health* **2019**, *22*, 383–390, doi:10.1007/s00737-018-0905-3.
34. Weisskopf, E.; Panchaud, A.; Nguyen, K.A.; Grosjean, D.; Hascoët, J.-M.; Csajka, C.; Eap, C.B.; Ansermot, N. Simultaneous Determination of Selective Serotonin Reuptake Inhibitors and Their Main Metabolites in Human Breast Milk by Liquid Chromatography-Electrospray Mass Spectrometry. *J. Chromatogr. B, Anal. Technol. Biomed. life Sci.* **2017**, *1057*, 101–109, doi:10.1016/j.jchromb.2017.04.039.
35. Wisner, K.L.; Perel, J.M.; Blumer, J. In Breast-Feeding Mother-Infant Pairs. **1998**, 690–692.
36. Kobayashi, K.; Ishizuka, T.; Shimada, N.; Yoshimura, Y.; Kamijima, K.; Chiba, K. Sertraline N-Demethylation Is Catalyzed by Multiple Isoforms of Human Cytochrome P-450 in Vitro. *Drug Metab. Dispos.* **1999**, *27*, 763–766.
