# Supplementary material for: Generic Workflow to Predict Medicine Concentrations in Human Milk Using Physiologically-Based Pharmacokinetic (PBPK) Modelling—A Contribution from the ConcePTION Project
Source: Pharmaceutics. 2023 May 11;15(5):1469. doi: 10.3390/pharmaceutics15051469 (PMC10223637; doi:10.3390/pharmaceutics15051469)
Supplement: Supplementary file 1 [file pharmaceutics-15-01469-s001.zip › File S8. Evaluation_report_tenofovir.pdf]

Building and evaluation of a Physiologically-Based PharmacoKinetic (PBPK) model for **tenofovir** in adults and lactating women

Disclaimer: The research project leading to these results was conducted as part of the ConcePTION consortium. This report only reflects the personal views of the stated authors. The results of this report are only intended for research purpose, and are not intended to be used in clinical practice.

Glossary

AUC	Area Under the Curve
C_{ave}	Average concentration
CL_{re}	Reuptake clearance (i.e. from milk to blood)
CL_{sec}	Secretion clearance (i.e. from blood to milk)
C_{max}	Maximum (~peak) concentration
DF	Disoproxil fumarate
f_u	Fraction unbound in plasma
GFR	Glomerular Filtration Rate
HBD	Hydrogen Bond Donors
HIV	Human immunodeficiency virus
IV	Intravenous (administration)
$LogD_{7.2}$	Logarithm of the partition coefficient between an octanol phase and an aqueous (buffer) phase at pH 7.2
$LogD_{7.4}$	Logarithm of the partition coefficient between an octanol phase and an aqueous (buffer) phase at pH 7.4
LogP	Logarithm of the partition coefficient between an octanol phase and (unbuffered) water as aqueous phase. This is the default parameter to express lipophilicity of a substance.
MD	Multiple dose
M/P ratio	Milk-to-Plasma ratio
MW	Molecular Weight (Da)
PAMPA	Parallel artificial membrane permeability assay
PBPK	Physiologically-Based PharmacoKinetic [*modeling*]
pKa	Logarithm of the acid dissociation constant
PO	Oral administration
PSA	Polar Surface Area
RID	Relative Infant Dose (%)
SD	Single dose

1. Table of Contents

1. Table of Contents *3*

2. Introduction *4*

3. Methods *5*

3.1 Modelling strategy **5**

3.1.1. Reference PBPK models 6

3.1.2. Lactation model 6

3.2 Data **6**

3.2.1 *In vitro* / physicochemical data 6

3.2.2 Clinical data 11

3.3 Model Parameters and assumptions **13**

3.3.1 Absorption 13

3.3.2 Distribution 14

3.3.3 Metabolism and excretion 14

3.3.4 Secretion to milk 14

3.3.5 Automated parameter optimization 15

3.4. Infant dosage calculation **15**

4. Results *16*

4.1 Final input parameters **16**

4.2 Diagnostic plots **18**

4.3 Concentration-time profiles **20**

4.3.1 Model building 21

4.3.2 Model verification 26

4.3.3 Lactation PBPK model 28

4.4 Estimated infant dosage **31**

5. Discussion *32*

6. Conclusion *32*

7. List of Appendix and Supplementary Materials *33*

8. References *33*

2. Introduction

Tenofovir (figure S1) is a nucleotide (nucleoside monophosphate) analogue with activity against retroviruses, including HIV-1, HIV-2 and hepdnaviruses [1]. The recommended dose is 300 mg a day (Drug label Viread). Tenofovir can be prescribed either as monotherapy or combination therapy (e.g. with emtricitabine). Tenofovir is orally administered as the prodrug tenofovir disoproxil fumarate (tenofovir DF). Following absorption, tenofovir DF is rapidly converted to tenofovir. The mean steady-state volume of distribution is approximately 0.8 L/kg after IV administration of 1 mg/kg tenofovir to HIV-positive patients [2]. Tenofovir is metabolized intracellularly to its active anabolite tenofovir diphospate. Tenofovir is metabolized and excreted unchanged in the urine. Tenofovir clearance exceeds the GFR rate, indicating urinary excretion through a combination of active tubular secretion and filtration [2].

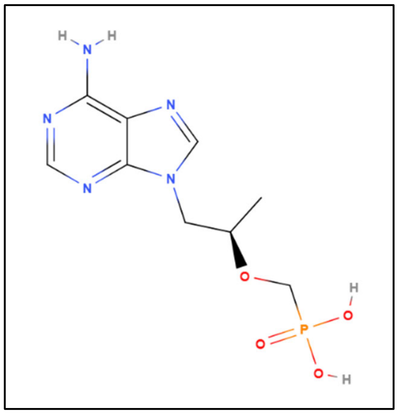

Figure S1. Chemical structure of tenofovir

The scope of this report is to:

(a) specify the details and underlying assumptions associated with the building of physiologically-based pharmacokinetic (PBPK) models for tenofovir in adult healthy volunteers or patients, and in postpartum women during lactation.

(b) evaluate the predictive performance of these PBPK models. This is achieved by comparing model-predicted plasma or milk concentrations with corresponding clinical observations.

3. Methods

The software used for the development of PBPK models presented in this report is tabulated below:

Software	Version
PK-Sim®	v9.1
MoBi®	v9.1

3.1 Modelling strategy

In the present report, a reference PBPK model was first established for adults (patients as well as healthy volunteers), and subsequently verified against clinical pharmacokinetic data reported for tenofovir in the scientific literature.

Relevant information on the anthropometry (height, weight) was gathered from the respective clinical studies, if reported. Information on physiological parameters (e.g. blood flows, organ volumes, hematocrit) in adults is available in the PK-Sim® database.

In a second step, a lactation PBPK model was developed, based on the general workflow described by Dallmann *et al. 2018* [3–5].

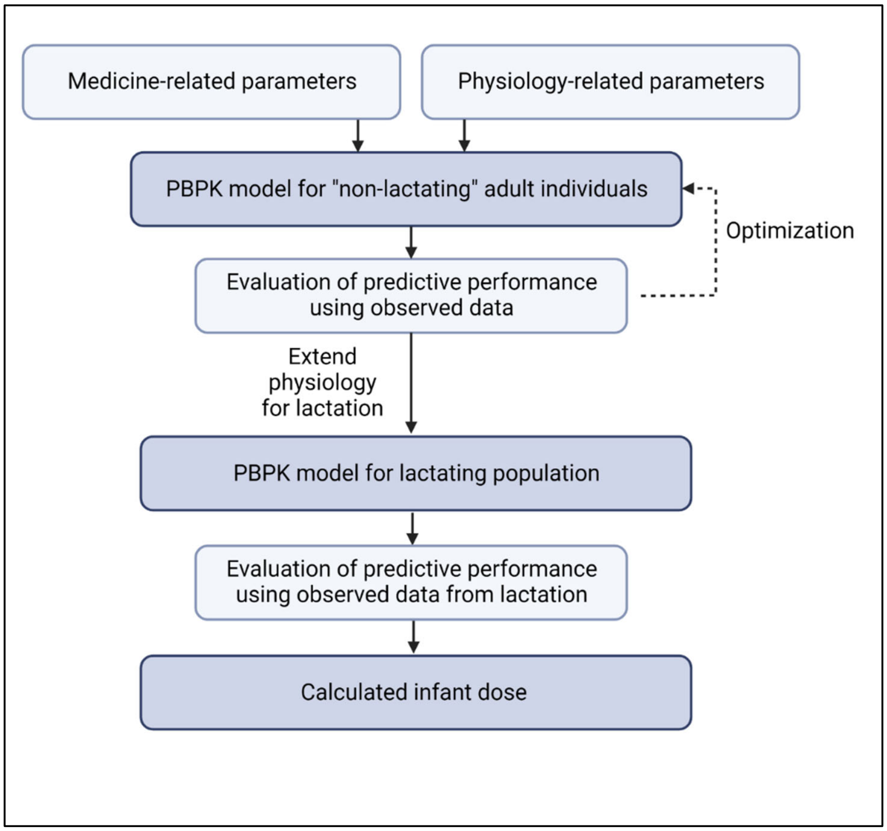

Figure S2 General workflow that was used in the present project to develop and evaluate the lactation PBPK model

Details about input data (physicochemical, *in vitro* and clinical data) can be found in section 3.2. Details about the structural models and their parameters can be found in section 3.3.

3.1.1. Reference PBPK models

The reference PBPK models were built based on studies with adult volunteers and/or adult patients, using the reported mean values for age, weight, height, and genetic background as described in each study protocol. When no information on these parameters could be found, a healthy male European individual, 30 years of age, with a body weight of 73 kg and a height of 176 cm was used.

The abundance (including population variability) of plasma proteins and enzymes/transporters that are integrated into PK-Sim are described in the publicly available 'PK-Sim Ontogeny Database Version 7.3' (PK-Sim Ontogeny Database Version 7.3).

Esterases are responsible for the conversion of tenofovir DF to tenofovir. In the PBPK model, expression of the esterases was manually added in plasma and the GI tract. Hepatic metabolism, glomerular filtration and active first-order tubular secretion of tenofovir were enabled, as they are involved in tenofovir excretion. It is recommended to take tenofovir in fed condition, therefore the PBPK model was first developed for fed condition.

Structural model selection was mainly guided by biological plausibility and by visual inspection of the predicted concentration time profiles. Uninformed parameter values (see below) were estimated using the parameter identification module of PK-Sim®.

The predictive performance of the models was evaluated by simulating:
- Single intravenous dose studies
- Single and multiple oral dose studies
- Fed and fasted state
- Males and females

For some parameters, parameter optimization was performed as described below to obtain improved concordance between predicted profiles and observed data.

3.1.2. Lactation model

After development of the reference model, the model was exported to MoBi® and a lactation PBPK model was constructed. To model the passage of tenofovir into human milk, i.e. across the blood/milk biological barrier, both the secretion (CL_{sec}) and reuptake clearance (CL_{re}) values were obtained using the empirical model developed by Koshimichi *et al. 2011* [6].

3.2 Data

3.2.1 *In vitro* / physicochemical data

A literature search was performed to collect available information on physicochemical properties of tenofovir and tenofovir disoproxil fumarate. The obtained information from literature is summarized in Table S1 and Table S3.

Table S2 shows the parameters that were additionally used for the lactation PBPK model.

Tenofovir

Table S1 Physicochemical parameters used as input for the tenofovir PBPK models

**Parameter**	**Value**	**Unit**	**Description**	**Source**
MW	287.21	g/mol	Molecular weight	Drugbank
pK_a	1.35 (acid)<br>6.70 (acid)<br>3.80 (base)	-	Logarithm of the acid dissociation constant	Drugbank<br>MarvinSketch
Solubility (pH 7)	1.87	mg/mL	Aqueous solubility	Human Metabolome database
LogP	-1.60	-	Log_{10} of the partition coefficient between octanol and water (~lipophilicity)	Drugbank
f_u	0.993	-	Fraction unbound in human plasma	Drugbank
**Liver plasma clearance** – Liver Plasma Clearance	70.00	mL/h/kg	Rate constant describing intrinsic hepatic plasma clearance (= $C_{lint, hep}$ normalized for liver volume)	Drugbank
GFR fraction	1.00	-	Fraction of the glomerular filtration rate used for passive renal elimination	
**Tubular secretion** – "TSspec"	91.00	min^{-1}	Active tubular secretion	Drugbank

Table S2 Physicochemical parameters used as input for the lactation PBPK model of tenofovir

Parameter	Value	Unit	Description	Source
Milk logP[a]	-1.60	-	Log_{10} of the partition coefficient between octanol and water	Drugbank
	-1.30			MarvinSketch
$LogD_{7.2}$	-3.65		Log_{10} of the partition coefficient between octanol and water at pH 7.2	MarvinSketch
$LogD_{7.4}$	-3.59		Log_{10} of the partition coefficient between octanol and water at pH 7.4	MarvinSketch
HBD	3.00	-	Hydrogen bond donors	Pubchem
PSA	136.38	Å^2	Polar surface area	Pubchem

[a] Milk logP is Log_{10} of the partition coefficient between octanol and water and is used as input for the calculations in the postpartum model (see equations below). In theory, this value is identical to the logP specified in Table S1. However, in some PBPK models, logP (Table S1) might be optimized using parameter identication. Therefore, it was chosen to use a separate parameter (i.e. Milk logP) to represent the logP used as input for the equations in the postpartum model.

<u>Tenofovir disoproxil fumarate</u>

Table S3 Physicochemical parameters used as input for the tenofovir disoproxil fumarate PBPK models

Parameter	Value	Unit	Description	Source
MW	635.52	g/mol	Molecular weight	Travuda label
pK_a	3.75 (base)	-	Logarithm of the acid dissociation constant	Travuda label
Solubility (pH 7)	13.40	mg/mL	Aqueous solubility	Travuda label
LogP	1.25	-	Log_{10} of the partition coefficient between octanol and	Travuda label

			water (~lipophilicity)	
f_u	0.993	-	Fraction unbound in human plasma	Drugbank
Esterase in vitro metabolic rate in the presence of liver microsomes – Michaelis Menten:<br>Km<br>Vmax	 <br>0.02<br>46.80	 <br>mmol/L<br>nmol/min/mg mic. protein		[7]

The default equations for free fraction in human milk and logD that were implemented in the spatial structure building block that was developed for the postpartum women are described below. Alternatively, these values can be overwritten by values calculated elsewhere (e.g. MarvinSketch) or determined *in vitro.*

The free fraction in human milk was calculated with the equations proposed by Atkinson and Begg [8], as follows:

$$f_{u\_skimmed\ milk} = \frac{f_u \times 0.448}{(0.000694^{0.448} + f_u^{0.448})}$$

$$P_{milk} = 10^{(-0.88+1.29\times \text{logD7.2})}$$

$$\text{Total free fraction in milk} = \frac{1}{(\frac{0.955}{f_{u\_skimmed\ milk}} + 0.045 \times P_{milk})}$$

Where: f_u *skimmed milk:* binding to proteins in milk; P_{milk}: partitioning between aqueous and lipid phase of milk; Total free fraction in milk: 'total' free fraction, i.e. accounting for both protein and lipid binding processes.

LogD values taking into account up to three pka values (as provided in the compound building block), were calculated as follows:

$$LogD = \text{Log}\,P + Log_{10}(logD_{factor})$$

With Milk logP (Table S2) as input for logP

$$LogD_{factor} = \text{K}_1 + (\text{K}_2 + \text{K}_3 + \text{K}_4) \times \text{base}^1 + \text{K}_5 \times \text{base}^{\max(\text{CT}_0+\text{CT}_1;-\text{CT}_0-\text{CT}_1)} + \text{K}_6 \times \text{base}^{\max(CT_0+CT_2;-CT_0-CT_2)} + \text{K}_7 \times \text{base}^{\max(\text{CT}_2+\text{CT}_1;-\text{CT}_2-\text{CT}_1)} + \text{K}_8 \times \text{base}^{\max(\text{CT}_0+\text{CT}_1+\text{CT}_2;-\text{CT}_0-\text{CT}_1-\text{CT}_2)}$$

$$K_1 = F_1 \times F_2 \times F_3$$
$$K_2 = (1 - F_1) \times F_2 \times F_3$$

$K_3 = F_1 \times (1 - F_2) \times F_3$
$K_4 = F_1 \times F_2 \times (1 - F_3)$
$K_5 = (1 - F_1) \times (1 - F_2) \times F_3$
$K_6 = (1 - F_1) \times F_2 \times (1 - F_3)$
$K_7 = (1 - F_1) \times F_2 \times (1 - F_3)$
$K_8 = (1 - F_1) \times (1 - F_2) \times (1 - F_3)$

F1 = CT0 != CT_NEUTRAL ? 1/(1+10^(CT0*(pKa_0- pH))) : 1
F2 = CT1 != CT_NEUTRAL ? 1/(1+10^(CT1*(pKa_1- pH))) : 1
F3 = CT2 != CT_NEUTRAL ? 1/(1+10^(CT2*(pKa_2- pH))) : 1

With CT = compound type (-1: acid; +1: base; 0: neutral), and pH = 7.2 or 7.4 respectively for $logD_{7.2}$ *and* $logD_{7.4}$

The transports that were added in the passive transport building block for 'transfer to milk' and 'transfer from milk' are based on secretion and reuptake and clearance values, Cl_{sec} and Cl_{re}, which were calculated according to the empirical equations proposed by Koshimich et al. 2011 [6], as follows:

$$\text{Log}\, CL_{re} = 2.793 + 0.179 \times LogP - 0.132 \times HBD$$

$$\text{Log}\, CL_{sec} = 3.367 \times Log10(MW) - 0.164 \times (LogP - LogD) - 0.015 \times PSA - 3.912$$

3.2.2 Clinical data

Literature searches were performed to collect available data on tenofovir in adults and postpartum women. The tenofovir reference PBPK model was developed using two different clinical studies with pharmacokinetic (PK) blood sampling. First, a study was used where tenofovir was administered intravenous to develop the PBPK model for tenofovir [2]. Next, oral administration of the prodrug was considered using a study with different oral doses of tenofovir disoproxil fumarate in fed condition [9]. Finally, a study with oral administration of 300 mg in fed and fasted condition was used to develop the PBPK model for fasted condition [10]. Five clinical trials with administration of tenofovir disoproxil fumarate in either fed or fasted condition were used for verification of the predictive performance of the PBPK model [11–14].

The evaluation of the predictive performance of the tenofovir lactation PBPK model was performed using 5 different studies were tenofovir was administered as an oral dose of 300 mg per day in the form of tenofovir disoproxil fumarate to lactating women [15–19]. The women were between 1 and 6 months postpartum. The samples were assumed to be trough samples when the exact timing was not reported in the articles.

Detailed information and data from the studies used for model building, verification, and lactation model can be found in Supplementary material 1 and 2.

3.2.2.1 Model building

The studies that were used for model building are shown in Table S4 (**training data**).

Table S4 Summary of studies used for PBPK model building of tenofovir in reference populations

Study ID	Reference	Arm/treatment/information used for model building
Deeks 1998	[2]	8 subjects received 1 mg/kg IV (single dose)
Deeks 1998	[2]	8 subjects received 3 mg/kg IV (single dose)
Deeks 1998	[2]	8 subjects received 1 mg/kg IV (multiple dose)
Deeks 1998	[2]	8 subjects received 3 mg/kg IV (multiple dose)
Barditch-Crovo 2001	[9]	10 subjects received 600 mg PO in fed condition (multiple dose)
Barditch-Crovo 2001	[9]	7 subjects received 300 mg PO in fed condition (multiple dose)
Barditch-Crovo 2001	[9]	5 subjects received 150 mg PO in fed condition (multiple dose)
Barditch-Crovo 2001	[9]	12 subjects received 75 mg PO in fed condition (multiple dose)
Gebroers 2015	[10]	5 subjects received 300 mg PO in fed condition (single dose)
Gebroers 2015	[10]	5 subjects received 300 mg PO in fasted condition (single dose)

Table S5 Demographic information

Study ID	Reference	Number of subjects (female ratio)	Age (year)	Weight (kg)
Deeks 1998	[2]	8 (0)<br>8 (0)<br>8 (0)<br>8 (0)	39<br>39<br>38<br>38	81.1 ± 22.0<br>81.7 ± 21.8<br>71.7 ± 11.8<br>70.5 ± 10.1
Barditch-Crovo 2001	[9]	10 (0)<br>7 (0.14)<br>5 (0.60)<br>12 (0)	42.5 (37-44)<br>41 (33-40.5)<br>38 (35-42)<br>38.5 (32.5-44)	-<br>-<br>-<br>-
Gebroers 2015	[10]	5 (0.60)	- (22-26)	-

3.2.2.2 Model verification

The studies that were used to evaluate the predictive performance of the PBPK model are shown in Table S6 (**verification data**).

Table S6 Summary of studies used for model verification of tenofovir PBPK model in reference population

Study ID	Reference	Arm/treatment/information used for model verification
Mathias 2007	[11]	48 subjects received 300 mg PO in fasted condition (single dose)
Kearney 2005	[12]	24 subjects received 300 mg PO in fed condition (multiple dose)
Hendrix 2013	[20]	168 subjects received 300 mg PO in fed condition (multiple dose)

Hu 2013	[13]	14 subjects received 300 mg PO in fasted condition (single dose)
Hu 2013	[13]	14 subjects received 300 mg PO in fasted condition (multiple dose)
Dickinson 2015	[14]	18 subjects received 245 mg PO in fed condition (multiple dose)

Table S7 Demographic information

**Study ID**	**Reference**	**Number of subjects (female ratio)**	**Age (year)**	**Weight (kg)**
Mathias 2007	[11]	48 (0.73)	30 ± 7.1	65.2 ± 7.15
Kearney 2005	[12]	24 (-)	-	-
Hendrix 2013	[20]	168 (1)	29 (27-37)	73 (65-88)
Hu 2013	[13]	14 (0.50)	24.6 (19-37)	58.7 (46.7-69.6)
Dickinson 2015	[14]	18 (0.61)	31 (19-47)	75 (60-105)

3.2.2.3 Lactation PBPK model

Table S8 shows the study that was used for the lactation PBPK model.

Table S8 Summary of study used for PBPK model development of tenofovir in lactating women

**Study ID**	**Publication**	**Arm/treatment/information used for model building and verification**
Erturk 2018	[18]	11 women (2-6 months postpartum) received PO 300 mg/day (multiple dose)
Mugwanya 2016	[17]	50 women (9-19 weeks postpartum) received PO 300 mg/day (multiple dose)
Palombi 2016	[19]	47 or 33 women (1 or 12 months postpartum) received PO 300 mg/day (multiple dose)
Waitt 2017	[15]	6 women (83-146 days postpartum) received PO 300 mg/day (multiple dose)
Waitt 2018	[16]	27 and 18 women received PO 300 mg/day (multiple dose)

3.3 Model Parameters and assumptions

3.3.1 Absorption

Tenofovir is administered orally as a prodrug, tenofovir disoproxil fumarate. It has been described in literature that esterases are responsible for the conversion. We assumed that the conversion takes place in plasma, the GI lumen (stomach, duodenum, lower jejunum, upper jejunum, lower ileum and upper ileum) and the GI mucosa (duodenum, lower jejunum, upper jejunum, lower ileum and upper ileum). Relative expression was manually adjusted to fit the observed data in fed condition [9]. It was assumed that the conversion of the prodrug to tenofovir in the GI tract was bigger in the fasted condition. Therefore, relative expression in the GI tract was multiplied with a factor 2 for fasted condition.

Release from the tablet was implemented as the dissolution profile from a VIREAD tablet.

Tenofovir specific intestinal permeability was calculated using lipophilicity, according to PK Sim standard methods. For tenofovir disoproxil fumarate, a PAMPA value was found in literature (1.30E-5 cm/s) [21]. For the simulation during lactation, we assumed fed state.

3.3.2 Distribution

An important parameter influencing the distribution of a compound is lipophilicity. Lipophilicity was taken from literature for tenofovir disoproxil fumarate and tenofovir. The tissue partition coefficients (Kp) calculation was according to 'PK-Sim Standard' and the cellular permeability calculation was 'PK-Sim Standard'.

3.3.3 Metabolism and excretion

The conversion of tenofovir disoproxil fumarate to tenofovir was implemented using *in vitro* Michaelis-Menten kinetics [7]. Tenofovir was metabolized via hepatic metabolism. Renal elimination of tenofovir was implemented as glomerular filtration and active tubular secretion.

3.3.4 Secretion to milk

To model the transfer process of tenofovir into human milk, both the secretion (CL_{sec}) and reuptake clearance (CL_{re}) were calculated using the empirical equations developed by Koshimichi *et al.* 2011 (see **Error! Reference source not found.**) [4].

First, in MoBi®, a spatial structure for the postpartum women was constructed, similar to the workflow from Dallmann *et al.* 2018 [2]. Here, breasts were added as a compartment. In addition, the human milk was connected to the plasma subcompartment of the breasts. The human milk volume was specified as 0.5 L to represent the structure of Koshimichi *et al.* 2011, and a geometric standard deviation of 1.16 was assumed in the population. The free fraction in human milk, and logD values were implemented as the equations described previously. The transfer between plasma and milk was defined as two kinetic processes (transfer to milk and transfer from milk) under passive transports (see below). Next, the simulation was combined with the postpartum population from Job *et al.* 2021 in PK-Sim to account for the postpartum physiology [3].

<u>*Kinetics*</u>

Transfer to milk

$$\frac{dNmilk}{dt} = Cplasma \times f_u \times CL_{sec}$$

where Cplasma is the concentration in plasma (in breast compartment), f_u is the free fraction in plasma and CL_{sec} is the secretion clearance.

Transfer from milk

$$\frac{dNplasma}{dt} = Cmilk \times f_u \times CL_{re}$$

where Cmilk is the concentration in human milk, f_u is the total free fraction in human milk (protein and lipid) and CL_{re} is the reuptake clearance.

The median simulated plasma and human milk concentration-time profiles can be used to calculate the M/P ratio as follows:

$$M/P\ ratio\ = \frac{AUC_{milk}}{AUC_{plasma}}$$

3.3.5 Automated parameter optimization

There were no parameters estimated via automated parameter identification.

Relative expression of the esterases were adjusted manually, to fit to the observed data in fed condition [9]. The expression in the GI tract was multiplied with factor 2 to described fasted condition [10].

**Location**	**Relative expression in fed condition**	**Relative expression in fasted condition**
Plasma	1.00	1.00
Lumen – Stomach	5.00E-4	1.00E-04
Lumen – Duodenum	0.30	0.60
Lumen – upper jejunum	0.30	0.60
Lumen – lower jejunum	0.30	0.60
Lumen – upper ileum	0.30	0.60
Lumen – lower ileum	0.30	0.60
Mucosa – Duodenum	0.35	0.70
Mucosa – upper jejunum	0.35	0.70
Mucosa – lower jejunum	0.35	0.70
Mucosa – upper ileum	0.35	0.70
Mucosa – lower ileum	0.35	0.70

3.4. Infant dosage calculation

Infant dosage via human milk was then calculated based on the predicted (average and maximal) steady-state tenofovir concentration in human milk, as well as the daily milk intake volume. The daily infant dosage was then compared to the maternal dosage, resulting in the relative infant dose (RID).

$$Daily\ infant\ dosage = C_{average} * 150\ \frac{mL}{kg\ .day}$$

$$Daily\ infant\ dosage = C_{max} * 150\ \frac{mL}{kg\ .day}$$

$$Relative\ infant\ dose\ (RID) = \frac{Infant\ dosage}{Maternal\ dosage} * 100\ \%$$

4. Results

Both the reference and postpartum PBPK model of tenofovir was developed and verified with clinical PK data.

The models were evaluated covering studies including in particular:

- Intravenous and oral administration
- Single and multiple doses
- A dose range from 75 up to 600 mg
- Fed and fasted conditions

The model describes the administration of tenofovir disoproxil fumarate, and its conversion to tenofovir. Tenofovir is metabolized via hepatic metabolism, and excreted unchanged in the urine via GFR and active tubular secretion. Moreover, secretion and reuptake to human milk were described by CL_{sec} and CL_{re}.

The next sections show:

- The final model parameters for the building blocks: section 4.1
- The overall predictive performance: section 4.2
- The simulated versus observed concentration-time profiles for the clinical studies used for model building and for model verification: section 4.3

4.1 Final input parameters

The compound values of the final postpartum PBPK model for tenofovir are illustrated below.

Tenofovir

Physicochemical parameters

Parameter	Value	Unit	Source
MW	287.21	g/mol	Drugbank
pKa	1.35 (acid)<br>6.70 (acid)<br>3.80 (base)	-	Drugbank<br>MarvinSketch
Solubility	1.87	mg/mL	Human Metabolome database
Lipophilicity	-1.60	-	Drugbank
f_u	0.993	-	Drugbank
Small molecule (Y/N)	Yes	-	

Calculation methods

Name	Value
Tissue partition coefficients	PK-Sim Standard
Cellular permeabilities	PK-Sim Standard

AMDE-related parameters

Parameter	Value	Unit	Source
Intestinal permeability	3.48E-9	cm/min	PK Sim default
**Hepatic clearance** – liver plasma clearance	70	mL/h/kg	Drugbank
GFR fraction	1	-	
Tubular secretion – first order	91.00	mL/min	Drugbank

<u>Tenofovir DF</u>

Physicochemical parameters

Parameter	Value	Unit	Source
MW	635.52	g/mol	Travuda Label
pKa	3.75 (base)	-	Travuda Label
Solubility	13.40	mg/mL	Travuda Label
Lipophilicity	1.25	-	Travuda Label
f_u	0.993	-	Asssumption tenofovir
Small molecule (Y/N)	Yes	-	
Plasma protein binding partner	Albumin		

Calculation methods

Name	Value
Tissue partition coefficients	PK-Sim Standard
Cellular permeabilities	PK-Sim Standard

AMDE-related parameters

Parameter	Value	Unit	Source
Intestinal permeability	1.30E-5	cm/s	[21]
Esterase in vitro metabolic rate in the presence of liver microsomes – Michaelis Menten:<br>Km<br>Vmax	 0.02<br>46.80	 mmol/L<br>nmol/min/mg mic. protein	[7]

Formulation-related parameters

Type: Tablet formulation

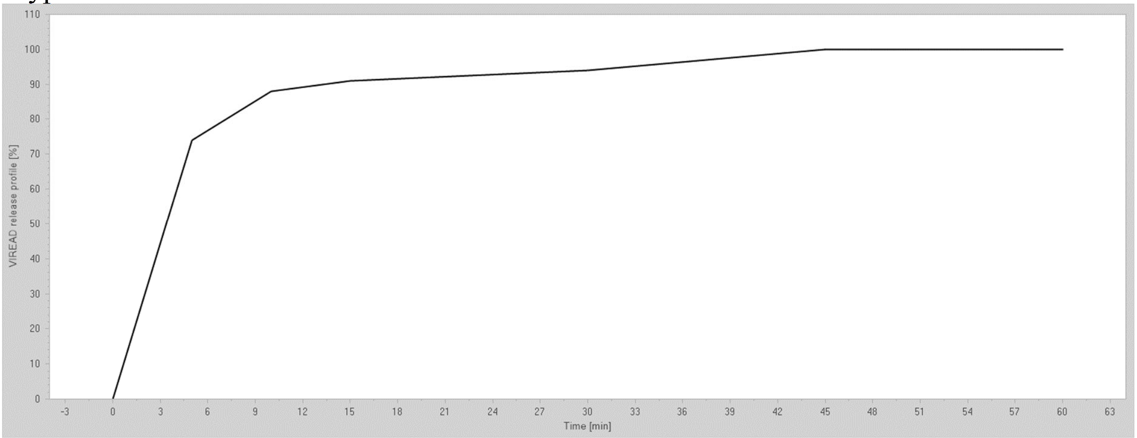

Figure S3. In vitro dissolution profile of tenofovir in tablet formulation (Viread release profile)

Physicochemical and physiological parameters relevant to the lactation model

Tenofovir

**Parameter**	**Value**	**Unit**	**Source**
Milk logP	-1.6	-	Drugbank
HBD	3.00	-	Pubchem
PSA	136.38	$Å^2$	Pubchem
CL_{sec}	8.66E-4	L/min	Default
CL_{re}	2.15E-3	L/min	Default
*fu*_skimmed milk[a]	0.96	-	Default
Pmilk[b]	3.78E-8	-	Default
Total free fraction in milk[c]	1.01	-	Default
$logD_{7.2}$	-5.07	Log units	Default
$logD_{7.4}$	-5.28	Log units	Default

[a] binding to proteins in milk; [b] partitioning between aqueous and lipid phase of milk; [c] total free fraction, accounting for both protein and lipid binding

4.2 Diagnostic plots

The geometric mean fold errors (GMFE) on AUC and Cmax were 1.26 and 1.21 for the model building dataset, and 1.26 and 1.20 for the model verification dataset.

The following plot shows the predictive performance graph for Cmax and AUC of tenofovir for the PBPK model performance of all data used.

Predicted over observed ratio values of all data listed in section 3.2.2 are presented below.

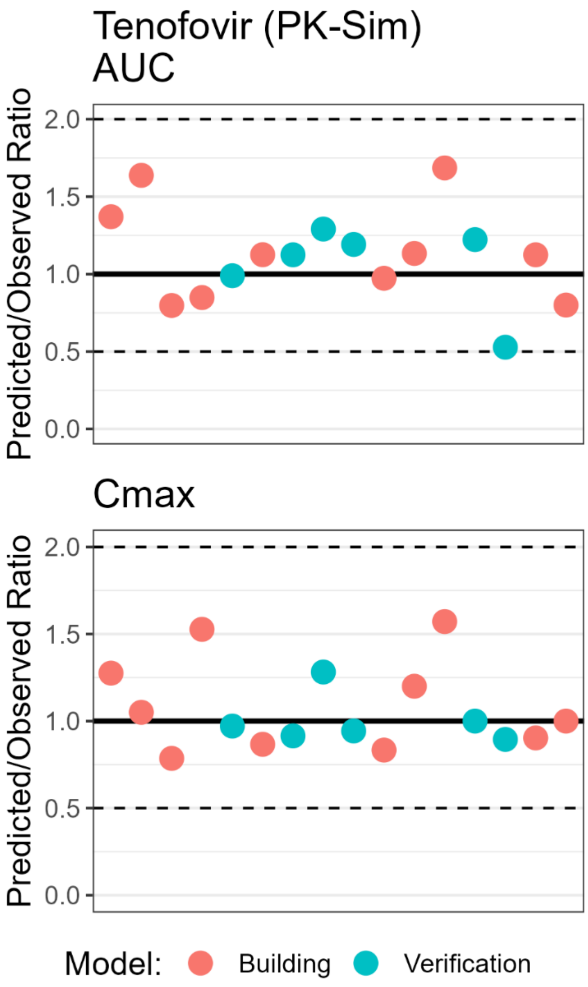

Figure S4. Predicted over observed ratio profile

Table S9 Ratio between the predicted and observed pharmacokinetic parameters of tenofovir in different dosing regimens for model building

Study ID/ Reference	Dose/ Route	AUC_{obs} (mg*h/L)	AUC_{pred} (mg*h/L)	Fold error	$Cmax_{obs}$ (mg/L)	$Cmax_{pred}$ (mg/L)	Fold error
Deeks 1998 [2]	1 mg/kg IV SD	3.81	6.24	1.64	2.98	3.13	1.05
Deeks 1998 [2]	3 mg/kg IV SD	15.26	17.17	1.13	10.2	8.84	0.87
Deeks 1998 [2]	1 mg/kg IV MD	4.86	6.66	1.37	2.47	3.15	1.28
Deeks 1998 [2]	2 mg/kg IV MD	20.48	17.38	0.85	5.77	8.81	1.53
Barditch-Crovo 2001 [9]	600 mg PO MD	5.78	6.50	1.12	0.62	0.56	0.90

Barditch-Crovo 2001 [9]	300 mg PO MD	2.97	2.89	0.97	0.30	0.25	0.83
Barditch-Crovo 2001 [9]	150 mg PO MD	1.58	1.26	0.80	0.14	0.11	0.79
Barditch-Crovo 2001 [9]	75 mg PO MD	0.45	0.55	1.22	0.05	0.05	1.00
Gebroers 2015 [10]	300 mg PO SD fed	0.70	1.18	1.69	0.14	0.22	1.57
Gebroers 2015 [10]	300 mg PO SD fasted	0.75	0.85	1.13	0.26	0.25	0.96

Table S10 Ratio between the predicted and observed pharmacokinetic parameters of tenofovir in different dosing regimens used for model verification

Study ID/ Reference	Dose/ Route	AUC_{obs} (mg*h/L)	AUC_{pred} (mg*h/L)	Fold error	$Cmax_{obs}$ (mg/L)	$Cmax_{pred}$ (mg/L)	Fold error
Mathias 2007 [11]	300 mg PO SD	2.69	1.42	0.53	0.94	0.85	0.90
Kearney 2005 [12]	300 mg PO MD	2.57	2.89	1.12	0.94	0.86	0.91
Hendrix 2013 [20]	300 mg PO MD	1.41	1.68	1.19	0.31	0.29	0.94
Hu 2013 [13]	300 mg PO SD	2.38	2.91	1.22	0.19	0.34	1.79
Hu 2013 [13]	300 mg PO MD	3.54	4.57	1.29	0.32	0.40	1.25
Dickinson 2015 [14]	245 mg PO MD	3.84	3.80	0.99	0.20	0.19	0.95

4.3 Concentration-time profiles

Simulated versus observed concentration-time profiles of all data listed in section 3.2.2 are presented below. The original.pksim5 are provided in Supplemented material 3.

4.3.1 Model building

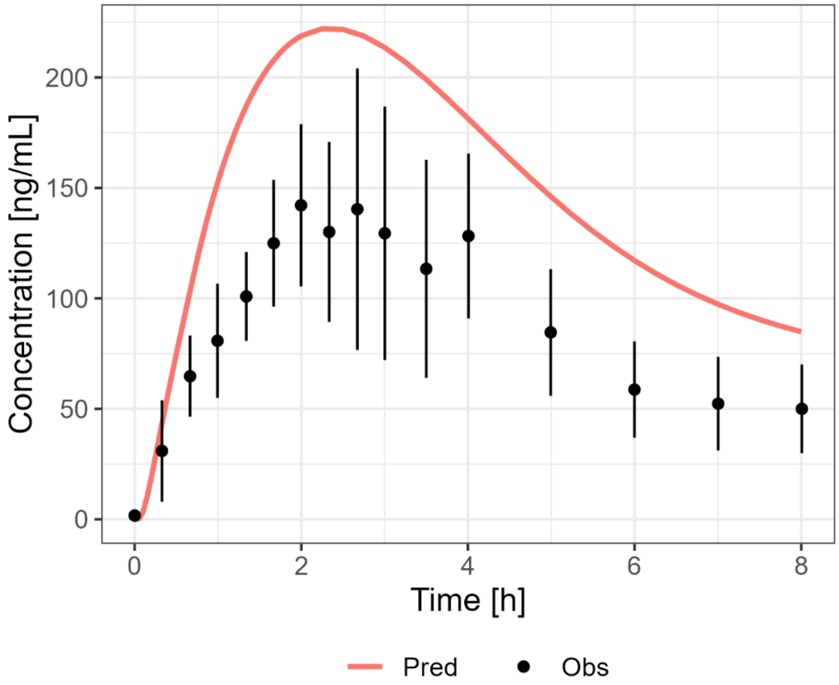

Figure S5 Predicted (Pred) versus observed (Obs) concentration-time profile after administration of 300 mg fed PO [10]

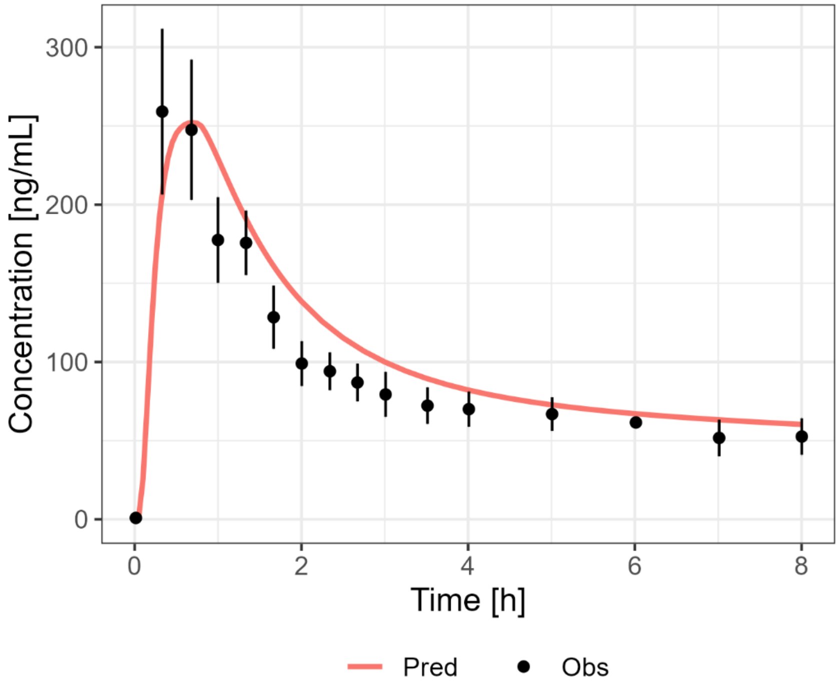

Figure S6 Predicted (Pred) versus observed (Obs) concentration-time profile after administration of 300 mg fasted PO [10]

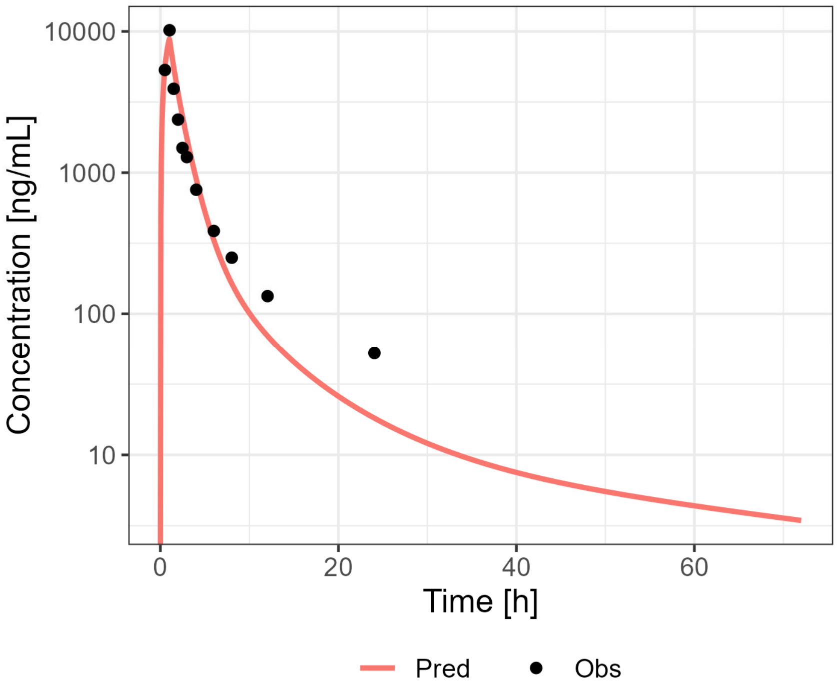

Figure S7 Predicted (Pred) versus observed (Obs) concentration-time profile after administration of 3 mg/kg IV SD [2]

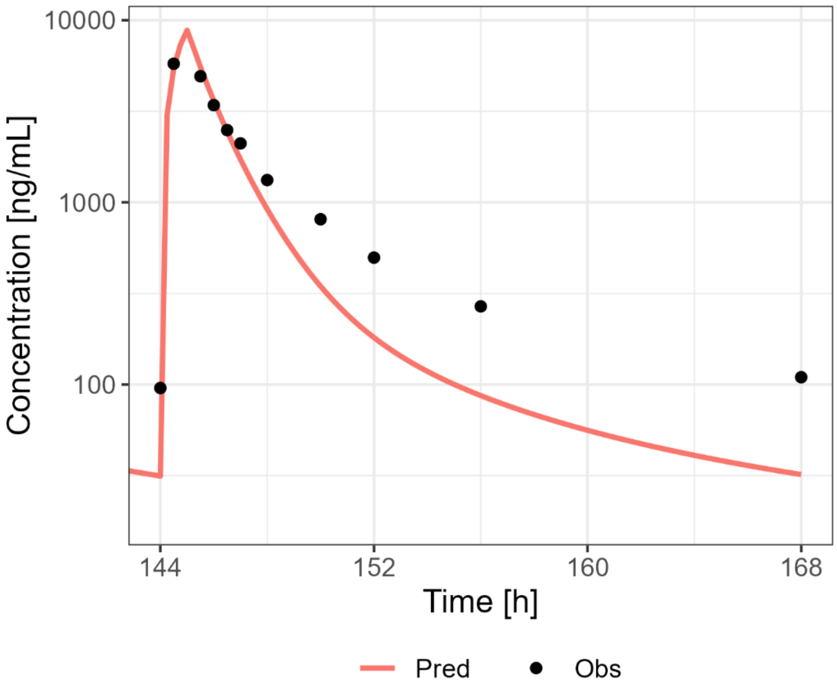

Figure S8 Predicted (Pred) versus observed (Obs) concentration-time profile after administration of 3 mg/kg IV MD [2]

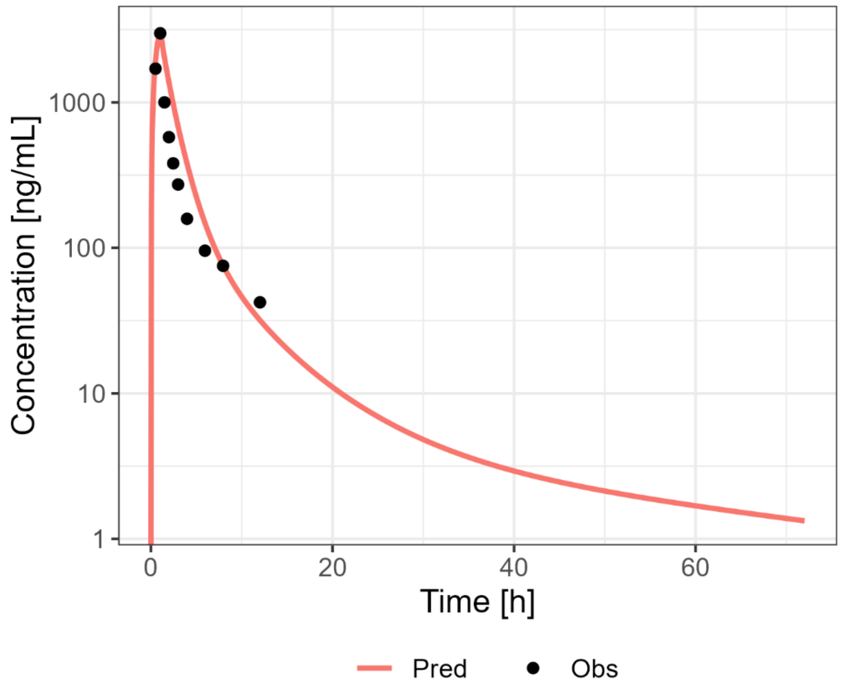

Figure S9 Predicted (Pred) versus observed (Obs) concentration-time profile after administration of 1 mg/kg IV SD [2]

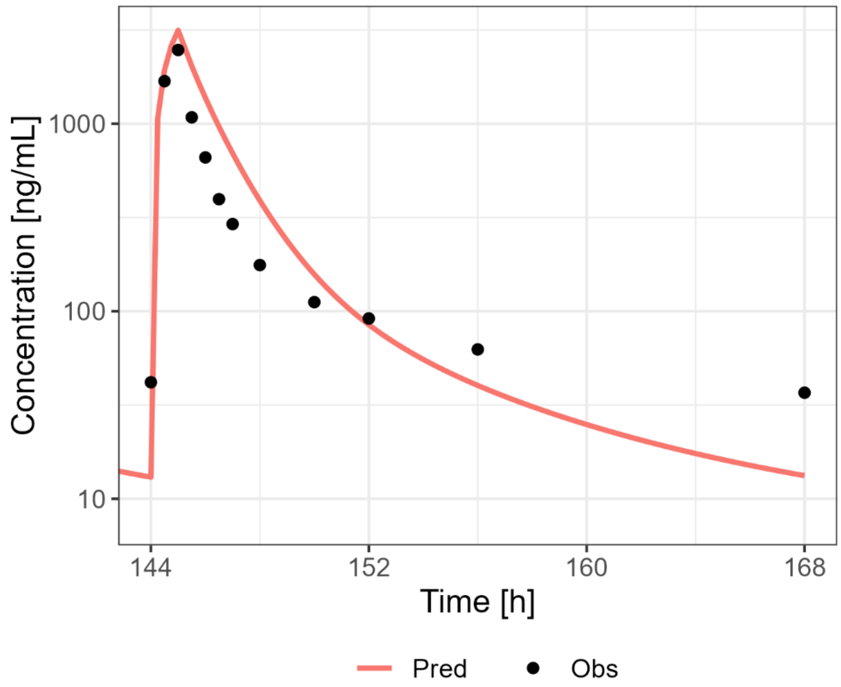

Figure S10 Predicted (Pred) versus observed (Obs) concentration-time profile after administration of 1 mg/kg IV MD [2]

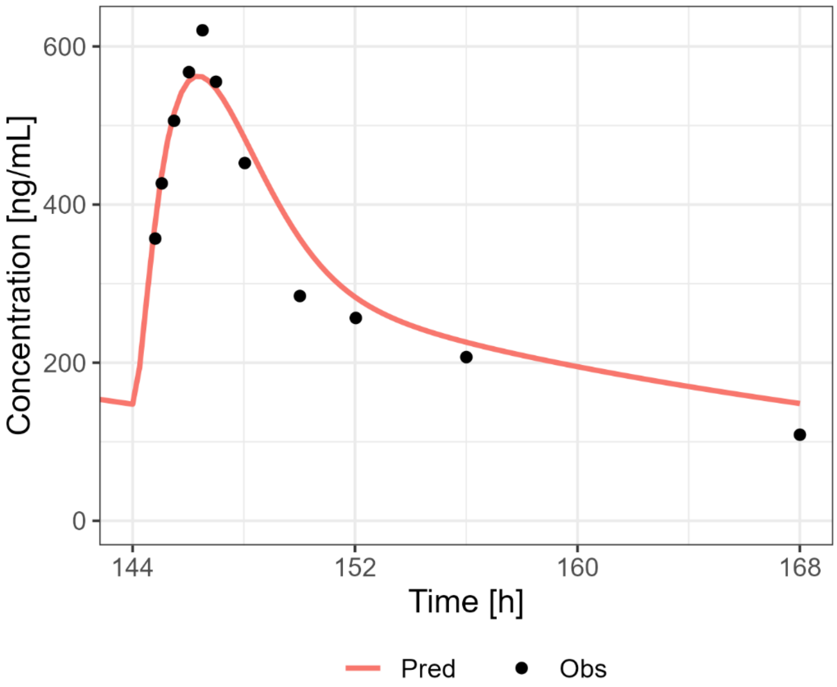

Figure S11 Predicted (Pred) versus observed (Obs) concentration-time profile after administration of 600 mg PO [9]

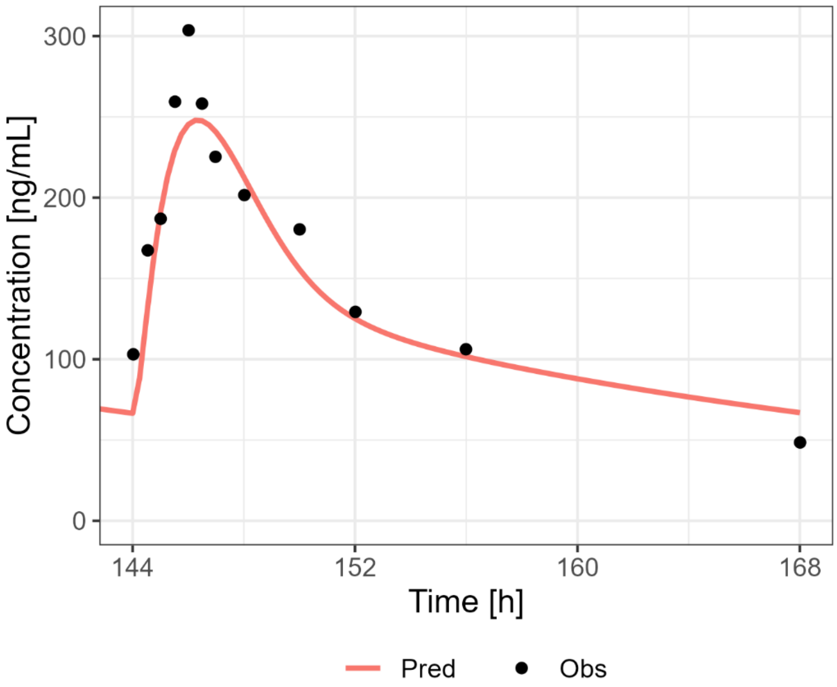

Figure S12 Predicted (Pred) versus observed (Obs) concentration-time profile after administration of 300 mg PO [9]

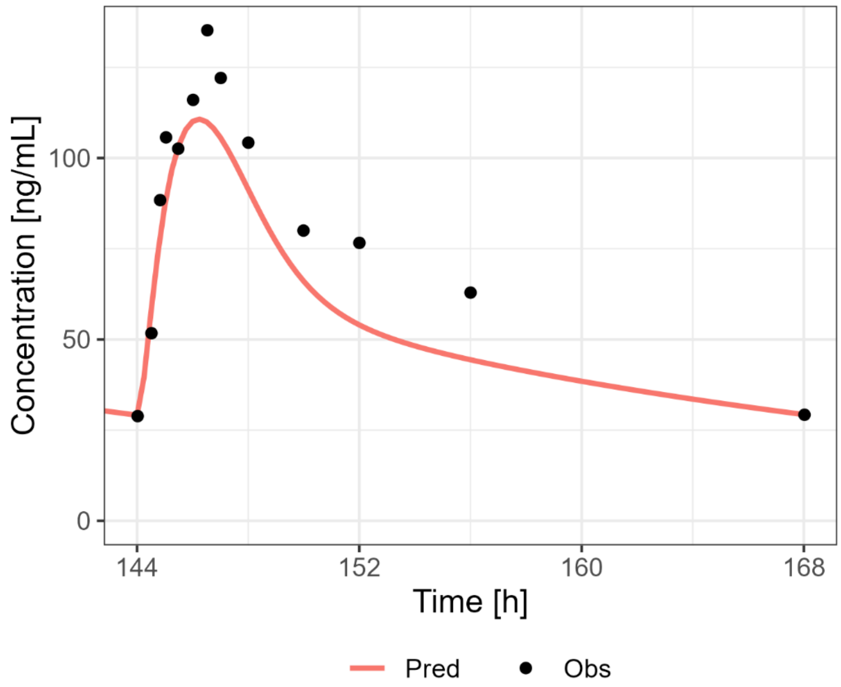

Figure S13 Predicted (Pred) versus observed (Obs) concentration-time profile after administration of 150 mg PO [9]

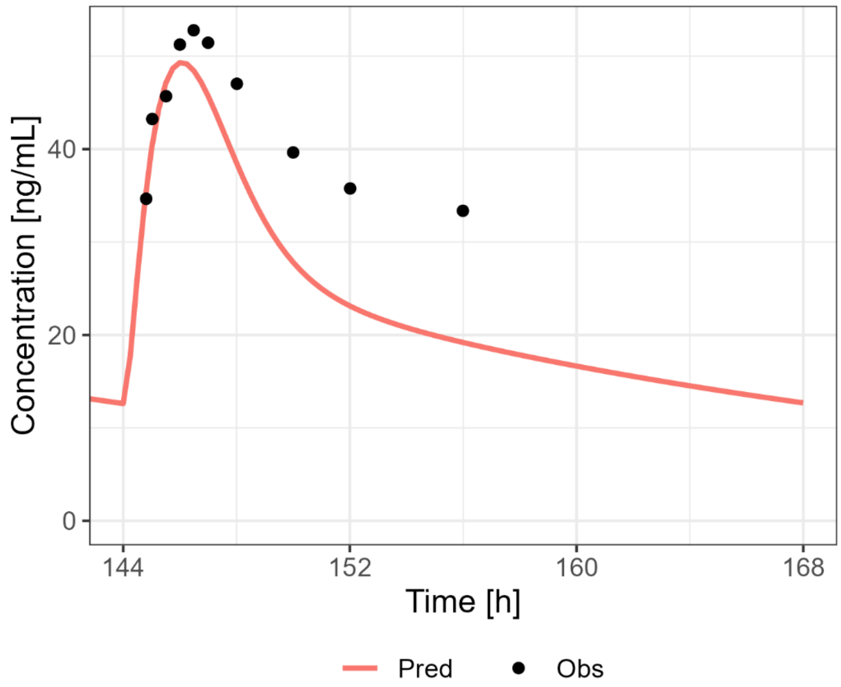

Figure S14 Predicted (Pred) versus observed (Obs) concentration-time profile after administration of 75 mg PO [9]

4.3.2 Model verification

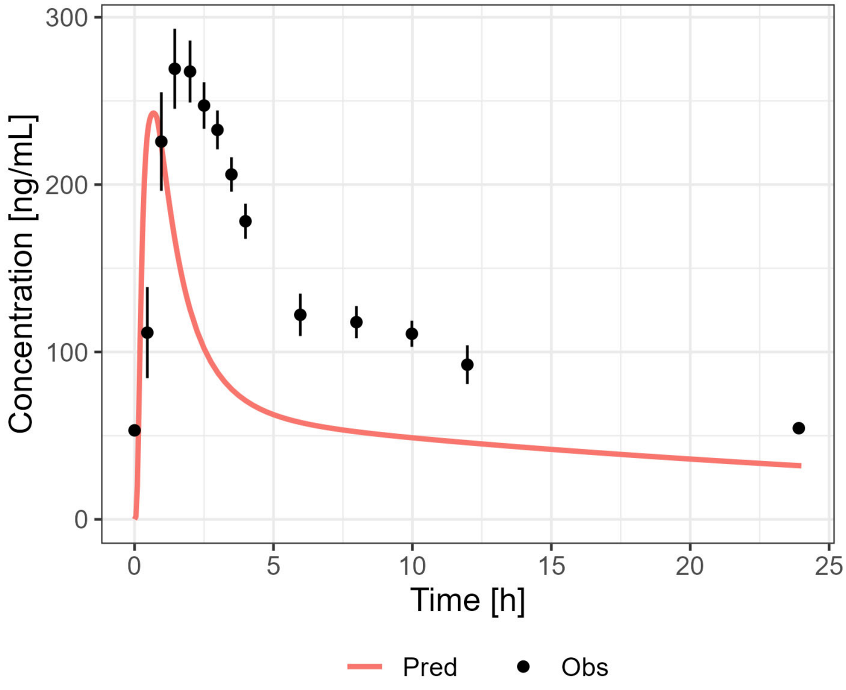

Figure S15 Predicted (Pred) versus observed (Obs) concentration-time profile after administration of 300 mg fasted PO [11]

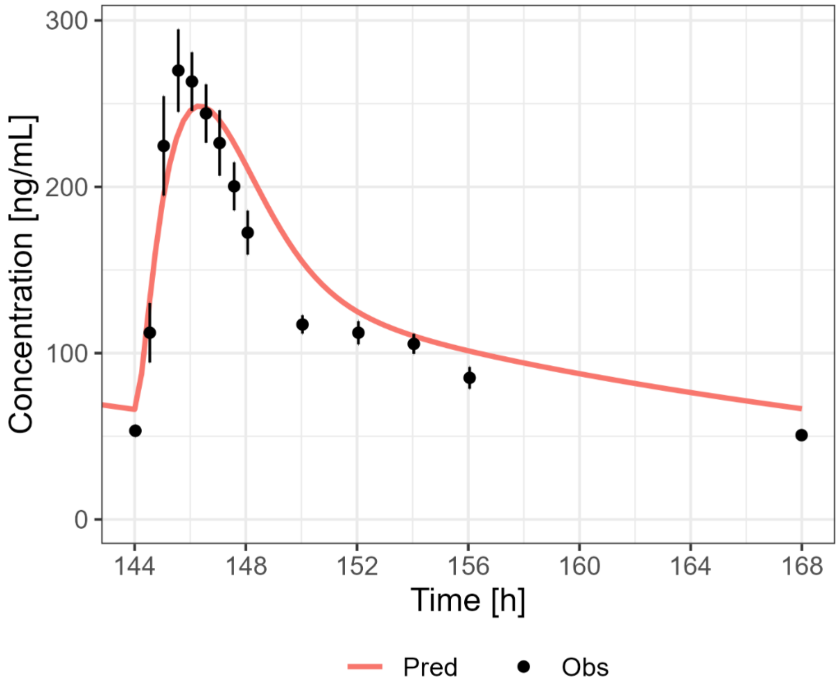

Figure S16 Predicted (Pred) versus observed (Obs) concentration-time profile after administration of 300 mg PO [12]

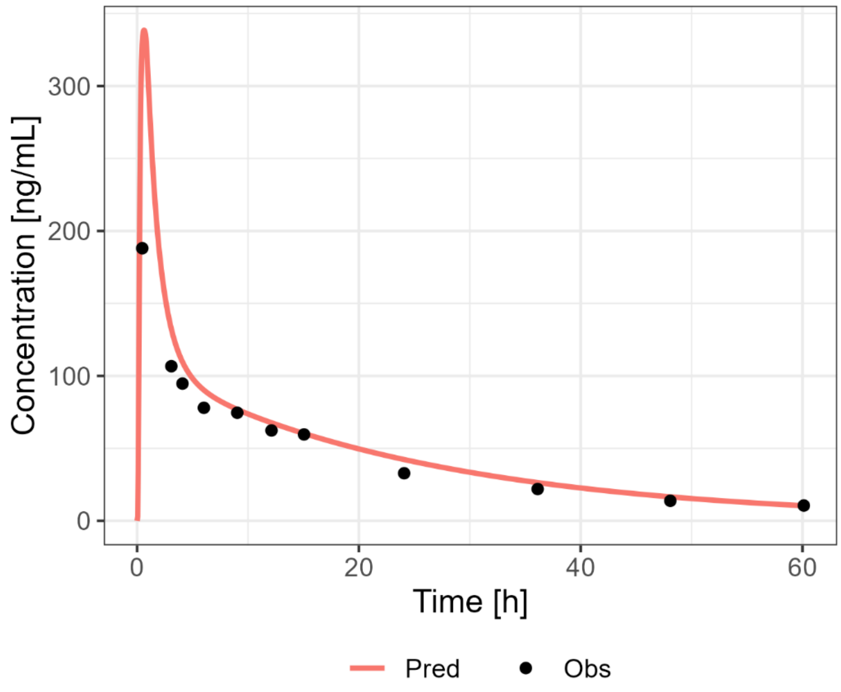

Figure S17 Predicted (Pred) versus observed (Obs) concentration-time profile after administration of 300 mg fasted PO [13]

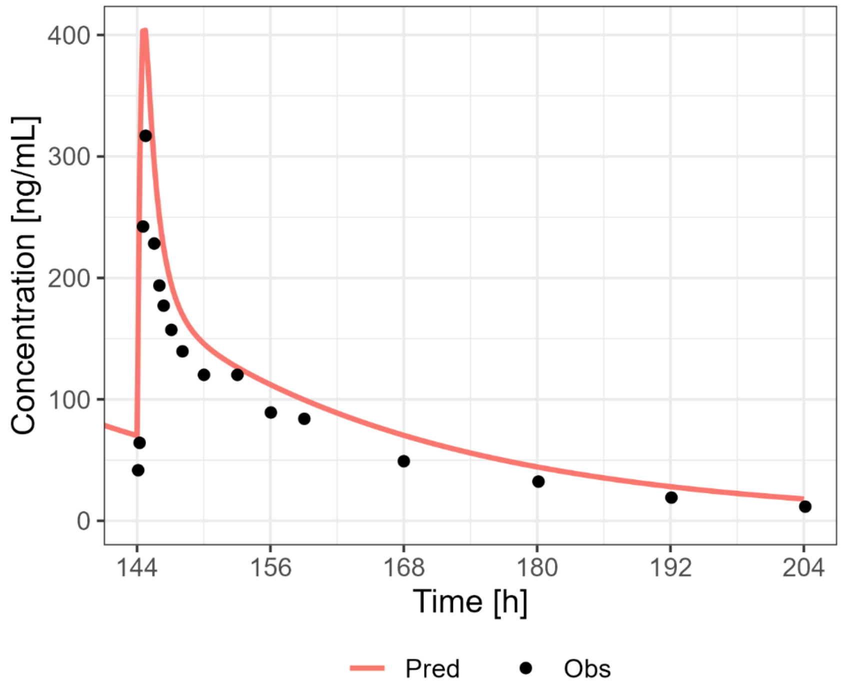

Figure S18 Predicted (Pred) versus observed (Obs) concentration-time profile after administration of 300 mg fasted PO MD [13]

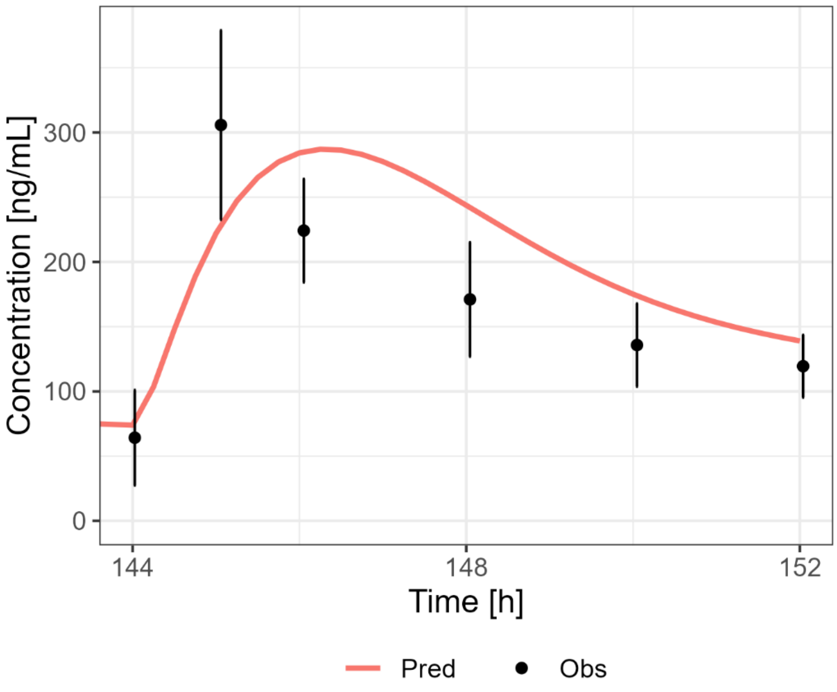

Figure S19 Predicted (Pred) versus observed (Obs) concentration-time profile after administration of 300 mg PO [20]

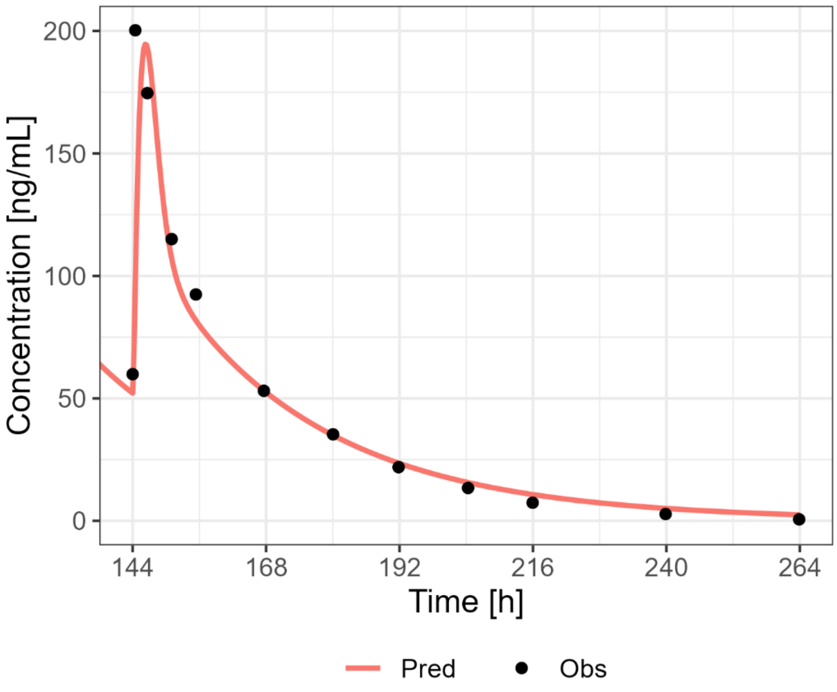

Figure S20 Predicted (Pred) versus observed (Obs) concentration-time profile after administration of 245 mg PO [14]

4.3.3 Lactation PBPK model

A sample size of 1000 individuals, three months postpartum, was used in each simulation of the virtual lactation population.

Model A: First, the transfer of tenofovir was predicted using the LogP value of -1.60, and the default values for logD based on the equations implemented in the spatial structure building block as described above.

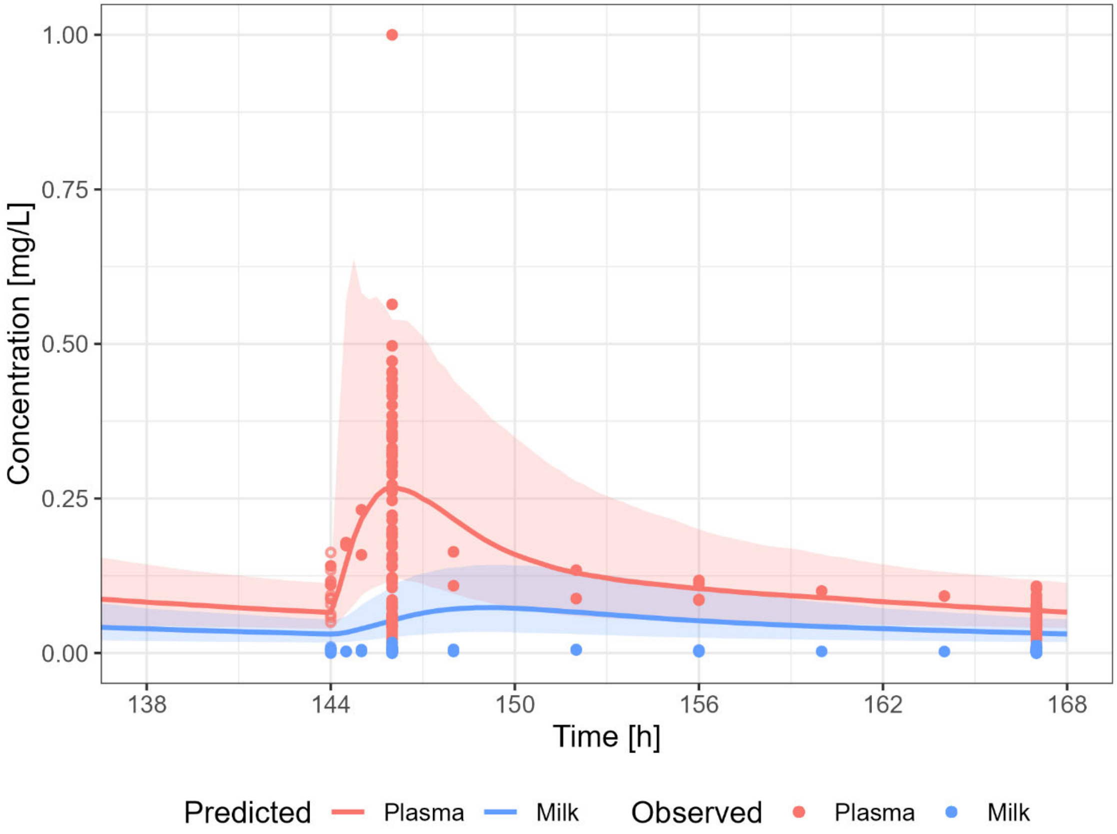

Figure S21 Predicted (Pred) versus observed (Obs) concentration-time profile after administration of 300 mg PO MD [22–26]

A dosing regimen of PO 300 mg daily was assumed to calculate the milk transfer of tenofovir.

Dosing interval: 24 h	Plasma	Milk
Cmax (mg/L)	0.27	0.07
AUC (mg*h/L)	2.99	1.20
Cave (mg/L)	0.12	0.05

M/P ratio = 0.40

Model B: Alternatively, the logD values were overwritten with the values obtained from MarvinSketch (see section 4.2).

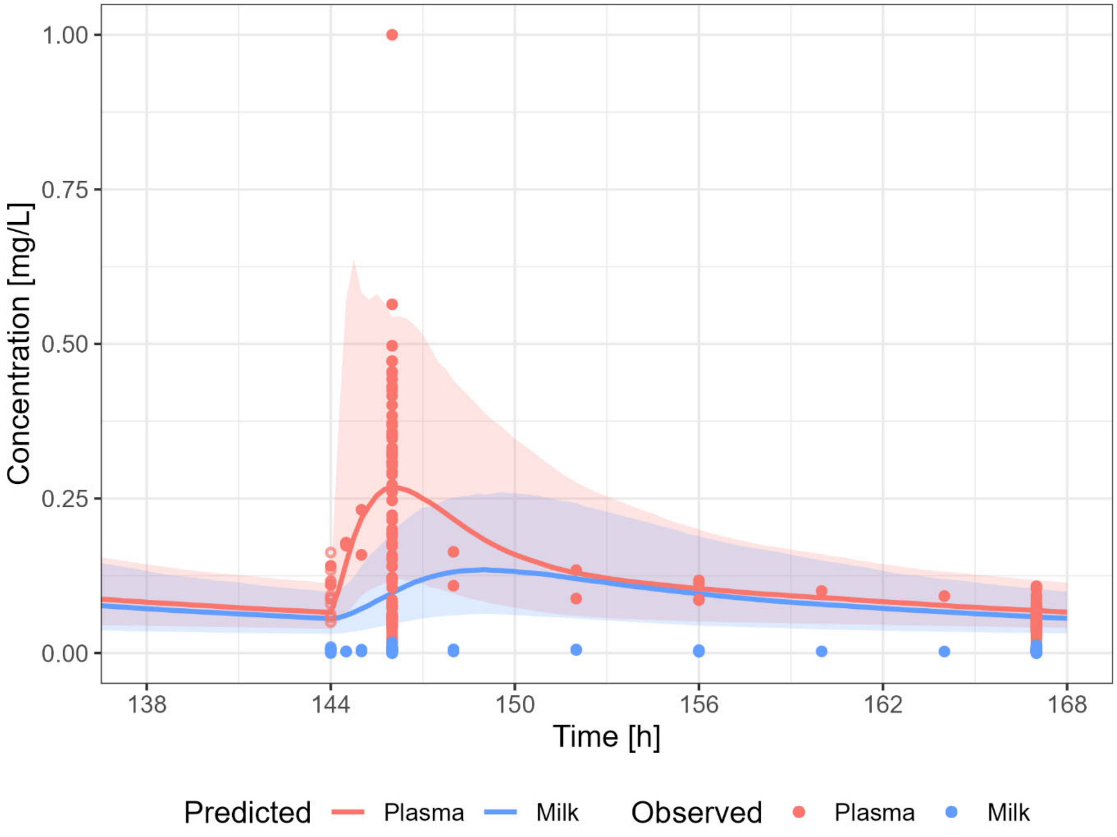

Figure S22 Predicted (Pred) versus observed (Obs) concentration-time profile after administration of 300 mg PO MD [22–26]

A dosing regimen of PO 300 mg daily was assumed to calculate the milk transfer of tenofovir.

Dosing interval: 24 h	Plasma	Milk
Cmax (mg/L)	0.27	0.13
AUC (mg*h/L)	2.99	2.20
Cave (mg/L)	0.12	0.09

M/P ratio = 0.74

Model C: Finally, the LogP value as well as the LogD values were overwritten with the values obtained from MarvinSketch (see section 4.2).

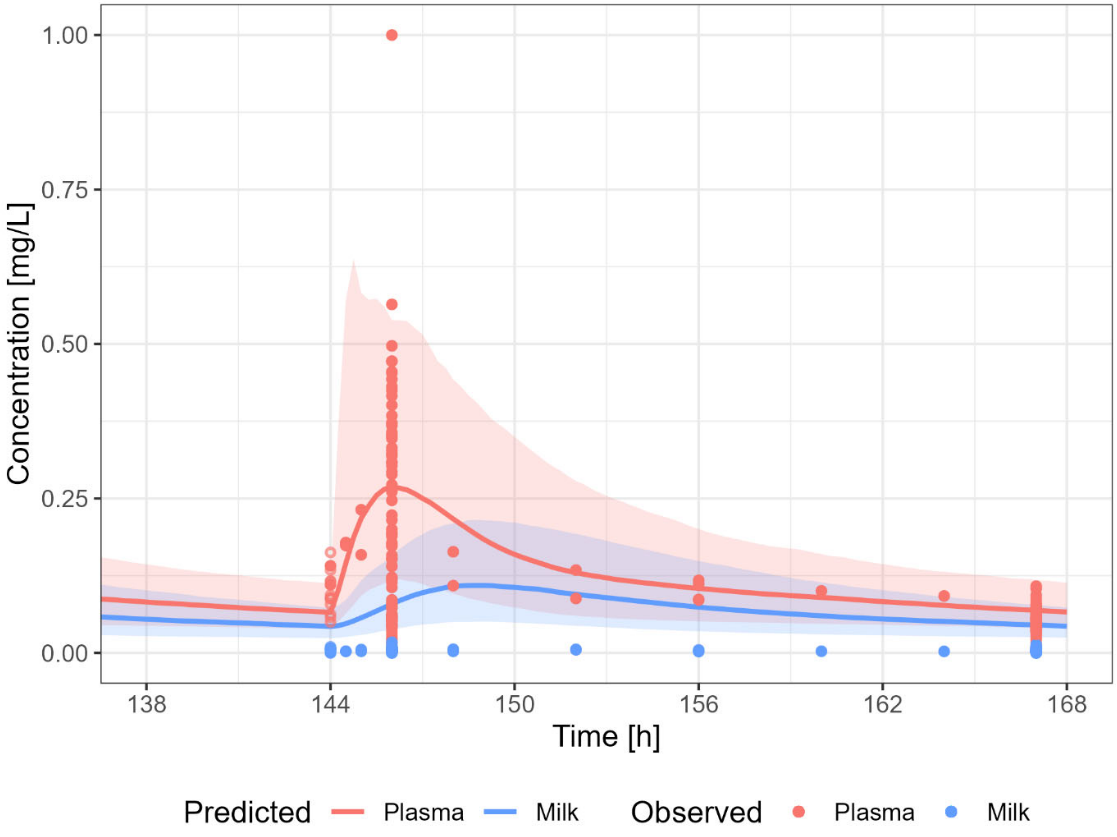

Figure S23 Predicted (Pred) versus observed (Obs) concentration-time profile after administration of 300 mg PO MD [22–26]

A dosing regimen of PO 300 mg daily was assumed to calculate the milk transfer of tenofovir.

Dosing interval: 24 h	Plasma	Milk
Cmax (mg/L)	0.27	0.11
AUC (mg*h/L)	2.99	1.73
Cave (mg/L)	0.12	0.07

M/P ratio = 0.58

Model A was selected as final PBPK model for lactation for amoxicillin. The M/P ratio was 0.40.

4.4 Estimated infant dosage

A maternal dosing regimen of PO 300 mg daily was used to calculate the infant dosage. The daily infant dosage and relative infant dose (RID) for 3 months old infants were calculated using a milk intake of 150 mL/kg/day. The daily infant dosage was 0.01 mg/kg/day (RID: 0.15 %) or 0.01 mg/kg/day (RID: 0.21 %) based on the average steady-state concentration and maximum concentration in human milk, respectively.

5. Discussion

First, the reference PBPK model was developed and evaluated. Evaluation of the predictive performance showed that the reference PBPK model for tenofovir was able to capture the pharmacokinetic behavior of the medicines in healthy volunteers and/or patients.

Next, the PBPK model was extended to a lactation PBPK model. The PBPK model results in an overprediction of the human milk concentrations.

The predicted M/P ratio (0.40) is 4- to 16-fold higher than the observed range of M/P ratios (0.025-0.11).

The calculated infant dosage should be interpreted with caution, since the predicted human milk concentration was too high compared to the observed data.

6. Conclusions

The herein presented PBPK model adequately describes the PK of tenofovir in adults including breastfeeding women. In particular, it applies the conversion of the prodrug via esterases. For tenofovir, it applies hepatic metabolism and renal excretion via glomerular filtration and active tubular secretion. The PBPK model for lactation results in an overprediction of the milk concentration (M/P ratio: 0.40). The daily infant dosage was 0.01 mg/kg/day (RID: 0.15 %) or 0.01 mg/kg/day (RID: 0.21 %) based on the average steady-state concentration and maximum concentration in human milk, respectively.

7. List of Appendix and Supplementary Materials

Supplementary material 1 – ObsDataPK_OSP_reference_tenofovir
Supplementary material 2 – ObsDataPK_OSP_lactation_tenofovir
Supplementary material 3 – Tenofovir.pksim5

8. References

[1] B.P. Kearney, J.F. Flaherty, J. Shah, Tenofovir disoproxil fumarate: clinical pharmacology and pharmacokinetics., Clin. Pharmacokinet. 43 (2004) 595–612. https://doi.org/10.2165/00003088-200443090-00003.

[2] S.G. Deeks, P. Barditch-Crovo, P.S. Lietman, F. Hwang, K.C. Cundy, J.F. Rooney, N.S. Hellmann, S. Safrin, J.O. Kahn, Safety, pharmacokinetics, and antiretroviral activity of intravenous 9- [2-(R)-(phosphonomethoxy)propyl]adenine, a novel anti-human immunodeficiency virus (HIV) therapy, in HIV-infected adults, Antimicrob. Agents Chemother. 42 (1998) 2380–2384. https://doi.org/10.1128/aac.42.9.2380.

[3] A. Dallmann, J. Solodenko, I. Ince, T. Eissing, Applied Concepts in PBPK Modeling: How to Extend an Open Systems Pharmacology Model to the Special Population of Pregnant Women, CPT Pharmacometrics Syst. Pharmacol. 7 (2018) 419–431. https://doi.org/10.1002/psp4.12300.

[4] A. Dallmann, A. Himstedt, J. Solodenko, I. Ince, G. Hempel, T. Eissing, Integration of physiological changes during the postpartum period into a PBPK framework and prediction of amoxicillin disposition before and shortly after delivery, J. Pharmacokinet. Pharmacodyn. 47 (2020) 341–359. https://doi.org/10.1007/s10928-020-09706-z.

[5] K.M. Job, A. Dallmann, S. Parry, G. Saade, D.M. Haas, B. Hughes, P. Berens, J. Chen, C. Fu, K. Humphrey, C. Hornik, S. Balevic, K. Zimmerman, K. Watt, Development of a Generic Physiologically-Based Pharmacokinetic Model for Lactation and Prediction of Maternal and Infant Exposure to Ondansetron via Breast Milk, Clin. Pharmacol. Ther. 111 (2022) 1111–1120. https://doi.org/10.1002/cpt.2530.

[6] H. Koshimichi, K. Ito, A. Hisaka, M. Honma, H. Suzuki, Analysis and prediction of drug transfer into human milk taking into consideration secretion and reuptake clearances across the mammary epithelia, Drug Metab. Dispos. 39 (2011) 2370–2380. https://doi.org/10.1124/dmd.111.040972.

[7] J. Van Gelder, S. Deferme, L. Naesens, E. De Clercq, G. Van Den Mooter, R. Kinget, P. Augustijns, Intestinal absorption enhancement of the ester prodrug tenofovir disoproxil fumarate through modulation of the biochemical barrier by defined ester mixtures, Drug Metab. Dispos. 30 (2002) 924–930. https://doi.org/10.1124/dmd.30.8.924.

[8] U.C. Atkinson, E.J. Begg, Prediction of Drug Distribution into Human Milk from Physicochemical Characteristics, Clin. Pharmacokinet. 18 (1990) 151–167. https://doi.org/10.2165/00003088-199018020-00005.

[9] B.-C. P., D. S.G., C. A., S. S., C. D.F., M. M., K. B.P., C. R.L., L. P.D., K. J.O., M. I., L. P.S., P. Barditch-Crovo, S.G. Deeks, A. Collier, S. Safrin, D.F. Coakley, M. Miller, B.P. Kearney, R.L. Coleman, P.D. Lamy, J.O. Kahn, I. McGowan, P.S. Lietman, Phase I/II trial of the pharmacokinetics, safety, and antiretroviral activity of tenofovir disoproxil fumarate in human immunodeficiency virus-infected adults, Antimicrob. Agents Chemother. 45 (2001) 2733–2739. https://doi.org/10.1128/AAC.45.10.2733-2739.2001 LK - http://limo.libis.be/resolver?&sid=EMBASE&issn=00664804&id=doi:10.1128%2FA

AC.45.10.2733-
2739.2001&atitle=Phase+I%2FII+trial+of+the+pharmacokinetics%2C+safety%2C+and+antiretroviral+activity+of+tenofovir+disoproxil+fumarate+in+human+immunodeficiency+virus-infected+adults&stitle=Antimicrob.+Agents+Chemother.&title=Antimicrobial+Agents+and+Chemotherapy&volume=45&issue=10&spage=2733&epage=2739&aulast=Barditch-Crovo&aufirst=P.&auinit=P.&aufull=Barditch-Crovo+P.&c.

[10] S. Geboers, S. Haenen, R. Mols, J. Brouwers, J. Tack, P. Annaert, P. Augustijns, Intestinal behavior of the ester prodrug tenofovir DF in humans, Int. J. Pharm. 485 (2015) 131–137. https://doi.org/10.1016/j.ijpharm.2015.03.002.

[11] A.A. Mathias, J. Hinkle, M. Menning, J. Hui, S. Kaul, B.P. Kearney, E. Tenofovir, Bioequivalence of Efavirenz / Emtricitabine / Tenofovir, 46 (2007) 167–173.

[12] B.P. Kearney, S. Ramanathan, A.K. Cheng, R. Ebrahimi, J. Shah, K. B.P., R. S., C. A.K., E. R., S. J., B.P. Kearney, S. Ramanathan, A.K. Cheng, R. Ebrahimi, J. Shah, Systemic and renal pharmacokinetics of adefovir and tenofovir upon coadministration, J. Clin. Pharmacol. 45 (2005) 935–940. https://doi.org/10.1177/0091270005278949 LK -
http://limo.libis.be/resolver?&sid=EMBASE&issn=00912700&id=doi:10.1177%2F0091270005278949&atitle=Systemic+and+renal+pharmacokinetics+of+adefovir+and+tenofovir+upon+coadministration&stitle=J.+Clin.+Pharmacol.&title=Journal+of+Clinical+Pharmacology&volume=45&issue=8&spage=935&epage=940&aulast=Kearney&aufirst=Brian+P.&auinit=B.P.&aufull=Kearney+B.P.&coden=JCPCB&isbn=&pages=935-940&date=2005&auinit1=B&auinitm=P.

[13] C.-Y. Hu, Y.-M. Liu, Y. Liu, Q. Chen, W. Wang, K. Wu, J. Dong, J. Li, J.-Y. Jia, C. Lu, S.-X. Sun, C. Yu, X. Li, Pharmacokinetics and tolerability of Tenofovir disoproxil fumarate 300 mg once daily: an open-label, single- and multiple-dose study in healthy Chinese subjects., Clin. Ther. 35 (2013) 1884–1889. https://doi.org/10.1016/j.clinthera.2013.09.020.

[14] L. Dickinson, H.M. Yapa, A. Jackson, G. Moyle, L. Else, A. Amara, S. Khoo, D. Back, Z. Karolia, C. Higgs, M. Boffito, Plasma tenofovir, emtricitabine, and rilpivirine and intracellular tenofovir diphosphate and emtricitabine triphosphate pharmacokinetics following drug intake cessation, Antimicrob. Agents Chemother. 59 (2015) 6080–6086. https://doi.org/10.1128/AAC.01441-15.

[15] C. Waitt, S. Diliiy Penchala, A. Olagunju, A. Amara, L. Else, M. Lamorde, S. Khoo, W. C., D.P. S., O. A., A. A., E. L., L. M., K. S., Development, validation and clinical application of a method for the simultaneous quantification of lamivudine, emtricitabine and tenofovir in dried blood and dried breast milk spots using LC–MS/MS, J. Chromatogr. B Anal. Technol. Biomed. Life Sci. 1060 (2017) 300–307. https://doi.org/10.1016/j.jchromb.2017.06.012 LK -
http://limo.libis.be/resolver?&sid=EMBASE&issn=1873376X&id=doi:10.1016%2Fj.jchromb.2017.06.012&atitle=Development%2C+validation+and+clinical+application+of+a+method+for+the+simultaneous+quantification+of+lamivudine%2C+emtricitabine+and+tenofovir+in+dried+blood+and+dried+breast+milk+spots+using+LC%E2%80%93MS%2FMS&stitle=J.+Chromatogr.+B+Anal.+Technol.+Biomed.+Life+Sci.&title=Journal+of+Chromatography+B%3A+Analytical+Technologies+in+the+Biomedical+and+Life+Scien.

[16] C. Waitt, A. Olagunju, S. Nakalema, I. Kyohaire, A. Owen, M. Lamorde, S. Khoo, W. C., O.O. A., N. S., K. I., O.O. A., L. M., K. S., C. Waitt, A. Olagunju, S. Nakalema, I. Kyohaire, A. Owen, M. Lamorde, S. Khoo, W. C., O.O. A., N. S., K. I., O.O. A., L. M., K. S., Plasma and breast milk pharmacokinetics of emtricitabine, tenofovir and

lamivudine using dried blood and breast milk spots in nursing African mother-infant pairs, J. Antimicrob. Chemother. 73 (2018) 1013–1019. https://doi.org/10.1093/jac/dkx507 LK - http://limo.libis.be/resolver?&sid=EMBASE&issn=14602091&id=doi:10.1093%2Fjac%2Fdkx507&atitle=Plasma+and+breast+milk+pharmacokinetics+of+emtricitabine%2C+tenofovir+and+lamivudine+using+dried+blood+and+breast+milk+spots+in+nursing+African+mother-infant+pairs&stitle=J.+Antimicrob.+Chemother.&title=Journal+of+Antimicrobial+Chemotherapy&volume=73&issue=4&spage=1013&epage=1019&aulast=Waitt&aufirst=Catriona&auinit=C.&aufull=Waitt+C.&coden=JACHD&isbn=&pages=1013-1019&date=2018&a.

[17] K.K. Mugwanya, C.W. Hendrix, N.R. Mugo, M. Marzinke, E.T. Katabira, K. Ngure, N.B. Semiyaga, G. John-Stewart, T.R. Muwonge, G. Muthuri, A. Stergachis, C.L. Celum, J.M. Baeten, M. K.K., H. C.W., M. N.R., M. M., K. E.T., N. K., S. N.B., J.-S. G., M. T.R., M. G., S. A., C. C.L., B. J.M., K.K. Mugwanya, C.W. Hendrix, N.R. Mugo, M. Marzinke, E.T. Katabira, K. Ngure, N.B. Semiyaga, G. John-Stewart, T.R. Muwonge, G. Muthuri, A. Stergachis, C.L. Celum, J.M. Baeten, M. K.K., H. C.W., M. N.R., M. M., K. E.T., N. K., S. N.B., J.-S. G., M. T.R., M. G., S. A., C. C.L., B. J.M., K.K. Mugwanya, C.W. Hendrix, N.R. Mugo, M. Marzinke, E.T. Katabira, K. Ngure, N.B. Semiyaga, G. John-Stewart, T.R. Muwonge, G. Muthuri, A. Stergachis, C.L. Celum, J.M. Baeten, M. K.K., H. C.W., M. N.R., M. M., K. E.T., N. K., S. N.B., J.-S. G., M. T.R., M. G., S. A., C. C.L., B. J.M., K.K. Mugwanya, C.W. Hendrix, N.R. Mugo, M. Marzinke, E.T. Katabira, K. Ngure, N.B. Semiyaga, G. John-Stewart, T.R. Muwonge, G. Muthuri, A. Stergachis, C.L. Celum, J.M. Baeten, Pre-exposure Prophylaxis Use by Breastfeeding HIV-Uninfected Women: A Prospective Short-Term Study of Antiretroviral Excretion in Breast Milk and Infant Absorption, PLoS Med. 13 (2016) e1002132. https://doi.org/10.1371/journal.pmed.1002132.

[18] E. U.S., M. B., O. R., S. N., I.B. I., M. A., K. B., S. O., G. B., S. O.A., T. F., The determination of tenofovir level in breast milk of nursing mothers under tenofovir therapy, Hepatology. 68 (2018) 256A-257A. https://doi.org/10.1002/hep.30257 LK - http://limo.libis.be/resolver?&sid=EMBASE&issn=15273350&id=doi:10.1002%2Fhep.30257&atitle=The+determination+of+tenofovir+level+in+breast+milk+of+nursing+mothers+under+tenofovir+therapy&stitle=Hepatology&title=Hepatology&volume=68&issue=&spage=256A&epage=257A&aulast=Erturk&aufirst=Umran+Sumeyse&auinit=U.S.&aufull=Erturk+U.S.&coden=&isbn=&pages=256A-257A&date=2018&auinit1=U&auinitm=S.

[19] L. Palombi, M.F. Pirillo, E. Marchei, H. Jere, J.-B. Sagno, R. Luhanga, M. Floridia, M. Andreotti, C.M. Galluzzo, S. Pichini, R. Mwenda, S. Mancinelli, M.C. Marazzi, S. Vella, G. Liotta, M. Giuliano, P. L., P. M.F., M. E., J. H., S. J.-B., L. R., F. M., A. M., G. C.M., P. S., M. R., M. S., M. M.C., V. S., L. G., G. M., L. Palombi, M.F. Pirillo, E. Marchei, H. Jere, J.-B. Sagno, R. Luhanga, M. Floridia, M. Andreotti, C.M. Galluzzo, S. Pichini, R. Mwenda, S. Mancinelli, M.C. Marazzi, S. Vella, G. Liotta, M. Giuliano, P. L., P. M.F., M. E., J. H., S. J.-B., L. R., F. M., A. M., G. C.M., P. S., M. R., M. S., M. M.C., V. S., L. G., G. M., L. Palombi, M.F. Pirillo, E. Marchei, H. Jere, J.-B. Sagno, R. Luhanga, M. Floridia, M. Andreotti, C.M. Galluzzo, S. Pichini, R. Mwenda, S. Mancinelli, M.C. Marazzi, S. Vella, G. Liotta, M. Giuliano, P. L., P. M.F., M. E., J. H., S. J.-B., L. R., F. M., A. M., G. C.M., P. S., M. R., M. S., M. M.C., V. S., L. G., G. M., Concentrations of tenofovir, lamivudine and efavirenz in mothers and children enrolled under the Option B-Plus approach in Malawi, J. Antimicrob. Chemother. 71 (2016) 1027–1030. https://doi.org/10.1093/jac/dkv435 LK -

http://limo.libis.be/resolver?&sid=EMBASE&issn=14602091&id=doi:10.1093%2Fjac%2Fdkv435&atitle=Concentrations+of+tenofovir%2C+lamivudine+and+efavirenz+in+mothers+and+children+enrolled+under+the+Option+B-Plus+approach+in+Malawi&stitle=J.+Antimicrob.+Chemother.&title=Journal+of+Antimicrobial+Chemotherapy&volume=71&issue=4&spage=1027&epage=1030&aulast=Palombi&aufirst=Leonardo&auinit=L.&aufull=Palombi+L.&coden=JACHD&isbn=&pages=1027-1030&date=2016&auinit1=L&auinitm=.
[20] C.W. Hendrix, B.A. Chen, V. Guddera, C. Hoesley, J. Justman, C. Nakabiito, R. Salata, L. Soto-Torres, K. Patterson, A.M. Minnis, S. Gandham, K. Gomez, B.A. Richardson, N.N. Bumpus, H. C.W., C. B.A., G. V., H. C., J. J., N. C., S. R., S.-T. L., P. K., M. A.M., G. S., G. K., R. B.A., B. N.N., MTN-001: Randomized Pharmacokinetic Cross-Over Study Comparing Tenofovir Vaginal Gel and Oral Tablets in Vaginal Tissue and Other Compartments, PLoS One. 8 (2013) e55013. https://doi.org/10.1371/journal.pone.0055013 LK - http://limo.libis.be/resolver?&sid=EMBASE&issn=19326203&id=doi:10.1371%2Fjournal.pone.0055013&atitle=MTN-001%3A+Randomized+Pharmacokinetic+Cross-Over+Study+Comparing+Tenofovir+Vaginal+Gel+and+Oral+Tablets+in+Vaginal+Tissue+and+Other+Compartments&stitle=PLoS+ONE&title=PLoS+ONE&volume=8&issue=1&spage=&epage=&aulast=Hendrix&aufirst=Craig+W.&auinit=C.W.&aufull=Hendrix+C.W.&coden=&isbn=&pages=-&date=2013&auinit1=C&auinitm=W.
[21] E. Taneva, K. Crooker, S.H. Park, J.T. Su, A. Ott, N. Cheshenko, I. Szleifer, P.F. Kiser, B. Frank, P.M.M. Mesquita, B.C. Herold, Differential mechanisms of tenofovir and tenofovir disoproxil fumarate cellular transport and implications for topical preexposure prophylaxis, Antimicrob. Agents Chemother. 60 (2016) 1667–1675. https://doi.org/10.1128/AAC.02793-15.
[22] U.S. Erturk, B. Mete, R. Ozaras, N. Saltoglu, I.I. Balkan, A. Mert, B. Kacmaz, O. Saglam, B. Guney, O.A. Sayman, F. Tabak, Plasma and breast milk pharmacokinetics of tenofovir disoproxil fumarate in nursing mother with chronic hepatitis B-infant pairs, Antimicrob. Agents Chemother. 65 (2021) 1–6. https://doi.org/10.1128/AAC.01110-21.
[23] K.K. Mugwanya, C.W. Hendrix, N.R. Mugo, M. Marzinke, E.T. Katabira, K. Ngure, N.B. Semiyaga, G. John-Stewart, T.R. Muwonge, G. Muthuri, A. Stergachis, C.L. Celum, J.M. Baeten, Pre-exposure Prophylaxis Use by Breastfeeding HIV-Uninfected Women: A Prospective Short-Term Study of Antiretroviral Excretion in Breast Milk and Infant Absorption., PLoS Med. 13 (2016) e1002132. https://doi.org/10.1371/journal.pmed.1002132.
[24] L. Palombi, M.F. Pirillo, E. Marchei, H. Jere, J.-B. Sagno, R. Luhanga, M. Floridia, M. Andreotti, C.M. Galluzzo, S. Pichini, R. Mwenda, S. Mancinelli, M.C. Marazzi, S. Vella, G. Liotta, M. Giuliano, Concentrations of tenofovir, lamivudine and efavirenz in mothers and children enrolled under the Option B-Plus approach in Malawi, J. Antimicrob. Chemother. 71 (2016) 1027–1030. https://doi.org/10.1093/jac/dkv435.
[25] C. Waitt, S. Diliiy Penchala, A. Olagunju, A. Amara, L. Else, M. Lamorde, S. Khoo, Development, validation and clinical application of a method for the simultaneous quantification of lamivudine, emtricitabine and tenofovir in dried blood and dried breast milk spots using LC-MS/MS., J. Chromatogr. B, Anal. Technol. Biomed. Life Sci. 1060 (2017) 300–307. https://doi.org/10.1016/j.jchromb.2017.06.012.
[26] C. Waitt, A. Olagunju, S. Nakalema, I. Kyohaire, A. Owen, M. Lamorde, S. Khoo, Plasma and breast milk pharmacokinetics of emtricitabine, tenofovir and lamivudine using dried blood and breast milk spots in nursing African mother-infant pairs, J. Antimicrob. Chemother. 73 (2018) 1013–1019. https://doi.org/10.1093/jac/dkx507.
