# Supplementary material for: Generic Workflow to Predict Medicine Concentrations in Human Milk Using Physiologically-Based Pharmacokinetic (PBPK) Modelling—A Contribution from the ConcePTION Project
Source: Pharmaceutics. 2023 May 11;15(5):1469. doi: 10.3390/pharmaceutics15051469 (PMC10223637; doi:10.3390/pharmaceutics15051469)
Supplement: Supplementary file 1 [file pharmaceutics-15-01469-s001.zip › File S9. Evaluation_report_valproic acid.pdf]

Building and evaluation of a Physiologically-Based PharmacoKinetic (PBPK) model for **valproic acid** in adults and lactating women

Disclaimer: The research project leading to these results was conducted as part of the ConcePTION consortium. This report only reflects the personal views of the stated authors. The results of this report are only intended for research purpose, and are not intended to be used in clinical practice.

Glossary

ASCM1	Acyl-CoA synthetase medium chain family member 1
AUC	Area Under the Curve
C_{ave}	Average concentration
CL_{int}	Intrinsic clearance
CL_{re}	Reuptake clearance (i.e. from milk to blood)
CL_{sec}	Secretion clearance (i.e. from blood to milk)
C_{max}	Maximum (~peak) concentration
DID	Daily Infant Dosage (expressed for instance in mg/kg/day)
f_u	Fraction unbound in plasma
GFR	Glomerular Filtration Rate
HBD	Hydrogen Bond Donors
IV	Intravenous (administration)
$LogD_{7.2}$	Logarithm of the partition coefficient between an octanol phase and an aqueous (buffer) phase at pH 7.2
$LogD_{7.4}$	Logarithm of the partition coefficient between an octanol phase and an aqueous (buffer) phase at pH 7.4
LogP	Logarithm of the partition coefficient between an octanol phase and (unbuffered) water as aqueous phase. This is the default parameter to express lipophilicity of a substance.
MD	Multiple dose
M/P ratio	Milk-to-Plasma ratio
MW	Molecular Weight (Da)
PBPK	Physiologically-Based PharmacoKinetic [*modeling*]
pKa	Logarithm of the acid dissociation constant
PO	Oral administration
PSA	Polar Surface Area
RID	Relative Infant Dose (%)
SD	Single dose
UGT	Glucuronosyl transferase
VPA	Valproic acid

1. Table of Contents

1. Table of Contents 3

2. Introduction 4

3. Methods 5

3.1 Modelling strategy 5

3.1.1. Reference PBPK models 6

3.1.2. Lactation model 6

3.2 Data 6

3.2.1 *In vitro* / physicochemical data 6

3.2.2 Clinical data 9

3.3 Model Parameters and assumptions 12

3.3.1 Absorption 12

3.3.2 Distribution 12

3.3.3 Metabolism and excretion 12

3.3.4 Secretion to milk 13

3.3.5 Automated parameter optimization 13

3.4. Infant dosage calculation 14

4. Results 14

4.1 Final input parameters 15

4.2 Diagnostic plots 16

4.3 Concentration-time profiles 20

4.3.1 Model building 21

4.3.2 Model verification 33

4.3.3 Lactation PBPK model 51

4.4 Estimated infant dosage 67

5. Discussion 67

6. Conclusion 68

7. List of Appendix and Supplementary Materials 69

8. References 69

2. Introduction

Valproic acid (VPA, figure S1) is and anticonvulsant is indicated for [1]:

(i) Complex partial seizures and simple or complex absence seizures
(ii) Adjunctive therapy in the management of multiple seizure types that include absence seizures
(iii) Prophylaxis of migraine headaches
(iv) Acute management of mania associated with bipolar disorder.

Recommended doses range from 4 mg/kg/day up to 20 mg/kg/day [2]. Therapeutic plasma concentrations range from 40 to 100 µg/mL. VPA exists as intravenous, and different oral formulations, including extended release or delayed release formulations. Absorption of VPA is rapid, and peak concentrations are reached within 1 to 4 h [3]. Bioavailability is estimated to be 90-100 %. The volume of distribution is small (0.13-0.20 L/kg). VPA is highly bound to plasma albumin (7-15 % unbound), and it has been described that concentration-dependent protein binding is responsible for nonlinear kinetics. VPA is mainly metabolized via glucuronidation by different UGT enzymes (UGT1A1, 1A3, 1A4, 1A6, 1A8, 1A9, 1A10, and 2B7). About 40 % of the metabolism occurs via mitochondrial β-oxidation. cytochrome P450 (CYP) oxidation is responsible < 10 % of the metabolism (CYP2C9, CYP2A6, CYP2B6, and possibly CYP2C19), and only a small fraction (< 3 %) is excreted unchanged in the urine. The elimination half-life is 4-16 h, depending on the population.

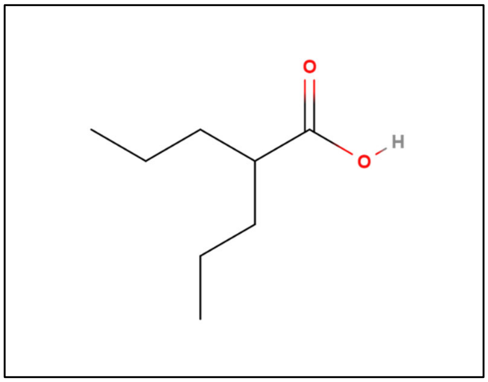

Figure S1. Chemical Structure of valproic acid

The scope of this report is to:

(a) specify the details and underlying assumptions associated with the building of physiologically-based pharmacokinetic (PBPK) models for VPA in adult healthy volunteers or patients, and in postpartum women during lactation.
(b) evaluate the predictive performance of these PBPK models. This is achieved by comparing model-predicted plasma or milk concentrations with corresponding clinical observations.

3. Methods

The software used for the development of PBPK models presented in this report is tabulated below:

Software	Version
PK-Sim®	v9.1
MoBi®	v9.1

3.1 Modelling strategy

In the present report, a reference PBPK model was first established for adults (patients as well as healthy volunteers), and subsequently verified against clinical pharmacokinetic data reported for VPA in the scientific literature.

Relevant information on the anthropometry (height, weight) was gathered from the respective clinical studies, if reported. Information on physiological parameters (e.g. blood flows, organ volumes, hematocrit) in adults is available in the PK-Sim® database.

In a second step, a lactation PBPK model was developed, based on the general workflow described by Dallmann *et al. 2018* [4–6].

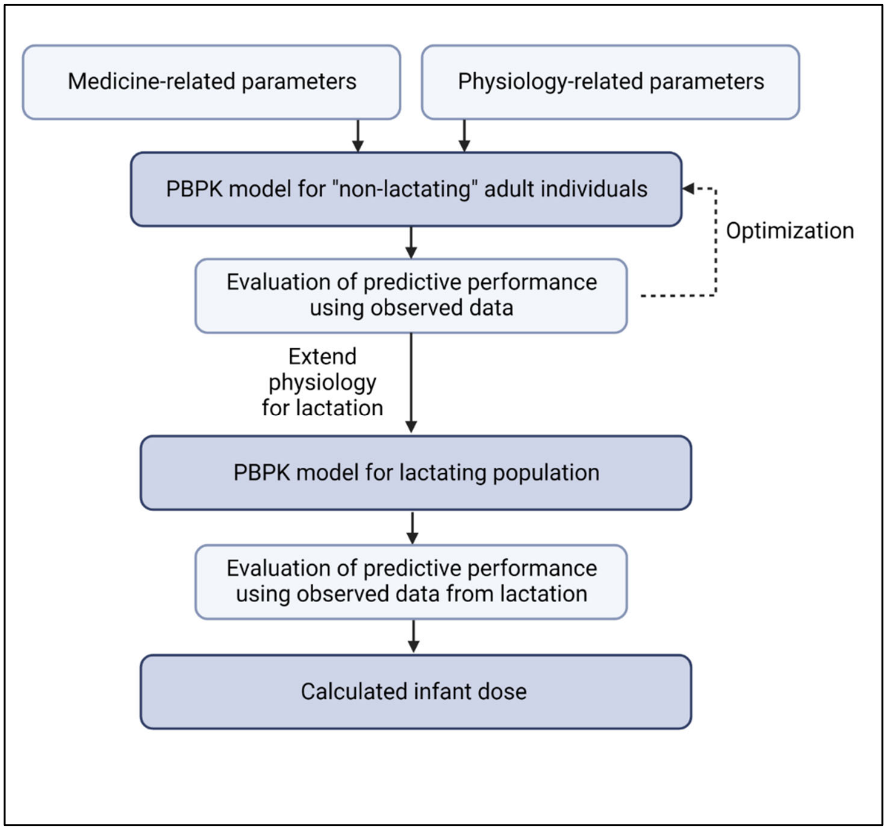

Figure S2 General workflow that was used in the present project to develop and evaluate the lactation PBPK model

Details about input data (physicochemical, *in vitro* and clinical data) can be found in section 3.2. Details about the structural models and their parameters can be found in section 3.3.

3.1.1. Reference PBPK models

The reference PBPK models were built based on studies with adult volunteers and/or adult patients, using the reported mean values for age, weight, height, and genetic background as described in each study protocol. When no information on these parameters could be found, a healthy male European individual, 30 years of age, with a body weight of 73 kg and a height of 176 cm was used.

The abundance (including population variability) of plasma proteins and enzymes/transporters that are integrated into PK-Sim are described in the publicly available 'PK-Sim Ontogeny Database Version 7.3' (PK-Sim Ontogeny Database Version 7.3).

To model the specific metabolic clearance of VPA, it was assumed that glucuronidation (UGT1A3/4/6/8/9/10 and UGT2B7) was responsible for 49 % if the metabolism, beta-oxidation (ASCM1) for 40 %, oxidation (CYP2C9) for 10 % and renal clearance for 1 %. UGT1A3/4/6/8/9/10, UGT2B7, ASCM1 and CYP2C9 were implemented in accordance with literature, using the PK-Sim expression database array profiles to define their relative expression in the different organs of the body. Glucuronidation enzymes were implemented using in vitro metabolic rate in the presence of liver microsomes based on Michaelis-Menten kinetics. CYP2C9 and ASCM1 were implemented using intrinsic clearance – first order. Renal excretion was implemented as kidney plasma clearance.

Structural model selection was mainly guided by biological plausibility and by visual inspection of the predicted concentration time profiles in comparison with observed data. The generally applied acceptance criterium was less than 2-fold misprediction. Uninformed parameter values (see below) were estimated using the parameter identification module of PK-Sim®.

The predictive performance of the models was evaluated by simulating:

- Single intravenous dose studies
- Single and multiple oral dose studies
- Fed and fasted state studies

For some parameters, parameter optimization was performed as described below to obtain improved concordance between predicted profiles and observed data.

3.1.2. Lactation model

After development of the reference model, the model was exported to MoBi® and a lactation PBPK model was constructed. To model the passage of VPA into human milk, i.e. across the blood/milk biological barrier, both the secretion (CL_{sec}) and reuptake clearance (CL_{re}) values were obtained using the empirical model developed by Koshimichi *et al. 2011* [7].

3.2 Data

3.2.1 *In vitro* / physicochemical data

A literature search was performed to collect available information on physicochemical properties of VPA. The obtained information from literature is summarized in Table S1. Table S2 shows the parameters that were additionally used for the lactation PBPK model.

Table S1 Physicochemical parameters used as input for the valproic acid (VPA) PBPK models

Parameter	Value	Unit	Description	Source
MW	144.21	g/mol	Molecular weight	Pubchem
pK_a	4.80 (acid)	-	Logarithm of the acid dissociation constant	Pubchem
Solubility (pH 7)	2.00	mg/mL	Aqueous solubility	Pubchem
LogP	2.75	-	Log_{10} of the partition coefficient between octanol and water (~lipophilicity)	Pubchem
f_u	0.14	-	Fraction unbound in human plasma	[8]
UGT1A3 In vitro Vmax/liver microsomes	600	pmol/min/mg mic. protein	Metabolic enzyme activity	[9]
UGT1A3 Km	6400	µM		
UGT1A4 In vitro Vmax/liver microsomes	400	pmol/min/mg mic. protein		
UGT1A4 Km	3100	µM		
UGT1A6 In vitro Vmax/liver microsomes	700	pmol/min/mg mic. protein		
UGT1A6 Km	3200	µM		
UGT1A8 In vitro Vmax/liver microsomes	870	pmol/min/mg mic. protein		
UGT1A8 Km	5900	µM		
UGT1A9 In vitro Vmax/liver microsomes	960	pmol/min/mg mic. protein		
UGT1A9 Km	6300	µM		
UGT1A10 In vitro Vmax/liver microsomes	880	pmol/min/mg mic. protein		
UGT1A10 Km	5500	µM		
UGT2B7 In vitro Vmax/liver microsomes	1200	pmol/min/mg mic. protein		
UGT2B7 Km	1400	µM		

Table S2 Physicochemical parameters used as input for the lactation PBPK model of valproic acid (VPA)

Parameter	Value	Unit	Description	Source
Milk logP[a]	2.75	-	Log_{10} of the partition coefficient between octanol and water	Pubchem
HBD	1.00	-	Hydrogen bond donors	Pubchem
PSA	37.30	Å^2	Polar surface area	Pubchem

[a] Milk logP is Log_{10} of the partition coefficient between octanol and water and is used as input for the calculations in the postpartum model (see equations below). In theory, this value is identical to the logP specified in Table S1. However, in some PBPK models, logP (Table S1) might be optimized using parameter identication. Therefore, it was chosen to use a separate parameter (i.e. Milk logP) to represent the logP used as input for the equations in the postpartum model.

The default equations for free fraction in human milk and logD that were implemented in the spatial structure building block that was developed for the postpartum women are described below. Alternatively, these values can be overwritten by values calculated elsewhere (e.g. MarvinSketch) or determined *in vitro.*

The free fraction in human milk was calculated with the equations proposed by Atkinson and Begg [10], as follows:

$$f_{u\_skimmed\ milk} = \frac{f_u \times 0.448}{(0.000694^{0.448} + \mathrm{f}_\mathrm{u}^{0.448})}$$

$$P_{milk} = 10^{(-0.88+1.29\times \mathrm{logD7.2})}$$

$$\text{Total free fraction in milk} = \frac{1}{(\frac{0.955}{f_{u\_skimmed\ milk}} + 0.045 \times P_{milk})}$$

Where: *f_u skimmed milk:* binding to proteins in milk; P_{milk}: partitioning between aqueous and lipid phase of milk; Total free fraction in milk: ‘total’ free fraction, i.e. accounting for both protein and lipid binding processes.

LogD values taking into account up to three pka values (as provided in the compound building block), were calculated as follows:

$$LogD = \mathrm{Log}\,P + Log_{10}(logD_{factor})$$

With Milk logP (Table S2) as input for logP

$$LogD_{factor} = \mathrm{K}_1 + (\mathrm{K}_2 + \mathrm{K}_3 + \mathrm{K}_4) \times \mathrm{base}^1 + \mathrm{K}_5 \times \mathrm{base}^{\max(\mathrm{CT}_0+\mathrm{CT}_1;-\mathrm{CT}_0-\mathrm{CT}_1)} + \mathrm{K}_6 \times \mathrm{base}^{\max(CT_0+CT_2;-CT_0-CT_2)} + \mathrm{K}_7 \times \mathrm{base}^{\max(\mathrm{CT}_2+\mathrm{CT}_1;-\mathrm{CT}_2-\mathrm{CT}_1)} + \mathrm{K}_8 \times \mathrm{base}^{\max(\mathrm{CT}_0+\mathrm{CT}_1+\mathrm{CT}_2;-\mathrm{CT}_0-\mathrm{CT}_1-\mathrm{CT}_2)}$$

$K_1 = F_1 \times F_2 \times F_3$
$K_2 = (1 - F_1) \times F_2 \times F_3$
$K_3 = F_1 \times (1 - F_2) \times F_3$
$K_4 = F_1 \times F_2 \times (1 - F_3)$
$K_5 = (1 - F_1) \times (1 - F_2) \times F_3$
$K_6 = (1 - F_1) \times F_2 \times (1 - F_3)$
$K_7 = (1 - F_1) \times F_2 \times (1 - F_3)$
$K_8 = (1 - F_1) \times (1 - F_2) \times (1 - F_3)$

F1 = CT0 != CT_NEUTRAL ? 1/(1+10^(CT0*(pKa_0- pH))) : 1
F2 = CT1 != CT_NEUTRAL ? 1/(1+10^(CT1*(pKa_1- pH))) : 1
F3 = CT2 != CT_NEUTRAL ? 1/(1+10^(CT2*(pKa_2- pH))) : 1

With CT = compound type (-1: acid; +1: base; 0: neutral), and pH = 7.2 or 7.4 respectively for $logD_{7.2}$ and $logD_{7.4}$

The transports that were added in the passive transport building block for 'transfer to milk' and 'transfer from milk' are based on secretion and reuptake and clearance values, Cl_{sec} and Cl_{re}, which were calculated according to the empirical equations proposed by Koshimich et al. 2011 [7], as follows:

$$\mathrm{Log}\, CL_{re} = 2.793 + 0.179 \times LogP - 0.132 \times HBD$$

$$\mathrm{Log}\, CL_{sec} = 3.367 \times Log10(MW) - 0.164 \times (LogP - LogD) - 0.015 \times PSA - 3.912$$

3.2.2 Clinical data

Literature searches were performed to collect available data on VPA in adults and postpartum women. The VPA reference PBPK model was developed using different clinical studies with pharmacokinetic (PK) blood sampling. First, a cross-over clinical trials, with oral and intravenous administration to 6 individuals was taken into account [11]. The observed data from the intravenous administration, together with fraction excreted into urine (1 %) and fraction metabolized via CYP2C9 (10%) and ASCM1 (40%) were used to estimate clearance parameters. Next, the oral administration was used to estimate formulation parameters. Another study where valproic acid was administered oral and intravenous to 6 individuals was used for evaluation of the predictive performance [12]. A study with different doses of intravenous administration was also used for validation [13]. Subsequently, the single and multiple dose administration from two additional clinical trials were used for evaluation of the predictive performance [14,15]. In addition, two more studies with oral administrations were used for model verification [16,17]. Finally, two clinical trials investigating the food effect on VPA pharmacokinetics after oral administration were used for additional verification [18,19].

The evaluation of the predictive performance of the VPA lactation PBPK model was performed using different studies were valproic acid was administered in different doses to lactating

women [20–24]. The women were between 3 days and 118 days postpartum. The samples were assumed to be trough samples if the exact timing was not reported in the articles.

Detailed information and data from the studies used for model building, verification, and lactation model can be found in Supplementary material 1 and 2.

3.2.2.1 Model building

The studies that were used for model building are shown in Table S3 (**training data**).

Table S3 Summary of studies used for PBPK model building of valproic acid (VPA) in reference populations

**Study ID**	**Reference**	**Arm/treatment/information used for model building**
Nitsche and Mascher 1982	[11]	6 individuals received 1000 mg VPA IV (single dose)
Nitsche and Mascher 1982	[11]	6 individuals received 1000 mg VPA PO (multiple dose)
Nitsche and Mascher 1982	[11]	6 individuals received 900 mg (2x450 mg) VPA PO (multiple dose)
Nitsche and Mascher 1982	[11]	6 individuals received 900 mg (3x300 mg) VPA PO (multiple dose)

Table S4 Demographic information

**Study ID**	**Reference**	**Number of subjects (female ratio)**	**Age (year)**	**Weight (kg)**
Nitsche and Mascher 1982	[11]	6	- (19-31)	- (62-79)

3.2.2.2 Model verification

The studies that were used to evaluate the predictive performance of the PBPK model are shown in

Table S5 (**verification data**).

Table S5 Summary of studies used for model verification of valproic acid (VPA) PBPK model in reference population

**Study ID**	**Reference**	**Arm/treatment/information used for model verification**
Dulac 2005	[14]	27 subjects received 500 mg sodium valproate bidaily PO (multiple dose)
Dulac 2005	[14]	27 subjects received 500 mg sodium valproate bidaily PO (single dose)
Perucca 1978	[12]	6 individuals received 800 mg VPA IV (single dose)
Perucca 1978	[12]	6 individuals received 800 mg VPA PO (single dose)
Rha 1993	[15]	12 subjects received 300 mg VPA PO (single dose)

Rha 1993	[15]	12 subjects received 1200 mg/day of VPA PO (multiple dose)
Pokjarac 1993	[17]	5 males and 5 females received 900 mg/day sodium valproate PO (multiple dose)
Ibarra 2013	[16]	7 females received 500 mg VPA PO (single dose)
Ibarra 2013	[16]	7 males received 500 mg VPA PO (single dose)
Georgoff 2018	[13]	6 subjects receive 15 mg/kg IV (single dose)
Georgoff 2018	[13]	6 subjects receive 30 mg/kg IV (single dose)
Georgoff 2018	[13]	6 subjects receive 60 mg/kg IV (single dose)
Georgoff 2018	[13]	6 subjects receive 90 mg/kg IV (single dose)
Georgoff 2018	[13]	6 subjects receive 120 mg/kg IV (single dose)
Georgoff 2018	[13]	6 subjects receive 130 mg/kg IV (single dose)
Georgoff 2018	[13]	6 subjects receive 140 mg/kg IV (single dose)
Georgoff 2018	[13]	2 subjects receive 150 mg/kg IV (single dose)
Chun 1980	[18]	14 subjects received 250 mg VPA PO syrup fasted (single dose)
Chun 1980	[18]	14 subjects received 250 mg VPA PO syrup fed (single dose)
Chun 1980	[18]	9 subjects received 250 mg VPA PO syrup fasted (single dose)
Chun 1980	[18]	9 subjects received 250 mg VPA PO capsule fasted (single dose)
Chun 1980	[18]	15 subjects received 250 mg VPA PO capsule fed (regimen A) (single dose)
Chun 1980	[18]	15 subjects received 250 mg VPA PO capsule fed (regimen B) (single dose)
Chun 1980	[18]	15 subjects received 250 mg VPA capsule fasted (single dose)
Ohdo 1992	[19]	8 subjects received 800 mg PO with a heavy meal in the evening (1) (single dose)
Ohdo 1992	[19]	8 subjects received 800 mg PO with a light meal in the morning (1) (single dose)
Ohdo 1992	[19]	8 subjects received 800 mg PO with a light meal in the evening (2) (single dose)
Ohdo 1992	[19]	8 subjects received 800 mg PO with a light meal in the morning (2) (single dose)

Table S6 Demographic information

**Study ID**	**Reference**	**Number of subjects (female ratio)**	**Age (year)**	**Weight (kg)**
Dulac 2005	[14]	27 (0)	24.3 (18-34)	-
Perucca 1978	[12]	6 (0)	- (22-38)	68 (60-76)
Rha 1993	[15]	12 (-)	-	-
Pokjarac 1993	[17]	10 (0.50)	28 (19-48)	61 (45-70)
Ibarra 2013	[16]	7 (0)<br>7 (1)	- (19-35)<br>- (19-35)	79 ± 9.7<br>59 ± 8.3
Georgoff 2018	[13]	6 (-)	30.2 (18-65)	78.8 ± 14.3

		6 (-)<br>6 (-)<br>6 (-)<br>6 (-)<br>6 (-)<br>6 (-)<br>2 (-)	30.2 (18-65)<br>30.2 (18-65)<br>30.2 (18-65)<br>30.2 (18-65)<br>30.2 (18-65)<br>30.2 (18-65)<br>30.2 (18-65)	71.7 ± 9.6<br>66.8 ± 6.9<br>77.4 ± 14.7<br>74.9 ± 12.2<br>82.1 ± 9.5<br>77.9 ± 6.8<br>71
Chun 1980	[18]	14 (0)<br>9 (0)<br>15 (0)	24 (21-31)<br>24 (21-28)<br>31 (22-41)	75 (63 – 91)<br>77 (68 – 88)<br>72 (66 – 83)
Ohdo 1992	[19]	8 (0)	25.3 (22-31)<br>25.1 (22-29)	67.1 ± 7.7<br>67.5 ± 9

3.2.2.3 Lactation PBPK model

Table S7 shows the study that was used for the lactation PBPK model.

Table S7 Summary of study used for PBPK model development of valproic acid (VPA) in lactating women

**Study ID**	**Publication**	**Arm/treatment/information used for model building and verification**
Alexander 1979	[20]	1 woman (29 days postpartum) received PO 1600 mg/day (multiple dose)
Kacirova 2019	[25]	17 women (6-32 days postpartum) received PO 11.3 mg/kg (multiple dose)
Nau 1981	[26]	11 women (3-58 days postpartum) received PO 9.5 – 31.0 mg/kg (multiple dose)
Tsuru 1998	[23]	3 women (1-118 days postpartum) received PO 1000-1400 mg/day (multiple dose)
VonUnruh 1984	[24]	16 women received PO 900-2400 mg/day (multiple dose)

3.3 Model Parameters and assumptions

3.3.1 Absorption

The convulex capsule formulation was implemented as Weibull function, and parameter were as determined via parameter identification (see 3.3.5) [11]. The same Weibull formulation was assumed for the other studies, although different kind of tablets and/or capsules were administered. The release from a syrup was implemented as a dissolved formulation. For the lactation PBPK model, we assumed the Weibull function.

3.3.2 Distribution

An important parameter influencing the distribution of a compound is lipophilicity. Lipophilicity was taken from Pubchem (LogP: 2.75). The tissue partition coefficients (Kp) calculation was according to 'Rodgers and Rowland' and the cellular permeability calculation was 'PK-dependent Schmitt normalized to PK-Sim'.

3.3.3 Metabolism and excretion

The final model applies metabolism by glucuronidation (49 %), beta-oxidation (40 %), cytochrome P450 oxidation (10 %), and renal clearance (1 %). For glucuronidation, the *in vitro* values determined in microsomes were used [9]. For beta-oxidation, it was assumed that

ASCM1 is the enzyme responsible for this pathway. The intrinsic clearance was estimated via parameter identification, based on the assumption that this pathway is responsible for 40 % of the metabolism. Similarly, it was assumed that cytochrome P450 oxidation is trough CYP2C9, and responsible for 10% of the total metabolism to estimate the intrinsic clearance. Finally, kidney plasma clearance was estimated based on the assumption that this pathway accounts for 1 % of the metabolism. Parameter identification was performed using the observed data from the intravenous profiles from Nitsche and Mascher (1982) [11].

3.3.4 Secretion to milk

To model the transfer process of valproic acid into human milk, both the secretion (CL_{sec}) and reuptake clearance (CL_{re}) were calculated using the empirical equations developed by Koshimichi *et al.* 2011 (see 3.2.1 *In vitro* / physicochemical data) [4].

First, in MoBi®, a spatial structure for the postpartum women was constructed, similar to the workflow from Dallmann *et al.* 2018 [2]. Here, breasts were added as a compartment. In addition, the human milk was connected to the plasma subcompartment of the breasts. The human milk volume was specified as 0.5 L to represent the structure of Koshimichi *et al.* 2011, and a geometric standard deviation of 1.16 was assumed in the population. The free fraction in human milk, and logD values were implemented as the equations described previously. The transfer between plasma and milk was defined as two kinetic processes (transfer to milk and transfer from milk) under passive transports (see below). Next, the simulation was combined with the postpartum population from Job *et al.* 2021 in PK-Sim to account for the postpartum physiology [3].

<u>*Kinetics*</u>

Transfer to milk

$$\frac{dNmilk}{dt} = Cplasma \times f_u \times CL_{sec}$$

where Cplasma is the concentration in plasma (in breast compartment), f_u is the free fraction in plasma and CL_{sec} is the secretion clearance.

Transfer from milk

$$\frac{dNplasma}{dt} = Cmilk \times f_u \times CL_{re}$$

where Cmilk is the concentration in human milk, f_u is the total free fraction in human milk (protein and lipid) and CL_{re} is the reuptake clearance.

The median simulated plasma and human milk concentration-time profiles can be used to calculate the M/P ratio as follows:

$$M/P\ ratio\ = \frac{AUC_{milk}}{AUC_{plasma}}$$

3.3.5 Automated parameter optimization

The following table depicts the results of the final parameter optimization according to the different clinical studies.

a) Nitsche and Mascher (1982) 1000 mg IV (single dose) [11]:

**Model parameter**	**Optimized value**	**Unit**
CYP2C9 - Intrinsic clearance	0.11	L/h
ASCM1 - Intrinsic clearance	5.00E-3	L/min
Kidney – Plasma clearance	0.01	mL/h/kg

b) Nitsche and Mascher (1982) 900 mg and 1000 mg PO [11]:

**Model parameter**	**Optimized value**	**Unit**
Dissolution time (50 % dissolved)	91.84	Min
Dissolution shape	2.43	-

3.4. Infant dosage calculation

Infant dosage via human milk was then calculated based on the predicted (average and maximal) steady-state VPA concentration in human milk, as well as the daily milk intake volume. The daily infant dosage was then compared to the maternal dosage, resulting in the relative infant dose (RID).

$$Daily\ infant\ dosage = C_{average} * 150\ \frac{mL}{kg\ .day}$$

$$Daily\ infant\ dosage = C_{max} * 150\ \frac{mL}{kg\ .day}$$

$$Relative\ infant\ dose\ (RID) = \frac{Infant\ dosage}{Maternal\ dosage} * 100\ \%$$

4. Results

Both the reference and postpartum PBPK model of VPA was developed and verified with clinical PK data.

The models were evaluated covering studies including in particular:

- Intravenous and oral administration
- Single and multiple doses
- A dose range from 250 up to 1000 mg
- Fed and fasted state

The model describes the metabolism glucuronidation, beta-oxidation, Cytochrome P450 oxidation and renal excretion for VPA. Moreover, secretion and reuptake to human milk were described by CL_{sec} and CL_{re}.

The next sections show:

- The final model parameters for the building blocks: section 4.1
- The overall predictive performance: section 4.2
- The simulated versus observed concentration-time profiles for the clinical studies used for model building and for model verification: section 4.3

4.1 Final input parameters

The compound values of the final postpartum PBPK model for VPA are illustrated below.

Physicochemical parameters

Parameter	Value	Unit	Source
MW	144.21	g/mol	Pubchem
pKa	4.80	-	Pubchem
Solubility	2.00	mg/mL	Pubchem
Lipophilicity	2.75	-	Pubchem
f_u	0.14	-	Cloyd et al. 2003 [8]
Small molecule (Y/N)	Yes	-	-
Plasma protein binding partner	Albumin		-

Calculation methods

Name	Value
Tissue partition coefficients	Rodgers and Rowland
Cellular permeabilities	Charge dependent Schmitt normalized to PK-Sim

AMDE-related parameters

Parameter	Value	Unit	Source
Intestinal permeability	1.73E-3	cm/min	Default
Intrinsic clearance ASCM1	5.00E-3	L/min	Parameter identification – assumption 40 %
Intrinsic clearance CYP2C9	0.11	L/h	Parameter identification – assumption 10 %
Kidney plasma clearance	0.01	mL/min/kg	Parameter identification – assumption 1 %
UGT1A3 In vitro Vmax/liver microsomezs	600	pmol/min/mg mic. protein	[9]
UGT1A3 Km	6400	µM	
UGT1A4 In vitro Vmax/liver microsomezs	400	pmol/min/mg mic. protein	
UGT1A4 Km	3100	µM	
UGT1A6 In vitro Vmax/liver microsomezs	700	pmol/min/mg mic. protein	
UGT1A6 Km	3200	µM	

UGT1A8 In vitro Vmax/liver microsomezs	870	pmol/min/mg mic. protein	
UGT1A8 Km	5900	µM	
UGT1A9 In vitro Vmax/liver microsomezs	960	pmol/min/mg mic. protein	
UGT1A9 Km	6300	µM	
UGT1A10 In vitro Vmax/liver microsomezs	880	pmol/min/mg mic. protein	
UGT1A10 Km	5500	µM	
UGT2B7 In vitro Vmax/liver microsomezs	1200	pmol/min/mg mic. protein	
UGT2B7 Km	1400	µM	

Formulation-related parameters
Type: Weibull

Formulation-related parameters

Parameter	Value	Unit	Source
Dissolution shape	2.43	-	Parameter identification
Dissolution time	91.84	min	

Physicochemical and physiological parameters relevant to the lactation model

Parameter	Value	Unit	Source
Milk logP	2.75	-	Pubchem
HBD	1.00	-	Pubchem
PSA	37.30	$Å^2$	Pubchem
CL_{sec}	4.14E-3	L/min	Default
CL_{re}	0.02	L/min	Default
*fu*_skimmed milk[a]	0.92	-	Default
Pmilk[b]	0.50	-	Default
Total free fraction in milk[c]	0.94	-	Default
$logD_{7.2}$	0.45	Log units	Default
$logD_{7.4}$	0.29	Log units	Default

[a] binding to proteins in milk; [b] partitioning between aqueous and lipid phase of milk; [c] total free fraction, accounting for both protein and lipid binding

4.2 Diagnostic plots

The geometric mean fold errors (GMFE) on AUC and Cmax were 1.24 and 1.19 for the model building dataset, and 1.37 and 1.36 for the model verification dataset.

The following plot shows the predictive performance graph for Cmax and AUC of VPA for the PBPK model performance of all data used.
Predicted over observed ratio values of all data listed in section 3.2.2 are presented below.

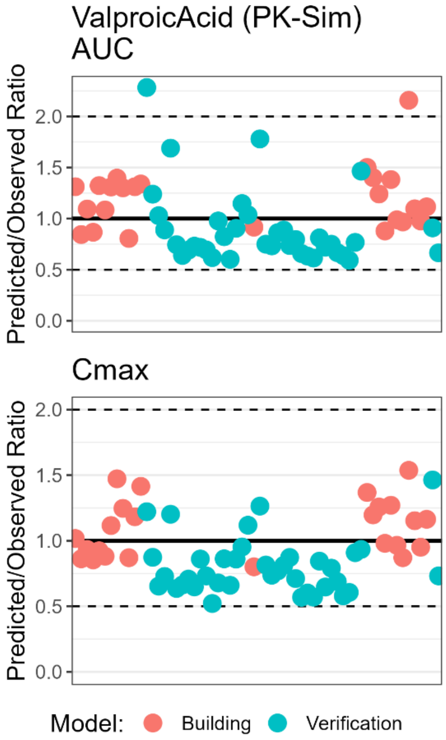

Figure S3. Predicted over observed ratio profile

Table S8 Ratio between the predicted and observed pharmacokinetic parameters of valproic acid (VPA) in different dosing regimens for model building

Study ID/ Reference	Dose/ Route	AUC_{obs} (mg*h/L)	AUC_{pred} (mg*h/L)	Fold error	$Cmax_{obs}$ (mg/L)	$Cmax_{pred}$ (mg/L)	Fold error
Nitsche and Mascher 1982 [11]	1000 mg IV (1)	1414.58	1295.70	0.92	113.00	90.52	0.80
	1000 mg IV (2)	987.50	1295.70	1.31	89.00	90.52	1.02
	1000 mg IV (3)	1534.83	1295.70	0.84	105.00	90.52	0.86
	1000 mg IV (4)	1184.62	1295.70	1.09	97.00	90.52	0.93
	1000 mg IV (5)	1495.13	1295.70	0.87	106.00	90.52	0.85
	1000 mg IV (6)	979.54	1295.70	1.32	98.00	90.52	0.92
Nitsche and Mascher 1982 [11]	1000 mg PO (1)	1190.00	1290.97	1.08	157.00	138.40	0.88
	1000 mg PO (2)	986.00	1290.97	1.31	124.00	138.40	1.12

	1000 mg PO (3)	924.50	1290.97	1.40	94.00	138.40	1.47
	1000 mg PO (4)	992.50	1290.97	1.30	111.00	138.40	1.25
	1000 mg PO (5)	1601.50	1290.97	0.81	159.00	138.40	0.87
	1000 mg PO (6)	984.00	1290.97	1.31	117.00	138.40	1.18
Nitsche and Mascher 1982 [11]	900 mg PO (1) A	867.00	1160.91	1.34	88.00	124.50	1.41
	900 mg PO (2) A	773.50	1160.91	1.50	91.00	124.50	1.37
	900 mg PO (3) A	828.50	1160.91	1.40	104.00	124.50	1.20
	900 mg PO (4) A	935.50	1160.91	1.24	99.00	124.50	1.26
	900 mg PO (5) A	1321.00	1160.91	0.88	127.00	124.50	0.98
	900 mg PO (6) A	840.00	1160.91	1.38	98.00	124.50	1.27
Nitsche and Mascher 1982 [11]	900 mg PO (1) B	1174.50	1160.93	0.99	129.00	124.50	0.97
	900 mg PO (2) B	1200.00	1160.93	0.97	143.00	124.50	0.87
	900 mg PO (3) B	538.00	1160.93	2.16	81.00	124.50	1.54
	900 mg PO (4) B	1059.50	1160.93	1.10	108.00	124.50	1.15
	900 mg PO (5) B	1188.50	1160.93	0.98	131.00	124.50	0.95
	900 mg PO (6) B	1041.00	1160.93	1.12	107.00	124.50	1.16

Table S9 Ratio between the predicted and observed pharmacokinetic parameters of valproic acid (VPA) in different dosing regimens used for model verification

Study ID/ Reference	Dose/ Route	AUC_{obs} (mg*h/L)	AUC_{pred} (mg*h/L)	Fold error	$Cmax_{obs}$ (mg/L)	$Cmax_{pred}$ (mg/L)	Fold error
Dulac 2005 SD [14]	500 mg SD	612.42	556.90	0.91	21.81	31.92	1.46
Dulac 2005 [14]	500 mg MD	764.60	563.24	0.74	69.42	60.57	0.87
Perucca 1978 [12]	800 mg IV (1)	1196.82	952.31	0.80	99.14	70.54	0.71
	800 mg IV (2)	1491.60	980.92	0.66	128.73	73.23	0.57
	800 mg IV (3)	1420.61	899.36	0.63	106.42	63.97	0.60
	800 mg IV (4)	1478.24	907.82	0.61	114.59	65.30	0.57

	800 mg IV (5)	1204.96	977.66	0.81	80.86	68.28	0.84
	800 mg IV (6)	1373.68	989.40	0.72	114.43	74.06	0.65
Perucca 1978 [12]	800 mg PO (1)	1254.37	939.86	0.75	74.82	59.18	0.79
	800 mg PO (2)	1452.10	968.36	0.67	90.00	61.92	0.69
	800 mg PO (3)	1389.36	886.63	0.64	90.00	52.13	0.58
	800 mg PO (4)	1511.41	895.22	0.59	88.40	53.56	0.61
	800 mg PO (5)	1196.36	918.25	0.77	62.43	56.75	0.91
	800 mg PO (6)	1623.69	976.79	0.60	94.93	62.66	0.66
Rha 1993 SD[15]	300 mg PO SD	429.69	445.84	1.04	22.40	25.05	1.12
Rha 1993 [15]	300 mg PO MD	945.51	777.87	0.82	85.77	73.95	0.86
Pokjarac 1993 [17]	900 mg/day MD	936.12	622.62	0.67	67.82	49.62	0.73
Ibarra 2013 [16]	500 mg female	813.93	736.88	0.91	49.62	42.69	0.86
Ibarra 2013 [16]	500 mg male	497.89	571.16	1.15	34.03	32.41	0.95
Chun 1980 [18]	250 mg SD 1A	378.67	281.96	0.74	27.90	17.75	0.64
Chun 1980 [18]	250 mg SD 1B (capsule)	433.50	277.37	0.64	27.00	17.90	0.66
Chun 1980 [18]	250 mg 2A (fasted)	385.45	266.55	0.69	24.40	17.26	0.71
Chun 1980 [18]	250 mg 2B (fed)	381.70	278.81	0.73	24.20	15.68	0.65
Chun 1980 [18]	250 mg 3A (fed)	368.27	264.62	0.72	18.60	16.01	0.86
Chun 1980 [18]	250 mg 3B (fed)	381.65	264.52	0.69	20.90	15.29	0.73
Chun 1980 [18]	250 mg 3C (syrup)	454.63	281.07	0.62	33.40	17.42	0.52
Ohdo 1992 [19]	800 mg 1A (evening; heavy meal)	1259.54	944.34	0.75	65.22	53.13	0.81

Ohdo 1992 [19]	800 mg 1B (morning; light meal)	1294.02	948.56	0.73	76.76	56.60	0.74
Ohdo 1992 [19]	800 mg 2A (evening; light meal)	931.49	803.22	0.86	74.44	57.30	0.77
Ohdo 1992 [19]	800 mg 2B (morning; light meal)	899.86	799.02	0.89	69.41	56.35	0.81
Georgoff 2018 [13]	15 mg/kg IV	1554.45	1383.03	0.89	115.96	84.35	0.73
Georgoff 2018 [13]	30 mg/kg IV	2675.99	2614.66	0.98	243.34	164.97	0.68
Georgoff 2018 [13]	60 mg/kg IV	2783.90	4954.50	1.78	255.39	322.83	1.26
Georgoff 2018 [13]	90 mg/kg IV	5488.38	8039.95	1.46	535.92	500.47	0.93
Georgoff 2018 [13]	120 mg/kg IV	4715.63	10764.13	2.28	544.63	665.17	1.22
Georgoff 2018 [13]	130 mg/kg IV	9907.04	12275.61	1.24	841.47	735.13	0.87
Georgoff 2018 [13]	140 mg/kg IV	12602.84	12963.53	1.03	1199.46	784.15	0.65
Georgoff 2018 [13]	150 mg/kg IV	7720.91	13042.40	1.69	682.44	820.23	1.20

4.3 Concentration-time profiles

Simulated versus observed concentration-time profiles of all data listed in section 3.2.2 are presented below. The original.pksim5 are provided in Supplemented material 3.

4.3.1 Model building

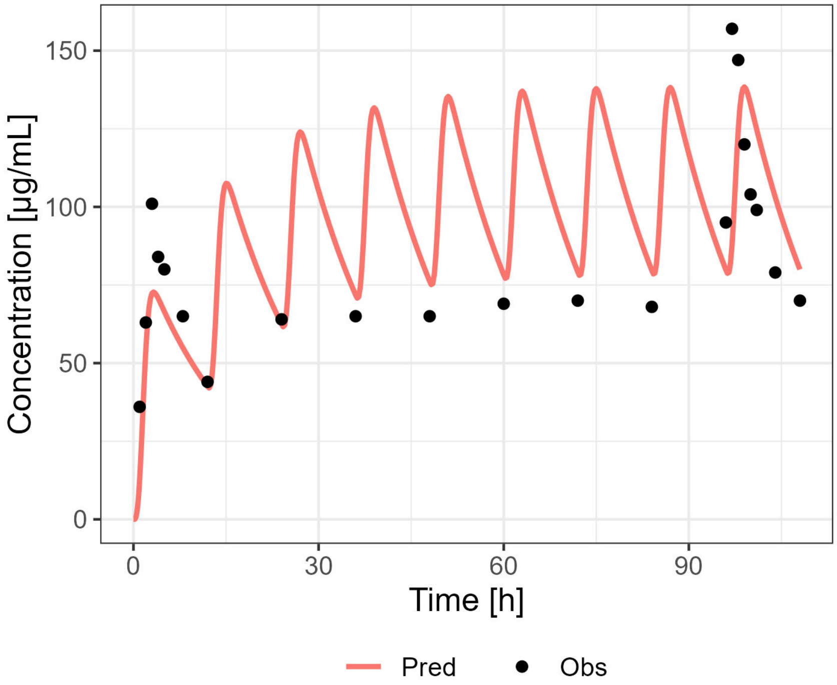

Figure S4 Predicted (Pred) versus observed (Obs) concentration-time profile after administration of 1000 mg PO MD [11]

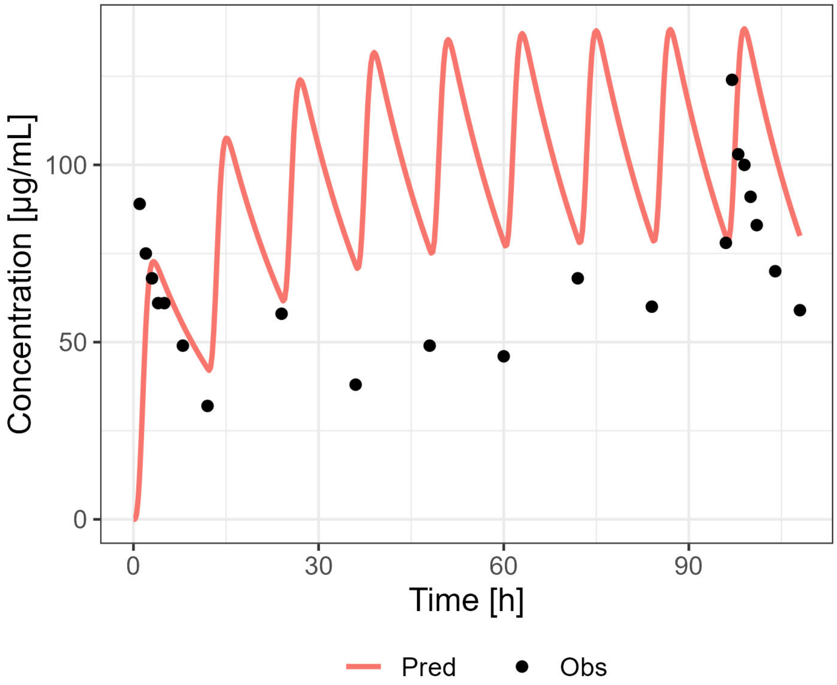

Figure S5 Predicted (Pred) versus observed (Obs) concentration-time profile after administration of 1000 mg PO MD [11]

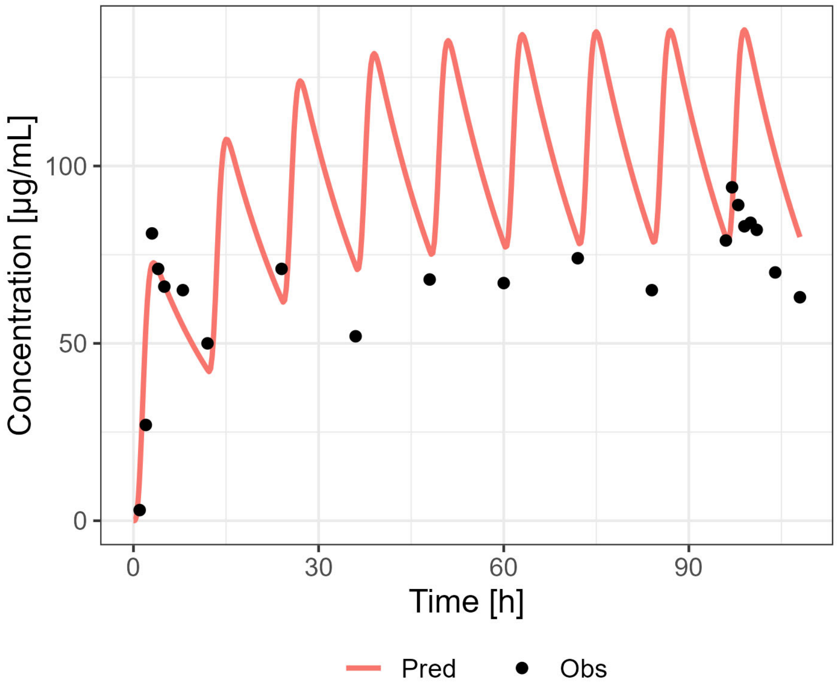

Figure S6 Predicted (Pred) versus observed (Obs) concentration-time profile after administration of 1000 mg PO MD [11]

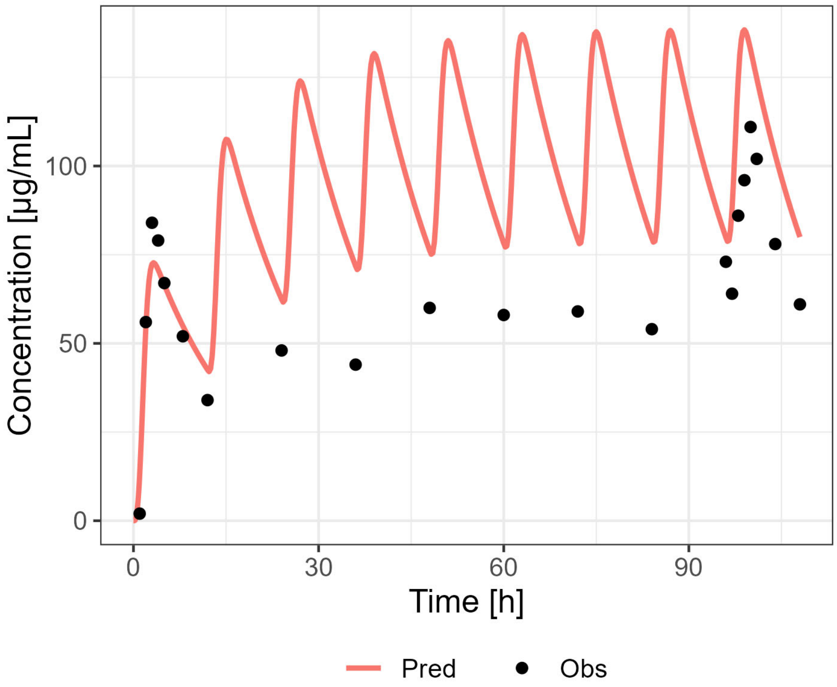

Figure S7 Predicted (Pred) versus observed (Obs) concentration-time profile after administration of 1000 mg PO MD [11]

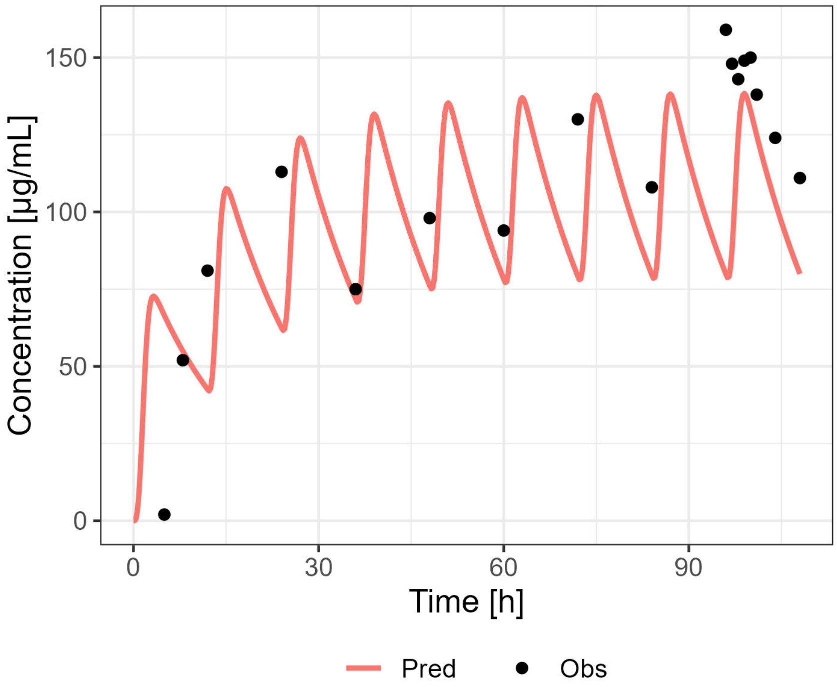

Figure S8 Predicted (Pred) versus observed (Obs) concentration-time profile after administration of 1000 mg PO MD [11]

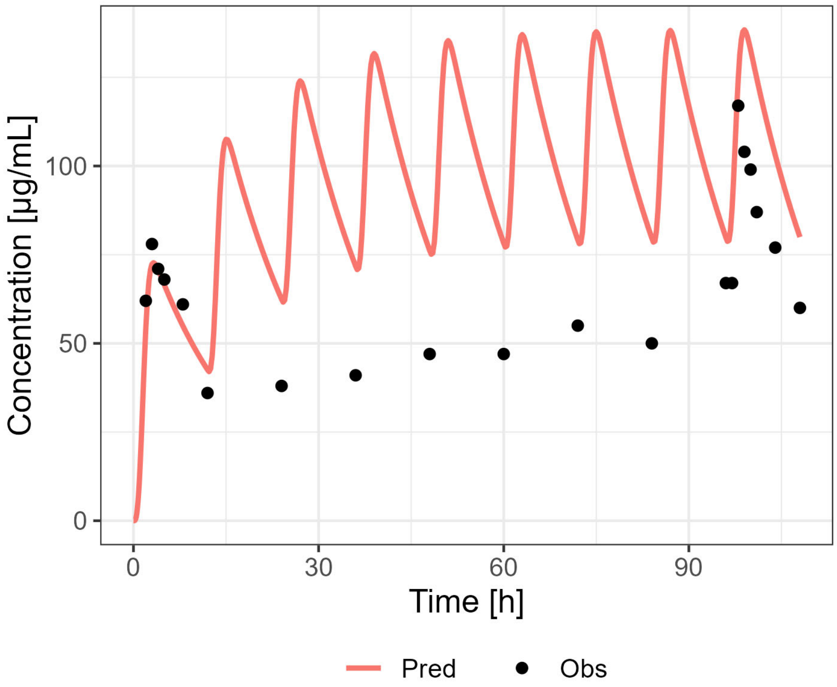

Figure S9 Predicted (Pred) versus observed (Obs) concentration-time profile after administration of 1000 mg PO MD [11]

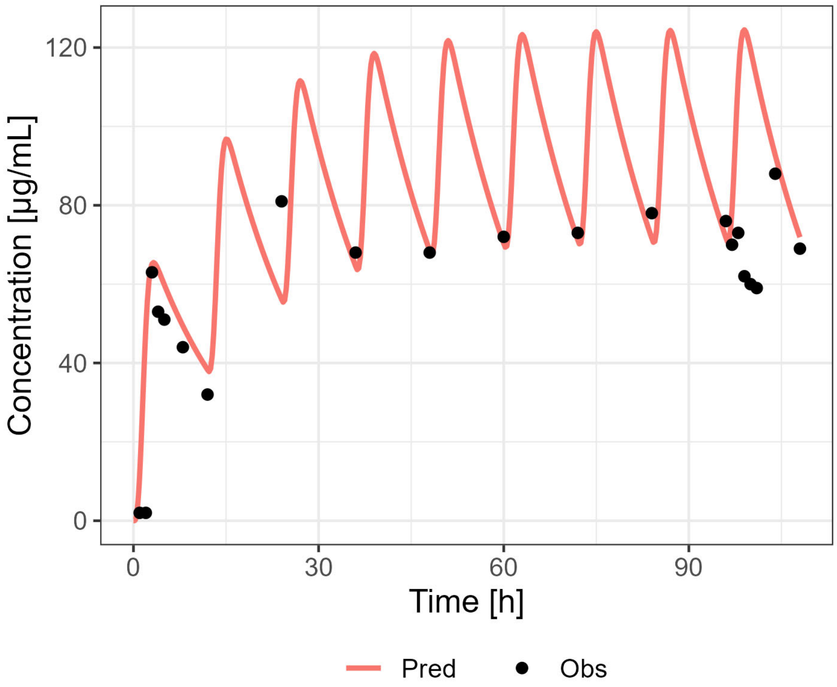

Figure S10 Predicted (Pred) versus observed (Obs) concentration-time profile after administration of 900 mg (2x450 mg) PO MD [11]

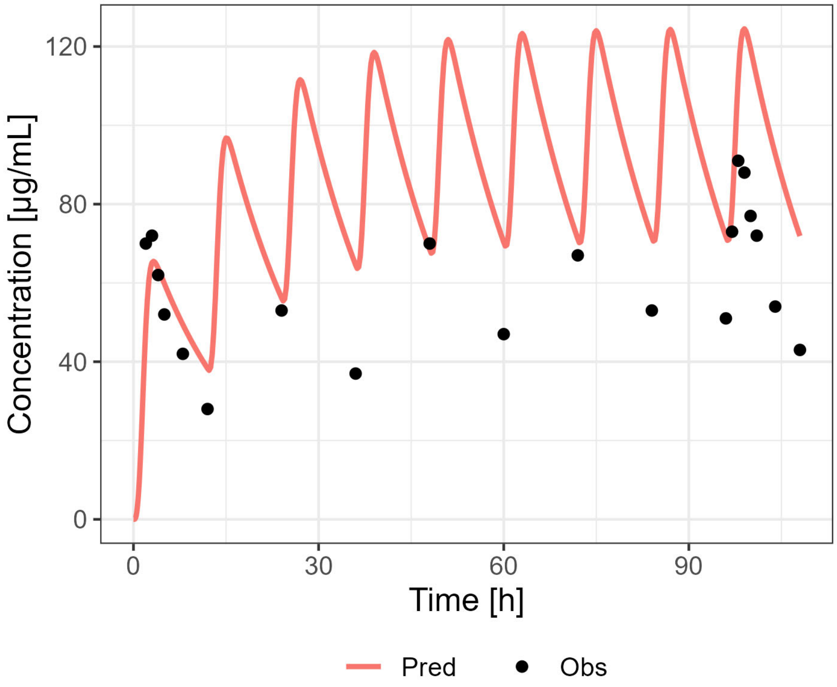

Figure S11 Predicted (Pred) versus observed (Obs) concentration-time profile after administration of 900 mg (2x450 mg) PO MD [11]

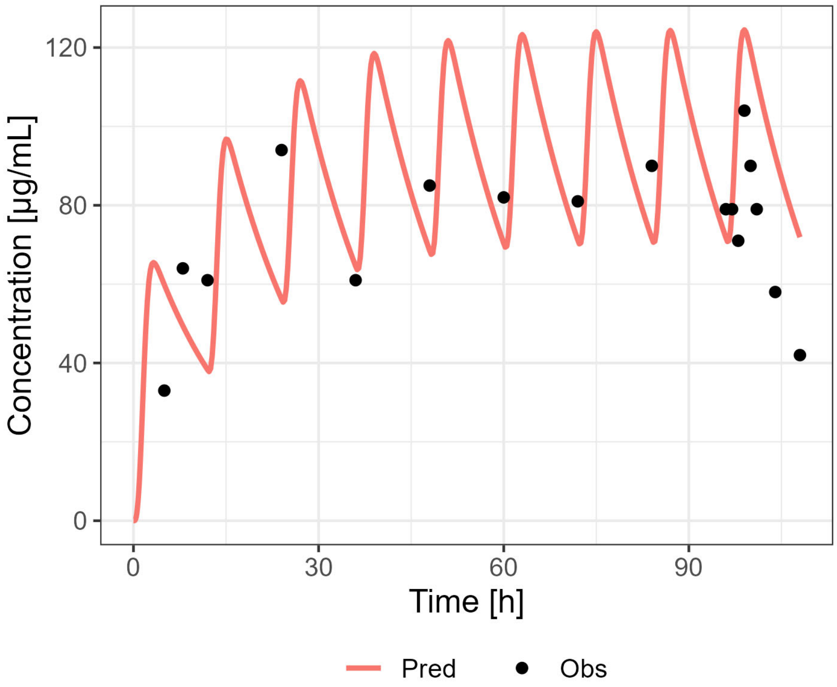

Figure S12 Predicted (Pred) versus observed (Obs) concentration-time profile after administration of 900 mg (2x450 mg) PO MD [11]

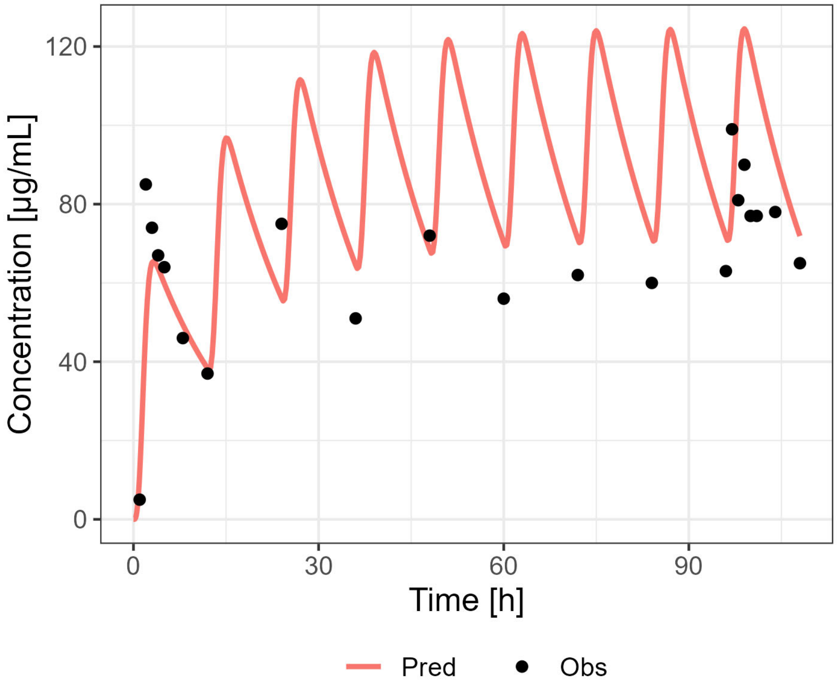

Figure S13 Predicted (Pred) versus observed (Obs) concentration-time profile after administration of 900 mg (2x450 mg) PO MD [11]

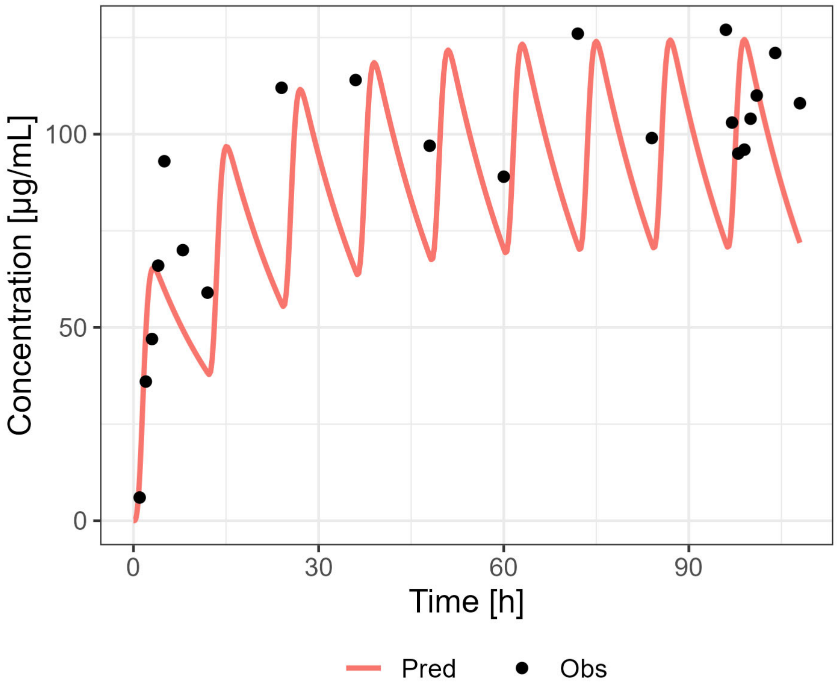

Figure S14 Predicted (Pred) versus observed (Obs) concentration-time profile after administration of 900 mg (2x450 mg) PO MD [11]

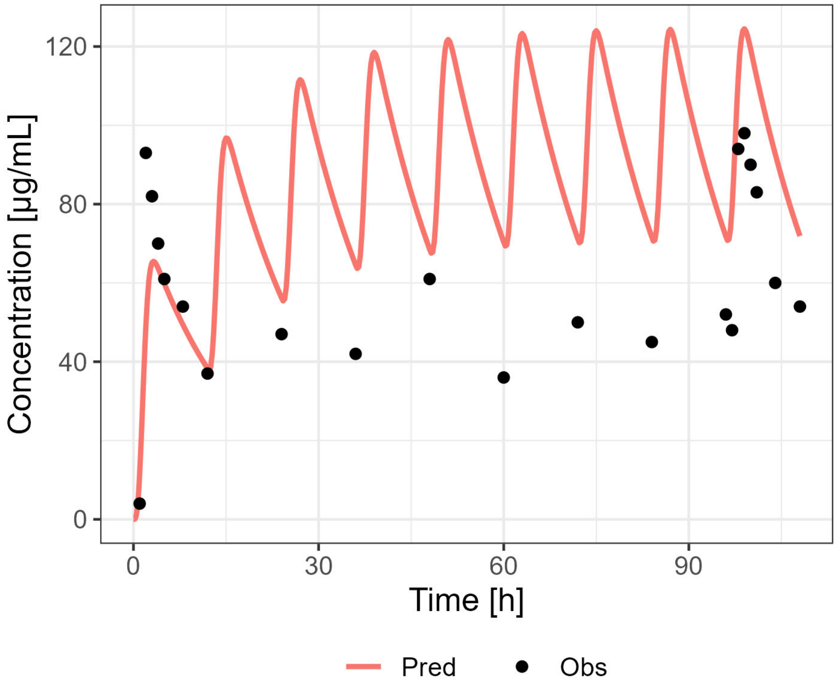

Figure S15 Predicted (Pred) versus observed (Obs) concentration-time profile after administration of 900 mg (2x450 mg) PO MD [11]

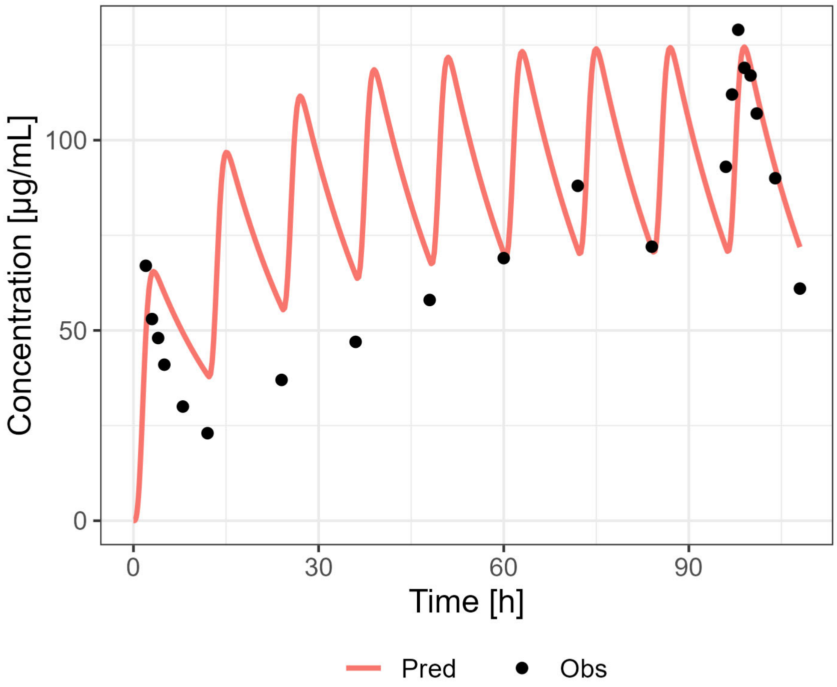

Figure S16 Predicted (Pred) versus observed (Obs) concentration-time profile after administration of 900 mg (3x300 mg) PO MD [11]

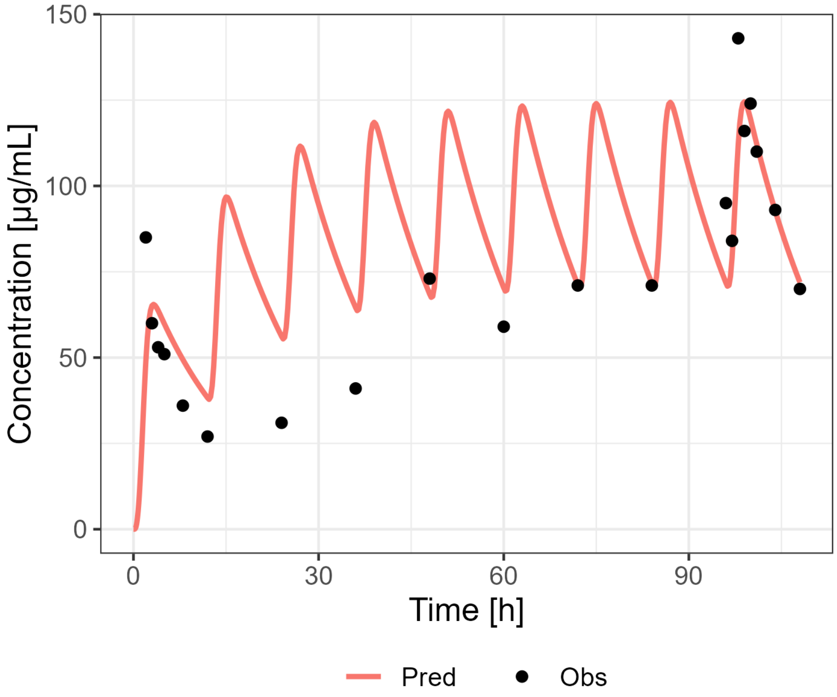

Figure S17 Predicted (Pred) versus observed (Obs) concentration-time profile after administration of 900 mg (3x300 mg) PO MD [11]

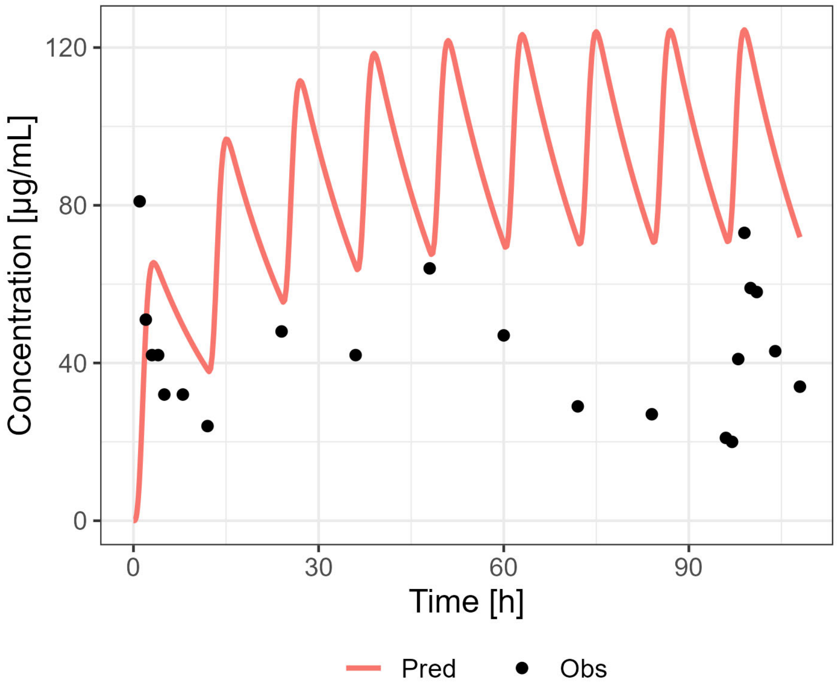

Figure S18 Predicted (Pred) versus observed (Obs) concentration-time profile after administration of 900 mg (3x300 mg) PO MD [11]

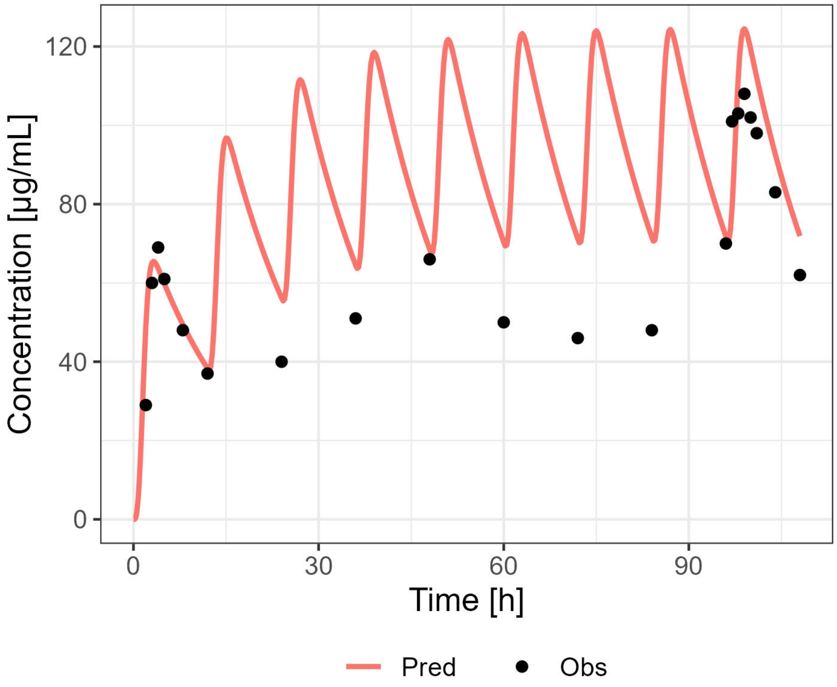

Figure S19 Predicted (Pred) versus observed (Obs) concentration-time profile after administration of 900 mg (3x300 mg) PO MD [11]

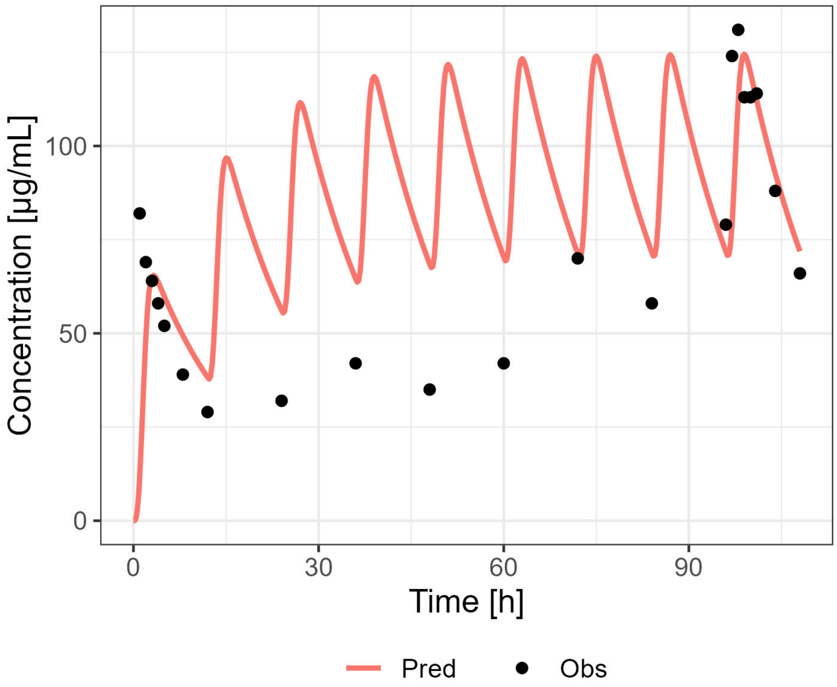

Figure S20 Predicted (Pred) versus observed (Obs) concentration-time profile after administration of 900 mg (3x300 mg) PO MD [11]

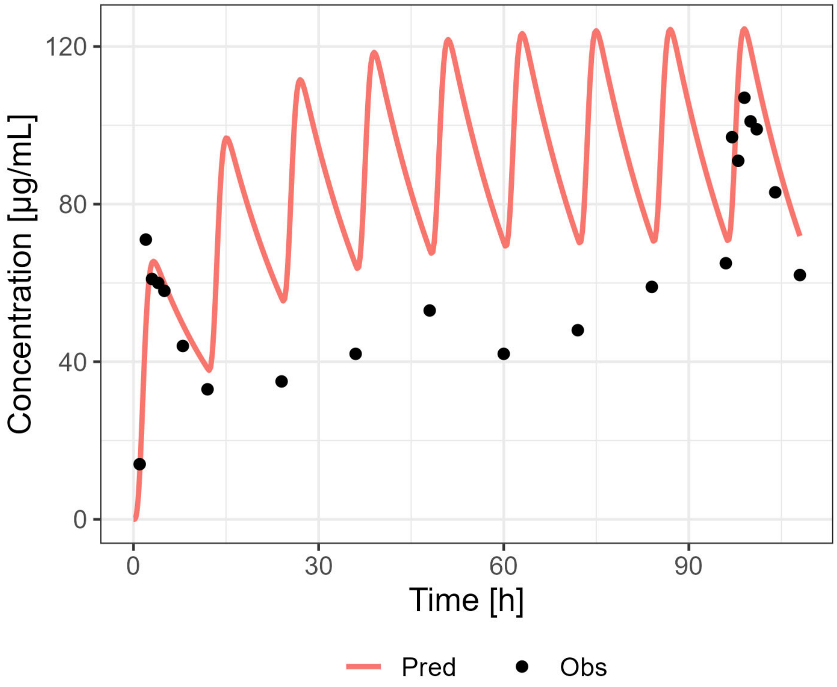

Figure S21 Predicted (Pred) versus observed (Obs) concentration-time profile after administration of 900 mg (3x300 mg) PO MD [11]

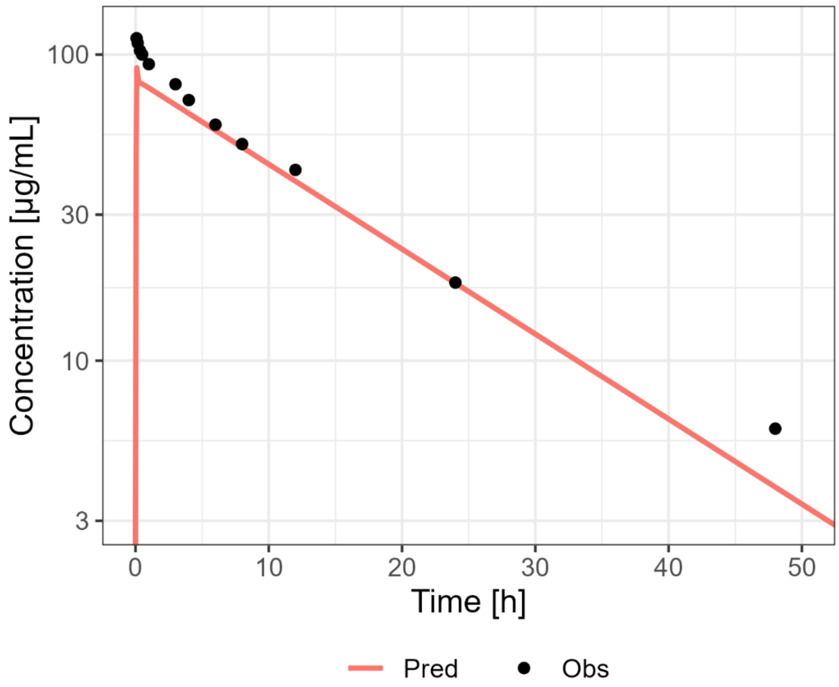

Figure S22 Predicted (Pred) versus observed (Obs) concentration-time profile after administration of 1000 mg IV [11]

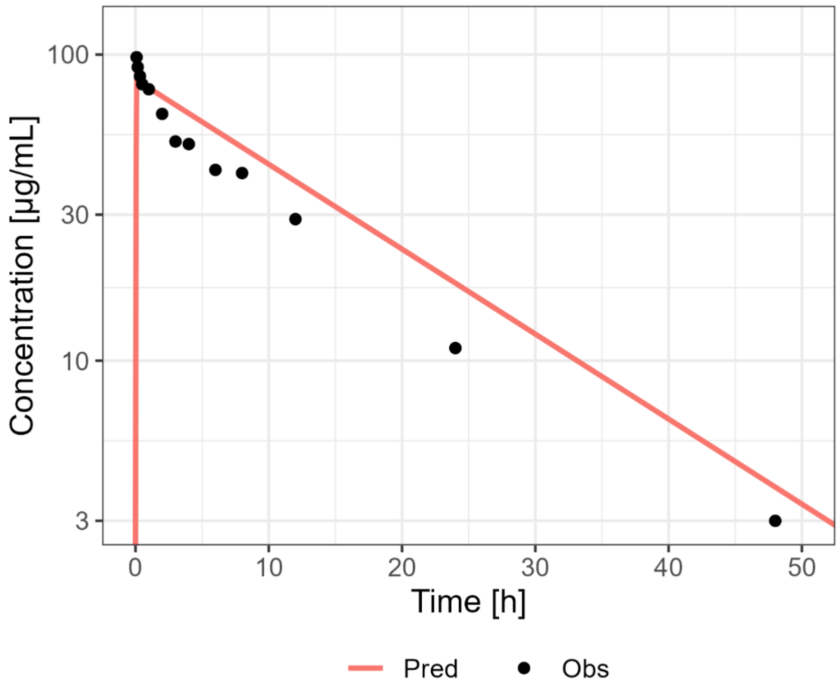

Figure S23 Predicted (Pred) versus observed (Obs) concentration-time profile after administration of 1000 mg IV [11]

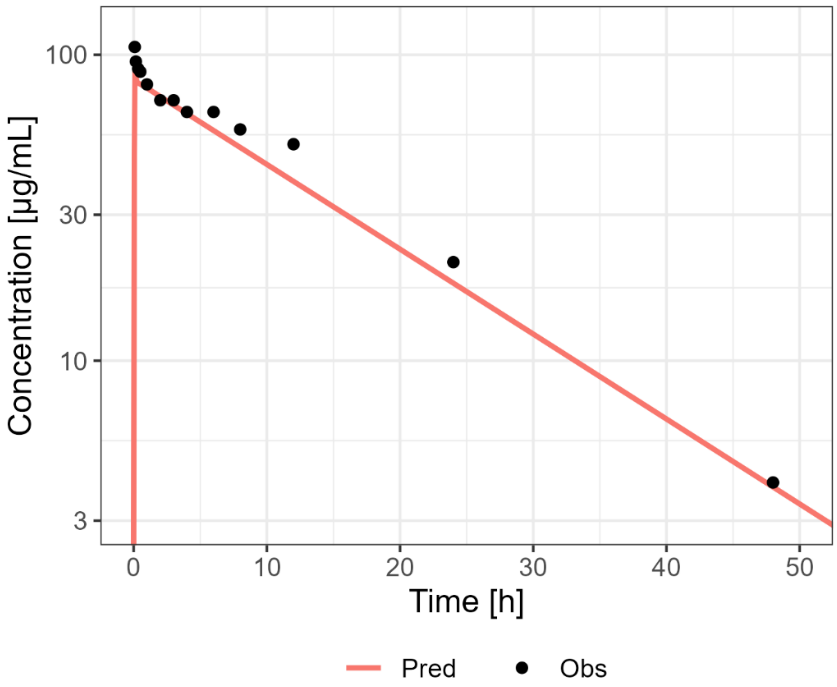

Figure S24 Predicted (Pred) versus observed (Obs) concentration-time profile after administration of 1000 mg IV [11]

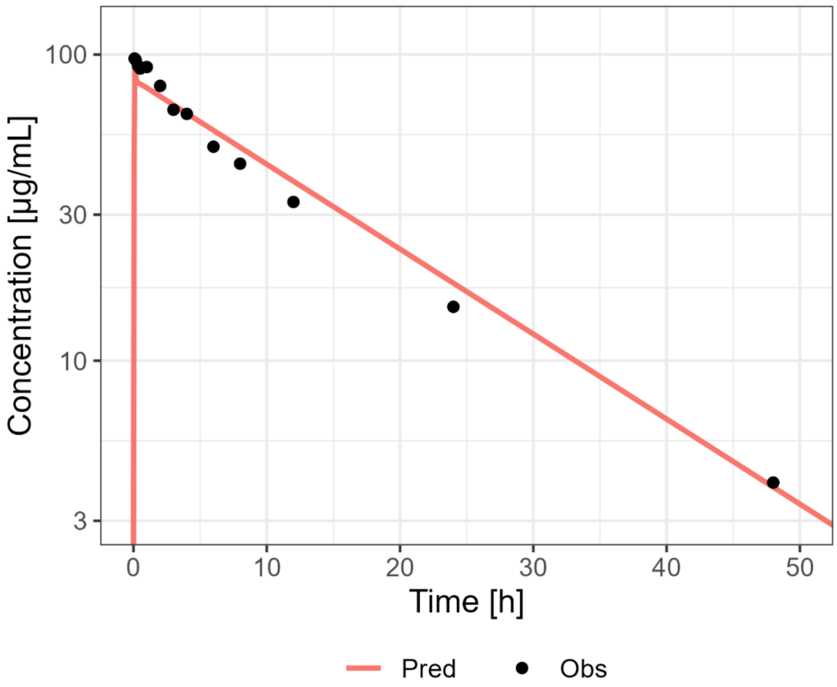

Figure S25 Predicted (Pred) versus observed (Obs) concentration-time profile after administration of 1000 mg IV [11]

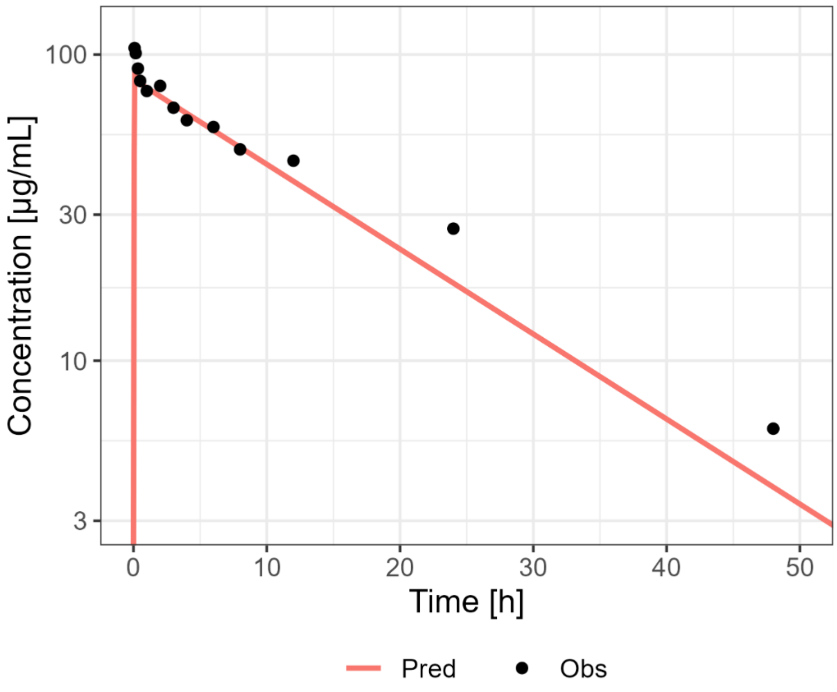

Figure S26 Predicted (Pred) versus observed (Obs) concentration-time profile after administration of 1000 mg IV [11]

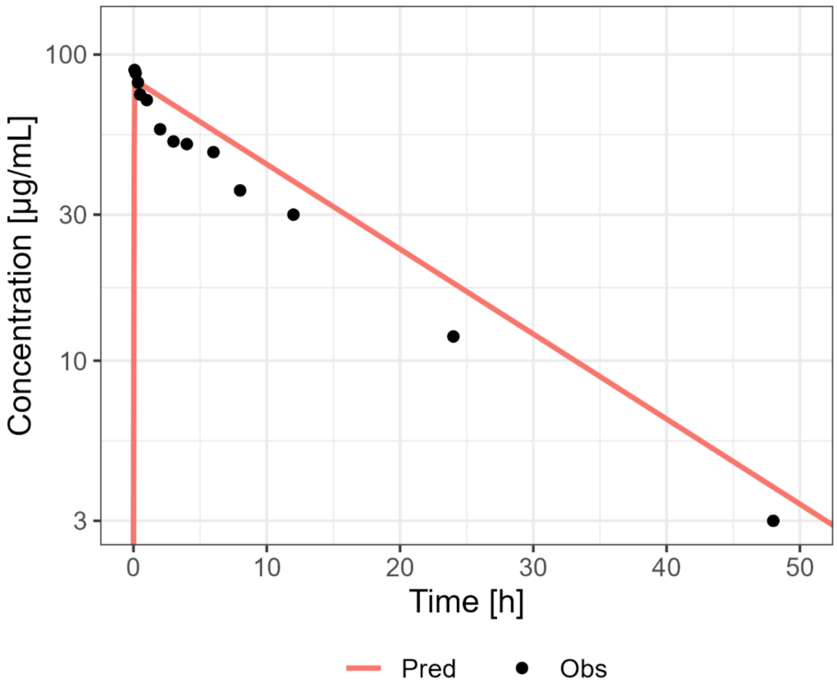

Figure S27 Predicted (Pred) versus observed (Obs) concentration-time profile after administration of 1000 mg IV [11]

4.3.2 Model verification

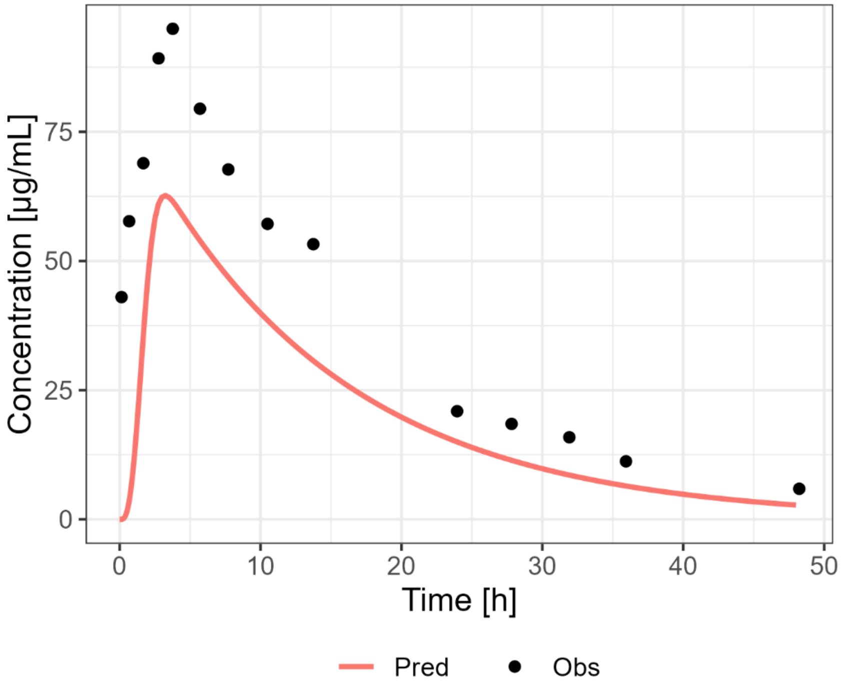

Figure S28 Predicted (Pred) versus observed (Obs) concentration-time profile after administration of 800 mg PO [12]

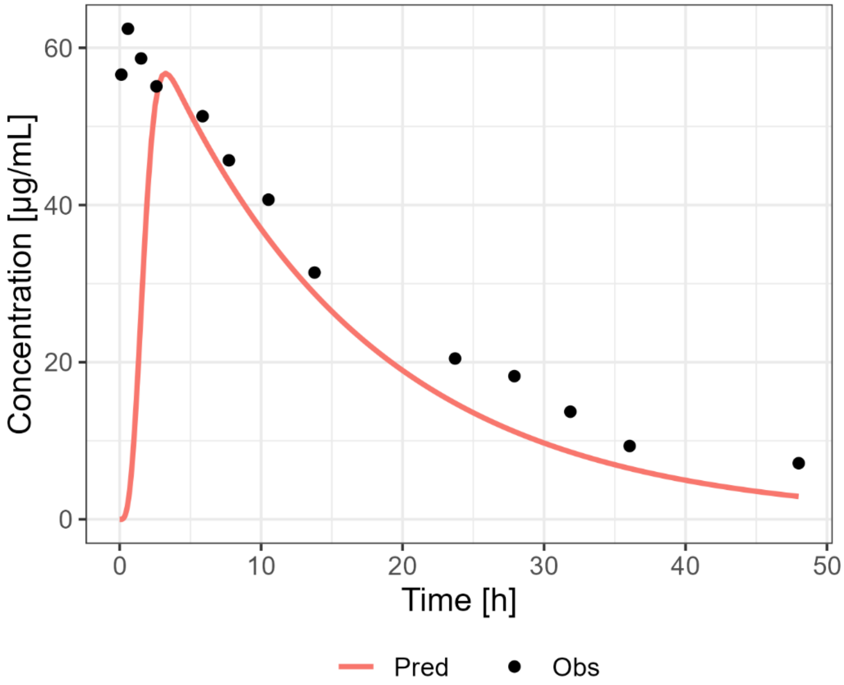

Figure S29 Predicted (Pred) versus observed (Obs) concentration-time profile after administration of 800 mg PO [12]

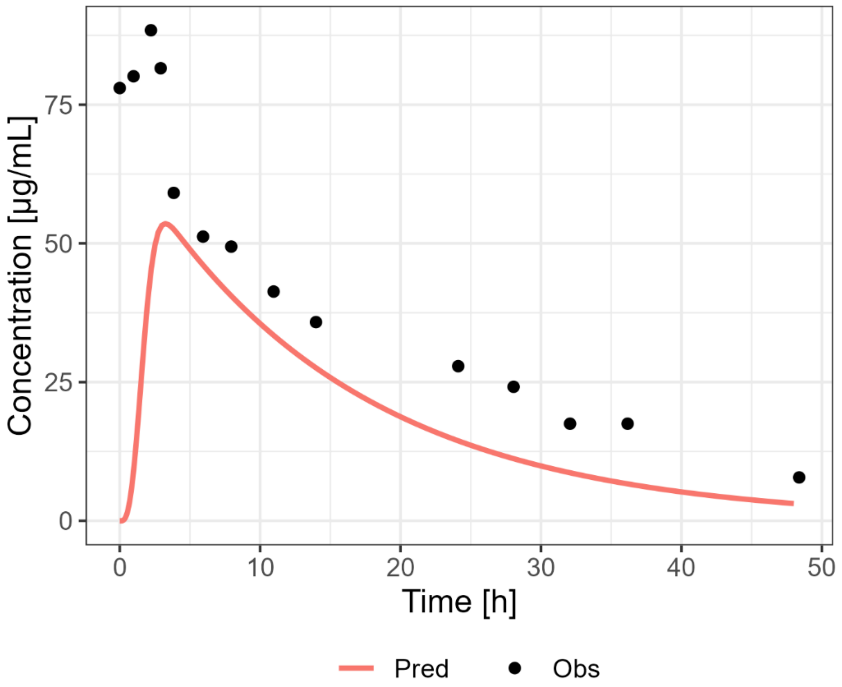

Figure S30 Predicted (Pred) versus observed (Obs) concentration-time profile after administration of 800 mg PO [12]

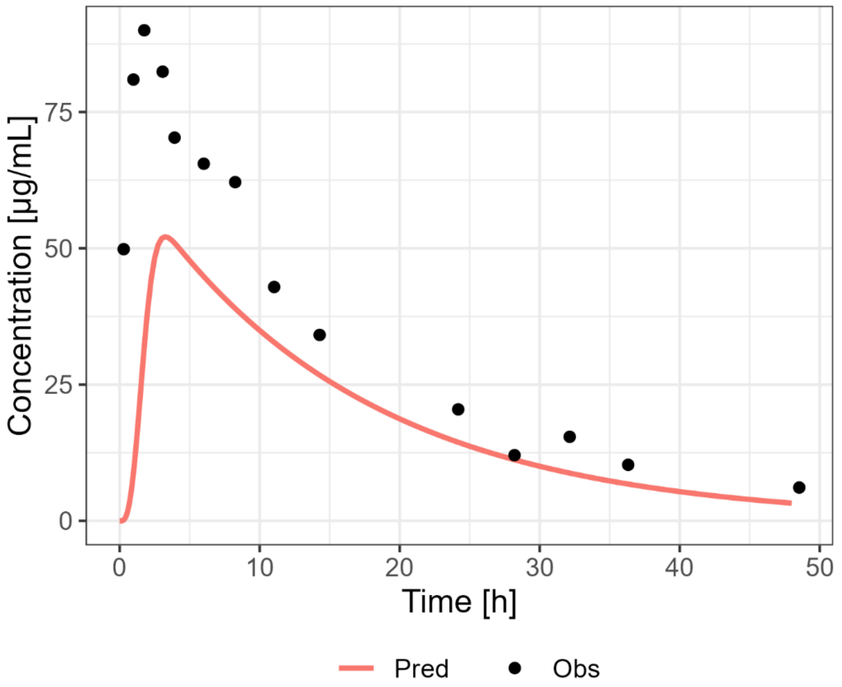

Figure S31 Predicted (Pred) versus observed (Obs) concentration-time profile after administration of 800 mg PO [12]

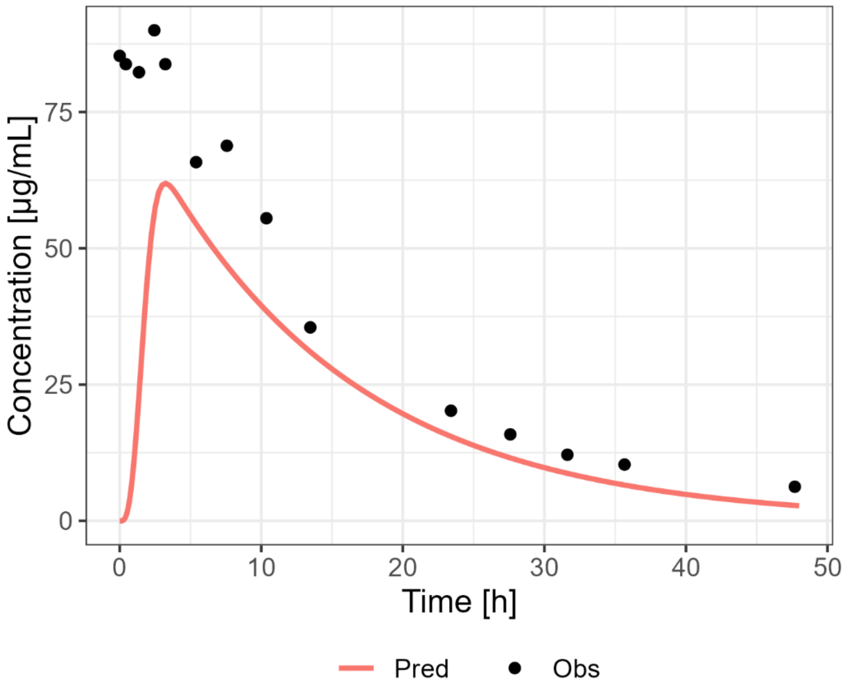

Figure S32 Predicted (Pred) versus observed (Obs) concentration-time profile after administration of 800 mg PO [12]

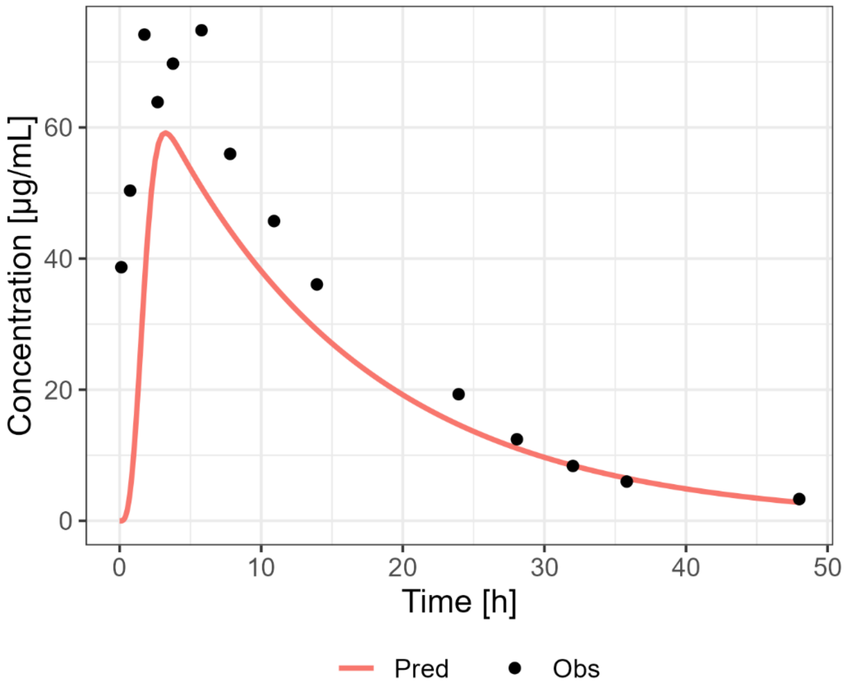

Figure S33 Predicted (Pred) versus observed (Obs) concentration-time profile after administration of 800 mg PO [12]

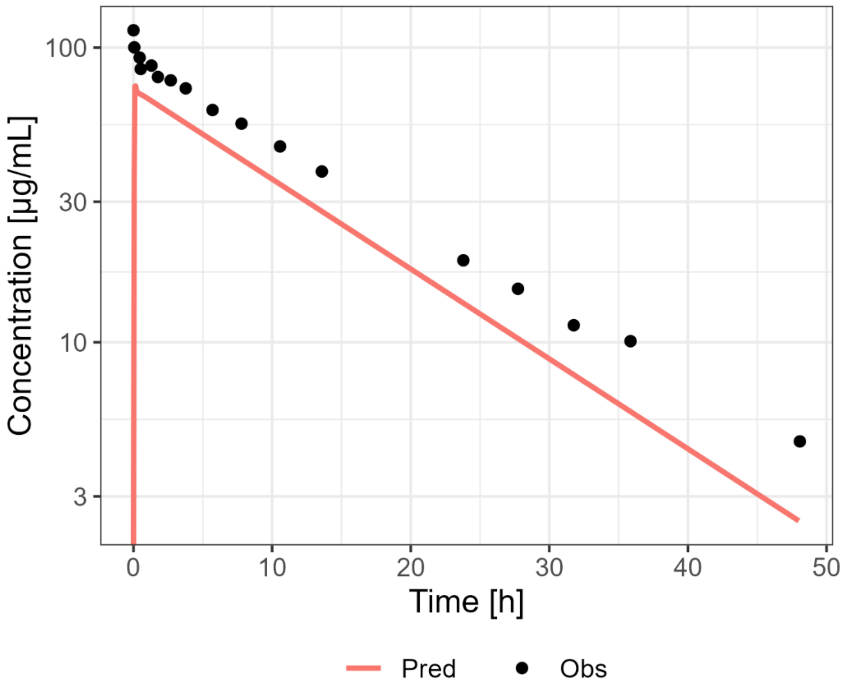

Figure S34 Predicted (Pred) versus observed (Obs) concentration-time profile after administration of 800 mg IV [12]

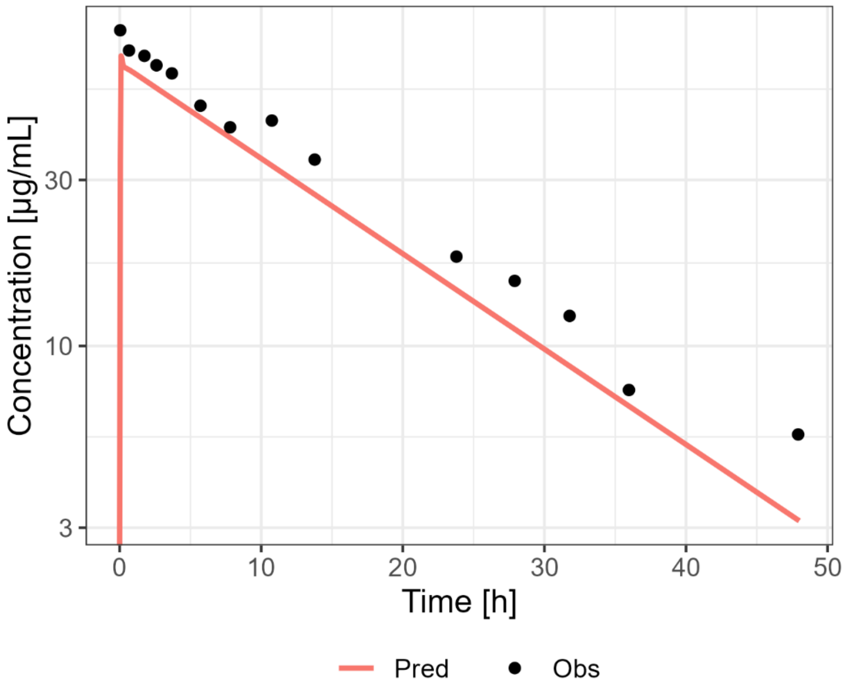

Figure S35 Predicted (Pred) versus observed (Obs) concentration-time profile after administration of 800 mg IV [12]

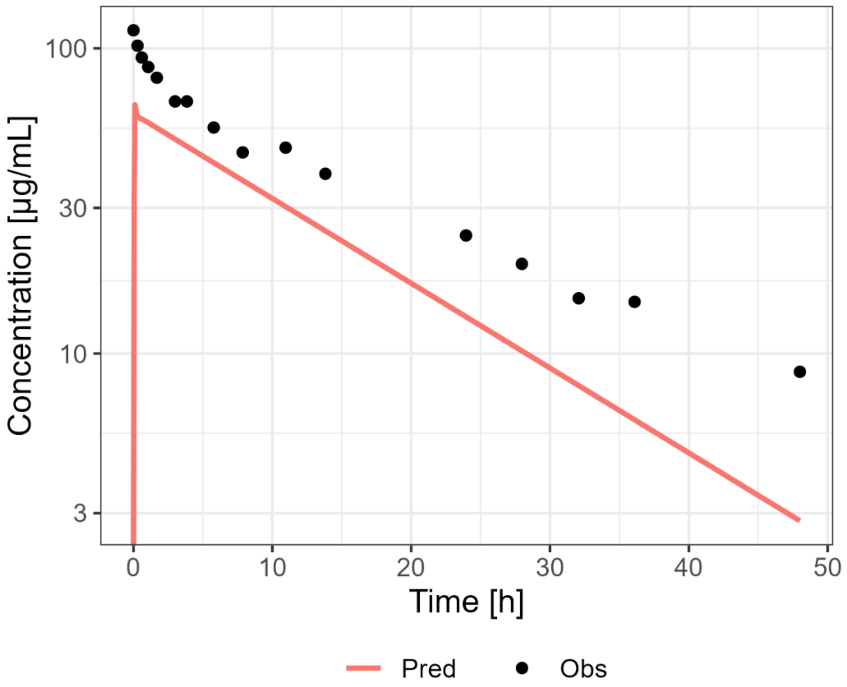

Figure S36 Predicted (Pred) versus observed (Obs) concentration-time profile after administration of 800 mg IV [12]

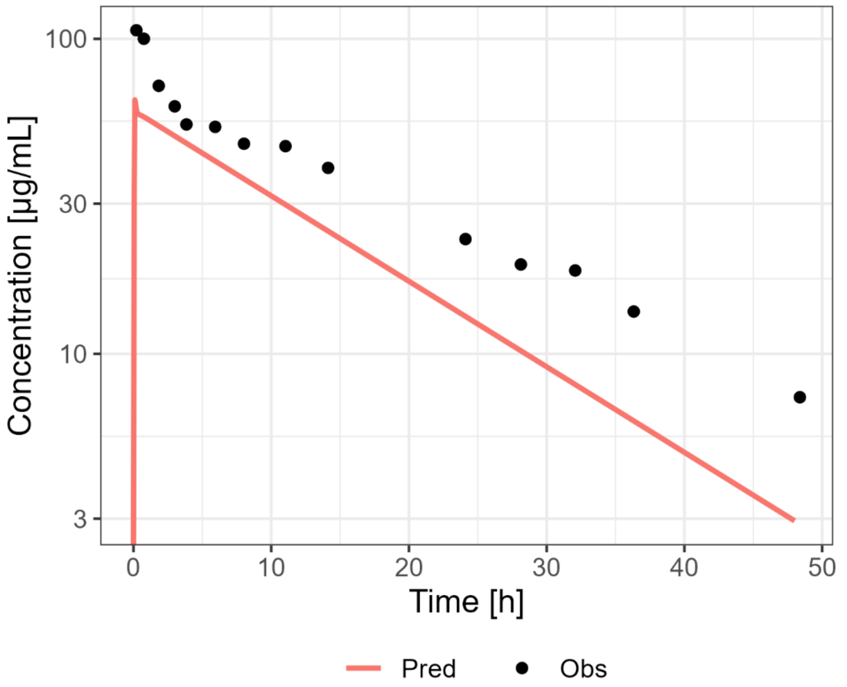

Figure S37 Predicted (Pred) versus observed (Obs) concentration-time profile after administration of 800 mg IV [12]

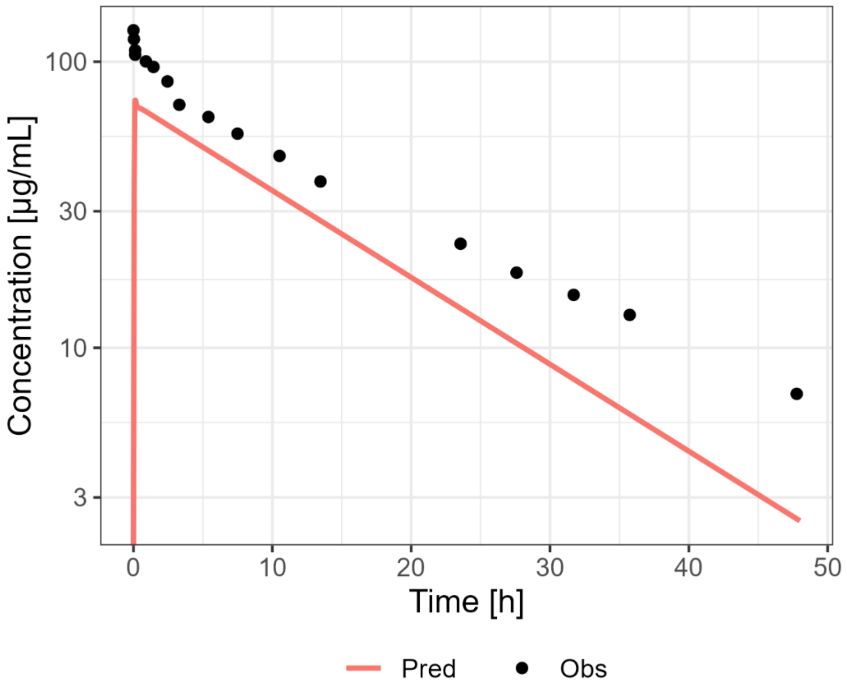

Figure S38 Predicted (Pred) versus observed (Obs) concentration-time profile after administration of 800 mg IV [12]

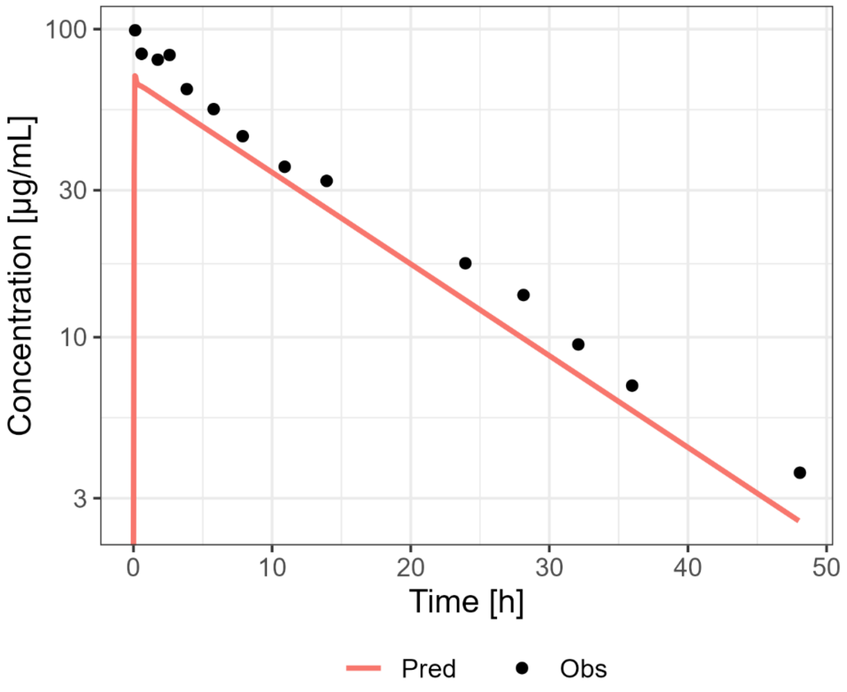

Figure S39 Predicted (Pred) versus observed (Obs) concentration-time profile after administration of 800 mg IV [12]

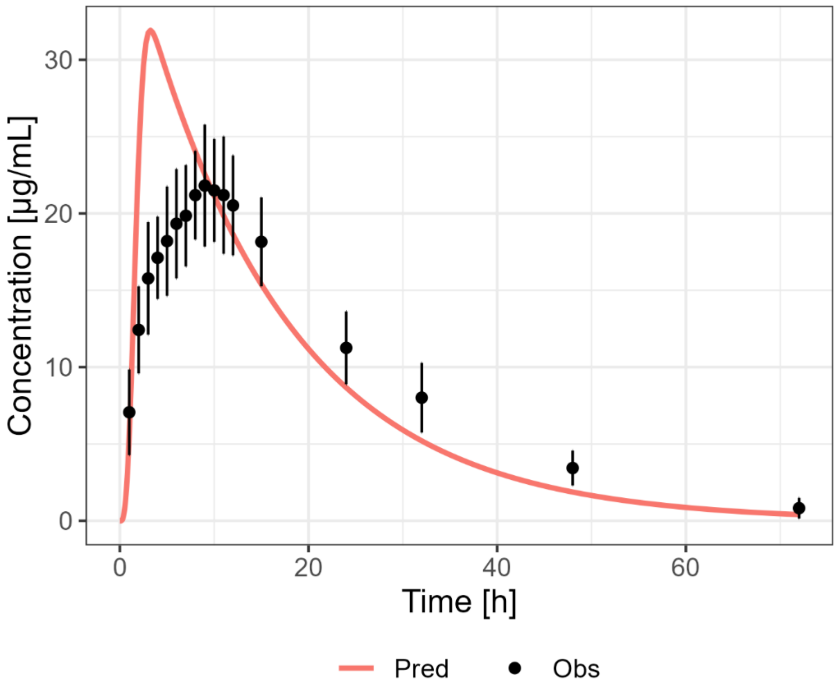

Figure S40 Predicted (Pred) versus observed (Obs) concentration-time profile after administration of 500 mg PO SD [14]

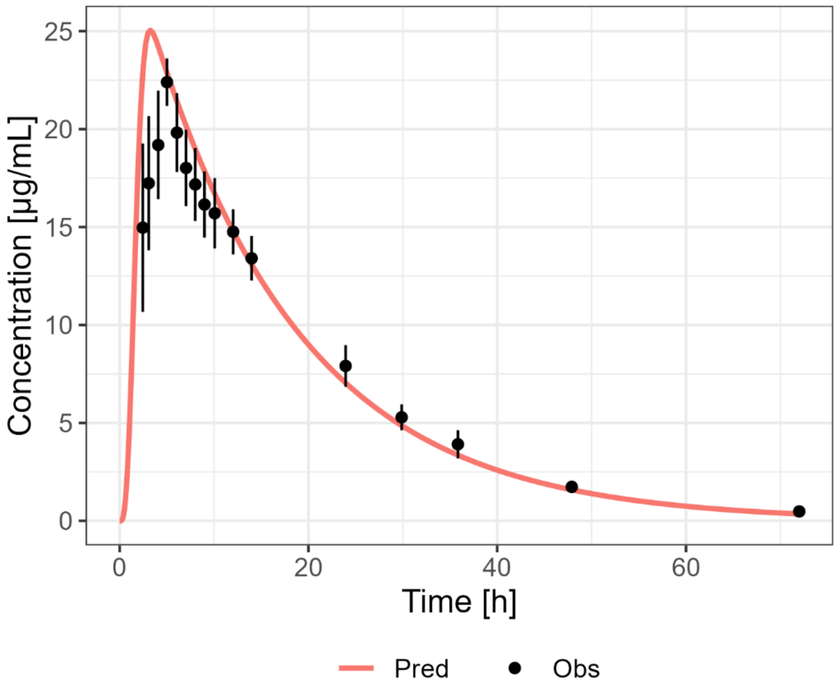

Figure S41 Predicted (Pred) versus observed (Obs) concentration-time profile after administration of 300 mg PO SD [15]

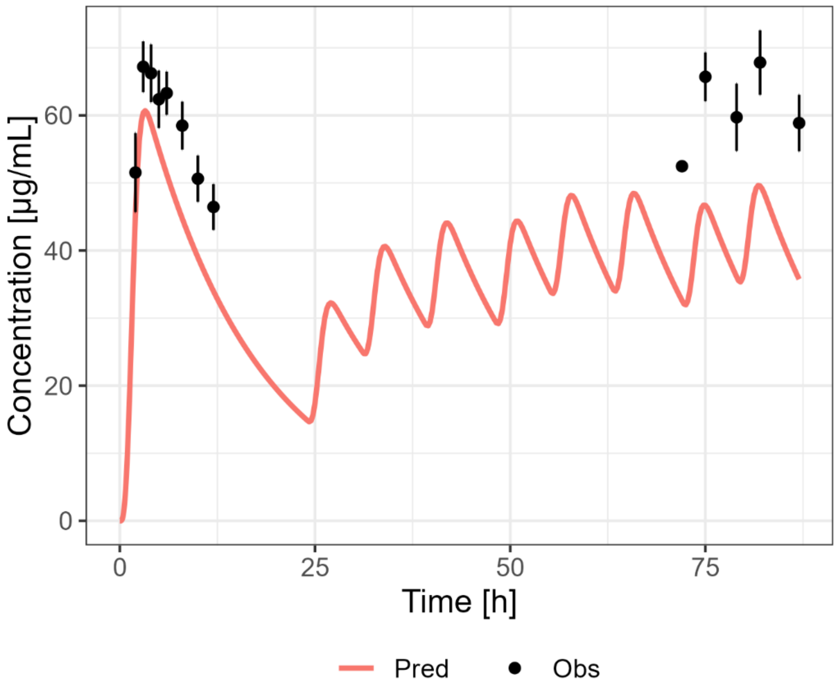

Figure S42 Predicted (Pred) versus observed (Obs) concentration-time profile after administration of 900 mg/day PO MD [17]

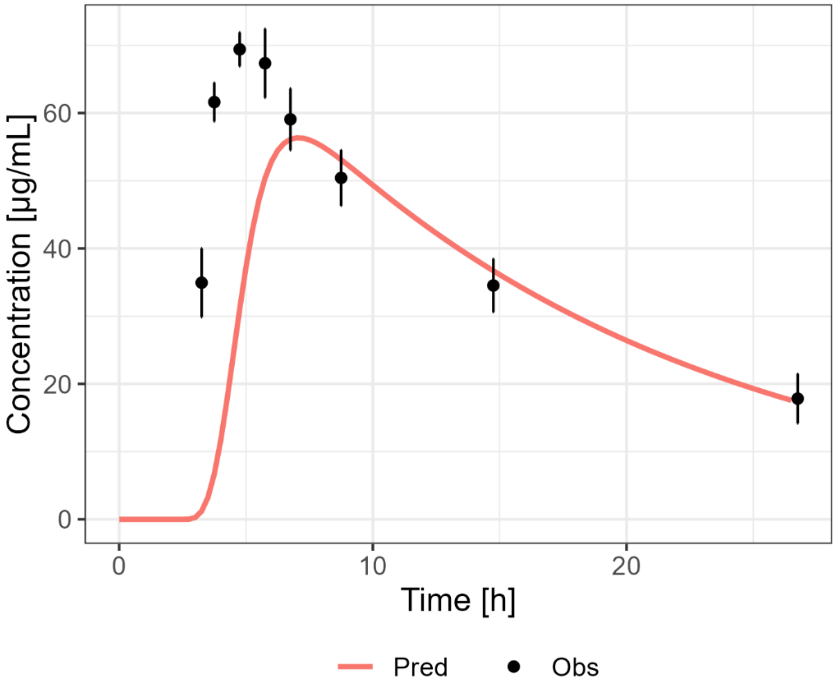

Figure S43 Predicted (Pred) versus observed (Obs) concentration-time profile after administration of 800 mg PO [19]

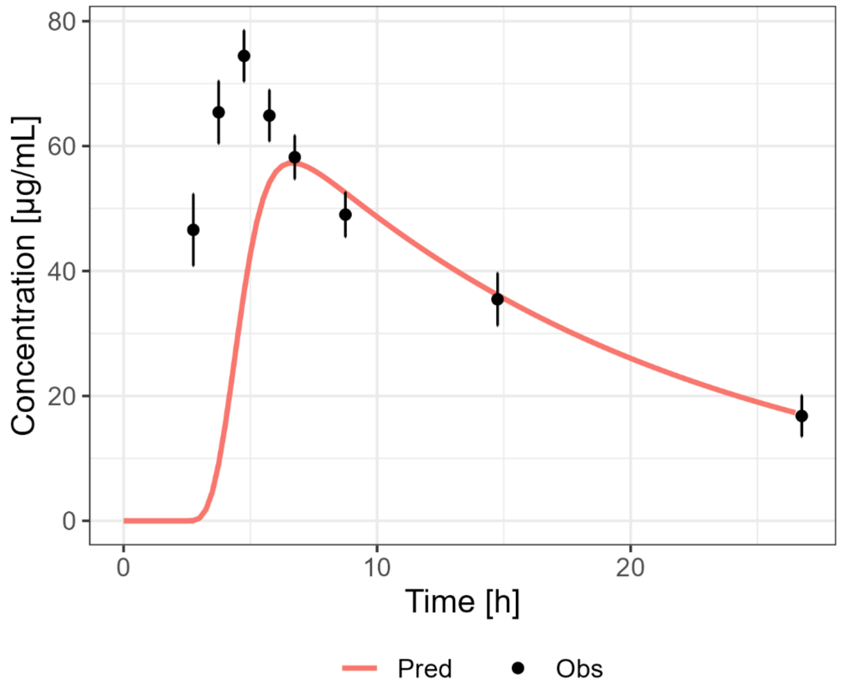

Figure S44 Predicted (Pred) versus observed (Obs) concentration-time profile after administration of 800 mg PO [19]

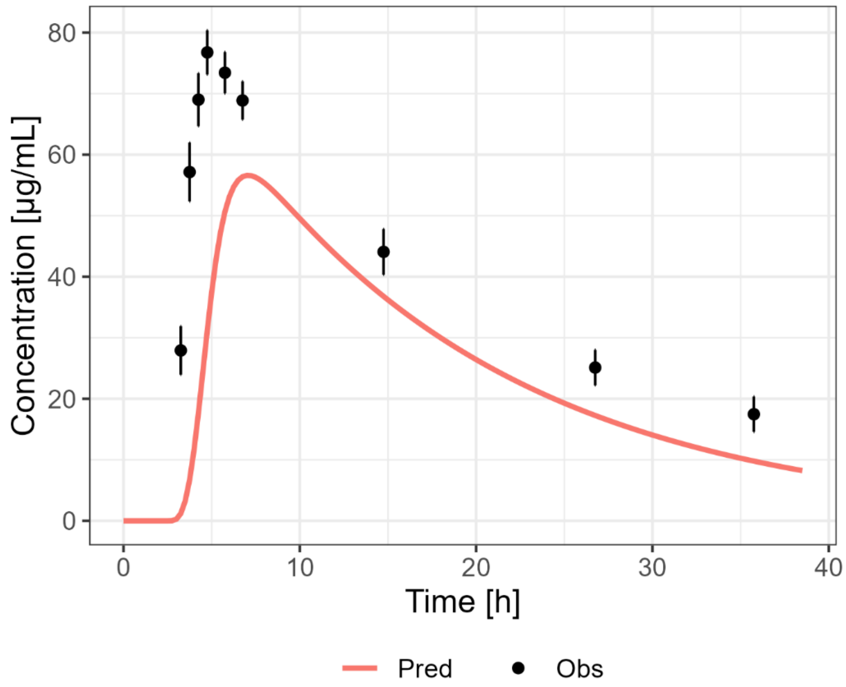

Figure S45 Predicted (Pred) versus observed (Obs) concentration-time profile after administration of 800 mg PO [19]

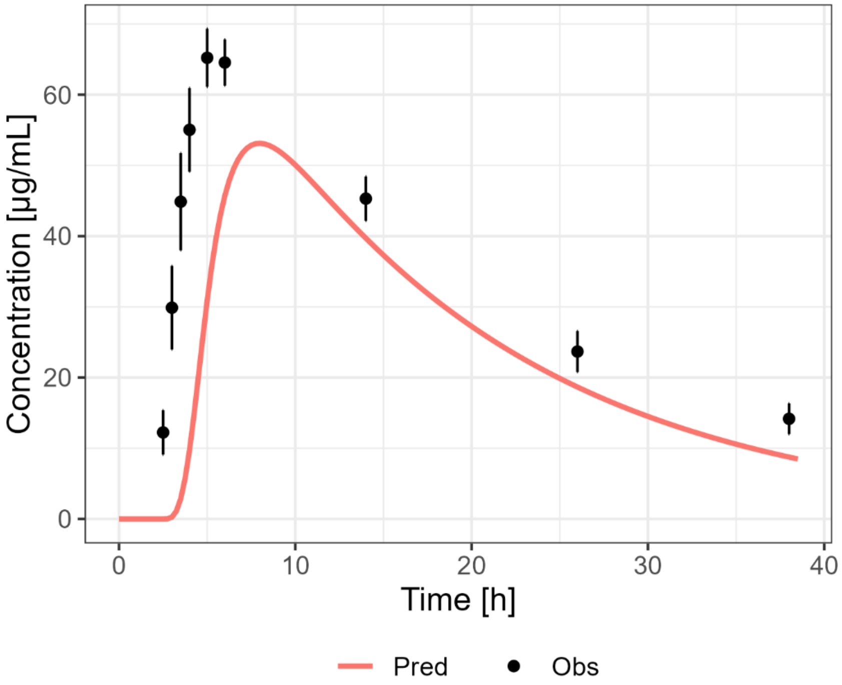

Figure S46 Predicted (Pred) versus observed (Obs) concentration-time profile after administration of 800 mg PO [19]

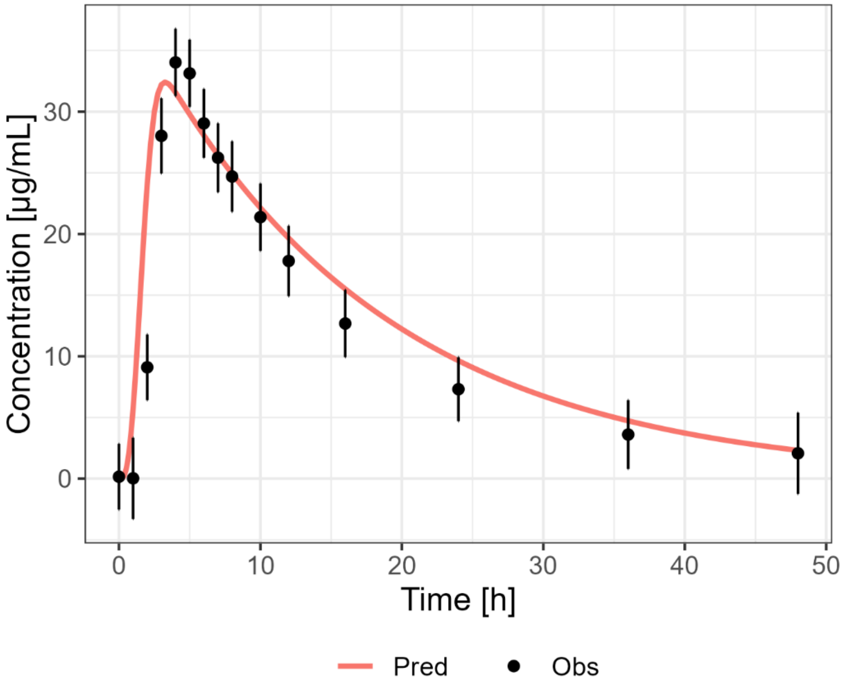

Figure S47 Predicted (Pred) versus observed (Obs) concentration-time profile after administration of 500 mg PO male [16]

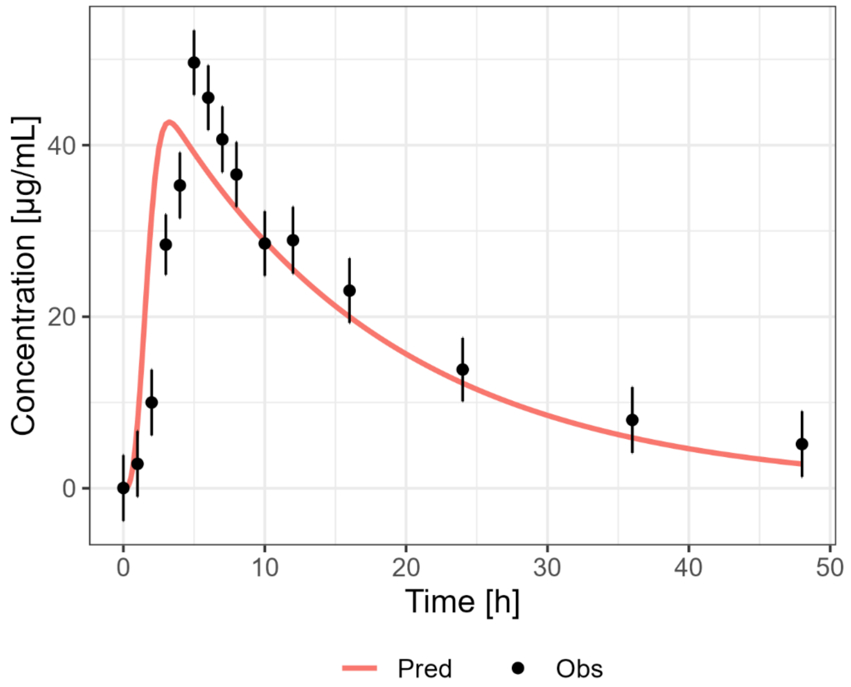

Figure S48 Predicted (Pred) versus observed (Obs) concentration-time profile after administration of 500 mg PO female [16]

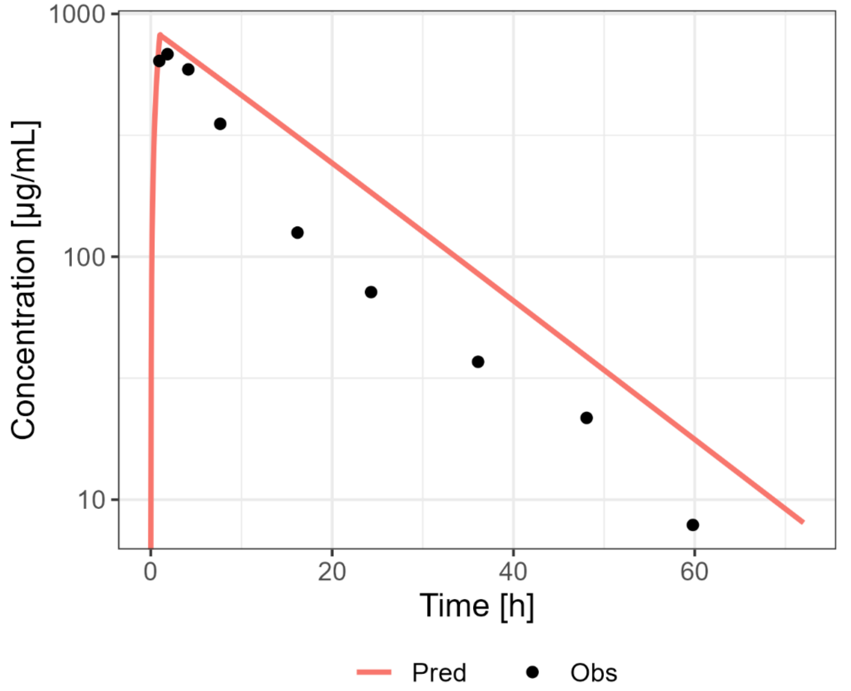

Figure S49 Predicted (Pred) versus observed (Obs) concentration-time profile after administration of 150 mg/kg IV [13]

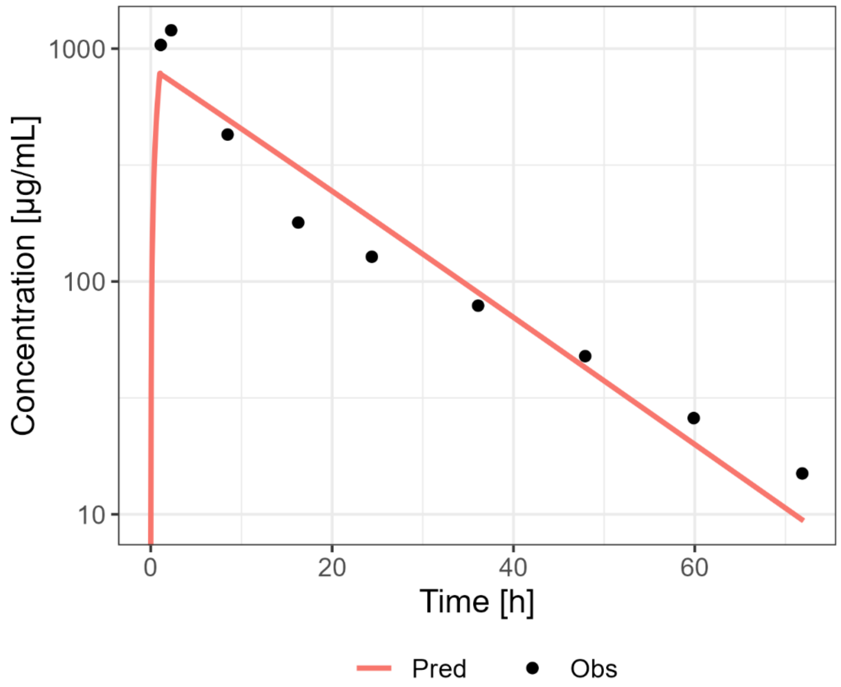

Figure S50 Predicted (Pred) versus observed (Obs) concentration-time profile after administration of 140 mg/kg IV [13]

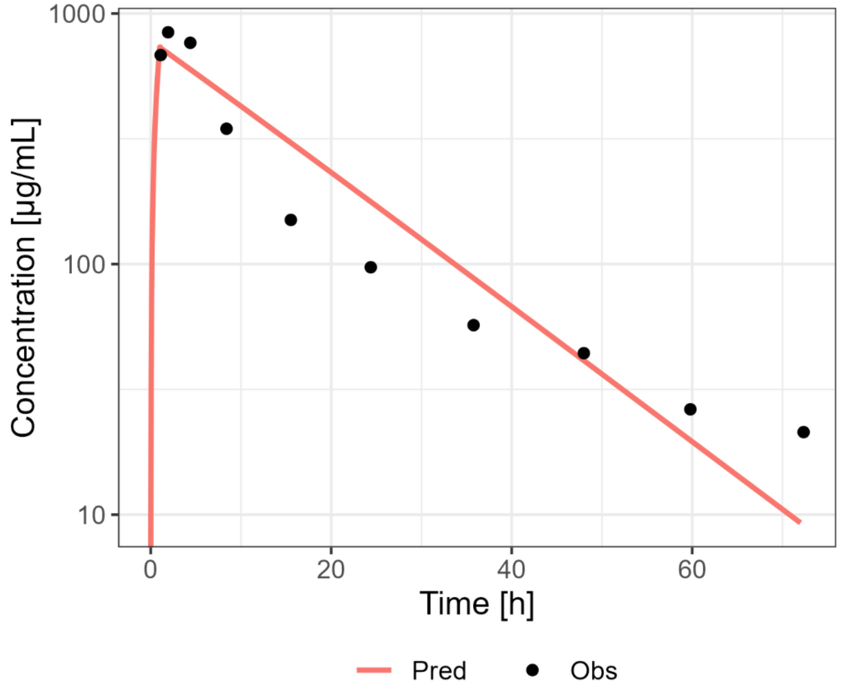

Figure S51 Predicted (Pred) versus observed (Obs) concentration-time profile after administration of 130 mg/kg IV [13]

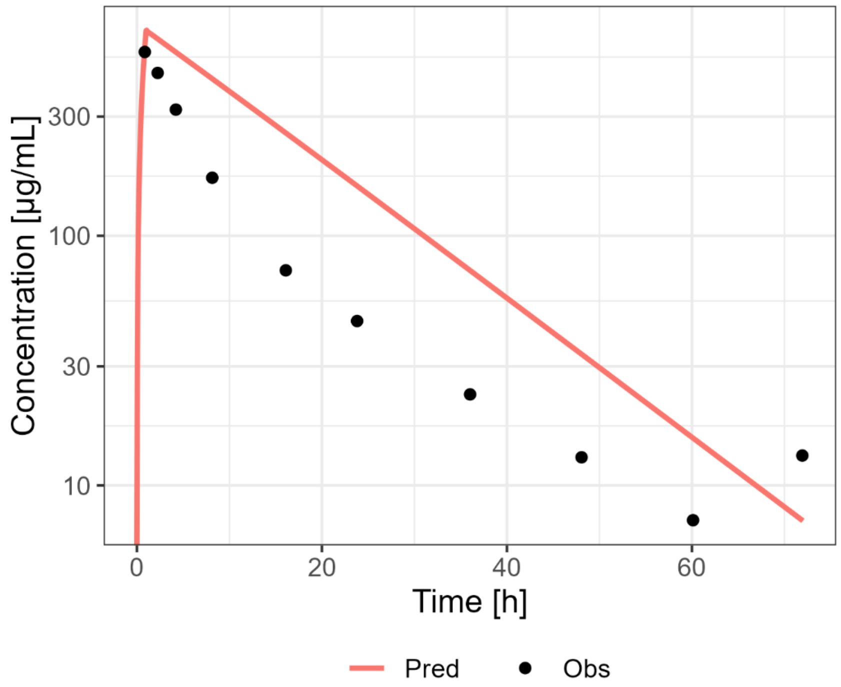

Figure S52 Predicted (Pred) versus observed (Obs) concentration-time profile after administration of 120 mg/kg IV [13]

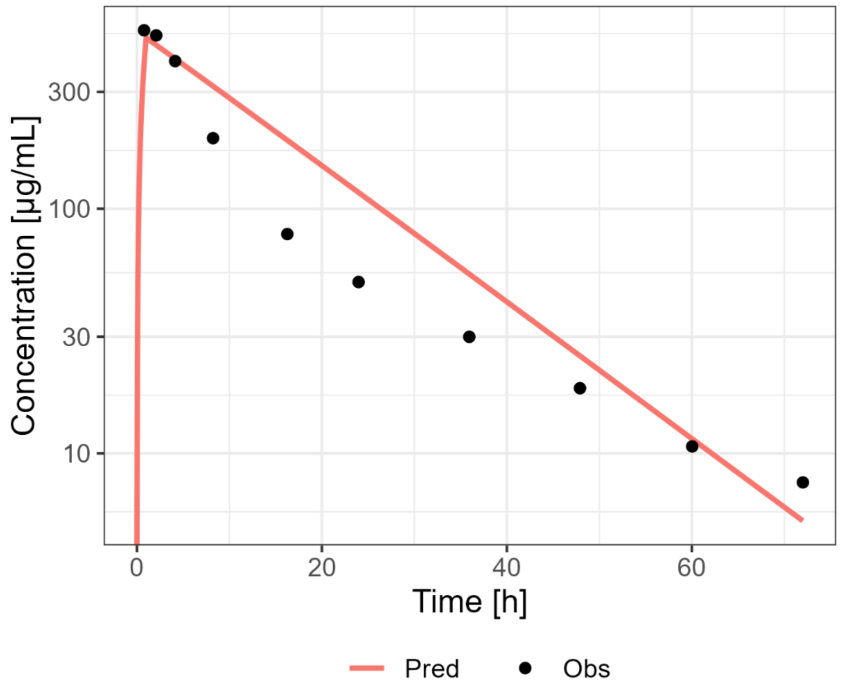

Figure S53 Predicted (Pred) versus observed (Obs) concentration-time profile after administration of 90 mg/kg IV [13]

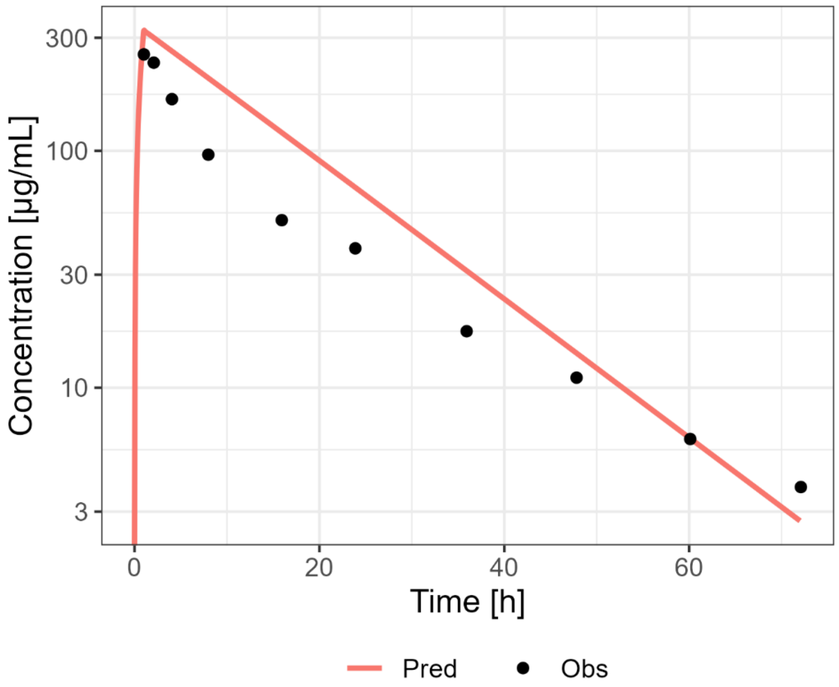

Figure S54 Predicted (Pred) versus observed (Obs) concentration-time profile after administration of 60 mg/kg IV [13]

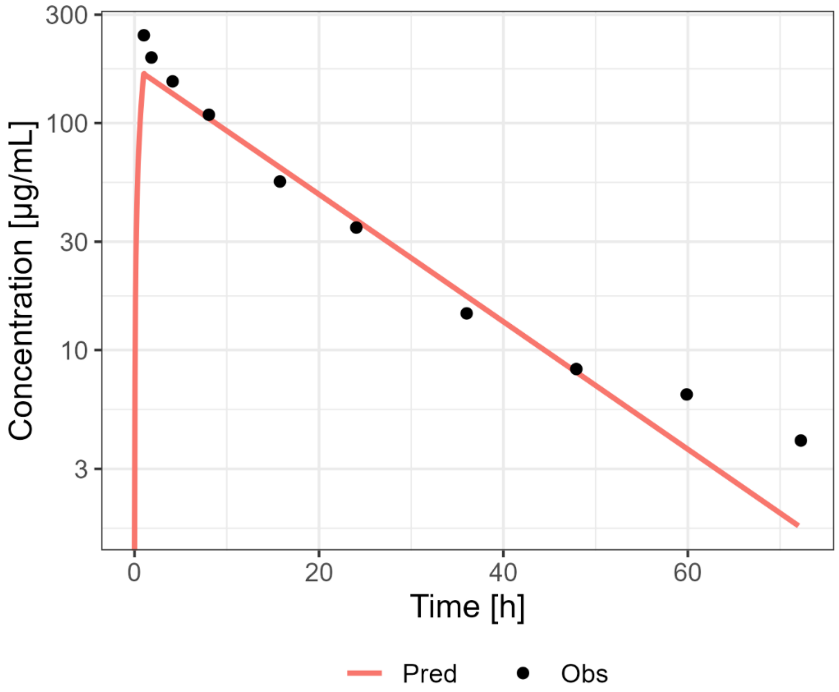

Figure S55 Predicted (Pred) versus observed (Obs) concentration-time profile after administration of 30 mg/kg IV [13]

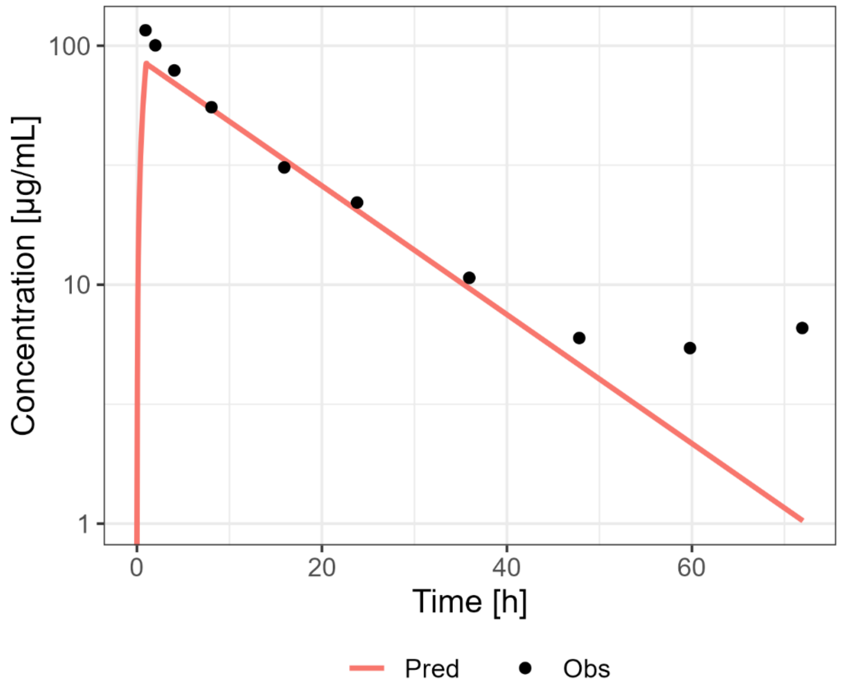

Figure S56 Predicted (Pred) versus observed (Obs) concentration-time profile after administration of 15mg/kg IV [13]

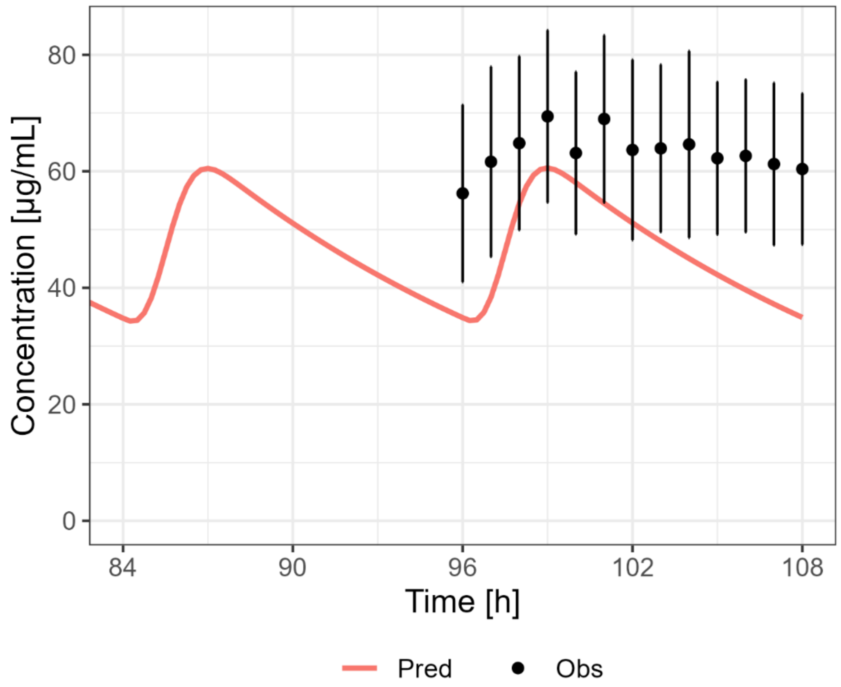

Figure S57 Predicted (Pred) versus observed (Obs) concentration-time profile after administration of 500 mg PO MD [14]

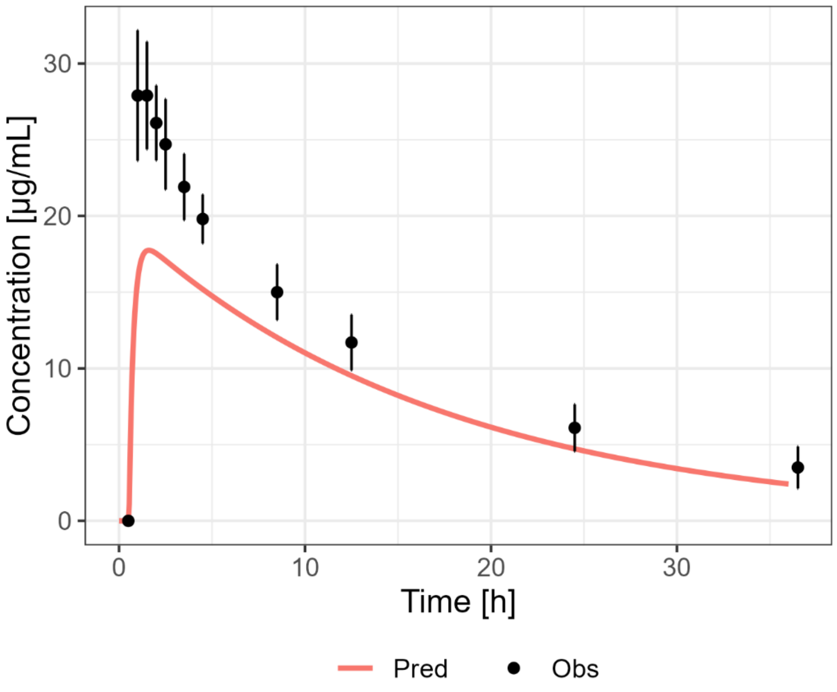

Figure S58 Predicted (Pred) versus observed (Obs) concentration-time profile after administration of 250 mg PO SD [18]

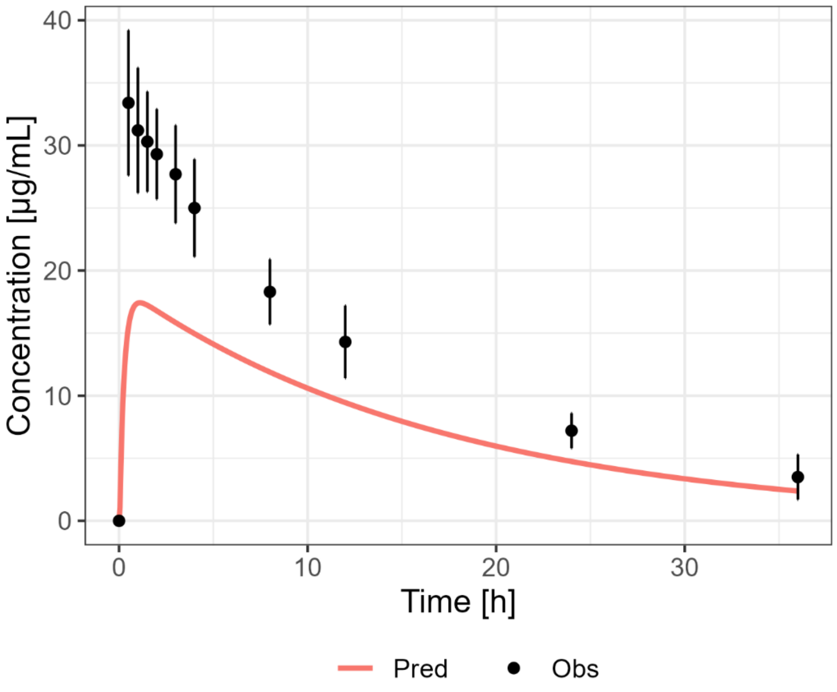

Figure S59 Predicted (Pred) versus observed (Obs) concentration-time profile after administration of250 mg PO syrup SD [18]

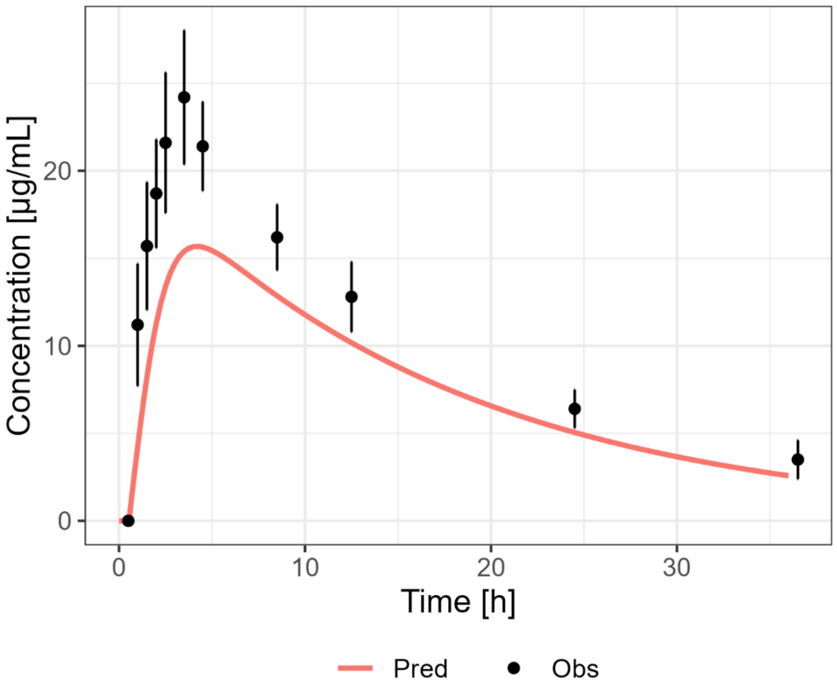

Figure S60 Predicted (Pred) versus observed (Obs) concentration-time profile after administration of 250 mg PO fed SD [18]

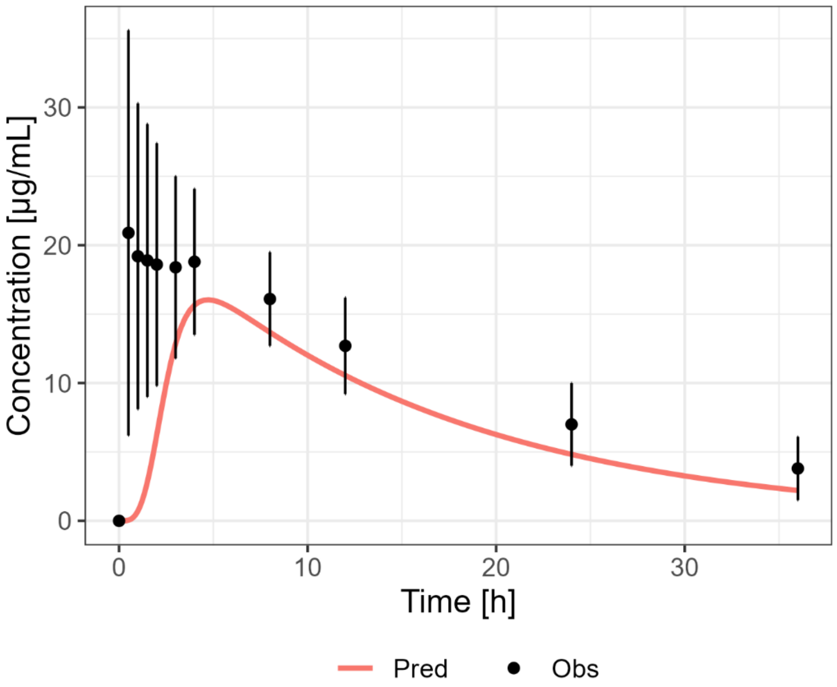

Figure S61 Predicted (Pred) versus observed (Obs) concentration-time profile after administration of 250 mg PO fed SD [18]

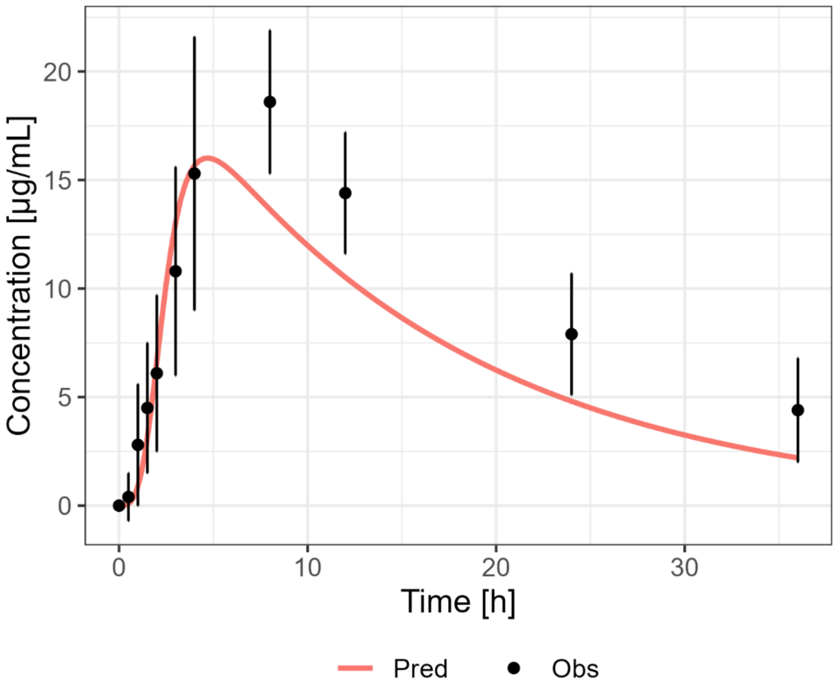

Figure S62 Predicted (Pred) versus observed (Obs) concentration-time profile after administration of 250 mg PO fed SD [18]

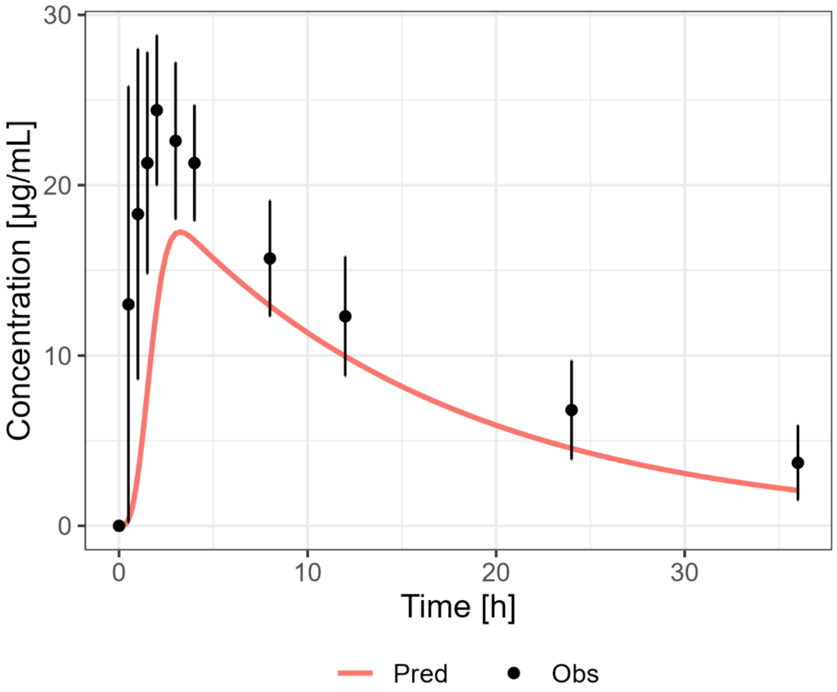

Figure S63 Predicted (Pred) versus observed (Obs) concentration-time profile after administration of 250 mg PO fasted SD [18]

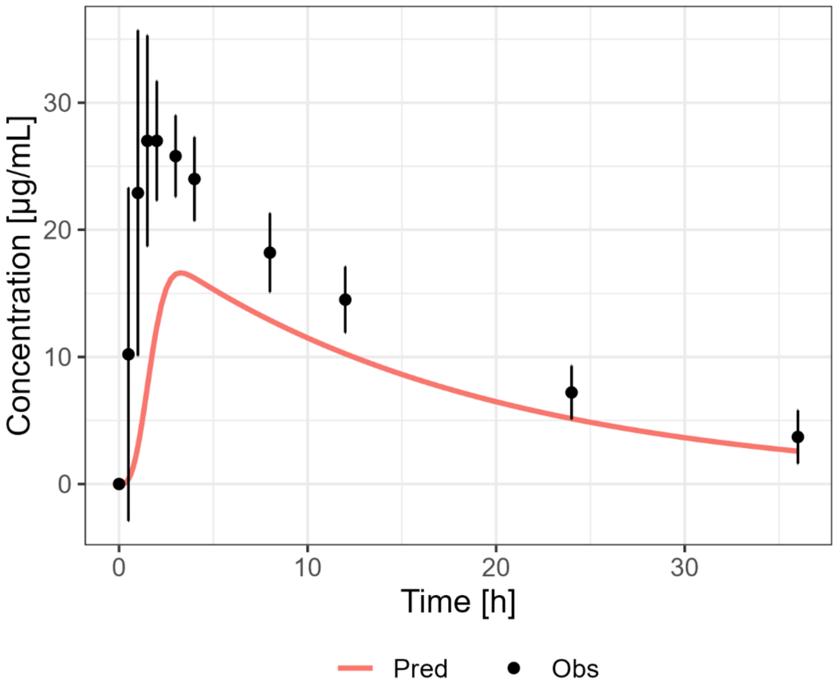

Figure S64 Predicted (Pred) versus observed (Obs) concentration-time profile after administration of 250 mg PO capsule SD [18]

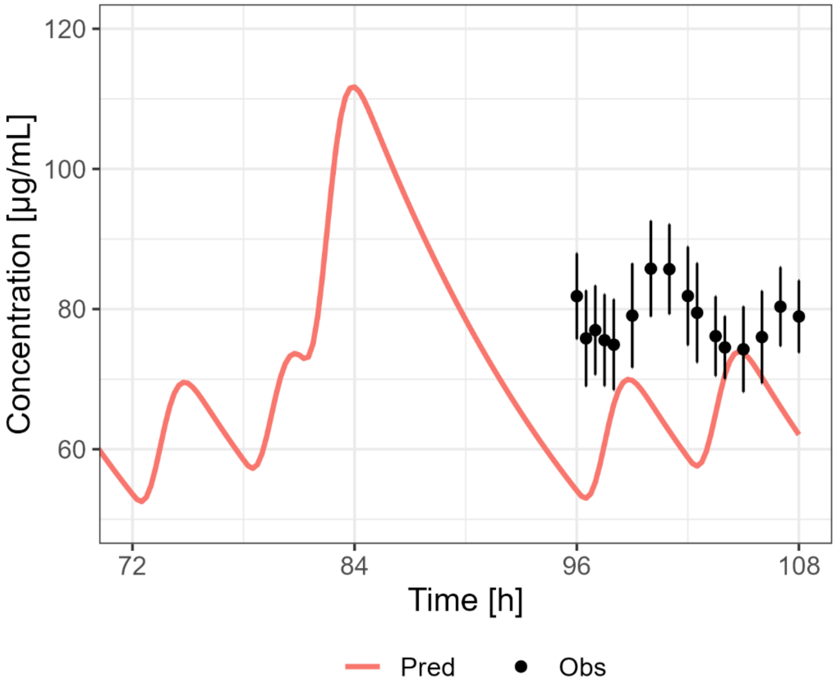

Figure S65 Predicted (Pred) versus observed (Obs) concentration-time profile after administration of 300 mg PO MD [15]

4.3.3 Lactation PBPK model

A sample size of 1000 individuals, was used in each simulation of the virtual lactation population.

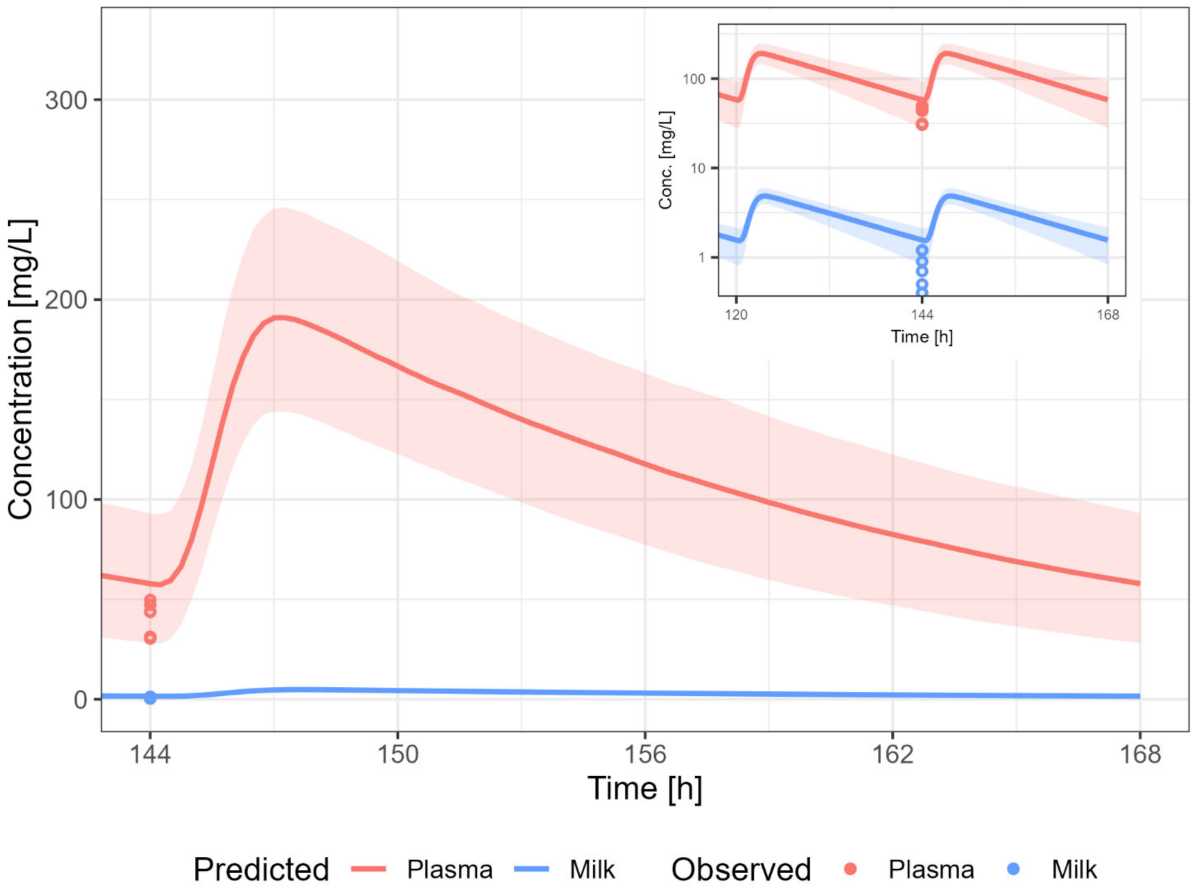

Figure S66 Predicted (Pred) versus observed (Obs) concentration-time profile after administration of 1800 mg/day PO [24]

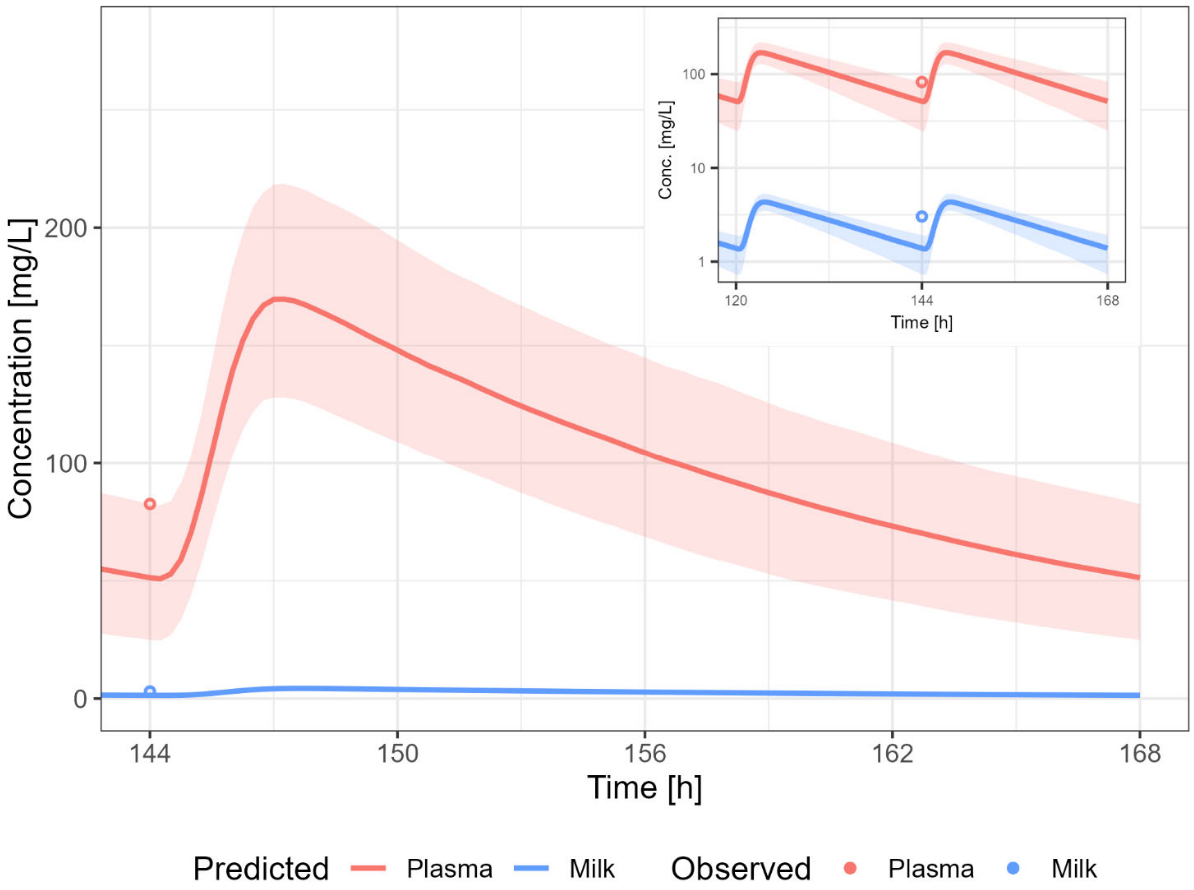

Figure S67 Predicted (Pred) versus observed (Obs) concentration-time profile after administration of 1600 mg/day PO [20]

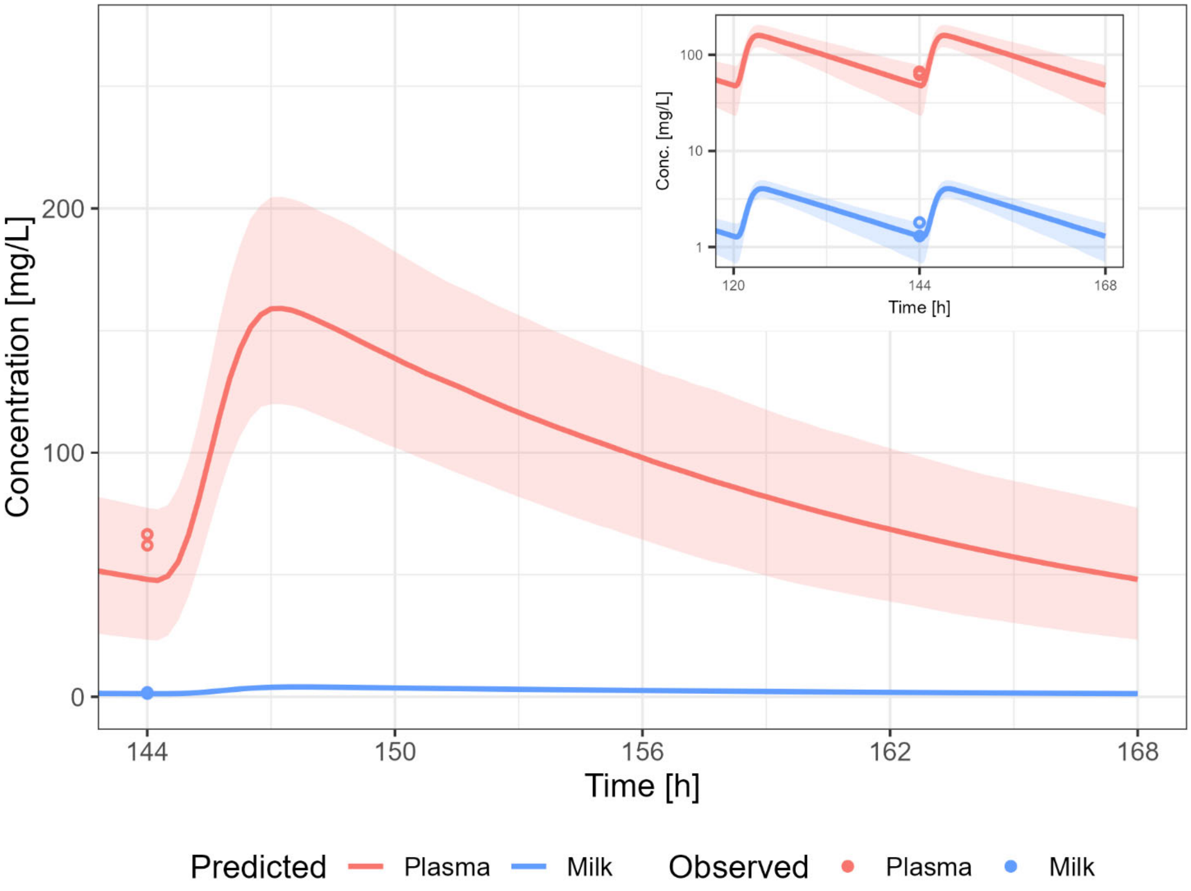

Figure S68 Predicted (Pred) versus observed (Obs) concentration-time profile after administration of 1500 mg/day PO [24]

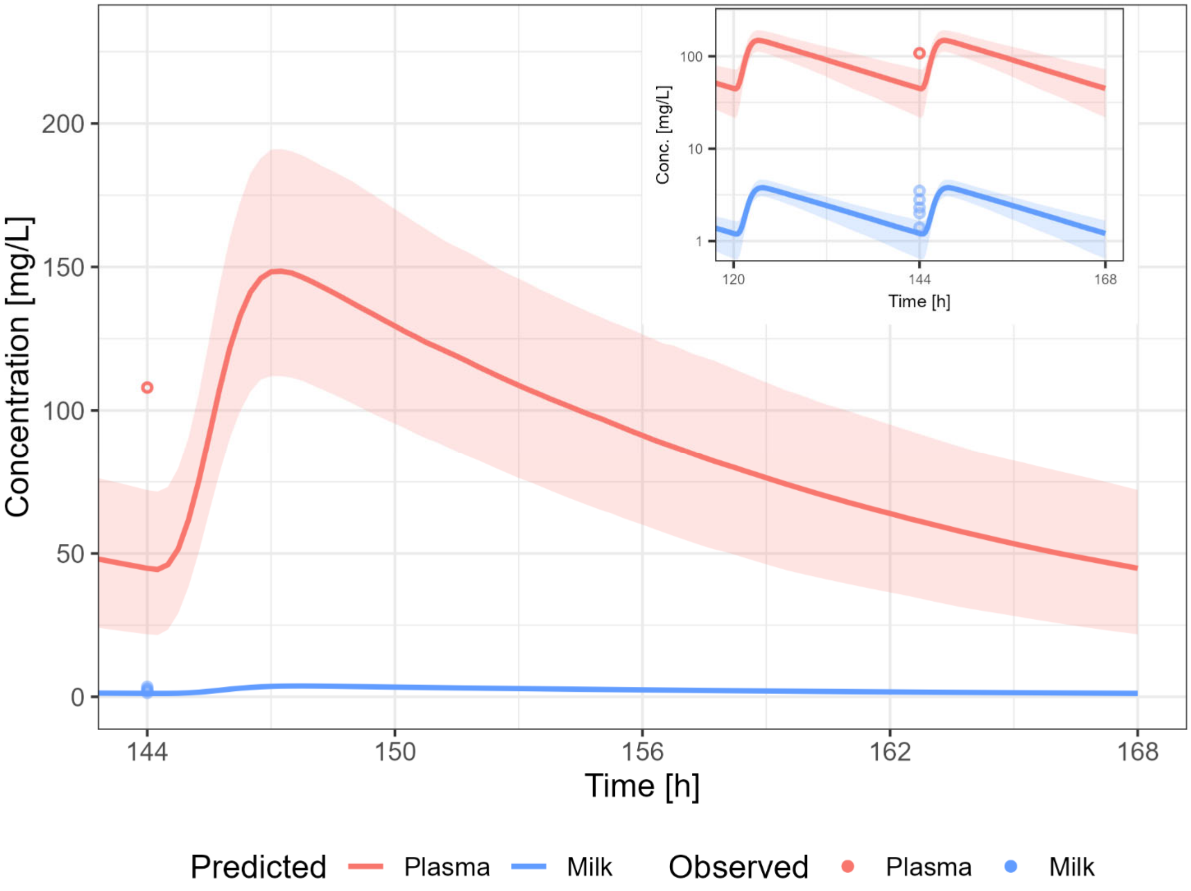

Figure S69 Predicted (Pred) versus observed (Obs) concentration-time profile after administration of 1400 mg/day PO [23]

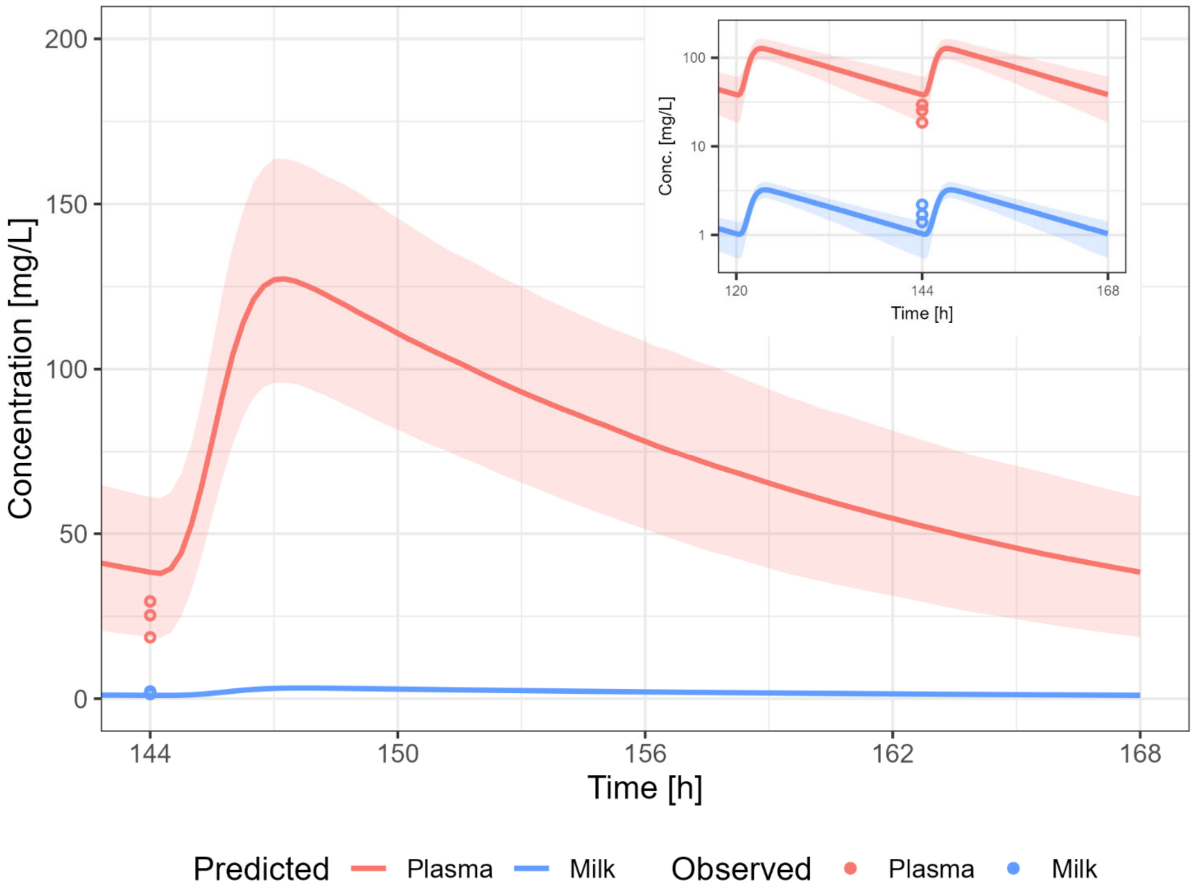

Figure S70 Predicted (Pred) versus observed (Obs) concentration-time profile after administration of 1200 mg/day PO [24]

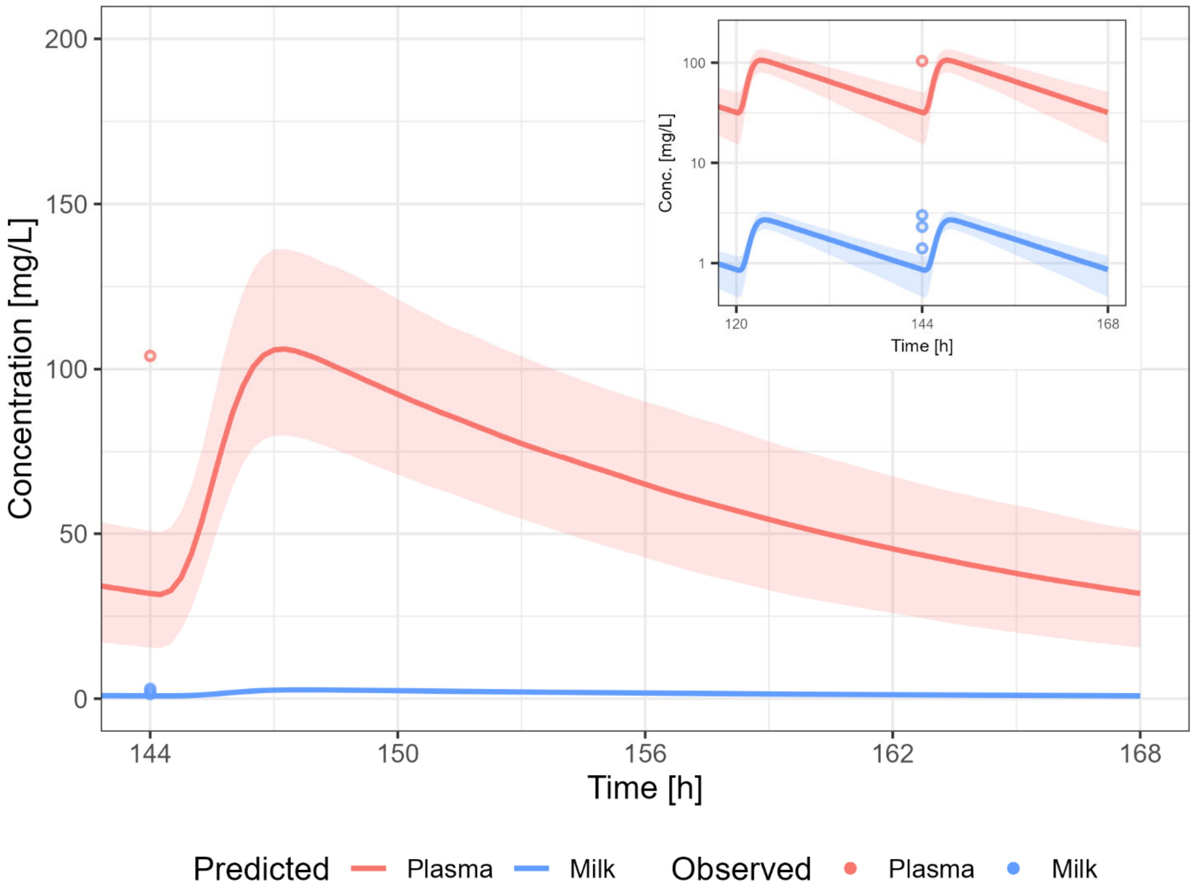

Figure S71 Predicted (Pred) versus observed (Obs) concentration-time profile after administration of 1000 mg/day PO [23]

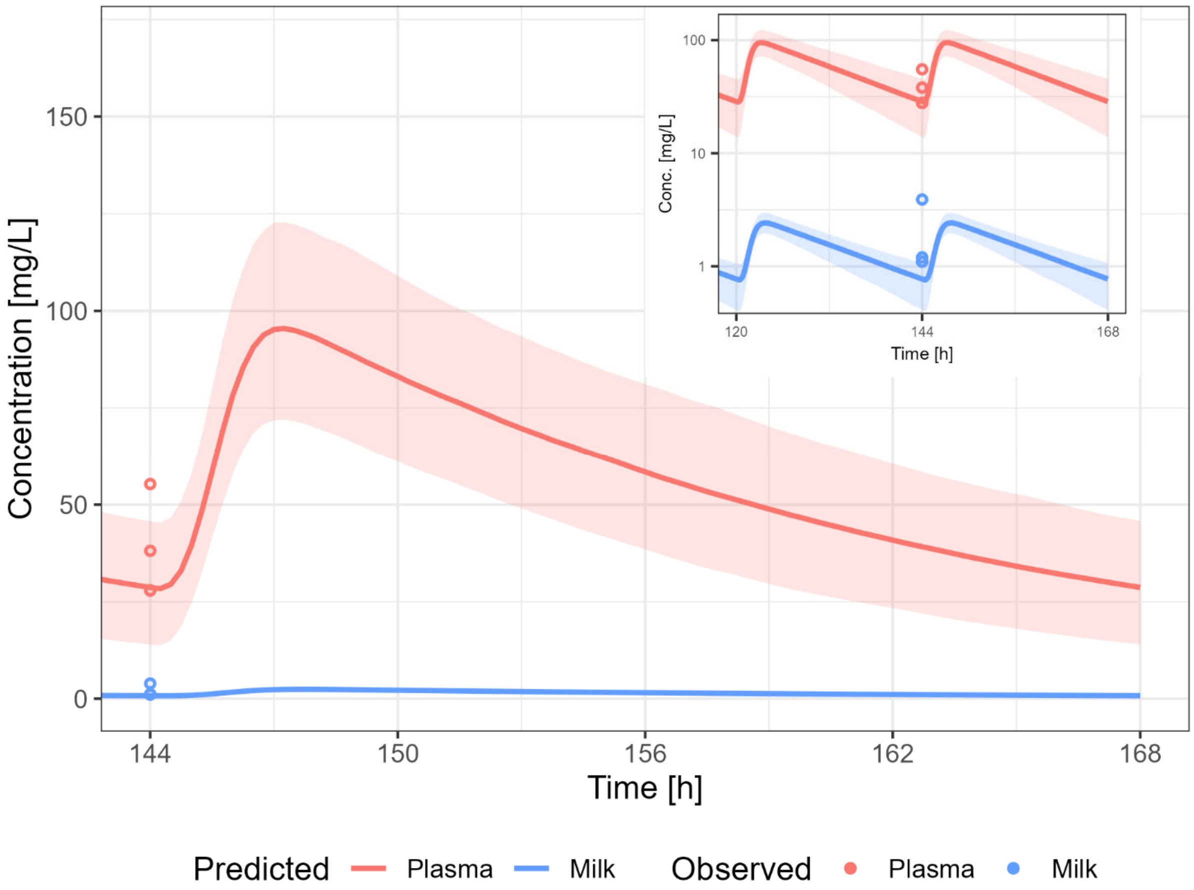

Figure S72 Predicted (Pred) versus observed (Obs) concentration-time profile after administration of 900 mg/day PO [24]

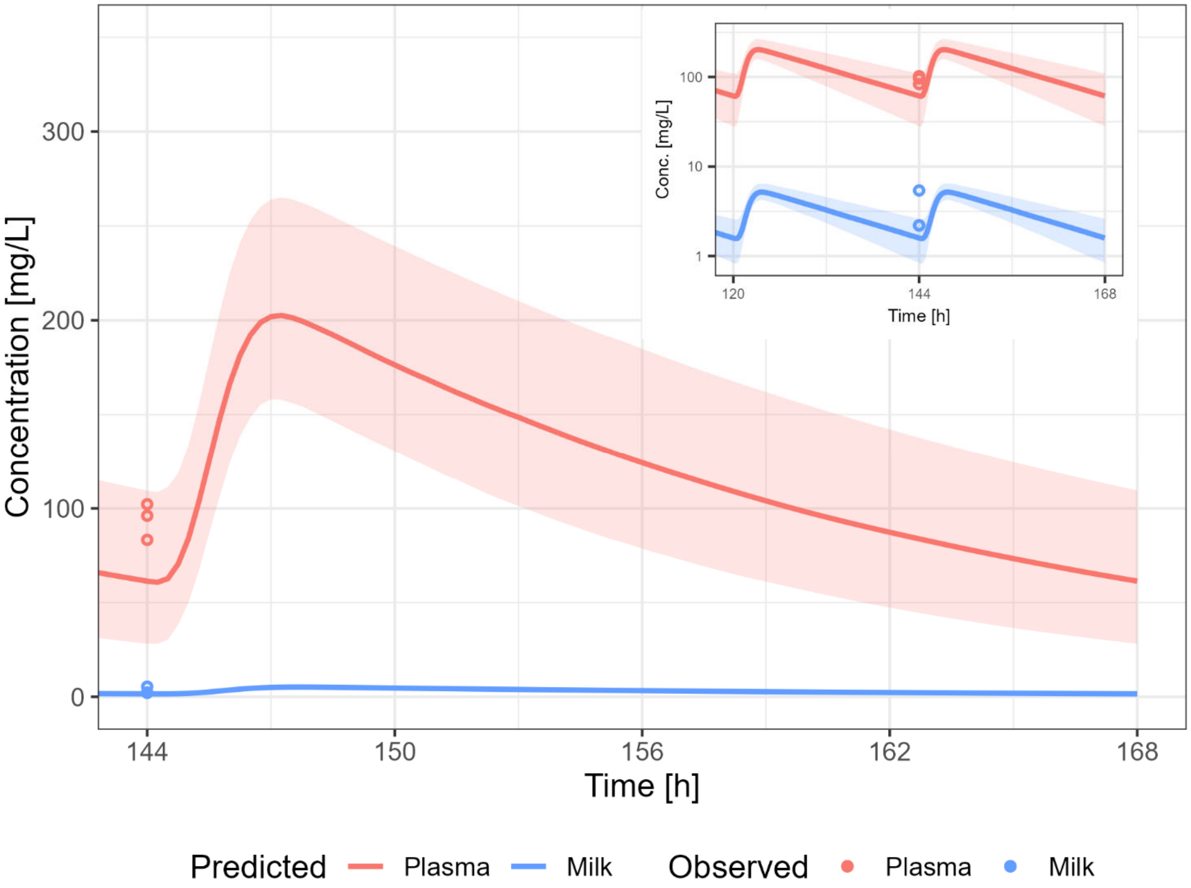

Figure S73 Predicted (Pred) versus observed (Obs) concentration-time profile after administration of 31 mg/kg/day PO [22]

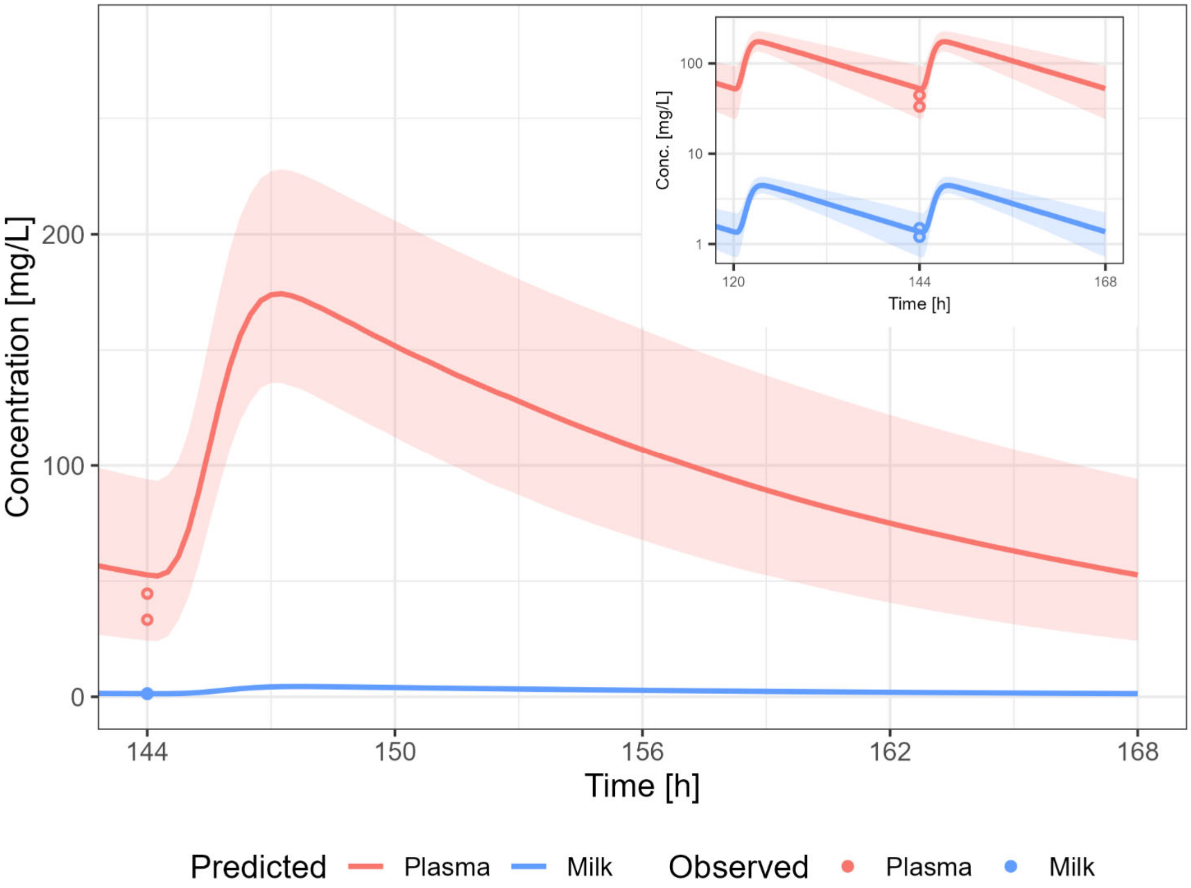

Figure S74 Predicted (Pred) versus observed (Obs) concentration-time profile after administration of 26.7 mg/kg/day PO [22]

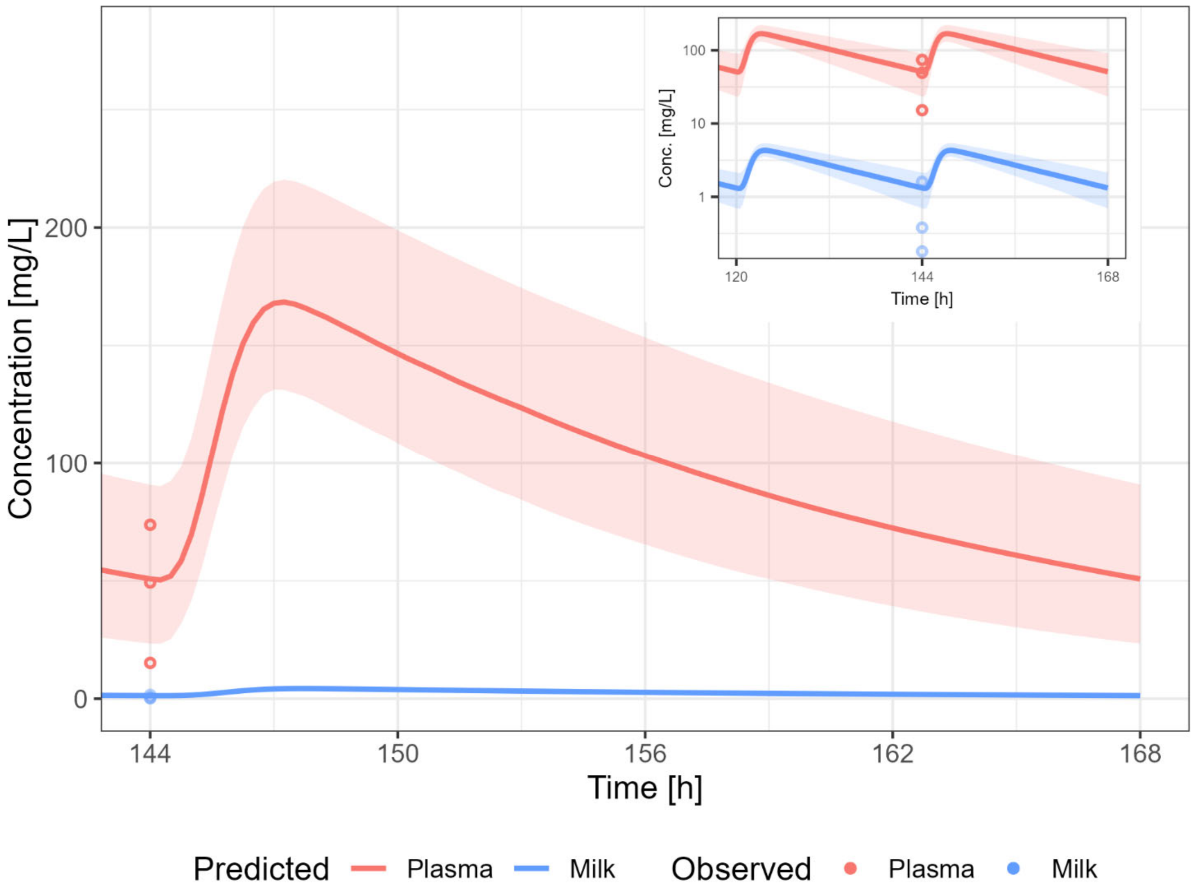

Figure S75 Predicted (Pred) versus observed (Obs) concentration-time profile after administration of 25.8 mg/kg/day PO [22]

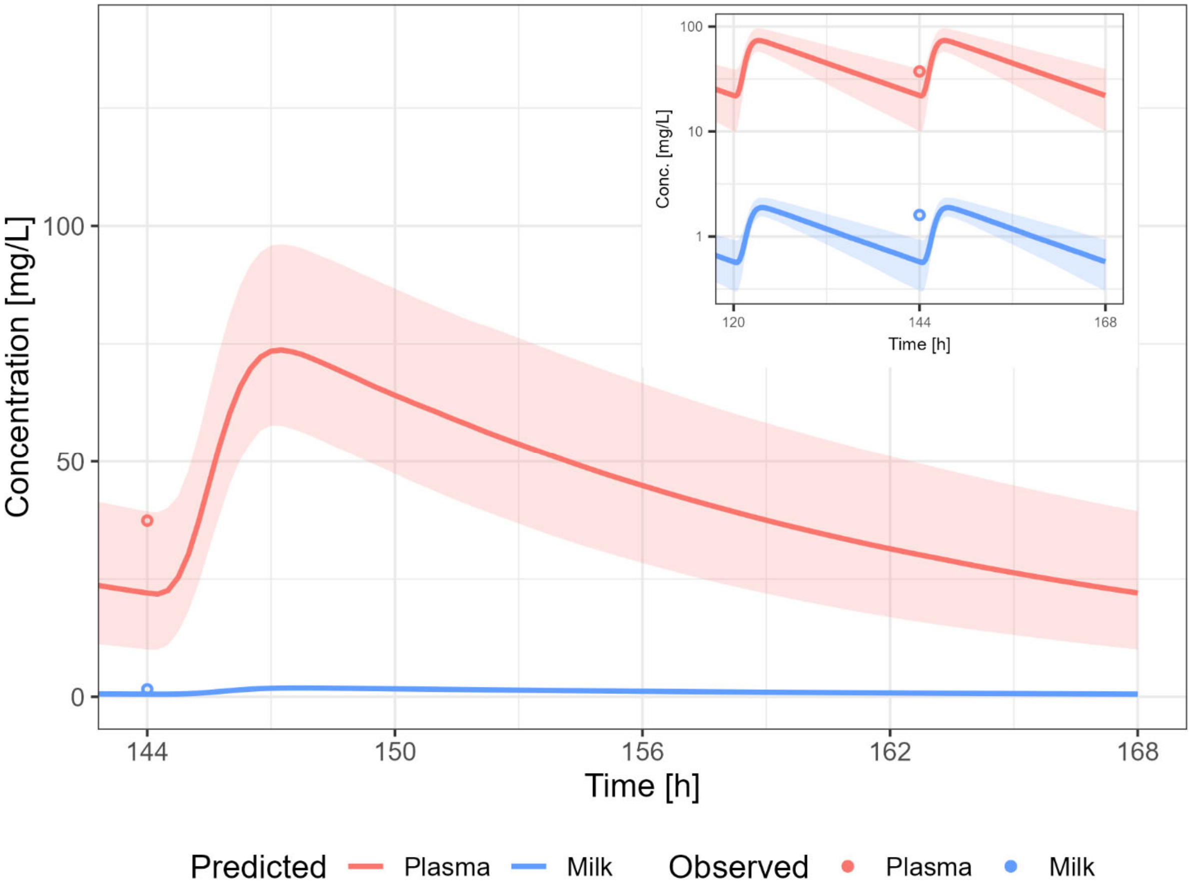

Figure S76 Predicted (Pred) versus observed (Obs) concentration-time profile after administration of 11.3 mg/kg/day PO [21]

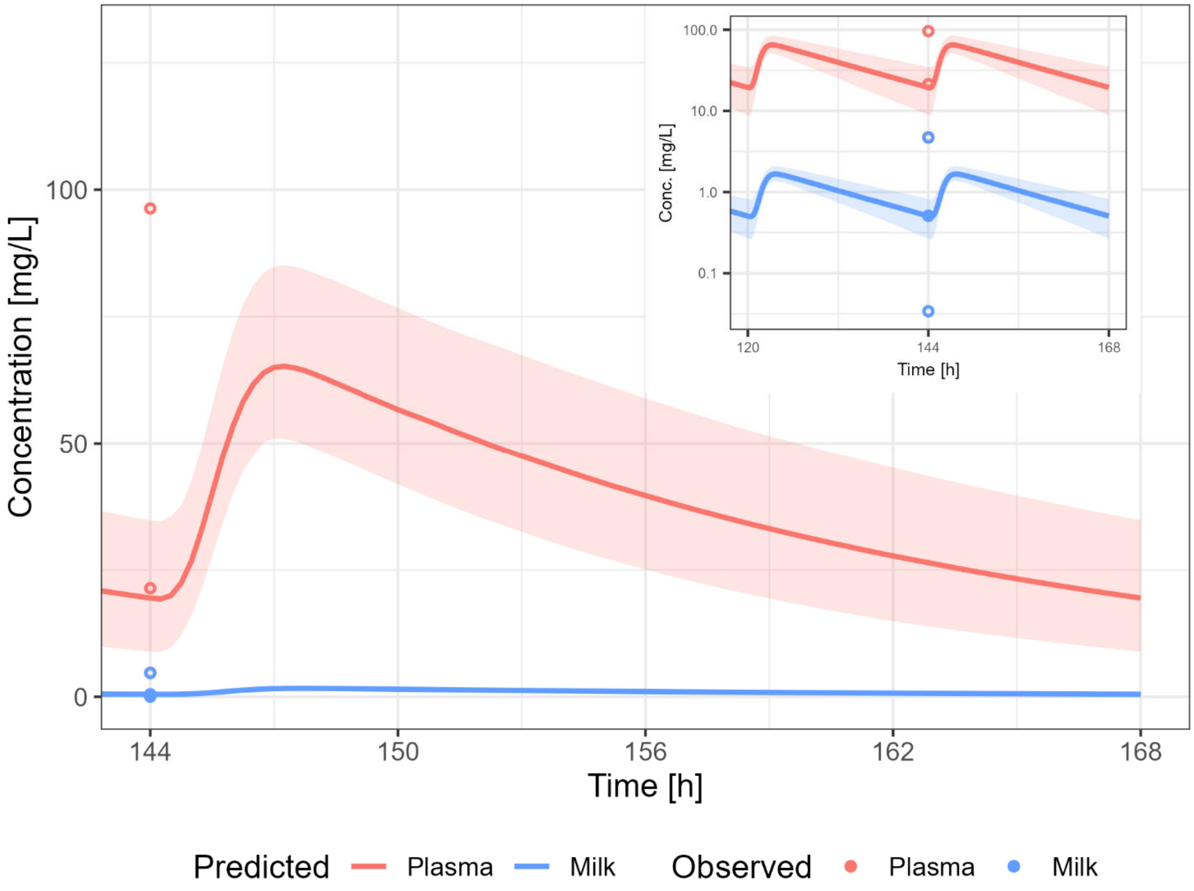

Figure S77 Predicted (Pred) versus observed (Obs) concentration-time profile after administration of 10 mg/kg/day PO [22]

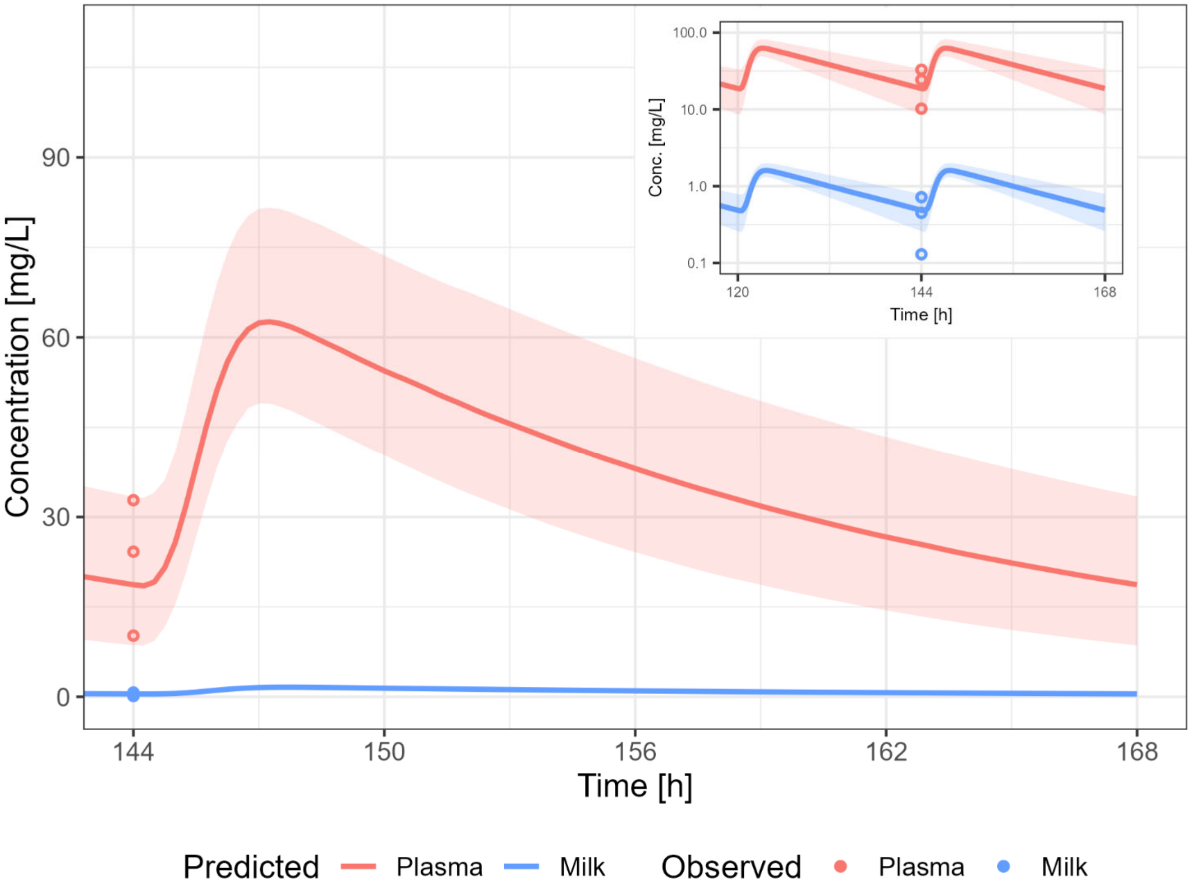

Figure S78 Predicted (Pred) versus observed (Obs) concentration-time profile after administration of 9.6 mg/kg/day PO [22]

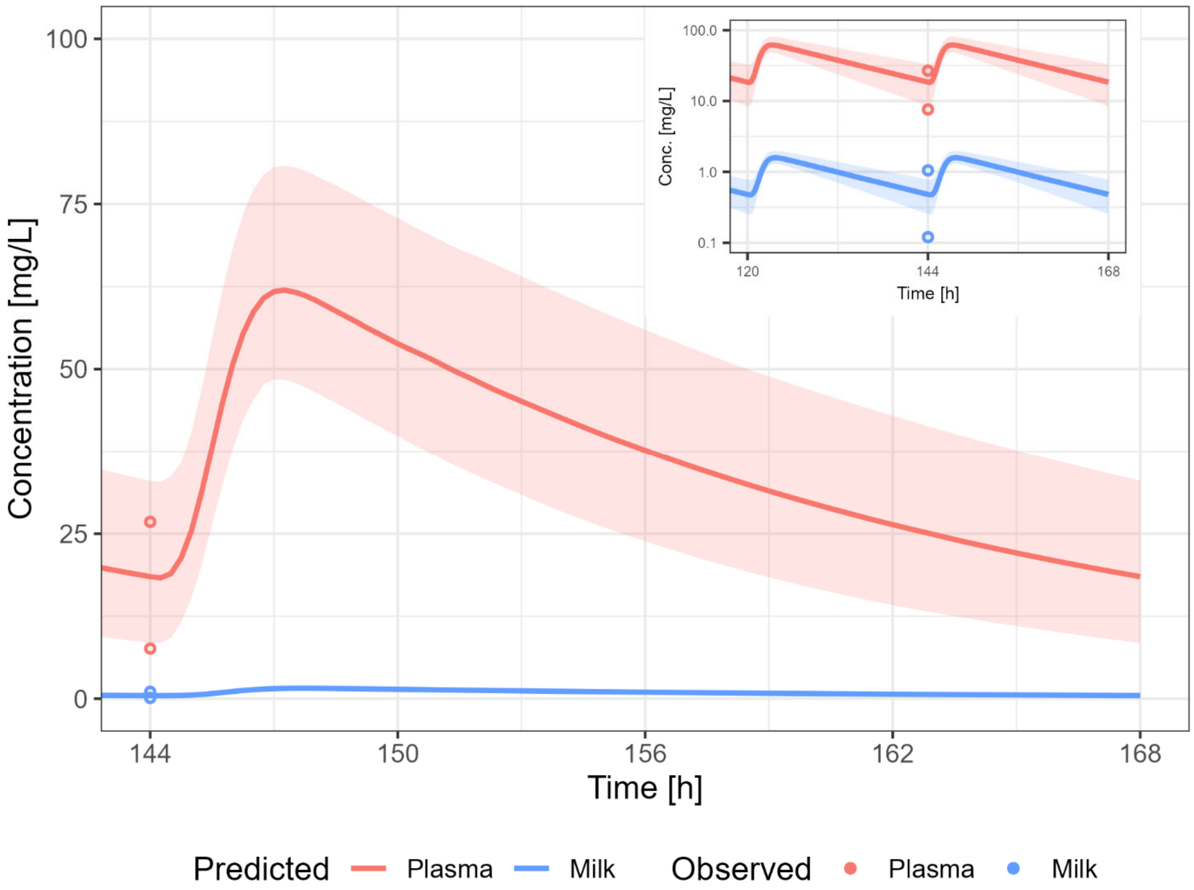

Figure S79 Predicted (Pred) versus observed (Obs) concentration-time profile after administration of 9.5 mg/kg/day PO [22]

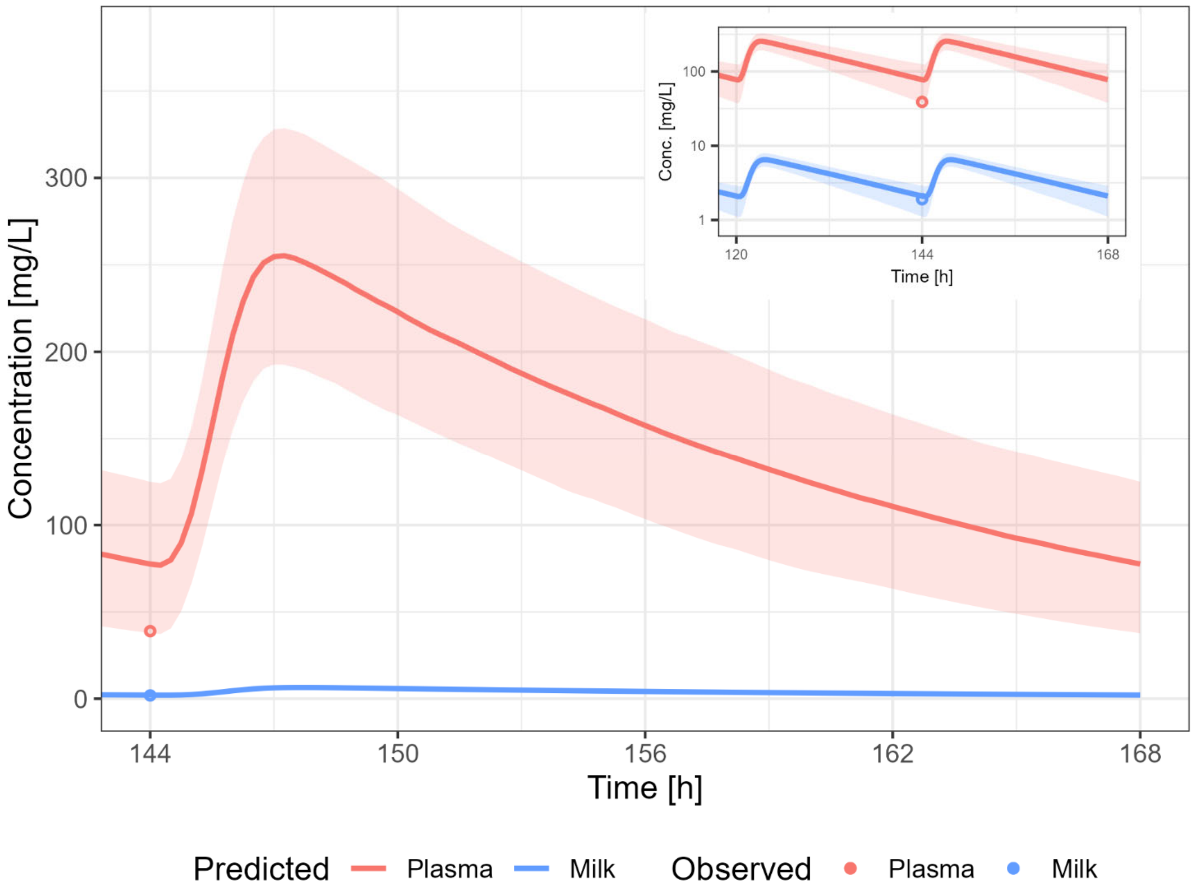

Figure S80 Predicted (Pred) versus observed (Obs) concentration-time profile after administration of 2400 mg/day PO [24]

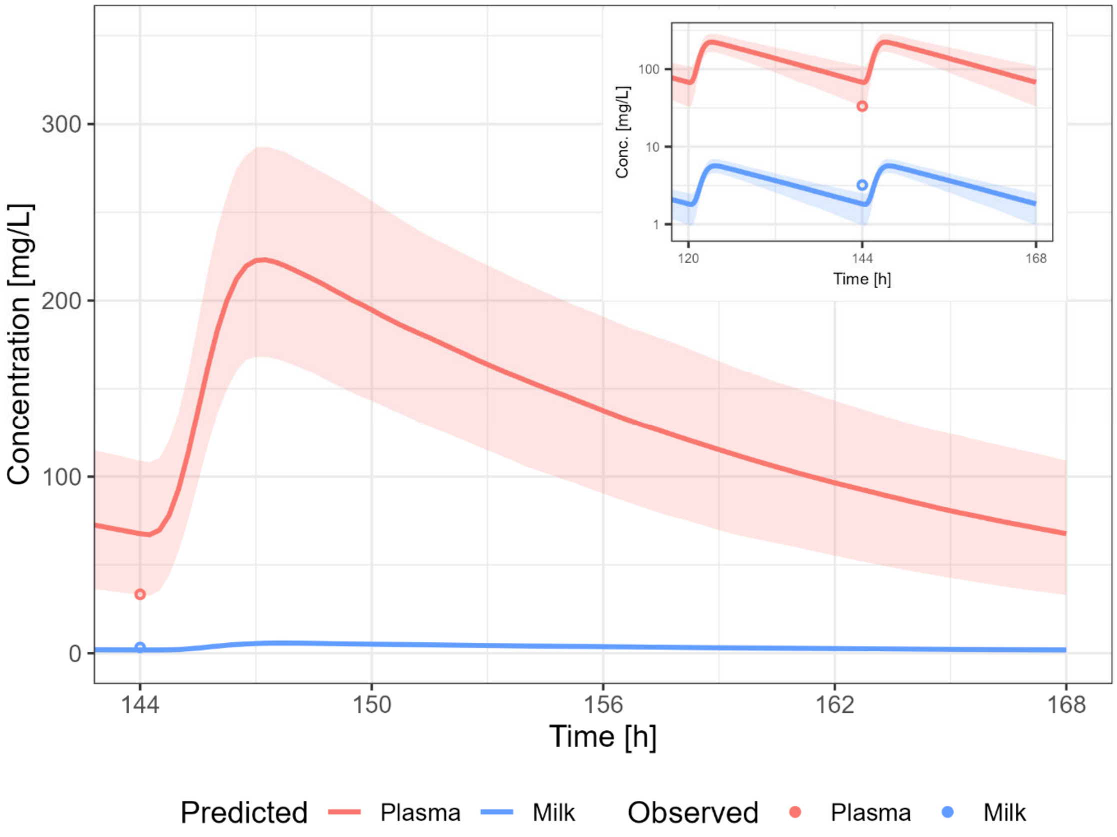

Figure S81 Predicted (Pred) versus observed (Obs) concentration-time profile after administration of 2100 mg/day PO [24]

A dosing regimen of 2100 mg/day was assumed to calculate the milk transfer of valproic acid.

Dosing interval: 24 h	Plasma	Milk
Cmax (mg/L)	223.1	5.66
AUC (mg*h/L)	3223.29	83.73
Cave (mg/L)	134.30	3.49

M/P ratio = 0.03

4.4 Estimated infant dosage

A maternal dosing regimen of 2100 mg daily was used to calculate the infant dosage. The daily infant dosage and relative infant dose (RID) for 3 months old infant were calculated using a milk intake of 150 mL/kg/day. The daily infant dosage was 0.52 mg/kg/day (RID: 1.50 %) or 0.85 mg/kg/day (RID: 2.44 %) based on the average steady-state concentration and maximum concentration in human milk, respectively.

5. Discussion

First, the reference PBPK model was developed and evaluated. Evaluation of the predictive performance showed that the reference PBPK model for VPA was able to capture the

pharmacokinetic behavior of the medicines in healthy volunteers and/or patients in 97 % of the simulations.

For Nitsche and Mascher, the plasma-concentration of 1 subject was not predicted well when 900 mg was given as 2 capsules [11]. Importantly, the prediction for the same subject after 900 mg as three capsules or after administration of 1000 mg were good. In addition, the plasma concentration of the 5 other subjects were predicted within 2-fold for the three dosing regimens. The observed data also showed that the trough concentrations of the last 2 doses before administration of the last dose decline, which might be explained by patient-specific autoinduction processes or a missed dose. Another study with different doses of intravenous administration was not predicted within 2-fold prediction error on AUC for one dose level [13]. this study did not find an increase in plasma concentration with an increase in dose. Moreover, the AUC even declines when the dose is increased from 90 to 120 mg/kg and from 140 to 150 mg/kg. It is not clear what is the cause for these results, potentially complex non-linear kinetics are involved for valproic acid.

Next, the PBPK model was extended to a lactation PBPK model. The PBPK model results in an acceptable prediction of the human milk concentrations, with most datapoints within the 5-95th percentile of the population prediction. Some datapoints are slightly above the 5-95th percentile, but this is most likely due to the fact that we assumed all samples to be trough samples when there was no information about the time respective to the last dose. In addition, several studies did report the average dose of VPA.

The predicted M/P ratio was within the range of observed M/P ratios.

The calculated infant dosage of VPA via breastfeeding was low, especially when compared to the maternal daily dosage.

6. Conclusions

The herein presented PBPK model adequately describes the PK of valproic acid in adults including breastfeeding women. In particular, it applies quantitative metabolism by glucuronidation, beta-oxidation, cytochrome P450 oxidation and renal clearance. The PBPK model was able to predict the human milk concentrations of valproic acid (M/P ratio: 0.03). The daily infant dosage was 0.52 mg/kg/day (RID: 1.50 %) or 0.85 mg/kg/day (RID: 2.44 %) based on the average steady-state concentration and maximum concentration in human milk, respectively.

7. List of Appendix and Supplementary Materials

Supplementary material 1 – ObsDataPK_OSP_reference_valproic acid
Supplementary material 2 – ObsDataPK_OSP_lactation_valproic acid
Supplementary material 3 – Valproic acid.pksim5

8. References

1. Valproic Acid | DrugBank Online Available online: https://go.drugbank.com/drugs/DB00313 (accessed on 18 July 2022).
2. BCFI Valproïnezuur Available online: https://www.bcfi.be/nl/chapters/11?matches=Valproïnezuur%7Cvalproïnezuur&frag=8722.
3. Conner, T.M.; Nikolian, V.C.; Georgoff, P.E.; Pai, M.P.; Alam, H.B.; Sun, D.; Reed, R.C.; Zhang, T. Physiologically Based Pharmacokinetic Modeling of Disposition and Drug-Drug Interactions for Valproic Acid and Divalproex. *Eur. J. Pharm. Sci.* **2018**, *111*, 465–481, doi:10.1016/j.ejps.2017.10.009.
4. Dallmann, A.; Solodenko, J.; Ince, I.; Eissing, T. Applied Concepts in PBPK Modeling: How to Extend an Open Systems Pharmacology Model to the Special Population of Pregnant Women. *CPT Pharmacometrics Syst. Pharmacol.* **2018**, *7*, 419–431, doi:10.1002/psp4.12300.
5. Dallmann, A.; Himstedt, A.; Solodenko, J.; Ince, I.; Hempel, G.; Eissing, T. Integration of Physiological Changes during the Postpartum Period into a PBPK Framework and Prediction of Amoxicillin Disposition before and Shortly after Delivery. *J. Pharmacokinet. Pharmacodyn.* **2020**, *47*, 341–359, doi:10.1007/s10928-020-09706-z.
6. Job, K.M.; Dallmann, A.; Parry, S.; Saade, G.; Haas, D.M.; Hughes, B.; Berens, P.; Chen, J.; Fu, C.; Humphrey, K.; et al. Development of a Generic Physiologically-Based Pharmacokinetic Model for Lactation and Prediction of Maternal and Infant Exposure to Ondansetron via Breast Milk. *Clin. Pharmacol. Ther.* **2022**, *111*, 1111–1120, doi:10.1002/cpt.2530.
7. Koshimichi, H.; Ito, K.; Hisaka, A.; Honma, M.; Suzuki, H. Analysis and Prediction of Drug Transfer into Human Milk Taking into Consideration Secretion and Reuptake Clearances across the Mammary Epithelia. *Drug Metab. Dispos.* **2011**, *39*, 2370–2380, doi:10.1124/dmd.111.040972.
8. Cloyd, J.C.; Dutta, S.; Cao, G.; Walch, J.K.; Collins, S.D.; Granneman, G.R. Valproate Unbound Fraction and Distribution Volume Following Rapid Infusions in Patients with Epilepsy. *Epilepsy Res.* **2003**, *53*, 19–27, doi:10.1016/S0920-1211(02)00251-6.
9. Argikar, U.A.; Remmel, R.P. Effect of Aging on Glucuronidation of Valproic Acid in Human Liver Microsomes and the Role of UDP-Glucuronosyltransferase UGT1A4, UGT1A8, and UGT1A10. *Drug Metab. Dispos.* **2009**, *37*, 229–236, doi:10.1124/dmd.108.022426.
10. Atkinson, U.C.; Begg, E.J.E.J.; Atkinson, H.C.; Begg, E.J.E.J. Prediction of Drug Distribution into Human Milk from Physicochemical Characteristics. *Clin. Pharmacokinet.* **1990**, *18*, 151–167, doi:10.2165/00003088-199018020-00005.
11. Nitsche, V.; Mascher, H. The Pharmacokinetics of Valproic Acid After Oral and Parenteral Administration in Healthy Volunteers. *Epilepsia* **1982**, *23*, 153–162, doi:10.1111/j.1528-1157.1982.tb05063.x.
12. Perucca, E.; Gatti, G.; Frigo, G.M.; Crema, A. Pharmacokinetics of Valproic Acid after Oral and Intrevenous Administration. *Br. J. Clin. Pharmacol.* **1978**, *5*, 313–318.

13. Georgoff, P.E.; Nikolian, V.C.; Bonham, T.; Pai, M.P.; Tafatia, C.; Halaweish, I.; To, K.; Watcharotone, K.; Parameswaran, A.; Luo, R.; et al. Safety and Tolerability of Intravenous Valproic Acid in Healthy Subjects: A Phase I Dose-Escalation Trial. *Clin. Pharmacokinet.* **2018**, *57*, 209–219, doi:10.1007/s40262-017-0553-1.
14. Dulac, O.; Alvarez, J.-C. Bioequivalence of a New Sustained-Release Formulation of Sodium Valproate, Valproate Modified-Release Granules, Compared with Existing Sustained-Release Formulations after Once- or Twice-Daily Administration. *Pharmacotherapy* **2005**, *25*, 35–41, doi:15767218.
15. Rha, J.H.; Jang, I.J.; Lee, K.H.; Chong, W.S.; Shin, S.G.; Lee, N.; Myung, H.J. Pharmacokinetic Comparison of Two Valproic Acid Formulations--a Plain and a Controlled Release Enteric-Coated Tablets. *J. Korean Med. Sci.* **1993**, *8*, 251–256, doi:10.3346/jkms.1993.8.4.251.
16. Ibarra, M.; Vázquez, M.; Fagiolino, P.; Derendorf, H. Sex Related Differences on Valproic Acid Pharmacokinetics after Oral Single Dose. *J. Pharmacokinet. Pharmacodyn.* **2013**, *40*, 479–486, doi:10.1007/s10928-013-9323-3.
17. Pokrajac, M.; Miljković, B.; Varagić, V.M.; Lević, Z. Pharmacokinetic Interaction between Valproic Acid and Phenobarbital. *Biopharm. Drug Dispos.* **1993**, *14*, 81–86, doi:10.1002/bdd.2510140108.
18. Chun, A.H.; Hoffman, D.J.; Friedmann, N.; Carrigan, P.J. Bioavailability of Valproic Acid under Fasting/Nonfasting Regimens. *J. Clin. Pharmacol.* **1980**, *20*, 30–36, doi:10.1002/j.1552-4604.1980.tb01663.x.
19. Ohdo, S.; Nakano, S.; Ogawa, N. Circadian Changes of Valproate Kinetics Depending on Meal Condition in Humans. *J. Clin. Pharmacol.* **1992**, *32*, 822–826, doi:10.1002/j.1552-4604.1992.tb03889.x.
20. Alexander, F.W. Sodium Valproate and Pregnancy. *Arch. Dis. Child.* **1979**, *54*, 240, doi:10.1136/adc.54.3.240.
21. Kacirova, I.; Grundmann, M.; Brozmanova, H. Valproic Acid Concentrations in Nursing Mothers, Mature Milk, and Breastfed Infants in Monotherapy and Combination Therapy. *Epilepsy Behav.* **2019**, *95*, 112–116, doi:10.1016/j.yebeh.2019.04.002.
22. Nau, H.; Rating, D.; Koch, S.; Häuser, I.; Helge, H. Valproic Acid and Its Metabolites: Placental Transfer, Neonatal Pharmacokinetics, Transfer via Mother's Milk and Clinical Status in Neonates of Epileptic Mothers. *J Pharmacol Exp Ther.* **1981**, *219*, 768–777.
23. Tsuru, N.; Maeda, T.; Tsuruoka, M. Three Cases of Delivery under Sodium Valproate—Placental Transfer, Milk Transfer and Probable Teratogenicity of Sodium Valproate—. *Psychiatry Clin. Neurosci.* **1988**, *42*, 89–96, doi:10.1111/j.1440-1819.1988.tb01960.x.
24. von Unruh, G.E.; Froescher, W.; Hoffmann, F.; Niesen, M. Valproic Acid in Breast Milk. *Ther. Drug Monit.* **1984**, *6*, 272–276, doi:10.1097/00007691-198409000-00003.
25. I., K.; M., G.; H., B.; Kacirova, I.; Grundmann, M.; Brozmanova, H.; I., K.; M., G.; H., B. Valproic Acid Concentrations in Nursing Mothers, Mature Milk, and Breastfed Infants in Monotherapy and Combination Therapy. *Epilepsy Behav.* **2019**, *95*, 112–116, doi:10.1016/j.yebeh.2019.04.002 LK - http://limo.libis.be/resolver?&sid=EMBASE&issn=15255069&id=doi:10.1016%2Fj.yebeh.2019.04.002&atitle=Valproic+acid+concentrations+in+nursing+mothers%2C+mature+milk%2C+and+breastfed+infants+in+monotherapy+and+combination+therapy&stitle=Epilepsy+Behav.&title=Epilepsy+and+Behavior&volume=95&issue=&spage=112&epage=116&aulast=Kacirova&aufirst=Ivana&auinit=I.&aufull=Kacirova+I.&coden=EBPEA&isbn=&pages=112-116&date=2019&auinit1=I&auinitm=.

26. Nau, H.; Rating, D.; Koch, S.; Häuser, I.; Helge, H.; H., N.; D., R.; S., K.; Nau, H.; Rating, D.; et al. Valproic Acid and Its Metabolites: Placental Transfer, Neonatal Pharmacokinetics, Transfer via Mother's Milk and Clinical Status in Neonates of Epileptic Mothers. *J. Pharmacol. Exp. Ther.* **1981**, *219*, 768–777.
